# Supplementary material for: Living with relatives offsets the harm caused by pathogens in natural populations
Source: eLife. 2021 Jul 26;10:e66649. doi: 10.7554/eLife.66649 (PMC8313236; doi:10.7554/eLife.66649)
Supplement: Supplementary file 1. — Tables S1-19 can be found in excel format at the open science framework DOI 10.17605/OSF.IO/Q3ANE. Table S1: The references screened for effect sizes of within-group relatedness on mortality and pathogen abundance and whether they were included in analyses. Table S2: Data used for analysis of the effect of within-group relatedness on mortality and pathogen abundance (r). Table S3: Data on whether species typically live with kin or non-kin. Table S4: Data used for analysis of the effect of within-group relatedness on variance in mortality and pathogen abundance (LnCVR). Table S5: The overall effect of within-group relatedness on mortality and pathogen abundance on Zr. Table S6: The effect of pathogen presence and whether mortality or pathogen abundance was measured on Zr. Table S7: The effect of pathogen manipulation on Zr. Table S8: The effect of relatedness manipulations and pathogen presence on Zr. Table S9: The effect of kin structure and pathogen presence measures on Zr. Table S10: The overall effect of within-group relatedness on variance (LnCVR) mortality and pathogen abundances. Table S11: The effect of pathogen manipulation on LnCVR. Table S12: The effect of pathogen manipulation and whether mortality or pathogen abundance was measured on LnCVR. Table S13: The effect of relatedness manipulations and pathogen presence on LnCVR. Table S14: The effect of kin structure and pathogen presence measures on Zr excluding potential effects of inbreeding. Table S15: Effect of parasite manipulations and fitness measures on LnCVR excluding potential effects of inbreeding. Table S16: Laboratory effects on Zr. Table S17: Laboratory effects on LnCVR. Table S18: The sensitivity of analyses on Zr to the statistical techniques used in primary studies. Table S19: Data on within-group relatedness on mortality in ostrich chicks. [file elife-66649-supp1.zip › SIinformation.html]

| 5170 | 2020 | 44113 | Jimenez-Becerril, MF; Hernandez-Delgado, S; Solis-Oba, M; Prieto, JMG | 2018 | Analysis of mitochondrial genetic diversity of Ustilago maydis in Mexico | The current understanding of the genetic diversity of the phytopathogenic fungus Ustilago maydis is limited. To determine the genetic diversity and structure of U. maydis, 48 fungal isolates were analyzed using mitochondrial simple sequence repeats (SSRs). Tumours (corn smut or `huitlacoche') were collected from different Mexican states with diverse environmental conditions. Using bioinformatic tools, five microsatellites were identified within intergenic regions of the U. maydis mitochondrial genome. SSRMUM4 was the most polymorphic marker. The most common repeats were hexanucleotides. A total of 12 allelic variants were identified, with a mean of 2.4 alleles per locus. An estimate of the genetic diversity using analysis of molecular variance (AMOVA) revealed that the highest variance component is within states (84%), with moderate genetic differentiation between states (16%) (FST = 0.158). A dendrogram generated using the unweighted paired-grouping method with arithmetic averages (UPGMA) and the Bayesian analysis of population structure grouped the U. maydis isolates into two subgroups (K = 2) based on their shared SSRs. </td> <td style="text-align:left;"> NA </td> <td style="text-align:left;"> no </td> <td style="text-align:left;"> No good fitness measure and measure host GD </td> <td style="text-align:left;"> NA </td> <td style="text-align:left;"> no </td> <td style="text-align:left;"> no </td> </tr> <tr> <td style="text-align:left;"> 5171 </td> <td style="text-align:left;"> 2020 </td> <td style="text-align:left;"> 44113 </td> <td style="text-align:left;"> Joel, KA; Runo, S; Muchugi, A </td> <td style="text-align:left;"> 2018 </td> <td style="text-align:left;"> Genetic diversity and virulence of Striga hermonthica from Kenya and Uganda on selected sorghum varieties </td> <td style="text-align:left;"> Parasitic weeds pose a severe problem in agricultural production, causing massive crop losses in many regions of the world and especially Africa. One example to be considered the most significant biological constraint to food production in sub-Saharan Africa (SSA) is Striga hermonthica, the most widespread among the Striga species in the semi-arid tropical African zones. The wide geographical distribution set conditions for genetically structured populations. The genetic variations among the weed populations allow for the quick breakdown of resistance in crops hence making control of the weed difficult. Efficient and effective control of S. hermonthica demands knowledge on inherent genetic variability within local and regional races of the weed. However, the genetic diversity and virulence of S. hermonthica ecotypes in Kenya and Uganda on selected sorghum varieties remain unknown. This study aimed at evaluating the genetic diversity among seven S. hermonthica populations from locations in Kenya and Uganda using 5 primer sets of Expressed Sequence Tags-Simple Sequence Repeats (EST-SSR). The genetic diversity was moderate as shown by the Nei's genetic distance values. AMOVA measured low genetic differentiation among the populations. This study also investigated twelve sorghum varieties for their response to S. hermonthica infection. The result demonstrated that the varieties resistance responses to S. hermonthica varied widely. The phenotype of resistant interaction was characterized by the inability of the weeds haustoria to penetrate the sorghums root endodermis due to severe necrosis and in rare cases the parasites radicle growing away from the host root. The resistant sorghum varieties were the Asareca W2, Asareca AG3, N13 and the Wild-type which had low mean number of S. hermonthica plantlets growing on their roots, while the most susceptible varieties were Sap 027, Epurpur which had the highest mean number of S. hermonthica plantlets growing on their roots. There was a highly significant difference in the means of the number of Striga growing on the roots of sorghum varieties, Striga dry biomass and S. hermonthica length between the susceptible and resistant ones. This knowledge holds unique potentiality since resistant sorghum germplasm tested will be sourced and targeted to the seven specific geographical areas where virulence of the particular S. hermonthica populations was characterized. </td> <td style="text-align:left;"> NA </td> <td style="text-align:left;"> no </td> <td style="text-align:left;"> No good fitness measure and measure host GD </td> <td style="text-align:left;"> NA </td> <td style="text-align:left;"> no </td> <td style="text-align:left;"> no </td> </tr> <tr> <td style="text-align:left;"> 5172 </td> <td style="text-align:left;"> 2020 </td> <td style="text-align:left;"> 44112 </td> <td style="text-align:left;"> John, SE; Antony, D; Eaaswarkhanth, M; Hebbar, P; Channanath, AM; Thomas, D; Devarajan, S; Tuomilehto, J; Al-Mulla, F; Alsmadi, O; Thanaraj, TA </td> <td style="text-align:left;"> 2018 </td> <td style="text-align:left;"> Assessment of coding region variants in Kuwaiti population: implications for medical genetics and population genomics </td> <td style="text-align:left;"> Consanguineous populations of the Arabian Peninsula have been underrepresented in global efforts that catalogue human exome variability. We sequenced 291 whole exomes of unrelated, healthy native Arab individuals from Kuwait to a median coverage of 45X and characterised 170,508 single-nucleotide variants (SNVs), of which 21.7% were 'personal'. Up to 12% of the SNVs were novel and 36% were population-specific. Half of the SNVs were rare and 54% were missense variants. The study complemented the Greater Middle East Variome by way of reporting many additional Arabian exome variants. The study corroborated Kuwaiti population genetic substructures previously derived using genome-wide genotype data and illustrated the genetic relatedness among Kuwaiti population subgroups, Middle Eastern, European and Ashkenazi Jewish populations. The study mapped 112 rare and frequent functional variants relating to pharmacogenomics and disorders (recessive and common) to the phenotypic characteristics of Arab population. Comparative allele frequency data and carrier distributions of known Arab mutations for 23 disorders seen among Arabs, of putative OMIM-listed causal mutations for 12 disorders observed among Arabs but not yet characterized for genetic basis in Arabs, and of 17 additional putative mutations for disorders characterized for genetic basis in Arab populations are presented for testing in future Arab studies. </td> <td style="text-align:left;"> NA </td> <td style="text-align:left;"> no </td> <td style="text-align:left;"> human </td> <td style="text-align:left;"> NA </td> <td style="text-align:left;"> no </td> <td style="text-align:left;"> no </td> </tr> <tr> <td style="text-align:left;"> 5173 </td> <td style="text-align:left;"> 2020 </td> <td style="text-align:left;"> 44113 </td> <td style="text-align:left;"> Johnson, AE; Pruett-Jones, S </td> <td style="text-align:left;"> 2018 </td> <td style="text-align:left;"> Reproductive promiscuity in the variegated fairy-wren: an alternative reproductive strategy in the absence of helpers? </td> <td style="text-align:left;"> Cooperative breeding, in which auxiliary group members help rear related, but nondescendent young, is often explained by kin selection. Reproductive monogamy is predicted in cooperatively breeding systems, as monogamy increases intragroup relatedness and maximizes auxiliary inclusive fitness. While monogamy is observed across many systems, including eusocial insects and cooperatively breeding mammals, some cooperatively breeding birds exhibit high rates of extrapair paternity. Here we quantify paternity and examine the role of auxiliaries on extrapair paternity in the highly cooperative variegated fairy-wren, Malurus lamberti, a species with both male and female auxiliaries. Extrapair paternity occurred in 55.4% of nests, and 39.8% of offspring were the result of extrapair matings. The presence of both male and female auxiliaries had a positive relationship with the percentage of within-pair young sired by dominant males, however, the presence of male auxiliaries had a stronger impact than the presence of females. The number of extrapair young sired by dominant males decreased as the number of male auxiliaries increased. The total number of young sired by dominant males, however, was not predicted by group size or relatedness to their social partner, nor did group composition or relatedness to the breeding pair predict the reproductive success of subordinate males. We hypothesize that breeders use alternative reproductive strategies in the presence or absence of auxiliaries. Males and females may seek extrapair reproductive opportunities when no help is available in their group and nest survival is expected to be low. When help is available, breeders may reduce extrapair paternity, either to increase intragroup relatedness or because confidence in nest survival is high. Our data suggest that group composition is important in understanding extrapair paternity rates in cooperatively breeding birds and that variation in extrapair paternity rates may be the result of flexible breeding strategies when auxiliary presence and identity varies. (c) 2018 The Association for the Study of Animal Behaviour. Published by Elsevier Ltd. All rights reserved. </td> <td style="text-align:left;"> NA </td> <td style="text-align:left;"> no </td> <td style="text-align:left;"> No good fitness measure and measure host GD </td> <td style="text-align:left;"> NA </td> <td style="text-align:left;"> no </td> <td style="text-align:left;"> no </td> </tr> <tr> <td style="text-align:left;"> 5174 </td> <td style="text-align:left;"> 2020 </td> <td style="text-align:left;"> 44040 </td> <td style="text-align:left;"> Johnson, AJ; Orth, RJ; Moore, KA </td> <td style="text-align:left;"> 2020 </td> <td style="text-align:left;"> The role of sexual reproduction in the maintenance of established Zostera marina meadows </td> <td style="text-align:left;"> For clonal plants, the role of sexual reproduction in the maintenance of populations can vary widely. Some species are dependent on repeated seedling recruitment. For other species, interactions between adults and seedlings within existing populations can affect seedling survival and limit sexual reproduction in existing populations. Genetic studies of seagrass populations increasingly suggest sexual reproduction is important for the resilience and stability of their populations, but as of yet little observational data support these findings. Because seagrass populations provide important ecosystem services and are threatened with increasing anthropogenic impacts, understanding their reliance on sexual reproduction is evolutionarily and ecologically important. The goals of this study were to determine (a) whether seedlings of a marine angiosperm, Zostera marina, establish and recruit within existing Z. marina meadows and (b) whether interactions between seedlings and surrounding adult shoots influence the survival of established seedlings. To meet these goals, surveys estimated seedling establishment and tracked seedling survival within multiple populations. Manipulative experiments then tested the impact of neighbouring adult shoots on seedling survival and the overall trajectory of experimental plots with and without sexual reproduction. A 3-year survey identified established seedlings within Z. marina meadows each year. Additionally, concurrent seed addition experiments indicated seed supply could influence seedling establishment rates. A survey tracking the survival of tagged seedlings, as well as the height and density of surrounding adult shoots, showed adult shoots may negatively impact seedling survival. Experiments then demonstrated that seedlings without neighbouring shoots survived longer than those with neighbouring shoots. Lastly, two transplant garden experiments comparing the survival of plots with and without seeds highlighted that seedling recruitment is likely most important to maintain bottom cover where disturbances generate gaps in the adult population. Synthesis. This study demonstrates that seedlings do establish within existing seagrass meadows, and that some survive to recruit into the adult population. Competition with existing vegetation, however, can be a factor compromising seedling survival. Sexual reproduction may thus most likely occur in, and be most important for, clonal plant populations that experience seasonal disturbance. </td> <td style="text-align:left;"> NA </td> <td style="text-align:left;"> no </td> <td style="text-align:left;"> No good measureGD of host </td> <td style="text-align:left;"> NA </td> <td style="text-align:left;"> no </td> <td style="text-align:left;"> no </td> </tr> <tr> <td style="text-align:left;"> 5175 </td> <td style="text-align:left;"> 2020 </td> <td style="text-align:left;"> 44040 </td> <td style="text-align:left;"> Jones, JE; Hurst, GDD </td> <td style="text-align:left;"> 2020 </td> <td style="text-align:left;"> Symbiont-mediated protection varies with wasp genotype in the Drosophila melanogaster-Spiroplasma interaction </td> <td style="text-align:left;"> The ability of an insect to survive attack by natural enemies can be modulated by the presence of defensive symbionts. Study of aphid-symbiont-enemy interactions has indicated that protection may depend on the interplay of symbiont, host and attacking parasite genotypes. However, the importance of these interactions is poorly understood outside of this model system. Here, we study interactions within a Drosophila model system, in which Spiroplasma protect their host against parasitoid wasps and nematodes. We examine whether the strength of protection conferred by Spiroplasma to its host, Drosophila melanogaster varies with strain of attacking Leptopilina heterotoma wasp. We perform this analysis in the presence and absence of ethanol, an environmental factor that also impacts the outcome of parasitism. We observed that Spiroplasma killed all strains of wasp. However, the protection produced by Spiroplasma following wasp attack depended on wasp strain. A composite measure of protection, including both the chance of the fly surviving attack and the relative fecundity/fertility of the survivors, varied from a Spiroplasma-Drosophila-wasp tripartite interaction depend upon the genetic diversity within the attacking wasp population, and that prediction of symbiont dynamics in natural systems will thus require analysis across natural enemy genotypes and levels of environmental ethanol. </td> <td style="text-align:left;"> NA </td> <td style="text-align:left;"> no </td> <td style="text-align:left;"> n </td> <td style="text-align:left;"> NA </td> <td style="text-align:left;"> no </td> <td style="text-align:left;"> no </td> </tr> <tr> <td style="text-align:left;"> 5176 </td> <td style="text-align:left;"> 2020 </td> <td style="text-align:left;"> 44040 </td> <td style="text-align:left;"> Jones, K; Burgess, G; Budd, AM; Huerlimann, R; Mashkour, N; Ariel, E </td> <td style="text-align:left;"> 2020 </td> <td style="text-align:left;"> Molecular evidence for horizontal transmission of chelonid alphaherpesvirus 5 at green turtle (Chelonia mydas) foraging grounds in Queensland, Australia </td> <td style="text-align:left;"> Fibropapillomatosis (FP) is a marine turtle disease recognised by benign tumours on the skin, eyes, shell, oral cavity and/or viscera. Despite being a globally distributed disease that affects an endangered species, research on FP and its likely causative agent chelonid alphaherpesvirus 5 (ChHV5) in Australia is limited. Here we present improved molecular assays developed for detection of ChHV5, in combination with a robust molecular and phylogenetic analysis of ChHV5 variants. This approach utilised a multi-gene assay to detect ChHV5 in all FP tumors sampled from 62 marine turtles found at six foraging grounds along the Great Barrier Reef. Six distinct variants of ChHV5 were identified and the distribution of these variants was associated with host foraging ground. Conversely, no association between host genetic origin and ChHV5 viral variant was found. Together this evidence supports the hypothesis that marine turtles undergo horizontal transmission of ChHV5 at foraging grounds and are unlikely to be contracting the disease at rookeries, either during mating or vertically from parent to offspring. </td> <td style="text-align:left;"> NA </td> <td style="text-align:left;"> no </td> <td style="text-align:left;"> No good fitness measure and measure host GD </td> <td style="text-align:left;"> NA </td> <td style="text-align:left;"> no </td> <td style="text-align:left;"> no </td> </tr> <tr> <td style="text-align:left;"> 5177 </td> <td style="text-align:left;"> 2020 </td> <td style="text-align:left;"> 44113 </td> <td style="text-align:left;"> Jorge, PH; Mastrochirico-Filho, VA; Hata, ME; Mendes, NJ; Ariede, RB; de Freitas, MV; Vera, M; Porto-Foresti, F; Hashimoto, DT </td> <td style="text-align:left;"> 2018 </td> <td style="text-align:left;"> Genetic Characterization of the Fish Piaractus brachypomus by Microsatellites Derived from Transcriptome Sequencing </td> <td style="text-align:left;"> The pirapitinga, Piaractus brachypomus (Characiformes, Serrasalmidae), is a fish from the Amazon basin and is considered to be one of the main native species used in aquaculture production in South America. The objectives of this study were: (1) to perform liver transcriptome sequencing of pirapitinga through NGS and then validate a set of microsatellite markers for this species; and (2) to use polymorphic microsatellites for analysis of genetic variability in farmed stocks. The transcriptome sequencing was carried out through the Roche/454 technology, which resulted in 3,696 non-redundant contigs. Of this total, 2,568 contigs had similarity in the non-redundant (nr) protein database (Genbank) and 2,075 sequences were characterized in the categories of Gene Ontology (GO). After the validation process of 30 microsatellite loci, eight markers showed polymorphism. The analysis of these polymorphic markers in farmed stocks revealed that fish farms from North Brazil had a higher genetic diversity than fish farms from Southeast Brazil. AMOVA demonstrated that the highest proportion of variation was presented within the populations. However, when comparing different groups (1: Wild; 2: North fish farms; 3: Southeast fish farms), a considerable variation between the groups was observed. The F-ST values showed the occurrence of genetic structure among the broodstocks from different regions of Brazil. The transcriptome sequencing in pirapitinga provided important genetic resources for biological studies in this non-model species, and microsatellite data can be used as the framework for the genetic management of breeding stocks in Brazil, which might provide a basis for a genetic pre-breeding programme. </td> <td style="text-align:left;"> NA </td> <td style="text-align:left;"> no </td> <td style="text-align:left;"> No good fitness measure and measure host GD </td> <td style="text-align:left;"> NA </td> <td style="text-align:left;"> no </td> <td style="text-align:left;"> no </td> </tr> <tr> <td style="text-align:left;"> 5178 </td> <td style="text-align:left;"> 2020 </td> <td style="text-align:left;"> 44113 </td> <td style="text-align:left;"> Jossart, Q; De Ridder, C; Lessios, HA; Bauwens, M; Motreuil, S; Rigaud, T; Wattier, RA; David, B </td> <td style="text-align:left;"> 2017 </td> <td style="text-align:left;"> Highly contrasted population genetic structures in a host-parasite pair in the Caribbean Sea </td> <td style="text-align:left;"> Evolution and population genetic structure of marine species across the Caribbean Sea are shaped by two complex factors: the geological history and the present pattern of marine currents. Characterizing and comparing the genetic structures of codistributed species, such as host-parasite associations, allow discriminating the relative importance of environmental factors and life history traits that influenced gene flow and demographic events. Using microsatellite and Cytochrome Oxidase I markers, we investigated if a host-parasite pair (the heart urchin Meoma ventricosa and its parasitic pea crab Dissodactylus primitivus) exhibits comparable population genetic structures in the Caribbean Sea and how the observed patterns match connectivity regions from predictive models and other taxa. Highly contrasting patterns were found: the host showed genetic homogeneity across the whole studied area, whereas the parasite displayed significant differentiation at regional and local scales. The genetic diversity of the parasitic crabs (both in microsatellites and COI) was distributed in two main groups, Panama-Jamaica-St Croix on the one hand, and the South-Eastern Caribbean on the other. At a smaller geographical scale, Panamanian and Jamaican parasite populations were genetically more similar, while more genetic differentiation was found within the Lesser Antilles. Both species showed a signature of population expansion during the Quaternary. Some results match predictive models or data from previous studies (e.g., the Western-Eastern dichotomy in the parasite) while others do not (e.g., genetic differentiation within the Lesser Antilles). The sharp dissimilarity of genetic structure of these codistributed species outlines the importance of population expansion events and/or contrasted patterns of gene flow. This might be linked to differences in several life history traits such as fecundity (higher for the host), swimming capacity of larval stages (higher for the parasite), and habitat availability (higher for the host). </td> <td style="text-align:left;"> NA </td> <td style="text-align:left;"> no </td> <td style="text-align:left;"> No good fitness measure and measure host GD </td> <td style="text-align:left;"> NA </td> <td style="text-align:left;"> no </td> <td style="text-align:left;"> no </td> </tr> <tr> <td style="text-align:left;"> 5179 </td> <td style="text-align:left;"> 2020 </td> <td style="text-align:left;"> 44111 </td> <td style="text-align:left;"> Juanillas, V; Dereeper, A; Beaume, N; Droc, G; Dizon, J; Mendoza, JR; Perdon, JP; Mansueto, L; Triplett, L; Lang, J; Zhou, G; Ratharanjan, K; Plale, B; Haga, J; Leach, JE; Ruiz, M; Thomson, M; Alexandrov, N; Larmande, P; Kretzschmar, T; Mauleon, RP </td> <td style="text-align:left;"> 2019 </td> <td style="text-align:left;"> Rice Galaxy: an open resource for plant science </td> <td style="text-align:left;"> Background: Rice molecular genetics, breeding, genetic diversity, and allied research (such as rice-pathogen interaction) have adopted sequencing technologies and high-density genotyping platforms for genome variation analysis and gene discovery. Germplasm collections representing rice diversity, improved varieties, and elite breeding materials are accessible through rice gene banks for use in research and breeding, with many having genome sequences and high-density genotype data available. Combining phenotypic and genotypic information on these accessions enables genome-wide association analysis, which is driving quantitative trait loci discovery and molecular marker development. Comparative sequence analyses across quantitative trait loci regions facilitate the discovery of novel alleles. Analyses involving DNA sequences and large genotyping matrices for thousands of samples, however, pose a challenge to non-computer savvy rice researchers. Findings: The Rice Galaxy resource has shared datasets that include high-density genotypes from the 3,000 Rice Genomes project and sequences with corresponding annotations from 9 published rice genomes. The Rice Galaxy web server and deployment installer includes tools for designing single-nucleotide polymorphism assays, analyzing genome-wide association studies, population diversity, rice-bacterial pathogen diagnostics, and a suite of published genomic prediction methods. A prototype Rice Galaxy compliant to Open Access, Open Data, and Findable, Accessible, Interoperable, and Reproducible principles is also presented. Conclusions: Rice Galaxy is a freely available resource that empowers the plant research community to perform state-of-the-art analyses and utilize publicly available big datasets for both fundamental and applied science. </td> <td style="text-align:left;"> NA </td> <td style="text-align:left;"> no </td> <td style="text-align:left;"> No good fitness measure and measure host GD </td> <td style="text-align:left;"> NA </td> <td style="text-align:left;"> no </td> <td style="text-align:left;"> no </td> </tr> <tr> <td style="text-align:left;"> 5180 </td> <td style="text-align:left;"> 2020 </td> <td style="text-align:left;"> 44113 </td> <td style="text-align:left;"> Jueterbock, A; Coyer, JA; Olsen, JL; Hoarau, G </td> <td style="text-align:left;"> 2018 </td> <td style="text-align:left;"> Decadal stability in genetic variation and structure in the intertidal seaweed Fucus serratus (Heterokontophyta: Fucaceae) </td> <td style="text-align:left;"> Background: The spatial distribution of genetic diversity and structure has important implications for conservation as it reveals a species' strong and weak points with regard to stability and evolutionary capacity. Temporal genetic stability is rarely tested in marine species other than commercially important fishes, but is crucial for the utility of temporal snapshots in conservation management. High and stable diversity can help to mitigate the predicted northward range shift of seaweeds under the impact of climate change. Given the key ecological role of fucoid seaweeds along rocky shores, the positive effect of genetic diversity may reach beyond the species level to stabilize the entire intertidal ecosystem along the temperate North Atlantic. In this study, we estimated the effective population size, as well as temporal changes in genetic structure and diversity of the seaweed F. serratus using 22 microsatellite markers. Samples were taken across latitudes and a range of temperature regimes at seven locations with decadal sampling (2000 and 2010). Results: Across latitudes, genetic structure and diversity remained stable over 5-10 generations. Stable small-scale structure enhanced regional diversity throughout the species' range. In accordance with its biogeographic history, effective population size and diversity peaked in the species' mid-range in Brittany (France), and declined towards its leading and trailing edge to the north and south. At the species' southern edge, multi-locus-heterozygosity displayed a strong decline from 1999 to 2010. Conclusion: Temporally stable genetic structure over small spatial scales is a potential driver for local adaptation and species radiation in the genus Fucus. Survival and adaptation of the low-diversity leading edge of F. serratus may be enhanced by regional gene flow and 'surfing' of favorable mutations or impaired by the accumulation of deleterious mutations. Our results have clear implications for the conservation of F. serratus at its genetically unique southern edge in Northwest Iberia, where increasing temperatures are likely the major cause for the decline not only of F. serratus, but also other intertidal and subtidal macroalgae. We expect that F. serratus will disappear from Northwest Iberia by 2100 if genetic rescue is not induced by the influx of genetic variation from Brittany. </td> <td style="text-align:left;"> NA </td> <td style="text-align:left;"> no </td> <td style="text-align:left;"> no good gd measure host pop </td> <td style="text-align:left;"> NA </td> <td style="text-align:left;"> no </td> <td style="text-align:left;"> no </td> </tr> <tr> <td style="text-align:left;"> 5181 </td> <td style="text-align:left;"> 2020 </td> <td style="text-align:left;"> 44111 </td> <td style="text-align:left;"> Jungwirth, A; Balzarini, V; Zottl, M; Salzmann, A; Taborsky, M; Frommen, JG </td> <td style="text-align:left;"> 2019 </td> <td style="text-align:left;"> Long-term individual marking of small freshwater fish: the utility of Visual Implant Elastomer tags </td> <td style="text-align:left;"> Tracking wild animals over long periods of time is a non-trivial challenge. This has caused a bias in the availability of individual-based long-term datasets with the majority including birds and mammals. Visual Implant Elastomer (VIE) tags are now a widely used technique that may facilitate the collection of such data for fish and amphibians. However, VIE tags might have important drawbacks. Overall, four potential issues with VIE tags have been proposed: tag loss or misidentification, limited number of individual identifiers, enhanced mortality risk, and effects on intra-specific interactions. Here, we present three experiments in which we investigated these potential problems with VIE tagging in small freshwater fish both in the laboratory and in the wild, using the cooperatively breeding Lake Tanganyika cichlid Neolamprologus pulcher. We find VIE tags to be generally suitable for work with these fish as they did not impair survival, were recognisable up to 2years after injection, and did not generally disturb group formation. Nevertheless, we identify specific issues of VIE tagging, including colour- and position-dependent variation in tag identification rates, and indications that specific colours may influence social behaviour. Our results demonstrate the suitability of VIE tags for long-term studies on small freshwater fish, while also highlighting the need of validating this method carefully for any species and study.Significance statementInformation on the survival, dispersal, and reproductive success of wild individuals across their lifespan is among the most valuable data in Behavioural Ecology. Because tracking of free-ranging individuals over extended periods of time is challenging, there exists a bias in the taxonomic distribution of such long-term datasets. Here, we investigate the suitability of visible implant elastomers (VIE) as a tracking technique to allow for the collection of such data also in small tropical freshwater fish. We show that VIE tags neither alter social behaviour in our study species, nor do they reduce survival, but they enable the tracking of wild individuals across years. We also identify colours and tag positions that are less suitable. We conclude that VIE tags can help produce long-term datasets also for small fish, provided certain precautions are met. </td> <td style="text-align:left;"> NA </td> <td style="text-align:left;"> no </td> <td style="text-align:left;"> No good fitness measure and measure host GD </td> <td style="text-align:left;"> NA </td> <td style="text-align:left;"> no </td> <td style="text-align:left;"> no </td> </tr> <tr> <td style="text-align:left;"> 5182 </td> <td style="text-align:left;"> 2020 </td> <td style="text-align:left;"> 44113 </td> <td style="text-align:left;"> Kaiser, SA; Martin, TE; Oteyza, JC; Armstad, C; Fleischer, RC </td> <td style="text-align:left;"> 2018 </td> <td style="text-align:left;"> Direct fitness benefits and kinship of social foraging groups in an Old World tropical babbler </td> <td style="text-align:left;"> Molecular studies have revealed that social groups composed mainly of nonrelatives may be widespread in group-living vertebrates, but the benefits favoring such sociality are not well understood. In the Old World, birds often form conspecific foraging groups that are maintained year-round and offspring usually disperse to other social groups. We tested the hypothesis that nonbreeding group members are largely unrelated and gain direct fitness benefits through breeding opportunities (males) and brood parasitism (females) in the tropical gray-throated babbler, Stachyris nigriceps, in Malaysian Borneo. Babblers foraged in social groups containing one or more breeding pairs (median = 8 group members of equal sex ratio), but group members rarely assisted with breeding (9% of 67 breeding pairs had a third helper; exhibiting facultative cooperative breeding). Although 20% of 266 group member dyads were first-order relatives of one or both members of the breeding pairs, 80% were unrelated. Male group members gained direct fitness benefits through extrapair and extra-group paternity (25% of 73 offspring), which was independent of their relatedness to the breeding pair and increased with decreasing group size. In contrast, females did not gain direct fitness benefits through brood parasitism. The low levels of relatedness and helping in social groups suggest that most group members do not gain indirect fitness benefits by helping to raise unrelated offspring. These findings highlight the importance of examining benefits of sociality for unrelated individuals that largely do not help and broaden the direct fitness benefits of group foraging beyond assumed survival benefits. </td> <td style="text-align:left;"> NA </td> <td style="text-align:left;"> no </td> <td style="text-align:left;"> No good fitness measure and measure host GD </td> <td style="text-align:left;"> NA </td> <td style="text-align:left;"> no </td> <td style="text-align:left;"> no </td> </tr> <tr> <td style="text-align:left;"> 5183 </td> <td style="text-align:left;"> 2020 </td> <td style="text-align:left;"> 44041 </td> <td style="text-align:left;"> Kaiser, SA; Martin, TE; Oteyza, JC; Danner, JE; Armstad, CE; Fleischer, RC </td> <td style="text-align:left;"> 2019 </td> <td style="text-align:left;"> Within-group relatedness and patterns of reproductive sharing and cooperation in the tropical chestnut-crested yuhina </td> <td style="text-align:left;"> In cooperatively breeding animals, genetic relatedness among group members often determines the extent of reproductive sharing, cooperation and competition within a group. Studies of species for which cooperative behaviour is not entirely based on kinship are key for understanding the benefits favouring the evolution and maintenance of cooperative breeding among nonrelatives. In the cooperatively breeding chestnut-crested yuhina, Yuhina everetti, a songbird endemic to Borneo, we tested whether unrelated helpers are more likely to gain parentage than are related helpers consistent with the hypothesis that inbreeding risk constrains reproduction by related helpers. We also examined whether related or unrelated helpers provision broods more because of differences in their potential indirect or direct fitness benefits of helping. Kin structure of breeding groups (breeding pair and up to eight helpers of both sexes, median = 2 helpers, 96% of 57 pairs had helpers) based on genetic analysis was mixed; 48% of 76 breeder/helper dyads were first-order (26%) or second-order (22%) relatives of one or both members of the breeding pair, and 52% were nonrelatives. Only unrelated male and female helpers gained parentage, and helpers did not differ in their provisioning rate according to their relatedness to the broods. We documented quasi-parasitism or co-breeding by female helpers in 14% of 29 broods and extrapair paternity by male helpers in 21% of 47 broods. This rate of extrapair paternity is relatively high among the few tropical species examined but fit with predictions for mixed-kin groups where inbreeding is avoided. These findings support the emerging pattern for cooperative breeding in birds with mixed-kin groups, wherein unrelated helpers are more likely to gain parentage than are related helpers and helping effort is not necessarily predicted by kinship. (C) 2019 The Association for the Study of Animal Behaviour. Published by Elsevier Ltd. All rights reserved. </td> <td style="text-align:left;"> NA </td> <td style="text-align:left;"> no </td> <td style="text-align:left;"> n </td> <td style="text-align:left;"> NA </td> <td style="text-align:left;"> no </td> <td style="text-align:left;"> no </td> </tr> <tr> <td style="text-align:left;"> 5184 </td> <td style="text-align:left;"> 2020 </td> <td style="text-align:left;"> 44112 </td> <td style="text-align:left;"> Kalyebi, A; Macfadyen, S; Parry, H; Tay, WT; De Barro, P; Colvin, J </td> <td style="text-align:left;"> 2018 </td> <td style="text-align:left;"> African cassava whitefly, Bemisia tabaci, cassava colonization preferences and control implications </td> <td style="text-align:left;"> Cassava is a staple food for people across sub-Saharan Africa. Over the last 20 years, there has been an increased frequency of outbreaks and crop damage in this region caused by the cassava-adapted Bemisia tabaci putative species. Little is known about when and why B. tabaci adults move and colonize new cassava crops, especially in farming systems that contain a mixture of cultivar types and plant ages. Here, we assessed experimentally whether the age and variety of cassava affected the density of B. tabaci. We also tested whether the age and variety of the source cassava field affected the variety preference of B. tabaci when they colonized new cassava plants. We placed uninfested potted sentinel plants of three cassava varieties (Nam 130, Nase 14, and Njule Red) in source fields containing one of two varieties (Nam 130 or Nase 14) and one of three age classes (young, medium, or old). After two weeks, the numbers of nymphs on the sentinel plants were used as a measure of colonization. Molecular identification revealed that the B. tabaci species was sub-Saharan Africa 1 (SSA1). We found a positive correlation between the density of nymphs on sentinel plants and the density of adults in the source field. The density of nymphs on the sentinels was not significantly related to the age of the source field. Bemisia tabaci adults did not preferentially colonize the sentinel plant of the same variety as the source field. There was a significant interactive effect, however, between the source and sentinel variety that may indicate variability in colonization. We conclude that managing cassava source fields to reduce B. tabaci abundance will be more effective than manipulating nearby varieties. We also suggest that planting a whitefly sink variety is unlikely to reduce B. tabaci SSA1 populations unless fields are managed to reduce B. tabaci densities using other integrative approaches. </td> <td style="text-align:left;"> NA </td> <td style="text-align:left;"> no </td> <td style="text-align:left;"> No good fitness measure and measure host GD </td> <td style="text-align:left;"> NA </td> <td style="text-align:left;"> no </td> <td style="text-align:left;"> no </td> </tr> <tr> <td style="text-align:left;"> 5185 </td> <td style="text-align:left;"> 2020 </td> <td style="text-align:left;"> 44042 </td> <td style="text-align:left;"> Kamath, PL; Manlove, K; Cassirer, EF; Cross, PC; Besser, TE </td> <td style="text-align:left;"> 2019 </td> <td style="text-align:left;"> Genetic structure of Mycoplasma ovipneumoniae informs pathogen spillover dynamics between domestic and wild Caprinae in the western United States </td> <td style="text-align:left;"> Spillover diseases have significant consequences for human and animal health, as well as wildlife conservation. We examined spillover and transmission of the pneumonia-associated bacterium Mycoplasma ovipneumoniae in domestic sheep, domestic goats, bighorn sheep, and mountain goats across the western United States using 594 isolates, collected from 1984 to 2017. Our results indicate high genetic diversity of M. ovipneumoniae strains within domestic sheep, whereas only one or a few strains tend to circulate in most populations of bighorn sheep or mountain goats. These data suggest domestic sheep are a reservoir, while the few spillovers to bighorn sheep and mountain goats can persist for extended periods. Domestic goat strains form a distinct clade from those in domestic sheep, and strains from both clades are found in bighorn sheep. The genetic structure of domestic sheep strains could not be explained by geography, whereas some strains are spatially clustered and shared among proximate bighorn sheep populations, supporting pathogen establishment and spread following spillover. These data suggest that the ability to predict M. ovipneumoniae spillover into wildlife populations may remain a challenge given the high strain diversity in domestic sheep and need for more comprehensive pathogen surveillance. </td> <td style="text-align:left;"> NA </td> <td style="text-align:left;"> no </td> <td style="text-align:left;"> dometicated host </td> <td style="text-align:left;"> NA </td> <td style="text-align:left;"> no </td> <td style="text-align:left;"> no </td> </tr> <tr> <td style="text-align:left;"> 5186 </td> <td style="text-align:left;"> 2020 </td> <td style="text-align:left;"> 44113 </td> <td style="text-align:left;"> Kamvar, ZN; Amaradasa, BS; Jhala, R; McCoy, S; Steadman, JR; Everhart, SE </td> <td style="text-align:left;"> 2017 </td> <td style="text-align:left;"> Population structure and phenotypic variation of Sclerotinia sclerotiorum from dry bean (Phaseolus vulgaris) in the United States </td> <td style="text-align:left;"> The ascomycete pathogen Sclerotinia sclerotiorum is a necrotrophic pathogen on over 400 known host plants, and is the causal agent of white mold on dry bean. Currently, there are no known cultivars of dry bean with complete resistance to white mold. For more than 20 years, bean breeders have been using white mold screening nurseries (wmn) with natural populations of S. sclerotiorum to screen new cultivars for resistance. It is thus important to know if the genetic diversity in populations of S. sclerotiorum within these nurseries (a) reflect the genetic diversity of the populations in the surrounding region and (b) are stable over time. Furthermore, previous studies have investigated the correlation between mycelial compatibility groups (MCG) and multilocus haplotypes (MLH), but none have formally tested these patterns. We genotyped 366 isolates of S. sclerotiorum from producer fields and wmn surveyed over 10 years in 2003-2012 representing 11 states in the United States of America, Australia, France, and Mexico at 11 microsatellite loci resulting in 165 MLHs. Populations were loosely structured over space and time based on analysis of molecular variance and discriminant analysis of principal components, but not by cultivar, aggressiveness, or field source. Of all the regions tested, only Mexico (n = 18) shared no MLHs with any other region. Using a bipartite networkbased approach, we found no evidence that the MCGs accurately represent MLHs. Our study suggests that breeders should continue to test dry bean lines in several wmn across the United States to account for both the phenotypic and genotypic variation that exists across regions. </td> <td style="text-align:left;"> NA </td> <td style="text-align:left;"> no </td> <td style="text-align:left;"> gd of pathogen </td> <td style="text-align:left;"> NA </td> <td style="text-align:left;"> no </td> <td style="text-align:left;"> no </td> </tr> <tr> <td style="text-align:left;"> 5187 </td> <td style="text-align:left;"> 2020 </td> <td style="text-align:left;"> 44111 </td> <td style="text-align:left;"> Kanagasundaram, NS; Baudouin, SV; Rowling, S; Prabhu, M; Dark, JH; Goodship, THJ; Chinnery, PF; Hudson, G </td> <td style="text-align:left;"> 2019 </td> <td style="text-align:left;"> Mitochondrial Haplogroup and the Risk of Acute Kidney Injury Following Cardiac Bypass Surgery </td> <td style="text-align:left;"> Although mitochondrial dysfunction plays a key role in the pathophysiology of acute kidney injury (AKI), the influence of mitochondrial genetic variability in this process remains unclear. We explored the association between the risk of post-cardiac bypass AKI and mitochondrial haplotype - inherited mitochondrial genomic variations of potentially functional significance. Our single-centre study recruited consecutive patients prior to surgery. Exclusions included stage 5 CKD, non-Caucasian race and subsequent off-pump surgery. Haplogroup analysis allowed characterisation of the study population using the common mutations and by phylogenetic supergroup (WXI and HV). Chi-square tests for association allowed the identification of potential predictors of AKI for use in logistic regression analysis. AKI occurred in 12.8% of the study population (n = 881; male 69.6%, non-diabetic 78.5%, median (interquartile range) age 68.0 (61.0-75.0) years). The haplogroup profile comprised H (42.7%), J (12.1%), T (10.9%), U (14.4%) and K (7.6%). Although the regression model was statistically significant (chi(2) = 95.483, p &lt; 0.0005), neither the phylogenetic supergroups nor any individual haplogroup was a significant contributor. We found no significant association between common European haplogroups and the risk of post-cardiac bypass AKI. However, given the major role of mitochondrial dysfunction in AKI, there is a need to replicate our findings in other cohorts and with other aetiologies of AKI. </td> <td style="text-align:left;"> NA </td> <td style="text-align:left;"> no </td> <td style="text-align:left;"> No good fitness measure and measure host GD </td> <td style="text-align:left;"> NA </td> <td style="text-align:left;"> no </td> <td style="text-align:left;"> no </td> </tr> <tr> <td style="text-align:left;"> 5188 </td> <td style="text-align:left;"> 2020 </td> <td style="text-align:left;"> 44112 </td> <td style="text-align:left;"> Kano, FS; de Souza, AM; Torres, LD; Costa, MA; Souza-Silva, FA; Sanchez, BAM; Fontes, CJF; Soares, IS; de Brito, CFA; Carvalho, LH; Sousa, TN </td> <td style="text-align:left;"> 2018 </td> <td style="text-align:left;"> Susceptibility to Plasmodium vivax malaria associated with DARC (Duffy antigen) polymorphisms is influenced by the time of exposure to malaria </td> <td style="text-align:left;"> Malaria has provided a major selective pressure and has modulated the genetic diversity of the human genome. The variants of the Duffy Antigen/Receptor for Chemokines (DARC) gene have probably been selected by malaria parasites, particularly the FY*O allele, which is fixed in sub-Saharan Africa and confers resistance to Plasmodium vivax infection. Here, we showed the influence of genomic ancestry on the distribution of DARC genotypes in a highly admixed Brazilian population and confirmed the decreased susceptibility of the FY*A/FY*O genotype to clinical P. vivax malaria. FY*B/FY*O individuals were associated with a greater risk of developing clinical malaria. A remarkable difference among DARC variants concerning the susceptibility to clinical malaria was more evident for individuals who were less exposed to malaria, as measured by the time of residence in the endemic area. Additionally, we found that DARC-negative and FY*A/FY*O individuals had a greater chance of acquiring high levels of antibodies against the 19-kDa C-terminal region of the P. vivax merozoite surface protein-1. Altogether, our results provide evidence that DARC polymorphisms modulate the susceptibility to clinical P. vivax malaria and influence the naturally-acquired humoral immune response to malaria blood antigens, which may interfere with the efficacy of a future vaccine against malaria. </td> <td style="text-align:left;"> NA </td> <td style="text-align:left;"> no </td> <td style="text-align:left;"> No good fitness measure and measure host GD </td> <td style="text-align:left;"> NA </td> <td style="text-align:left;"> no </td> <td style="text-align:left;"> no </td> </tr> <tr> <td style="text-align:left;"> 5189 </td> <td style="text-align:left;"> 2020 </td> <td style="text-align:left;"> 44113 </td> <td style="text-align:left;"> Kanthaswamy, S; Ng, J; Oldt, RF; Phillippi-Falkenstein, K; Kubisch, HM </td> <td style="text-align:left;"> 2018 </td> <td style="text-align:left;"> SNP-based genetic characterization of the Tulane National Primate Research Center's conventional and specific pathogen-free rhesus macaque (Macaca mulatta) populations </td> <td style="text-align:left;"> Background: The rhesus macaque is an important biomedical model organism, and the Tulane National Primate Research Center (TNPRC) has one of the largest rhesus macaque breeding colonies in the United States. Methods: SNP profiles from 3266 rhesus macaques were used to examine the TNPRC colony genetic composition over time and across conventional or SPF animals of Chinese and Indian ancestry. Results: Chinese origin animals were the least genetically diverse and the most inbred; however, since their derivation from their conventional forebearers, neither the Chinese nor the Indian SPF animals exhibit any significant loss of genetic diversity or differentiation. Conclusions: The TNPRC colony managers have successfully minimized loss in genetic variation across generations. Although founder effects and bottlenecks among the Indian animals have been successfully curtailed, the Chinese subpopulation still show some influences from these events. </td> <td style="text-align:left;"> NA </td> <td style="text-align:left;"> no </td> <td style="text-align:left;"> No good fitness measure </td> <td style="text-align:left;"> NA </td> <td style="text-align:left;"> no </td> <td style="text-align:left;"> no </td> </tr> <tr> <td style="text-align:left;"> 5190 </td> <td style="text-align:left;"> 2020 </td> <td style="text-align:left;"> 44040 </td> <td style="text-align:left;"> Kareinen, I; Lavonen, E; Viranta-Kovanen, S; Holmala, K; Laakkonen, J </td> <td style="text-align:left;"> 2020 </td> <td style="text-align:left;"> Anatomical variations and pathological changes in the hearts of free-ranging Eurasian lynx (Lynx lynx) in Finland </td> <td style="text-align:left;"> The Eurasian lynx (Lynx lynx) despite the wide distribution has fragmented populations with possibly decreased genetic variability. Reports from Central Europe have raised cardiac health as possible risk factor for lynx populations. The knowledge on normal anatomic variations of lynx heart is crucial to assess emerging pathological or hereditary disorders. The aim of this study was to give a detailed description of the cardiac anatomy and circulation of the Eurasian lynx and to report the cardiac health of the lynx in Finland. The cardiac anatomy and pathology were studied post mortem from 63 legally hunted lynx. In general, the cardiac anatomy of Eurasian lynx corresponded with that described for other felids. In the ventricles, varying number of thin ventricular bands was a common feature and their histological appearance was characterized by a fibromuscular pattern. The size of the heart varied between males and females, but the relative size was similar to that described for most domesticated carnivores. No marked pathologic lesions were observed in the lynx hearts. Fibrosis was observed in 56% of the hearts, but it was focal and mild in degree and unlikely to affect cardiac function or to have clinical significance. In conclusion, the cardiac health of the Finnish Eurasian lynx population is good with no signs of heritable cardiac disorders. Furthermore, we were able to give a detailed anatomic description of the lynx heart, which can serve as a reference for further epidemiological investigations on cardiac diseases in lynx populations. </td> <td style="text-align:left;"> NA </td> <td style="text-align:left;"> no </td> <td style="text-align:left;"> No group level </td> <td style="text-align:left;"> NA </td> <td style="text-align:left;"> no </td> <td style="text-align:left;"> no </td> </tr> <tr> <td style="text-align:left;"> 5191 </td> <td style="text-align:left;"> 2020 </td> <td style="text-align:left;"> 44112 </td> <td style="text-align:left;"> Kaspar, RE; Cook, CN; Breed, MD </td> <td style="text-align:left;"> 2018 </td> <td style="text-align:left;"> Experienced individuals influence the thermoregulatory fanning behaviour in honey bee colonies </td> <td style="text-align:left;"> The survival of an animal society depends on how individual interactions influence group coordination. Interactions within a group determine coordinated responses to environmental changes. Individuals that are especially influential affect the behavioural responses of other group members. This is exemplified by honey bee worker responses to increasing ambient temperatures by fanning their wings to circulate air through the hive. Groups of workers are more likely to fan than isolated workers, suggesting a coordinated group response. But are some individuals more influential than others in this response? This study tests the hypothesis that an individual influences other group members to perform thermoregulatory fanning behaviour in the western honey bee, Apis mellifera L. We show that groups of young nurse bees placed with fanners are more likely to initiate fanning compared to groups of nurses without fanners. Furthermore, we find that groups with young nurse bees have lower response thresholds than groups of just fanners. Our results suggest that individuals have the capability to influence other individuals to follow their fanning response as temperatures increase, and these social dynamics balance probability of fanning with thermal response thresholds. An influential individual may ultimately affect the ability for a society to efficiently respond to environmental fluctuations. (C) 2018 The Association for the Study of Animal Behaviour. Published by Elsevier Ltd. All rights reserved. </td> <td style="text-align:left;"> NA </td> <td style="text-align:left;"> no </td> <td style="text-align:left;"> no good gd measure host pop </td> <td style="text-align:left;"> NA </td> <td style="text-align:left;"> no </td> <td style="text-align:left;"> no </td> </tr> <tr> <td style="text-align:left;"> 5192 </td> <td style="text-align:left;"> 2020 </td> <td style="text-align:left;"> 44111 </td> <td style="text-align:left;"> Kaszta, Z; Cushman, SA; Hearn, AJ; Burnham, D; Macdonald, EA; Goossens, B; Nathan, SKSS; Macdonald, DW </td> <td style="text-align:left;"> 2019 </td> <td style="text-align:left;"> Integrating Sunda clouded leopard (Neofelis diardi) conservation into development and restoration planning in Sabah (Borneo) </td> <td style="text-align:left;"> Changes in land use/cover are the main drivers of global biodiversity loss, and thus tools to evaluate effects of landscape change on biodiversity are crucial. In this study we integrated several methods from landscape ecology and landscape genetics into a GIS-based analytical framework, and evaluated the impacts of development and forest restoration scenarios on landscape connectivity, population dynamics and genetic diversity of Sunda clouded leopard in the Malaysian state of Sabah. We also investigated the separate and interactive effects of changing mortality risk and connectivity. Our study suggested that the current clouded leopard population size is larger (+26%) than the current carrying capacity of the landscape due to time lag effects and extinction debt. Additionally, we predicted that proposed developments in Sabah may decrease landscape connectivity by 23% and, when including the increased mortality risk associated with these developments, result in a 40-63% decrease in population size and substantial reduction in genetic diversity. These negative impacts could be mitigated only to a very limited degree through extensive and targeted forest restoration. Our results suggest that realignment of roads and railways based on resistance to movement, without including mortality risk, might be misleading and may in some cases lead to decrease in population size. We therefore recommend that efforts to optimally plan road and railway locations base the optimization on effects of development on population size, density and distribution rather than solely on population connectivity. </td> <td style="text-align:left;"> NA </td> <td style="text-align:left;"> no </td> <td style="text-align:left;"> No good fitness measure and measure host GD </td> <td style="text-align:left;"> NA </td> <td style="text-align:left;"> no </td> <td style="text-align:left;"> no </td> </tr> <tr> <td style="text-align:left;"> 5193 </td> <td style="text-align:left;"> 2020 </td> <td style="text-align:left;"> 44112 </td> <td style="text-align:left;"> Katsumura, T; Oda, S; Mitani, H; Oota, H </td> <td style="text-align:left;"> 2019 </td> <td style="text-align:left;"> Medaka Population Genome Structure and Demographic History Described via Genotyping-by-Sequencing </td> <td style="text-align:left;"> Medaka is a model organism in medicine, genetics, developmental biology and population genetics. Lab stocks composed of more than 100 local wild populations are available for research in these fields. Thus, medaka represents a potentially excellent bioresource for screening disease-risk- and adaptation-related genes in genome-wide association studies. Although the genetic population structure should be known before performing such an analysis, a comprehensive study on the genome-wide diversity of wild medaka populations has not been performed. Here, we performed genotyping-by-sequencing (GBS) for 81 and 12 medakas captured from a bioresource and the wild, respectively. Based on the GBS data, we evaluated the genetic population structure and estimated the demographic parameters using an approximate Bayesian computation (ABC) framework. The genome-wide data confirmed that there were substantial differences between local populations and supported our previously proposed hypothesis on medaka dispersal based on mitochondrial genome (mtDNA) data. A new finding was that a local group that was thought to be a hybrid between the northern and the southern Japanese groups was actually an origin of the northern Japanese group. Thus, this paper presents the first population-genomic study of medaka and reveals its population structure and history based on chromosomal genetic diversity. </td> <td style="text-align:left;"> NA </td> <td style="text-align:left;"> no </td> <td style="text-align:left;"> No good fitness measure and measure host GD </td> <td style="text-align:left;"> NA </td> <td style="text-align:left;"> no </td> <td style="text-align:left;"> no </td> </tr> <tr> <td style="text-align:left;"> 5194 </td> <td style="text-align:left;"> 2020 </td> <td style="text-align:left;"> 44042 </td> <td style="text-align:left;"> Kaye, TN; Bahm, MA; Thorpe, AS; Gray, EC; Pfingsten, I; Waddell, C </td> <td style="text-align:left;"> 2019 </td> <td style="text-align:left;"> Population extinctions driven by climate change, population size, and time since observation may make rare species databases inaccurate </td> <td style="text-align:left;"> Loss of biological diversity through population extinctions is a global phenomenon that threatens many ecosystems. Managers often rely on databases of rare species locations to plan land use actions and conserve at-risk taxa, so it is crucial that the information they contain is accurate and dependable. However, small population sizes, long gaps between surveys, and climate change may be leading to undetected extinctions of many populations. We used repeated survey records for a rare but widespread orchid, Cypripedium fasciculaturn (clustered lady's slipper), to model population extinction risk based on elevation, population size, and time between observations. Population size and elevation were negatively associated with extinction, while extinction probability increased with time between observations. We interpret population losses at low elevations as a potential signal of climate change impacts. We used this model to estimate the probability of persistence of populations across California and Oregon, and found that 39%-52% of the 2415 populations reported in databases from this region are likely extinct. Managers should be aware that the number of populations of rare species in their databases is potentially an overestimate, and consider resurveying these populations to document their presence and condition, with priority given to older reports of small populations, especially those at low elevations or in other areas with high vulnerability to climate or land cover change. </td> <td style="text-align:left;"> NA </td> <td style="text-align:left;"> no </td> <td style="text-align:left;"> no good gd measure host </td> <td style="text-align:left;"> NA </td> <td style="text-align:left;"> no </td> <td style="text-align:left;"> no </td> </tr> <tr> <td style="text-align:left;"> 5195 </td> <td style="text-align:left;"> 2020 </td> <td style="text-align:left;"> 44113 </td> <td style="text-align:left;"> Kean, EF; Bruford, MW; Russo, IRM; Muller, CT; Chadwick, EA </td> <td style="text-align:left;"> 2017 </td> <td style="text-align:left;"> Odour dialects among wild mammals </td> <td style="text-align:left;"> Across multiple taxa, population structure and dynamics depend on effective signalling between individuals. Among mammals, chemical communication is arguably the most important sense, underpinning mate choice, parental care, territoriality and even disease transmission. There is a growing body of evidence that odours signal genetic information that may confer considerable benefits including inbreeding avoidance and nepotism. To date, however, there has been no clear evidence that odours encode population-level information in wild mammals. Here we demonstrate for the first time the existence of 'odour dialects' in genetically distinct mammalian subpopulations across a large geographical scale. We found that otters, Lutra lutra, from across the United Kingdom possess sex and biogeography-specific odours. Subpopulations with the most distinctive odour profiles are also the most genetically diverse but not the most genetically differentiated. Furthermore, geographic distance between individuals does not explain regional odour differences, refuting other potential explanations such as group odour sharing behaviour. Differences in the language of odours between subpopulations have the potential to affect individual interactions, which could impact reproduction and gene-flow. </td> <td style="text-align:left;"> NA </td> <td style="text-align:left;"> no </td> <td style="text-align:left;"> No good fitness measure and measure host GD </td> <td style="text-align:left;"> NA </td> <td style="text-align:left;"> no </td> <td style="text-align:left;"> no </td> </tr> <tr> <td style="text-align:left;"> 5196 </td> <td style="text-align:left;"> 2020 </td> <td style="text-align:left;"> 44113 </td> <td style="text-align:left;"> Kempel, A; Rindisbacher, A; Fischer, M; Allan, E </td> <td style="text-align:left;"> 2018 </td> <td style="text-align:left;"> Plant soil feedback strength in relation to large-scale plant rarity and phylogenetic relatedness </td> <td style="text-align:left;"> Understanding why some species are rare while others are common remains a central and fascinating question in ecology. Recently, interactions with soil organisms have been shown to affect local abundances of plant species within communities, however, it is not known whether they might also drive patterns of rarity at large scales. Further, little is known about the specificity of soil-feedback effects, and whether closely related plants share more soil pathogens than more distantly related plants. In a multi-species soil-feedback experiment (using 19 species) we tested whether regionally and locally rare species differed in their response to soil biota. Regional rarity was measured using range size or IUCN status and local rarity by typical abundance within an area. All species were grown on soils trained by a variety of regionally and locally rare and common species, which also varied in their degree of relatedness to the target. We found that, in general, regionally rare species suffered more than twice as much from soil biota than regionally common species. Soil cultured by regionally rare species also had a more negative effect on subsequent plant growth, suggesting they may have also accumulated more pathogens. Local rarity did not predict feedback strength. Further, soil trained by closely related plants had a more negative effect on growth than soil trained by distant relatives, which indicates a phylogenetic signal in the host range of soil biota. We conclude that soil biota may well contribute to plant rarity at large spatial scales, which offers a novel explanation for plant rarity and commonness. Moreover, our results show that phylogenetic relatedness between plants was a good predictor of the likelihood that two plant species interacted negatively via soil biota, which might mean that soil pathogens could prevent the coexistence of closely related plants and could drive patterns of phylogenetic overdispersion. Our results suggest that soil pathogens could restrict the ability of rare species to shift their ranges and might need to be considered by conservation biologists seeking to protect populations of rare plants. </td> <td style="text-align:left;"> NA </td> <td style="text-align:left;"> no </td> <td style="text-align:left;"> No good fitness measure and measure host GD </td> <td style="text-align:left;"> NA </td> <td style="text-align:left;"> no </td> <td style="text-align:left;"> no </td> </tr> <tr> <td style="text-align:left;"> 5197 </td> <td style="text-align:left;"> 2020 </td> <td style="text-align:left;"> 44041 </td> <td style="text-align:left;"> Khan, A; Fujita, AW; Randle, N; Regidor-Cerrillo, J; Shaik, JS; Shen, K; Oler, AJ; Quinones, M; Latham, SM; Akanmori, BD; Cleaveland, S; Innes, EA; Ryan, U; Slapeta, J; Schares, G; Ortega-Mora, LM; Dubey, JP; Wastling, JM; Grigg, ME </td> <td style="text-align:left;"> 2019 </td> <td style="text-align:left;"> Global selective sweep of a highly inbred genome of the cattle parasite Neospora caninum </td> <td style="text-align:left;"> Neospora caninum, a cyst-forming apicomplexan parasite, is a leading cause of neuromuscular diseases in dogs as well as fetal abortion in cattle worldwide. The importance of the domestic and sylvatic life cycles of Neospora, and the role of vertical transmission in the expansion and transmission of infection in cattle, is not sufficiently understood. To elucidate the population genomics of Neospora, we genotyped 50 isolates collected worldwide from a wide range of hosts using 19 linked and unlinked genetic markers. Phylogenetic analysis and genetic distance indices resolved a single genotype of N. caninum. Whole-genome sequencing of 7 isolates from 2 different continents identified high linkage disequilibrium, significant structural variation, but only limited polymorphism genome-wide, with only 5,766 biallelic single nucleotide polymorphisms (SNPs) total. Greater than half of these SNPs (similar to 3,000) clustered into 6 distinct haploblocks and each block possessed limited allelic diversity (with only 4 to 6 haplotypes resolved at each cluster). Importantly, the alleles at each haploblock had independently segregated across the strains sequenced, supporting a unisexual expansion model that is mosaic at 6 genomic blocks. Integrating seroprevalence data from African cattle, our data support a global selective sweep of a highly inbred livestock pathogen that originated within European dairy stock and expanded transcontinentally via unisexual mating and vertical transmission very recently, likely the result of human activities, including recurrent migration, domestication, and breed development of bovid and canid hosts within similar proximities. </td> <td style="text-align:left;"> NA </td> <td style="text-align:left;"> no </td> <td style="text-align:left;"> gd of pathogen </td> <td style="text-align:left;"> NA </td> <td style="text-align:left;"> no </td> <td style="text-align:left;"> no </td> </tr> <tr> <td style="text-align:left;"> 5198 </td> <td style="text-align:left;"> 2020 </td> <td style="text-align:left;"> 44042 </td> <td style="text-align:left;"> Khan, S; Zhao, XC; Hou, YN; Yuan, CX; Li, YM; Luo, XP; Liu, JZ; Feng, XG </td> <td style="text-align:left;"> 2019 </td> <td style="text-align:left;"> Analysis of genome-wide SNPs based on 2b-RAD sequencing of pooled samples reveals signature of selection in different populations of Haemonchus contortus </td> <td style="text-align:left;"> The parasitic nematode Haemonchus contortus is one of the world's most important parasites of small ruminants that causes significant economic losses to the livestock sector. The population structure and selection in its various strains are poorly understood. No study so far compared its different populations using genome-wide data. Here, we focused on different geographic populations of H. contours from China (Tibet, TB; Hubei, HB; Inner Mongolia, IM; Sichuan, SC), UK and Australia (AS), using genome-wide population-genomic approaches, to explore genetic diversity, population structure and selection. We first performed next-generation high-throughput 2b RAD pool sequencing using Illumina technology, and identified single-nucleotide polymorphisms (SNPs) in all the strains. We identified 75,187 SNPs for TB, 82,271 for HB, 82,420 for IM, 79,803 for SC, 83,504 for AS and 78,747 for UK strain. The SNPs revealed low-nucleotide diversity (pi = 0.0092-0.0133) within each strain, and a significant differentiation level (average F-st = 0.34264) among them. Chinese populations TB and SC, along with the UK strain, were more divergent populations. Chinese populations IM and HB showed affinities to the Australian strain. We then analysed signature of selection and detected 44 (UK) and 03 (AS) private selective sweeps containing 49 and 05 genes, respectively. Finally, we performed the functional annotation of selective sweeps and proposed biological significance to signature of selection. Our data suggest that 2b-RAD pool sequencing can be used to assess the signature of selection in H. contortus. </td> <td style="text-align:left;"> NA </td> <td style="text-align:left;"> no </td> <td style="text-align:left;"> gd of pathogen </td> <td style="text-align:left;"> NA </td> <td style="text-align:left;"> no </td> <td style="text-align:left;"> no </td> </tr> <tr> <td style="text-align:left;"> 5199 </td> <td style="text-align:left;"> 2020 </td> <td style="text-align:left;"> 44112 </td> <td style="text-align:left;"> Khrustaleva, AM; Ponomareva, EV; Ponomareva, MV; Shubina, EA; Uglova, TY; Klovach, NV </td> <td style="text-align:left;"> 2018 </td> <td style="text-align:left;"> Inferring Adaptive Nature of Major Histocompatibility Complex (MHC) Polymorphism from Single Nucleotide Substitutions in Asian Sockeye Salmon Populations: I. Different Forms of Selection Act in Sockeye Salmon Populations from the Ozernaya and Kamchatka Rivers </td> <td style="text-align:left;"> Variability of the Onne-DAB gene, which encodes the -chain of class II major histocompatibility complex molecule (MHCII), is studied using One_MHC2_109, One_MHC2_190v2, and One_MHC2_251v2 single nucleotide polymorphisms (SNP) in the two largest Asian-Pacific coast populations of sockeye salmon that reproduce in the Ozernaya River and Kamchatka River basins. Differences in the nature of inheritance and degree of polymorphism of One_MHC2_190v2 and One_MHC2_251v2 are observed in samples from both lake-river systems, whereas One_MHC2_109 was uninformative. Samples collected from the lake and river spawning grounds of Lake Kurilskoe are characterized by high estimates of intrapopulation genetic diversity, whereas no intersample differences are revealed in the frequencies of haplotypes and phenotypes of the joint MHC2 locus. This can be interpreted as the result of balancing selection in sequences encoding the MHCII peptide-binding region in this sockeye salmon population. This hypothesis is also confirmed by Ewens-Watterson neutrality tests. On the contrary, low genetic diversity estimates and significant heterogeneity of haplotype and phenotypic frequencies at the MHC2 locus are observed in the samples of early (spring) sockeye salmon from the Kamchatka River basin, apparently owing to the action of directional pathogen-induced selection in the Onne-DAB gene in some localities of the lake-river system. </td> <td style="text-align:left;"> NA </td> <td style="text-align:left;"> no </td> <td style="text-align:left;"> No good fitness measure and measure host GD </td> <td style="text-align:left;"> NA </td> <td style="text-align:left;"> no </td> <td style="text-align:left;"> no </td> </tr> <tr> <td style="text-align:left;"> 5200 </td> <td style="text-align:left;"> 2020 </td> <td style="text-align:left;"> 44113 </td> <td style="text-align:left;"> Kiemnec-Tyburczy, KM; Tracy, KE; Lips, KR; Zamudio, KR </td> <td style="text-align:left;"> 2018 </td> <td style="text-align:left;"> Genetic variation and selection of MHC class I loci differ in two congeneric frogs </td> <td style="text-align:left;"> Major histocompatibility complex (MHC) genes encode proteins in the acquired immune response pathway that often show distinctive selection-driven patterns in wild vertebrate populations. We examined genetic variation and signatures of selection in the MHC class I alpha 1 (A1)- and alpha 2 (A2)-domain encoding exons of two frog congeners [Agalychnis callidryas (n = 20) and A. lemur (n = 20)] from a single locality in Panama. We also investigated how historical demographic processes may have impacted MHC genetic diversity by analyzing a neutral mitochondrial marker. We found that both MHC domains were highly variable in both species, with both species likely expressing three loci. Our analyses revealed different signatures of selection between the two species, most notably that the A. callidryas A2 domain had experienced positive selection while the A2 domain of A. lemur had not. Diversifying selection acted on the same number of A1 and A2 allelic lineages, but on a higher percentage of A1 sites compared to A2 sites. Neutrality tests of mitochondrial haplotypes predominately indicated that the two species were at genetic equilibrium when the samples were collected. In addition, two historical tests of demography indicated both species have had relatively stable population sizes over the past 100,000 years; thus large population size changes are unlikely to have greatly influenced MHC diversity in either species during this time period. In conclusion, our results suggest that the impact of selection on MHC diversity varied between these two closely related species, likely due to a combination of distinct ecological conditions and past pathogenic pressures. </td> <td style="text-align:left;"> NA </td> <td style="text-align:left;"> no </td> <td style="text-align:left;"> mhc diversity </td> <td style="text-align:left;"> NA </td> <td style="text-align:left;"> no </td> <td style="text-align:left;"> no </td> </tr> <tr> <td style="text-align:left;"> 5201 </td> <td style="text-align:left;"> 2020 </td> <td style="text-align:left;"> 44113 </td> <td style="text-align:left;"> Kierepka, EM; Kilgo, JC; Rhodes, OE </td> <td style="text-align:left;"> 2017 </td> <td style="text-align:left;"> Effect of Compensatory Immigration on the Genetic Structure of Coyotes </td> <td style="text-align:left;"> Despite efforts to reduce their effects on livestock and native ungulates within the southeastern United States, coyotes (Canis latrans) can recover from control programs. It is unknown how coyotes compensate for high mortality following trapping, so there is great interest to identify methods that can provide insight into coyote response to intensive trapping. To investigate if population genetic tools can decipher how coyotes recover from intensive trapping, we combined an empirical test of how genetic differentiation, diversity, and familial structure changed following trapping on the Savannah River Site (SRS), South Carolina, USA, with spatially explicit genetic simulations. The pre- and post-trapping periods had similar genetic diversities and were not genetically differentiated as expected by either compensatory reproduction or immigration from a single genetic source. The post-trapping coyote populations exhibited weaker signatures of philopatry with little evidence for increased dispersal distances of young coyotes, which suggests immigration caused a decrease in familial structure. Our simulations indicated that spatial autocorrelation coefficients and observed heterozygosities change as immigration increases, whereas population differentiation, allelic richness, and displacement distances do not. Collectively, our results suggest that coyotes recover from intensive trapping via reproduction and immigration, which likely makes preventing compensation difficult. Monitoring post-trapping populations may offer more insight into maximizing the effectiveness of control efforts, and based on our simulations, population genetics can provide critical information about the amount of compensatory immigration following trapping. (C) 2017 The Wildlife Society. </td> <td style="text-align:left;"> NA </td> <td style="text-align:left;"> no </td> <td style="text-align:left;"> No good fitness measure and measure host GD </td> <td style="text-align:left;"> NA </td> <td style="text-align:left;"> no </td> <td style="text-align:left;"> no </td> </tr> <tr> <td style="text-align:left;"> 5202 </td> <td style="text-align:left;"> 2020 </td> <td style="text-align:left;"> 44112 </td> <td style="text-align:left;"> Kilic, S; Taskin, V; Dogaroglu, T; Dogac, E; Gocmen Taskin, B </td> <td style="text-align:left;"> 2019 </td> <td style="text-align:left;"> Genetic characterization of field populations of Culex pipiens Linnaeus, 1758 (Diptera: Culicidae) sampled from the Aegean region of Turkey </td> <td style="text-align:left;"> Mosquitoes are one of the organisms subjected to frequent insecticide application due to their status as vectors that carry a wide range of life-threatening diseases. Turkey has climatic and other ecological features required for the breeding and living of 50 species from 8 genera. The members of the Culex pipiens complex are the dominant mosquitoes among them. In order to design more sustainable insecticide resistance management strategies, it is important to investigate the genetic structure of mosquito populations using molecular techniques. The aim of this study was the genetic characterization of field populations of C. pipiens sampled from 25 different sublocations belonging to 6 provinces in the Aegean region of Turkey by using random amplified polymorphic DNA (RAPD) markers. Eighty 10-mer RAPD primers were screened on a subset of DNAs. Among them, 20 reproducible and clear band-producing polymorphic primers were selected and applied to all study material. A low level of genetic differentiation and a high level of gene flow were detected between the populations. </td> <td style="text-align:left;"> NA </td> <td style="text-align:left;"> no </td> <td style="text-align:left;"> No good fitness measure </td> <td style="text-align:left;"> NA </td> <td style="text-align:left;"> no </td> <td style="text-align:left;"> no </td> </tr> <tr> <td style="text-align:left;"> 5203 </td> <td style="text-align:left;"> 2020 </td> <td style="text-align:left;"> 44040 </td> <td style="text-align:left;"> Kim, H; Cho, M; Son, HS </td> <td style="text-align:left;"> 2020 </td> <td style="text-align:left;"> Comparative analysis of codon usage patterns in Rift Valley fever virus </td> <td style="text-align:left;"> Rift Valley fever virus (RVFV) is a vector borne pathogen and is the most widely known virus in the genus Phiebovirus. Since it was first reported, RVFV has spread to western Africa, Egypt and Madagascar from its traditional endemic region, and infections continue to occur in new areas. In this study, we analyzed genomic patterns according to the infection properties of RVFV. Among the four segments of RVFV, the nucleotide composition, overall GC content and the difference of GC composition in the third position of the codons (%GC3) between groups were the largest in the S (NP) segment, showing that more diverse codons were used than in other segments. Furthermore, the results of CAI analysis of the S (NP) segment showed that viruses isolated from regions where no previous infections had been reported had the highest values, indicating greater adaptability to human hosts compared with other viruses. This result suggests that mutations in the S (NP) segment co-evolve with the infected hosts and may lead to expansion of the geographic range. The distinctive codon usage patterns observed in specific genomic regions of a group with similar infection properties may be related to the increasing likelihood of RVFV infections in new areas. </td> <td style="text-align:left;"> NA </td> <td style="text-align:left;"> no </td> <td style="text-align:left;"> gd of pathogen </td> <td style="text-align:left;"> NA </td> <td style="text-align:left;"> no </td> <td style="text-align:left;"> no </td> </tr> <tr> <td style="text-align:left;"> 5204 </td> <td style="text-align:left;"> 2020 </td> <td style="text-align:left;"> 44113 </td> <td style="text-align:left;"> Kim, JE; Goo, IB; Hwang, JA; Kim, HS; Choi, HS; Lee, JH </td> <td style="text-align:left;"> 2018 </td> <td style="text-align:left;"> Genetic variability comparison of cultured Israeli carp (Cyprinus carpio) from Korea using microsatellites </td> <td style="text-align:left;"> In aquaculture, cultured fish often undergo continuous cross-fertilization without any inflow of new broodstock. This lowers genetic diversity, leading to increased disease rates and decreased survival rates. To improve the mass production and easy culture of Israeli carp, it is essential to investigate the population structure and genetic diversity of these fish. However, such a survey has not yet been performed on Korean Israeli carp. In this study, we used seven microsatellite markers to analyze the genetic diversity and association of cultured Israeli carp from Korea and China. The average numbers of alleles per locus (N (A) ) for two Korean (KorA and KorB) and two Chinese (ChA and ChB) populations were as follows: KorA (10.42), KorB (14.43), ChA (20.57) and ChB (20.71). The expected heterozygosity (H (e) ) ranged from 0.672 to 0.897 and from 0.827 to 0.938 in the Korean sample and Chinese sample respectively. The genetic diversity of the Korean Israeli carp was about half that of the Chinese carp. The diversity of the Korean Israeli carp was very low, suggesting that the immunity of this population could be weak, and that diversity-recovery studies are urgently needed. Therefore, our results may therefore form the foundation for future research efforts towards genetic monitoring and selective breeding, continuous research needs to be conducted in order to recover the genetic diversity of the Korean Israeli carp. </td> <td style="text-align:left;"> NA </td> <td style="text-align:left;"> no </td> <td style="text-align:left;"> No good fitness measure </td> <td style="text-align:left;"> NA </td> <td style="text-align:left;"> no </td> <td style="text-align:left;"> no </td> </tr> <tr> <td style="text-align:left;"> 5205 </td> <td style="text-align:left;"> 2020 </td> <td style="text-align:left;"> 44042 </td> <td style="text-align:left;"> Kim, JY; Jeong, S; Kim, KH; Lim, WJ; Lee, HY; Kim, N </td> <td style="text-align:left;"> 2019 </td> <td style="text-align:left;"> Discovery of Genomic Characteristics and Selection Signatures in Korean Indigenous Goats Through Comparison of 10 Goat Breeds </td> <td style="text-align:left;"> Indigenous breeds develop their own genomic characteristics by adapting to local environments or cultures over long periods of time. Most of them are not particularly productive in commercial terms, but they have abilities to survive in harsh environments or tolerate to specific diseases. Their adaptive characteristics play an important role as genetic materials for improving commercial breeds. As a step toward this goal, we analyzed the genome of Korean indigenous goats within 10 goat breeds. We collected 136 goat individuals by sequencing 46 new goats and employing 90 publicly available goats. Our whole-genome data was comprised of three indigenous breeds (Korean indigenous goat, Iranian indigenous goat, and Moroccan indigenous goat; n = 29, 18, 20), six commercial breeds (Saanen, Boer, Anglo-Nubian, British Alpine, Alpine, and Korean crossbred; n = 16, 11, 5, 5, 2, 13), and their ancestral species (Capra aegagrus; n = 17). We identified that the Iranian indigenous goat and the Moroccan indigenous goat have relatively similar genomic characteristics within a large category of genomic diversity but found that the Korean indigenous goat has unique genomic characteristics distinguished from the other nine breeds. Through population analysis, we confirmed that these characteristics have resulted from a near-isolated environment with strong genetic drift. The Korean indigenous goat experienced a severe genetic bottleneck upon entering the Korean Peninsula about 2,000 years ago, and has subsequently rarely experienced genetic interactions with other goat breeds. From selection analysis and gene-set enrichment analysis, we revealed selection signals for Salmonella infection and cardiomyopathy in the genome of the Korean indigenous goat. These adaptive characteristics were further identified with genomic-based evidence. We uncovered genomic regions of selective sweeps in the LBP and BPI genes (Salmonella infection) and the TTN and ITGB6 genes (cardiomyopathy), among several candidate genes. Our research presents unique genomic characteristics and distinctive selection signals of the Korean indigenous goat based on the extensive comparison. Although the adaptive traits require further validation through biological experiments, our findings are expected to provide a direction for future biodiversity conservation strategies and to contribute another option to genomic-based breeding programmes for improving the viability of Capra hircus. </td> <td style="text-align:left;"> NA </td> <td style="text-align:left;"> no </td> <td style="text-align:left;"> No good fitness measure and measure host GD </td> <td style="text-align:left;"> NA </td> <td style="text-align:left;"> no </td> <td style="text-align:left;"> no </td> </tr> <tr> <td style="text-align:left;"> 5206 </td> <td style="text-align:left;"> 2020 </td> <td style="text-align:left;"> 44113 </td> <td style="text-align:left;"> Kindler, C; Gracia, E; Fritz, U </td> <td style="text-align:left;"> 2018 </td> <td style="text-align:left;"> Extra-Mediterranean glacial refuges in barred and common grass snakes (Natrix helvetica, N. natrix) </td> <td style="text-align:left;"> Extra-Mediterranean glacial refugia of thermophilic biota, in particular in northern latitudes, are controversial. In the present study we provide genetic evidence for extra-Mediterranean refugia in two species of grass snake. The refuge of a widely distributed western European lineage of the barred grass snake (Natrix helvetica) was most likely located in southern France, outside the classical refuges in the southern European peninsulas. One genetic lineage of the common grass snake (N. natrix), distributed in Scandinavia, Central Europe and the Balkan Peninsula, had two distinct glacial refuges. We show that one was located in the southern Balkan Peninsula. However, Central Europe and Scandinavia were not colonized from there, but from a second refuge in Central Europe. This refuge was located in between the northern ice sheet and the Alpine glaciers of the last glaciation and most likely in a permafrost region. Another co-distributed genetic lineage of N. natrix, now massively hybridizing with the aforementioned lineage, survived the last glaciation in a structured refuge in the southern Balkan Peninsula, according to the idea of 'refugia-within-refugia'. It reached Central Europe only very recently. This study reports for the first time the glacial survival of a thermophilic egg-laying reptile species in Central Europe. </td> <td style="text-align:left;"> NA </td> <td style="text-align:left;"> no </td> <td style="text-align:left;"> No good fitness measure and measure host GD </td> <td style="text-align:left;"> NA </td> <td style="text-align:left;"> no </td> <td style="text-align:left;"> no </td> </tr> <tr> <td style="text-align:left;"> 5207 </td> <td style="text-align:left;"> 2020 </td> <td style="text-align:left;"> 44039 </td> <td style="text-align:left;"> Kinhoegbe, G; Djedatin, G; Loko, LEY; Favi, AG; Adomou, A; Agbangla, C; Dansi, A </td> <td style="text-align:left;"> 2020 </td> <td style="text-align:left;"> On-farm management and participatory evaluation of pigeonpea (Cajanus cajan [L.] Millspaugh) diversity across the agro-ecological zones of the Republic of Benin </td> <td style="text-align:left;"> Background Pigeonpea is a multipurpose food legume crop that contributes to food security in the Republic of Benin. For the establishment of conservation and breeding programs, previous ethnobotanical surveys on pigeonpea were done in Benin but restricted to south and central regions. In previous years, pigeonpea landraces were introduced in northern Benin for soil fertility management; it is therefore important to evaluate the diversity in this legume in this region. Exhaustive documentation of pigeonpea diversity grown in the Republic of Benin will be necessary for effective breeding and conservation programs. Therefore, the aim of this study was to document genetic diversity of pigeonpea, across the agro-ecological zones of the Republic of Benin for its promotion and valorization. Methods A total of 500 pigeonpea farmers representing 13 sociolinguistic groups were selected from 50 villages. The data were collected using methods and tools of participatory research appraisal. Folk nomenclatures, taxonomy of pigeonpea and seed system were investigated. The distribution and extent of pigeonpea landraces were evaluated using the Four Square Analysis method. A comparative analysis of pigeonpea use categories production systems, production constraints, famers' preference criteria, and participative evaluation for existing landraces across agro-ecological zones was done. Result Folk nomenclature and taxonomy were mainly based on seed coat color and size. Seven pigeonpea use categories were recorded including sacrifice, grain processing and fertilization. The results showed that the pigeonpea seed system is informal. Based on seed characteristics, fifteen landraces were recorded including seven new landraces. The Sudano-Guinean zone contained the highest number (11) of landraces. The average number of landraces per village was 2.7. A high rate of landraces facing threat of disappearance was observed across the ecological zones. Ten constraints are known to affect pigeonpea production in Benin, with pests and diseases as the most critical in all agro-ecological zones. This study revealed that pigeonpea cultivation is increasing in the Sudanian zone. Varieties to be produced must be selected based on 11 criteria which included precocity and resistance to pests and diseases in the three ecological zones and adaptability to any type of soil in the Sudanian zone. The participatory evaluation revealed the existence of a few performing cultivars. Conclusions Our results revealed that implementation of a pigeonpea genetic conservation program in Benin must take into account the diversity, production constraints and varietal preference, which varied according to agro-ecological zones. In situ and ex situ conservation strategies are important to preserve pigeonpea landraces. Morphological and molecular characterizations of identified cultivars are highly recommended to help select suitable varieties for breeding programs. </td> <td style="text-align:left;"> NA </td> <td style="text-align:left;"> no </td> <td style="text-align:left;"> No good fitness measure and measure host GD </td> <td style="text-align:left;"> NA </td> <td style="text-align:left;"> no </td> <td style="text-align:left;"> no </td> </tr> <tr> <td style="text-align:left;"> 5208 </td> <td style="text-align:left;"> 2020 </td> <td style="text-align:left;"> 44113 </td> <td style="text-align:left;"> Kirk, KF; Meric, G; Nielsen, HL; Pascoe, B; Sheppard, SK; Thorlacius-Ussing, O; Nielsen, H </td> <td style="text-align:left;"> 2018 </td> <td style="text-align:left;"> Molecular epidemiology and comparative genomics of Campylobacter concisus strains from saliva, faeces and gut mucosal biopsies in inflammatory bowel disease </td> <td style="text-align:left;"> Campylobacter concisus is an emerging pathogen associated with inflammatory bowel disease (IBD), yet little is known about the genetic diversity of C. concisus in relation to host niches and disease. We isolated 104 C. concisus isolates from saliva, mucosal biopsies and faecal samples from 41 individuals (26 IBD, 3 Gastroenteritis (GE), 12 Healthy controls (HC)). Whole genomes were sequenced and the dataset pan-genome examined, and genomic information was used for typing using multi-locus-sequence typing (MLST). C. concisus isolates clustered into two main groups/genomospecies (GS) with 71 distinct sequence types (STs) represented. Sampling site (p &lt; 0.001), rather than disease phenotype (p = 1.00) was associated with particular GS. We identified 97 candidate genes associated with increase or decrease in prevalence during the anatomical descent from the oral cavity to mucosal biopsies to faeces. Genes related to cell wall/membrane biogenesis were more common in oral isolates, whereas genes involved in cell transport, metabolism and secretory pathways were more prevalent in enteric isolates. Furthermore, there was no correlation between individual genetic diversity and clinical phenotype. This study confirms the genetic heterogeneity of C. concisus and provides evidence that genomic variation is related to the source of isolation, but not clinical phenotype. </td> <td style="text-align:left;"> NA </td> <td style="text-align:left;"> no </td> <td style="text-align:left;"> gd of pathogen </td> <td style="text-align:left;"> NA </td> <td style="text-align:left;"> no </td> <td style="text-align:left;"> no </td> </tr> <tr> <td style="text-align:left;"> 5209 </td> <td style="text-align:left;"> 2020 </td> <td style="text-align:left;"> 44113 </td> <td style="text-align:left;"> Kjaerbolling, I; Vesth, TC; Frisvad, JC; Nybo, JL; Theobald, S; Kuo, A; Bowyer, P; Matsuda, Y; Mondo, S; Lyhne, EK; Kogle, ME; Clum, A; Lipzen, A; Salamov, A; Ngan, CY; Daum, C; Chiniquy, J; Barry, K; LaButti, K; Haridas, S; Simmons, BA; Magnuson, JK; Mortensen, UH; Larsen, TO; Grigoriev, IV; Baker, SE; Andersen, MR </td> <td style="text-align:left;"> 2018 </td> <td style="text-align:left;"> Linking secondary metabolites to gene clusters through genome sequencing of six diverse Aspergillus species </td> <td style="text-align:left;"> The fungal genus of Aspergillus is highly interesting, containing everything from industrial cell factories, model organisms, and human pathogens. In particular, this group has a prolific production of bioactive secondary metabolites (SMs). In this work, four diverse Aspergillus species (A. campestris, A. novofumigatus, A. ochraceoroseus, and A. steynii) have been whole-genome PacBio sequenced to provide genetic references in three Aspergillus sections. A. taichungensis and A. candidus also were sequenced for SM elucidation. Thirteen Aspergillus genomes were analyzed with comparative genomics to determine phylogeny and genetic diversity, showing that each presented genome contains 15-27% genes not found in other sequenced Aspergilli. In particular, A. novofumigatus was compared with the pathogenic species A. fumigatus. This suggests that A. novofumigatus can produce most of the same allergens, virulence, and pathogenicity factors as A. fumigatus, suggesting that A. novofumigatus could be as pathogenic as A. fumigatus. Furthermore, SMs were linked to gene clusters based on biological and chemical knowledge and analysis, genome sequences, and predictive algorithms. We thus identify putative SM clusters for aflatoxin, chlorflavonin, and ochrindol in A. ochraceoroseus, A. campestris, and A. steynii, respectively, and novofumigatonin, ent-cycloechinulin, and epi-aszonalenins in A. novofumigatus. Our study delivers six fungal genomes, showing the large diversity found in the Aspergillus genus; highlights the potential for discovery of beneficial or harmful SMs; and supports reports of A. novofumigatus pathogenicity. It also shows how biological, biochemical, and genomic information can be combined to identify genes involved in the biosynthesis of specific SMs. </td> <td style="text-align:left;"> NA </td> <td style="text-align:left;"> no </td> <td style="text-align:left;"> No good fitness measure </td> <td style="text-align:left;"> NA </td> <td style="text-align:left;"> no </td> <td style="text-align:left;"> no </td> </tr> <tr> <td style="text-align:left;"> 5210 </td> <td style="text-align:left;"> 2020 </td> <td style="text-align:left;"> 44039 </td> <td style="text-align:left;"> Klass, K; Van Belle, S; Estrada, A </td> <td style="text-align:left;"> 2020 </td> <td style="text-align:left;"> Demographic population structure of black howler monkeys in fragmented and continuous forest in Chiapas, Mexico: Implications for conservation </td> <td style="text-align:left;"> For wild primates, demography studies are increasingly recognized as necessary for assessing the viability of vulnerable populations experiencing rapid environmental change. In particular, anthropogenic changes such as habitat loss and fragmentation can cause ecological and behavioral changes in small, isolated populations, which may, over time, alter population density and demographic structure (age/sex classes and group composition) in fragment populations relative to continuous forest populations. We compared our study population of Endangered black howler monkeys (Alouatta pigra) in 34 forest fragments around Palenque National Park (PNP), Mexico (62 groups, 407 individuals), to the adjacent population in PNP, protected primary forest (21 groups, 134 individuals), and to previous research on black howlers in fragments in our study area (18 groups, 115 individuals). We used chi(2)and Mann-WhitneyUtests to address the questions: (a) what is the current black howler demographic population structure in unprotected forest fragments around PNP? (b) How does it compare to PNP's stable, continuous population? (c) How has it changed over time? Compared to the PNP population, the fragment populations showed higher density, a significantly lower proportion of multimale groups, and significantly fewer adult males per group. The population's age/sex structure in the fragmented landscape has been stable over the last 17 years, but differed in a higher proportion of multifemale groups, higher density, and higher patch occupancy in the present. In the context of conservation, some of our results may be positive as they indicate possible population growth over time. However, long-term scarcity of adult males in fragments and associated effects on population demographic structure might be cause for concern, in that it may affect gene flow and genetic diversity. The scarcity of adult males might stem from males experiencing increased mortality while dispersing in the fragmented landscape, whereas females might be becoming more philopatric in fragments. </td> <td style="text-align:left;"> NA </td> <td style="text-align:left;"> no </td> <td style="text-align:left;"> No good fitness measure and measure host GD </td> <td style="text-align:left;"> NA </td> <td style="text-align:left;"> no </td> <td style="text-align:left;"> no </td> </tr> <tr> <td style="text-align:left;"> 5211 </td> <td style="text-align:left;"> 2020 </td> <td style="text-align:left;"> 44111 </td> <td style="text-align:left;"> Klunk, J; Duggan, AT; Redfern, R; Gamble, J; Boldsen, JL; Golding, GB; Walter, BS; Eaton, K; Stangroom, J; Rouillard, JM; Devault, A; Dewitte, SN; Poinar, HN </td> <td style="text-align:left;"> 2019 </td> <td style="text-align:left;"> Genetic resiliency and the Black Death: No apparent loss of mitogenomic diversity due to the Black Death in medieval London and Denmark </td> <td style="text-align:left;"> Objectives In the 14th century AD, medieval Europe was severely affected by the Great European Famine as well as repeated bouts of disease, including the Black Death, causing major demographic shifts. This high volatility led to increased mobility and migration due to new labor and economic opportunities, as evidenced by documentary and stable isotope data. This study uses ancient DNA (aDNA) isolated from skeletal remains to examine whether evidence for large-scale population movement can be gleaned from the complete mitochondrial genomes of 264 medieval individuals from England (London) and Denmark. Materials and Methods Using a novel library-conserving approach to targeted capture, we recovered 264 full mitochondrial genomes from the petrous portion of the temporal bones and teeth and compared genetic diversity across the medieval period within and between English (London) and Danish populations and with contemporary populations through population pairwise phi(ST) analysis. Results We find no evidence of significant differences in genetic diversity spatially or temporally in our dataset, yet there is a high degree of haplotype diversity in our medieval samples with little exact sequence sharing. Discussion The mitochondrial genomes of both medieval Londoners and medieval Danes suggest high mitochondrial diversity before, during and after the Black Death. While our mitochondrial genomic data lack geographically correlated signals, these data could be the result of high, continual female migration before and after the Black Death or may simply indicate a large female effective population size unaffected by the upheaval of the medieval period. Either scenario suggests a genetic resiliency in areas of northwestern medieval Europe. </td> <td style="text-align:left;"> NA </td> <td style="text-align:left;"> no </td> <td style="text-align:left;"> human </td> <td style="text-align:left;"> NA </td> <td style="text-align:left;"> no </td> <td style="text-align:left;"> no </td> </tr> <tr> <td style="text-align:left;"> 5212 </td> <td style="text-align:left;"> 2020 </td> <td style="text-align:left;"> 44111 </td> <td style="text-align:left;"> Koenig, D; Hagmann, J; Li, R; Bemm, F; Slotte, T; Nueffer, B; Wright, SI; Weigel, D </td> <td style="text-align:left;"> 2019 </td> <td style="text-align:left;"> Long-term balancing selection drives evolution of immunity genes in Capsella </td> <td style="text-align:left;"> Genetic drift is expected to remove polymorphism from populations over long periods of time, with the rate of polymorphism loss being accelerated when species experience strong reductions in population size. Adaptive forces that maintain genetic variation in populations, or balancing selection, might counteract this process. To understand the extent to which natural selection can drive the retention of genetic diversity, we document genomic variability after two parallel species-wide bottlenecks in the genus Capsella. We find that ancestral variation preferentially persists at immunity related loci, and that the same collection of alleles has been maintained in different lineages that have been separated for several million years. By reconstructing the evolution of the disease-related locus MLO2b, we find that divergence between ancient haplotypes can be obscured by referenced based re-sequencing methods, and that trans-specific alleles can encode substantially diverged protein sequences. Our data point to long-term balancing selection as an important factor shaping the genetics of immune systems in plants and as the predominant driver of genomic variability after a population bottleneck. </td> <td style="text-align:left;"> NA </td> <td style="text-align:left;"> no </td> <td style="text-align:left;"> No good fitness measure and measure host GD </td> <td style="text-align:left;"> NA </td> <td style="text-align:left;"> no </td> <td style="text-align:left;"> no </td> </tr> <tr> <td style="text-align:left;"> 5213 </td> <td style="text-align:left;"> 2020 </td> <td style="text-align:left;"> 44043 </td> <td style="text-align:left;"> Kolodziej-Sobocinska, M; Stojak, J; Kondzior, E; Ruczynska, I; Wojcik, JM </td> <td style="text-align:left;"> 2019 </td> <td style="text-align:left;"> Genetic diversity of two mitochondrial DNA genes in Spirometra erinaceieuropaei (Cestoda: Diphyllobothridae) from Poland </td> <td style="text-align:left;"> The tapeworm species Spirometra erinaceieuropaei was documented mainly in Asia and Europe. In recent years, plerocercoid larvae (spargana) of this parasite have been found in different hosts in north-eastern Poland. The evolutionary history and way of S. erinaceieuropaei spreading across Eurasia have been not described yet. However, this phenomenon could be closely related to the evolutionary history and migration routes of studied tapeworm host species. We investigated the genetic variability and divergence pattern among S. erinaceieuropaei populations in intermediate and paratenic hosts from north-eastern Poland based on complete mitochondrial sequences of cytochrome b (cytb) and cytochrome c oxidase subunit I (cox1) genes. Analysis of 319 consolidated sequences of these two genes showed no genetic structure across study area. Comparison of sequences from Poland and China showed distinct separation of S. erinaceieuropaei populations from these two regions. They split from their common ancestor approximately 28.6 million years ago. Demographic expansion of Polish population of S. erinaceieuropaei started from glacial refugia approximately 12.5 thousand years ago, and recent population expansion has been observed in the tapeworm population from north-eastern Poland. </td> <td style="text-align:left;"> NA </td> <td style="text-align:left;"> no </td> <td style="text-align:left;"> gd of pathogen </td> <td style="text-align:left;"> NA </td> <td style="text-align:left;"> no </td> <td style="text-align:left;"> no </td> </tr> <tr> <td style="text-align:left;"> 5214 </td> <td style="text-align:left;"> 2020 </td> <td style="text-align:left;"> 44113 </td> <td style="text-align:left;"> Koo, KS; Kim, TW; Yang, KS; Oh, HS </td> <td style="text-align:left;"> 2018 </td> <td style="text-align:left;"> First observation on the breeding behavior of endangered species, Chinese many-tooth snake, Sibynophis chinensis </td> <td style="text-align:left;"> We report the first observation of reproductive behavior of endangered snake, Sibynophis chinensis in South Korea. On September 24, 2014, one female and three males were observed during the group mating in Oedo-dong, Jeju. Our observation indicates that group mating and the breeding behavior of S. chinensis could be an important way to increase genetic diversity and survival rate. Moreover, autumnal mating affects the breeding success rate of species in isolated areas. Group and autumn mating strategies by S. chinensis are considered to be the most effective way to survive in isolated areas. (C) 2018 National Science Museum of Korea (NSMK) and Korea National Arboretum (KNA), Publishing Services by Elsevier. </td> <td style="text-align:left;"> NA </td> <td style="text-align:left;"> no </td> <td style="text-align:left;"> No good fitness measure and measure host GD </td> <td style="text-align:left;"> NA </td> <td style="text-align:left;"> no </td> <td style="text-align:left;"> no </td> </tr> <tr> <td style="text-align:left;"> 5215 </td> <td style="text-align:left;"> 2020 </td> <td style="text-align:left;"> 44043 </td> <td style="text-align:left;"> Kordes, A; Preusse, M; Willger, SD; Braubach, P; Jonigk, D; Haverich, A; Warnecke, G; Haussler, S </td> <td style="text-align:left;"> 2019 </td> <td style="text-align:left;"> Genetically diverse Pseudomonas aeruginosa populations display similar transcriptomic profiles in a cystic fibrosis explanted lung </td> <td style="text-align:left;"> Previous studies have demonstrated substantial genetic diversification of Pseudomonas aeruginosa across sub-compartments in cystic fibrosis (CF) lungs. Here, we isolate P. aeruginosa from five different sampling areas in the upper and lower airways of an explanted CF lung, analyze ex vivo transcriptional profiles by RNA-seq, and use colony re-sequencing and deep population sequencing to determine the genetic diversity within and across the various sub-compartments. We find that, despite genetic variation, the ex vivo transcriptional profiles of P. aeruginosa populations inhabiting different regions of the CF lung are similar. Although we cannot estimate the extent to which the transcriptional response recorded here actually reflects the in vivo transcriptomes, our results indicate that there may be a common in vivo transcriptional profile in the CF lung environment. </td> <td style="text-align:left;"> NA </td> <td style="text-align:left;"> no </td> <td style="text-align:left;"> gd of pathogen </td> <td style="text-align:left;"> NA </td> <td style="text-align:left;"> no </td> <td style="text-align:left;"> no </td> </tr> <tr> <td style="text-align:left;"> 5216 </td> <td style="text-align:left;"> 2020 </td> <td style="text-align:left;"> 44113 </td> <td style="text-align:left;"> Kotsakiozi, P; Richardson, JB; Pichler, V; Favia, G; Martins, AJ; Urbanelli, S; Armbruster, PA; Caccone, A </td> <td style="text-align:left;"> 2017 </td> <td style="text-align:left;"> Population genomics of the Asian tiger mosquito, Aedes albopictus: insights into the recent worldwide invasion </td> <td style="text-align:left;"> Aedes albopictus, the Asian tiger mosquito, is an aggressive biting mosquito native to Asia that has colonized all continents except Antarctica during the last similar to 30-40years. The species is of great public health concern as it can transmit at least 26 arboviruses, including dengue, chikungunya, and Zika viruses. In this study, using double-digest Restriction site-Associated DNA (ddRAD) sequencing, we developed a panel of similar to 58,000 single nucleotide polymorphisms (SNPs) based on 20 worldwide Ae.albopictus populations representing both the invasive and the native range. We used this genomic-based approach to study the genetic structure and the differentiation of Ae.albopictus populations and to understand origin(s) and dynamics of the recent invasions. Our analyses indicated the existence of two major genetically differentiated population clusters, each one including both native and invasive populations. The detection of additional genetic structure within each major cluster supports that these SNPs can detect differentiation at a global and local scale, while the similar levels of genomic diversity between native and invasive range populations support the scenario of multiple invasions or colonization by a large number of propagules. Finally, our results revealed the possible source(s) of the recent invasion in Americas, Europe, and Africa, a finding with important implications for vector-control strategies. </td> <td style="text-align:left;"> NA </td> <td style="text-align:left;"> no </td> <td style="text-align:left;"> No good fitness measure and measure host GD </td> <td style="text-align:left;"> NA </td> <td style="text-align:left;"> no </td> <td style="text-align:left;"> no </td> </tr> <tr> <td style="text-align:left;"> 5217 </td> <td style="text-align:left;"> 2020 </td> <td style="text-align:left;"> 44043 </td> <td style="text-align:left;"> Kotze, A; Smith, RM; Moodley, Y; Luikart, G; Birss, C; Van Wyk, AM; Grobler, JP; Dalton, DL </td> <td style="text-align:left;"> 2019 </td> <td style="text-align:left;"> Lessons for conservation management: Monitoring temporal changes in genetic diversity of Cape mountain zebra (Equus zebra zebra) </td> <td style="text-align:left;"> The Cape mountain zebra (Equus zebra zebra) is a subspecies of mountain zebra endemic to South Africa. The Cape mountain zebra experienced near extinction in the early 1900's and their numbers have since recovered to more than 4,800 individuals. However, there are still threats to their long-term persistence. A previous study reported that Cape mountain zebra had low genetic diversity in three relict populations and that urgent conservation management actions were needed to mitigate the risk of further loss. As these suggestions went largely unheeded, we undertook the present study, fifteen years later to determine the impact of management on genetic diversity in three key populations. Our results show a substantial loss of heterozygosity across the Cape mountain zebra populations studied. The most severe losses occurred at De Hoop Nature Reserve where expected heterozygosity reduced by 22.85% from 0.385 to 0.297. This is alarming, as the De Hoop Nature Reserve was previously identified as the most genetically diverse population owing to its founders originating from two of the three remaining relict stocks. Furthermore, we observed a complete loss of multiple private alleles from all populations, and a related reduction in genetic structure across the subspecies. These losses could lead to inbreeding depression and reduce the evolutionary potential of the Cape mountain zebra. We recommend immediate implementation of evidence-based genetic management and monitoring to prevent further losses, which could jeopardise the long term survival of Cape mountain zebra, especially in the face of habitat and climate change and emerging diseases. </td> <td style="text-align:left;"> NA </td> <td style="text-align:left;"> no </td> <td style="text-align:left;"> No good fitness measure and measure host GD </td> <td style="text-align:left;"> NA </td> <td style="text-align:left;"> no </td> <td style="text-align:left;"> no </td> </tr> <tr> <td style="text-align:left;"> 5218 </td> <td style="text-align:left;"> 2020 </td> <td style="text-align:left;"> 44042 </td> <td style="text-align:left;"> Kowalski, A </td> <td style="text-align:left;"> 2019 </td> <td style="text-align:left;"> A status of guinea fowl (Numida meleagris) and pheasant (Phasianus colchicus) population transferred from wildlife to the breeding assessed based on the histone H1.c ' polymorphic variation </td> <td style="text-align:left;"> The genetic changes accompanying a relocation of population to the captivity are mostly adverse and usually associated with deterioration of its status. These alterations are greater in small populations in which a loss of genetic variation limits the capability to adaptation. In this work, a status of small-sized guinea fowl and pheasant population relocated to the breeding is presented. These populations were analyzed based on the polymorphism of histone H1.c', the protein for the first time identified as a heterogeneous. Histone H1.c' was resolved in the two-dimensional polyacrylamide gel into the isoform H1.c'1 and H1.c'2, so its heterogeneity corresponds to the presence of homozygous phenotypes c'1 and c'2. Because no histone H1.c' heterozygous phenotype was found, a significant phenotypic diversity in the guinea fowl (P=0.023) and pheasant (P=0.018) population was detected, together with its departures from Hardy-Weinberg equilibrium (P &lt; 0.0001). Both populations characterize an extreme loss of genetic diversity due to complete inbreeding (F= 1) and an impact of genetic drift which, according to the expected values for guinea fowl (0.192) and pheasant (0.182) population, may strongly diminish allele frequency in the following generations. Thus, condition of populations evaluated based on the histone H1.c' polymorphic variants, recognized as reasonable informative genetic markers (polymorphism information content of guinea fowl= 0.4 and pheasant= 0.38), corresponds to reduction of genetic variability caused by inbreeding and genetic drift. Therefore, it seems that rearing in the captivity can bring negative effects that favor restriction of animals' vitality and survival of the population. </td> <td style="text-align:left;"> NA </td> <td style="text-align:left;"> no </td> <td style="text-align:left;"> No good fitness measure and measure host GD </td> <td style="text-align:left;"> NA </td> <td style="text-align:left;"> no </td> <td style="text-align:left;"> no </td> </tr> <tr> <td style="text-align:left;"> 5219 </td> <td style="text-align:left;"> 2020 </td> <td style="text-align:left;"> 44113 </td> <td style="text-align:left;"> Kramarenko, SS; Lugovoy, SI; Kharzinova, VR; Lykhach, VY; Kramarenko, AS; Lykhach, AV </td> <td style="text-align:left;"> 2018 </td> <td style="text-align:left;"> Genetic diversity of Ukrainian local pig breeds based on microsatellite markers </td> <td style="text-align:left;"> Preserving the current diversity of the living material on Earth is fundamental for the survival of future generations. A study was conducted to investigate the genetic diversity of Ukrainian local pig breeds. A total of 350 pigs representing five local pig breeds from Ukraine (Mirgorod - MIR, Poltava Meat - PM, Ukrainian Meat - UM, Ukrainian White Steppe - UWS and Ukrainian Spotted Steppe - USS) and one commercial breed (Duroc, DUR) were sampled. Twelve microsatellite loci (SW24, S0155, SW72, SW951, S0386, S0355, SW240, SW857, S0101, SW936, SW911 and S0228) were selected and belong to the list of microsatellite markers recommended by ISAG. The results indicate that there exists, in general, a high degree of genetic variability within the five Ukrainian local pig breeds. However, the genetic variability in the MIR and PM breeds was significantly lower (mean Na = 2.92-3.92; Ho = 0.382-0.411; F-IS = 0.178-0.184) than in the other three Ukrainian local pig breeds - UM, UWS and USS (mean Na = 5.00-8.42; Ho = 0.549-0.668; F-IS = 0.027-0.066). Thirty-four private alleles were identified among the six analyzed genetic groups which were distributed between 11 of the 12 loci. A high number of alleles typical for the breed (private alleles) was observed in Duroc pigs - 9 alleles did not occur in Ukrainian local pig breeds. The HWE test showed that all of the polymorphic loci deviated from HWE (P &lt; 0.05) in at least one population. Loci S0355 (5), S0386 (4) and SW24 (4) presented a higher number of populations in imbalance. The mean F-ST showed that approximately 77.8% of the genetic variation was within-population and 12.2% was across the populations. The five Ukrainian local breeds were classified into two major groups, according to the phylogenetic tree, which was based on standard genetic distance. Overall, we found that 92.6% of the individual pigs were correctly assigned (324 out of 350) to the respective breed of origin, which is likely a consequence of the well-defined breed structure. Probabilities from the allocation test of individuals for the six pig genetic groups were estimated with Structure Harvester. In cluster 1 the highest grouping probabilities were found for the MIR (0.917) and PM (0.750) breeds. Local breeds UM (0.824) and USS (0.772) were grouped in cluster 2. Cluster 3 was related to the local pig breed USW (0.873). Cluster 4 presented high allocation probabilities for the commercial pig breed Duroc (0.924). The obtained results are important for the future conservation of Ukrainian local pig breeds. </td> <td style="text-align:left;"> NA </td> <td style="text-align:left;"> no </td> <td style="text-align:left;"> dometicated host </td> <td style="text-align:left;"> NA </td> <td style="text-align:left;"> no </td> <td style="text-align:left;"> no </td> </tr> <tr> <td style="text-align:left;"> 5220 </td> <td style="text-align:left;"> 2020 </td> <td style="text-align:left;"> 44113 </td> <td style="text-align:left;"> Krapohl, E; Hannigan, LJ; Pingault, JB; Patel, H; Kadeva, N; Curtis, C; Breen, G; Newhouse, SJ; Eley, TC; O'Reilly, PF; Plomin, R </td> <td style="text-align:left;"> 2017 </td> <td style="text-align:left;"> Widespread covariation of early environmental exposures and trait-associated polygenic variation </td> <td style="text-align:left;"> Although gene-environment correlation is recognized and investigated by family studies and recently by SNP-heritability studies, the possibility that genetic effects on traits capture environmental risk factors or protective factors has been neglected by polygenic prediction models. We investigated covariation between trait-associated polygenic variation identified by genome-wide association studies (GWASs) and specific environmental exposures, controlling for overall genetic relatedness using a genomic relatedness matrix restricted maximum-likelihood model. In a UK-representative sample (n = 6,710), we find widespread covariation between offspring trait-associated polygenic variation and parental behavior and characteristics relevant to children's developmental outcomes-independently of population stratification. For instance, offspring genetic risk for schizophrenia was associated with paternal age (R-2 = 0.002; P = 1e-04), and offspring education-associated variation was associated with variance in breastfeeding (R-2 = 0.021; P = 7e-30), maternal smoking during pregnancy (R-2 = 0.008; P = 5e-13), parental smacking (R-2 = 0.01; P = 4e-15), household income (R-2 = 0.032; P = 1e-22), watching television (R-2 = 0.034; P = 5e-47), and maternal education (R-2 = 0.065; P = 3e-96). Education-associated polygenic variation also captured covariation between environmental exposures and children's inattention/hyperactivity, conduct problems, and educational achievement. The finding that genetic variation identified by trait GWASs partially captures environmental risk factors or protective factors has direct implications for risk prediction models and the interpretation of GWAS findings. </td> <td style="text-align:left;"> NA </td> <td style="text-align:left;"> no </td> <td style="text-align:left;"> No good fitness measure and measure host GD </td> <td style="text-align:left;"> NA </td> <td style="text-align:left;"> no </td> <td style="text-align:left;"> no </td> </tr> <tr> <td style="text-align:left;"> 5221 </td> <td style="text-align:left;"> 2020 </td> <td style="text-align:left;"> 44112 </td> <td style="text-align:left;"> Kreyling, J; Puechmaille, SJ; Malyshev, AV; Valladares, F </td> <td style="text-align:left;"> 2019 </td> <td style="text-align:left;"> Phenotypic plasticity closely linked to climate at origin and resulting in increased mortality under warming and frost stress in a common grass </td> <td style="text-align:left;"> Phenotypic plasticity is important for species responses to global change and species coexistence. Phenotypic plasticity differs among species and traits and changes across environments. Here, we investigated phenotypic plasticity of the widespread grass Arrhenatherum elatius in response to winter warming and frost stress by comparing phenotypic plasticity of 11 geographically and environmentally distinct populations of this species to phenotypic plasticity of populations of different species originating from a single environment. The variation in phenotypic plasticity was similar for populations of a single species from different locations compared to populations of functionally and taxonomically diverse species from one environment for the studied traits (leaf biomass production and root integrity after frost) across three indices of phenotypic plasticity (RDPI, PIN, slope of reaction norm). Phenotypic plasticity was not associated with neutral genetic diversity but closely linked to the climate of the populations' origin. Populations originating from warmer and more variable climates showed higher phenotypic plasticity. This indicates that phenotypic plasticity can itself be considered as a trait subject to local adaptation to climate. Finally, our data emphasize that high phenotypic plasticity is not per se positive for adaptation to climate change, as differences in stress responses are resulting in high phenotypic plasticity as expressed by common plasticity indices, which is likely to be related to increased mortality under stress in more plastic populations. </td> <td style="text-align:left;"> NA </td> <td style="text-align:left;"> no </td> <td style="text-align:left;"> no good gd measure host pop </td> <td style="text-align:left;"> NA </td> <td style="text-align:left;"> no </td> <td style="text-align:left;"> no </td> </tr> <tr> <td style="text-align:left;"> 5222 </td> <td style="text-align:left;"> 2020 </td> <td style="text-align:left;"> 44112 </td> <td style="text-align:left;"> Kriangwanich, W; Nganvongpanit, K; Buddhachat, K; Brown, JL; Siengdee, P; Chomdej, S; Bansiddhi, P; Thitaram, C </td> <td style="text-align:left;"> 2018 </td> <td style="text-align:left;"> Genetic Diversity and Variation in Captive Asian Elephants (Elephas maximus) in Thailand </td> <td style="text-align:left;"> Numbers of wild Asian elephants (Elephas maximus) have been decreasing gradually throughout Asia due primarily to human activities, such as poaching, and habitat encroachment and destruction that lead to human-elephant conflict. Sustainability problems exist in captive populations as well, where morbidity and mortality rates are high and reproduction is low. Determining the genetic diversity of these populations is essential for conservation and sustainable utilization efforts. Intersimple sequence repeat markers were used to assess the genetic variation and differentiation in 97 captive Asian elephants from seven elephant camps in Chiang Mai, Thailand. The nine primers chosen for the analysis revealed 88 bands in male and 115 bands in female elephants, of which 37 (42.05%) and 83 (63.64%) were polymorphic, respectively. Shannon's index information (I = 2.415 +/- 0.054) and expected heterozygosity (He = 0.892 +/- 0.008) indicated high species level genetic diversity. The fixation index (F-st) was 0. 130 +/- 0.016, demonstrating there was no genetic subdivision between populations. A cluster analysis was performed using Unweight Pair-Group Method with Arithmetic Mean and dendrograms, which illustrated genetic relationships among captive Asian elephants that included 2 main clusters across the seven camps and 27 clusters for the 97 individual elephants. This high variability may be due to the different origins of these individuals, including originating from other Asian countries. Thus, this study showed that intersimple sequence repeat marker analysis was effective in demonstrating high genetic diversity among captive Asian elephants in Chiang Mai province and found cluster differences that could be used to guide breeding management to decrease the risk of inbreeding among Asian elephant groups. </td> <td style="text-align:left;"> NA </td> <td style="text-align:left;"> no </td> <td style="text-align:left;"> No good fitness measure </td> <td style="text-align:left;"> NA </td> <td style="text-align:left;"> no </td> <td style="text-align:left;"> no </td> </tr> <tr> <td style="text-align:left;"> 5223 </td> <td style="text-align:left;"> 2020 </td> <td style="text-align:left;"> 44111 </td> <td style="text-align:left;"> Krojerova-Prokesova, J; Turbakova, B; Jelencic, M; Bojda, M; Kutal, M; Skrbinsek, T; Koubek, P; Bryja, J </td> <td style="text-align:left;"> 2019 </td> <td style="text-align:left;"> Genetic constraints of population expansion of the Carpathian lynx at the western edge of its native distribution range in Central Europe </td> <td style="text-align:left;"> Even though populations of many large carnivores are expanding throughout Europe, the Eurasian lynx population in the Western Carpathians seems unable to spread beyond the western boundaries of its current distributional range. Many factors, both extrinsic and intrinsic, can influence the potential for range expansion: landscape fragmentation, natal philopatry, low natural fecundity and high mortality, and low and sex-biased dispersal rates. In this study we used non-invasive genetic sampling to determine population size fluctuation, sub-structuring and social organisation of the peripheral lynx population at the Czech-Slovak border. Even though the population size has been relatively stable over the period studied (2010-2016), the individual inbreeding coefficients of residents at the end of the study were much higher than those of founders at the beginning of the study. While non-resident individuals (predominantly males) occurred regularly in the study population, only resident individuals with well-established home ranges participated in breeding and produced offspring. Almost half the offspring detected in the study (predominantly females) settled in or near the natal area. Subsequent incestuous mating resulted in production of inbred individuals, reduction of effective population size of the population, and sub-structuring of the population through formation of two distinct family lineages. Our study illustrates how social constraints, such as territoriality, breeding of residents and natal philopatry of females, lead to incestuous mating in small-sized populations, especially at the periphery of their distribution. This threat should be taken into account in planning of conservation and population recovery of species with similar social structure. </td> <td style="text-align:left;"> NA </td> <td style="text-align:left;"> no </td> <td style="text-align:left;"> No good fitness measure and measure host GD </td> <td style="text-align:left;"> NA </td> <td style="text-align:left;"> no </td> <td style="text-align:left;"> no </td> </tr> <tr> <td style="text-align:left;"> 5224 </td> <td style="text-align:left;"> 2020 </td> <td style="text-align:left;"> 44040 </td> <td style="text-align:left;"> Kropf, M; Bardy, K; Hohn, M; Plenk, K </td> <td style="text-align:left;"> 2020 </td> <td style="text-align:left;"> Phylogeographical structure and genetic diversity of Adonis vernalis L. (Ranunculaceae) across and beyond the Pannonian region </td> <td style="text-align:left;"> Hypotheses of peripherality have characterised peripheral populations by increasing population differentiation and by declining genetic diversity towards the absolute range edge. However, such biogeographical patterns are not generally confirmed. Moreover, it was recently argued that lower levels of genetic diversity at the western and eastern periphery of the Eurasian steppe belt are only detectable by full-range longitudinal sampling. To uncover common genetic patterns of Central European steppe plants we here investigated Adonis vernalis populations across a transect towards their (north)westernmost range edge. Our study species shows a disjunct peripheral distribution with common Pannonian (Central Hungary) and more eastern occurrences (Romanian Dobruja) compared to the western edge of their continuous Pannonian range (Eastern Austria) and (north) westernmost isolated exclaves (Western Germany). We analysed 3-5 populations from each of these four study regions by cpDNA sequence (atpl-atpH, rpL16 intron) and AFLP fragment variation. Our AFLP results, based on 323 individuals, showed strong population differentiation, most pronounced within the Hungarian Pannonicum. Moreover, AFLP variation indicated high genetic diversity in the German exclave and at the Pannonian range edge in Eastern Austria compared to less peripheral, more eastern populations. While AFLP results are opposed to theoretical expectations, cpDNA data (i.e. 12 haplotypes) demonstrated lower haplotype diversity towards the (north)westernmost exclave and no monomorphic population within the Romanian Dobruja. Therefore, the latter marker complies with the peripheral transect predictions, although no material from Asia was included. Hence, not only the geographical scale is of importance when analysing phylogeographical patterns, but also possible differences of genetic marker systems. Showing strong signals of recent bottlenecks based on cpDNA in the German exclave, the biogeographical history of A. vernalis somehow differs from other steppe plants investigated to date across the same peripheral transect. </td> <td style="text-align:left;"> NA </td> <td style="text-align:left;"> no </td> <td style="text-align:left;"> No good fitness measure </td> <td style="text-align:left;"> NA </td> <td style="text-align:left;"> no </td> <td style="text-align:left;"> no </td> </tr> <tr> <td style="text-align:left;"> 5225 </td> <td style="text-align:left;"> 2020 </td> <td style="text-align:left;"> 44040 </td> <td style="text-align:left;"> Krstin, L; Katanic, Z; Repar, J; Lezic, M; Kobas, A; Curkovic-Perica, M </td> <td style="text-align:left;"> 2020 </td> <td style="text-align:left;"> Genetic Diversity of Cryphonectria hypovirus 1, a Biocontrol Agent of Chestnut Blight, in Croatia and Slovenia </td> <td style="text-align:left;"> Transmissible hypovirulence associated with Cryphonectria hypovirus 1 (CHV1) has been used for biological control of chestnut blight, devastating disease of chestnut caused by the fungus Cryphonectria parasitica. The main aims of this study were to provide molecular characterization of CHV1 from Croatia and Slovenia and to reveal its genetic variability, phylogeny, and diversification of populations. Fifty-one CHV1 haplotypes were detected among 54 partially sequenced CHV1 isolates, all belonging to Italian subtype (I). Diversity was mainly generated by point mutations while evidence of recombination was not found. The level of conservation over analyzed parts of ORF-A proteins p29 and p40 varied, but functional sites were highly conserved. Phylogenetic analysis revealed close relatedness and intermixing of Croatian and Slovenian CHV1 populations. Our CHV1 isolates were also related to Swiss and Bosnian hypoviruses supporting previously suggested course of CHV1 invasion in Europe. Overall, this study indicates that phylogeny of CHV1 subtype I in Europe is complex and characterized with frequent point mutations resulting in many closely related variants of the virus. Possible association between variations within CHV1 ORF-A and growth of the hypovirulent fungal isolates is tested and presented. </td> <td style="text-align:left;"> NA </td> <td style="text-align:left;"> no </td> <td style="text-align:left;"> No good fitness measure and measure host GD </td> <td style="text-align:left;"> NA </td> <td style="text-align:left;"> no </td> <td style="text-align:left;"> no </td> </tr> <tr> <td style="text-align:left;"> 5226 </td> <td style="text-align:left;"> 2020 </td> <td style="text-align:left;"> 44040 </td> <td style="text-align:left;"> Kruger, A </td> <td style="text-align:left;"> 2020 </td> <td style="text-align:left;"> Functional Redundancy of Batrachochytrium dendrobatidis Inhibition in Bacterial Communities Isolated from Lithobates clamitans Skin </td> <td style="text-align:left;"> The cutaneous microbial community can influence the health of amphibians exposed to Batrachochytrium dendrobatidis (Bd), a fungal pathogen that has contributed to recent amphibian declines. Resistance to Bd in amphibian populations is correlated with the presence of anti-Bd cutaneous microbes, which confer disease resistance by inhibiting Bd growth. I aimed to determine if green frogs (Lithobates clamitans), an abundant and widely distributed species in New Jersey, harbored bacteria that inhibit Bd and whether the presence and identity of these microbes varied among sites. I used in vitro challenge assays to determine if bacteria isolated from green frog skin could inhibit or enhance the growth of Bd. I found that green frogs at all sites harbored anti-Bd bacteria. However, there were differences in Bd inhibition capabilities among bacterial isolates identified as the same operational taxonomic unit (OTU), lending support to the idea that phylogenetic relatedness does not always predict Bd inhibition status. Additionally, anti-Bd bacterial richness did not vary by site, but the composition of anti-Bd bacterial taxa was distinct at each site. This suggests that there is functional redundancy of Bd inhibition across unique communities of anti-Bd symbionts found on frogs at different sites. These findings highlight the need to better elucidate the structure-function relationship of microbiomes and their role in disease resistance. </td> <td style="text-align:left;"> NA </td> <td style="text-align:left;"> no </td> <td style="text-align:left;"> n </td> <td style="text-align:left;"> NA </td> <td style="text-align:left;"> no </td> <td style="text-align:left;"> no </td> </tr> <tr> <td style="text-align:left;"> 5227 </td> <td style="text-align:left;"> 2020 </td> <td style="text-align:left;"> 44113 </td> <td style="text-align:left;"> Krutzen, M; Beasley, I; Ackermann, CY; Lieckfeldt, D; Ludwig, A; Ryan, GE; Bejder, L; Parra, GJ; Wolfensberger, R; Spencer, PBS </td> <td style="text-align:left;"> 2018 </td> <td style="text-align:left;"> Demographic collapse and low genetic diversity of the Irrawaddy dolphin population inhabiting the Mekong River </td> <td style="text-align:left;"> In threatened wildlife populations, it is important to determine whether observed low genetic diversity may be due to recent anthropogenic pressure or the consequence of historic events. Historical size of the Irrawaddy dolphin (Orcaella brevirostris) population inhabiting the Mekong River is unknown and there is significant concern for long-term survival of the remaining population as a result of low abundance, slow reproduction rate, high neonatal mortality, and continuing anthropogenic threats. We investigated population structure and reconstructed the demographic history based on 60 Irrawaddy dolphins samples collected between 2001 and 2009. The phylogenetic analysis indicated reciprocal monophyly of Mekong River Orcaella haplotypes with respect to haplotypes from other populations, suggesting long-standing isolation of the Mekong dolphin population from other Orcaella populations. We found that at least 85% of all individuals in the two main study areas: Kratie and Stung Treng, bore the same mitochondrial haplotype. Out of the 21 microsatellite loci tested, only ten were polymorphic and exhibited very low levels of genetic diversity. Both individual and frequency-based approaches suggest very low and non-significant genetic differentiation of the Mekong dolphin population. Evidence for recent bottlenecks was equivocal. Some results suggested a recent exponential decline in the Mekong dolphin population, with the current size being only 5.2% of the ancestral population. In order for the Mekong dolphin population to have any potential for long-term survival, it is imperative that management priorities focus on preventing any further population fragmentation or genetic loss, reducing or eliminating anthropogenic threats, and promoting connectivity between all subpopulations. </td> <td style="text-align:left;"> NA </td> <td style="text-align:left;"> no </td> <td style="text-align:left;"> no good gd measure host pop </td> <td style="text-align:left;"> NA </td> <td style="text-align:left;"> no </td> <td style="text-align:left;"> no </td> </tr> <tr> <td style="text-align:left;"> 5228 </td> <td style="text-align:left;"> 2020 </td> <td style="text-align:left;"> 44113 </td> <td style="text-align:left;"> Kubiak, K; Dzika, E; Paukszto, L </td> <td style="text-align:left;"> 2017 </td> <td style="text-align:left;"> Enterobiasis epidemiology and molecular characterization of Enterobius vermicularis in healthy children in north-eastern Poland </td> <td style="text-align:left;"> Enterobiasis is a human intestinal parasitic disease caused by human pinworm, Enterobius vermicularis. Despite being the most prevalent nematode infection in Europe and North America, predominantly among in school aged children, the data concerning infection rate and knowledge of genetic variability of pinworms are incomplete. The aim of the study was the estimation of prevalence and molecular typing of Enterobius vermicularis among healthy children in north-eastern Poland. In 2013 - 2015, 296 individuals (aged 2 - 18 years) from 12 kindergartens, schools and orphanages were examined by the adhesive cellophane tape method. Data on socio-demographic status were collected using a questionnaire. Molecular analysis was performed using the DNA of adult female pinworms and primers targeting the region of cytochrome oxidase I gene. The overall prevalence of enterobiasis was 10.1 %. Enterobius vermicularis infection rates were 3.9 % in children living in families and 32.8 % among the orphans (OR= 0.08; 95 % CI: 0.04 - 0.19; p&lt; 0.001). There were no associations between distribution of enterobiasis and gender, pets possession and the season of examination. In 43.3 % of the infected children enterobiasis was asymptomatic. Based on a molecular marker three different haplotypes of pinworm were identified. All sequences clustered within type B, together with human E. vermicularis isolates from Denmark, Germany, Greece, and Japan. This paper provides complementary data on the occurrence and intraspecific variability of E. vermicularis in human population in Europe. </td> <td style="text-align:left;"> NA </td> <td style="text-align:left;"> no </td> <td style="text-align:left;"> human </td> <td style="text-align:left;"> NA </td> <td style="text-align:left;"> no </td> <td style="text-align:left;"> no </td> </tr> <tr> <td style="text-align:left;"> 5229 </td> <td style="text-align:left;"> 2020 </td> <td style="text-align:left;"> 44112 </td> <td style="text-align:left;"> Kumar, C; Song, S; Jiang, L; He, XH; Zhao, QJ; Pu, YB; Malhi, KK; Kamboh, AA; Ma, YH </td> <td style="text-align:left;"> 2018 </td> <td style="text-align:left;"> Sequence Characterization of DSG3 Gene to Know Its Role in High-Altitude Hypoxia Adaptation in the Chinese Cashmere Goat </td> <td style="text-align:left;"> The Tibetan cashmere goat is one of the main goat breeds used by people living in the plateau. It exhibits the distinct phenotypic characteristics observed in lowland goats, allowing them to adapt to the challenging conditions at high altitudes. It provides an ideal model for understanding the genetic mechanisms underlying high-altitude adaptation and hypoxia-related diseases. Our previous exome sequencing of five Chinese cashmere breeds revealed a candidate gene, DSG3 (Desmoglein 3), responsible for the high-altitude adaptation of the Tibetan goat. However, the whole DSG3 gene (44 kbp) consisting of 16 exons in the goat genome was not entirely covered by the exome sequencing. In this study, we resequenced all the 16 exons of the DSG3 gene in ten Chinese native goat populations. Twenty-seven SNP variants were found between the lowland and highland goat populations. The genetic distance (F-ST) of significant SNPs between the lowland and highland populations ranged from 0.42 to 0.58. By using correlation coefficient analysis, linkage disequilibrium, and haplotype network construction, we found three non-synonymous SNPs (R597E, T595I, and G572S) in exon 5 and two synonymous SNPs in exons 8 and 16 in DSG3. These mutations significantly segregated high- and low-altitude goats in two clusters, indicating the contribution of DSG3 to the high-altitude hypoxia adaptation in the Tibetan goat. </td> <td style="text-align:left;"> NA </td> <td style="text-align:left;"> no </td> <td style="text-align:left;"> dometicated host </td> <td style="text-align:left;"> NA </td> <td style="text-align:left;"> no </td> <td style="text-align:left;"> no </td> </tr> <tr> <td style="text-align:left;"> 5230 </td> <td style="text-align:left;"> 2020 </td> <td style="text-align:left;"> 44042 </td> <td style="text-align:left;"> Kumar, S; Saxena, S; Rai, A; Radhakrishna, A; Kaushal, P </td> <td style="text-align:left;"> 2019 </td> <td style="text-align:left;"> Ecological, genetic, and reproductive features of Cenchrus species indicate evolutionary superiority of apomixis under environmental stresses </td> <td style="text-align:left;"> Many Cenchrus species are resilient to harsh environmental conditions and reproduce through apomixis (asexual seed production), and the resulting progeny is genetically identical to the mother plant. Although the apomictic mode of reproduction restricts the efforts of plant breeders to pyramiding desirable traits, it facilitates the fixation of genetic combinations over multiple generations. Eight Cenchrus species are found in India of which only four apomictic species are grown in sown pastures. We assessed the variability in the morphological, behavioral, ecological, reproductive, genetic and molecular features among the different Cenchrus species, and envisaged a relationship between apomixis and stress tolerance for survival and species propagation under unfavorable ecological conditions. Our findings revealed considerable variability among the Cenchrus species. Molecular diversity analysis grouped the Cenchrus species into two distinct clusters. The apomictic species exhibited a lower diversity than the sexual species. Reproductive pathway diversity (B-II and B(III)( )seed formation) among the apomictic Cenchrus species indicated segregation of the components of apomixis, namely parthenogenesis, apomeiosis and functional endosperm formation. For the first time we report partitioning of the components of apomixis in C. glaucus. The earlier reported correlation between apomixis and polyploidy was not observed in the genus Cenchrus. Apomictic Cenchrus species are usually perennial, highly productive and invasive, and adapted to yearly fluctuations in the weather. Sexual Cenchrus species are usually annual, relatively less productive and do not survive during winter. Therefore, we concluded that apomixis facilitates the expansion of species by enabling reproduction even under stressful conditions. The information on genetic and reproductive diversity among the Cenchrus species presented in this study might be useful for interspecific hybridization among these species, as well as to decipher the molecular basis of apomixis in plants. </td> <td style="text-align:left;"> NA </td> <td style="text-align:left;"> no </td> <td style="text-align:left;"> No good fitness measure and measure host GD </td> <td style="text-align:left;"> NA </td> <td style="text-align:left;"> no </td> <td style="text-align:left;"> no </td> </tr> <tr> <td style="text-align:left;"> 5231 </td> <td style="text-align:left;"> 2020 </td> <td style="text-align:left;"> 44113 </td> <td style="text-align:left;"> Kwon, DH; Jeong, IH; Lee, SW; Lee, SH </td> <td style="text-align:left;"> 2018 </td> <td style="text-align:left;"> Comparing the genetic diversity and structure of indigenous Korean and Chinese populations of Laodelphax striatellus Fallen using mitochondrial haplotypes </td> <td style="text-align:left;"> The small brown planthopper, Laodelphax striatellus Fallen, is a major migratory pest from China to Korea and transmits viral diseases of rice plants. In this study, we compared the genetic structures of overwintered indigenous Korean and Chinese populations. The eight Korean populations consisted of 33 haplotypes, and 16 of these were newly identified. The genetic diversity of the Korean population revealed high haplotype diversity (Hd) and low nucleotide diversity (pi) of 0.86 +/- 0.017 and 0.0024 +/- 0.0001 on average, respectively. These values for the Chinese population were Hd = 0.92 +/- 0.016 and n = 0.0026 +/- 0.0002. Overall, genetic distances by pairwise F-ST were generally low, ranging from -0.022 to 0.089. Moreover, the Korean population revealed its ongoing population expansion by showing negative values in the neutrality test (Tajima's D = -1.4, Fu's Fs = -15.8) and a unimodal pattern in the mismatch distribution analysis. The genetic structures and population relationships were determined using 8 indigenous Korean and 30 Chinese populations by Bayesian approaches and population tree construction, respectively. The genetic structure was inferred as five (K = 5), and all indigenous Korean populations as well as 10 Chinese populations from the Shanghai city, Anhui, Henan, Jiangsu, Liaoning, Shandong, and Zhejiang provinces were grouped into 'G3'. These results provide partial support for unidirectional gene flow by migratory Chinese populations into Korea. Further analysis conducted using specimens from China and Korea collected in similar periods is necessary to clarify the migration population's origin and its effect on the genetic structure of the indigenous populations. </td> <td style="text-align:left;"> NA </td> <td style="text-align:left;"> no </td> <td style="text-align:left;"> No good fitness measure and measure host GD </td> <td style="text-align:left;"> NA </td> <td style="text-align:left;"> no </td> <td style="text-align:left;"> no </td> </tr> <tr> <td style="text-align:left;"> 5232 </td> <td style="text-align:left;"> 2020 </td> <td style="text-align:left;"> 44111 </td> <td style="text-align:left;"> Lacey, EA; Takenaka, R; LaBarbera, K; Tammone, MN </td> <td style="text-align:left;"> 2019 </td> <td style="text-align:left;"> Ecological and demographic impacts of a recent volcanic eruption on two endemic patagonian rodents </td> <td style="text-align:left;"> Catastrophic events can significantly impact the demographic processes that shape natural populations of organisms. However, linking the outcomes of such events to specific demographic parameters is often challenging due to a lack of detailed pre-event data. The eruption of the Puyehue-Cordon Caulle volcanic complex on 4 June 2011 had profound consequences for the biota of southwestern Argentina. Our long-term behavioral, ecological, and demographic studies of two species of tuco-tucos (Ctenomys sociabilis and C. help) that occur in the region most heavily impacted by ash fall from the eruption provided an unusual opportunity to assess the effects of this event on natural populations of mammals. The post-eruption density of the study population for each species was markedly reduced compared to pre-eruption values, with the relative magnitude of this reduction being greater for the group-living C. sociabilis. The more extensive data set for this species indicated that ash fall from the eruption altered the food resources available to these animals; differences in pre- and post-eruption stable isotope signatures for fur samples from C. sociabilis were consistent with observed changes in vegetation. Per capita female reproductive success was also reduced in this species during the first breeding season following the eruption. Based on our detailed demographic records for C. sociabilis, neither survival of yearling females from 2010 to 2011 nor the percentage of unmarked females in the study population in 2011 differed from pre-eruption values. Instead, the post-eruption decrease in population density for C. sociabilis appeared to reflect reduced within-population recruitment of juvenile females to the 2011 breeding population. Although the eruption did not result in the local extinction of either study population, the demographic consequences detected are likely to have impacted the effective sizes of these populations, creating important opportunities to link specific demographic parameters to previously reported decreases in genetic variability detected after this significant natural event. </td> <td style="text-align:left;"> NA </td> <td style="text-align:left;"> no </td> <td style="text-align:left;"> no good gd measure host pop </td> <td style="text-align:left;"> NA </td> <td style="text-align:left;"> no </td> <td style="text-align:left;"> no </td> </tr> <tr> <td style="text-align:left;"> 5233 </td> <td style="text-align:left;"> 2020 </td> <td style="text-align:left;"> 44111 </td> <td style="text-align:left;"> Laine, AL; Barres, B; Numminen, E; Siren, JP </td> <td style="text-align:left;"> 2019 </td> <td style="text-align:left;"> Variable opportunities for outcrossing result in hotspots of novel genetic variation in a pathogen metapopulation </td> <td style="text-align:left;"> Many pathogens possess the capacity for sex through outcrossing, despite being able to reproduce also asexually and/or via selfing. Given that sex is assumed to come at a cost, these mixed reproductive strategies typical of pathogens have remained puzzling. While the ecological and evolutionary benefits of outcrossing are theoretically well-supported, support for such benefits in pathogen populations are still scarce. Here, we analyze the epidemiology and genetic structure of natural populations of an obligate fungal pathogen, Podosphaera plantaginis. We find that the opportunities for outcrossing vary spatially. Populations supporting high levels of coinfection -a prerequisite of sex - result in hotspots of novel genetic diversity. Pathogen populations supporting coinfection also have a higher probability of surviving winter. Jointly our results show that outcrossing has direct epidemiological consequences as well as a major impact on pathogen population genetic diversity, thereby providing evidence of ecological and evolutionary benefits of outcrossing in pathogens. </td> <td style="text-align:left;"> NA </td> <td style="text-align:left;"> no </td> <td style="text-align:left;"> gd of pathogen </td> <td style="text-align:left;"> NA </td> <td style="text-align:left;"> no </td> <td style="text-align:left;"> no </td> </tr> <tr> <td style="text-align:left;"> 5234 </td> <td style="text-align:left;"> 2020 </td> <td style="text-align:left;"> 44112 </td> <td style="text-align:left;"> Lam, MMC; Wick, RR; Wyres, KL; Gorrie, CL; Judd, LM; Jenney, AWJ; Brisse, S; Holt, KE </td> <td style="text-align:left;"> 2018 </td> <td style="text-align:left;"> Genetic diversity, mobilisation and spread of the yersiniabactin-encoding mobile element ICEKp in Klebsiella pneumoniae populations </td> <td style="text-align:left;"> Mobile genetic elements (MGEs) that frequently transfer within and between bacterial species play a critical role in bacterial evolution, and often carry key accessory genes that associate with a bacteria's ability to cause disease. MGEs carrying antimicrobial resistance (AMR) and/or virulence determinants are common in the opportunistic pathogen Klebsiella pneumoniae, which is a leading cause of highly drug-resistant infections in hospitals. Well-characterised virulence determinants in K. pneumoniae include the polyketide synthesis loci ybt and clb (also known as pks), encoding the iron-scavenging siderophore yersiniabactin and genotoxin colibactin, respectively. These loci are located within an MGE called ICEKp, which is the most common virulence-associated MGE of K. pneumoniae, providing a mechanism for these virulence factors to spread within the population. Here we apply population genomics to investigate the prevalence, evolution and mobility of ybt and clb in K. pneumoniae populations through comparative analysis of 2498 whole-genome sequences. The ybt locus was detected in 40% of K. pneumoniae genomes, particularly amongst those associated with invasive infections. We identified 17 distinct ybt lineages and 3 clb lineages, each associated with one of 14 different structural variants of ICEKp. Comparison with the wider population of the family Enterobacteriaceae revealed occasional ICEKp acquisition by other members. The clb locus was present in 14% of all K. pneumoniae and 38.4% of ybt+ genomes. Hundreds of independent ICEKp integration events were detected affecting hundreds of phylogenetically distinct K. pneumoniae lineages, including at least 19 in the globally-disseminated carbapenem-resistant clone CG258. A novel plasmid-encoded form of ybt was also identified, representing a new mechanism for ybt dispersal in K. pneumoniae populations. These data indicate that MGEs carrying ybt and clb circulate freely in the K. pneumoniae population, including among multidrug-resistant strains, and should be considered a target for genomic surveillance along with AMR determinants. </td> <td style="text-align:left;"> NA </td> <td style="text-align:left;"> no </td> <td style="text-align:left;"> No good fitness measure and measure host GD </td> <td style="text-align:left;"> NA </td> <td style="text-align:left;"> no </td> <td style="text-align:left;"> no </td> </tr> <tr> <td style="text-align:left;"> 5235 </td> <td style="text-align:left;"> 2020 </td> <td style="text-align:left;"> 44112 </td> <td style="text-align:left;"> Lam, MMC; Wyres, KL; Judd, LM; Wick, RR; Jenney, A; Brisse, S; Holt, KE </td> <td style="text-align:left;"> 2018 </td> <td style="text-align:left;"> Tracking key virulence loci encoding aerobactin and salmochelin siderophore synthesis in Klebsiella pneumoniae </td> <td style="text-align:left;"> BackgroundKlebsiella pneumoniae is a recognised agent of multidrug-resistant (MDR) healthcare-associated infections; however, individual strains vary in their virulence potential due to the presence of mobile accessory genes. In particular, gene clusters encoding the biosynthesis of siderophores aerobactin (iuc) and salmochelin (iro) are associated with invasive disease and are common amongst hypervirulent K. pneumoniae clones that cause severe community-associated infections such as liver abscess and pneumonia. Concerningly, iuc has also been reported in MDR strains in the hospital setting, where it was associated with increased mortality, highlighting the need to understand, detect and track the mobility of these virulence loci in the K. pneumoniae population.MethodsHere, we examined the genetic diversity, distribution and mobilisation of iuc and iro loci amongst 2503 K. pneumoniae genomes using comparative genomics approaches and developed tools for tracking them via genomic surveillance.ResultsIro and iuc were detected at low prevalence (&lt;10%). Considerable genetic diversity was observed, resolving into five iro and six iuc lineages that show distinct patterns of mobilisation and dissemination in the K. pneumoniae population. The major burden of iuc and iro amongst the genomes analysed was due to two linked lineages (iuc1/iro1 74% and iuc2/iro2 14%), each carried by a distinct non-self-transmissible IncFIB(K) virulence plasmid type that we designate KpVP-1 and KpVP-2. These dominant types also carry hypermucoidy (rmpA) determinants and include all previously described virulence plasmids of K. pneumoniae. The other iuc and iro lineages were associated with diverse plasmids, including some carrying IncFII conjugative transfer regions and some imported from Escherichia coli; the exceptions were iro3 (mobilised by ICEKp1) and iuc4 (fixed in the chromosome of K. pneumoniae subspecies rhinoscleromatis). Iro/iuc mobile genetic elements (MGEs) appear to be stably maintained at high frequency within known hypervirulent strains (ST23, ST86, etc.) but were also detected at low prevalence in others such as MDR strain ST258.ConclusionsIuc and iro are mobilised in K. pneumoniae via a limited number of MGEs. This study provides a framework for identifying and tracking these important virulence loci, which will be important for genomic surveillance efforts including monitoring for the emergence of hypervirulent MDR K. pneumoniae strains. </td> <td style="text-align:left;"> NA </td> <td style="text-align:left;"> no </td> <td style="text-align:left;"> No good fitness measure and measure host GD </td> <td style="text-align:left;"> NA </td> <td style="text-align:left;"> no </td> <td style="text-align:left;"> no </td> </tr> <tr> <td style="text-align:left;"> 5236 </td> <td style="text-align:left;"> 2020 </td> <td style="text-align:left;"> 44040 </td> <td style="text-align:left;"> Lapegue, S; Heurtebise, S; Cornette, F; Guichoux, E; Gagnaire, PA </td> <td style="text-align:left;"> 2020 </td> <td style="text-align:left;"> Genetic Characterization of Cupped Oyster Resources in Europe Using Informative Single Nucleotide Polymorphism (SNP) Panels </td> <td style="text-align:left;"> The Pacific oyster, Crassostrea gigas, was voluntarily introduced from Japan and British Columbia into Europe in the early 1970s, mainly to replace the Portuguese oyster, Crassostrea angulata, in the French shellfish industry, following a severe disease outbreak. Since then, the two species have been in contact in southern Europe and, therefore, have the potential to exchange genes. Recent evolutionary genomic works have provided empirical evidence that C. gigas and C. angulata exhibit partial reproductive isolation. Although hybridization occurs in nature, the rate of interspecific gene flow varies across the genome, resulting in highly heterogeneous genome divergence. Taking this biological property into account is important to characterize genetic ancestry and population structure in oysters. Here, we identified a subset of ancestry-informative makers from the most differentiated regions of the genome using existing genomic resources. We developed two different panels in order to (i) easily differentiate C. gigas and C. angulata, and (ii) describe the genetic diversity and structure of the cupped oyster with a particular focus on French Atlantic populations. Our results confirm high genetic homogeneity among Pacific cupped oyster populations in France and reveal several cases of introgressions between Portuguese and Japanese oysters in France and Portugal. </td> <td style="text-align:left;"> NA </td> <td style="text-align:left;"> no </td> <td style="text-align:left;"> No good fitness measure and measure host GD </td> <td style="text-align:left;"> NA </td> <td style="text-align:left;"> no </td> <td style="text-align:left;"> no </td> </tr> <tr> <td style="text-align:left;"> 5237 </td> <td style="text-align:left;"> 2020 </td> <td style="text-align:left;"> 44113 </td> <td style="text-align:left;"> Larrucea, ES; Robinson, ML; Rippert, JS; Matocq, MD </td> <td style="text-align:left;"> 2018 </td> <td style="text-align:left;"> Genetically distinct populations of the pygmy rabbit (Brachylagus idahoensis) in the Mono Basin of California </td> <td style="text-align:left;"> We examined patterns of genetic variation and diversity of extant pygmy rabbit (Brachylagus idahoensis) populations across the species' current range in Nevada and California. Our aims were to determine population genetic structure and levels of diversity across the southern portion of the species' range. We genotyped 13 microsatellite loci from 194 fecal samples collected across 14 localities. Our Bayesian cluster analyses found 2 genetically distinct groups: 1 in the Mono Basin of California and the other encompassing all remaining Nevada Great Basin populations. Considering only the Nevada Great Basin group, we found 4 minimally divergent groups that overlap spatially with many individuals maintaining composite genomes with representation from multiple genetic groups. On average, the Mono Basin populations maintain lower levels of genetic diversity than the Nevada Great Basin populations as measured by both heterozygosity and numbers of alleles. The Mono Basin contains the only populations of pygmy rabbits remaining in California. Given their geographic isolation from other known populations and their genetic distinction, these remnant, peripheral populations warrant special attention to ensure maintenance of genetic variation and continued viability. </td> <td style="text-align:left;"> NA </td> <td style="text-align:left;"> no </td> <td style="text-align:left;"> No good fitness measure </td> <td style="text-align:left;"> NA </td> <td style="text-align:left;"> no </td> <td style="text-align:left;"> no </td> </tr> <tr> <td style="text-align:left;"> 5238 </td> <td style="text-align:left;"> 2020 </td> <td style="text-align:left;"> 44112 </td> <td style="text-align:left;"> Larsson, V; Torisson, G; Londos, E </td> <td style="text-align:left;"> 2018 </td> <td style="text-align:left;"> Connections and containers: Using genetic data to understand how watershed evolution and human activities influence cutthroat trout biogeography </td> <td style="text-align:left;"> Species with large geographic distributions often exhibit complex patterns of diversity that can be further complicated by human activities. Cutthroat trout (Oncorhynchus clarkii) are one of the most widely distributed freshwater fish species in western North America exhibiting substantial phenotypic and genetic variability; however, fish stocking practices have translocated populations outside of their native range and may have obscured intraspecific boundaries. This study focuses on cutthroat trout populations representing three distinct evolutionary clades that are found intermixed within a contact zone between the Bonneville and upper Snake River watersheds in the western United States. We used mitochondrial and microsatellite genetic data, as well as historical stocking records, to evaluate whether populations of cutthroat trout in the contact zone are native or are introduced. We found significant genetic differentiation and fine-scale genetic population structure that was organized primarily by watershed boundaries. While we detected increased genetic diversity in some areas in close proximity to the greatest number of stocking events, the highly organized population structure both within and between areas of the contact zone indicates that the populations are native to the watersheds. Intermixing of distinct evolutionary lineages of cutthroat trout appears to be the result of historical connections between paleodrainages. Our analyses provide a context for understanding how genetic data can be used to assess the status of populations as native or introduced. </td> <td style="text-align:left;"> NA </td> <td style="text-align:left;"> no </td> <td style="text-align:left;"> No good fitness measure and measure host GD </td> <td style="text-align:left;"> NA </td> <td style="text-align:left;"> no </td> <td style="text-align:left;"> no </td> </tr> <tr> <td style="text-align:left;"> 5239 </td> <td style="text-align:left;"> 2020 </td> <td style="text-align:left;"> 44113 </td> <td style="text-align:left;"> Lasserre, M; Fresia, P; Greif, G; Iraola, G; Castro-Ramos, M; Juambeltz, A; Nunez, A; Naya, H; Robello, C; Berna, L </td> <td style="text-align:left;"> 2018 </td> <td style="text-align:left;"> Whole genome sequencing of the monomorphic pathogen Mycobacterium bovis reveals local differentiation of cattle clinical isolates </td> <td style="text-align:left;"> Background: Bovine tuberculosis (bTB) poses serious risks to animal welfare and economy, as well as to public health as a zoonosis. Its etiological agent, Mycobacterium bovis, belongs to the Mycobacterium tuberculosis complex (MTBC), a group of genetically monomorphic organisms featured by a remarkably high overall nucleotide identity (99.9%). Indeed, this characteristic is of major concern for correct typing and determination of strain-specific traits based on sequence diversity. Due to its historical economic dependence on cattle production, Uruguay is deeply affected by the prevailing incidence of Mycobacterium bovis. With the world's highest number of cattle per human, and its intensive cattle production, Uruguay represents a particularly suited setting to evaluate genomic variability among isolates, and the diversity traits associated to this pathogen. Results: We compared 186 genomes from MTBC strains isolated worldwide, and found a highly structured population in M. bovis. The analysis of 23 new M. bovis genomes, belonging to strains isolated in Uruguay evidenced three groups present in the country. Despite presenting an expected highly conserved genomic structure and sequence, these strains segregate into a clustered manner within the worldwide phylogeny. Analysis of the non-pe/ppe differential areas against a reference genome defined four main sources of variability, namely: regions of difference (RD), variable genes, duplications and novel genes. RDs and variant analysis segregated the strains into clusters that are concordant with their spoligotype identities. Due to its high homoplasy rate, spoligotyping failed to reflect the true genomic diversity among worldwide representative strains, however, it remains a good indicator for closely related populations. Conclusions: This study introduces a comprehensive population structure analysis of worldwide M. bovis isolates. The incorporation and analysis of 23 novel Uruguayan M. bovis genomes, sheds light onto the genomic diversity of this pathogen, evidencing the existence of greater genetic variability among strains than previously contemplated. </td> <td style="text-align:left;"> NA </td> <td style="text-align:left;"> no </td> <td style="text-align:left;"> gd of pathogen </td> <td style="text-align:left;"> NA </td> <td style="text-align:left;"> no </td> <td style="text-align:left;"> no </td> </tr> <tr> <td style="text-align:left;"> 5240 </td> <td style="text-align:left;"> 2020 </td> <td style="text-align:left;"> 44113 </td> <td style="text-align:left;"> Lau, Q; Igawa, T; Kosch, TA; Satta, Y </td> <td style="text-align:left;"> 2018 </td> <td style="text-align:left;"> Selective constraint acting on TLR2 and TLR4 genes of Japanese Rana frogs </td> <td style="text-align:left;"> Toll-like receptors (TLRs) are an important component of innate immunity, the first line of pathogen defence. One of the major roles of TLRs includes recognition of pathogen-associated molecular patterns. Amphibians are currently'facing population declines and even lextinction due to chytridiomycosis caused by the Batrachochytrium dendrobatidis (Bd) fungus. Evidence from other vertebrates shows that TLR2 and TLR4 are involved in innate immunity against various fungi. such genes therefore may play a functional role in amphibian-chytridiomycosis dynamics. Frogs from East Asia appear to be tolerant to Bd, so we examined the genetic diversity that underlies TLR2 and TLR4 from three Japanese Ranidae frog species, Rana japonica, R, ornattventris and R. tagot tagoi (n = 5 per species). We isolated 27 TLR2 and 20 TLR4 alleles and found that these genes are evolutionarily conserved, with overall evidence supporting purifying selection. In contrast, site-by-site analysis of selection identified several specific codon sites under positive selection, some of which were located in the variable leucineIrish' repeat domains. In addition, preliminary expiression levels of TLR2 and TLR4 from transcriptorne data showed overall low expression. Although It remains unclear whether infectious pathogens area selective force acting on TLRs of Japanese frogs,our results support that certain sites in TLRs of these species may have experienced pathogenmediated selection. </td> <td style="text-align:left;"> NA </td> <td style="text-align:left;"> no </td> <td style="text-align:left;"> No good fitness measure </td> <td style="text-align:left;"> NA </td> <td style="text-align:left;"> no </td> <td style="text-align:left;"> no </td> </tr> <tr> <td style="text-align:left;"> 5241 </td> <td style="text-align:left;"> 2020 </td> <td style="text-align:left;"> 44041 </td> <td style="text-align:left;"> Lauer, S; Gresham, D </td> <td style="text-align:left;"> 2019 </td> <td style="text-align:left;"> An evolving view of copy number variants </td> <td style="text-align:left;"> Copy number variants (CNVs) are regions of the genome that vary in integer copy number. CNVs, which comprise both amplifications and deletions of DNA sequence, have been identified across all domains of life, from bacteria and archaea to plants and animals. CNVs are an important source of genetic diversity, and can drive rapid adaptive evolution and progression of heritable and somatic human diseases, such as cancer. However, despite their evolutionary importance and clinical relevance, CNVs remain understudied compared to single-nucleotide variants (SNVs). This is a consequence of the inherent difficulties in detecting CNVs at low-to-intermediate frequencies in heterogeneous populations of cells. Here, we discuss molecular methods used to detect CNVs, the limitations associated with using these techniques, and the application of new and emerging technologies that present solutions to these challenges. The goal of this short review and perspective is to highlight aspects of CNV biology that are understudied and define avenues for further research that address specific gaps in our knowledge of these complex alleles. We describe our recently developed method for CNV detection in which a fluorescent gene functions as a single-cell CNV reporter and present key findings from our evolution experiments in Saccharomyces cerevisiae. Using a CNV reporter, we found that CNVs are generated at a high rate and undergo selection with predictable dynamics across independently evolving replicate populations. Many CNVs appear to be generated through DNA replication-based processes that are mediated by the presence of short, interrupted, inverted-repeat sequences. Our results have important implications for the role of CNVs in evolutionary processes and the molecular mechanisms that underlie CNV formation. We discuss the possible extension of our method to other applications, including tracking the dynamics of CNVs in models of human tumors. </td> <td style="text-align:left;"> NA </td> <td style="text-align:left;"> no </td> <td style="text-align:left;"> No good fitness measure and measure host GD </td> <td style="text-align:left;"> NA </td> <td style="text-align:left;"> no </td> <td style="text-align:left;"> no </td> </tr> <tr> <td style="text-align:left;"> 5242 </td> <td style="text-align:left;"> 2020 </td> <td style="text-align:left;"> 44041 </td> <td style="text-align:left;"> Le Pendu, J; Ruvoen-Clouet, N </td> <td style="text-align:left;"> 2020 </td> <td style="text-align:left;"> Fondness for sugars of enteric viruses confronts them with human glycans genetic diversity </td> <td style="text-align:left;"> Together, norovirus and rotavirus are responsible for the majority of gastroenteritis cases worldwide, leading to a large number of deaths of children in low-income countries. Both attach to glycans of the histo-blood group antigen type (HBGAs) widely expressed in the digestive tract of vertebrates, albeit with interspecies differences. In humans, their synthesis is performed by glycosyltransferases encoded by the highly polymorphic ABO, FUT2 and FUT3 genes that are under long-term balanced selection. The combination of functional and null or weak alleles at these loci provides a diversity of glycan structures that define the ABO, Secretor and Lewis phenotypes. At the initial stage of infection norovirus and rotavirus attach to these glycans, although distinct strains of each virus present different specificities for individual glycans, hence exhibiting preferences for different human phenotypes. Absence or low expression of the recognized glycan motifs due to genetic polymorphism is associated with resistance to the disease, showing that the HBGA polymorphisms provide a population-based innate protection. Epidemiologically dominant strains of either norovirus or rotavirus display specificity for glycan motifs present in large fractions of the population, which may differ between geographical areas in accordance with the frequency of the ABO, FUT2, FUT3 gene polymorphisms. Evidence for virus adaptation to these geographical differences is amounting, indicative of a host-pathogen co-evolution and suggesting that enteric pathogens such as norovirus and rotavirus are likely the driving forces behind the balanced HBGA polymorphisms. </td> <td style="text-align:left;"> NA </td> <td style="text-align:left;"> no </td> <td style="text-align:left;"> human </td> <td style="text-align:left;"> NA </td> <td style="text-align:left;"> no </td> <td style="text-align:left;"> no </td> </tr> <tr> <td style="text-align:left;"> 5243 </td> <td style="text-align:left;"> 2020 </td> <td style="text-align:left;"> 44112 </td> <td style="text-align:left;"> le Roex, N; Paxton, M; Adendorff, J; Ferreira, S; O'Riain, MJ </td> <td style="text-align:left;"> 2018 </td> <td style="text-align:left;"> Starting small: long-term consequences in a managed large-mammal population </td> <td style="text-align:left;"> Small populations are vulnerable to founder effects, stochastic demographic events and inbreeding depression. These factors raise the extinction probability of small populations, and thus effective management plans for endangered species have become essential. The black rhinoceros (Diceros bicornis) survived a historic global population crash and is currently facing renewed threat through poaching, population fragmentation and isolation. South Africa is home to approximately 36% of the world's remaining black rhinos; however, this population is fragmented across parks and reserves, and many of these small populations remain geographically and genetically isolated. The historic population crash, subsequent fragmentation and relentless poaching pressure together threaten the viability of the black rhino population within South Africa. This study employed a practical, non-invasive sampling protocol and genetic analyses to assess the growth and performance of a small founder population in a fenced reserve. We used these data and recorded life-history data to compare the population growth and vital rates between the first and second 10-year periods since founding. We report a decrease in population growth rate and male survival in the second 10-year period (0.06; 0.89) compared to the first (0.09; 0.97). Genetic diversity (H-EXP = 0.405) was low when compared to larger black rhino populations, and average pairwise relatedness was high (0.193). These results suggest that negative genetic consequences and the beginning of density-dependant growth regulation may be present in this population. This highlights the importance of establishing an evaluation framework for the assessment of small populations of long-lived mammals in order to inform metapopulation management. </td> <td style="text-align:left;"> NA </td> <td style="text-align:left;"> no </td> <td style="text-align:left;"> No good fitness measure and measure host GD </td> <td style="text-align:left;"> NA </td> <td style="text-align:left;"> no </td> <td style="text-align:left;"> no </td> </tr> <tr> <td style="text-align:left;"> 5244 </td> <td style="text-align:left;"> 2020 </td> <td style="text-align:left;"> 44113 </td> <td style="text-align:left;"> Leclerc, JB; Detrain, C </td> <td style="text-align:left;"> 2018 </td> <td style="text-align:left;"> Impact of colony size on survival and sanitary strategies in fungus-infected ant colonies </td> <td style="text-align:left;"> Group-living species have to deal with higher risks of exposure to pathogens and of disease propagation between group members. Insect societies have therefore evolved behaviours that contribute to the social immunity of the whole colony. Here, we investigate how the sanitary strategies displayed by ants depend on colony size, which is known to influence their social organisation. Myrmica rubra ant colonies of different sizes were faced with waste items that were infected or not by the entomopathogen fungus Metarhizium brunneum. By keeping nest parameters and pathogen loads proportional to colony size, we show that the largest colonies less suffered from exposure to life-threatening spores. Indeed, the largest colonies showed the lowest mortality rates and were the fastest to reject all the infected waste items out from their nest. Unexpectedly, small-sized colonies displayed distinct sanitary strategies according to the pathogenicity of the waste items. When challenged with fungus-bearing items, they opted out for an emergency strategy in which workers first moved out from their nest carrying brood and reintegrate it after sanitization by waste-transporting individuals. This demonstrates the behavioural plasticity of ant colonies of which both the size and the sanitary threat determine the efficiency and the type of hygienic responses to hygienic challenges. Significance statement Insect societies have evolved several collective hygienic behaviours that contribute to their social immunity. Here, we found out that both internal and external factors determine the efficiency and the type of hygienic responses to waste items introduced in an ant colony. In particular, small-sized colonies demonstrated a behavioural plasticity with distinct sanitary strategies according to the pathogenicity of the waste items. Such a plasticity appears to be an adaptive way to limit exposure of larvae to pathogens and hence to preserve colony development. It also raises some questions about the origin of the differences in worker's internal threshold allowing colonies to shift between sanitary strategies. </td> <td style="text-align:left;"> NA </td> <td style="text-align:left;"> no </td> <td style="text-align:left;"> no good gd measure host pop </td> <td style="text-align:left;"> NA </td> <td style="text-align:left;"> no </td> <td style="text-align:left;"> no </td> </tr> <tr> <td style="text-align:left;"> 5245 </td> <td style="text-align:left;"> 2020 </td> <td style="text-align:left;"> 44112 </td> <td style="text-align:left;"> Lecocq, T; Michez, D; Gerard, M; Vereecken, NJ; Delangre, J; Rasmont, P; Vray, S; Dufrene, M; Mardulyn, P; Dellicour, S </td> <td style="text-align:left;"> 2018 </td> <td style="text-align:left;"> Divergent geographic patterns of genetic diversity among wild bees: Conservation implications </td> <td style="text-align:left;"> Aim Genetic diversity is a key factor to species survival. This diversity is unevenly distributed across the species range, delimiting genetic diversity hotspots (GDH). Focusing conservation efforts on regions where GDH of several species overlap (i.e., multispecies GDH) could rationalize conservation efforts by protecting several taxa in one go. However, recent studies suggest the existence of many species-specific GDH. This would make spatially prioritizing protection even more challenging as it requires the integration of these multiple GDH rather than few hotspots into conservation planning. Here, we characterize GDH of nine co-distributed bee species through an original comparative mapping approach to assess the suitability of a spatial prioritization strategy to protect their genetic diversity. Location Methods We studied bee populations from Europe. First, we used a sliding window approach to estimate the nucleotide diversity and its geographic distribution to highlight GDH of each species. Second, we assessed the overlap of GDH between species by generating consensus maps based on the species-specific maps of nucleotide diversity. Third, we used the GDH distribution patterns to identify the extent of cost-effective area network that would be needed to protect genetic diversity of all nine species. Results Main conclusions Genetic diversity was unevenly distributed across species ranges, but we found no evidence of a large overlap among GDH from all species. Cost-effective area network needed to protect genetic diversity of all species spreads over several large geographic areas including regions under high human development pressures. Genetic diversity hotspots' location is species-specific. Therefore, focusing conservation efforts strictly on the few regions harbouring GDH for many bee species is unlikely to be sufficient to ensure long-term persistence of all species. Conservation actions should be implemented simultaneously in different regions according to a complementary-based conservation approach, to optimize the conservation of all bee diversity. </td> <td style="text-align:left;"> NA </td> <td style="text-align:left;"> no </td> <td style="text-align:left;"> No good fitness measure </td> <td style="text-align:left;"> NA </td> <td style="text-align:left;"> no </td> <td style="text-align:left;"> no </td> </tr> <tr> <td style="text-align:left;"> 5246 </td> <td style="text-align:left;"> 2020 </td> <td style="text-align:left;"> 44040 </td> <td style="text-align:left;"> Lee, DE; Fienieg, E; Van Oosterhout, C; Muller, Z; Strauss, M; Carter, KD; Scheijen, CPJ; Deacon, F </td> <td style="text-align:left;"> 2020 </td> <td style="text-align:left;"> Giraffe translocation population viability analysis </td> <td style="text-align:left;"> Most populations of giraffes have declined in recent decades, leading to the recent IUCN decision to upgrade the species to Vulnerable status, and some subspecies to Endangered. Translocations have been used as a conservation tool to re-introduce giraffes to previously occupied areas or establish new populations, but guidelines for founding populations are lacking. To provide general guidelines for translocation projects regarding feasibility, we simulated various scenarios of translocated giraffe populations to identify viable age and sex distributions of founding populations using population viability analysis (PVA) implemented in Vortex software. We explored the parameter space for demography and the genetic load, examining how variation in founding numbers and sex ratios affected 100 yr probability of population extinction and genetic diversity. We found that even very small numbers of founders (N &lt;= 10 females) can appear to be successful in the first decades due to transient positive population growth, but with moderate population growth rate and moderate genetic load, long-term population viability (probability of extinction &lt;0.01) was only achieved with &gt;= 30 females and &gt;= 3 males released. To maintain &gt;95% genetic diversity of the source population in an isolated population, 50 females and 5 males are recommended to compose the founding population. Sensitivity analyses revealed first-year survival and reproductive rate were the simulation parameters with the greatest proportional influence on probability of extinction and genetic diversity. These simulations highlight important considerations for translocation success and data gaps including true genetic load in wild giraffe populations. </td> <td style="text-align:left;"> NA </td> <td style="text-align:left;"> no </td> <td style="text-align:left;"> n </td> <td style="text-align:left;"> NA </td> <td style="text-align:left;"> no </td> <td style="text-align:left;"> no </td> </tr> <tr> <td style="text-align:left;"> 5247 </td> <td style="text-align:left;"> 2020 </td> <td style="text-align:left;"> 44113 </td> <td style="text-align:left;"> Lee, JY; Moon, S; Kim, YK; Lee, SH; Lee, BS; Park, MY; Park, JE; Jang, Y; Han, BG </td> <td style="text-align:left;"> 2017 </td> <td style="text-align:left;"> Genome-based exome sequencing analysis identifies GYG1, DIS3L and DDRGK1 are associated with myocardial infarction in Koreans </td> <td style="text-align:left;"> Myocardial infarction (MI) is a complex disease caused by combination of genetic and environmental factors. Although genome-wide association studies (GWAS) identified more than 46 risk loci which are associated with coronary artery disease and MI, most of the genetic variability in MI still remains undefined. Here, we screened the susceptibility loci for MI using exome sequencing and validated candidate variants in replication sets. We identified that three genes (GYG1, DIS3L and DDRGK1) were associated with MI at the discovery and replication stages. Further research will be required to determine the functional association of these genes with MI risk, and these associations have to be confirmed in other ethnic populations. </td> <td style="text-align:left;"> NA </td> <td style="text-align:left;"> no </td> <td style="text-align:left;"> human </td> <td style="text-align:left;"> NA </td> <td style="text-align:left;"> no </td> <td style="text-align:left;"> no </td> </tr> <tr> <td style="text-align:left;"> 5248 </td> <td style="text-align:left;"> 2020 </td> <td style="text-align:left;"> 44112 </td> <td style="text-align:left;"> Lee, KJ; Raveendar, S; Choi, JS; Gil, J; Lee, JH; So, YS; Chung, JW </td> <td style="text-align:left;"> 2019 </td> <td style="text-align:left;"> Development of chloroplast microsatellite markers for identification of Glycyrrhiza species </td> <td style="text-align:left;"> Licorice (Glycyrrhiza glabra) is an important medicinal herb and has long been used in traditional medicine for the treatment of several diseases worldwide. Understanding the genetic diversity within Glycyrrhiza species is important for the efficient conservation of these medicinal herbs. In this study, we have developed 20 polymorphic chloroplast microsatellite (cpSSR) markers using the chloroplast genome of G. lepidota. The cpSSR markers were tested on a total of 27 Glycyrrhiza individual plants. The number of alleles per locus ranged from two to eight among the Glycyrrhiza accessions. Overall, the Shannon index (I) for each cpSSR ranged from 0.315 to 1.694, the diversity indices (h) were 0.140-0.793 and the unbiased diversity indices (uh) were 0.145-0.825. In addition, the cpSSR markers were successfully divided and classified the 27 Glycyrrhiza individuals into four groups. The cpSSR markers developed in this study could be used in the assessment of genetic diversity and rapid identification of Glycyrrhiza species. </td> <td style="text-align:left;"> NA </td> <td style="text-align:left;"> no </td> <td style="text-align:left;"> No good fitness measure and measure host GD </td> <td style="text-align:left;"> NA </td> <td style="text-align:left;"> no </td> <td style="text-align:left;"> no </td> </tr> <tr> <td style="text-align:left;"> 5249 </td> <td style="text-align:left;"> 2020 </td> <td style="text-align:left;"> 44112 </td> <td style="text-align:left;"> Lee, L; Tirrell, N; Burrell, C; Chambers, S; Vogel, S; Domyan, ET </td> <td style="text-align:left;"> 2018 </td> <td style="text-align:left;"> Genetic tests reveal extra-pair paternity among Gentoo penguins (Pyogoscelis papua ellsworthii) at Loveland Living Planet Aquarium: Implications for ex situ colony management </td> <td style="text-align:left;"> Maintenance of ex situ populations for species conservation is a collaborative effort involving multiple agencies, institutions, and individuals around the world. Gentoo penguins (Pyogoscelis papua) are one species involved in such a conservation effort, and a Species Survival Plan (SSP) has been put in place by the Association of Zoos and Aquariums (AZA) to foster their long-term sustainability. As a part of their SSP, a Breeding and Transfer Plan has been created to support interagency exchanges of specimens. These transfers are vital to the demographic health and stability of ex situ populations, as well as the maintenance of genetic diversity. In populations such as the Gentoo, where social monogamy exists, paternal inferences of offspring are usually made through observation of birds' social behavior. However, social monogamy does not guarantee reproductive monogamy. In this study, we utilize Illumina high-throughput DNA sequencing to genetically test the postulated paternity of Gentoo penguins born at Loveland Living Planet Aquarium (LLPA) in Draper, UT. While our data support the majority of the postulated relationships, we did identify two offspring that were the result of extra-pair paternity (EPP). The results of this research highlight the importance of genetic tests to validate pedigrees used in SSPs, to provide more-accurate data for the support of species conservation. </td> <td style="text-align:left;"> NA </td> <td style="text-align:left;"> no </td> <td style="text-align:left;"> No good fitness measure and measure host GD </td> <td style="text-align:left;"> NA </td> <td style="text-align:left;"> no </td> <td style="text-align:left;"> no </td> </tr> <tr> <td style="text-align:left;"> 5250 </td> <td style="text-align:left;"> 2020 </td> <td style="text-align:left;"> 44111 </td> <td style="text-align:left;"> Lee, MK; Lyu, JI; Hong, MJ; Kim, DG; Kim, JM; Kim, JB; Eom, SH; Ha, BK; Kwon, SJ </td> <td style="text-align:left;"> 2019 </td> <td style="text-align:left;"> Utility of TRAP markers to determine indel mutation frequencies induced by gamma-ray irradiation of faba bean (Vicia faba L.) seeds </td> <td style="text-align:left;"> Purpose: The aims were to determine the optimal gamma ray dose for faba bean (Vicia faba) and to identify genetic variation and indel mutation frequency among mutants using target region amplification polymorphism (TRAP) markers. Materials and methods: Seeds from 10 elite lines were irradiated with gamma rays (50-700 Gy), and germination, survival rate, and representative morphological traits were measured. The extent of DNA damage was investigated using comet assays, and TRAP markers were used to evaluate genetic variation, genetic diversity, and mutation frequencies. Results: Germination percentages significantly decreased at doses &gt;100 Gy. Survival percentages and morphological traits decreased with elevation in dose. The comet assays revealed that high irradiation doses decreased head DNA levels. Phylogenetic and principal component analysis of 555 individual faba bean plants resolved eight major groups. Genetic variation between controls and mutants was limited to within groups. Mutation frequencies were associated with gamma dose in each mutant line. Conclusions: The optimal gamma dose was 100-150 Gy on the basis of survival percentage and morphological response analysis. The TRAP markers distinguished mutant lines and showed association between mutation frequency and gamma doses. This study will be useful for faba bean mutation breeding and may be applicable to other crops. </td> <td style="text-align:left;"> NA </td> <td style="text-align:left;"> no </td> <td style="text-align:left;"> No good fitness measure and measure host GD </td> <td style="text-align:left;"> NA </td> <td style="text-align:left;"> no </td> <td style="text-align:left;"> no </td> </tr> <tr> <td style="text-align:left;"> 5251 </td> <td style="text-align:left;"> 2020 </td> <td style="text-align:left;"> 44113 </td> <td style="text-align:left;"> Lee, SY; Ng, WL; Lamasudin, DU; Mohamed, R </td> <td style="text-align:left;"> 2018 </td> <td style="text-align:left;"> Inter-simple Sequence Repeat Markers Reveal Genetic Relatedness Between Natural Aquilaria Populations in Peninsular Malaysia </td> <td style="text-align:left;"> Aquilaria is a major agarwood-producing genus from the Indomalesian region. The high demand for natural agarwood resources in the market is increasing, directly threatening the survival of the trees in the wild. In order to develop proper conservation strategies for these species, information on their genetic variation is needed. The current levels of genetic variation found in Aquilaria populations in Peninsular Malaysia, as well as their genetic relationships, were evaluated using inter-simple sequence repeat (ISSR) markers. A total of 42 individuals, from 21 populations of three different Aquilaria species were collected and genotyped using 25 ISSR primers. Twenty four primers amplified bands in all three species, and showed 95%, 82.6%, and 63% polymorphic bands in A. malaccensis, A. hirta, and A. rostrata, respectively. Clustering analyses through the construction of a UPGMA dendrogram and a principal component analysis (PCA) plot revealed three major clusters, grouping samples of each species into their respective clusters. The A. malaccensis populations seemed to also be divided into two sub-clades, most probably as a result of isolation by mountain ranges as physical barriers to gene flow between the populations. Findings in this study provide valuable information for the planning of more effective management and conservation of existing Aquilaria populations in Peninsular Malaysia. </td> <td style="text-align:left;"> NA </td> <td style="text-align:left;"> no </td> <td style="text-align:left;"> No good fitness measure </td> <td style="text-align:left;"> NA </td> <td style="text-align:left;"> no </td> <td style="text-align:left;"> no </td> </tr> <tr> <td style="text-align:left;"> 5252 </td> <td style="text-align:left;"> 2020 </td> <td style="text-align:left;"> 44043 </td> <td style="text-align:left;"> Leeksl, A; dos Santos, M; West, SA </td> <td style="text-align:left;"> 2019 </td> <td style="text-align:left;"> Transmission, relatedness, and the evolution of cooperative symbionts </td> <td style="text-align:left;"> Cooperative interactions between species, termed mutualisms, play a key role in shaping natural ecosystems, economically important agricultural systems, and in influencing human health. Across different mutualisms, there is significant variation in the benefit that hosts receive from their symbionts. Empirical data suggest that transmission mode can help explain this variation: vertical transmission, where symbionts infect their host's offspring, leads to symbionts that provide greater benefits to their hosts than horizontal transmission, where symbionts leave their host and infect other hosts in the population. However, two different theoretical explanations have been given for this pattern: firstly, vertical transmission aligns the fitness interests of hosts and their symbionts; secondly, vertical transmission leads to increased relatedness between symbionts sharing a host, favouring cooperation between symbionts. We used a combination of analytical models and dynamic simulations to tease these factors apart, in order to compare their separate influences and see how they interact. We found that relatedness between symbionts sharing a host, rather than transmission mode per se, was the most important factor driving symbiont cooperation. Transmission mode mattered mainly because it determined relatedness. We also found evolutionary branching throughout much of our simulation, suggesting that a combination of transmission mode and multiplicity of infections could lead to the stable coexistence of different symbiont strategies. </td> <td style="text-align:left;"> NA </td> <td style="text-align:left;"> no </td> <td style="text-align:left;"> gd of pathogen </td> <td style="text-align:left;"> NA </td> <td style="text-align:left;"> no </td> <td style="text-align:left;"> no </td> </tr> <tr> <td style="text-align:left;"> 5253 </td> <td style="text-align:left;"> 2020 </td> <td style="text-align:left;"> 44042 </td> <td style="text-align:left;"> Leonova, IN </td> <td style="text-align:left;"> 2019 </td> <td style="text-align:left;"> Genome-Wide Association Study of Powdery Mildew Resistance in Russian Spring Wheat (T. aestivum L.) Varieties </td> <td style="text-align:left;"> Powdery mildew caused by the fungal pathogen Blumeria graminis f. sp. tritici (Bgt) is an econo-mically important disease of common wheat T. aestivum L. One of the most effective and environmentally important ways of protection of wheat against Bgt is cultivation of the varieties with genetic resistance. The aim of this work was to study the genetic diversity of Russian spring wheat varieties for the powdery mildew resistance loci. Phytopathological evaluation of 97 wheat varieties showed that no more than 10% of the varieties have low level of susceptibility to the Bgt population specific to the Western Siberian region. Association mapping carried out on the basis of SNP genotyping and phytopathological testing during three environmental seasons identified eight loci in chromosomes 1AL, 1DS, 2BL, 5AS, 5DS, 6AL, 6DL, and 7AL. A high impact to the phenotypic manifestation of the trait was established for genetic factors localized in chromosomes 5AS, 6AL, and 6DL. The long arm of chromosome 6D contains the gene Pm6Ai=2, which was introduced from wheatgrass Thinopyrum intermedium and provides effective protection against the powdery mildew pathogen. On the basis of comparative analysis of the chromosomal localization of the known Pm resistance genes and loci mapped in this work, it was assumed that QTLs in chromosomes 1DS, 5AS, and 6AL are novel, not previously described resistance loci. The obtained results can be used in breeding programs for selection of target loci and for development of molecular markers specific to Bgt resistance loci. </td> <td style="text-align:left;"> NA </td> <td style="text-align:left;"> no </td> <td style="text-align:left;"> No good fitness measure and measure host GD </td> <td style="text-align:left;"> NA </td> <td style="text-align:left;"> no </td> <td style="text-align:left;"> no </td> </tr> <tr> <td style="text-align:left;"> 5254 </td> <td style="text-align:left;"> 2020 </td> <td style="text-align:left;"> 44113 </td> <td style="text-align:left;"> Lestari, CSW; Yohan, B; Yunita, A; Meutiawati, F; Hayati, RF; Trimarsanto, H; Sasmono, RT </td> <td style="text-align:left;"> 2017 </td> <td style="text-align:left;"> Phylogenetic and evolutionary analyses of dengue viruses isolated in Jakarta, Indonesia </td> <td style="text-align:left;"> Dengue has affected Indonesia for the last five decades and become a major health problem in many cities in the country. Jakarta, the capital of Indonesia, reports dengue cases annually, with several outbreaks documented. To gain information on the dynamic and evolutionary history of dengue virus (DENV) in Jakarta, we conducted phylogenetic and evolutionary analyses of DENV isolated in 2009. Three hundred thirty-three dengue-suspected patients were recruited. Our data revealed that dengue predominantly affected young adults, and the majority of cases were due to secondary infection. A total of 171 virus isolates were successfully serotyped. All four DENV serotypes were circulating in the city, and DENV-1 was the predominant serotype. The DENV genotyping of 17 isolates revealed the presence of Genotypes I and IV in DENV-1, while DENV-2 isolates were grouped into the Cosmopolitan genotype. The grouping of isolates into Genotype I and II was seen for DENV-3 and DENV-4, respectively. Evolutionary analysis revealed the relatedness of Jakarta isolates with other isolates from other cities in Indonesia and isolates from imported cases in other countries. We revealed the endemicity of DENV and the role of Jakarta as the potential source of imported dengue cases in other countries. Our study provides genetic information regarding DENV from Jakarta, which will be useful for upstream applications, such as the study of DENV epidemiology and evolution and transmission dynamics. </td> <td style="text-align:left;"> NA </td> <td style="text-align:left;"> no </td> <td style="text-align:left;"> gd of pathogen </td> <td style="text-align:left;"> NA </td> <td style="text-align:left;"> no </td> <td style="text-align:left;"> no </td> </tr> <tr> <td style="text-align:left;"> 5255 </td> <td style="text-align:left;"> 2020 </td> <td style="text-align:left;"> 44113 </td> <td style="text-align:left;"> Levade, I; Terrat, Y; Leducq, JB; Weil, AA; Mayo-Smith, LM; Chowdhury, F; Khan, AI; Boncy, J; Buteau, J; Ivers, LC; Ryan, ET; Charles, RC; Calderwood, SB; Qadri, F; Harris, JB; LaRocque, RC; Shapiro, BJ </td> <td style="text-align:left;"> 2017 </td> <td style="text-align:left;"> Vibrio cholerae genomic diversity within and between patients </td> <td style="text-align:left;"> Cholera is a severe, water-borne diarrhoeal disease caused by toxin-producing strains of the bacterium Vibrio cholerae. Comparative genomics has revealed` waves’ of cholera transmission and evolution, in which clones are successively replaced over decades and centuries. However, the extent of V. cholerae genetic diversity within an epidemic or even within an individual patient is poorly understood. Here, we characterized V. cholerae genomic diversity at a micro-epidemiological level within and between individual patients from Bangladesh and Haiti. To capture within-patient diversity, we isolated multiple (8 to 20) V. cholerae colonies from each of eight patients, sequenced their genomes and identified point mutations and gene gain/loss events. We found limited but detectable diversity at the level of point mutations within hosts (zero to three single nucleotide variants within each patient), and comparatively higher gene content variation within hosts (at least one gain/loss event per patient, and up to 103 events in one patient). Much of the gene content variation appeared to be due to gain and loss of phage and plasmids within the V. cholerae population, with occasional exchanges between V. cholerae and other members of the gut microbiota. We also show that certain intra-host variants have phenotypic consequences. For example, the acquisition of a Bacteroides plasmid and non-synonymous mutations in a sensor histidine kinase gene both reduced biofilm formation, an important trait for environmental survival. Together, our results show that V. cholerae is measurably evolving within patients, with possible implications for disease outcomes and transmission dynamics. | NA | no | gd of pathogen | NA | no | no |
| 5256 | 2020 | 44040 | Leybourne, DJ; Bos, JIB; Valentine, TA; Karley, AJ | 2020 | The price of protection: a defensive endosymbiont impairs nymph growth in the bird cherry-oat aphid, Rhopalosiphum padi | Bacterial endosymbionts have enabled aphids to adapt to a range of stressors, but their effects in many aphid species remain to be established. The bird cherry-oat aphid, Rhopalosiphum padi (Linnaeus), is an important pest of cereals worldwide and has been reported to form symbiotic associations with Serratia symbiotica and Sitobion miscanthi L-type symbiont endobacteria, although the resulting aphid phenotype has not been described. This study presents the first report of R. padi infection with the facultative bacterial endosymbiont Hamiltonella defensa. Individuals of R. padi were sampled from populations in Eastern Scotland, UK, and shown to represent seven R. padi genotypes based on the size of polymorphic microsatellite markers; two of these genotypes harbored H. defensa. In parasitism assays, survival of H. defensa-infected nymphs following attack by the parasitoid wasp Aphidius colemani (Viereck) was 5 fold higher than for uninfected nymphs. Aphid genotype was a major determinant of aphid performance on two Hordeum species, a modern cultivar of barley H. vulgare and a wild relative H. spontaneum, although aphids infected with H. defensa showed 16% lower nymph mass gain on the partially resistant wild relative compared with uninfected individuals. These findings suggest that deploying resistance traits in barley will favor the fittest R. padi genotypes, but symbiont-infected individuals will be favored when parasitoids are abundant, although these aphids will not achieve optimal performance on a poor quality host plant. | NA | no | n | NA | no | no |
| 5257 | 2020 | 44112 | Li, CY; Sun, Y; Xie, Y; Zhou, X; Gu, XB; Lai, WM; Peng, XR; Yang, GY | 2018 | Genetic variability of wildlife-derived Sarcoptes scabiei determined by the ribosomal ITS-2 and mitochondrial 16S genes | Infestation by the ectoparasitic mite Sarcoptes scabiei (Acari: Sarcoptidae) has important implications for global wildlife conservation and both animal and human health. Ribosomal and mitochondrial DNA sequences of parasites are useful to determine genetic diversity and to describe their likely dynamic evolution. In this study, we described the genetic diversity of S. scabiei individuals collected from wild animals in China by sequencing the ribosomal ITS-2 and mitochondrial 16S rRNA genes. A total of 13 Sarcoptes isolates of wildlife, coupled with one of rabbit origin, were subjected to genetic characteristics. After cloning and sequencing, 14 ITS-2 sequences and 12 16S rRNA sequences were obtained and analyzed. Further analysis of haplotype network and population genetic structure revealed that there were 79 haplotypes in ITS-2 (main haplotype H2) and 31 haplotypes in 16S rRNA (main haplotype C10). The phylogenetic trees showed some partial clustering by location and host, and the analysis of gene polymorphism may prompt that all isolates of S. scabiei have a similar origin. We speculate that the genetic evolution of S. scabiei may be related with that of the hosts, but more research is necessary to better understand the host-parasite co-evolutionary relationship in S. scabiei. These results provide new insights into understanding the population genetics and evolutionary biology of S. scabiei and therefore a better understanding of controlling its infestation pathways worldwide. | NA | no | gd of pathogen | NA | no | no |
| 5258 | 2020 | 44111 | Li, DY; Li, Y; Li, M; Che, TD; Tian, SL; Chen, BL; Zhou, XM; Zhang, GL; Gaur, U; Luo, MJ; Tian, K; He, MN; He, S; Xu, ZX; Jin, L; Tang, QZ; Dai, YF; Xu, HL; Hu, YD; Zhao, XL; Yin, HD; Wang, Y; Zhou, RJ; Yang, CW; Du, HR; Jiang, XS; Zhu, Q; Li, MZ | 2019 | Population genomics identifies patterns of genetic diversity and selection in chicken | BackgroundThere are hundreds of phenotypically distinguishable domestic chicken breeds or lines with highly specialized traits worldwide, which provide a unique opportunity to illustrate how selection shapes patterns of genetic variation. There are many local chicken breeds in China.ResultsHere, we provide a population genome landscape of genetic variations in 86 domestic chickens representing 10 phenotypically diverse breeds. Genome-wide analysis indicated that sex chromosomes have less genetic diversity and are under stronger selection than autosomes during domestication and local adaptation. We found an evidence of admixture between Tibetan chickens and other domestic population. We further identified strong signatures of selection affecting genomic regions that harbor genes underlying economic traits (typically related to feathers, skin color, growth, reproduction and aggressiveness) and local adaptation (to high altitude). By comparing the genomes of the Tibetan and lowland fowls, we identified genes associated with high-altitude adaptation in Tibetan chickens were mainly involved in energy metabolism, body size maintenance and available food sources.ConclusionsThe work provides crucial insights into the distinct evolutionary scenarios occurring under artificial selection for agricultural production and under natural selection for success at high altitudes in chicken. Several genes were identified as candidates for chicken economic traits and other phenotypic traits. | NA | no | dometicated host | NA | no | no |
| 5259 | 2020 | 44041 | Li, F; Upadhyaya, NM; Sperschneider, J; Matny, O; Hoa, NP; Mago, R; Raley, C; Miller, ME; Silverstein, KAT; Henningsen, E; Hirsch, CD; Visser, B; Pretorius, ZA; Steffenson, BJ; Schwessinger, B; Dodds, PN; Figueroa, M | 2019 | Emergence of the Ug99 lineage of the wheat stem rust pathogen through somatic hybridisation | Parasexuality contributes to diversity and adaptive evolution of haploid (monokaryotic) fungi. However, non-sexual genetic exchange mechanisms are not defined in dikaryotic fungi (containing two distinct haploid nuclei). Newly emerged strains of the wheat stem rust pathogen, Puccinia graminis f. sp. tritici (Pgt), such as Ug99, are a major threat to global food security. Here, we provide genomics-based evidence supporting that Ug99 arose by somatic hybridisation and nuclear exchange between dikaryons. Fully haplotype-resolved genome assembly and DNA proximity analysis reveal that Ug99 shares one haploid nucleus genotype with a much older African lineage of Pgt, with no recombination or chromosome reassortment. These findings indicate that nuclear exchange between dikaryotes can generate genetic diversity and facilitate the emergence of new lineages in asexual fungal populations. | NA | no | No good fitness measure and measure host GD | NA | no | no |
| 5260 | 2020 | 44043 | Li, J; Jin, Q; Zhu, GP; Jiang, C; Zhang, AB | 2019 | Phylogeography of Dendrolimus punctatus (Lepidoptera: Lasiocampidae): Population differentiation and last glacial maximum survival | Although the Masson pine moth, Dendrolimus punctatus, is one of the most destructive forest pest insects and is an endemic condition in China, we still do not fully understand the patterns of how its distribution range varies in response to Quaternary climatic oscillations. Here, we sequenced one maternally inherited mitochondrial gene (COI) and biparentally inherited nuclear data (ITS1 and ITS2) among 23 natural populations across the entire range of the species in China. A total of 51 mitotypes and 38 ribotypes were separately obtained using mtDNA and ITS1 data. Furthermore, significant phylogeographical structure (N-ST > G(ST), p < 0.01) were detected. The spatial distribution of mitotypes implied that two distinct groups existed in the species: one in the southwest distribution, including 10 locations, and the other located in the northeast region of China. It is suggested, therefore, that each group was derived from ancestors that occupied different isolated refugia during previous periods, possibly last glacial maximum. Mismatch distribution and Bayesian population dynamics analysis suggested the population size underwent sudden expansion, which is consistent with the results of ecological niche modeling. As a typical phytophagous insect, the history of population expansion was in accordance with the host plants, providing abundant food resources and habitat. Intraspecific success rate of barcoding identification was lower than interspecific ones, indicating a level of difficulty in barcoding individuals from different populations. However, it still provides an early insight into the pattern of genetic diversity within a species. | NA | no | No good fitness measure and measure host GD | NA | no | no |
| 5261 | 2020 | 44041 | Li, J; Pei, J; Liu, YY; Xia, WW; Cheng, FF; Tian, WH; Lin, ZP; Zhu, JB; Wang, AY | 2019 | Transcriptome sequencing of Verticillium dahliae from a cotton farm reveals positive correlation between virulence and tolerance of sugar-induced hyperosmosis | Verticillium dahliae causes disease symptoms in its host plants; however, due to its rapid variability, V. dahliae is difficult to control. To analyze the reason for this pathogenic differentiation, 22 V. dahliae strains with different virulence were isolated from a cotton farm. The genetic diversity of cotton varieties make cotton cultivars have different Verticillium wilt resistance, so the Xinluzao 7 (susceptible to V. dahliae), Zhongmian 35 (tolerant), and Xinluzao 33 (resistant) were used to investigate the pathogenicity of the strains in a green house. Vegetative compatibility groups (VCGs) assays, Internal Transcribed Spacer (ITS) PCR, and pathogenicity analysis showed that SHZ-4, SHZ-5, and SHZ-9 had close kinship and significantly different pathogenicity. Transcriptome sequencing of the three strains identified 19 of 146 unigenes in SHZ-4\_vs\_SHZ-5, SHZ-5\_vs\_SHZ-9, and SHZ-4\_vs\_SHZ-9. In these unigenes, three proteinase and four polysaccharide degrading hydrolases were found to be associated with the pathogenicity. However, due to a number of differentially expressed genes in the transport, these unigenes not only played a role in nutrition absorption but might also contribute to the resistance of sugar-induced hyperosmosis. Moreover, the tolerance ability was positively related to the pathogenicity of V. dahliae. This resistance to sugar-induced hyperosmosis might help V. dahliae to access the nutrition of the host. The pathogenicity of V. dahliae correlated with the resistance of sugar-induced-hyperosmosis, which provides clues for the cultivation of V. dahliae resistant varieties. | NA | no | No good fitness measure and measure host GD | NA | no | no |
| 5262 | 2020 | 44112 | Li, JJ; Hong, XM; Mesiano, S; Muglia, LJ; Wang, XB; Snyder, M; Stevenson, DK; Shaw, GM | 2018 | Natural Selection Has Differentiated the Progesterone Receptor among Human Populations | The progesterone receptor (PGR) plays a central role in maintaining pregnancy and is significantly associated with medical conditions such as preterm birth that affects 12.6% of all the births in U.S. PGR has been evolving rapidly since the common ancestor of human and chimpanzee, and we herein investigated evolutionary dynamics of PGR during recent human migration and population differentiation. Our study revealed substantial population differentiation at the PGR locus driven by natural selection, where very recent positive selection in East Asians has substantially decreased its genetic diversity by nearly fixing evolutionarily novel alleles. On the contrary, in European populations, the PGR locus has been promoted to a highly polymorphic state likely due to balancing selection. Integrating transcriptome data across multiple tissue types together with large-scale genome-wide association data for preterm birth, our study demonstrated the consequence of the selection event in East Asians on remodeling PGR expression specifically in the ovary and determined a significant association of early spontaneous preterm birth with the evolutionarily selected variants. To reconstruct its evolutionary trajectory on the human lineage, we observed substantial differentiation between modern and archaic humans at the PGR locus, including fixation of a deleterious missense allele in the Neanderthal genome that was later introgressed in modern human populations. Taken together, our study revealed substantial evolutionary innovation in PGR even during very recent human evolution, and its different forms among human populations likely result in differential susceptibility to progesterone-associated disease conditions including preterm birth. | NA | no | human | NA | no | no |
| 5263 | 2020 | 44113 | Li, JJ; Hu, ZM; Sun, ZM; Yao, JT; Liu, FL; Fresia, P; Duan, DL | 2017 | Historical isolation and contemporary gene flow drive population diversity of the brown alga Sargassum thunbergii along the coast of China | Background: Long-term survival in isolated marginal seas of the China coast during the late Pleistocene ice ages is widely believed to be an important historical factor contributing to population genetic structure in coastal marine species. Whether or not contemporary factors (e.g. long-distance dispersal via coastal currents) continue to shape diversity gradients in marine organisms with high dispersal capability remains poorly understood. Our aim was to explore how historical and contemporary factors influenced the genetic diversity and distribution of the brown alga Sargassum thunbergii, which can drift on surface water, leading to long-distance dispersal. Results: We used 11 microsatellites and the plastid RuBisCo spacer to evaluate the genetic diversity of 22 Sargassum thunbergii populations sampled along the China coast. Population structure and differentiation was inferred based on genotype clustering and pairwise FST and allele-frequency analyses. Integrated genetic analyses revealed two genetic clusters in S. thunbergii that dominated in the Yellow-Bohai Sea (YBS) and East China Sea (ECS) respectively. Higher levels of genetic diversity and variation were detected among populations in the YBS than in the ECS. Bayesian coalescent theory was used to estimate contemporary and historical gene flow. High levels of contemporary gene flow were detected from the YBS (north) to the ECS (south), whereas low levels of historical gene flow occurred between the two regions. Conclusions: Our results suggest that the deep genetic divergence in S. thunbergii along the China coast may result from long-term geographic isolation during glacial periods. The dispersal of S. thunbergii driven by coastal currents may facilitate the admixture between southern and northern regimes. Our findings exemplify how both historical and contemporary forces are needed to understand phylogeographical patterns in coastal marine species with long-distance dispersal. | NA | no | No good fitness measure | NA | no | no |
| 5264 | 2020 | 44041 | Li, JQ; Karim, MR; Li, DF; Sumon, SMMR; Siddiki, SHMF; Rume, FI; Sun, RQ; Jia, YQ; Zhang, LX | 2019 | Molecular characterization of Blastocystis sp. in captive wildlife in Bangladesh National Zoo: Non-human primates with high prevalence and zoonotic significance | Blastocystis sp. is a protozoan parasite, commonly found in the gastrointestinal tracts of animals and humans globally. The parasitic species has wide genetic diversity. Currently the mammalian and avian isolates of the parasite are grouped into 17 well known subtypes (STs), of which ten (ST1-ST9, ST12) are reported in humans. To assess the genetic diversity of Blastocystis sp. in wildlife, a total of 200 fresh fecal samples were collected from 32 mammalian wildlife species in Bangladesh National Zoo. Blastocystis sp. was screened and subtyped by PCR amplification and sequencing of the small subunit ribosomal RNA (SSU rRNA) gene. The minimum prevalence of Blastocystis sp. infection was 15.5% (31/200) in zoo animals. Eight out of 32 wildlife animal species (25.0%) were infected with Blastocystis sp. Among them, the occurrence of Blastocystis sp. was higher in non-human primates (NHPs) (31.8%) than that in herbivores (4.9%) and carnivores (0). Nucleotide sequence analysis of the SSU rRNA gene revealed seven different Blastocystis sp. subtypes, such as ST1, ST2, ST3, ST10, ST11, ST13 and ST14 in the wild animals. ST3 was the dominant subtype (41.9%, 13/31) being detected in NHPs. Of the 31 Blastocystis sp. isolates from the wild animals, 24 (77.4%) isolates belonged to the most common subtypes (ST1 to ST3) found in humans. This is the first molecular study of Blastocystis sp. in wild animals in Bangladesh. This study highlights the remarkable genetic diversity in Blastocystis sp. isolates from zoo animals and provides the first molecular evidence from spotted deer, gayal and grey langur. Due to circulation of large percentage of potentially zoonotic subtypes in the wild animals, there is a higher risk of zoonotic transmission of Blastocystis sp. in the zoo keepers and visitors. | NA | no | gd of pathogen | NA | no | no |
| 5265 | 2020 | 44041 | Li, K; Stanojevic, M; Stamenkovic, G; Ilic, B; Paunovic, M; Lu, M; Pesic, B; Maslovara, ID; Siljic, M; Cirkovic, V; Zhang, YZ | 2019 | Insight into diversity of bacteria belonging to the order Rickettsiales in 9 arthropods species collected in Serbia | Rickettsiales bacteria in arthropods play a significant role in both public health and arthropod ecology. However, the extensive genetic diversity of Rickettsiales endosymbionts of arthropods is still to be discovered. In 2016, 515 arthropods belonging to 9 species of four classes (Insecta, Chilopoda, Diplopoda and Arachnida) were collected in Serbia. The presence and genetic diversity of Rickettsiales bacteria were evaluated by characterizing the 16S rRNA (rrs), citrate synthase (gltA) and heat shock protein (groEL) genes. The presence of various Rickettsiales bacteria was identified in the majority of tested arthropod species. The results revealed co-circulation of five recognized Rickettsiales species including Rickettsia, Ehrlichia and Wolbachia, as well as four tentative novel species, including one tentative novel genus named Neowolbachia. These results suggest the remarkable genetic diversity of Rickettsiales bacteria in certain arthropod species in this region. Furthermore, the high prevalence of spotted fever group Rickettsia in Ixodes ricinus ticks highlights the potential public health risk of human Rickettsia infection. | NA | no | No good fitness measure and measure host GD | NA | no | no |
| 5266 | 2020 | 44039 | Li, Q; Cao, ZX; Rahman, P | 2020 | Genetic variability of human angiotensin-converting enzyme 2 (hACE2) among various ethnic populations | Background There appears to be large regional variation for susceptibility, severity, and mortality for COVID-19 infections. Numerous potential factors could explain the wide variability in the number of infections and death among the countries. We examined genetic differences in the human angiotensin-converting enzyme 2 (hACE2) gene, as its receptor serves as a cellular entry for SARS-CoV-2. At present, there is a paucity of data regarding the differences for ACE2 polymorphisms and expression levels between ethnicities. Methods We compared the allele frequency of mutations between European and East Asians. Molecular dynamic simulation were performed to investigate the influences of significant mutant on protein structure. The binding free energies were calculated between S protein and hACE2. We also examined hACE2 gene expression in eight global populations from HapMap3. Results Four missense mutations showed significant minor allele frequency difference between Asians and Caucasians. Molecular dynamic demonstrated that two of these variants (K26R and I468V) may affect binding characteristics between S protein of the virus and hACE2 receptor. We also noted marginal differences in gene expression for some populations in HapMap3 as compared to the Chinese population. Conclusion Our studies reveal subtle changes in the genetics of hACE2 between human populations, but the magnitude of the difference was small and the significance is not clear in the absence of further in vitro and functional studies. | NA | no | Covid-19 , not relevate | NA | no | no |
| 5267 | 2020 | 44040 | Li, R; Li, C; Chen, H; Li, R; Chong, Q; Xiao, H; Chen, S | 2020 | Genome-wide scan of selection signatures in Dehong humped cattle for heat tolerance and disease resistance | Dehong humped cattle (DHH) is an indigenous zebu breed from southwestern China that possesses characteristics of heat tolerance and strong disease resistance and adapts well to the local tropical and subtropical climatic conditions. However, information on selection signatures of DHH is scarce. Herein, we compared the genomes of DHH and each of Diqing and Zhaotong cattle breeds using the population differentiation index (F-ST), cross-population extended haplotype homozygosity (XP-EHH) and cross-population composite likelihood ratio (XP-CLR) methods to explore the genomic signatures of heat tolerance and disease resistance in DHH. Several pathways and genes carried selection signatures, including thermal sweating (calcium signaling pathway), heat shock (HSF1) and oxidative stress response (PLCB1, PLCB4), coat color (RAB31), feed intake (ATP8A1, SHC3) and reproduction (TP63, MAP3K13, PTPN4, PPP3CC, ADAMTSL1, SS18L1, OSBPL2, TOX, RREB1, GRK2). These identified pathways and genes may contribute to heat tolerance in DHH. Simultaneously, we also identified LIPH, TP63 and CBFA2T3 genes under positive selection that were associated with immunity. | NA | no | dometicated host | NA | no | no |
| 5268 | 2020 | 44111 | Lichtenauer, W; van de Pol, M; Cockburn, A; Brouwer, L | 2019 | Indirect fitness benefits through extra-pair mating are large for an inbred minority, but cannot explain widespread infidelity among red-winged fairy-wrens | Extra-pair paternity (EPP) has been suggested to improve the genetic quality of offspring, but evidence has been equivocal. Benefits of EPP may be only available to specific individuals or under certain conditions. Red-winged fairy-wrens have extremely high levels of EPP, suggesting fitness benefits might be large and available to most individuals. Furthermore, extreme philopatry commonly leads to incestuous social pairings, so inbreeding avoidance may be an important selection pressure. Here, we quantified the fitness benefits of EPP under varying conditions and across life-stages. Extra-pair offspring (EPO) did not appear to have higher fitness than within-pair offspring (WPO), neither in poor years nor in the absence of helpers-at-the-nest. However, EPP was beneficial for closely related social pairs, because inbred WPO suffered an overall 75% reduction in fitness. Inbreeding depression was nonlinear and reduced nestling body condition, first year survival and reproductive success. Our comprehensive study indicates that EPP should be favored for the 17% of females paired incestuously, but cannot explain the widespread infidelity in this species. Furthermore, our finding that fitness benefits of EPP only become apparent for a small part of the population could potentially explain the apparent absence of fitness differences in population wide comparisons of EPO and WPO. | NA | no | no good gd measure host pop | NA | no | no |
| 5269 | 2020 | 44039 | Lilley, TM; Wilson, IW; Field, KA; Reeder, DM; Vodzak, ME; Turner, GG; Kurta, A; Blomberg, AS; Hoff, S; Herzog, CJ; Sewall, BJ; Paterson, S | 2020 | Genome-Wide Changes in Genetic Diversity in a Population of Myotis lucifugus Affected by White-Nose Syndrome | Novel pathogens can cause massive declines in populations, and even extirpation of hosts. But disease can also act as a selective pressure on survivors, driving the evolution of resistance or tolerance. Bat white-nose syndrome (WNS) is a rapidly spreading wildlife disease in North America. The fungus causing the disease invades skin tissues of hibernating bats, resulting in disruption of hibernation behavior, premature energy depletion, and subsequent death. We used whole-genome sequencing to investigate changes in allele frequencies within a population of Myotis lucifugus in eastern North America to search for genetic resistance to WNS. Our results show low F-ST values within the population across time, i.e., prior to WNS (Pre-WNS) compared to the population that has survived WNS (Post-WNS). However, when dividing the population with a geographical cut-off between the states of Pennsylvania and New York, a sharp increase in values on scaffold GL429776 is evident in the Post-WNS samples. Genes present in the diverged area are associated with thermoregulation and promotion of brown fat production. Thus, although WNS may not have subjected the entire M. lucifugus population to selective pressure, it may have selected for specific alleles in Pennsylvania through decreased gene flow within the population. However, the persistence of remnant sub-populations in the aftermath of WNS is likely due to multiple factors in bat life history. | NA | no | No good fitness measure and measure host GD | NA | no | no |
| 5270 | 2020 | 44111 | Lin, WH; Shih, HC; Lin, CF; Yang, CY; Lin, CN; Chiou, MT | 2019 | Genotypic analyses and virulence characterization of Glaesserella parasuis isolates from Taiwan | Background: Glaesserella (Haemophilus) parasuis (G. parasuis) causes severe economic losses in the swine industry. Multiple G. parasuis strains can exist in single animals. Typing techniques are required for identifying G. parasuis isolates. Different strains within a serovar display varying virulence. Enterobacterial repetitive intergenic consensus polymerase chain reaction (ERIC-PCR) can assess the heterogeneity. The group 1 virulence-associated trimeric autotransporters (vtaA) gene is an indicator of virulence. The aim of this study was to characterize Taiwanese G. parasuis isolates via molecular serotyping, vtaA PCR and ERIC-PCR. Methods: One hundred and forty-five strains were collected between November 2013 and March 2017 in Taiwan and further examined by molecular serotyping, vtaA PCR and ERIC-PCR. Results: The dendrogram revealed heterogeneous genetic diversity within many clusters. Partial correlation between the ERIC-PCR clusters of different strains, serovars and lesion patterns was observed. Twelve herds (8.3%) infected with more than one strain. Group 1 vtaA positive rate reached 98.6%. Discussion: This study showed the high genetic diversity of G. parasuis in Taiwan by a high discriminatory capability of ERIC-PCR. Group 1 vtaA commonly exists in G. parasuis isolates and may play important roles in the pathogenesis of Taiwanese G. parasuis isolates. | NA | no | No good fitness measure and measure host GD | NA | no | no |
| 5271 | 2020 | 44043 | Linde, CC; Smith, LM | 2019 | Host specialisation and disparate evolution of Pyrenophora teres f. teres on barley and barley grass | BackgroundPathogens evolve in an arms race, frequently evolving virulence that defeats resistance genes in their hosts. Infection of multiple hosts may accelerate this virulence evolution. Theory predicts that host diversity affects pathogen diversity, with more diverse hosts expected to harbour more diverse pathogens that reproduce sexually. We tested this hypothesis by comparing the microsatellite (SSR) genetic diversity of the barley leaf pathogen Pyrenophora teres f. teres (Ptt) from barley (monoculture) and barley grass (outbreeding). We also aim to investigate host specificity and attempt to track virulence on two barley cultivars, Maritime and Keel.ResultsGenetic diversity in barley Ptt populations was higher than in populations from barley grass. Barley Ptt populations also had higher linkage disequilibrium levels, indicating less frequent sexual reproduction, consistent with the Red Queen hypothesis theory that genetically diverse hosts should select for higher levels of sexual reproduction of the pathogen. SSR analyses indicate that host-associated Ptt populations do not share genotypes and have independent evolutionary histories. Pathogenicity studies showed host specificity as host-associated Ptt isolates could not cross-infect hosts. Minimum spanning network analyses indicated two major clusters of barley Ptt. One cluster represents Maritime virulent and isolates from Western Australia (WA). Low PhiPt population differentiation between WA populations and those from Maritime and Keel, indicated a WA origin of the Maritime and Keel virulences. The main minimum spanning network cluster is represented by a panmictic population structure, represented by isolates from all over Australia.ConclusionsAlthough barley Ptt populations are more diverse than barley grass Ptt populations, this may be a result of the size and number of founder Ptt populations to Australia, with larger and more barley Ptt populations introduced. More frequent sexual reproduction of Ptt on barley grass support the Red Queen Hypothesis and suggest evolutionary potential of pathogens on diverse hosts are high. Extensive gene flow of Ptt between regions in Australia is suggested to maintain a panmictic population structure, with human-mediated dispersal aiding in virulence evolution of Ptt on barley. | NA | no | No good fitness measure and measure host GD | NA | no | no |
| 5272 | 2020 | 44113 | Linster, M; Do, LAH; Minh, NNQ; Chen, YH; Zhe, Z; Tuan, TA; Tuan, HM; Su, YCF; van Doorn, HR; Moorthy, M; Smith, GJD | 2018 | Clinical and Molecular Epidemiology of Human Parainfluenza Viruses 1-4 in Children from Viet Nam | HPIVs are serologically and genetically grouped into four species that account for up to 10% of all hospitalizations due to acute respiratory infection in children under the age of five. Genetic and epidemiological data for the four HPIVs derived from two pediatric cohorts in Viet Nam are presented. Respiratory samples were screened for HPIV1-4 by real-time PCR. Demographic and clinical data of patients infected with different HPIV were compared. We used a hemi-nested PCR approach to generate viral genome sequences from HPIV-positive samples and conducted a comprehensive phylogenetic analysis. In total, 170 samples tested positive for HPIV. HPIV3 was most commonly detected in our cohort and 80 co-detections of HPIV with other respiratory viruses were found. Phylogenetic analyses suggest local endemic circulation as well as punctuated introductions of new HPIV lineages. Viral gene flow analysis revealed that Viet Nam is a net importer of viral genetic diversity. Epidemiological analyses imply similar disease severity for all HPIV species. HPIV sequences from Viet Nam formed local clusters and were interspersed with sequences from diverse geographic regions. Combined, this new knowledge will help to investigate global HPIV circulation patterns in more detail and ultimately define more suitable vaccine strains. | NA | no | human | NA | no | no |
| 5273 | 2020 | 44043 | Litzke, V; Ottensmann, M; Forcada, J; Heitzmann, L; Hoffman, JI | 2019 | Heterozygosity at neutral and immune loci is not associated with neonatal mortality due to microbial infection in Antarctic fur seals | Numerous studies have reported correlations between the heterozygosity of genetic markers and fitness. These heterozygosity-fitness correlations (HFCs) play a central role in evolutionary and conservation biology, yet their mechanistic basis remains open to debate. For example, fitness associations have been widely reported at both neutral and functional loci, yet few studies have directly compared the two, making it difficult to gauge the relative contributions of genome-wide inbreeding and specific functional genes to fitness. Here, we compared the effects of neutral and immune gene heterozygosity on death from bacterial infection in Antarctic fur seal (Arctocephalus gazella) pups. We specifically developed a panel of 13 microsatellites from expressed immune genes and genotyped these together with 48 neutral loci in 234 individuals, comprising 39 pups that were classified at necropsy as having most likely died of bacterial infection together with a five times larger matched sample of healthy surviving pups. Identity disequilibrium quantified from the neutral markers was positive and significant, indicative of variance in inbreeding within the study population. However, multilocus heterozygosity did not differ significantly between healthy and infected pups at either class of marker, and little evidence was found for fitness associations at individual loci. These results support a previous study of Antarctic fur seals that found no effects of heterozygosity at nine neutral microsatellites on neonatal survival and thereby help to refine our understanding of how HFCs vary across the life cycle. Given that nonsignificant HFCs are underreported in the literature, we also hope that our study will contribute toward a more balanced understanding of the wider importance of this phenomenon. | NA | no | no good gd measure host | NA | no | no |
| 5274 | 2020 | 44041 | Liu, D; Cui, YJ; He, JH; Li, SY; Li, Q; Liang, D; Wang, JH; Shi, XW; Wang, CL; Dong, KJ; Liu, TP; Zhang, L; Ren, RY; Yang, TY; Feng, G; Liu, ZL | 2019 | Genetic Diversity and Classification of the Cytoplasm of Chinese Elite Foxtail Millet [Setaria italica (L.) P. Beauv.] Parental Lines Revealed by Chloroplast Deoxyribonucleic Acid Variation | Due to the maternal inheritance of cytoplasm, using foxtail millet [Setaria italica (L.) P. Beauv.] male sterile lines with a single cytoplasmic source as the female parent will inevitably lead to a narrow source of cytoplasm in hybrids, which may make them vulnerable to infection by cytoplasm-specific pathogens, ultimately leading to destructive yield losses. To assess cytoplasmic genetic diversity in plants, molecular markers derived from chloroplast DNA (cpDNA) have been used. However, such markers have not yet been applied to foxtail millet. In this study, we designed and screened nine pairs of polymorphic foxtail millet-specific primers based on its completely sequenced cpDNA. Using these primers, we analyzed the genetic diversity and cytoplasmic types of 130 elite foxtail millet parental lines collected in China. Our results revealed that the cytoplasmic genetic diversity of these accessions was low and needs to be increased. The parental lines were divided into four cytoplasmic types according to population structure analysis and a female parent-derivative evolutionary graph, indicating that the cytoplasmic types of elite foxtail millet lines were rather limited. A principal component analysis (PCA) plot was linked with the geographic and ecological distribution of accessions for each cytoplasmic type, as well as their basal maternal parents. Collectively, our results suggest that enriching cytoplasmic sources through the use of accessions from diverse ecological regions and other countries as the female parent may improve foxtail millet breeding programs, and prevent infection by cytoplasm-specific pathogens. | NA | no | No good fitness measure and measure host GD | NA | no | no |
| 5275 | 2020 | 44042 | Liu, JD; Rasheed, A; He, ZH; Imtiaz, M; Arif, A; Mahmood, T; Ghafoor, A; Siddiqui, SU; Ilyas, MK; Wen, WE; Gao, FM; Xie, CJ; Xia, XC | 2019 | Genome-wide variation patterns between landraces and cultivars uncover divergent selection during modern wheat breeding | Key message Genetic diversity, population structure, LD decay, and selective sweeps in 687 wheat accessions were analyzed, providing relevant guidelines to facilitate the use of the germplasm in wheat breeding. Common wheat (Triticum aestivum L.) is one of the most widely grown crops in the world. Landraces were subjected to strong human-mediated selection in developing high-yielding, good quality, and widely adapted cultivars. To investigate the genome-wide patterns of allelic variation, population structure and patterns of selective sweeps during modern wheat breeding, we tested 687 wheat accessions, including landraces (148) and cultivars (539) mainly from China and Pakistan in a wheat 90 K single nucleotide polymorphism array. Population structure analysis revealed that cultivars and landraces from China and Pakistan comprised three relatively independent genetic clusters. Cultivars displayed lower nucleotide diversity and a wider average LD decay across whole genome, indicating allelic erosion and a diversity bottleneck due to the modern breeding. Analysis of genetic differentiation between landraces and cultivars from China and Pakistan identified allelic variants subjected to selection during modern breeding. In total, 477 unique genome regions showed signatures of selection, where 109 were identified in both China and Pakistan germplasm. The majority of genomic regions were located in the B genome (225), followed by the A genome (175), and only 77 regions were located in the D genome. EigenGWAS was further used to identify key selection loci in modern wheat cultivars from China and Pakistan by comparing with global winter wheat and spring wheat diversity panels, respectively. A few known functional genes or loci found within these genome regions corresponded to known phenotypes for disease resistance, vernalization, quality, adaptability and yield-related traits. This study uncovered molecular footprints of modern wheat breeding and explained the genetic basis of polygenic adaptation in wheat. The results will be useful for understanding targets of modern wheat breeding, and in devising future breeding strategies to target beneficial alleles currently not pursued. | NA | no | dometicated host | NA | no | no |
| 5276 | 2020 | 44111 | Liu, Q; Hu, Q; Yao, S; Kwan, ML; Roh, JM; Zhao, H; Ambrosone, CB; Kushi, LH; Liu, S; Zhu, QQ | 2019 | SeqSQC: A Bioconductor Package for Evaluating the Sample Quality of Next-generation Sequencing Data | As next-generation sequencing (NGS) technology has become widely used to identify genetic causal variants for various diseases and traits, a number of packages for checking NGS data quality have sprung up in public domains. In addition to the quality of sequencing data, sample quality issues, such as gender mismatch, abnormal inbreeding coefficient, cryptic relatedness, and population outliers, can also have fundamental impact on downstream analysis. However, there is a lack of tools specialized in identifying problematic samples from NGS data, often due to the limitation of sample size and variant counts. We developed SeqSQC, a Bioconductor package, to automate and accelerate sample cleaning in NGS data of any scale. SeqSQC is designed for efficient data storage and access, and equipped with interactive plots for intuitive data visualization to expedite the identification of problematic samples. SeqSQC is available at http://bioconductor.org/packages/SeqSQC. | NA | no | No good fitness measure and measure host GD | NA | no | no |
| 5277 | 2020 | 44042 | Liu, TT; Feng, XJ; Zhang, YD; Liu, JZ; Bao, R | 2019 | Genetic diversity of Haemaphysalis longicornis from China and molecular detection of Rickettsia | The tick Haemaphysalis longicornis (Neumann) (Acari, Ixodidae) is distributed throughout China and is the most notorious blood feeding ectoparasite of livestock. Haemaphysalis longicornis can transmit a large number of pathogens that cause human infectious diseases, such as Rickettsia spp. Here, we characterized the genetic structure of H. longicornis and tested for the presence of Rickettsia spp. from five regions in China. Analysis of the two mitochondrial marker sequences (16S rRNA and COI) and the nuclear sequence (ITS2) showed that the overall level of nucleotide diversity was low and the variability did not differ among the five regions. From the five locations, the infection rates of Rickettsia species ranged from 0 to 65%. The nucleotide diversities of the high-infected group were lower than those of the low- and uninfected group. And in neutrality tests for the high-infected group based on COI sequences, the Tajima’s D and Fu’s FS were coincidentally < 0 and significant, whereas they were closer to zero and non-significant in low- and uninfected groups. | NA | no | No good fitness measure and measure host GD | NA | no | no |
| 5278 | 2020 | 44113 | Liu, WJ; Aaskov, JG | 2018 | Fitness peaks of dengue virus populations | The role of intra-host genetic diversity in dengue viral populations remains a topic of debate, particularly the impact on transmission of changes in this diversity. Several approaches have been taken to increasing and decreasing the genetic diversity of populations of RNA viruses and have drawn what appear to be contradictory conclusions. A 2-6 fold increase in genetic diversity of a wild type population of dengue virus serotype 1 (DENV1) and of an infectious clone population derived from the wild type population, produced by treatment with nucleotide analogue 5 fluorouracil (5FU), drove the populations to extinction. Removal of 5FU immediately prior to extinction, resulted in a return to pre-treatment levels of fitness and genetic diversity, albeit with novel single nucleotide polymorphisms. These observations support the concept that DENV populations exist on fitness peaks determined by their transmission requirements and either an increase or a decrease in genetic diversity may result in a loss of fitness. | NA | no | gd of pathogen | NA | no | no |
| 5279 | 2020 | 44113 | Lloyd-Jones, LR; Robinson, MR; Yang, J; Visscher, PM | 2018 | Transformation of Summary Statistics from Linear Mixed Model Association on All-or-None Traits to Odds Ratio | Genome-wide association studies (GWAS) have identified thousands of loci that are robustly associated with complex diseases. The use of linear mixed model (LMM) methodology for GWAS is becoming more prevalent due to its ability to control for population structure and cryptic relatedness and to increase power. The odds ratio (OR) is a common measure of the association of a disease with an exposure (e.g., a genetic variant) and is readably available from logistic regression. However, when the LMM is applied to all-or-none traits it provides estimates of genetic effects on the observed 0-1 scale, a different scale to that in logistic regression. This limits the comparability of results across studies, for example in a meta-analysis, and makes the interpretation of the magnitude of an effect from an LMM GWAS difficult. In this study, we derived transformations from the genetic effects estimated under the LMM to the OR that only rely on summary statistics. To test the proposed transformations, we used real genotypes from two large, publicly available data sets to simulate all-or-none phenotypes for a set of scenarios that differ in underlying model, disease prevalence, and heritability. Furthermore, we applied these transformations to GWAS summary statistics for type 2 diabetes generated from 108,042 individuals in the UK Biobank. In both simulation and real-data application, we observed very high concordance between the transformed OR from the LMM and either the simulated truth or estimates from logistic regression. The transformations derived and validated in this study improve the comparability of results from prospective and already performed LMM GWAS on complex diseases by providing a reliable transformation to a common comparative scale for the genetic effects. | NA | no | No good fitness measure | NA | no | no |
| 5280 | 2020 | 44112 | Lo, E; Bonizzoni, M; Hemming-Schroeder, E; Ford, A; Janies, DA; James, AA; Afrane, Y; Etemesi, H; Zhou, GF; Githeko, A; Yan, GY | 2018 | Selection and Utility of Single Nucleotide Polymorphism Markers to Reveal Fine-Scale Population Structure in Human Malaria Parasite Plasmodium falciparum | Single nucleotide polymorphisms (SNPs) have been shown to be useful in revealing population structure with continental-and regional-scale samples. In epidemiological study, a careful selection of SNPs to track disease spread in local communities would provide an important addition to traditional disease surveillance. This study used SNPs and microsatellites to examine population structure of Plasmodium falciparum at fine- scale in malaria-endemic areas of Western Kenya. A set of high performance (HP) SNPs were selected from a large SNP panel based on BELS ranking, FST values and minor allele frequency criteria. The discriminative power and assignment accuracy of different SNP panels including nonsynonymous SNPs, silent SNPs, previously published barcode SNPs, and the HP SNPs were evaluated together with microsatellites. Among all SNP panels, HP SNPs showed the highest level of differentiation and self-assignment accuracy on average among sites. Clear distinction was observed between the northern and southern P. falciparum samples, whereas samples from the south were least diverged from one another. These results were comparable to those by microsatellites. Nonsynonymous, silent, and barcode SNPs all showed similar levels of genetic variability to one another and weaker structure than the HP SNPs. We described here the procedure of selecting a set of HP SNPs from a large panel of SNPs that resolve population structure of P. falciparum between the northern and southern regions of Western Kenya. Future work is needed to determine if this procedure can result in SNPs panels capable of tracing Plasmodium spread at finer geographical scales. | NA | no | gd of pathogen | NA | no | no |
| 5281 | 2020 | 44113 | Lompo, D; Vinceti, B; Gaisberger, H; Konrad, H; Duminil, J; Ouedraogo, M; Sina, S; Geburek, T | 2017 | Genetic conservation in Parkia biglobosa (Fabaceae: Mimosoideae) - what do we know? | The medicinal and food tree species Parkia biglobosa (Fabaceae: Mimosoideae) is widespread in the Sudanian savannahs of sub-Saharan Africa, where it has a strong socio-cultural and economic importance. Populations of this species are highly threatened in large parts of its range due to overexploitation and environmental degradation. In the light of climatic changes, safeguarding the genetic diversity of the species is crucial to foster adaptation and to support its long-term survival. Genetic insight is also relevant to guide sustainable harvesting. This paper has the objective to review information on the species’ geographic distribution, reproductive biology, genetic characteristics and existing conservation practices, and to identify knowledge gaps to orientate future conservation and research focus. The literature review revealed that the species is mainly outcrossed and is pollinated by a diversity of vectors, including bats that allow long-pollen dispersal. When bats are absent, pollination is mainly carried out by honey bees and stingless bees and in such case pollen-mediated gene flow is relatively restricted. Data of a large-scale genetic study based on allozyme markers showing a moderate genetic differentiation among populations were reanalyzed using an inverse distance weighted interpolation function. Three distinctive regions of diversity based on allelic richness and expected heterozygosity were identified. Finally, we discuss future challenges for genetic conservation by emphasizing the need to use both neutral and adaptive markers in future research. | NA | no | No good fitness measure and measure host GD | NA | no | no |
| 5282 | 2020 | 44112 | Long, E; Evans, C; Chaston, J; Udall, JA | 2018 | Genomic Structural Variations Within Five Continental Populations of Drosophila melanogaster | Chromosomal structural variations (SV) including insertions, deletions, inversions, and translocations occur within the genome and can have a significant effect on organismal phenotype. Some of these effects are caused by structural variations containing genes. Large structural variations represent a significant amount of the genetic diversity within a population. We used a global sampling of Drosophila melanogaster (Ithaca, Zimbabwe, Beijing, Tasmania, and Netherlands) to represent diverse populations within the species. We used long-read sequencing and optical mapping technologies to identify SVs in these genomes. Among the five lines examined, we found an average of 2,928 structural variants within these genomes. These structural variations varied greatly in size and location, included many exonic regions, and could impact adaptation and genomic evolution. | NA | no | No good fitness measure and measure host GD | NA | no | no |
| 5283 | 2020 | 44112 | Lopes, PC; Block, P; Pontiggia, A; Lindholm, AK; Konig, B | 2018 | No evidence for kin protection in the expression of sickness behaviors in house mice | When infected, animals change their behaviors in several ways, including by decreasing their activity, their food and water intake, and their interest in social interactions. These behavioral alterations are collectively called sickness behaviors and, for several decades, the main hypotheses put forward to explain this phenomenon were that engaging in sickness behaviors facilitated the fever response and improved the likelihood of host survival. However, a new hypothesis was recently proposed suggesting that engaging in sickness behaviors may serve to protect kin. We tested this kin protection hypothesis by combining a field and a laboratory experiment in house mice. In both experiments, we induced sickness behaviors by administration of a pro-inflammatory agent. In the field experiment, we then collected genetic data and assessed whether relatedness affected the intensity of sickness behaviors. In the lab experiment, we manipulated relatedness in small social groups and assessed whether having a closely related individual (a sibling) in the group altered social interactions or visits to common resources (such as food and water containers) once immune-challenged. Our results do not support the kinship protection hypothesis and therefore advance our understanding of why such an apparently costly set of behavioral changes would be evolutionarily maintained. | NA | no | No good fitness measure and measure host GD | NA | no | no |
| 5284 | 2020 | 44040 | Lopez, MG; Dogba, JB; Torres-Puente, M; Goig, GA; Moreno-Molina, M; Villamayor, LM; Cadmus, S; Comas, I | 2020 | Tuberculosis in Liberia: high multidrug-resistance burden, transmission and diversity modelled by multiple importation events | Tuberculosis (TB) surveillance is scarce in most African countries, even though it is the continent with the greatest disease incidence according to the World Health Organization. Liberia is within the 30 countries with the highest TB burden, probably as a consequence of the long civil war and the recent Ebola outbreak, both crippling the health system and depreciating the TB prevention and control programmes. Due to difficulties working in the country, there is a lack of resistance surveys and bacillus characterization. Here, we use genome sequencing of Mycobacterium tuberculosis clinical isolates to fill this gap. Our results highlight that the bacillus population structure is dominated by lineage 4 strains that harbour an outstanding genetic diversity, higher than in the rest of Africa as a whole. Coalescent analyses demonstrate that strains currently circulating in Liberia were introduced several times beginning in the early year 600 CE until very recently coinciding with migratory movements associated with the civil war and Ebola epidemics. A higher multidrug-resistant (MDR)-TB frequency (23.5 %) than current estimates was obtained together with non-catalogued drug-resistance mutations. Additionally, 39 % of strains were in genomic clusters revealing that ongoing transmission is a major contribution to the TB burden in the country. Our report emphasizes the importance of TB surveillance and control in African countries where bacillus diversity, MDR-TB prevalence and transmission are coalescing to jeopardize TB control programmes. | NA | no | gd of pathogen / human | NA | no | no |
| 5285 | 2020 | 44041 | Lott, MJ; Wright, BR; Kemp, LF; Johnson, RN; Hogg, CJ | 2020 | Genetic Management of Captive and Reintroduced Bilby Populations | Captive breeding and translocation, whereby selected individuals are used to supplement or re-establish failing populations, are powerful tools for conserving threatened fauna. These tools, however, are rarely successful at establishing self-sustaining populations that can survive without ongoing human assistance. The maintenance of genetic diversity and demographic security in captivity, or following wildlife translocation events, is important for improving the long-term effectiveness of threatened species recovery efforts around the world. Routine population monitoring using hypervariable genetic markers represents a promising technique for evaluating the effect of established management practices on population structure and genetic diversity across various spatial and temporal scales. In this study, we employed a data set of 1,068 single nucleotide polymorphisms to conduct a comprehensive survey of population structure and genetic diversity in greater bilbies (Macrotis lagotis) held at 13 zoos and wildlife sanctuaries across Australia between August 1996 and December 2016. We observed significant genetic structuring across the study sites, consistent with the limited exchange of animals between independently managed facilities. The majority of variation, however, still occurred at the level of individual bilbies (75%, P < 0.001). We also uncovered evidence for an ongoing loss of genetic diversity in some conservation-fenced populations, despite a slight excess of heterozygosity across the sampling sites as a whole. Maintaining the genetic health of bilbies in captivity or following translocation will therefore require stakeholders to focus on reducing individual mortality, and maintaining genetic connectivity across all existing populations through the regular exchange of selected individuals. As such, admixture is expected to play an increasingly important role in future conservation programs. (c) 2019 The Authors. Journal of Wildlife Management published by Wiley Periodicals, Inc. on behalf of The Wildlife Society. | NA | no | No good fitness measure and measure host GD | NA | no | no |
| 5286 | 2020 | 44113 | Lounnas, M; Correa, AC; Alda, P; David, P; Dubois, MP; Calvopina, M; Caron, Y; Celi-Erazo, M; Dung, BT; Jarne, P; Loker, ES; Noya, O; Rodriguez-Hidalgo, R; Toty, C; Uribe, N; Pointier, JP; Hurtrez-Bousses, S | 2018 | Population structure and genetic diversity in the invasive freshwater snail Galba schirazensis (Lymnaeidae) | We studied the population genetic structure of the freshwater snail Galba schirazensis (Kuster, 1862), a potential vector of infectious diseases such as fascioliasis. Galba schirazensis has now a worldwide distribution but a poorly known origin because this species has been distinguished only recently from the morphologically similar and cosmopolitan Galba truncatula (O.F. Muller, 1774). Wedeveloped specific microsatellite markers and sequenced amitochondrial gene (cytochrome oxidase subunit I (CO1)) to study individuals of G. schirazensis from the Old World and the New World. We found very low genetic diversity within populations, no heterozygotes, and marked population structure-a pattern observed in other highly selfing lymnaeid species with recently enlarged distributions as a result of biological invasions. The total lack of observed heterozygosity in the few populations of G. schirazensis that displayed some allelic diversity suggests high selfing rates. We also found that the center of diversity, and by extension the origin area of this species, should be found in the New World, whereas Old World populations should rather result from a recent introduction of a genetically uniform population. The microsatellite markers developed here will help to clarify the history of expansion of G. schirazensis and might help to understand its role as a potential vector of infectious diseases. | NA | no | No good fitness measure | NA | no | no |
| 5287 | 2020 | 44113 | Lu, JQ; Lou, HY; Fu, RQ; Lu, DS; Zhang, F; Wu, ZD; Zhang, X; Li, CH; Fang, BJ; Pu, FF; Wei, JN; Wei, Q; Zhang, C; Wang, XJ; Lu, Y; Yan, S; Yang, YJ; Jin, L; Xu, SH | 2017 | Assessing genome-wide copy number variation in the Han Chinese population | Background Copy number variation (CNV) is a valuable source of genetic diversity in the human genome and a well-recognised cause of various genetic diseases. However, CNVs have been considerably under-represented in population-based studies, particularly the Han Chinese which is the largest ethnic group in the world. Objectives To build a representative CNV map for the Han Chinese population. Methods We conducted a genome-wide CNV study involving 451 male Han Chinese samples from 11 geographical regions encompassing 28 dialect groups, representing a less-biased panel compared with the currently available data. We detected CNVs by using 4.2M NimbleGen comparative genomic hybridisation array and whole-genome deep sequencing of 51 samples to optimise the filtering conditions in CNV discovery. Results A comprehensive Han Chinese CNV map was built based on a set of high-quality variants (positive predictive value >0.8, with sizes ranging from 369 bp to 4.16 Mb and a median of 5907 bp). The map consists of 4012 CNV regions (CNVRs), and more than half are novel to the 30 East Asian CNV Project and the 1000 Genomes Project Phase 3. We further identified 81 CNVRs specific to regional groups, which was indicative of the subpopulation structure within the Han Chinese population. Conclusions Our data are complementary to public data sources, and the CNV map may facilitate in the identification of pathogenic CNVs and further biomedical research studies involving the Han Chinese population. | NA | no | human | NA | no | no |
| 5288 | 2020 | 44112 | Luna-Pineda, VM; Ochoa, SA; Cruz-Cordova, A; Cazares-Dominguez, V; Reyes-Grajeda, JP; Flores-Oropeza, MA; Arellano-Galindo, J; Castro-Hernandez, R; Flores-Encarnacion, M; Ramirez-Vargas, A; Flores-Garcia, HJ; Moreno-Fierros, L; Xicohtencatl-Cortes, J | 2018 | Features of urinary Escherichia coli isolated from children with complicated and uncomplicated urinary tract infections in Mexico | The Hospital Infantil de Mexico Federico Gomez (HIMFG) is a tertiary care hospital in Mexico City where Escherichia coli is frequently isolated from the urine samples of pediatric patients with urinary tract infections. A collection of 178 urinary Escherichia coli (UEc) isolates associated with complicated and uncomplicated urinary tract infections were evaluated in this study. The patterns of resistance to 9 antibiotic classes showed that 60.7% of the UEc isolates had a highly multidrug-resistant (MDR) profile. Genetic diversity analyses of the UEc isolates showed a high variability and revealed 16 clusters associated with four phylogenetic groups, namely, groups A, B1, B2, and D. Phylogenetic group B2 was widely associated with the 16 clusters as well as with virulence and fitness genes. The virulence and fitness genes in the UEc isolates, which included fimbriae-, siderophore-, toxin-, and mobility-associated genes, were grouped as occurring at a low, variable, or high frequency. Interestingly, only the papF gene could be amplified from some UEc isolates, and the sequence analysis of the pap operon identified an insertion sequence (IS) element and gene loss. These data suggested pathoadaptability and the development of immune system evasion, which was confirmed by the loss of P fimbriae-associated agglutination in the UEc isolates. E. coli clone O25-ST131 had a prevalence of 20.2% among the UEc isolates; these isolates displayed both a highly MDR profile and the presence of the papGII, fimH, papGIII, iutD, sat, hlyA, and motA genes. In conclusion, the UEc isolates from complicated urinary tract infection (cUTI) were characterized as being MDR, highly genetically diverse, and associated with phylogenetic group B2 and many virulence and fitness genes. Additionally, gene loss and IS elements were identified in some UEc isolates identified as clone O25-ST131. | NA | no | No good fitness measure and measure host GD | NA | no | no |
| 5289 | 2020 | 44039 | Lundberg, M; Zhong, XQ; Konrad, A; Olsen, RA; Raberg, L | 2020 | Balancing selection in Pattern Recognition Receptor signalling pathways is associated with gene function and pleiotropy in a wild rodent | Pathogen-mediated balancing selection is commonly considered to play an important role in the maintenance of genetic diversity, in particular in immune genes. However, the factors that may influence which immune genes are the targets of such selection are largely unknown. To address this, here we focus on Pattern Recognition Receptor (PRR) signalling pathways, which play a key role in innate immunity. We used whole-genome resequencing data from a population of bank voles (Myodes glareolus) to test for associations between balancing selection, pleiotropy and gene function in a set of 123 PRR signalling pathway genes. To investigate the effect of gene function, we compared genes encoding (a) receptors for microbial ligands versus downstream signalling proteins, and (b) receptors recognizing components of microbial cell walls, flagella and capsids versus receptors recognizing features of microbial nucleic acids. Analyses based on the nucleotide diversity of full coding sequences showed that balancing selection primarily targeted receptor genes with a low degree of pleiotropy. Moreover, genes encoding receptors recognizing components of microbial cell walls etc. were more important targets of balancing selection than receptors recognizing nucleic acids. Tests for localized signatures of balancing selection in coding and noncoding sequences showed that such signatures were mostly located in introns, and more evenly distributed among different functional categories of PRR pathway genes. The finding that signatures of balancing selection in full coding sequences primarily occur in receptor genes, in particular those encoding receptors for components of microbial cell walls etc., is consistent with the idea that coevolution between hosts and pathogens is an important cause of balancing selection on immune genes. | NA | no | only one group, no good gd or fitness measure | NA | no | no |
| 5290 | 2020 | 44112 | Luplertlop, N; Muangkaew, W; Pumeesat, P; Suwanmanee, S; Singkum, P | 2019 | Distribution of Scedosporium species in soil from areas with high human population density and tourist popularity in six geographic regions in Thailand | Scedosporium is a genus comprising at least 10 species of airborne fungi (saprobes) that survive and grow on decaying organic matter. These fungi are found in high density in human-affected areas such as sewage-contaminated water, and five species, namely Scedosporium apiospermum, S. boydii, S. aurantiacum, S. dehoogii, and S. minutisporum, cause human infections. Thailand is a popular travel destination in the world, with many attractions present in densely populated areas; thus, large numbers of people may be exposed to pathogens present in these areas. We conducted a comprehensive survey of Scedosporium species in 350 soil samples obtained from 35 sites of high human population density and tourist popularity distributed over 23 provinces and six geographic regions of Thailand. Soil suspensions of each sample were inoculated on three plates of Scedo-Select III medium to isolate Scedosporium species. In total, 191 Scedosporium colonies were isolated from four provinces. The species were then identified using PCR and sequencing of the beta-tubulin (BT2) gene. Of the 191 isolates, 188 were S. apiospermum, one was S. dehoogii, and species of two could not be exactly identified. Genetic diversity analysis revealed high haplotype diversity of S. apiospermum. Soil is a major ecological niche for Scedosporium and may contain S. apiospermum populations with high genetic diversity. This study of Scedosporium distribution might encourage health care providers to consider Scedosporium infection in their patients. | NA | no | gd of pathogen | NA | no | no |
| 5291 | 2020 | 44042 | Lv, SL; Zhang, HH; Li, JY; Hu, WQ; Song, YT; Opriessnig, T; Xiao, CT | 2019 | High genetic diversity and recombination events of porcine astrovirus strains identified from ill and asymptomatic pigs in 2017, Hunan Province, China | Astroviruses (AstV) are associated with enteric and systemic disease in mammals and birds. Astroviruses have received increased attention recently as they have been found to be associated with sporadic neurologic disease in mammals including humans. In pigs, porcine astrovirus (PoAstV) can be widely detected and has been grouped in five genotypes (PoAstV1 to PoAstV5). In the present study, we detected multiple PoAstVs in serum samples, nasal swabs, and fecal swabs collected from pigs suffering from respiratory disease or diarrhea but also from asymptomatic pigs, indicating a wide tissue tropism of the identified PoAstV genotypes. Coinfection of different genotypes in the same pig was commonly observed, and within an individual pig a high genetic diversity was observed for viruses belonging to the same PoAstV genotype. Two complete genomes of PoAstV2-WG-R2/2017 and PoAstV4-WG-R2/2017 were successfully obtained and characterized, with genome sizes of 6396 and 6643 nucleotides, respectively. The PoAstV2-WG-R2/2017 genome showed identities of 67.2-77.4% to other known PoAstV2 genomes, and the PoAstV4-WG-R2/2017 genome showed identities of 72.8-80.5% to other known PoAstV4 genomes. The predicted spike domain of open reading frame 2 (ORF2) of these strains showed the highest genetic heterogeneity, with amino acid identities of 13.7-70.9% for PoAstV2-WG-R2/2017 to other known PoAstV2 strains, and identities of 24.4-63.3% for the PoAstV4-WG-R2/2017 to other known PoAstV4 strains. Possible recombination events were identified in each of the two sequences. Two subclades of PoAstV2 and three subclades of PoAstV4 were defined in the present analyses. The obtained data provide further evidence for extraintestinal infectivity of PoAstVs, and confirmed the high genetic diversity of PoAstVs and the coinfection potential of different PoAstV types in a single pig. | NA | no | gd of pathogen | NA | no | no |
| 5292 | 2020 | 44111 | Ma, XM; Jia, CJ; Fu, DH; Chu, M; Ding, XZ; Wu, XY; Guo, X; Pei, J; Bao, PJ; Liang, CN; Yan, P | 2019 | Analysis of Hematological Traits in Polled Yak by Genome-Wide Association Studies Using Individual SNPs and Haplotypes | Yak (Bos grunniens) is an important domestic animal living in high-altitude plateaus. Due to inadequate disease prevention, each year, the yak industry suffers significant economic losses. The identification of causal genes that affect blood- and immunity-related cells could provide preliminary reference guidelines for the prevention of diseases in the population of yaks. The genome-wide association studies (GWASs) utilizing a single-marker or haplotype method were employed to analyze 15 hematological traits in the genome of 315 unrelated yaks. Single-marker GWASs identified a total of 43 significant SNPs, including 35 suggestive and eight genome-wide significant SNPs, associated with nine traits. Haplotype analysis detected nine significant haplotype blocks, including two genome-wide and seven suggestive blocks, associated with seven traits. The study provides data on the genetic variability of hematological traits in the yak. Five essential genes (GPLD1, EDNRA, APOB, HIST1H1E, and HIST1H2BI) were identified, which affect the HCT, HGB, RBC, PDW, PLT, and RDWSD traits and can serve as candidate genes for regulating hematological traits. The results provide a valuable reference to be used in the analysis of blood properties and immune diseases in the yak. | NA | no | dometicated host | NA | no | no |
| 5293 | 2020 | 44040 | Machado, DHB; do Livramento, KG; Maximo, WPF; Negri, BF; Paiva, LV; Valicente, FH | 2020 | Molecular characterization of Bacillus thuringiensis strains to control Spodoptera eridania (Cramer) (Lepidoptera: Noctuidae) population | The main objective of this study was to characterize the toxicity and genetic divergence of 18 Bacillus thuringiensis strains in the biological control of Spodoptera eridania. Bacterial suspensions were added to the S. eridania diet. Half of the selected B. thuringiensis strains caused high mortality seven days after infection. The genetic divergence of B. thuringiensis strains was assessed based on Enterobacterial Repetitive Intergenic Consensus (ERIC) and Repetitive Extragenic Palindromic (REP) sequences, and five phylogenetic groups were formed. Despite their genetic diversity B. thuringiensis strains did not show any correlation between the collection sites and toxicity to larvae. Some B. thuringiensis strains are highly toxic to S. eridania, thus highlighting the potential of their endotoxins as biopesticides. | NA | no | No good fitness measure and measure host GD | NA | no | no |
| 5294 | 2020 | 44042 | Machado, DLM; de Freitas, JA; Luz, JMQ; Maciel, GM; Nogueira, APO; Finzi, RR; Oliveira, RC | 2019 | Genetic divergence between onion populations derived from three different crossing methods | The production of onion hybrids depends on the development of genetically pure lineages. Successive self-fertilization guarantees obtaining endogenous lines quickly. However, onion undergoes a strong process of inbreeding depression when self-fertilized for several generations, which reduces plant vigor, bulb size and seed production, increasing the cost to produce hybrid seed. An estimate of the genetic distance between genotypes is a way of predicting if genetic variability is being maintained. We evaluated possible negative effects in populations due to self-fertilization, compared to interbreeding between two or three plants. Eleven onion populations in different genetic segregation stages, obtained from generations that already had low or moderate inbreeding levels were included. The populations were from the breeding program of Bayer Vegetable Seeds. In order to assess the agronomic descriptors contribution for the genetic divergence, three types of crossing methods of populations were performed: self-fertilization of plants, crossing between two plants and crossings between three plants, during two consecutive years (2014 and 2015). Morphological differences were detected through dissimilarity measures. The traits bulbs/plot (28.32%), fruit fixation (13.12%) and seed weight/umbel (13.41%) together contributed 54.85% to the divergence of onion genotypes. The crosses between two and three plants provided greater divergence among the genotypes, compared to self-fertilization. Economically important traits such as bulb production per plot, fruit fixation, resistance to disease caused by Alternaria porri (Pleosporaceae) and seed weight per umbel can be measured to assess divergence for appropriate selection of onion lineages among segregant populations. | NA | no | inbreeding | NA | no | no |
| 5295 | 2020 | 44040 | Madrenas, R; Balanya, J; Arenas, C; Khadem, M; Mestres, F | 2020 | Global warming and chromosomal inversion adaptation in isolated islands: Drosophila subobscura populations from Madeira | Global warming is an environmental phenomenon to which species must adapt to survive. Drosophila subobscura presents an adaptive capacity due to its chromosomal inversion polymorphism. Until now, the impact of global warming on this polymorphism has been studied in D. subobscura populations located either on a continental mainland or on islands not far from a continent. In this context, gene flow could be a relevant mechanism allowing the movement of thermally adapted inversions between populations. Our aim was to sample and study the chromosomal polymorphism on Madeira, a small isolated island in the Atlantic Ocean. We compared our findings with those reported in the same location approximately four and five decades ago. Moreover, we studied whether global warming has occurred on this island by analyzing mean, maximum and minimum temperatures over a 55-year period. All atmospheric parameters have increased significantly, consistent with climate change expectations. Frequencies and chromosomal thermal index values of thermal adapted inversions remained quite stable over years. Furthermore, J, U and O chromosomes are almost fixed for warm adapted inversions. Thus, if there is little genetic variability remaining and temperatures continue increasing, island populations of D. subobscura might be on the threshold of endangerment. However, apart from selection, genetic drift and inbreeding, other processes, such as phenotypic plasticity or thermoregulatory behavior, could be involved in the survival of the species’ populations. Finally, although in danger, D. subobscura is a generalist that lives in humanized environments, and this fact could favor its persistence on Madeira Island. | NA | no | n | NA | no | no |
| 5296 | 2020 | 44042 | Maebe, K; Haramboure, M; Lucia, M; Alvarez, LJ; Smagghe, G | 2019 | Temporal drop of genetic diversity in Bombus pauloensis | Bumblebees are economically important insects which perform essential pollination tasks in natural and managed ecosystems. Recent research studying Neotropical bumblebee species in Brazil showed a clear decrease in genetic diversity over time in Bombus pauloensis. A new temporal assessment of genetic diversity is needed to know whether this was a location-specific result, or a more general phenomenon. This knowledge is essential to be able to prioritize conservation and management needs. Here, the genetic variability of B. pauloensis populations in Argentina was investigated over time using museum collection specimens from 1933 to 2016, and compared with reanalyzed data from Brazilian populations. Furthermore, specific time series were made for two Argentinean locations, Candelaria and La Plata, and compared with the time series of Porto Alegre (Brazil). All collected specimens were genotyped with 16 microsatellite loci to estimate genetic diversity parameters. Our results showed no drop in either allelic richness or expected heterozygosity over all Argentinean populations. However, a clear drop in genetic diversity was observed in two out of three location-specific time series. This loss of diversity will have negative impacts on population survival, especially over longer periods of time. Furthermore, the use and release of mass-reared specimens of B. pauloensis, which may be inbred and specifically selected for certain commercial but non-adaptive traits, could further diminish the genetic pool. Thus, our result implies the urgent need for regional conservation policies of B. pauloensis in South Brazil and North Argentina. | NA | no | No good fitness measure | NA | no | no |
| 5297 | 2020 | 44041 | Maebe, K; Karise, R; Meeus, I; Mand, M; Smagghe, G | 2019 | Level of Genetic Diversity in European Bumblebees is Not Determined by Local Species Abundance | Bumblebee species with declining population trends tend to show lower genetic diversity levels than stable species. The observed difference might be explained by abundance differences, with declining bumblebee species having lower genetic diversity levels simply due to their lower local species abundances. However, whether this holds true is not known. Here, we investigated whether bumblebee local abundances determines population genetic diversity levels. Therefore, local species abundances were measured for bumblebee species at four locations in Belgium and two locations in Estonia during bumblebee foraging years 2013-2017. These locations and countries were chosen to ensure the greatest possible variance in both local abundances and population trends for these species. Hence, genetic diversity levels were obtained for seven species by genotyping collected specimens with 16 microsatellites. Our results showed that the observed patterns in genetic diversity did not depend on local species abundance. So, although declining bumblebee species can become locally abundant, they will still show lower genetic diversity levels than stable species. This result implies that measuring bumblebees’ local abundance cannot be used to directly determine the health status of a population. Furthermore, this result has also major impact on future conservation strategies as increasing the genetic diversity levels of declining species will be very difficult, and habitat quality should be high to maintain their populations, otherwise these species are doomed to disappear first. | NA | no | No good fitness measure | NA | no | no |
| 5298 | 2020 | 44042 | Magdama, F; Monserrate-Maggi, L; Serrano, L; Sosa, D; Geiser, DM; Jimenez-Gasco, MD | 2019 | Comparative analysis uncovers the limitations of current molecular detection methods for Fusarium oxysporum f. sp. cubense race 4 strains | Fusarium oxysporum f. sp. cubense Tropical Race 4 (Foc TR4) is threatening banana production worldwide. Despite quarantine efforts, the pathogen continues to spread; thus, early diagnosis plays an essential role for the proper execution of contingency plans. Here, we assess the accuracy of four PCR-based molecular methods described in the literature for the identification and detection of race 4 strains, including Subtropical (Foc STR4) and Tropical Race 4 causing Fusarium wilt of banana. We screened a total of 302 isolates using these four markers, and performed phylogenetic analyses, Vegetative Compatibility Group (VCG) testing, sequence comparison, and pathogenicity tests for selected isolates. Our results show that three out of the four markers tested are not reliable for identification of Foc STR4 and TR4, as DNA from isolates from Ecuador, pathogenic and nonpathogenic to banana, obtained from different banana cultivars, displayed cross-reaction with these methods; that is, false positives can occur during the diagnostic process for race 4. Phylogenetic analyses, VCG testing, sequence comparison, and pathogenicity tests suggest the presence of non-target F. oxysporum isolates that share genomic regions with pathogenic strains but lack true pathogenicity to banana. The findings of this work are of foremost importance for international regulatory agencies performing surveillance tests in pathogen-free areas using the current diagnostic methods. We suggest the use of a genetic locus possibly related to virulence, previously identified by T-DNA, and amplified with primers W2987F/W2987R, for diagnosis of Foc TR4 as the most reliable alternative. We urge the adoption of a more holistic view in the study of F. oxysporum as a plant pathogen that considers the biology and diversity of the species for the development of better diagnostic tools. | NA | no | No good fitness measure and measure host GD | NA | no | no |
| 5299 | 2020 | 44113 | Magill, DJ; Kucher, PA; Krylov, VN; Pleteneva, EA; Quinn, JP; Kulakov, LA | 2017 | Localised genetic heterogeneity provides a novel mode of evolution in dsDNA phages | The Red Queen hypothesis posits that antagonistic co-evolution between interacting species results in recurrent natural selection via constant cycles of adaptation and counter-adaptation. Interactions such as these are at their most profound in host-parasite systems, with bacteria and their viruses providing the most intense of battlefields. Studies of bacteriophage evolution thus provide unparalleled insight into the remarkable elasticity of living entities. Here, we report a novel phenomenon underpinning the evolutionary trajectory of a group of dsDNA bacteriophages known as the phiKMVviruses. Employing deep next generation sequencing (NGS) analysis of nucleotide polymorphisms we discovered that this group of viruses generates enhanced intraspecies heterogeneity in their genomes. Our results show the localisation of variants to genes implicated in adsorption processes, as well as variation of the frequency and distribution of SNPs within and between members of the phiKMVviruses. We link error-prone DNA polymerase activity to the generation of variants. Critically, we show trans-activity of this phenomenon (the ability of a phiKMVvirus to dramatically increase genetic variability of a co-infecting phage), highlighting the potential of phages exhibiting such capabilities to influence the evolutionary path of other viruses on a global scale. | NA | no | No good fitness measure and measure host GD | NA | no | no |
| 5300 | 2020 | 44113 | Mahakalkar, AL; Sapkal, HP; Baig, MM | 2017 | Report of high genetic diversity of filarial worm, Wuchereria bancrofti from endemic region of Eastern Maharashtra (India) | Filariasis free India is National Health Policy’s vision. Currently, India has undertaken Mass Drug Administration drive (MDA) to eliminate the filariasis infection. Taking this into account, the emergence of new Wuchereria bancrofti resistant strains against the current choice of drugs cannot be ruled out. In this study, we report the genetic diversity of Wuchereria bancrofti from four districts (Amravati, Nagpur, Chandrapur and Wardha) of Vidarbha, a region in the eastern part of Maharashtra state of India and considered a hot spot of filariasis infections. The RAPD profiles were generated for 21 populations using nine random RAPD primers. The RAPD-PCR based distance matrix shows maximum genetic distance of 0.425 between the parasites from Nagpur and Amravati region and minimum genetic distance of 0.210 between the parasites of Wardha and Nagpur. The tree inferred by Neighbour-Joining (NJ) method shows four distinct clusters. With the single exception of isolates from Amravati, all other clusters show the intermingling of isolates with other districts. Further, the representation of isolates from Chandrapur in three out of four clusters revealed to be a founder and indicates towards the entry of the filarial worm into the Maharashtra state through southern route. | NA | no | gd of pathogen | NA | no | no |
| 5301 | 2020 | 44112 | Mahomed, TG; Kock, MM; Masekela, R; Hoosien, E; Ehlers, MM | 2018 | Genetic relatedness of Staphylococcus aureus isolates obtained from cystic fibrosis patients at a tertiary academic hospital in Pretoria, South Africa | Cystic fibrosis (CF) is an inherited recessive disease that affects mucocillary clearance in the lung, allowing it to be colonised with bacteria such as Staphylococcus aureus. To survive in the CF lung S. aureus adapts both phenotypically and genotypically, through various mechanisms. In this study, multiple specimens were collected from the participants and were processed routinely and were additionally cultured in chromogenic media. Multiplex PCR assays were employed to detect methicillin resistance and selected virulence and quaternary ammonium compound (qac) genes. Genetic relatedness of the S. aureus was determined using agr, SCCmec and spa typing as well as pulsed field gel electrophoresis (PFGE) and multi-locus sequence typing (MLST). Thirty-three S. aureus isolates were isolated, of which 51% (17/33) were methicillin resistant S. aureus (MRSA). The virulence and qac genes were more prevalent in MRSA than the methicillin sensitive S. aureus (MSSA) isolates. The PFGE analysis showed nine distinct pulsotypes while MLST showed eight sequence types. All the STs detected in this study, except for ST508 have been previously isolated from CF patients according to the literature. This study showed a genetically diverse S. aureus population with a high prevalence of virulence genes among the MRSA isolates from the CF clinic. | NA | no | gd of pathogen | NA | no | no |
| 5302 | 2020 | 44041 | Maibach, V; Langergraber, K; Leendertz, FH; Wittig, OM; Vigilant, L | 2019 | Differences in MHC-B diversity and KIR epitopes in two populations of wild chimpanzees | The major histocompatibility complex (MHC) class I genes play a critical role within the immune system, both by the presentation of antigens from intracellular pathogens to immunocompetent cells and by the interaction with killer cell immunoglobulin-like receptors (KIR) on natural killer cells (NK cells). Genes of the MHC are highly diverse, and MHC variation can have effects on the immune functionality of individuals; hence, comparisons of MHC diversity among closely related phylogenetic taxa may give insight into the factors responsible for the shaping of its diversity. The four geographically separated chimpanzee subspecies differ in their overall genetic diversity, have different population histories, and are confronted with different pathogens in their natural habitat, all of which may affect MHC class I DNA sequence diversity. Here, we compare the MHC-B exon two DNA sequence diversity from 24 wild western and 46 wild eastern chimpanzees using necropsy and noninvasively collected fecal samples, respectively. We found a higher MHC-B exon two nucleotide diversity, in our western than eastern chimpanzees. The inclusion of previously published MHC-B exon two data from other western and eastern chimpanzees supported this finding. In addition, our results confirm and extend the finding of a very low C1 epitope frequency at eastern chimpanzee MHC-B molecules, which likely affects the ability of these molecules to interact with NK cells. While the understanding of the differing pathogen environments encountered by disparate populations of a species is a challenging endeavor, these findings highlight the potential for these pathogens to selectively shape immune system variation. | NA | no | only two pop | NA | no | no |
| 5303 | 2020 | 44112 | Maibach, V; Vigilant, L | 2019 | Reduced bonobo MHC class I diversity predicts a reduced viral peptide binding ability compared to chimpanzees | BackgroundThe highly polymorphic genes of the major histocompatibility complex (MHC) class I are involved in defense against viruses and other intracellular pathogens. Although several studies found reduced MHC class I diversity in bonobos in comparison to the closely related chimpanzee, it is unclear if this lower diversity also influences the functional ability of MHC class I molecules in bonobos. Here, we use a bioinformatic approach to analyze the viral peptide binding ability of all published bonobo MHC class I molecules (n=58) in comparison to all published chimpanzee MHC class I molecules (n=161) for the class I loci A, B, C and A-like.ResultsWe examined the peptide binding ability of all 219 different MHC class I molecules to 5,788,712 peptides derived from 1432 different primate viruses and analyzed the percentage of bound peptides and the overlap of the peptide binding repertoires of the two species. We conducted multiple levels of analysis on the species-, population- and individual-level to account for the characterization of MHC variation in a larger number of chimpanzees and their broader geographic distribution. We found a lower percentage of bound peptides in bonobos at the B locus in the population-level comparison and at the B and C loci in the individual-level comparison. Furthermore, we found evidence of a limited peptide binding repertoire in bonobos by tree-based visualization of functional clustering of MHC molecules, as well as an analysis of peptides bound by both species.ConclusionOur results suggest a reduced MHC class I viral peptide binding ability at the B and C loci in bonobos compared to chimpanzees. The effects of this finding on the immune defense against viruses in wild living bonobos are unclear. However, special caution is needed to prevent introduction and spread of new viruses to bonobos, as their defensive ability to cope with new viruses could be limited compared to chimpanzees. | NA | no | no good gd measure host pop | NA | no | no |
| 5304 | 2020 | 44041 | Maktabi, S; Ghorbanpoor, M; Hossaini, M; Motavalibashi, A | 2019 | Detection of multi-antibiotic resistant Campylobacter coli and Campylobacter jejuni in beef, mutton, chicken and water buffalo meat in Ahvaz, Iran | Campylobacter jejuni and C. coli are the main causes of gastrointestinal diseases in humans even in industrialized countries affecting public health. The aim of the current study was to evaluate the occurrence and antibiotic resistance of C. jejuni and C. coli in chicken meat, beef, mutton and water buffalo meat slaughtered in Ahvaz city, Iran. A total of 380 samples including chicken meat from industrial abattoirs (n = 150), chicken meat from traditional abattoirs (n = 50), fresh packed chicken meat from local markets (n = 30) and beef, mutton and water buffalo meat from industrial abattoirs (50 samples for each meat) in Ahvaz, were collected and tested for the presence of Campylobacter spp. The procedure was one-step enrichment in Preston enrichment broth followed by plating on supplemented blood agar for 24 hr under microaerophilic conditions at 42 degrees C. Suspected colonies were tested by polymerase chain reaction assay and susceptibility of the confirmed isolates to various antibiotics was investigated by the Kirby-Bauer disk diffusion method. Overall, 32 samples (8.40%) were contaminated with Campylobacter spp. Mutton was the most contaminated meat (24%), while fresh packed chicken meat were not contaminated. Among the 32 isolates, 40.60%, 34.40%, 21.90%, and 15.60% were resistant to tetracycline, ciprofloxacin, ampicillin, and streptomycin, respectively. Moreover, a high number of multi-antibiotic resistant Campylobacter spp. was determined. Since foods of animal origin are the most sources of Campylobacter infection, the presence of resistant strains to antibiotics is a potential risk to public health. (C) 2019 Urmia University. All rights reserved. | NA | no | No good fitness measure and measure host GD | NA | no | no |
| 5305 | 2020 | 44111 | Manangwa, O; De Meeus, T; Grebaut, P; Segard, A; Byamungu, M; Ravel, S | 2019 | Detecting Wahlund effects together with amplification problems: Cryptic species, null alleles and short allele dominance in Glossina pallidipes populations from Tanzania | Population genetics is a convenient tool to study the population biology of non-model and hard to sample species. This is particularly true for parasites and vectors. Heterozygote deficits and/or linkage disequilibrium often occur in such studies and detecting the origin of those (Wahlund effect, reproductive system or amplification problems) is uneasy. We used new tools (correlation between the number of times a locus is found in significant linkage disequilibrium and its genetic diversity, correlations between Wright’s F-IS and F-ST, F-IS and number of missing data, F-IT and allele size and standard errors comparisons) for the first time on a real data set of tsetse flies, a vector of dangerous diseases to humans and domestic animals in sub-Saharan Africa. With these new tools, and cleaning data from null allele, temporal heterogeneity and short allele dominance effects, we unveiled the coexistence of two highly divergent cryptic clades in the same sites. These results are in line with other studies suggesting that the biodiversity of many taxa still largely remain undescribed, in particular pathogenic agents and their vectors. Our results also advocate that including individuals from different cohorts tends to bias subdivision measures and that keeping loci with short allele dominance and/or too frequent missing data seriously jeopardize parameter’s estimations. Finally, separated analyses of the two clades suggest very small tsetse densities and relatively large dispersal. | NA | no | No good fitness measure and measure host GD | NA | no | no |
| 5306 | 2020 | 44113 | Manawasinghe, IS; Zhang, W; Li, XH; Zhao, WS; Chethana, KWT; Xu, JP; Chen, Z; Dissanayaka, AJ; Mugnai, L; Urbez-Torres, JR; Savocchia, S; Hyde, KD; Yan, JY | 2018 | Novel microsatellite markers reveal multiple origins of Botryosphaeria dothidea causing the Chinese grapevine trunk disease | Botryosphaeria dothidea causes diseases in many different hosts worldwide. This species has become the most prominent fungal taxon causing grapevine trunk disease in China, with a recent disease outbreak. To reveal the genetic diversity and explore the origin of B. dothidea, six novel microsatellite markers were developed and used for the genotyping of 151 isolates obtained from China and overseas. The results demonstrated the high genetic diversity of the B. dothidea populations. Bayesian cluster analysis separated the total B. dothidea isolates into five genetic populations. B. dothidea isolates from Chinese grapevines were observed to share alleles with isolates from different hosts within China and from grapevines growing overseas, indicating both endemic host shifts and exotic introduction. In addition, unique pathogen genotypes were identified in Chinese grapevine isolates. Hence, we infer that B. dothidea isolates from multiple origins are contributing to the dieback and canker outbreak currently occurring in China. (C) 2018 The Authors. Published by Elsevier Ltd. | NA | no | No good fitness measure and measure host GD | NA | no | no |
| 5307 | 2020 | 44111 | Manlik, O; Krutzen, M; Kopps, AM; Mann, J; Bejder, L; Allen, SJ; Frere, C; Connor, RC; Sherwin, WB | 2019 | Is MHC diversity a better marker for conservation than neutral genetic diversity? A case study of two contrasting dolphin populations | Genetic diversity is essential for populations to adapt to changing environments. Measures of genetic diversity are often based on selectively neutral markers, such as microsatellites. Genetic diversity to guide conservation management, however, is better reflected by adaptive markers, including genes of the major histocompatibility complex (MHC). Our aim was to assess MHC and neutral genetic diversity in two contrasting bottlenose dolphin (Tursiops aduncus) populations in Western Australia-one apparently viable population with high reproductive output (Shark Bay) and one with lower reproductive output that was forecast to decline (Bunbury). We assessed genetic variation in the two populations by sequencing the MHC class II DQB, which encompasses the functionally important peptide binding regions (PBR). Neutral genetic diversity was assessed by genotyping twenty-three microsatellite loci. We confirmed that MHC is an adaptive marker in both populations. Overall, the Shark Bay population exhibited greater MHC diversity than the Bunbury population-for example, it displayed greater MHC nucleotide diversity. In contrast, the difference in microsatellite diversity between the two populations was comparatively low. Our findings are consistent with the hypothesis that viable populations typically display greater genetic diversity than less viable populations. The results also suggest that MHC variation is more closely associated with population viability than neutral genetic variation. Although the inferences from our findings are limited, because we only compared two populations, our results add to a growing number of studies that highlight the usefulness of MHC as a potentially suitable genetic marker for animal conservation. The Shark Bay population, which carries greater adaptive genetic diversity than the Bunbury population, is thus likely more robust to natural or human-induced changes to the coastal ecosystem it inhabits. | NA | no | No good fitness measure | NA | no | no |
| 5308 | 2020 | 44041 | Mansoor, S; Ahmed, N; Sharma, V; Jan, S; Nabi, SU; Mir, JI; Mir, MA; Masoodi, KZ | 2019 | Elucidating genetic variability and population structure in Venturia inaequalis associated with apple scab diseaseusing SSR markers | Apple scab caused by Venturia inaequalis Cooke (Wint.) is one the important diseases of trade and industrial significance in apple. In present study variability studies in pathogen isolates were studied, which is one of the most important factors for devising management studies of scab disease in apple. Genetic diversity of 30 Venturia inaequalis isolates from 12 districts of two geographical distinct regions of Jammu and Kashmir was calculated based on the allele frequencies of 28 SSR markers and the internal transcribed spacer (ITS) region of the ribosomal DNA. The ITS based characterized sequences were submitted to NCBI Gen Bank and accession numbers were sanctioned. Dendrogram showed that all the accessions formed 2 main clusters with various degree of sub clustering within the clusters. Analysis based on SSR study reveals that the heterozygosity ranged from 0.0 and 0.5, with an average value of 0.39. The expected heterozygosis or gene diversity (He) ranged from 0.0 to 0.50 with an average of 0.40. The Fst value ranges from 0 to 0.6 with an average of 0.194. Diversity within each population (HS) values ranging from 0.26 to 0.33. Average differentiation among populations (GST) was 0.11 and populations were isolated by significant distance (r(2) = 0.50, P < 0.01). From the AMOVA analysis, 25% of variation was observed among population, 9% among individuals and 66% within individuals observed in the population. Structure analysis grouped isolates into two populations. Principle coordinate analysis explained variation of 36.6% in population 1, 14.30% in population 2 and 13.10% in population 3(Admixture) with 64.07% as overall cumulative percentage of variation. This indicates that extensive short-distance gene flow occurs in Kashmir region that dispersal over longer distances also appears to occur frequently enough to prevent differentiation due to genetic drift. Also it is evident that Jammu and Kashmir most likely has V. inaequalissubpopulations linked to diverse climatic conditions of the Jammu region compared to the mountainous inland Kashmir region. The results of present study would help to understand the genetic diversity of V. inaequalis from Jammu and Kashmir that would lead in the development of more effective management strategies and development of new resistant cultivars through marker-assisted selection. | NA | no | gd of pathogen | NA | no | no |
| 5309 | 2020 | 44112 | Marchi, N; Mennecier, P; Georges, M; Lafosse, S; Hegay, T; Dorzhu, C; Chichlo, B; Segurel, L; Heyer, E | 2018 | Close inbreeding and low genetic diversity in Inner Asian human populations despite geographical exogamy | When closely related individuals mate, they produce inbred offspring, which often have lower fitness than outbred ones. Geographical exogamy, by favouring matings between distant individuals, is thought to be an inbreeding avoidance mechanism; however, no data has clearly tested this prediction. Here, we took advantage of the diversity of matrimonial systems in humans to explore the impact of geographical exogamy on genetic diversity and inbreeding. We collected ethno-demographic data for 1,344 individuals in 16 populations from two Inner Asian cultural groups with contrasting dispersal behaviours (Turko-Mongols and Indo-Iranians) and genotyped genome-wide single nucleotide polymorphisms in 503 individuals. We estimated the population exogamy rate and confirmed the expected dispersal differences: Turko-Mongols are geographically more exogamous than Indo-Iranians. Unexpectedly, across populations, exogamy patterns correlated neither with the proportion of inbred individuals nor with their genetic diversity. Even more surprisingly, among Turko-Mongols, descendants from exogamous couples were significantly more inbred than descendants from endogamous couples, except for large distances (>40 km). Overall, 37% of the descendants from exogamous couples were closely inbred. This suggests that in Inner Asia, geographical exogamy is neither efficient in increasing genetic diversity nor in avoiding inbreeding, which might be due to kinship endogamy despite the occurrence of dispersal. | NA | no | human | NA | no | no |
| 5310 | 2020 | 44113 | Marco-Jimenez, F; Baselga, M; Vicente, JS | 2018 | Successful re-establishment of a rabbit population from embryos vitrified 15 years ago: The importance of biobanks in livestock conservation | Genetic resource banks (GRB) are a valuable tool for maintaining genetic variability and preserving breeds from pathogens or catastrophe, enabling us to assess and correct breeding schemes, minimizing the impact of genetic drift and facilitating diffusion. This study tests their efficiency in re-establishing two extinct populations of a synthetic rabbit line selected for daily weight gain, using vitrified embryos from two generations (18th and 36th) separated by 15 years of genetic selection. The effect of long-term storage of vitrified embryos in liquid nitrogen was also evaluated. A total of 516 vitrified embryos using the same protocol were transferred into 54 recipients. The embryos had been maintained in liquid nitrogen during 2 different periods, (i) 1 year (301 embryos and 26 transfers, 36th generation) and (ii) 15 years (259 embryos and 28 transfers, 18th generation). A total of 80.0% (8/10 to 18th) and 60.0% (9/15 to 36th) of the foundational sire families were eventually re-established. Over approximately one year, animals within each population were crossed to produce the next generation and re-establish the original population size. Our study demonstrated that our GRB of embryos vitrified 15 years ago is a successful strategy to re-establish rabbit populations to continue the breeding programme. | NA | no | No good fitness measure and measure host GD | NA | no | no |
| 5311 | 2020 | 44112 | Margres, MJ; Jones, ME; Epstein, B; Kerlin, DH; Comte, S; Fox, S; Fraik, AK; Hendricks, SA; Huxtable, S; Lachish, S; Lazenby, B; O’Rourke, SM; Stahlke, AR; Wiench, CG; Hamede, R; Schonfeld, B; McCallum, H; Miller, MR; Hohenlohe, PA; Storfer, A | 2018 | Large-effect loci affect survival in Tasmanian devils (Sarcophilus harrisii) infected with a transmissible cancer | Identifying the genetic architecture of complex phenotypes is a central goal of modern biology, particularly for disease-related traits. Genome-wide association methods are a classical approach for identifying the genomic basis of variation in disease phenotypes, but such analyses are particularly challenging in natural populations due to sample size difficulties. Extensive mark-recapture data, strong linkage disequilibrium and a lethal transmissible cancer make the Tasmanian devil (Sarcophilus harrisii) an ideal model for such an association study. We used a RAD-capture approach to genotype 624 devils at similar to 16,000 loci and then used association analyses to assess the heritability of three cancer-related phenotypes: infection case-control (where cases were infected devils and controls were devils that were never infected), age of first infection and survival following infection. The SNP array explained much of the phenotypic variance for female survival (>80%) and female case-control (>61%). We found that a few large-effect SNPs explained much of the variance for female survival (similar to 5 SNPs explained >61% of the total variance), whereas more SNPs (similar to 56) of smaller effect explained less of the variance for female case-control (similar to 23% of the total variance). By contrast, these same SNPs did not account for a significant proportion of phenotypic variance in males, suggesting that the genetic bases of these traits and/or selection differ across sexes. Loci involved with cell adhesion and cell-cycle regulation underlay trait variation, suggesting that the devil immune system is rapidly evolving to recognize and potentially suppress cancer growth through these pathways. Overall, our study provided necessary data for genomics-based conservation and management in Tasmanian devils. | NA | no | no good gd measure host pop | NA | no | no |
| 5312 | 2020 | 44112 | Margres, MJ; Ruiz-Aravena, M; Hamede, R; Jones, ME; Lawrance, MF; Hendricks, SA; Patton, A; Davis, BW; Ostrander, EA; McCallum, H; Hohenlohe, PA; Storfer, A | 2018 | The Genomic Basis of Tumor Regression in Tasmanian Devils (Sarcophilus harrisii) | Understanding the genetic basis of disease-related phenotypes, such as cancer susceptibility, is crucial for the advancement of personalized medicine. Although most cancers are somatic in origin, a small number of transmissible cancers have been documented. Two such cancers have emerged in the Tasmanian devil (Sarcophilus harrisii) and now threaten the species with extinction. Recently, cases of natural tumor regression in Tasmanian devils infected with the clonally contagious cancer have been detected. We used whole-genome sequencing and F-ST-based approaches to identify the genetic basis of tumor regression by comparing the genomes of seven individuals that underwent tumor regression with those of three infected individuals that did not. We found three highly differentiated candidate genomic regions containing several genes related to immune response and/or cancer risk, indicating that the genomic basis of tumor regression was polygenic. Within these genomic regions, we identified putative regulatory variation in candidate genes but no nonsynonymous variation, suggesting that natural tumor regression may be driven, at least in part, by differential host expression of key loci. Comparative oncology can provide insight into the genetic basis of cancer risk, tumor development, and the pathogenicity of cancer, particularly due to our limited ability to monitor natural, untreated tumor progression in human patients. Our results support the hypothesis that host immune response is necessary for triggering tumor regression, providing candidate genes that may translate to novel treatments in human and nonhuman cancers. | NA | no | No good fitness measure and measure host GD | NA | no | no |
| 5313 | 2020 | 44042 | Marquez-Chavez, A; Guzman-Franco, AW; Santillan-Galicia, MT; Tamayo-Mejia, F; Rodriguez-Maciel, JC | 2019 | Effect of host plant on the genetic diversity of Tetranychus urticae Koch populations and their susceptibility to fungal infection | We studied the effect of host plant on the genetic population structure of Tetranychus urticae Koch populations and their susceptibility to fungal infection. We sampled adult T. urticae from ten locations, comprising six Rubus ulmifolius Schoot (blackberry) orchards and four R. idaeus L. (raspberry) orchards. Using sequence information from a fragment of the mitochondrial cytochrome oxidase subunit I (COI), phylogenetic relationships and genetic population structure of each group were determined using haplotype network analyses and an analysis of molecular variance (AMOVA). Based on these results, four populations were selected to compare their susceptibility to isolates of Beauveria bassiana (Bals.-Criv.) Vuill., Metnrhizium anisopliae (Metschn.) Sorokin, Lecanicillium lecanii (Zimm.) Zare & W. Gams and Isaria fumosorosea Wise. Two populations from raspberry (var. ‘7-UDV’ and var. ‘8-UDV’) and two from blackberry (var. ‘Tupy’ and var. ‘2-UDV’) were studied. Haplotype network analysis identified eight haplotypes. The greatest genetic diversity was found in the ‘7-UDV’ raspberry population which had five haplotypes; the remaining populations had one or two haplotypes each. The AMOVA analysis showed that neither host plant nor geographical origin explained all the genetic variation. The ‘2-UDV’ population was the most susceptible to fungal infection, followed by ‘Tupy’, both of which were from blackberry. The fungal isolates that caused greatest mortality in T. urticae were M. anisopline and B. bassiana. In the least susceptible populations (‘7-UDV’ and ‘8-UDV’, both from raspberry), all isolates still caused some mortality, but there were no differences amongst the isolates. Our results suggest that host plant plays a role in the susceptibility of T. urticae to fungal infection, but not in their genetic population structure. | NA | no | gd of pathogen | NA | no | no |
| 5314 | 2020 | 44112 | Marrero, MV; Oostermeijer, G; Nogales, M; Van Hengstum, T; Saro, I; Carque, E; Sosa, PA; Banares, A | 2019 | Comprehensive population viability study of a rare endemic shrub from the high mountain zone of the Canary Islands and its conservation implications | Oceanic island ecosystems harbour many endemic plant and animal species, which are often threatened because they have only a few small populations. Many factors contribute to the biological viability of such populations, such as demography and population dynamics, breeding system and pollination ecology, seed dispersal and genetic variation. In a collaborative project, all these factors were studied in the rare endemic, predominantly monoecious shrub Bencomia exstipulata Svent. (Rosaceae), which grows exclusively in the national parks of El Teide (Tenerife) and La Caldera de Taburiente (La Palma). Demography was monitored through annual censuses of individual plants in a natural and an augmented population on Tenerife. The breeding system and reproductive success were studied through bagging and pollination experiments, and insect visitation censuses. Seed dispersal by animals was assessed using cafetaria experiments. With matrix projection models and stochastic simulations, we show that the Tenerife population was demographically stable. This was largely explainable by the high survival of adult individuals. Despite frequent germination, successful seedling recruitment was very rare. Male and female flowers occurred in separate inflorescences within individuals, although some inflorescences were mixed and some shrubs were entirely male or female. Despite frequent visits by honeybees, the species is predominantly wind pollinated. Insect-proof bags reduced seed set by 12.5%, and pollen-proof bags by 44%. Large quantities of airborne pollen were detected on unbagged sticky microscope slides, this was 56% reduced by insect-proof and 96% by pollen-proof bags. Hence, some self-pollination also seems to occur. Cafetaria experiments showed that the local lizards (Gallotia galloti Oudart) readily eat the fruits and that the seeds pass through their intestines unharmed and germinable. Since other dispersal vectors are unknown, saurochory seems the most likely mode of dispersal. Our study strongly suggests that the population of B. exstipulata on Tenerife is viable, and that there are no significant threats associated with its breeding system, pollination or seed dispersal. To alleviate the natural extinction risk typical of narrow endemics, five main conservation measures are proposed. | NA | no | No good fitness measure and measure host GD | NA | no | no |
| 5315 | 2020 | 44111 | Marshall, TL; Baca, CR; Correa, DT; Forstner, MRJ; Hahn, D; Rodriguez, D | 2019 | Genetic characterization of chytrids isolated from larval amphibians collected in central and east Texas | Chytridiomycosis, an emerging infectious disease caused by the fungal pathogen Batrachochytrium dendrobatidis (Bd), has caused amphibian population declines worldwide. Bd was first described in the 1990s and there are still geographic gaps in the genetic analysis of this globally distributed pathogen. Relatively few genetic studies have focused on regions where Bd exhibits low virulence, potentially creating a bias in our current knowledge of the pathogen’s genetic diversity. Disease-associated declines have not been recorded in Texas (USA), yet Bd has been detected on amphibians in the state. These strains have not been isolated and characterized genetically; therefore, we isolated, cultured, and genotyped Bd from central Texas and compared isolates to a panel of previously genotyped strains distributed across the Western Hemisphere. We also isolated other chytrids from east Texas not known to infect amphibians. To identify larval amphibian hosts, we sequenced part of the COI gene. Among 37 Bd isolates from Texas, we detected 19 unique multi-locus genotypes, but found no genetic structure associated with host species, Texas localities, or across North America. Isolates from central Texas exhibit high diversity and genetically cluster with BdGPL isolates from the western U.S. that have caused amphibian population declines. This study genetically characterizes isolates of Bd from the south central U.S. and adds to the global knowledge of Bd genotypes. (C) 2018 Elsevier Ltd and British Mycological Society. All rights reserved. | NA | no | No good fitness measure and measure host GD | NA | no | no |
| 5316 | 2020 | 44113 | Martin-Torrijos, L; Kawai, T; Makkonen, J; Jussila, J; Kokko, H; Dieguez-Uribeondo, J | 2018 | Crayfish plague in Japan: A real threat to the endemic Cambaroides japonicus | Global introductions of aquatic species and their associated pathogens are threatening worldwide biodiversity. The introduction of two North American crayfish species, Procambarus clarkiiand Pacifastacus leniusculus, into Japan in 1927 seems to have negatively affected native Japanese crayfish populations of Cambaroides japonicus. Several studies have shown the decline of these native populations due to competition, predation and habitat colonization by the two invasive North American crayfish species. Here, we identify an additional factor contributing to this decline. We report the first crayfish plague outbreaks in C. japonicus populations in Japan, which were diagnosed using both histological and molecular approaches (analyses of the internal transcribed spacer region). Subsequent analyses of the mitochondrial ribosomal rnnS and rnnL regions of diseased specimens indicate that these outbreaks originated from a P. clarkii population and identify a novel haplotype of Aphanomyces astaci, d3-haplotype, hosted by P. clarkii. Overall, our findings demonstrate the first two cases of crayfish plague in Japan, and the first case in a non-European native crayfish species, which originated from the red swamp crayfish P. clarkii. This finding is a matter of concern for the conservation of the native freshwater species of Japan and also highlights the risk of introducing crayfish carrier species into biogeographic regions harboring species susceptible to the crayfish plague. | NA | no | No good fitness measure and measure host GD | NA | no | no |
| 5317 | 2020 | 44112 | Martin, FN; Zhang, YH; Cooke, DEL; Coffey, MD; Grunwald, NJ; Fry, WE | 2019 | Insights into evolving global populations of Phytophthora infestans via new complementary mtDNA haplotype markers and nuclear SSRs | In many parts of the world the damaging potato late blight pathogen, Phytophthora infestans, is spread as a succession of clonal lineages. The discrimination of genetic diversity within such evolving populations provides insights into the processes generating novel lineages and the pathways and drivers of pathogen evolution and dissemination at local and global scales. This knowledge, in turn, helps optimise management practices. Here we combine two key methods for dissecting mitochondrial and nuclear diversity and resolve intra and inter-lineage diversity of over 100 P. infestans isolates representative of key clonal lineages found globally. A novel set of PCR primers that amplify five target regions are provided for mitochondrial DNA sequence analysis. These five loci increased the number of mtDNA haplotypes resolved from four with the PCR RFLP method to 37 (17, 6, 8 and 4 for Ia, Ib, IIa, and IIb haplotypes, respectively, plus 2 Herb-1 haplotypes). As with the PCR RFLP method, two main lineages, I and II were defined. Group I contained 25 mtDNA haplotypes that grouped broadly according to the Ia and Ib types and resolved several sub-clades amongst the global sample. Group II comprised two distinct clusters with four haplotypes corresponding to the RFLP type IIb and eight haplotypes resolved within type IIa. The 12-plex SSR assay revealed 90 multilocus genotypes providing accurate discrimination of dominant clonal lineages and other genetically diverse isolates. Some association of genetic diversity and geographic region of contemporary isolates was observed; US and Mexican isolates formed a loose grouping, distinct from isolates from Europe, South America and other regions. Diversity within clonal lineages was observed that varied according to the age of the clone. In combination, these fine-scale nuclear and maternally inherited mitochondrial markers enabled a greater level of discrimination among isolates than previously available and provided complementary perspectives on evolutionary questions relating to the diversity, phylogeography and the origins and spread of clonal lineages of P. infestans. | NA | no | No good fitness measure | NA | no | no |
| 5318 | 2020 | 44113 | Martinez, JG; Mira, O; Sanchez-Prieto, CB; Barea-Azcon, JM; Tinaut, A | 2018 | Population size and genetic variability of a relict population of an endangered butterfly, Parnassius apollo filabricus | Parnassius apollo filabricus is a subspecies of apollo (Lepidoptera, Papilionidae) restricted to the Sierra de Baza-Filabres range in southeastern Spain that has become increasingly rare in the last decades, disappearing from most of its known locations. In this article, we calculate both census and effective population size of a local population discovered in 2009 that occupies c. 30ha. After 2years of capture-mark-recapture work we estimate a population size of about 100 individuals. Genetic variation was characterised using 9 microsatellite markers and 29 individuals. Effective population size was estimated from 13 microsatellites. The studied population is strongly differentiated from the nearby Sierra Nevada apollo populations, and its expected heterozygosity and allelic richness were higher than the average value for Sierra Nevada. Genetic diversity of the population is not as low as expected by its small size, which points out to a recent population decline. We discuss the implications of these results for the conservation of the species. | NA | no | No good fitness measure and measure host GD | NA | no | no |
| 5319 | 2020 | 44112 | Martinu, J; Hypsa, V; Stefka, J | 2018 | Host specificity driving genetic structure and diversity in ectoparasite populations: Coevolutionary patterns in Apodemus mice and their lice | A degree of host specificity, manifested by the processes of host-parasite cospeciations and host switches, is assumed to be a major determinant of parasites’ evolution. To understand these patterns and formulate appropriate ecological hypotheses, we need better insight into the coevolutionary processes at the intraspecific level, including the maintenance of genetic diversity and population structure of parasites and their hosts. Here, we address these questions by analyzing large-scale molecular data on the louse Polyplax serrata and its hosts, mice of the genus Apodemus, across a broad range of European localities. Using mitochondrial DNA sequences and microsatellite data, we demonstrate the general genetic correspondence of the Apodemus/Polyplax system to the scenario of the postglacial recolonization of Europe, but we also show several striking discrepancies. Among the most interesting are the evolution of different degrees of host specificity in closely related louse lineages in sympatry, or decoupled population structures of the host and parasites in central Europe. We also find strong support for the prediction that parasites with narrower host specificity possess a lower level of genetic diversity and a deeper pattern of interpopulation structure as a result of limited dispersal and smaller effective population size. | NA | no | No good fitness measure and measure host GD | NA | no | no |
| 5320 | 2020 | 44113 | Masoni, A; Frizzi, F; Mattioli, M; Turillazzi, S; Ciofi, C; Santini, G | 2017 | Pleometrotic colony foundation in the ant Crematogaster scutellaris (Hymenoptera: Formicidae): better be alone than in bad company | In this study, we investigated whether pleometrosis occurs in Crematogaster scutellaris (OLIVIER, 1792) and which advantages it may provide. A survey of C. scutellaris queens in aphid galls revealed that 19% of the occupied galls contained two or more queens (max. five). Moreover, the observed distribution of the number of queens per gall was not statistically different from that expected from a null model based on random allocation. Microsatellite analysis on naturally associated queens showed no evidence of relatedness among them. We then investigated the possible advantages of pleometrotic foundation, comparing the survival and brood production of queens spontaneously nesting alone or in pairs, in small and large nests. Paired queens never showed any form of cooperation in brood care and interacted aggressively or tried to avoid each other. Mortality of paired queens was always greater than that of solitary queens, and mortality was higher in small than large nests. Finally, the larger queen in a pair was more likely to survive the fights. The total number of eggs produced by queens in pairs was nearly twice that produced by isolated queens, although the total progeny production (larvae, pupae, and workers) was the same. This suggests significantly higher brood attrition in paired queens than in solitary ones, probably due to an increase in egg cannibalism or trophic egg production. Further, the time until first worker eclosion was the same for pleometrotic and haplometrotic queens. These results suggest that associative foundation in C. scutellaris is more likely to be the result of a random process, probably triggered by the need to escape from predation and abiotic stresses after the nuptial flight, than an active choice of queens. Additionally, the possible advantages of associative foundation, if any, should be very limited. | NA | no | no good gd measure host pop | NA | no | no |
| 5321 | 2020 | 44113 | Masry, A; Furlong, MJ; Clarke, AR; Cunningham, JP | 2018 | An improved culturing method for opiine fruit fly parasitoids and its application to parasitoid monitoring in the field | Good culturing methods play an important role in the study of insect behavior and its application to pest management. Here, we describe and validate a new method for rearing the parasitoid wasp, Diachasmimorpha kraussii, which attacks some of the world’s worst fruit fly pests and is an internationally used biological control agent. Our method differs from standard culturing approaches by presenting adult wasps with host-infested artificial media within a culturing bag, which mimics a natural (fruit) oviposition substrate. In laboratory trials using wild collectedD. kraussii, the culturing bag method was compared to the use of host-infested nectarines, and a commonly used laboratory method of presenting host-infested artificial media within Petri dishes. The culturing bag method proved to be a significant improvement on both methods, combining the advantages of high host survival in artificial media with parasitism levels that were the equivalent to those recorded using host-infested fruits. In our field study, culturing bags infested with the Queensland fruit fly, Bactrocera tryoni, and hung in a mixed peach and nectarine orchard proved to be effective artificial fruits attracting wild D. kraussii for oviposition. Significantly more adult wasps were reared from the culturing bags compared to field collected fruits. This was shown to be due to higher fruit fly larval density in the bags, as similar percentage parasitism rates were found between the culturing bags and ripe fruits. We discuss how this cheap, time-efficient method could be applied to collecting and monitoring wild D. kraussii populations in orchards, and assist in maintaining genetic variability in parasitoid laboratory cultures. | NA | no | No good fitness measure and measure host GD | NA | no | no |
| 5322 | 2020 | 44112 | Matias, AMA; Riginos, C | 2018 | Revisiting the Centre Hypotheses of the Indo-West Pacific: Idiosyncratic genetic diversity of nine reef species offers weak support for the Coral Triangle as a centre of genetic biodiversity | AimSeveral biogeographic hypotheses seek to explain the extreme species diversity of the coral reefs in the Coral Triangle (CT) of the Indo-West Pacific (IWP). Here, we investigate processes implicit to the Centre of Origin, Centre of Overlap and Centre of Survival hypotheses. We focus on intraspecific genetic data, where intraspecific diversity can provide information regarding directionality of past dispersal (gene flow) and changes in population size such as expansion from refugia. LocationIndian Ocean, Coral Triangle and West Pacific Ocean. MethodsWe use existing mitochondrial DNA data augmented with new samples from the Philippines, thus filling in key sampling gaps from previous studies, to examine nine reef species. We first test if genetic diversity peaks in the CT. We then estimate gene flow between regionsCT vs. Indian Ocean (IO) and CT vs. Pacific Ocean (PO)to assess concordance with predicted directions of movement (i.e. into the CT or out of the CT). Finally, we investigate the historical demography of these species and evaluate population expansions to ascertain whether genetic diversity is correlated with putative Pleistocene refugia. ResultsGenetic diversity in the CT was not greater than genetic diversity in the IO and PO, and we found highly variable spatial patterns of genetic diversity when species were evaluated individually. Gene flow estimates indicated very limited exchange between regions, consistent with peripheral isolation and/or signals of historical divergence. For some species, however, there were signals of movement out of the CT. While there was no significant correlation between genetic diversity and putative refugia, genetic patterns suggested that some populations have experienced population expansions. The locations of expansion were also variable across species. Overall, there was no signal of concordant processes shaping intraspecific genetic diversity. Main conclusionsGenetic patterns across species were highly variable with some species lending weak support for processes aligned with each of the Centre of diversity hypotheses. But overall there was no compelling evidence for uniform processes underlying all species, which possibly indicate that processes underlying the different Centre have not strongly affected IWP species within the recent past. | NA | no | No good fitness measure and measure host GD | NA | no | no |
| 5323 | 2020 | 44040 | Matter, SF; Goff, J; Keyghobadi, N; Roland, J | 2020 | Direct estimates of metapopulation capacity from dispersal show high interannual variability, but little effect of recent forest encroachment on network persistence | Context Habitat loss and isolation are leading threats for biodiversity. Alpine habitat throughout the world is being lost and fragmented by forest encroachment. Previous analysis of forest encroachment from 1952 to 1993 in a population network along Jumpingpound Ridge, Alberta, Canada showed a 78% loss in meadow area, resulting in an estimated 41% reduction in dispersal of the alpine butterfly Parnassius smintheus. Objectives Here, we pursue three questions. First, has forest encroachment continued since 1993? Second, if so, has it affected butterfly dispersal? And third, have any changes altered estimates of metapopulation persistence of this species? Methods The area of each meadow and distance between each pair of meadows were determined in GIS using aerial photographs taken in 1952, 1993, 1999, 2008, and 2012. Mark-recapture (1995-2015) and landscape data were used to estimate annual butterfly dispersal and network persistence. Results Alpine meadows along Jumpingpound Ridge continued to be fragmented by encroaching forest showing a decrease in area and increase in the amount of forest between them. Annual dispersal parameters varied over the study, but the only consistent response to forest encroachment was an increase in male dispersal distance within meadow habitat. Estimates of metapopulation capacity also varied greatly among years due to variability in dispersal. The network was predicted to persist based on nearly all estimates. Conclusions Metapopulation capacity is a potentially powerful tool for conservation, but interannual variability in dispersal hampers its utility under realistic conditions and could result in erroneous conclusions regarding metapopulation persistence. The 7 km long network of 18 subpopulations of Parnassius smintheus along the Jumpingpound Ridge and Cox Hill is currently at little risk of extinction due to forest encroachment. | NA | no | No good measureGD of host | NA | no | no |
| 5324 | 2020 | 44040 | Matthee, CA | 2020 | The influence of host dispersal on the gene flow and genetic diversity of generalist and specialist ectoparasites(double dagger) | The dispersal and subsequent gene flow within parasite species is the result of a complex interaction between parasite life history, host life history and abiotic environmental factors. To gain more insights into the drivers responsible for parasite dispersal, COI mtDNA genetic data derived from six southern African generalist parasite species, including fleas, mites and ticks were compared with four specialist species from the same geographic region. Generalist tick species represented by Amblyomma hebraeum, Hyalomma truncatum and H. rufipes all occur temporarily on highly mobile ungulate hosts and showed high levels of haplotypic genetic diversity and high levels of dispersal with an average intraspecific global F-st (population differentiation index) value of 0.27 (+/- 0.13). Generalist parasites, such as fleas, Listropsylla agrippinae and Chiastopsylla rossi, and one mite species, Laelaps muricola, that are all semi-permanent on the host and restricted to less mobile hosts species, showed a similar high level of genetic diversity, but an intermediate average F-st value of 0.67 (+/- 0.11). Highly specialised semi-permanent parasites, such as the mite L. giganteus and the permanent lice Polyplax praomydis, Hoplopleura patersoni and P. arvicanthis recorded the lowest level of genetic diversity and a low level of gene flow among geographic sampling localities with an average F-st value of 0.95 (+/- 0.05). This study provides strong support for the Specialist Generalist Variation Hypothesis (SGVH) and highlights the role that host dispersal and host specialisation by parasites play in the dispersal and evolution of ectoparasites. | NA | no | No good fitness measure and measure host GD | NA | no | no |
| 5325 | 2020 | 44112 | Matthee, CA; Engelbrecht, A; Matthee, S | 2018 | Comparative phylogeography of parasitic Laelaps mites contribute new insights into the specialist-generalist variation hypothesis (SGVH) | Background: The specialist-generalist variation hypothesis (SGVH) in parasites suggests that, due to patchiness in habitat (host availability), specialist species will show more subdivided population structure when compared to generalist species. In addition, since specialist species are more prone to local stochastic extinction events with their hosts, they will show lower levels of intraspecific genetic diversity when compared to more generalist. Results: To test the wider applicability of the SGVH we compared 337 cytochrome oxidase I mitochondrial DNA and 268 nuclear tropomyosin DNA sequenced fragments derived from two co-distributed Laelaps mite species and compared the data to 294 COI mtDNA sequences derived from the respective hosts Rhabdomys dilectus, R. bechuanae, Mastomys coucha and M. natalensis. In support of the SGVH, the generalist L. muricola was characterized by a high mtDNA haplotypic diversity of 0.97 (+/- 0.00) and a low level of population differentiation (mtDNA F-st = 0.56, p < 0.05; nuDNA F-st = 033, P < 0.05) while the specialist L. giganteus was overall characterized by a lower haplotypic diversity of 0.77 (+/- 0.03) and comparatively higher levels of population differentiation (mtDNA F-st = 0.87, P < 0.05; nuDNA F-st = 0.48, P < 0.05). When the two specialist L. giganteus lineages, which occur on two different Rhabdomys species, are respectively compared to the generalist parasite, L. muricola, the SGVH is not fully supported. One of the specialist L. giganteus species occurring on R. dilectus shows similar low levels of population differentiation (mtDNA F-st = 053, P < 0. 05; nuDNA F-st = 0.12, P < 0.05) than that found for the generalist L. muricola. This finding can be correlated to differences in host dispersal: R. bechuanae populations are characterized by a differentiated mtDNA F-st of 0.79 (P < 0.05) while R. dilectus populations are less structured with a mtDNA F-st = 0.18 (P < 0.05). Conclusions: These findings suggest that in ectoparasites, host specificity and the vagility of the host are both important drivers for parasite dispersal. It is proposed that the SGHV hypothesis should also incorporate reference to host dispersal since in our case only the specialist species who occur on less mobile hosts showed more subdivided population structure when compared to generalist species. | NA | no | No good fitness measure and measure host GD | NA | no | no |
| 5326 | 2020 | 44042 | Mattucci, F; Galaverni, M; Lyons, LA; Alves, PC; Randi, E; Velli, E; Pagani, L; Caniglia, R | 2019 | Genomic approaches to identify hybrids and estimate admixture times in European wildcat populations | The survival of indigenous European wildcat (Fells silvestris silvestris) populations can be locally threatened by introgressive hybridization with free-ranging domestic cats. Identifying pure wildcats and investigating the ancestry of admixed individuals becomes thus a conservation priority. We analyzed 63k cat Single Nucleotide Polymorphisms (SNPs) with multivariate, Bayesian and gene-search tools to better evaluate admixture levels between domestic and wild cats collected in Europe, timing and ancestry proportions of their hybrids and backcrosses, and track the origin (wild or domestic) of the genomic blocks carried by admixed cats, also looking for possible deviations from neutrality in their inheritance patterns. Small domestic ancestry blocks were detected in the genomes of most admixed cats, which likely originated from hybridization events occurring from 6 to 22 generations in the past. We identified about 1,900 outlier coding genes with excess of wild or domestic ancestry compared to random expectations in the admixed individuals. More than 600 outlier genes were significantly enriched for Gene Ontology (GO) categories mainly related to social behavior, functional and metabolic adaptive processes (wild-like genes), involved in cognition and neural crest development (domestic-like genes), or associated with immune system functions and lipid metabolism (parental-like genes). These kinds of genomic ancestry analyses could be reliably applied to unravel the admixture dynamics in European wildcats, as well as in other hybridizing populations, in order to design more efficient conservation plans. | NA | no | No good fitness measure and measure host GD | NA | no | no |
| 5327 | 2020 | 44040 | Maya, HES; Mercado-Flores, Y; Tellez-Jurado, A; Perez-Camarillo, JP; Mejia, O; Anducho-Reyes, MA | 2020 | Molecular Variation of the Phytopathogenic Fungus Sporisorium reilianum in Valle del Mezquital, Hidalgo | Head smut in maize (Zea mays) is a systemic disease caused by the phytopathogenic fungus Sporisorium reilianum. One of the most severely affected regions in Mexico is Valle del Mezquital in the state of Hidalgo, a high production zone with irrigated agriculture that produces over 50% of maize supplies in the state. This study was designed to analyze the genetic diversity of this phytopathogen isolated from different zones of Valle del Mezquital using the following molecular markers: ITS, aspartyl protease, beta-tubulin, and the mitochondrial endonuclease LAGLIDADG. Our objective was to estimate the level of genetic variability in a regional space and understand the evolutionary processes to which non-model organisms are subjected. A total of 53 strains of S. reilianum were isolated from the sampling sites and then purified. A Bayesian Analysis of Population Structure (BAPS) analysis of the genetic structuring allowed us to recover three genetic groupings (K = 3), while molecular variance analysis (AMOVA) showed a very low fixation index (F-ST = 0.0146), which was not statistically significant and indicated little between-group genetic differentiation. Also, the value determined for the coefficient of genetic differentiation (Gst) showed little variation among the genetic groups, while the gene flow number (Nm) was 8.25. The Tajima’s D estimator and haplotype network recently indicated a rapid population expansion. Together, these results suggest that reproduction and migration are key factors in the evolution and virulence of this fungus. | NA | no | gd of pathogen | NA | no | no |
| 5328 | 2020 | 44112 | Mayer, I | 2019 | The Role of Reproductive Sciences in the Preservation and Breeding of Commercial and Threatened Teleost Fishes | The teleost fishes are the largest and most diverse vertebrate group, accounting for nearly half of all known vertebrate species. Teleost fish exhibit greater species diversity than any other group of vertebrates and this is reflected in the unique variety of different reproductive strategies displayed by fish. Fish have always been an important resource for humans worldwide, especially as food. While wild capture fisheries have historically been the main source of fish, the farming of fish (aquaculture) is increasingly becoming the more dominant source of food fish, and is predicted to account for 60% of total global fish production by 2030. Fishes are increasingly threatened by a wide range of anthropogenic impacts, including loss of habitat, pollution, invasive species and over-exploitation. In addition, climate change, especially the consequences of global warming, can impact fish at all levels of biological organization from the individual to the population level, influencing both physiological and ecological processes in a variety of direct and indirect ways. As such, there is an urgent need to protect and conserve the huge genetic diversity offered by this diverse vertebrate group, not just as a source of genes for contemporary breeding and for protection against the consequences of climate change and disease, but also as part of our national heritage. While the cryopreservation of reproductive cells is a means of achieving these objectives, currently only fish sperm can be successfully frozen. Due to their large size, large yolk compartment, low membrane permeability and high chilling sensitivity, successful and reproducible protocols for the cryopreservation of fish oocytes and embryos still remains elusive. However, significant advances have been made in the cryopreservation of primordial germ cells as an alternative means of conserving both paternal and maternal genomes. Although more research needs to be carried out on how these cells can be optimally applied to emerging reproductive technologies, including transplantation techniques and surrogate broodstock technologies, the successful cryopreservation of fish germ cells, and the establishment of genetic resource banks, offers the possibility of both conserving and restoring threatened species. Further, current and future conservation efforts need to consider the impact of climate change in both in situ conservation and reintroduction efforts. In conclusion, it is anticipated that the successful cryopreservation of fish germplasm will result in a range of economic, ecological and societal benefits. In partnership with emerging assisted reproductive technologies, the successful cryopreservation of fish germplasm will lead to more efficient reproduction in aquaculture, assist selective breeding programmes, and be of crucial importance to future species conservation actions. | NA | no | No good fitness measure and measure host GD | NA | no | no |
| 5329 | 2020 | 44112 | McCoy, AM; Norton, EM; Kemper, AM; Beeson, SK; Mickelson, JR; McCue, ME | 2019 | SNP-based heritability and genetic architecture of tarsal osteochondrosis in North American Standardbred horses | Osteochondrosis is a common developmental orthopedic disease characterized by a failure of endochondral ossification. Standardbred horses are recognized as being predisposed to tarsal osteochondrosis. Prior heritability estimates for tarsal osteochondrosis in European Standardbreds and related trotting breeds have been based on pedigree data and range from 17-29%. Here, we report on genetic architecture and heritability based on high-density genotyping data in a cohort of North American Standardbreds (n = 479) stringently phenotyped for tarsal osteochondrosis. Whole-genome array genotyping data were imputed to similar to 2 million single nucleotide polymorphisms (SNPs). SNP-based heritability of osteochondrosis in this population was explained by 2326 SNPs. The majority of these SNPs (86.6%) had small effects, whereas fewer SNPs had moderate or large effects (10% and 2.9% respectively), which is consistent with a polygenic/complex disease. Heritability was estimated at 0.24 +/- 0.16 using two methods of restricted maximum likelihood analysis, as implemented in gcta (with and without a weighted relatedness matrix) and ldak software. Estimates were validated using bootstrapping. Heritability estimates were within the range previously reported and suggest that osteochondrosis is moderately heritable but that a significant portion of disease risk is due to environmental factors and/or genotype x environment interactions. Future identification of the genes/variants that have the most impact on disease risk may allow early recognition of high-risk individuals. | NA | no | No good fitness measure and measure host GD | NA | no | no |
| 5330 | 2020 | 44112 | McFarlane, S; Manseau, M; Flasko, A; Horn, RL; Arnason, N; Neufeld, L; Bradley, M; Wilson, P | 2018 | Genetic influences on male and female variance in reproductive success and implications for the recovery of severely endangered mountain caribou | Small and declining populations of large mammals are vulnerable to stochastic events and can be at high risk of extinction. Population viability is also susceptible to the detrimental effects of low genetic diversity and inbreeding. The objective of this study was to do an assessment of three endangered caribou subpopulations using non-invasive genetic sampling to assess demographic population changes by combining capture-recapture modeling, familial pedigree networks, individual fitness and inbreeding coefficients. Three subpopulations of the Central Mountain caribou ecotype (Rangifer tarandus caribou) in Jasper National Park, Canada, were systematically surveyed over a 10-year period and fecal samples collected for DNA analyses. For the Tonquin, population size declined at a rate of 0.86 (95% CI 0.82, 0.91), the number of females decreased from 41 in 2006 to only 14 in 2015. The Brazeau and Maligne subpopulations also declined over the same period, from 22 to 11 individuals (11 and 5 females) in 2006, to 12 and 3 individuals (6 and 1 females) in 2015. The pedigree network confirmed limited interbreeding among subpopulations, varied fitness levels in both males and females, and evidence of inbreeding avoidance among high-fitness individuals. All population parameters pointed to a rapid population decline and low population sizes, putting them at high risk of extinction. The varying reproductive fitness observed amongst males and females was significant and should be considered in future population augmentation or reintroduction efforts. Improved connectivity among and with neighbouring subpopulations should also be considered to sustain or enhance genetic diversity. (C) 2018 Published by Elsevier B.V. | NA | no | No good fitness measure and measure host GD | NA | no | no |
| 5331 | 2020 | 44042 | McGaughran, A; Terauds, A; Convey, P; Fraser, CI | 2019 | Genome-wide SNP data reveal improved evidence for Antarctic glacial refugia and dispersal of terrestrial invertebrates | Antarctica is isolated, surrounded by the Southern Ocean and has experienced extreme environmental conditions for millions of years, including during recent Pleistocene glacial maxima. How Antarctic terrestrial species might have survived these glaciations has been a topic of intense interest, yet many questions remain unanswered, particularly for Antarctica’s invertebrate fauna. We examine whether genetic data from a widespread group of terrestrial invertebrates, springtails (Collembola, Isotomidae) of the genus Cryptopygus, show evidence for long-term survival in glacial refugia along the Antarctic Peninsula. We use genome-wide SNP analyses (via genotyping-by-sequencing, GBS) and mitochondrial data to examine population diversity and differentiation across more than 20 sites spanning >950 km on the Peninsula, and from islands both close to the Peninsula and up to similar to 1,900 km away. Population structure analysis indicates the presence of strong local clusters of diversity, and we infer that patterns represent a complex interplay of isolation in local refugia coupled with occasional successful long-distance dispersal events. We identified wind and degree days as significant environmental drivers of genetic diversity, with windier and warmer sites hosting higher diversity. Thus, we infer that refugial areas along the Antarctic Peninsula have allowed populations of indigenous springtails to survive in situ throughout glacial periods. Despite the difficulties of dispersal in cold, desiccating conditions, Cryptopygus springtails on the Peninsula appear to have achieved multiple long-distance colonization events, most likely through wind-related dispersal events. | NA | no | No good fitness measure | NA | no | no |
| 5332 | 2020 | 44039 | McKnight, DT; Carr, LJ; Bower, DS; Schwarzkopf, L; Alford, RA; Zenger, KR | 2020 | Infection dynamics, dispersal, and adaptation: understanding the lack of recovery in a remnant frog population following a disease outbreak | Emerging infectious diseases can cause dramatic declines in wildlife populations. Sometimes, these declines are followed by recovery, but many populations do not recover. Studying differential recovery patterns may yield important information for managing disease-afflicted populations and facilitating population recoveries. In the late 1980s, a chytridiomycosis outbreak caused multiple frog species in Australia’s Wet Tropics to decline. Populations of some species (e.g., Litoria nannotis) subsequently recovered, while others (e.g., Litoria dayi) did not. We examined the population genetics and current infection status of L. dayi, to test several hypotheses regarding the failure of its populations to recover: (1) a lack of individual dispersal abilities has prevented recolonization of previously occupied locations, (2) a loss of genetic variation has resulted in limited adaptive potential, and (3) L. dayi is currently adapting to chytridiomycosis. We found moderate-to-high levels of gene flow and diversity (Fst range: <0.01-0.15; minor allele frequency (MAF): 0.192-0.245), which were similar to previously published levels for recovered L. nannotis populations. This suggests that dispersal ability and genetic diversity do not limit the ability of L. dayi to recolonize upland sites. Further, infection intensity and prevalence increased with elevation, suggesting that chytridiomycosis is still limiting the elevational range of L. dayi. Outlier tests comparing infected and uninfected individuals consistently identified 18 markers as putatively under selection, and several of those markers matched genes that were previously implicated in infection. This suggests that L. dayi has genetic variation for genes that affect infection dynamics and may be undergoing adaptation. | NA | no | No good fitness measure | NA | no | no |
| 5333 | 2020 | 44111 | McKnight, DT; Lal, MM; Bower, DS; Schwarzkopf, L; Alford, RA; Zenger, KR | 2019 | The return of the frogs: The importance of habitat refugia in maintaining diversity during a disease outbreak | Recent decades have seen the emergence and spread of numerous infectious diseases, often with severe negative consequences for wildlife populations. Nevertheless, many populations survive the initial outbreaks, and even undergo recoveries. Unfortunately, the long-term effects of these outbreaks on host population genetics are poorly understood; to increase this understanding, we examined the population genetics of two species of rainforest frogs (Litoria nannotis and Litoria serrata) that have largely recovered from a chytridiomycosis outbreak at two national parks in the Wet Tropics of northern Australia. At the wetter, northern park there was little evidence of decreased genetic diversity in either species, and all of the sampled sites had high minor allele frequencies (mean MAF = 0.230-0.235), high heterozygosity (0.318-0.325), and few monomorphic markers (1.4%-4.0%); however, some recovered L. nannotis populations had low N-e values (59.3-683.8) compared to populations that did not decline during the outbreak (1,537.4-1,756.5). At the drier, southern park, both species exhibited lower diversity (mean MAF = 0.084-0.180; heterozygosity = 0.126-0.257; monomorphic markers = 3.7%-43.5%; N-e = 18.4-676.1). The diversity patterns in this park matched habitat patterns, with both species having higher diversity levels and fewer closely related individuals at sites with higher quality habitat. These patterns were more pronounced for L. nannotis, which has lower dispersal rates than L. serrata. These results suggest that refugia with high quality habitat are important for retaining genetic diversity during disease outbreaks, and that gene flow following disease outbreaks is important for re-establishing diversity in populations where it was reduced. | NA | no | No good fitness measure | NA | no | no |
| 5334 | 2020 | 44113 | McLaughlin, HP; Cherney, B; Hakovirta, JR; Priestley, RA; Conley, A; Carter, A; Hodge, D; Pillai, SP; Weigel, LM; Kersh, GJ; Sue, D | 2017 | Phylogenetic inference of Coxiella burnetii by 16S rRNA gene sequencing | Coxiella burnetii is a human pathogen that causes the serious zoonotic disease Q fever. It is ubiquitous in the environment and due to its wide host range, long-range dispersal potential and classification as a bioterrorism agent, this microorganism is considered an HHS Select Agent. In the event of an outbreak or intentional release, laboratory strain typing methods can contribute to epidemiological investigations, law enforcement investigation and the public health response by providing critical information about the relatedness between C. burnetii isolates collected from different sources. Laboratory cultivation of C. burnetii is both time-consuming and challenging. Availability of strain collections is often limited and while several strain typing methods have been described over the years, a true gold-standard method is still elusive. Building upon epidemiological knowledge from limited, historical strain collections and typing data is essential to more accurately infer C. burnetii phylogeny. Harmonization of auspicious high-resolution laboratory typing techniques is critical to support epidemiological and law enforcement investigation. The single nucleotide polymorphism (SNP)-based genotyping approach offers simplicity, rapidity and robustness. Herein, we demonstrate SNPs identified within 16S rRNA gene sequences can differentiate C. burnetii strains. Using this method, 55 isolates were assigned to six groups based on six polymorphisms. These 16S rRNA SNP-based genotyping results were largely congruent with those obtained by analyzing restriction-endonuclease (RE)-digested DNA separated by SDS-PAGE and by the high-resolution approach based on SNPs within multispacer sequence typing (MST) loci. The SNPs identified within the 16S rRNA gene can be used as targets for the development of additional SNP-based genotyping assays for C. burnetii. | NA | no | No good fitness measure and measure host GD | NA | no | no |
| 5335 | 2020 | 44043 | McLennan, D; Auer, SK; Anderson, GJ; Reid, TC; Bassar, RD; Stewart, DC; Cauwelier, E; Sampayo, J; McKelvey, S; Nislow, KH; Armstrong, JD; Metcalfe, NB | 2019 | Simulating nutrient release from parental carcasses increases the growth, biomass and genetic diversity of juvenile Atlantic salmon | The net transport of nutrients by migratory fish from oceans to inland spawning areas has decreased due to population declines and migration barriers. Restoration of nutrients to increasingly oligotrophic upland streams (that were historically salmon spawning areas) have shown short-term benefits for juvenile salmon, but the longer term consequences are little known. Here we simulated the deposition of a small number of adult Atlantic salmon Salmo salar carcasses at the end of the spawning period in five Scottish upland streams (‘high parental nutrient’ treatment), while leaving five reference streams without carcasses (‘low parental nutrient’ treatment). All streams received exactly the same number of salmon eggs (n = 3,000) drawn in equal number from the same 30 wild-origin families, thereby controlling for initial egg density and genetic composition. We then monitored the resulting juvenile salmon and their macroinvertebrate prey, repeating the carcass addition treatment in the next spawning season. Macroinvertebrate biomass and abundance were five times higher in the high parental nutrient streams, even 1 year after the carcass addition, and led to faster growth of juvenile salmon over the next 2 years (but with no change in population density). This faster growth led to more fish exceeding the size threshold that would trigger emigration to sea at 2 rather than 3 years of age. There was also higher genetic diversity among surviving salmon in high parental nutrient streams; genotyping showed that these effects were not due to immigration but to differential survival. Synthesis and applications. This 2-year field experiment shows that adding nutrients that simulate the presence of small numbers of adult salmon carcasses can have long-term effects on the growth rate of juvenile salmon, likely increasing the number that will migrate to sea early and also increasing their genetic diversity. However, the feasibility of adding nutrients to spawning streams as a management tool to boost salmon populations will depend on whether the benefits at this stage are maintained over the entire life cycle. | NA | no | No good fitness measure and measure host GD | NA | no | no |
| 5336 | 2020 | 44113 | McLennan, EA; Gooley, RM; Wise, P; Belov, K; Hogg, CJ; Grueber, CE | 2018 | Pedigree reconstruction using molecular data reveals an early warning sign of gene diversity loss in an island population of Tasmanian devils (Sarcophilus harrisii) | Tasmanian devils have experienced an 85% population decline since the emergence of an infectious cancer. In response, a captive insurance population was established in 2006 with a subpopulation later introduced onto Maria Island, Tasmania. We aimed to (1) examine the genetic parameters of the Maria Island population as a stand-alone site and within its broader metapopulation context, (2) assess the efficacy of assisted colonisations, and (3) inform future translocations. This study reconstructs the pedigree of 86 island-born devils using 31 polymorphic microsatellite loci. Combined molecular and pedigree analysis was used to monitor change in population genetic parameters in 4 years since colonisation. Molecular analysis alone revealed no significant change in genetic diversity, while DNA-reconstructed pedigree analysis revealed a statistically significant increase in inbreeding due to skewed founder representation. Pedigree modelling predicted that gene diversity would only be maintained above the threshold of 95% for a further 2 years, dropping to 77.1% after 40 years. Modelling alternative supplementation strategies revealed introducing eight new founders every 3 years will enable the population to retain 95% gene diversity until 2056, provided the translocated animals breed; to ensure this we recommend introducing ten new females every 3 years. We highlight the value of combining pedigree analyses with molecular data, from both a single-site and metapopulation viewpoint, for analysing changes in genetic parameters within populations of conservation concern. The importance of post-release genetic monitoring in an established population is emphasised, given how quickly inbreeding can accumulate and gene diversity be lost. | NA | no | No good fitness measure and measure host GD | NA | no | no |
| 5337 | 2020 | 44040 | McLennan, EA; Grueber, CE; Wise, P; Belov, K; Hogg, CJ | 2020 | Mixing genetically differentiated populations successfully boosts diversity of an endangered carnivore | Biodiversity decline and genetic erosion are among the most challenging conservation issues. Genetic admixture, the mixing of two or more genetically differentiated populations, can increase genetic diversity of admixed individuals. However, genetic admixture for conservation purposes is rare due to concerns over outbreeding depression, loss of local adaptations and scepticism regarding the benefits of mixing populations. We used an introduced population of Tasmanian devils Sarcophilus harrisii descended from two genetically differentiated source populations to illustrate the benefits of genetic admixture for translocation programmes. Devils are endangered due to an infectious cancer causing 80% population declines across their range since disease emergence in 1996. Devil populations are known to be structured on an east-west cline across Tasmania. As part of their conservation management, devils were introduced to Maria Island, Tasmania in an assisted colonization in 2012 with supplementations in 2013 and 2017. Of the released individuals (N = 34), 23 were western (13M; 10F), four were eastern (4F) and seven were of mixed origin (2M; 5F). The genetic composition of devils born on Maria Island (N = 185) was examined using 927 single nucleotide polymorphism loci. Using a reconstructed pedigree for each individual, we examined the proportion of founding origin (east, west or mixed) and used Shannon’s Diversity Index to quantify the evenness of founder origin. Individuals with mixed origins (N = 102) had higher genetic diversity than purebred individuals (N = 83), and increased evenness of founder origin was positively correlated with genetic diversity. Increased genetic diversity had no influence on reproductive success. For a genetically depauperate species, mixing individuals descended from differentiated populations, resulted in increased diversity in subsequent generations. This finding permits conservation managers to select individuals for translocation that produce offspring with higher genetic diversity, creating high-diversity source populations that can be used when augmenting other declining wild populations. | NA | no | No good fitness measure and measure host GD | NA | no | no |
| 5338 | 2020 | 44113 | McMullan, M; Rafiqi, M; Kaithakottil, G; Clavijo, BJ; Bilham, L; Orton, E; Percival-Alwyn, L; Ward, B; Edwards, A; Saunders, DGO; Accinelli, GG; Wright, J; Verweij, W; Koutsovoulos, G; Yoshida, K; Hosoya, T; Williamson, L; Jennings, P; Ioos, R; Husson, C; Hietala, AM; Vivian-Smith, A; Solheim, H; MaClean, D; Fosker, C; Hall, N; Brown, JKM; Swarbreck, D; Blaxter, M; Downie, JA; Clark, MD | 2018 | The ash dieback invasion of Europe was founded by two genetically divergent individuals | Accelerating international trade and climate change make pathogen spread an increasing concern. Hymenoscyphus fraxineus, the causal agent of ash dieback, is a fungal pathogen that has been moving across continents and hosts from Asian to European ash. Most European common ash trees (Fraxinus excelsior) are highly susceptible to H. fraxineus, although a minority (-5%) have partial resistance to dieback. Here, we assemble and annotate a H. fraxineus draft genome, which approaches chromosome scale. Pathogen genetic diversity across Europe and in Japan, reveals a strong bottleneck in Europe, though a signal of adaptive diversity remains in key host interaction genes. We find that the European population was founded by two divergent haploid individuals. Divergence between these haplotypes represents the ancestral polymorphism within a large source population. Subsequent introduction from this source would greatly increase adaptive potential of the pathogen. Thus, further introgression of H. fraxineus into Europe represents a potential threat and Europe-wide biological security measures are needed to manage this disease. | NA | no | No good fitness measure and measure host GD | NA | no | no |
| 5339 | 2020 | 44113 | Mdladla, K; Dzomba, EF; Muchadeyi, FC | 2018 | Landscape genomics and pathway analysis to understand genetic adaptation of South African indigenous goat populations | In Africa, extensively raised livestock populations in most smallholder farming communities are exposed to harsh and heterogeneous climatic conditions and disease pathogens that they adapt to in order to survive. Majority of these livestock species, including goats, are of non-descript and uncharacterized breeds and their response to natural selection presented by heterogeneous environments is still unresolved. This study investigated genetic diversity and its association with environmental and geographic conditions in 194 South African indigenous goats from different geographic locations genotyped on the Illumina goat SNP50K panel. Population structure analysis revealed a homogeneous genetic cluster of the Tankwa goats, restricted to the Northern Cape province. Overall, the Boer, Kalahari Red, and Savanna showed a wide geographic spread of shared genetic components, whereas the village ecotypes revealed a longitudinal distribution. The relative importance of environmental factors on genetic variation of goat populations was assessed using redundancy analysis (RDA). Climatic and geographic variables explained 22% of the total variation while climatic variables alone accounted for 17% of the diversity. Geographic variables solitarily explained 1% of the total variation. The first axis (Model I) of the RDA analysis revealed 329 outlier SNPs. Landscape genomic approaches of spatial analysis method (SAM) identified a total of 843 (1.75%) SNPs, while latent factor mixed models (LFMM) identified 714 (1.48%) SNPs significantly associated with environmental variables. Significant markers were within genes involved in biological functions potentially important for environmental adaptation. Overall, the study suggested environmental factors to have some effect in shaping the genetic variation of South African indigenous goat populations. Loci observed to be significant and under selection may be responsible for the adaption of the goat populations to local production systems. | NA | no | No good fitness measure | NA | no | no |
| 5340 | 2020 | 44112 | Meemongkolkiat, T; Rattanawannee, A; Chanchao, C | 2019 | Genetic Diversity of Apis spp. in Thailand Inferred from 28S rRNA Nuclear and Cytochrome b Mitochondrial Gene Sequences | Knowledge of the genetic diversity of Apis spp. is important in order to provide a better understanding of breeding strategies that relate to the conservation of wild species and colony survival of farmed species. Here, honeybees of five Apis species were collected from 12 provinces throughout Thailand. After DNA extraction, 28S rRNA nuclear (710 bp) and cytochrome b (cytb) mitochondrial (520 bp) gene fragments were sequenced. Homologous sequences (nucleotide identity of over 95%) were obtained from GeneBank using the BLASTn algorithm, aligned, and analysed by maximum likelihood and Bayesian inference phylogenetics. For 28S rRNA, a low genetic variation was detected within species (haplotype diversity ranging from 0.212 to 0.394), while 1(polymorphic sites were detected between species. Although the relative haplotype diversity was high, a low nucleotide divergence was found (0.7%), with migratory species. For cytb, the sequence divergence ranged from 0.24 to 3.88% within species and 7.35 to 13.07% between species. The divergence of cytb was higher than that of 28S rRNA. A. cerana showed two distinct clades between Southern Thailand and the other regions. Groups of A. cerana (Asian cavity-nesting), A. mellifera (European cavity-nesting), A. dorsata (giant open-nesting), and A. florea and A. andreniformis (dwarf bees) were defined in the 28S rRNA and cytb tree topologies. | NA | no | No good fitness measure | NA | no | no |
| 5341 | 2020 | 44040 | Mehmood, S; Sajid, M; Huang, LL; Kang, ZS | 2020 | Alternate hosts and their impact on genetic diversity of Puccinia striiformis f. sp. tritici and disease epidemics | Since the discovery of Berberis and Mahonia species. as aecial hosts of the wheat stripe rust pathogen, Puccinia striiformis Westend. f. sp. tritici Eriks. (Pst), their possible role in generating new races of Pst through sexual reproduction, has become a hot issue in the agricultural scientific community. To date, all the investigations about the role of alternate hosts in the occurrence of the wheat stripe rust disease epidemics reveal that it depends on the pathogen species, environmental conditions, and specific climatic situations. In this review, we summarize the current status of alternate hosts of Pst, and their impact on genetic diversity and disease epidemics. Most importantly, an overview of the recent research progress in understanding the role of alternate hosts of Pst is provided. | NA | no | No good fitness measure and measure host GD | NA | no | no |
| 5342 | 2020 | 44039 | Mekonnen, T; Haileselassie, T; Goodwin, SB; Tesfayea, K | 2020 | Genetic diversity and population structure of Zymoseptoria tritici in Ethiopia as revealed by microsatellite markers | Septoria tritici blotch (STB), caused by Zymoseptoria tritici (formerly: Mycosphaerella graminicola or Septoria tritici), is one of the most devastating diseases of wheat globally. Understanding genetic diversity of the pathogen has supreme importance in developing best management strategies. However, there is dearth of information on the genetic structure of Z. tritici populations in Ethiopia. Therefore, the present study was targeted to uncover the genetic diversity and population structure of Z. tritici populations from the major wheat-growing areas of Ethiopia. Totally, 182 Z. tritici isolates representing eight populations were analyzed with 14 microsatellite markers. All the microsatellite loci were polymorphic and highly informative, and hence useful genetic tools to depict the genetic diversity and population structure of the pathogen. A wide range of diversity indices including number of observed alleles, effective number of alleles, Shannon’s diversity index, number of private alleles, Nei’s gene diversity and percentage of polymorphic loci (PPL) were computed to determine genetic variation within populations. A high within-populations genetic diversity was confirmed with gene diversity index and PPL values ranging from 0.34 - 0.58 and 79-100% with overall mean of 0.45 and 94%, respectively. Analysis of molecular variance (AMOVA) revealed a moderate genetic differentiation where 92% of the total genetic variation resides within populations, leaving only 8% among populations. Cluster (UPGMA), PCoA and STRUCTURE analyses did not group the populations into sharply genetically distinct clusters according to their geographical origins, likely due to high gene flow (Nm = 5.66) and reproductive biology of the pathogen. All individual samples shared alleles from two subgroups (K = 2) evidencing high potential of genetic admixture. In conclusion, the microsatellite markers used in the present study were highly informative and thus, helped to dissect the genetic structures of Z. tritici populations in Ethiopia. Among the studied populations, those of East Shewa, Arsi, South West Shewa and Bale showed a high genetic diversity, and hence these areas can be considered as hot spots for investigations planned on the pathogen and host-pathogen interactions. Therefore, the present study not only enriches missing information in Ethiopia but also provides new insights into the epidemiology and genetic structure of Z. tritici in Africa where the agro-climatic conditions and the wheat cropping systems are different from other parts of the world. Such baseline information is useful for designing and implementing durable and effective management strategies. | NA | no | GD of pathogen | NA | no | no |
| 5343 | 2020 | 44112 | Melendez-Rosa, J; Bi, K; Lacey, EA | 2018 | Genomic analysis of MHC-based mate choice in the monogamous California mouse | Variation at Major Histocompatibility Complex (MHC) genes is thought to be an important mechanism underlying mate choice in vertebrates, with individuals typically predicted to prefer MHC-dissimilar reproductive partners. However, analyses based on individual MHC loci have generated contradictory results regarding the role of these genes in mate-choice decisions. To provide a more comprehensive assessment of relationships between MHC variation and mating behavior, we used an exome capture strategy to characterize variability at 13 MHC loci, 312 innate immune system genes, and 1044 nonimmune genes in 25 obligate monogamous pairs of California mice (Peromyscus californicus) from 2 free-living populations of this species in Monterey County, California. Pairwise genotypic comparisons and analyses of SNP-based allelic differences failed to detect disassortative mating based on MHC variability; reproductive partners were not more dissimilar than randomly generated male-female pairs at MHC, innate or nonimmune loci. Within populations, individuals tended to be more closely related at MHC genes than at innate or nonimmune genes. Consistent with the functional role of immunogenes, the 2 study populations were highly differentiated at MHC and innate genes but not at nonimmune loci. Collectively, our results suggest that MHC genetic variation in California mice reflects local differences in pathogen exposure rather than disassortative mating based on variability at MHC Class I and II genes. | NA | no | No good fitness measure and measure host GD | NA | no | no |
| 5344 | 2020 | 44039 | Melo-Carrillo, A; Dunn, JC; Cortes-Ortiz, L | 2020 | Low genetic diversity and limited genetic structure across the range of the critically endangered Mexican howler monkey (Alouatta palliata mexicana) | Genetic diversity provides populations with the possibility to persist in ever-changing environments, where selective regimes change over time. Therefore, the long-term survival of a population may be affected by its level of genetic diversity. The Mexican howler monkey (Alouatta palliata mexicana) is a critically endangered primate restricted to southeast Mexico. Here, we evaluate the genetic diversity and population structure of this subspecies based on 83 individuals from 31 groups sampled across the distribution range of the subspecies, using 29 microsatellite loci. Our results revealed extremely low genetic diversity (H-O = 0.21,H-E = 0.29) compared to studies of otherA. palliatapopulations and to otherAlouattaspecies. Principal component analysis, a Bayesian clustering method, and analyses of molecular variance did not detect strong signatures of genetic differentiation among geographic populations of this subspecies. Although we detect small but significantF(ST)values between populations, they can be explained by a pattern of isolation by distance. These results and the presence of unique alleles in different populations highlight the importance of implementing conservation efforts in multiple populations across the distribution range ofA. p. mexicanato preserve its already low genetic diversity. This is especially important given current levels of population isolation due to the extreme habitat fragmentation across the distribution range of this primate. | NA | no | No good fitness measure and measure host GD - maybe inbred? | NA | no | no |
| 5345 | 2020 | 44040 | Melo, LC; Silva, ACF; Pereira, HS; Melo, PGS; Souza, TLPO | 2019 | Genetic diversity as an additional selection criterion in a carioca common bean recurrent selection program for resistance to common bacterial blight | Common beans are a key source of protein and are consumed daily by most of the Brazilian population. More than 70% of what is consumed in this country is classified as carioca beans, based on seed qualities and appearance. We evaluated progenies of carioca common beans of the Brazilian agriculture research agency (Embrapa) recurrent selection program. This recurrent selection program is based on resistance to common bean bacterial blight (CBB), seed yield, and other important agronomic traits. Selection also considers the genetic representativeness of the parents and the genetic diversity among phenotypically selected progenies. Initially, 60 superior progenies were selected based on resistance to CBB and on seed type, in two locations (Santo Antonio de Goias, GO and Ponta Grossa, PR). These progenies were evaluated in the C0S0:2 generation in field trials in six locations during the rainy growing season. A randomized block experimental design was used with two replications, in plots of two 3-m rows spaced at 0.5 m. The most promising C0S0:3 progenies for combined agronomic performance in the different environments were selected and were then evaluated by means of 24 microsatellite molecular markers for the purpose of determining the genetic representativeness of their parents and the genetic diversity among them. The phenotypic data was subjected to analysis of variance for each trait in each of the environments, and then joint analyses were performed. To estimate genetic diversity among the progenies, the Rogers-W genetic distance was used, and a dissimilarity matrix was used to construct a dendrogram of genetic distances through the UPGMA method. Among the 60 C0S0:2 progenies that were evaluated, those that stood out for resistance to CBB also stood out for seed yield and for resistance to other diseases. Various progenies were superior, exhibiting large genetic distances between each other and in relation to the parents. This indicates a possibility of direct and indirect gains from the recurrent selection program. This molecular information will help direct selection of individuals for future recombination cycles. | NA | no | agricultured plant | NA | no | no |
| 5346 | 2020 | 44041 | Menchaca, A; Rossi, NA; Froidevaux, J; Dias-Freedman, I; Caragiulo, A; Wultsch, C; Harmsen, B; Foster, R; de la Torre, JA; Medellin, RA; Rabinowitz, S; Amato, G | 2019 | Population genetic structure and habitat connectivity for jaguar (Panthera onca) conservation in Central Belize | Background Connectivity among jaguar (Panthera onca) populations will ensure natural gene flow and the long-term survival of the species throughout its range. Jaguar conservation efforts have focused primarily on connecting suitable habitat in a broad-scale. Accelerated habitat reduction, human-wildlife conflict, limited funding, and the complexity of jaguar behaviour have proven challenging to maintain connectivity between populations effectively. Here, we used non-invasive genetic sampling and individual-based conservation genetic analyses to assess genetic diversity and levels of genetic connectivity between individuals in the Cockscomb Basin Wildlife Sanctuary and the Maya Forest Corridor. We used expert knowledge and scientific literature to develop models of landscape permeability based on circuit theory with fine-scale landscape features as ecosystem types, distance to human settlements and roads to predict the most probable jaguar movement across central Belize. Results We used 12 highly polymorphic microsatellite loci to identify 50 individual jaguars. We detected high levels of genetic diversity across loci (H-E = 0.61, H-O = 0.55, and N-A = 9.33). Using Bayesian clustering and multivariate models to assess gene flow and genetic structure, we identified one single group of jaguars (K = 1). We identified critical areas for jaguar movement that fall outside the boundaries of current protected areas in central Belize. We detected two main areas of high landscape permeability in a stretch of approximately 18 km between Sittee River Forest Reserve and Manatee Forest Reserve that may increase functional connectivity and facilitate jaguar dispersal from and to Cockscomb Basin Wildlife Sanctuary. Our analysis provides important insights on fine-scale genetic and landscape connectivity of jaguars in central Belize, an area of conservation concern. Conclusions The results of our study demonstrate high levels of relatively recent gene flow for jaguars between two study sites in central Belize. Our landscape analysis detected corridors of expected jaguar movement between the Cockscomb Basin Wildlife Sanctuary and the Maya Forest Corridor. We highlight the importance of maintaining already established corridors and consolidating new areas that further promote jaguar movement across suitable habitat beyond the boundaries of currently protected areas. Continued conservation efforts within identified corridors will further maintain and increase genetic connectivity in central Belize. | NA | no | No group level | NA | no | no |
| 5347 | 2020 | 44039 | Mendelsohn, B; Bedrosian, B; Stowell, SML; Gagne, RB; LaCava, MEF; Godwin, BL; Hull, JM; Ernest, HB | 2020 | Population genomic diversity and structure at the discontinuous southern range of the Great Gray Owl in North America | Species’ distributions are often discontinuous near the edge of the range where the environment may be more variable than the core of the range. Range discontinuity can reduce or cut off gene flow to small peripheral populations and lead to genetic drift and subsequent loss of genetic diversity. The southern extent of the Great Gray Owl (Strix nebulosa) range in North America is discontinuous, unlike their northern core range across the boreal forests. We sampled owls from five different locations on the periphery of the range across the western US (Wyoming, Idaho, California, northern Oregon, and southern Oregon) to investigate genetic population structure and genetic diversity. Using a reduced-representation genomic sequencing approach to genotype 123 individuals at 4817 single nucleotide polymorphic loci, we identified four genetically differentiated populations: California, southern Oregon, northern Oregon, and Wyoming and Idaho grouped together as a single Rocky Mountain population. The four genetically differentiated populations of Great Gray Owls identified in this study display high differentiation and low genetic variation, which is suggestive of long-term isolation and lack of connectivity, potentially caused by range discontinuity. The populations that lack habitat connectivity to the rest of the breeding range (i.e. those in California and Oregon) had lower genetic diversity than the Rocky Mountain population that is connected to the core of the range. These factors and other risks (such as disease and human-caused mortality) heighten susceptibility of these range-edge populations to future habitat and climate changes, genetic diversity erosion, and potential extinction vortex. For these reasons, protecting and monitoring this species on the southern edge of their range is vital. | NA | no | No good fitness measure | NA | no | no |
| 5348 | 2020 | 44113 | Meng, LX; Wang, YM; Wei, WH; Zhang, HY | 2018 | Population genetic structure of Diaphorina citri Kuwayama (Hemiptera: Liviidae): host-driven genetic differentiation in China | The Asian citrus psyllid Diaphorina citri Kuwayama is a major pest in citrus production, transmitting Candidatus Liberibacter asiaticus. It has spread widely across eastern and southern China. Unfortunately, little is known about the genetic diversity and population structure of D. citri, making pest control difficult. In this study, nine specifically developed SSR markers and three known mitochondrial DNA were used for population genetics study of D. citri using 225 samples collected from all 7 distribution regions in China. Based on the SSR data, D. citri was found highly diverse with a mean observed heterozygosity of 0.50, and three subgroups were structured by host plant: (i) Shatangju, NF mandarin and Ponkan; (ii) Murraya paniculata and Lemon; (iii) Citrus unshiu, Bingtangcheng, Summer orange and Navel. No significant genetic differences were found with mtDNA data. We suggested the host-associated divergence is likely to have occurred very recently. A unimodal distribution of paired differences, the negative and significant Tajima’s D and Fu’s F-S parameters among mtDNA suggested a recent demographic expansion. The extensive citrus cultivation and increased suitable living habitat was recommended as a key for this expansion event. | NA | no | gd of pathogen | NA | no | no |
| 5349 | 2020 | 44113 | Mesquita, AFC; Lambertini, C; Lyra, M; Malagoli, LR; James, TY; Toledo, LF; Haddad, CFB; Becker, CG | 2017 | Low resistance to chytridiomycosis in direct-developing amphibians | Host-generalist pathogens sporadically infect naive hosts, potentially triggering epizootics. The waterborne fungus Batrachochytrium dendrobatidis (Bd) is linked to declines of hundreds of amphibian species with aquatic larvae. Although several population declines and extinctions attributed to Bd have been reported among cryptic species undergoing direct development away from water, epidemiological studies focused on these terrestrial frogs are lacking. Our field data support that terrestrial direct-developing hosts are less exposed to Bd during their ontogeny than species with aquatic larvae, and thus they might lack adaptive responses against waterborne chytrids. Using controlled laboratory experiments, we exposed wild-caught amphibian species with terrestrial and aquatic life histories to Bd and found that direct developers showed more rapid increases in infection loads and experienced higher mortality rates than species with aquatic larvae. Our findings provide novel information about host responses to generalist pathogens and specifically show that our focal direct developing species have low resistance to Bd infections. Finally, our results underscore that we should not ignore Bd as a potential threat to direct developing species simply because they are less exposed to Bd in nature; instead future amphibian conservation plans should include efforts to safeguard hundreds of direct-developing amphibian species globally. | NA | no | No good fitness measure | NA | no | no |
| 5350 | 2020 | 44040 | Meyermans, R; Gorssen, W; Wijnrocx, K; Lenstra, JA; Vellema, P; Buys, N; Janssens, S | 2020 | Unraveling the genetic diversity of Belgian Milk Sheep using medium-density SNP genotypes | The present study focuses on the Belgian Milk Sheep in Flanders (Belgium) and compares its genetic diversity and relationship with the Flemish Sheep, the Friesian Milk Sheep, the French Lacaune dairy sheep and other Northern European breeds. For this study, 94 Belgian Milk Sheep, 23 Flemish Sheep and 22 Friesian Milk Sheep were genotyped with the OvineSNP50 array. In addition, 29 unregistered animals phenotypically similar to Belgian Milk Sheep were genotyped using the 15K ISGC chip. Both Belgian and Friesian Milk Sheep as well as the East Friesian Sheep were found to be less diverse than the other seven breeds included in this study. Genomic inbreeding coefficients based on runs of homozygosity (ROH) were estimated at 14.5, 12.4 and 10.2% for Belgian Milk Sheep, Flemish Sheep and Friesian Milk Sheep respectively. Out of 29 unregistered Belgian Milk Sheep, 28 mapped in the registered Belgian Milk Sheep population. Ancestry analysis, PCA and F-ST calculations showed that Belgian Milk Sheep are more related to Friesian Milk Sheep than to Flemish Sheep, which was contrary to the breeders’ expectations. Consequently, breeders may prefer to crossbreed Belgian Milk Sheep with Friesian sheep populations (Friesian Milk Sheep or East Friesian Sheep) in order to increase diversity. This research underlines the usefulness of SNP chip genotyping and ROH analyses for monitoring genetic diversity and studying genetic links in small livestock populations, profiting from internationally available genotypes. As assessment of genetic diversity is vital for long-term breed survival, these results will aid flockbooks to preserve genetic diversity. | NA | no | dometicated host | NA | no | no |
| 5351 | 2020 | 44113 | Michalecka, M; Masny, S; Leroy, T; Pulawska, J | 2018 | Population structure of Venturia inaequalis, a causal agent of apple scab, in response to heterogeneous apple tree cultivation | Background: Tracking newly emergent virulent populations in agroecosystems provides an opportunity to increase our understanding of the co-evolution dynamics of pathogens and their hosts. On the one hand host plants exert selective pressure on pathogen populations, thus dividing them into subpopulations of different virulence, while on the other hand they create an opportunity for secondary contact between the two divergent populations on one tree. The main objectives of the study were to explore whether the previously reported structure between two Venturia inaequalis population types, virulent or avirulent towards Malus x domestica cultivars carrying Rvi6 gene, is maintained or broken several years after the first emergence of new virulent strains in Poland, and to investigate the relationship between ‘new’ and ‘native’ populations derived from the same commercial orchards. For this purpose, we investigated the genetic structure of populations of the apple scab fungus, occurring on apple tree cultivars containing Rvi6, Rvi1 or Rvi17 resistance gene or no resistance at all, based on microsatellite data obtained from 606 strains sampled in 10 orchards composed of various host cultivars. Results: Application of genetic distance inferring and clustering methods allowed us to observe clear genetic distinctness of the populations virulent towards cultivars carrying Rvi6 gene from the Rvi6-avirulent populations and substructures within the Rvi6-group as a consequence of independent immigration events followed by rare, long-distance dispersals. We did not observe such a structuring effect of other genes determining apple scab resistance on any other populations, which in turn were genetically homogenous. However, in two orchards the co-occurrence of strains of different virulence pattern on the same trees was detected, blurring the genetic boundaries between populations. Conclusions: Among several resistance genes studied, only Rvi6 exerted selective pressure on pathogens populations: those virulent toward Rvi6 hosts show unique and clear genetic and virulence pattern. For the first time in commercial Malus x domestica orchards, we reported secondary contacts between populations virulent and avirulent toward Rvi6 hosts. These two populations, first diverged in allopatry, second came into contact and subsequently began interbreeding, in such way that they show unambiguous footprints of gene flow today. | NA | no | No good fitness measure and measure host GD | NA | no | no |
| 5352 | 2020 | 44040 | Mikalova, L; Janeckova, K; Novakova, M; Strouhal, M; Cejkova, D; Harper, KN; Smajs, D | 2020 | Whole genome sequence of the Treponema pallidum subsp. endemicum strain Iraq B: A subpopulation of bejel treponemes contains full-length tprF and tprG genes similar to those present in T. p. subsp. pertenue strains | Treponema pallidum subsp. endemicum (TEN) is the causative agent of endemic syphilis (bejel). Until now, only a single TEN strain, Bosnia A, has been completely sequenced. The only other laboratory TEN strain available, Iraq B, was isolated in Iraq in 1951 by researchers from the US Centers for Disease Control and Prevention. In this study, the complete genome of the Iraq B strain was amplified as overlapping PCR products and sequenced using the pooled segment genome sequencing method and Illumina sequencing. Total average genome sequencing coverage reached 3469x, with a total genome size of 1,137,653 bp. Compared to the genome sequence of Bosnia A, a set of 37 single nucleotide differences, 4 indels, 2 differences in the number of tandem repetitions, and 18 differences in the length of homopolymeric regions were found in the Iraq B genome. Moreover, the tprF and tprG genes that were previously found deleted in the genome of the TEN Bosnia A strain (spanning 2.3 kb in length) were present in a subpopulation of TEN Iraq B and Bosnia A microbes, and their sequence was highly similar to those found in T. p. subsp. pertenue strains, which cause the disease yaws. The genome sequence of TEN Iraq B revealed close genetic relatedness between both available bejel-causing laboratory strains (i.e., Iraq B and Bosnia A) and also genetic variability within the bejel treponemes comparable to that found within yaws- or syphilis-causing strains. In addition, genetic relatedness to TPE strains was demonstrated by the sequence of the tprF and tprG genes found in sub-populations of both TEN Iraq B and Bosnia A. The loss of the tprF and tprG genes in most TEN microbes suggest that TEN genomes have been evolving via the loss of genomic regions, a phenomenon previously found among the treponemes causing both syphilis and rabbit syphilis. | NA | no | No good fitness measure and measure host GD | NA | no | no |
| 5353 | 2020 | 44111 | Mikhaylova, AV; Thornton, TA | 2019 | Accuracy of Gene Expression Prediction From Genotype Data With PrediXcan Varies Across and Within Continental Populations | Using genetic data to predict gene expression has garnered significant attention in recent years. PrediXcan has become one of the most widely used gene-based methods for testing associations between predicted gene expression values and a phenotype, which has facilitated novel insights into the relationship between complex traits and the component of gene expression that can be attributed to genetic variation. The gene expression prediction models for PrediXcan were developed using supervised machine learning methods and training data from the Depression Genes and Networks (DGN) study and the Genotype-Tissue Expression (GTEx) project, where the majority of subjects are of European descent. Many genetic studies, however, include samples from multi-ethnic populations, and in this paper we evaluate the accuracy of PrediXcan for predicting gene expression in diverse populations. Using transcriptomic data from the GEUVADIS (Genetic European Variation in Disease) RNA sequencing project and whole genome sequencing data from the 1000 Genomes project, we evaluate and compare the predictive performance of PrediXcan in an African population (Yoruban) and four European ancestry populations for thousands of genes. We evaluate a range of models from the PrediXcan weight databases and use Pearson’s correlation coefficient to assess gene expression prediction accuracy with PrediXcan. From our evaluation, we find that the predictive performance of PrediXcan varies substantially among populations from different continents (F-test p-value < 2.2 x 10(-16)), where prediction accuracy is lower in the Yoruban population from West Africa compared to the European-ancestry populations. Moreover, not only do we find differences in predictive performance between populations from different continents, we also find highly significant differences in prediction accuracy among the four European ancestry populations considered (F-test p-value < 2.2 x 10(-16)). Finally, while there is variability in prediction accuracy across different PrediXcan weight databases, we also find consistency in the qualitative performance of PrediXcan for the five populations considered, with the African ancestry population having the lowest accuracy across databases. | NA | no | human | NA | no | no |
| 5354 | 2020 | 44113 | Miles, A; Harding, NJ; Botta, G; Clarkson, CS; Antao, T; Kozak, K; Schrider, DR; Kern, AD; Redmond, S; Sharakhov, I; Pearson, RD; Bergey, C; Fontaine, MC; Donnelly, MJ; Lawniczak, MKN; Kwiatkowski, DP; Ayala, D; Besensky, NJ; Burt, A; Caputo, B; della Torre, A; Fontaine, MC; Godfray, HCJ; Hahn, MW; Midega, J; Neafsey, DE; O’Loughlin, S; Pinto, J; Riehle, MM; Vernick, KD; Weetman, D; Wilding, CS; White, BJ; Troco, AD; Diabate, A; Costantini, C; Rohatgi, KR; Besansky, NJ; Elissa, N; Coulibaly, B; Dinis, J; Midegal, J; Mbogo, C; Bejon, P; Mawejje, HD; Stalker, J; Rockett, K; Drury, E; Mead, D; Jeffreys, A; Hubbard, C; Rowlands, K; Isaacs, AT; Jyothi, D; Malangone, C; Vauterin, P; Jeffery, B; Wright, I; Hart, L; Kluczyriski, K; Cornelius, V; MacInnisn, B; Henrichs, C; Giacomantonio, R | 2017 | Genetic diversity of the African malaria vector Anopheles gambiae | The sustainability of malaria control in Africa is threatened by the rise of insecticide resistance in Anopheles mosquitoes, which transmit the disease(1). To gain a deeper understanding of how mosquito populations are evolving, here we sequenced the genomes of 765 specimens of Anopheles gambiae and Anopheles coluzzii sampled from 15 locations across Africa, and identified over 50 million single nucleotide polymorphisms within the accessible genome. These data revealed complex population structure and patterns of gene flow, with evidence of ancient expansions, recent bottlenecks, and local variation in effective population size. Strong signals of recent selection were observed in insecticide-resistance genes, with several sweeps spreading over large geographical distances and between species. The design of new tools for mosquito control using gene-drive systems will need to take account of high levels of genetic diversity in natural mosquito populations. | NA | no | No good fitness measure and measure host GD | NA | no | no |
| 5355 | 2020 | 44113 | Minard, G; Tran, FH; Van, VT; Fournier, C; Potier, P; Roiz, D; Mavingui, P; Moro, CV | 2018 | Shared larval rearing environment, sex, female size and genetic diversity shape Ae. albopictus bacterial microbiota | The Asian tiger mosquito Aedes albopictus became of public health concern as it can replicate and transmit viral and filarial pathogens with a strong invasive success over the world. Various strategies have been proposed to reduce mosquito population’s vectorial capacity. Among them, symbiotic control of mosquito borne disease offers promising perspectives. Such method is likely to be affected by the dynamics of mosquito-associated symbiotic communities, which might in turn be affected by host genotype and environment. Our previous study suggested a correlation between mosquitoes’ origin, genetic diversity and midgut bacterial diversity. To distinguish the impact of those factors, we have been studying the midgut bacterial microbiota of two Ae. albopictus populations from tropical (La Re A union) and temperate (Montpellier) origins under controlled laboratory conditions. the two populations experienced random mating or genetic bottleneck. Microbiota composition did not highlight any variation of the alpha and beta-diversities in bacterial communities related to host’s populations. However, sizes of the mosquitoes were negatively correlated with the bacterial a-diversity of females. Variations in mosquito sex were associated with a shift in the composition of bacterial microbiota. The females’ mosquitoes also exhibited changes in the microbiota composition according to their size and after experiencing a reduction of their genetic diversity. These results provide a framework to investigate the impact of population dynamics on the symbiotic communities associated with the tiger mosquito. | NA | no | No good fitness measure | NA | no | no |
| 5356 | 2020 | 44113 | Minias, P; Bateson, ZW; Whittingham, LA; Johnson, JA; Oyler-McCance, S; Dunn, PO | 2018 | Extensive shared polymorphism at non-MHC immune genes in recently diverged North American prairie grouse | Gene polymorphisms shared between recently diverged species are thought to be widespread and most commonly reflect introgression from hybridization or retention of ancestral polymorphism through incomplete lineage sorting. Shared genetic diversity resulting from incomplete lineage sorting is usually maintained for a relatively short period of time, but under strong balancing selection it may persist for millions of years beyond species divergence (balanced trans-species polymorphism), as in the case of the major histocompatibility complex (MHC) genes. However, balancing selection is much less likely to act on non-MHC immune genes. The aim of this study was to investigate the patterns of shared polymorphism and selection at non-MHC immune genes in five grouse species from Centrocercus and Tympanuchus genera. For this purpose, we genotyped five non-MHC immune genes that do not interact directly with pathogens, but are involved in signaling and regulate immune cell growth. In contrast to previous studies with MHC, we found no evidence for balancing selection or balanced trans-species polymorphism among the non-MHC immune genes. No haplotypes were shared between genera and in most cases more similar allelic variants sorted by genus. Between species within genera, however, we found extensive shared polymorphism, which was most likely attributable to introgression or incomplete lineage sorting following recent divergence and large ancestral effective population size (i.e., weak genetic drift). Our study suggests that North American prairie grouse may have attained relatively low degree of reciprocal monophyly at nuclear loci and reinforces the rarity of balancing selection in non-MHC immune genes. | NA | no | mhc diversity | NA | no | no |
| 5357 | 2020 | 44040 | Mironenko, NV; Orina, AS; Kovalenko, NM | 2020 | Differences between Pyrenophora tritici-repentis Isolates in Expression of ToxA and PtrPf2 Genes in Culture (in vitro) | For the first time, relative expression levels of the ToxA effector gene and PtrPf2 transcription factor gene in vitro were used to characterize the collection of isolates of the causative agent of tan spot of wheat, Pyrenophora tritici-repentis, from geographically distant populations. It was demonstrated that two groups of isolates, from Kazakhstan (Almaty oblast) and Russia (Krasnodar krai), differed in the levels of genetic diversity. The group of Krasnodar isolates was more heterogenous in expression levels of both genes. In Krasnodar isolates, positive correlation between the expression levels of examined genes was revealed (r = 0.96, p < 0.001). The isolate groups were statistically significantly different in the PtrPf2 expression levels (p = 0.002). According to preliminary data, high virulence of Kazakhstan isolates was associated with high expression levels of the PtrPf2 transcription factor gene in these isolates. A supposition on the existence of association between the PtrPf2 expression level in vitro and the ability of isolates to induce necrosis on the leaves of a susceptible cultivar is made. | NA | no | No good fitness measure and measure host GD | NA | no | no |
| 5358 | 2020 | 44111 | Mirshahi, UL; Luo, JZ; Manickam, K; Wardeh, AH; Mirshahi, T; Murray, MF; Carey, DJ | 2019 | Trajectory of exonic variant discovery in a large clinical population: implications for variant curation | Purpose: Precision health initiatives and reduced sequencing costs are driving large-scale human genome analyses. Genetic variant curation is a bottleneck in clinical applications. The burden of variant curation can be high for newly discovered variants because they are less likely to have undergone previous clinical annotation; the rate of discovery of genetic variants in large clinical populations has not been empirically determined. Methods: We determined the rate of accrual of unique sequence variants in 90,000 exome sequences. Separate analyses were done for 17,267 autosomal genes and a subset of 74 actionable genes; the effect of relatedness in the cohort was also determined. Results: Variant discovery showed a nonlinear growth pattern. The rate of unique variant accrual decreased as the database size increased; by 90,000 exomes 97% of all projected coding and splicing variants had been observed. Variants in 74 actionable genes showed a similar pattern. Family relatedness slightly reduced the rate of discovery of unique variants. Conclusion: The heaviest burden of interpretation for genetic variants occurs early and diminishes as the database size increases. Our data provide a framework for scaling pathogenic genetic variant discovery and curation, a critical element of patient care in the era of precision health. | NA | no | No good fitness measure and measure host GD | NA | no | no |
| 5359 | 2020 | 44040 | Misaka, BC; Wosula, EN; Marchelo-d’Ragga, PW; Hvoslef-Eide, T; Legg, JP | 2020 | Genetic Diversity of Bemisia tabaci (Gennadius) (Hemiptera: Aleyrodidae) Colonizing Sweet Potato and Cassava in South Sudan | Bemisia tabaci (Gennadius) is a polyphagous, highly destructive pest that is capable of vectoring viruses in most agricultural crops. Currently, information regarding the distribution and genetic diversity of B. tabaci in South Sudan is not available. The objectives of this study were to investigate the genetic variability of B. tabaci infesting sweet potato and cassava in South Sudan. Field surveys were conducted between August 2017 and July and August 2018 in 10 locations in Juba County, Central Equatoria State, South Sudan. The sequences of mitochondrial DNA cytochrome oxidase I (mtCOI) were used to determine the phylogenetic relationships between sampled B. tabaci. Six distinct genetic groups of B. tabaci were identified, including three non-cassava haplotypes (Mediterranean (MED), Indian Ocean (IO), and Uganda) and three cassava haplotypes (Sub-Saharan Africa 1 sub-group 1 (SSA1-SG1), SSA1-SG3, and SSA2). MED predominated on sweet potato and SSA2 on cassava in all of the sampled locations. The Uganda haplotype was also widespread, occurring in five of the sampled locations. This study provides important information on the diversity of B. tabaci species in South Sudan. A comprehensive assessment of the genetic diversity, geographical distribution, population dynamics, and host range of B. tabaci species in South Sudan is vital for its effective management. | NA | no | No good fitness measure and measure host GD | NA | no | no |
| 5360 | 2020 | 44113 | Mita, T; Hombhanje, F; Takahashi, N; Sekihara, M; Yamauchi, M; Tsukahara, T; Kaneko, A; Endo, H; Ohashi, J | 2018 | Rapid selection of sulphadoxine-resistant Plasmodium falciparum and its effect on within-population genetic diversity in Papua New Guinea | The ability of the human malarial parasite Plasmodium falciparum to adapt to environmental changes depends considerably on its ability to maintain within-population genetic variation. Strong selection, consequent to widespread antimalarial drug usage, occasionally elicits a rapid expansion of drugresistant isolates, which can act as founders. To investigate whether this phenomenon induces a loss of within-population genetic variation, we performed a population genetic analysis on 302 P. falciparum cases detected during two cross-sectional surveys in 2002/2003, just after the official introduction of sulphadoxine/pyrimethamine as a first-line treatment, and again in 2010/2011, in highly endemic areas in Papua New Guinea. We found that a single-origin sulphadoxine-resistant parasite isolate rapidly increased from 0% in 2002/2003 to 54% in 2010 and 84% in 2011. However, a considerable number of pairs exhibited random associations among 10 neutral microsatellite markers located in various chromosomes, suggesting that outcrossing effectively reduced non-random associations, albeit at a low average multiplicity of infection (1.35-1.52). Within-population genetic diversity was maintained throughout the study period. This indicates that the parasites maintained within-population variation, even after a clonal expansion of drug-resistant parasites. Outcrossing played a role in the preservation of within-population genetic diversity despite low levels of multiplicity of infection. | NA | no | gd of pathogen | NA | no | no |
| 5361 | 2020 | 44039 | Moe, SJ; Nater, CR; Rustadbakken, A; Vollestad, LA; Lund, E; Qvenild, T; Hegge, O; Aass, P | 2020 | Long-term mark-recapture and growth data for large-sized migratory brown trout (Salmo trutta) from Lake Mjosa, Norway | Background Long-term data from marked animals provide a wealth of opportunities for studies with high relevance to both basic ecological understanding and successful management in a changing world. The key strength of such data is that they allow us to quantify individual variation in vital rates (e.g. survival, growth, reproduction) and then link it mechanistically to dynamics at the population level. However, maintaining the collection of individual-based data over long time periods comes with large logistic efforts and costs and studies spanning over decades are therefore rare. This is the case particularly for migratory aquatic species, many of which are in decline despite their high ecological, cultural and economical value. New information This paper describes two unique publicly available time series of individual-based data originating from a 51-year mark-recapture study of a land-locked population of large-sized migratory brown trout (Salmo trutta) in Norway: the Hunder trout. In the period 1966-2015, nearly 14,000 adult Hunder trout have been captured and individually marked during their spawning migration from Lake Mjosa to the river Gubrandsdalslagen. Almost a third of those individuals were later recaptured alive during a later spawning run and/or captured by fishermen and reported dead or alive. This has resulted in the first data series: a markrecapture-recovery dataset spanning half a century and more than 18,000 capture records. The second data series consists of additional data on juvenile and adult growth and lifehistory schedules from half of the marked individuals, obtained by means of scale-sample analysis. The two datasets offer a rare long-term perspective on individuals and population dynamics and provide unique opportunities to gain insights into questions surrounding management, conservation and restoration of migratory salmonid populations and freshwater ecosystems. | NA | no | no group level or good fitness | NA | no | no |
| 5362 | 2020 | 44111 | Mogashoa, T; Melamu, P; Ley, SD; Streicher, EM; Iketleng, T; Kelentse, N; Mupfumi, L; Mokomane, M; Kgwaadira, B; Novitsky, V; Kasvosve, I; Moyo, S; Warren, RM; Gaseitsiwe, S | 2019 | Genetic diversity of Mycobacterium tuberculosis strains circulating in Botswana | Background Molecular typing of Mycobacterium tuberculosis (M.tb) isolates can inform Tuberculosis (TB) control programs on the relative proportion of transmission driving the TB epidemic. There is limited data on the M.tb genotypes that are circulating in Botswana. The aim of this study was to generate baseline data on the genetic diversity of M.tb isolates circulating in the country. Methods A total of 461 M.tb isolates received at the Botswana National Tuberculosis Reference Laboratory between March 2012 and October 2013 were included in this study. Drug susceptibility testing was conducted using the BD BACTEC MGIT 960 System. M.tb strains were genotyped using spoligotyping and spoligotype patterns were compared with existing patterns in the SITVIT Web database. A subset of drug resistant isolates which formed spoligo clusters (n = 65) was additionally genotyped with 12-loci MIRU. Factors associated with drug resistance and clustering were evaluated using logistic regression. Results Of the 461 isolates genotyped, 458 showed 108 distinct spoligotype patterns. The predominant M.tb lineages were Lineage 4 (81.9%), Lineage 2 (9%) and Lineage 1 (7.2%). The predominant spoligotype families within Lineage 4 were LAM (33%), S (14%), T (16%), X (16%). Three hundred and ninety-two (86%) isolates could be grouped into 44 clusters (2-46 isolates per cluster); giving a clustering rate of 76%. We identified 173 (37.8%) drug resistant isolates, 48 (10.5%) of these were multi-drug resistant. MIRU typing of the drug resistant isolates allowed grouping of 46 isolates into 14 clusters, giving a clustering rate of 49.2%. There was no association between age, sex, treatment category, region and clustering. Conclusions This study highlights the complexity of the TB epidemic in Botswana with multiple strains contributing to disease and provides baseline data on the population structure of M.tb strains in Botswana. | NA | no | gd of pathogen | NA | no | no |
| 5363 | 2020 | 44112 | Mohammed, M; Jaiswal, SK; Dakora, FD | 2018 | Distribution and correlation between phylogeny and functional traits of cowpea (Vigna unguiculata L. Walp.)-nodulating microsymbionts from Ghana and South Africa | Cowpea (Vigna unguiculata L. Walp.) is indigenous to Africa, and highly valued for its N-2-fixing trait and the nutritional attributes of its grain and leaves. The species’ ability to establish effective symbiosis with diverse rhizobial populations gives it survival and growth advantage in N-limited environments. To explore the functional diversity and phylogenetic positions of rhizobia nodulating cowpea in Africa, nodules were collected from various cowpea varieties grown in soils from the Guinea savanna and Sudano-sahelian agroecologies of Northern Ghana, and from the lowveld and middleveld areas of Mpumalanga Province in South Africa. Box-PCR profiling and multilocus sequence analysis revealed the presence of diverse microsymbionts responsible for cowpea nodulation across the study sites. BOX-PCR amplifications yielded variable band sizes, ranging from 618 bp to 5354 bp, which placed the isolates in six major clusters (Cluster A-F). Phylogenetic analysis based on 16S rRNA, atpD, glnII, gyrB, rpoB, nifH and nodC genes revealed the presence of diverse Bradyrhizobium sp. closely related to Bradyrhizobium daqingense, Bradyrhizobium subterraneum, Bradyrhizobium yuanmingense, Bradyrhizobium embrapense, Bradyrhizobium pachyrhizi, Bradyrhizobium elkanii and novel Bradyrhizobium species in the soils studied, a finding that could be attributed to the unique edapho-climatic conditions of the contrasting environments. The test isolates exhibited distinct symbiotic efficiencies, and also induced variable (p <= 0.001) photosynthetic rates, leaf transpiration, total chlorophyll and shoot biomass accumulation on cowpea (their homologous host). Canonical correspondence analysis showed that the distribution of these microsymbionts was influenced by the concentrations of macro-and micronutrients in soils. The pairwise genetic distances derived from phylogenies and nodule functioning showed significant (p < 0.05) correlation, which suggests that local environmental factors played a major role in the cowpea-Bradyrhizobium symbiosis. | NA | no | dometicated host | NA | no | no |
| 5364 | 2020 | 44041 | Mojbafan, M; Tina, S; Motlagh, FZ; Surguchov, A; Nilipour, Y; Zeinali, S | 2019 | Genetic variability in Iranian limb-girdle muscular dystrophy type 2B patients: An evidence of a founder effect | Background Dysferlinopathies are a group of autosomal recessive limb-girdle muscular dystrophies (LGMDs) caused by mutations in DYSF (#603,009). This gene encodes a transmembrane protein called dysferlin. Since there are few reports on Iranian dysferlinopathy patients, we tried to identify the DYSF mutations in affected individuals of Iran. Methods Eight unrelated Iranian families have been selected for this study. Sanger sequencing followed by haplotype analysis was performed to identify individual variations in DYSF sequence. Identified variants were analyzed, and their pathogenicity was interpreted according to the recommendations of the American College of Medical Genetics and Genomics. Results We identified two new mutations in DYSF, the first one is a nonsense mutation c.2419C > T (p.Gln807\*), which eliminates downstream part of the protein. Another novel mutation is c. (1,053 + 1\_1,054-1)\_(1,397 + 1\_1,398-1)del, which causes deletion of the DNA segment from exon 12 to exon 15. Conclusion Two of the other six families are from the same ethnicity and share the same mutation and haplotype patterns, suggesting a founder mutation. Genetic analysis of dysferlinopathy can prevent a wrong diagnosis of myositis for these patients. | NA | no | human | NA | no | no |
| 5365 | 2020 | 44039 | Mola, JM; Miller, MR; O’Rourke, SM; Williams, NM | 2020 | Wildfire reveals transient changes to individual traits and population responses of a native bumble bee Bombus vosnesenskii | Fire-induced changes in the abundance and distribution of organisms, especially plants, can alter resource landscapes for mobile consumers driving bottom-up effects on their population sizes, morphologies and reproductive potential. We expect these impacts to be most striking for obligate visitors of plants, like bees and other pollinators, but these impacts can be difficult to interpret due to the limited information provided by forager counts in the absence of survival or fitness proxies. Increased bumble bee worker abundance is often coincident with the pulses of flowers that follow recent fire. However, it is unknown if observed postfire activity is due to underlying population growth or a stable pool of colonies recruiting more foragers to abundant resource patches. This distinction is necessary for determining the net impact of disturbance on bumble bees: are there population-wide responses or do just a few colonies reap the rewards? We estimated colony abundance before and after fire in burned and unburned areas using a genetic mark-recapture framework. We paired colony abundance estimates with measures of body size, counts of queens, and estimates of foraging and dispersal to assess changes in worker size, reproductive output, and landscape-scale movements. Higher floral abundance following fire not only increased forager abundance but also the number of colonies from which those foragers came. Importantly, despite a larger population size, we also observed increased mean worker size. Two years following fire, queen abundance was higher in both burned and unburned sites, potentially due to the dispersal of queens from burned into unburned areas. The effects of fire were transient; within two growing seasons, worker abundance was substantially reduced across the entire sampling area and body sizes were similar between burned and unburned sites. Our results reveal how disturbance can temporarily release populations from resource limitation, boosting the genetic diversity, body size, and reproductive output of populations. Given that the effects of fire on bumble bees acted indirectly through pulsed resource availability, it is likely our results are generalizable to other situations, such as habitat restorations, where resource density is enhanced within the landscape. | NA | no | No good fitness measure and measure host GD | NA | no | no |
| 5366 | 2020 | 44111 | Moller, M; Schotanus, K; Soyer, JL; Haueisen, J; Happ, K; Stralucke, M; Happel, P; Smith, KM; Connolly, LR; Freitag, M; Stukenbrock, EH | 2019 | Destabilization of chromosome structure by histone H3 lysine 27 methylation | Chromosome and genome stability are important for normal cell function as instability often correlates with disease and dysfunction of DNA repair mechanisms. Many organisms maintain supernumerary or accessory chromosomes that deviate from standard chromosomes. The pathogenic fungus Zymoseptoria tritici has as many as eight accessory chromosomes, which are highly unstable during meiosis and mitosis, transcriptionally repressed, show enrichment of repetitive elements, and enrichment with heterochromatic histone methylation marks, e.g., trimethylation of H3 lysine 9 or lysine 27 (H3K9me3, H3K27me3). To elucidate the role of heterochromatin on genome stability in Z. tritici, we deleted the genes encoding the methyltransferases responsible for H3K9me3 and H3K27me3, kmt1 and kmt6, respectively, and generated a double mutant. We combined experimental evolution and genomic analyses to determine the impact of these deletions on chromosome and genome stability, both in vitro and in planta. We used whole genome sequencing, ChIP-seq, and RNA-seq to compare changes in genome and chromatin structure, and differences in gene expression between mutant and wildtype strains. Analyses of genome and ChIP-seq data in H3K9me3-deficient strains revealed dramatic chromatin reorganization, where H3K27me3 is mostly relocalized into regions that are enriched with H3K9me3 in wild type. Many genome rearrangements and formation of new chromosomes were found in the absence of H3K9me3, accompanied by activation of transposable elements. In stark contrast, loss of H3K27me3 actually increased the stability of accessory chromosomes under normal growth conditions in vitro, even without large scale changes in gene activity. We conclude that H3K9me3 is important for the maintenance of genome stability because it disallows H3K27me3 in regions considered constitutive heterochromatin. In this system, H3K27me3 reduces the overall stability of accessory chromosomes, generating a metastable state for these quasi-essential regions of the genome. Author summary Genome and chromosome stability are essential to maintain normal cell function and viability. However, differences in genome and chromosome structure are frequently found in organisms that undergo rapid adaptation to changing environmental conditions, and in humans are often found in cancer cells. We study genome instability in a fungal pathogen that exhibits a high degree of genetic diversity. Regions that show extraordinary diversity in this pathogen are the transposon-rich accessory chromosomes, which contain few genes that are of unknown benefit to the organism but maintained in the population and thus considered quasi-essential. Accessory chromosomes in all fungi studied so far are enriched with markers for heterochromatin, namely trimethylation of H3 lysine 9 and 27 (H3K9me3, H3K27me3). We show that loss of these heterochromatin marks has strong but opposing effects on genome stability. While loss of the transposon-associated mark H3K9me3 destabilizes the entire genome, presence of H3K27me3 favors instability of accessory chromosomes. Our study provides insight into the relationship between chromatin and genome stability and why some regions are more susceptible to genetic diversity than others. | NA | no | No good fitness measure and measure host GD | NA | no | no |
| 5367 | 2020 | 44040 | Molotsi, AH; Dube, B; Cloete, SWP | 2020 | The Current Status of Indigenous Ovine Genetic Resources in Southern Africa and Future Sustainable Utilisation to Improve Livelihoods | Indigenous sheep play an important role in the livelihoods of communal farmers in the Southern Africa Development Community (SADC), and this underlines the need to curb the genetic erosion of these valuable resources. This contribution reports that the phenotypic performance and genetics gains of institutional and commercial sheep in Southern Africa are well recorded. In contrast, there is a dearth of knowledge as far as the performance and genetic gains of indigenous ovine genetic resources utilized by smallholder farmers are concerned. High levels of genetic diversity have been observed in exotic breeds, whereas low levels of genetic diversity were found in the Zulu and Namaqua Afrikaner breeds. Phenotypic measurements for indigenous resources include linear measurements indicative of size and reproduction for Zulu sheep. Lamb survival, reproduction and resistance to ticks of the indigenous, fat-tailed Namaqua Afrikaner sheep, as well as growth and reproduction have also been recorded for Sabi and Landim sheep. This review discusses ways to sustainably utilize ovine genetic resources, which includes the suggested implementation of structured breeding and conservation programs, marketing, improving feed resources, health and diseases, as well as gender and age issues. Clearly, there is ample room for further research and development as far as the performance and improvement of African indigenous sheep are concerned. | NA | no | No good fitness measure and measure host GD | NA | no | no |
| 5368 | 2020 | 44041 | Mondal, D; Dutta, S; Chakrabarty, U; Mallik, A; Mandal, N | 2019 | Development and characterization of white spot disease linked microsatellite DNA markers in Penaeus monodon, and their application to determine the population diversity, cluster and structure | Pathogens that are introduced suddenly to natural populations can potentially cause quick changes to the genetics and diversity of the host. In the past three decades, white spot syndrome virus (WSSV) has caused damaging epizootics in Penaeus monodon populations. In this study, we developed WSSV resistance- or susceptibility-linked microsatellite DNA markers, and their effectiveness was validated experimentally. WSSV-resistant marker linked retroelements and genes that may have an important role in WSSV-resistance phenomena were partially identified. Allelic data of 1,694 samples from nine distinct geographic locations in India were revealed that populations from Digha and Kochi were highly dispersed, and also showed higher genetic diversity, higher population diversity, and lower prevalence of disease resistance. A very high level of gene flow was observed within all populations and a very high level of genetic variation was present within populations. Two genetically admixture population clusters were estimated in nature. WSSV-resistance has a significant link with genetic diversity, population cluster and population diversity. Microsatellite marker analysis characterized genetic divergence, diversity and structure among wild populations. | NA | no | gd of pathogen | NA | no | no |
| 5369 | 2020 | 44039 | Mondal, M; Lawarde, A; Somasundaram, K | 2020 | Genomics of Indian SARS-CoV-2: Implications in genetic diversity, possible origin and spread of virus | World Health Organization (WHO) declared COVID-19 as a pandemic disease on 11 March 2020. Comparison of genome sequences from diverse locations allows us to identify the genetic diversity among viruses which would help in ascertaining viral virulence, disease pathogenicity, origin and spread of the SARS-CoV-2 between countries. The aim of this study is to determine the genetic diversity among Indian SARS-CoV-2 isolates. Initial examination of the phylogenetic data of SARS-CoV-2 genomes (n = 3123) from different continents deposited at GISAID (Global Initiative on Sharing All Influenza Data) revealed multiple origin for Indian isolates. An in-depth analysis of 558 viral genomes derived from samples representing countries from USA, Europe, China, East Asia, South Asia, Oceania, Middle East regions and India revealed that most Indian samples are divided into two clusters. A1 sub-cluster showed more similarity to Oceania and Kuwait samples, while A2 sub-cluster grouped with South Asian samples. In contrast, cluster B grouped with countries from Europe, Middle East and South Asia. Viral clade analysis of Indian samples revealed a high occurrence of G clade (D614G in spike protein; 37%), which is a European clade, followed by I clade (V378I in ORF1ab; 12%), which is an Oceania clade with samples having Iran connections. While A1 cluster is enriched with I clade, the cluster B is enriched with G clade type. Thus our study identifies that the Indian SARS-CoV-2 viruses are enriched with G and I clades in addition to 50% samples with unknown genetic variations. The potential origin to be countries mainly from Europe, Middle East Oceania and South Asia regions, which strongly imply the spread of virus through most travelled countries. The study also emphasizes the importance of pathogen genomics through phylogenetic analysis to discover viral genetic diversity and understand the viral transmission dynamics with eventual grasp on viral virulence and disease pathogenesis. | NA | no | Covid-19 , not relevate | NA | no | no |
| 5370 | 2020 | 44039 | Monroy-Perez, E; Ceron, AB; Cortes, LRG; Alonso, NN; Dominguez-Trejo, P; Hernandez-Jaimes, T; Bustos-Martinez, J; Hamdan-Partida, A; Jimenez, EAR; Vaca, S; Vaca-Paniagua, F; Paniagua-Contreras, GL | 2020 | Virulence gene transcription, phylogroups, and antibiotic resistance of cervico-vaginal pathogenicE.coliin Mexico | The pathogenicity ofEscherichia colistrains that cause cervico-vaginal infections (CVI) is due to the presence of several virulence genes. The objective of this study was to define the variability regarding the genotype of antibiotic resistance, the transcription profiles of virulence genes after in vitro infection of the vaginal cell line A431 and the phylogroup composition of a group of cervico-vaginalE.colistrains (CVEC). A total of 200E.colistrains isolated from Mexican women with CVI from two medical units of the Mexican Institute of Social Security were analysed.E.colistrains and antibiotic resistance genes were identified using conventional polymerase chain reaction (PCR), and phylogroups were identified using multiplex PCR. Virulence gene transcription was measured through reverse-transcriptase real-time PCR after infection of the vaginal cell line A431. The most common antibiotic resistance genes among the CVEC strains wereaac(3)II, TEM,dfrA1,sul1, andqnrA. The predominant phylogroup was B2. The genes most frequently transcribed in these strains werefimH,papC,irp2,iroN,kpsMTII,cnf1, andompT, mainly in CVEC strains isolated from chronic and occasional vaginal infections. The strains showed a large diversity of transcription of the virulence genes phenotype and antibiotic resistance genotype, especially in the strains of phylogroups, B2, A, and D. The strains formed 2 large clusters, which contained several subclusters. The genetic diversity of CVEC strains was high. These strains have a large number of transcription patterns of virulence genes, and one-third of them carry three to seven antibiotic resistance genes. | NA | no | No good measureGD of host | NA | no | no |
| 5371 | 2020 | 44039 | Montagu, A; Lee, TRC; Ujvari, B; McCarl, V; Evans, TA; Lo, N | 2020 | High numbers of unrelated reproductives in the Australian ‘higher’ termite Nasutitermes exitiosus (Blattodea: Termitidae) | Social insect colonies are among the most complex social organisations in nature, with reproductive and non-reproductive individuals co-ordinating to maintain the survival of the colony. Multiple reproductive schemes occur in social insects, from simple schemes with one founding reproductive pair, to more complex ones involving within-colony inbreeding and more than two unrelated reproductives. Colony breeding schemes and genetic structure remain understudied in termites, compared to the Hymenoptera. In this study, we performed the first genetic characterisation of the colony breeding structure of Nasutitermes exitiosus (Blattodea: Termitidae), an endemic Australian termite with a broad distribution across southern mainland Australia. We analysed the genetic structure of 60 N. exitiosus colonies from the Eastern part of its distribution, using microsatellites and mitochondrial sequence data. We found that most colonies were headed by one founding pair of reproductives, although some colonies exhibited a more complex breeding structure, including within-colony inbreeding and the presence of multiple unrelated reproductives. We found evidence for the presence of seven unrelated queens in one colony, to our knowledge, the highest number of unrelated queens yet found in a termite from the family Termitidae. We found some evidence for genetic isolation by distance, indicating that the species is a relatively poor disperser over long ranges. | NA | no | No good fitness measure | NA | no | no |
| 5372 | 2020 | 44040 | Montakhabi, MK; Bonjar, GHS; Talebi, R | 2020 | Mating Type Profiling and SSR-Based Genetic Diversity Analysis of Iranian Fusarium oxysporum f. sp. ciceri Causing Wilt in Chickpea | Chickpea wilt disease caused by Fusarium oxysporum f. sp. ciceri (FOC) is the most important soil borne disease of chickpea in Iran and many other chickpea growing zones worldwide. In this study, a total of 65 FOC isolates have been collected from West of Iran (Kurdistan and Kermanshah provinces) and characterized for pathogenicity, mating type alleles and genetic diversity analysis by genic- and EST-SSR molecular markers. All the FOC isolates showed virulent pattern and 86% of them were moderately virulent (21-50% of wilt damage). Mating type assay using specific PCR-based primers showed that 60% of FOC isolates possess MAT-1 allele and 40% possess MAT-2 allele. Genetic diversity of FOC isolates using 10 SSR markers revealed high genetic diversity within FOC populations with an average of 3 alleles per locus. Cluster analysis divided FOC isolates into four groups. Isolates from Kurdistan were grouped distinctly from those collected from Kermanshah province. Results of present study will be useful for breeders to design effective breeding strategies and developing necessary integrated management for Fusarium wilt in chickpea. (C) 2020 Friends Science Publishers | NA | no | gd of pathogen | NA | no | no |
| 5373 | 2020 | 44112 | Montalva, N; Adhikari, K; Liebert, A; Mendoza-Revilla, J; Flores, SV; Mace, R; Swallow, DM | 2019 | Adaptation to milking agropastoralism in Chilean goat herders and nutritional benefit of lactase persistence | The genetic trait of lactase persistence (LP) evolved as an adaptation to milking pastoralism in the Old World and is a well-known example of positive natural selection in humans. However, the specific mechanisms conferring this selective advantage are unknown. To understand the relationship between milk drinking, LP, growth, reproduction, and survival, communities of the Coquimbo Region in Chile, with recent adoption of milking agropastoralism, were used as a model population. DNA samples and data on stature, reproduction, and diet were collected from 451 participants. Lactose tolerance tests were done on 41 of them. The European -13,910*T (rs4988235) was the only LP causative variant found, showing strong association (99.6%) with LP phenotype. Models of associations of inferred LP status and milk consumption, with fertility, mortality, height, and weight were adjusted with measures of ancestry and relatedness to control for population structure. Although we found no statistically significant effect of LP on fertility, a significant effect (P = 0.002) was observed of LP on body mass index (BMI) in males and of BMI on fertility (P = 0.003). These results fail to support a causal relationship between LP and fertility yet suggest the idea of a nutritional advantage of LP. Furthermore, the proportion of European ancestry around the genetic region of -13,910*T is significantly higher (P = 0.008) than the proportion of European ancestry genome-wide, providing evidence of recent positive selection since European-Amerindian admixture. This signature was absent in nonpastoralist Latin American populations, supporting the hypothesis of specific adaptation to milking agropastoralism in the Coquimbo communities. | NA | no | human | NA | no | no |
| 5374 | 2020 | 44112 | Monteiro, L; Traunspurger, W; Roeleveld, K; Lynen, F; Moens, T | 2018 | Direct toxicity of the water-soluble fractions of a crude and a diesel-motor oil on the survival of free-living nematodes | The water-soluble fraction (WSF) of an oil contains different classes of petroleum hydrocarbons and, despite their low potential for bioaccumulation, these can be highly toxic to biota. Nematodes are usually the most abundant and species-rich group of metazoans in marine and freshwater sediments as well as terrestrial soils and have been proposed as excellent models to monitor pollution effects. The first aim of this study was to assess the direct effects of the WSFs of a crude and a diesel-motor oil on the survival of 12 free-living bacterivorous nematode species from marine, freshwater and soil environments, and belonging to diverse taxonomic groups. The second aim was to compare the responses of these twelve test species and assess their suitability as candidate alternatives for the common model species Caenorhabditis elegans in future toxicity testing. The third and final aim was to test the common assumption that nematodes which are phylogenetically more closely related would exhibit more similar sensitivities than more distantly related species, and that - as is commonly stated in literature nematodes belonging to the family Rhabditidae would be the most pollution-tolerant species. While the crude oil was a complex matrix of substances, containing many soluble compounds, the diesel-motor oil 10W40 was characterized by only few soluble substances. Nevertheless, the diesel-motor oil WSF was as toxic (or even more toxic) to some of the tested nematode species as the crude-oil WSF. This could be linked to differences in compounds interactions in each oil-WSF, or to the presence of fuel additives in the 10W40-WSF. Most species exhibited moderate to extreme mortality levels in oil treatments, and experienced an increased mortality with time. Overall, marine nematodes were more sensitive than freshwater/soil organisms, albeit with some exceptions. Species sensitivities to oil did not follow patterns of taxonomic relatedness, contradicting the idea that closely related species should intrinsically respond similarly to pollution. Rhabditidae were not generally more sensitive than other species: out of 6 species of Rhabditidae tested, only Bursilla monhystera was highly tolerant, while C. elegans and cryptic species of Litoditis marina were among the most sensitive taxa. Therefore, we recommend that future effect studies do not focus on a single model species but instead incorporate multiple species for a better and more robust assessment of pollutant effects. | NA | no | No good fitness measure and measure host GD | NA | no | no |
| 5375 | 2020 | 44113 | Moolhuijzen, P; See, PT; Hane, JK; Shi, GJ; Liu, ZH; Oliver, RP; Moffat, CS | 2018 | Comparative genomics of the wheat fungal pathogen Pyrenophora tritici-repentis reveals chromosomal variations and genome plasticity | Background: Pyrenophora tritici-repentis (Ptr) is a necrotrophic fungal pathogen that causes the major wheat disease, tan spot. We set out to provide essential genomics-based resources in order to better understand the pathogenicity mechanisms of this important pathogen. Results: Here, we present eight new Ptr isolate genomes, assembled and annotated; representing races 1, 2 and 5, and a new race. We report a high quality Ptr reference genome, sequenced by PacBio technology with Illumina paired-end data support and optical mapping. An estimated 98% of the genome coverage was mapped to 10 chromosomal groups, using a two-enzyme hybrid approach. The final reference genome was 40.9 Mb and contained a total of 13,797 annotated genes, supported by transcriptomic and proteogenomics data sets. Conclusions: Whole genome comparative analysis revealed major chromosomal segmental rearrangements and fusions, highlighting intraspecific genome plasticity in this species. Furthermore, the Ptr race classification was not supported at the whole genome level, as phylogenetic analysis did not cluster the ToxA producing isolates. This expansion of available Ptr genomics resources will directly facilitate research aimed at controlling tan spot disease. | NA | no | gd of pathogen | NA | no | no |
| 5376 | 2020 | 44112 | Moraes, MA; Kubota, TYK; Rossini, BC; Marino, CL; Freitas, MLM; Moraes, MLT; da Silva, AM; Cambuim, J; Sebbenn, AM | 2018 | Long-distance pollen and seed dispersal and inbreeding depression in Hymenaea stigonocarpa (Fabaceae: Caesalpinioideae) in the Brazilian savannah | Hymenaea stigonocarpa is a neotropical tree that is economically important due to its high-quality wood; however, because it has been exploited extensively, it is currently considered threatened. Microsatellite loci were used to investigate the pollen and seed dispersal, mating patterns, spatial genetic structure (SGS), genetic diversity, and inbreeding depression in H.stigonocarpa adults, juveniles, and open-pollinated seeds, which were sampled from isolated trees in a pasture and trees within a forest fragment in the Brazilian savannah. We found that the species presented a mixed mating system, with population and individual variations in the outcrossing rate (0.53-1.0). The studied populations were not genetically isolated due to pollen and seed flow between the studied populations and between the populations and individuals located outside of the study area. Pollen and seed dispersal occurred over long distances (>8km); however, the dispersal patterns were isolated by distance, with a high frequency of mating occurring between near-neighbor trees and seeds dispersed near the parent trees. The correlated mating for individual seed trees was higher within than among fruits, indicating that fruits present a high proportion of full-sibs. Genetic diversity and SGS were similar among the populations, but offspring showed evidence of inbreeding, mainly originating from mating among related trees, which suggests inbreeding depression between the seed and adult stages. Selfing resulted in a higher inbreeding depression than mating among relatives, as assessed through survival and height. As the populations are not genetically isolated, both are important targets for in situ conservation to maintain their genetic diversity; for ex situ conservation, seeds can be collected from at least 78 trees in both populations separated by at least 250m. | NA | no | inbreeding | NA | no | no |
| 5377 | 2020 | 44040 | Morales-Arce, AY; Harris, RB; Stone, AC; Jensen, JD | 2020 | Evaluating the contributions of purifying selection and progeny-skew in dictating within-host Mycobacterium tuberculosis evolution | The within-host evolutionary dynamics of tuberculosis (TB) remain unclear, and underlying biological characteristics render standard population genetic approaches based upon the Wright-Fisher model largely inappropriate. In addition, the compact genome combined with an absence of recombination is expected to result in strong purifying selection effects. Thus, it is imperative to establish a biologically relevant evolutionary framework incorporating these factors in order to enable an accurate study of this important human pathogen. Further, such a model is critical for inferring fundamental evolutionary parameters related to patient treatment, including mutation rates and the severity of infection bottlenecks. We here implement such a model and infer the underlying evolutionary parameters governing within-patient evolutionary dynamics. Results demonstrate that the progeny skew associated with the clonal nature of TB severely reduces genetic diversity and that the neglect of this parameter in previous studies has led to significant mis-inference of mutation rates. As such, our results suggest an underlying de novo mutation rate that is considerably faster than previously inferred, and a progeny distribution differing significantly from Wright-Fisher assumptions. This inference represents a more appropriate evolutionary null model, against which the periodic effects of positive selection, associated with drug-resistance for example, may be better assessed. | NA | no | No good fitness measure and measure host GD | NA | no | no |
| 5378 | 2020 | 44039 | Morales-Gonzalez, A; Ruiz-Villar, H; Ordiz, A; Penteriani, V | 2020 | Large carnivores living alongside humans: Brown bears in human-modified landscapes | Expansion of human activities into large carnivore habitats and of large carnivore ranges into anthropogenic settings increase the potential for human-wildlife conflicts. Future carnivore survival and recovery depend on both their ability to adapt to human-modified landscapes and the application of adequate conservation strategies. We review human-related factors that negatively affect brown bears inhabiting human-modified landscapes, aiming to improve human-bear coexistence. Brown bears have triggered much research and a review on this model species should be useful for the conservation-oriented management of many large carnivores. In human-modified landscapes, main threats to bear populations are human settlements, habituation and availability of anthropogenic food, density and traffic load of roads and railways, and recreational and industrial activities. Main effects of coexistence with humans for bears are: increased disturbance, human-bear conflicts and human-caused mortality; behavioural alterations; reduced fitness and genetic diversity; and physiological alterations. To promote bear-human coexistence in human-modified landscapes, we identified nine key elements: reduction of human-induced mortality and use of scientific information for better assessment of new infrastructures; improve education on waste management and bear deterrence methods; safeguard and restore habitat connectivity; mitigate road effects and restrict motorized trail use; adjust viewing activity practices to local conservation status of bear populations and food availability; implement mitigation measures to minimize risk of human-bear encounters; quantify empirically the effects of recreational activities on the energetics and fitness of bears; better dissemination of scientific results and management guidelines; and further research on behavioural reactions of bears to different management regimes and persecution histories. (C) 2020 The Authors. Published by Elsevier B.V. | NA | no | No good fitness measure and measure host GD | NA | no | no |
| 5379 | 2020 | 44113 | Morcicek, B; Taskin, BG; Dogac, E; Dogaroglu, T; Taskin, V | 2018 | Evidence of natural Wolbachia infections and molecular identification of field populations of Culex pipiens complex (Diptera: Culicidae) mosquitoes in western Turkey | Establishing reliable risk projection information about the distribution pattern of members of the Culex pipiens complex is of particular interest, as these mosquitoes are competent vectors for certain disease-causing pathogens. Wolbachia, a maternally inherited bacterial symbiont, are distributed in various arthropod species and can induce cytoplasmic incompatibility, i.e., reduced egg hatch, in certain crosses. It is being considered as a tool for population control of mosquito disease vectors. The Aegean region is characterized by highly populated, rural, and agricultural areas and is also on the route of the migratory birds. In this study, a fragment of the 658 bp of the mitochondrial cytochrome c oxidase subunit 1 (COI) gene, which includes the barcode region, was employed to differentiate Cx. pipiens complex species found in this region. Moreover, for the first time, the prevalence of Wolbachia endobacteria in these natural populations was examined using PCR amplification of a specific wsp gene. Our results revealed a widespread (more than 90%, n=121) presence of the highly efficient West Nile virus vector Cx. quinquefasciatus in the region. We also found that Wolbachia infection is widespread; the average prevalence was 62% in populations throughout the region. This study provided valuable information about the composition of Cx. pipiens complex mosquitoes and the prevalence of Wolbachia infection in these populations in the Aegean region. This information will be helpful in tracking mosquito-borne diseases and designing and implementing Wolbachia-based control strategies in the region. | NA | no | No good fitness measure and measure host GD | NA | no | no |
| 5380 | 2020 | 44111 | Moreira, JCD; Rodrigues, DM; Gilioli, R; Costa, FF; Albuquerque, DM | 2019 | A semi-nested RT-PCR assay for detection of norovirus in rat fecal samples | Norovirus is a highly prevalent pathogen that can infect a wide range of host species. Thus far, there have only been two reports of norovirus infection in rats. Diagnostic assays for the detection of norovirus are well established, but a specific molecular assay for the diagnosis of norovirus infection in laboratory rats has not yet been reported. In this study, we describe the development of a sensitive, semi-nested RT-PCR assay for detection of norovirus in fecal samples from Rattus norvegicus, reared in animal facilities under different sanitary barrier conditions. Additionally, we describe the first report of the presence of norovirus in rat colonies from Brazilian animal facilities. | NA | no | No good fitness measure and measure host GD | NA | no | no |
| 5381 | 2020 | 44040 | Morenikeji, OB; Capria, AL; Ojurongbe, O; Thomas, BN | 2020 | SNP Diversity in CD14 Gene Promoter Suggests Adaptation Footprints in Trypanosome Tolerant N’Dama (Bos taurus) but not in Susceptible White Fulani (Bos indicus) Cattle | Immune response to infections has been shown to be mediated by genetic diversity in pattern recognition receptors, leading to disease tolerance or susceptibility. We elucidated naturally occurring variations within the bovine CD14 gene promoter in trypanosome-tolerant (N’Dama) and susceptible (White Fulani) cattle, with genomic and computational approaches. Blood samples were collected from White Fulani and N’Dama cattle, genomic DNA extracted and the entire promoter region of the CD14 gene amplified by PCR. We sequenced this region and performed in silico computation to identify SNP variants, transcription factor binding sites, as well as micro RNAs in the region. CD14 promoter sequences were compared with the reference bovine genome from the Ensembl database to identify various SNPs. Furthermore, we validated three selected N’Dama specific SNPs using custom Taqman SNP genotyping assay for genetic diversity. In all, we identified a total of 54 and 41 SNPs at the CD14 promoter for N’Dama and White Fulani respectively, including 13 unique SNPs present in N’Dama only. The significantly higher SNP density at the CD14 gene promoter region in N’Dama may be responsible for disease tolerance, possibly an evolutionary adaptation. Our genotype analysis of the three loci selected for validation show that mutant alleles (A/A, C/C, and A/A) were adaptation profiles within disease tolerant N’Dama. A similar observation was made for our haplotype analysis revealing that haplotypes H1 (ACA) and H2 (ACG) were significant combinations within the population. The SNP effect prediction revealed 101 and 89 new transcription factor binding sites in N’Dama and White Fulani, respectively. We conclude that disease tolerant N’Dama possessing higher SNP density at the CD14 gene promoter and the preponderance of mutant alleles potentially confirms the significance of this promoter in immune response, which is lacking in susceptible White Fulani. We, therefore, recommend further in vitro and in vivo study of this observation in infected animals, as the next step for understanding genetic diversity relating to varying disease phenotypes in both breeds. | NA | no | No good fitness measure and measure host GD | NA | no | no |
| 5382 | 2020 | 44112 | Morgan, EJ; Sutton, TL; Darwell, CT; Cook, JM | 2018 | Restructuring of a mutualism following introduction of Australian fig trees and pollinating wasps to Europe and the USA | Figs and fig-pollinating wasps are obligate mutualists that require each other to complete sexual reproduction. However, landscapers can establish populations of fig trees outside their native ranges by propagation through exported seeds, seedlings or cuttings. Once mature, these trees could be colonized by pollinating wasps and/or various non-pollinating wasps that also develop in figs. In recent decades, the Australian endemic Ficus rubiginosa has been planted widely in the Mediterranean region and in parts of the USA. Observation of ripe fruit production suggested that a pollination mutualism has been re-established by pollinating wasps colonizing trees in the plant’s introduced range. We therefore used sampling of pollinators from mainland Spain, Tenerife and California (USA) and molecular studies to characterize the restructured mutualism and compare it with the native range. In the native range, the plant is pollinated by five wasp species that form the Pleistodontes imperialis complex. However, all wasps in the introduced ranges belonged to just one of these species (P. imperialis sp. 1). Moreover, their mtDNA diversity was close to zero and the sequences clearly link them with the native southern population of this species. None of the>20 non-pollinating wasp species from the native range were found in the introduced ranges. In summary, the restructured mutualism has been dramatically simplified, lacking all non-pollinating wasps and all but one pollinator species from the native range. Moreover, the one pollinator species to establish successfully shows a drastic reduction in genetic diversity relative to its source population. | NA | no | No good fitness measure and measure host GD | NA | no | no |
| 5383 | 2020 | 44112 | Morgan, RA; Karl, JA; Bussan, HE; Heimbruch, KE; O’Connor, DH; Dudley, DM | 2018 | Restricted MHC class I A locus diversity in olive and hybrid olive/yellow baboons from the Southwest National Primate Research Center | Baboons are valuable models for complex human diseases due to their genetic and physiologic similarities to humans. Deep sequencing methods to characterize full-length major histocompatibility complex (MHC) class I (MHC-I) alleles in different nonhuman primate populations were used to identify novel MHC-I alleles in baboons. We combined data from Illumina MiSeq sequencing and Roche/454 sequencing to characterize novel full-length MHC-I transcripts in a cohort of olive and hybrid olive/yellow baboons from the Southwest National Primate Research Center (SNPRC). We characterized 57 novel full-length alleles from 24 baboons and found limited genetic diversity at the MHC-I A locus, with significant sharing of two MHC-I A lineages between 22 out of the 24 animals characterized. These shared alleles provide the basis for development of tools such as MHC:peptide tetramers for studying cellular immune responses in this important animal model. | NA | no | mhc diversity | NA | no | no |
| 5384 | 2020 | 44113 | Morgounov, A; Abugalieva, A; Akan, K; Akin, B; Baenziger, S; Bhatta, M; Dababat, AA; Demir, L; Dutbayev, Y; El Bouhssini, M; Erginbas-Orakci, G; Kishii, M; Keser, M; Koc, E; Kurespek, A; Mujeeb-Kazi, A; Yorgancilar, A; Ozdemir, F; Ozturk, I; Payne, T; Qadimaliyeva, G; Shamanin, V; Subasi, K; Suleymanova, G; Yakisir, E; Zelenskiy, Y | 2018 | High-yielding winter synthetic hexaploid wheats resistant to multiple diseases and pests | Development of winter wheat (Triticum aestivum) synthetics started at CIMMYT-Mexico in 2004, when winter durum wheat (Triticum turgidum) germplasm from Ukraine and Romania was crossed with Aegilops tauschii accessions from the Caspian Sea region. Chromosomes were doubled after pollination and embryo rescue, but chromosome number and cytological validation was not performed. F-2 populations were grown in Mexico and were shipped to Turkey in 2008. During 2009-2015, these populations were subjected to rigorous pedigree selection under dry, cold, disease-affected environments of the Central Anatolian Plateau. The wide segregation and partial sterility observed in 2009 gradually decreased and, by 2016, most of the F-8 single spike progenies demonstrated good fertility and agronomic performance. Since 2013, lines have been selected from synthetic populations and evaluated at multiple sites. Superior lines were characterized for resistance to leaf, stripe and stem rust, plant height, and reaction to common bunt and soil-borne pathogens. Thousand kernel weight of many lines exceeded 50 g, compared with the check varieties that barely reached 40 g. Threshability of synthetic lines varied from 0 to 95%, demonstrating genetic variation for this important domestication trait. Screening against Hessian fly, sunny pest and Russian wheat aphid identified several resistant genotypes. Both durum and Aegilops parents affected synthetic wheat traits. Several studies are underway to reveal the genetic diversity of synthetic lines and the basis of resistance to diseases and insects. This synthetic germplasm represents a new winter bread wheat parental pool. It is available upon request to interested breeding/research programmes. | NA | no | dometicated host | NA | no | no |
| 5385 | 2020 | 44111 | Morikawa, MK; Palumbi, SR | 2019 | Using naturally occurring climate resilient corals to construct bleaching-resistant nurseries | Ecological restoration of forests, meadows, reefs, or other foundational ecosystems during climate change depends on the discovery and use of individuals able to withstand future conditions. For coral reefs, climate-tolerant corals might not remain tolerant in different environments because of widespread environmental adjustment of coral physiology and symbionts. Here, we test if parent corals retain their heat tolerance in nursery settings, if simple proxies predict successful colonies, and if heat-tolerant corals suffer lower growth or survival in normal settings. Before the 2015 natural bleaching event in American Samoa, we set out 800 coral fragments from 80 colonies of four species selected by prior tests to have a range of intraspecific natural heat tolerance. After the event, nursery stock from heat-tolerant parents showed two to three times less bleaching across species than nursery stock from less tolerant parents. They also retained higher individual genetic diversity through the bleaching event than did less heat-tolerant corals. The three best proxies for thermal tolerance were response to experimental heat stress, location on the reef, and thermal microclimate. Molecular biomarkers were also predictive but were highly species specific. Colony genotype and symbiont genus played a similarly strong role in predicting bleaching. Combined, our results show that selecting for host and symbiont resilience produced a multispecies coral nursery that withstood multiple bleaching events, that proxies for thermal tolerance in restoration can work across species and be inexpensive, and that different coral clones within species reacted very differently to bleaching. | NA | no | No good fitness measure and measure host GD | NA | no | no |
| 5386 | 2020 | 44113 | Morin, RS; Gottschalk, KW; Ostry, ME; Liebhold, AM | 2018 | Regional patterns of declining butternut (Juglans cinerea L.) suggest site characteristics for restoration | Butternut trees dying from a canker disease were first reported in southwestern Wisconsin in 1967. Since then, the disease has caused extensive mortality of butternut throughout its North American range. The objectives of this study were to quantify changes in butternut populations and density across its range and identify habitat characteristics of sites where butternut is surviving in order to locate regions for potential butternut restoration. The natural range of butternut (Juglans cinerea L.) extends over a large region of eastern N. America encompassing New Brunswick south to North Carolina, north to Minnesota, and southwest to Missouri. Despite the species’ large range, it is typically not a common tree, comprising a relatively minor component of several different forest types. We evaluated change in butternut abundance and volume from current and historic data from 21 states in the eastern United States. We related abundance and volume at two time periods to a suite of ecological and site factors in order to characterize site conditions where butternut survived. We also assessed the current level of butternut mortality across its range. Since the 1980s, the number of butternut trees and butternut volume have decreased by 58% and 44%, respectively, across its US range. Substantial relative decreases in tree numbers and volume occurred in most ecoregion sections. Five environmental variables were found to be significant predictors of butternut presence. The potential impacts of butternut canker are particularly acute as the canker pathogen invasion pushes a rare tree species toward extinction, at least at a local scale. Based on the results presented here, large-diameter maple/beech/birch stands in dry, upland sites in eastern Minnesota, western Wisconsin, and upstate New York appear to offer the most favorable conditions for butternut growth and survival and thus may be the best stands for planting resistant butternut trees. | NA | no | No good fitness measure and measure host GD | NA | no | no |
| 5387 | 2020 | 44039 | Mosley, JD; Levinson, RT; Farber-Eger, E; Edwards, TL; Hellwege, JN; Hung, AM; Giri, A; Shuey, MM; Shaffer, CM; Shi, MJ; Brittain, EL; Chung, WK; Kullo, IJ; Arruda-Olson, AM; Jarvik, GP; Larson, EB; Crosslin, DR; Williams, MS; Borthwick, KM; Hakonarson, H; Denny, JC; Wang, TJ; Stein, CM; Roden, DM; Wells, QS | 2020 | The polygenic architecture of left ventricular mass mirrors the clinical epidemiology | Left ventricular (LV) mass is a prognostic biomarker for incident heart disease and all-cause mortality. Large-scale genome-wide association studies have identified few SNPs associated with LV mass. We hypothesized that a polygenic discovery approach using LV mass measurements made in a clinical population would identify risk factors and diseases associated with adverse LV remodeling. We developed a polygenic single nucleotide polymorphism-based predictor of LV mass in 7,601 individuals with LV mass measurements made during routine clinical care. We tested for associations between this predictor and 894 clinical diagnoses measured in 58,838 unrelated genotyped individuals. There were 29 clinical phenotypes associated with the LV mass genetic predictor at FDR q<0.05. Genetically predicted higher LV mass was associated with modifiable cardiac risk factors, diagnoses related to organ dysfunction and conditions associated with abnormal cardiac structure including heart failure and atrial fibrillation. Secondary analyses using polygenic predictors confirmed a significant association between higher LV mass and body mass index and, in men, associations with coronary atherosclerosis and systolic blood pressure. In summary, these analyses show that LV mass-associated genetic variability associates with diagnoses of cardiac diseases and with modifiable risk factors which contribute to these diseases. | NA | no | human | NA | no | no |
| 5388 | 2020 | 44111 | Mountford, HS; Villanueva, P; Fernandez, MA; De Barbieri, Z; Cazier, JB; Newbury, DF | 2019 | Candidate gene variant effects on language disorders in Robinson Crusoe Island | Background: Robinson Crusoe Island is a geographically and socially isolated settlement located over 600 km west of the Port of Valpariso, Chile. An unusually high incidence (30%) of the Chilean equivalent of developmental language disorder (in Spanish, trastorno especifico de lenguaje (TEL)), has been reported in Islander children, with 90% of these affected children found to be direct descendants of a pair of original founder-brothers, therefore strongly suggesting a shared genetic basis. Aim: This study reports a comprehensive examination of 34 genes that have been previously directly implicated in language-related mechanisms. It utilises whole-genome sequencing to investigate potential underlying variants in seven TEL affected and 10 unaffected islanders. The aim was to identify the underlying genetic cause of the TEL phenotype under two inheritance model paradigms; Mendelian monogenic and complex susceptibility. Subjects and methods: A targeted candidate gene approach was used to look for rare, shared variants that may underlie the diagnosis of TEL in a Mendelian genetic model. This study tested whether an overall burden of rare variants is enriched in individuals affected by TEL or with Islanders related to the founder-brother lineage. It further examined if any variants segregate with affection status or with founder-brother-related status and, therefore, may increase risk of developing a language disorder as part of a complex model. Finally, gene-based tests were performed to evaluate relationships between combined variation across candidate genes and TEL affection status. Results: No single pathogenic rare variant segregated with either affection or founder-related status within the 34 candidate genes. Additionally, no evidence was found of an overall increased variant burden in TEL individuals compared to those with TLD. Gene-based analysis found no clear association between the combined effects of variants across the 34 genes and affection status or founder-brother-relatedness. Conclusion: The high prevalence of language disorders found on Robinson Crusoe Island is not caused by either a shared high-impact variant, or an increased burden of variants within candidate genes previously implicated in language disorders. We have comprehensively tested for ‘low hanging fruit’ in genes implicated in language disorders. Therefore, the underlying cause of TEL on Robinson Crusoe lies outside of these known language disorder genes, or within a complex susceptibility model. | NA | no | No good fitness measure and measure host GD | NA | no | no |
| 5389 | 2020 | 44113 | Mueller, JC; Edelaar, P; Banos-Villalba, A; Carrete, M; Potti, J; Blas, J; Tella, JL; Kempenaers, B | 2017 | Selection on a behaviour-related gene during the first stages of the biological invasion pathway | Human-induced biological invasions are common worldwide and often have negative impacts on wildlife and human societies. Several studies have shown evidence for selection on invaders after introduction to the new range. However, selective processes already acting prior to introduction have been largely neglected. Here, we tested whether such early selection acts on known behaviour-related gene variants in the yellow-crowned bishop (Euplectes afer), a pet-traded African songbird. We tested for nonrandom allele frequency changes after trapping, acclimation and survival in captivity. We also compared the native source population with two independent invasive populations. Allele frequencies of two SNPs in the dopamine receptor D4 (DRD4) gene-known to be linked to behavioural activity in response to novelty in this species-significantly changed over all early invasion stages. They also differed between the African native population and the two invading European populations. The two-locus genotype associated with reduced activity declined consistently, but strongest at the trapping stage. Overall genetic diversity did not substantially decrease, and there is little evidence for new alleles in the introduced populations, indicating that selection at the DRD4 gene predominantly worked on the standing genetic variation already present in the native population. Our study demonstrates selection on a behaviour-related gene during the first stages of a biological invasion. Thus, pre-establishment stages of a biological invasion do not only determine the number of propagules that are introduced (their quantity), but also their phenotypic and genetic characteristics (their quality). | NA | no | No good fitness measure | NA | no | no |
| 5390 | 2020 | 44040 | Mueller, SA; Reiners, TE; Middelhoff, TL; Anders, O; Kasperkiewicz, A; Nowak, C | 2020 | The rise of a large carnivore population in Central Europe: genetic evaluation of lynx reintroduction in the Harz Mountains | Large carnivores have made a successful comeback across human-dominated landscapes in Central Europe. The Eurasian lynx, for instance, has been actively reintroduced in different regions. Genetic diversity is quickly eroding in these isolated, small populations, questioning the long-term success of lynx reintroductions. To track population development and genetic diversity in a reintroduced lynx population, we used microsatellite analysis and mtDNA haplotyping based on 379 samples collected during the initial 15 year period of lynx reintroduction in the Harz mountains National Park, Germany. The Harz lynx population shows higher genetic diversity relative to other lynx reintroductions, due to initial cross-breeding of divergent captive source lineages and a comparably high founder size. While the population shows significant population growth and spread into adjacent regions, genetic diversity is continiously declining. Expected heterozygosity values dropped from 0.63 after reintroduction (2006/2007) to 0.55 within a 10 year period. Despite this, the Harz lynx population is currently a viable component to an envisioned lynx metapopulation spanning across Central Europe. The ongoing genetic erosion in the Harz population along with a lack of geneflow from adjacent populations indicates that such connectivity is urgently needed to ensure long-term population persistence. | NA | no | No group level | NA | no | no |
| 5391 | 2020 | 44113 | Mugerwa, H; Seal, S; Wang, HL; Patel, MV; Kabaalu, R; Omongo, CA; Alicai, T; Tairo, F; Ndunguru, J; Sseruwagi, P; Colvin, J | 2018 | African ancestry of New World, Bemisia tabaci-whitefly species | Bemisia tabaci whitefly species are some of the world’s most devastating agricultural pests and plant-virus disease vectors. Elucidation of the phylogenetic relationships in the group is the basis for understanding their evolution, biogeography, gene-functions and development of novel control technologies. We report here the discovery of five new Sub-Saharan Africa (SSA) B. tabaci putative species, using the partial mitochondrial cytochrome oxidase 1 gene: SSA9, SSA10, SSA11, SSA12 and SSA13. Two of them, SSA10 and SSA11 clustered with the New World species and shared 84.8-86.5% sequence identities. SSA10 and SSA11 provide new evidence for a close evolutionary link between the Old and New World species. Re-analysis of the evolutionary history of B. tabaci species group indicates that the new African species (SSA10 and SSA11) diverged from the New World clade c. 25 million years ago. The new putative species enable us to: (i) re-evaluate current models of B. tabaci evolution, (ii) recognise increased diversity within this cryptic species group and (iii) re-estimate divergence dates in evolutionary time. | NA | no | gd of pathogen | NA | no | no |
| 5392 | 2020 | 44113 | Muhammad, S; Ahmad, A; Awan, FS; Khan, AI; Qasim, M; Aziz-Ur-Rehman; Rehman, A; Javed, MA; Manzoor, I; Sajjad, M | 2018 | Genome Wide Association Analysis for Leaf Rust Resistance in Spring Wheat (Triticum aestivum) Germplasm | Leaf rust caused by Puccinia triticina, is an important disease of wheat in Pakistan and many other wheat growing countries. Seventy six leaf rust resistance genes have been cataloged based on genetic analysis in different mapping populations. A majority of these genes belong to all stage resistance category. Only a small proportion of these genes are currently effective against P. triticina pathotypes of Pakistan. A set of 94 wheat genotypes was scored for adult plant leaf rust response variation under field conditions in Faisalabad, Pakistan during the 2011 and 2012 crop seasons and was genotyped by using 203 microsatellite markers covering all 21 wheat chromosomes. Marker-leaf rust associations were determined using single marker tests. The most important genomic regions for leaf rust resistance were IDS, 4AL, 4BL, 5AS, 5BS, 6AL, 6BS, 6DS. Different loci that were not mapped in previous studied were tagged to different chromosomal locations thus the tagged loci may be further studied for identification and designation of new genes to different chromosomes. This will help the wheat breeders to use this information for the development of new resistant cultivars against wheat leaf rust via utilizing marker assisted selection. (C) 2018 Friends Science Publishers | NA | no | dometicated host | NA | no | no |
| 5393 | 2020 | 44113 | Mujibi, FD; Okoth, E; Cheruiyot, EK; Onzere, C; Bishop, RP; Fevre, EM; Thomas, L; Masembe, C; Plastow, G; Rothschild, M | 2018 | Genetic diversity, breed composition and admixture of Kenyan domestic pigs | The genetic diversity of African pigs, whether domestic or wild has not been widely studied and there is very limited published information available. Available data suggests that African domestic pigs originate from different domestication centers as opposed to international commercial breeds. We evaluated two domestic pig populations in Western Kenya, in order to characterize the genetic diversity, breed composition and admixture of the pigs in an area known to be endemic for African swine fever (ASF). One of the reasons for characterizing these specific populations is the fact that a proportion of indigenous pigs have tested ASF virus (ASFv) positive but do not present with clinical symptoms of disease indicating some form of tolerance to infection. Pigs were genotyped using either the porcine SNP60 or SNP80 chip. Village pigs were sourced from Busia and Homabay counties in Kenya. Because bush pigs (Potamochoerus larvatus) and warthogs (Phacochoerus spp.) are known to be tolerant to ASFv infection (exhibiting no clinical symptoms despite infection), they were included in the study to assess whether domestic pigs have similar genomic signatures. Additionally, samples representing European wild boar and international commercial breeds were included as references, given their potential contribution to the genetic make-up of the target domestic populations. The data indicate that village pigs in Busia are a non-homogenous admixed population with significant introgression of genes from international commercial breeds. Pigs from Homabay by contrast, represent a homogenous population with a local indigenous’ composition that is distinct from the international breeds, and clusters more closely with the European wild boar than African wild pigs. Interestingly, village pigs from Busia that tested negative by PCR for ASFv genotype IX, had significantly higher local ancestry (> 54%) compared to those testing positive, which contained more commercial breed gene introgression. This may have implication for breed selection and utilization in ASF endemic areas. A genome wide scan detected several regions under preferential selection with signatures for pigs from Busia and Homabay being very distinct. Additionally, there was no similarity in specific genes under selection between the wild pigs and domestic pigs despite having some broad areas under similar selection signatures. These results provide a basis to explore possible genetic determinants underlying tolerance to infection by ASFv genotypes and suggests multiple pathways for genetically mediated ASFv tolerance given the diversity of selection signatures observed among the populations studied. | NA | no | dometicated host | NA | no | no |
| 5394 | 2020 | 44112 | Muller, KE; Denison, RF | 2018 | Resource acquisition and allocation traits in symbiotic rhizobia with implications for life-history outside of legume hosts | Resources that microbial symbionts obtain from hosts may enhance fitness during free-living stages when resources are comparatively scarce. For rhizobia in legume root nodules, diverting resources from nitrogen fixation to polyhydroxybutyrate (PHB) has been discussed as a source of host-symbiont conflict. Yet, little is known about natural variation in PHB storage and its implications for rhizobial evolution. We therefore measured phenotypic variation in natural rhizObia populations and investigated how PHB might contribute to fitness in the free-living stage. We found that natural populations of rhizribia from Glycine max and Chamaecrista fasciculata had substantial, heritable variation in PHB acquisition during symbiosis. A model simulating temperature-dependent metabolic activity showed that the observed range of stored PHB per cell could support survival for a few days, for active cells, or over a century for sufficiently dormant cells. Experiments with field-isolated Bradyrhizobium in starvation culture suggest PHB is partitioned asymmetrically in dividing cells, consistent with individual-level bet-hedging previously demonstrated in E. meliloti. High-PHB isolates used more PHB over the first month, yet still retained more PHB for potential long-term survival in a dormant state. These results suggest that stored resources like PHB may support both short-term and long-term functions that contribute to fitness in the free-living stage. | NA | no | No good fitness measure and measure host GD | NA | no | no |
| 5395 | 2020 | 44111 | Muniz, AC; Lemos, JP; Buzatti, RSD; Ribeiro, PCC; Fernandes, FM; Lovato, MB | 2019 | Genetic data improve the assessment of the conservation status based only on herbarium records of a Neotropical tree | Although there is a consensus among conservation biologists about the importance of genetic information, the assessment of extinction risk and conservation decision-making generally do not explicitly consider this type of data. Genetic data can be even more important in species where little other information is available. In this study, we investigated a poorly known legume tree, Dimorphandra exaltata, from the Brazilian Atlantic Forest, a hotspot for conservation. We coupled species distribution models and geospatial assessment based on herbarium records with population genetic analyses to evaluate its genetic status and extinction risk, and to suggest conservation measures. Dimorphandra exaltata shows low genetic diversity, inbreeding, and genetic evidence of decrease in population size, indicating that the species is genetically depleted. Geospatial assessment classified the species as Endangered. Species distribution models projected a decrease in range size in the near future (2050). The genetic status of the species suggests low adaptive potential, which compromises its chances of survival in the face of ongoing climatic change. Altogether, our coupled analyses show that the species is even more threatened than indicated by geospatial analyses alone. Thus, conservation measures that take into account genetic data and the impacts of climate change in the species should be implemented. | NA | no | No good fitness measure and measure host GD | NA | no | no |
| 5396 | 2020 | 44111 | Munoz, M; Bozzi, R; Garcia, F; Nunez, Y; Geraci, C; Crovetti, A; Garcia-Casco, J; Alves, E; Skrlep, M; Charneca, R; Martins, JM; Quintanilla, R; Tibau, J; Kusec, G; Djurkin-Kusec, I; Mercat, MJ; Riquet, J; Estelle, J; Zimmer, C; Razmaite, V; Araujo, JP; Radovic, C; Savic, R; Karolyi, D; Gallo, M; Candek-Potokar, M; Fontanesi, L; Fernandez, AI; Ovilo, C | 2018 | Diversity across major and candidate genes in European local pig breeds | The aim of this work was to analyse the distribution of causal and candidate mutations associated to relevant productive traits in twenty local European pig breeds. Also, the potential of the SNP panel employed for elucidating the genetic structure and relationships among breeds was evaluated. Most relevant genes and mutations associated with pig morphological, productive, meat quality, reproductive and disease resistance traits were prioritized and analyzed in a maximum of 47 blood samples from each of the breeds (Alentejana, ApuloCalabrese, Basque, Bisara, Majorcan Black, Black Slavonian (Crna slavonska), Casertana, Cinta Senese, Gascon, Iberian, Kr6kopolje (Kr6kopoljski), Lithuanian indigenous wattle, Lithuanian White Old Type, Mora Romagnola, Moravka, Nero Siciliano, Sarda, Schwabisch-Hallisches Schwein (Swabian Hall pig), Swallow-Bellied Mangalitsa and Turopolje). We successfully analyzed allelic variation in 39 polymorphisms, located in 33 candidate genes. Results provide relevant information regarding genetic diversity and segregation of SNPs associated to production and quality traits. Coat color and morphological trait-genes that show low level of segregation, and fixed SNPs may be useful for traceability. On the other hand, we detected SNPs which may be useful for association studies as well as breeding programs. For instance, we observed predominance of alleles that might be unfavorable for disease resistance and boar taint in most breeds and segregation of many alleles involved in meat quality, fatness and growth traits. Overall, these findings provide a detailed catalogue of segregating candidate SNPs in 20 European local pig breeds that may be useful for traceability purposes, for association studies and for breeding schemes. Population genetic analyses based on these candidate genes are able to uncover some clues regarding the hidden genetic substructure of these populations, as the extreme genetic closeness between Iberian and Alentejana breeds and an uneven admixture of the breeds studied. The results are in agreement with available knowledge regarding breed history and management, although largest panels of neutral markers should be employed to get a deeper understanding of the population’s structure and relationships. | NA | no | dometicated host | NA | no | no |
| 5397 | 2020 | 44111 | Murgia, F; Melotti, R; Foco, L; Gogele, M; Meraviglia, V; Motta, B; Steger, A; Toifl, M; Sinnecker, D; Muller, A; Merati, G; Schmidt, G; Rossini, A; Pramstaller, PP; Pattaro, C | 2019 | Effects of smoking status, history and intensity on heart rate variability in the general population: The CHRIS study | Background Heart rate variability (HRV) reflects the autonomous nervous system modulation on heart rate and is associated with several pathologies, including cardiac mortality. While mechanistic studies show that smoking is associated with lower HRV, population-based studies present conflicting results. Methods We assessed the mutual effects of active smoking status, cumulative smoking history, and current smoking intensity, on HRV among 4751 adults from the Cooperative Health Research In South Tyrol (CHRIS) study. The HRV metrics standard deviation of normal-tonormal (NN) inter-beat intervals (SDNN), square root of the mean squared differences of consecutive NN intervals (RMSSD), total power (TP), low (LF) and high frequency (HF) power, and their ratio (LF/HF), were derived from 20-minute electrocardiograms. Smoking status, pack-years (PY), and tobacco grams/day from standardized questionnaires were the main exposures. We fitted linear mixed models to account for relatedness, non-linearity, and moderating effects, and including fractional polynomials. Results Past smokers had higher HRV levels than never smokers, independently of PY. The association of HRV with current smoking became apparent when accounting for the interaction between smoking status and PY. In current smokers, but not in past smokers, we observed HRV reductions between 2.0% (SDNN) and 4.9% (TP) every 5 PY increase. Furthermore, current smokers were characterized by dose-response reductions of 9.8% (SDNN), 8.9% (RMSSD), 20.1% (TP), 17.7% (LF), and 19.1% (HF), respectively, every 10 grams/day of smoked tobacco, independently of common cardiometabolic conditions and HRV-modifying drugs. The LF/HF ratio was not associated with smoking status, history, or intensity. Conclusions Smoking cessation was associated with higher HRV levels. In current smokers, heavier smoking intensity appears gradually detrimental on HRV, corroborating previous evidence. By affecting both the sympathetic and parasympathetic nervous system indexes, but not the LF/HF balance, smoking intensity seems to exert a systemic dysautonomic effect. | NA | no | human | NA | no | no |
| 5398 | 2020 | 44111 | Murphy, C; Burnett, S; Conroy, GC; Howland, BWA; Lamont, RW; Sumner, J; Ogbourne, SM | 2019 | Genetic diversity and structure of the threatened striped legless lizard, Delma impar: management implications for the species and a translocated population | The striped legless lizard, Delma impar, is a specialist grassland species restricted to south-eastern Australia. Anthropogenic influences have seen the destruction of much of its habitat and the species is threatened with extinction. Known populations of D. impar in Canberra (Australia) have recently been cleared for urban development. In 2015, Bush Heritage Australia translocated 41 individuals from these populations to Scottsdale Reserve. In this study, we completed the first population genetics analysis of D. impar in Canberra, providing a baseline for assessment of the genetic success of the translocation to Scottsdale Reserve. We analysed 154 D. impar individuals from six populations in Canberra, assessing levels of genetic diversity and differentiation within and between populations, using eight highly polymorphic microsatellite loci. High levels of genetic diversity and negligible levels of genetic differentiation were observed. Measures of allelic diversity were lower in the translocated population compared to the Canberra populations and Bayesian analysis revealed a disproportionate representation of two genetic clusters identified by STRUCTURE between the Scottsdale Reserve and Canberra populations, indicating that the initial genetic capture failed to capture’ recommended levels of genetic diversity to support an ongoing population. If the species successfully establishes itself at Scottsdale Reserve, the data suggests that the population should be augmented with individuals from other sites in Canberra, with the aim of increasing genetic diversity to recommended levels (i.e. >95% genetic variation). This will maximise resilience, adaptability and long-term survival potential of the Scottsdale Reserve population of striped legless lizards from a genetic context. | NA | no | no good gd measure host pop | NA | no | no |
| 5399 | 2020 | 44113 | Muturi, EJ; Ramirez, JL; Rooney, AP; Kim, CH | 2018 | Comparative Analysis of Gut Microbiota of Culex restuans (Diptera: Culicidae) Females From Different Parents | The potential for gut microbiota to impede or enhance pathogen transmission is well-documented but the factors that shape this microbiota in mosquito vectors are poorly understood. We characterized and compared the gut microbiota of adult females of Culex restuans (Theobald; Diptera: Culicidae) from different parents. Cx. restuans larvae from nine field-collected egg rafts were reared on a common diet and gut microbiota of newly emerged adult females characterized by MiSeq sequencing of the V4 hypervariable region of the 16S rRNA gene. Bacterial diversity and evenness in individuals from one egg raft were significantly lower compared to those of three of the other eight egg rafts. The gut microbiota of adult females reared from seven of the nine egg rafts clustered together suggesting that individuals from most egg rafts had similar profiles of gut microbiota. These findings suggest that the microbiota of adult females from the same parents do not differ appreciably from the microbiota of adult females from different parents. However, additional studies using mosquitoes separated by geographic distances greater than those studied here and estimating the genetic distances between populations from different egg rafts are needed to provide further insights into the influence of host genetics on gut microbiota. Also worthwhile are studies evaluating how individuals from different egg rafts and harboring different gut microbiota compare in relation to vector competence for different pathogens. | NA | no | No good fitness measure and measure host GD | NA | no | no |
| 5400 | 2020 | 44041 | Mysterud, A; Madslien, K; Viljugrein, H; Vikoren, T; Andersen, R; Guere, ME; Benestad, SL; Hopp, P; Strand, O; Ytrehus, B; Roed, KH; Rolandsen, CM; Vage, J | 2019 | The demographic pattern of infection with chronic wasting disease in reindeer at an early epidemic stage | Infection patterns linked to age and sex are crucial to predict the population dynamic effects of diseases in long-lived species. How such demographic patterns of infection arise is often multifactorial, although the cause is commonly seen as a combination of immune status as well as variation in pathogen exposure. Prion diseases are particularly interesting, as they do not trigger an adaptive immune response; hence, differences in pathogen exposure linked to behavior could be the prime determinant of the pattern of infection. In cervids, the fatal prion disease, chronic wasting disease (CWD), is spreading geographically, with economic and cultural consequences in affected areas in North America, and all infected individuals eventually die from disease-associated sequelae if they live long enough. Understanding the causes of the demographic pattern of infection with CWD is therefore urgent but is limited by the fact that reported data primarily come from related deer species in North America. The recent (detected 2016) emergence of CWD among wild alpine reindeer (Rangifer tarandus) in Norway with a different social organization, that is, no home range behavior and no matrilineal female groups, offers an opportunity to advance our understanding of how behavior influences the infection patterns. Testing of 1081 males and 1278 females detected 19 animals positive for abnormal prion protein in brain and/or lymphatic tissues. No calves and only one male yearling were infected, with the remaining positives being adults (representing 1.5% of adult males and 0.5% of adult females). We found a strong sex-biased infection pattern in reindeer (with infection 2.7 times more likely in adult males), which is similar to the results reported in mule deer and white-tailed deer. The hazard of being detected as positive increased with age in males. There was no close genetic relatedness among positive animals. The results were consistent with the within-group contact of males being a possible major route of transmission. We discuss the demographic pattern of infection with CWD in view of the lack of stable home range behavior and other key behavioral traits of reindeer relevant to understanding pathogen exposure in general. | NA | no | No good fitness measure and measure host GD | NA | no | no |
| 5401 | 2020 | 44111 | Nakato, GV; Rojas, JLF; Verniere, C; Blondin, L; Coutinho, T; Mahuku, G; Wicker, E | 2019 | A new Multi Locus Variable Number of Tandem Repeat Analysis Scheme for epidemiological surveillance of Xanthomonas vasicola pv. musacearum, the plant pathogen causing bacterial wilt on banana and enset | Xanthomonas vasicola pv. musacearum (Xvm) which causes Xanthomonas wilt (XW) on banana (Musa accuminata x balbisiana) and enset (Ensete ventricosum), is closely related to the species Xanthomonas vasicola that contains the pathovars vasculorum (Xvv) and holcicola (Xvh), respectively pathogenic to sugarcane and sorghum. Xvm is considered a monomorphic bacterium whose intra-pathovar diversity remains poorly understood. With the sudden emergence of Xvm within east and central Africa coupled with the unknown origin of one of the two sublineages suggested for Xvm, attention has shifted to adapting technologies that focus on identifying the origin and distribution of the genetic diversity within this pathogen. Although microbiological and conventional molecular diagnostics have been useful in pathogen identification. Recent advances have ushered in an era of genomic epidemiology that aids in characterizing monomorphic pathogens. To unravel the origin and pathways of the recent emergence of XW in Eastern and Central Africa, there was a need for a genotyping tool adapted for molecular epidemiology. Multi-Locus Variable Number of Tandem Repeat Analysis (MLVA) is able to resolve the evolutionary patterns and invasion routes of a pathogen. In this study, we identified microsatellite loci from nine published Xvm genome sequences. Of the 36 detected microsatellite loci, 21 were selected for primer design and 19 determined to be highly typeable, specific, reproducible and polymorphic with two-to four-alleles per locus on a sub-collection. The 19 markers were multiplexed and applied to genotype 335 Xvm strains isolated from seven countries over several years. The microsatellite markers grouped the Xvm collection into three clusters; with two similar to the SNP-based sublineages 1 and 2 and a new cluster 3, revealing an unknown diversity in Ethiopia. Five of the 19 markers had alleles present in both Xvm and Xanthomonas vasicola pathovars holcicola and vasculorum, supporting the phylogenetic closeliness of these three pathovars. Thank to the public availability of the haplotypes on the MLVABank database, this highly reliable and polymorphic genotyping tool can be further used in a transnational surveillance network to monitor the spread and evolution of XW throughout Africa.. It will inform and guide management of Xvm both in banana-based and enset-based cropping systems. Due to the suitability of MLVA-19 markers for population genetic analyses, this genotyping tool will also be used in future microevolution studies. | NA | no | gd of pathogen | NA | no | no |
| 5402 | 2020 | 44040 | Naude, VN; Balme, GA; O’Riain, J; Hunter, LTB; Fattebert, J; Dickerson, T; Bishop, JM | 2020 | Unsustainable anthropogenic mortality disrupts natal dispersal and promotes inbreeding in leopards | Anthropogenic mortality of wildlife is typically inferred from measures of the absolute decline in population numbers. However, increasing evidence suggests that indirect demographic effects including changes to the age, sex, and social structure of populations, as well as the behavior of survivors, can profoundly impact population health and viability. Specifically, anthropogenic mortality of wildlife (especially when unsustainable) and fragmentation of the spatial distribution of individuals (home-ranges) could disrupt natal dispersal mechanisms, with long-term consequences to genetic structure, by compromising outbreeding behavior and gene flow. We investigate this threat in African leopards (Panthera pardus pardus), a polygynous felid with male-biased natal dispersal. Using a combination of spatial (home-range) and genetic (21 polymorphic microsatellites) data from 142 adult leopards, we contrast the structure of two South African populations with markedly different histories of anthropogenically linked mortality. Home-range overlap, parentage assignment, and spatio-genetic autocorrelation together show that historical exploitation of leopards in a recovering protected area has disrupted and reduced subadult male dispersal, thereby facilitating opportunistic male natal philopatry, with sons establishing territories closer to their mothers and sisters. The resultant kin-clustering in males of this historically exploited population is comparable to that of females in a well-protected reserve and has ultimately led to localized inbreeding. Our findings demonstrate novel evidence directly linking unsustainable anthropogenic mortality to inbreeding through disrupted dispersal in a large, solitary felid and expose the genetic consequences underlying this behavioral change. We therefore emphasize the importance of managing and mitigating the effects of unsustainable exploitation on local populations and increasing habitat fragmentation between contiguous protected areas by promoting in situ recovery and providing corridors of suitable habitat that maintain genetic connectivity. | NA | no | inbreeding | NA | no | no |
| 5403 | 2020 | 44041 | Nazarian, A; Arbeev, KG; Kulminski, AM | 2020 | The impact of disregarding family structure on genome-wide association analysis of complex diseases in cohorts with simple pedigrees | The generalized linear mixed models (GLMMs) methodology is the standard framework for genome-wide association studies (GWAS) of complex diseases in family-based cohorts. Fitting GLMMs in very large cohorts, however, can be computationally demanding. Also, the modified versions of GLMM using faster algorithms may underperform, for instance when a single nucleotide polymorphism (SNP) is correlated with fixed-effects covariates. We investigated the extent to which disregarding family structure may compromise GWAS in cohorts with simple pedigrees by contrasting logistic regression models (i.e., with no family structure) to three LMMs-based ones. Our analyses showed that the logistic regression models in general resulted in smaller P values compared with the LMMs-based models; however, the differences in P values were mostly minor. Disregarding family structure had little impact on determining disease-associated SNPs at genome-wide level of significance (i.e., P < 5E-08) as the four P values resulted from the tested methods for any SNP were all below or all above 5E-08. Nevertheless, larger discrepancies were detected between logistic regression and LMMs-based models at suggestive level of significance (i.e., of 5E-08 <= P < 5E-06). The SNP effects estimated by the logistic regression models were not statistically different from those estimated by GLMMs that implemented Wald’s test. However, several SNP effects were significantly different from their counterparts in LMMs analyses. We suggest that fitting GLMMs with Wald’s test on a pre-selected subset of SNPs obtained from logistic regression models can ensure the balance between the speed of analyses and the accuracy of parameters. | NA | no | No good fitness measure and measure host GD | NA | no | no |
| 5404 | 2020 | 44113 | Nazir, F; Ibrahim, M; Zaman, G; Hussain, A; Yar, AM; Bo, Z | 2018 | Genetic Diversity and Functional Analysis of Sigma Factors in Enterobacter cloacae Complex Resourced From Various Niche | Sigma factors are bacterial transcription factors that bind the core RNA polymerase and direct transcription initiation at a specific promoter site. These specialized sigma factors bind the promoters of genes appropriate to the environmental conditions and selectively increase the transcription of those genes. Here, we attempt to identify sigma factors in 5 genomes belonging to the Enterobacter cloacae complex (Ecc), a group of gram-negative bacteria that are important nosocomial pathogens. This process includes the identification of orthologous sequences, conserved motifs, domains, families, phylogenetic profiles, and protein-protein associations of these components. Based on the reference genome, genome-wide comparison revealed that the genomes of Enterobacter asburiae JCM6051, Enterobacter nimipressuralis CIP 104980, Enterobacter hormaechei ATCC49162, Enterobacter kobei JCM 8580, and Enterobacter ludwigii EN-119 encode 10 sigma factors that exist in the reference strain Enterobacter cloacae subsp cloacae ATCC13047. Moreover, the sequence similarity, protein domains and families of the sigma factors, protein-protein association, and phylogenetic profile indicate that the sigma factor proteins of these 5 strains may have evolutionary relatedness and functional characteristics important to their various environmental niches. Interestingly, the absence of RpoS in E kobei, which contributes to bacterial survival under environmental stress conditions, indicates that RpoS might have been independently acquired and may play different roles relating to pathogenicity, host range determination, and/or niche adaptation. Future work such as RNA sequencing will be directed towards investigating the roles that these sigma factors play in the biology of the Ecc. | NA | no | No good fitness measure and measure host GD | NA | no | no |
| 5405 | 2020 | 44112 | Nehring, V; Dijkstra, MB; Sumner, S; Hughes, WOH; Boomsma, JJ | 2018 | Reconstructing the relatedness of cooperatively breeding queens in the Panamanian leaf-cutting ant Acromyrmex echinatior (Hymenoptera: Formicidae) | The evolution of permanent inquiline social parasites in ants has been conjectured to be facilitated by secondary polygyny, that is, the re-adoption of new queens into existing mature colonies. This idea was first formulated by Wasmann, Wheeler, and Emery more than a century ago. Emery predicted that inquilines should be the sister-lineages of their hosts, which prompted Alfred Buschinger to propose that they evolve by sympatric speciation. However, these scenarios hinge on two vital conditions that have not been quantitatively documented: 1. That host sister species are secondarily polygynous and primarily recruit close kin, and 2. That such adoptions are prone to occasional mistakes that would select for the condition-dependent expression of exploitative traits and reproductive isolation by disruptive selection. Here, we use a long-term data set on the leaf-cutting ant Acromyrmex echinatior (FOREL, 1899), known to have a closely related inquiline social parasite A. insinuator SCHULTZ, BEKKEVOLD & BOOMSMA, 1998, to address the first of these conditions. We estimate the frequency of secondary polygyny and the degree to which cooperatively breeding queens are related. We find that the overall frequency of polygynous colonies is ca. 8% and that polygynous colonies typically have two queens. Most queen pairs are first-degree relatives, consistent with colonies adopting one or two daughters either before or just after becoming orphaned. However, we also document a few pairs of cooperatively breeding queens that are unrelated and estimate that this social structure may apply to ca. 20% of the polygynous colonies, and thus ca. 1% of all colonies. Our findings show that the breeding system of A. echinatior matches the polygyny characteristics that are believed to facilitate the emergence of socially parasitic queen morphs. | NA | no | No good fitness measure | NA | no | no |
| 5406 | 2020 | 44112 | Nelson-Flower, MJ; Germain, RR; MacDougall-Shackleton, EA; Taylor, SS; Arcese, P | 2018 | Purifying Selection in the Toll-Like Receptors of Song Sparrows Melospiza melodia | Variation in immune gene sequences is known to influence resistance to infectious diseases and parasites, and hence survival and mate choice, across animal taxa. Toll-like receptors (TLRs) comprise one essential gene family in the vertebrate innate immune system and recognize evolutionarily conserved structures from all major microorganism classes. However, the causes and consequences of TLR variation in passerine birds remain largely unexplored. We examined 7 TLR genes in song sparrows (Melospiza melodia), a species that is studied across North America. We then examined sequences from 4 unduplicated TLRs (TLR1LB, TLR3, TLR4, and TLR15) from birds in 2 parts of the species’ range (N = 27, N = 6), tested for evidence of selection, and conducted pilot analyses of the role of TLR heterozygosity in survival. We identified 45 SNPs: 19 caused changes in amino acid sequences and 2 of these were likely deleterious. We found no evidence of codonlevel episodic positive selection but detected purifying selection at codons in all TLRs. Contrary to expectations we found no strong correlation between heterozygosity at TLRs and inbreeding coefficient f (estimate +/- standard error [SE] = -0.68 +/- 0.37, R-adj(2) = 0.08, F-1,F-25 = 3.38, P = 0.08). In addition, pilot analyses revealed no relationship between TLR heterozygosity and survival (ss +/- SE: 0.09 +/- 2.00, P = 0.96), possibly due to small sample size. Further analyses of genetic diversity in TLRs are likely to advance understanding of the effects of innate immune gene diversity on the fitness and persistence of wild populations. | NA | no | No good fitness measure and measure host GD | NA | no | no |
| 5407 | 2020 | 44113 | Nemeshazi, E; Szabo, K; Horvath, Z; Kover, S | 2018 | GENETIC STRUCTURE CONFIRMS FEMALE-BIASED NATAL DISPERSAL IN THE WHITE-TAILED EAGLE POPULATION OF THE CARPATHIAN BASIN | Individuals can avoid inbreeding or competition with kin via long-distance natal dispersal. On the other hand, staying close to the well-known natal area may be a safer choice with respect to recruiting opportunities, reproductive success and the individual’s survival probability as well. Natal dispersal strategy often differs between sexes, being generally female-biased in birds. We explored if the Carpathian Basin White-tailed Eagle population shows fine scale genetic structure and if it does, what is the extent of philopatry in the two sexes. We furthermore investigated sex bias in natal dispersal distance inferred from spatial distributions of genetically close relative breeding females and males. Spatial autocorrelation analyses failed to find fine-scale genetic structure, despite the species being known to be philopatric. Pairwise breeding distances of close relatives showed female bias according to Wilcoxon rank sum test. The median distance of two close relative females was 136 km, while it was only 38 km in males. Since White-tailed Eagles are known to be faithful to their breeding territory, we assumed that the breeding distance between an individual and its parents refers to the individual’s natal dispersal distance. Due to the same reason, the breeding distance of two siblings should also be related to their individual dispersal distances from their shared natal area. Therefore, we argue that the difference we found between sexes in pairwise breeding distances of close relatives stands for a female-biased natal dispersal. This bias may be a consequence of the species’ breeding strategy, and it decreases the inbreeding probability as well. | NA | no | No good fitness measure | NA | no | no |
| 5408 | 2020 | 44042 | Ngernmuen, A; Suktrakul, W; Damchuay, K; Longya, A; Kate-Ngam, S; Jantasuriyarat, C | 2019 | Substantial enhancement of high polymorphic SSR marker development using in silico method from 18 available rice blast fungus genome sequences and its application in genetic diversity assessment | Rice blast fungus, Magnaporthe oryzae, causes rice blast disease leading to the reduction of rice yield. Utilization of rice blast resistant cultivar is one of efficient ways to cope with this disease. The genetic diversity of rice blast is of great importance as it indicates the genotype of rice blast isolates in a population of epidemic area and reveals the dynamics of genotype change over time. Simple sequence repeat (SSR) marker is frequently used for indicating the genetic diversity of several organisms including rice blast fungus. The in silico SSR development method provides the enhancement of polymorphic rate and requires less time and labour. This study aimed to develop in silico SSR marker and study genetic diversity of rice blast isolates collected from Thailand. The 11,072 SSR loci were examined in rice blast reference genome 70-15. The most abundant SSR type and pattern were mono-nucleotide repeat and AGC/CTG motif respectively. Fourteen highly polymorphic SSR loci, identified by in silico from 18 rice blast genome sequences, were selected and specific primer were developed. Ten SSR loci were novel and detected the polymorphism in Thai rice blast isolates, which showed 2-7 alleles per locus and PIC values ranged from 0.07 to 0.56. The genetic relationship analysis showed that rice blast isolates were clustered by collection time and site. Our result indicated that in silico SSR development is a powerful technique to enhance the production of highly polymorphic SSR markers and these newly developed SSR markers were useful for genetic diversity assessment. | NA | no | No good fitness measure and measure host GD | NA | no | no |
| 5409 | 2020 | 44042 | Ni, WW; E, GX; Basang, WD; Zhu, YB; Huang, YF | 2019 | Molecular Variant Estimation of SLC11A1 Related Microsatellites in Chinese Indigenous Goats | The aim of this study was to investigate the polymorphism of the SLC11A1 gene in Chinese indigenous goats. A total of 215 individuals from nine goat breeds were genotyped using two pairs of known SLC11A1. Thirty-four alleles were found in these breeds across two microsatellite loci. Observed heterozygosity ranged from 0.2292 +/- 0.0607 in CDB to 0.3958 +/- 0.0706 in CDM. The mean number of alleles ranged from 4.50 +/- 2.12 in CDB to 7.50 +/- 2.12 in CDM. The coefficient of inbreeding (F-IS) was lowest in CDB (0.267) and highest in DZ (0.459). All populations deviated from HWE in these two markers, except for JZ and CDM populations, which had one (EX-15). According to the F-ST results, only nine pairs were significantly different. Bayesian analysis using the program STRUCTURE, as well as Neighbor-joining tree analysis, both expressed 2 clusters, revealing genetic clustering patterns consistent with geographic location. In summary, the results of this study indicated that nine breeds in the Chuan-Yu area were divided into two groups according to geographical distribution. Our study may provide guidance for future research investigating marker-assisted selection and disease-resistant breeding using the SLC11A1 gene. | NA | no | No good fitness measure and measure host GD | NA | no | no |
| 5410 | 2020 | 44042 | Nice, L; Huang, YD; Steffenson, BJ; Gyenis, L; Schwarz, P; Smith, KP; Muehlbauer, GJ | 2019 | Mapping malting quality and yield characteristics in a north American two-rowed malting barley x wild barley advanced backcross population | Due to the stringent quality requirements imposed by the US malting barley industry, barley breeders have been reluctant to introduce exotic germplasm into cultivar development programs. To determine whether wild barley (Hordeum vulgare ssp. spontaneum) contains favorable alleles for yield and malting quality characteristics, we mapped quantitative trait loci (QTL) for heading date, height, lodging, yield, and nine malting parameters important to the malting and brewing industry. Traits were mapped in a wild x cultivated barley BC2-derived advanced backcross mapping population. Harrington, the recurrent parent, is a North American two-rowed malting barley cultivar, and OUH602, the donor parent, is a wild barley accession that exhibits resistance to multiple barley diseases. The 98 derived lines were grown in replicated field trials at St. Paul and Crookston, MN in 2009-2011. One to four QTL were identified for each trait, for a total of 36 QTL. Trangressive segregants for increased yield were identified and four lines had higher yield than Harrington across all environments; however, for yield QTL the OUH602 alleles decreased the trait value. Wild barley alleles had both positive and negative effects on the malting traits of diastatic power, free amino nitrogen, and soluble protein. Combined with the previously identified QTL associated with resistance to fungal diseases, this population represents a rich resource for barley breeding. To facilitate future breeding and genetics studies with this population, a set of pre-introgression lines composed of 28 BC2-derived and 6 BC3-derived lines were identified that collectively contain introgressions across the entire OUH602 genome. | NA | no | No good fitness measure and measure host GD | NA | no | no |
| 5411 | 2020 | 44042 | Niederle, MV; Bosch, J; Ale, CE; Nader-Macias, ME; Ficoseco, CA; Toledo, LF; Valenzuela-Sanchez, A; Soto-Azat, C; Pasteris, SE | 2019 | Skin-associated lactic acid bacteria from North American bullfrogs as potential control agents of Batrachochytrium dendrobatidis | The fungal pathogen Batrachochytrium dendrobatidis (Bd) is the causative agent of chytridiomycosis and has been a key driver in the catastrophic decline of amphibians globally. While many strategies have been proposed to mitigate Bd outbreaks, few have been successful. In recent years, the use of probiotic formulations that protect an amphibian host by killing or inhibiting Bd have shown promise as an effective chytridiomycosis control strategy. The North American bullfrog (Lithobates catesbeianus) is a common carrier of Bd and harbours a diverse skin microbiota that includes lactic acid bacteria (LAB), a microbial group containing species classified as safe and conferring host benefits. We investigated beneficial/probiotic properties: anti-Bd activity, and adhesion and colonisation characteristics (hydrophobicity, biofilm formation and exopolysaccharide-EPS production) in two confirmed LAB (cLAB-Enterococcus gallinarum CRL 1826, Lactococcus garvieae CRL 1828) and 60 presumptive LAB (pLAB) [together named as LABs] isolated from bullfrog skin. We challenged LABs against eight genetically diverse Bd isolates and found that 32% of the LABs inhibited at least one Bd isolate with varying rates of inhibition. Thus, we established a score of sensitivity from highest (BdGPL AVS7) to lowest (BdGPL C2A) for the studied Bd isolates. We further reveal key factors underlying host adhesion and colonisation of LABs. Specifically, 90.3% of LABs exhibited hydrophilic properties that may promote adhesion to the cutaneous mucus, with the remaining isolates (9.7%) being hydrophobic in nature with a surface polarity compatible with colonisation of acidic, basic or both substrate types. We also found that 59.7% of LABs showed EPS synthesis and 66.1% produced biofilm at different levels: 21% weak, 29% moderate, and 16.1% strong. Together all these properties enhance colonisation of the host surface (mucus or epithelial cells) and may confer protective benefits against Bd through competitive exclusion. Correspondence analysis indicated that biofilm synthesis was LABs specific with high aggregating bacteria correlating with strong biofilm producers, and EPS producers being correlated to negative biofilm producing LABs. We performed Random Amplified Polymorphic DNA (RAPD)-PCR analysis and demonstrated a higher degree of genetic diversity among rod-shaped pLAB than cocci. Based on the LAB genetic analysis and specific probiotic selection criteria that involve beneficial properties, we sequenced 16 pLAB which were identified as Pediococcus pentosaceus, Enterococcus thailandicus, Lactobacillus pentosus/L. plantarum, L. brevis, and L. curvatus. Compatibility assays performed with cLAB and the 16 species described above indicate that all tested LAB can be included in a mixed probiotic formula. Based on our analyses, we suggest that E. gallinarum CRL 1826, L. garvieae CRL 1828, and P. pentosaceus 15 and 18B represent optimal probiotic candidates for Bd control and mitigation. | NA | no | No good fitness measure and measure host GD | NA | no | no |
| 5412 | 2020 | 44113 | Noer, Z; Hasanuddin; Lisnawita; Suryanto, D | 2018 | Molecular identification xanthomonas oryzae pv. Oryzae causes of bacterial blight for rice with a specific primers | The Xanthomonas genus is comprised of more than 20 species and many cause important diseases in various plants. The bacterial blight (BB) is a disease caused by Xanthomonas oryzae pv. oryzae (Xoo). This pathogen infects the leaves in all plant growth phases. This study aims to identify bacterial Xoo isolates from North Sumatra province using Xoo2976 specific primers. The bacterial isolates were grown on Wakimoto Agar (WA) medium in petri dishes after which the bacteria suspected of being Xoo were identified molecularly. DNA of bacteria indicating Xoo was extracted and then amplified using the PCR method with Xoo2976F specific primers. The amplification results show positive band continued with sequencing. The sequencing results were analyzed homologically using the BLASTN (Basic Local Alignment Sequence Tool Nucleotide) program based on data from the NCBI (National Center for Biotechnology Information). The results show that the specific primers used were able to produce DNA bands 337 bp-long. The result of BLASTN analysis showed that the DNA sequence was from homology amplification with Xoo bacteria. GC content of sequencing results reached 62.59%, whereas AT content reached 37.41%. Phylogenetic tree analysis by Neighbor Joining method classifies isolate samples into the same group with Xoo isolates and apart from X. citri, X. fuscans, X. axonopodis, X. campestris, and X. perforans isolates. Specific markers used in the study can be used to identify Xoo bacteria. The results of the study can be used as a method to identify Xoo bacteria for various needs related to plant diseases caused by the said bacteria | NA | no | gd of pathogen | NA | no | no |
| 5413 | 2020 | 44112 | Norris, ET; Wang, L; Conley, AB; Rishishwar, L; Marino-Ramirez, L; Valderrama-Aguirre, A; Jordan, IK | 2018 | Genetic ancestry, admixture and health determinants in Latin America | BackgroundModern Latin American populations were formed via genetic admixture among ancestral source populations from Africa, the Americas and Europe. We are interested in studying how combinations of genetic ancestry in admixed Latin American populations may impact genomic determinants of health and disease. For this study, we characterized the impact of ancestry and admixture on genetic variants that underlie health- and disease-related phenotypes in population genomic samples from Colombia, Mexico, Peru, and Puerto Rico.ResultsWe analyzed a total of 347 admixed Latin American genomes along with 1102 putative ancestral source genomes from Africans, Europeans, and Native Americans. We characterized the genetic ancestry, relatedness, and admixture patterns for each of the admixed Latin American genomes, finding a spectrum of ancestry proportions within and between populations. We then identified single nucleotide polymorphisms (SNPs) with anomalous ancestry-enrichment patterns, i.e. SNPs that exist in any given Latin American population at a higher frequency than expected based on the population’s genetic ancestry profile. For this set of ancestry-enriched SNPs, we inspected their phenotypic impact on disease, metabolism, and the immune system. All four of the Latin American populations show ancestry-enrichment for a number of shared pathways, yielding evidence of similar selection pressures on these populations during their evolution. For example, all four populations show ancestry-enriched SNPs in multiple genes from immune system pathways, such as the cytokine receptor interaction, T cell receptor signaling, and antigen presentation pathways. We also found SNPs with excess African or European ancestry that are associated with ancestry-specific gene expression patterns and play crucial roles in the immune system and infectious disease responses. Genes from both the innate and adaptive immune system were found to be regulated by ancestry-enriched SNPs with population-specific regulatory effects.ConclusionsAncestry-enriched SNPs in Latin American populations have a substantial effect on health- and disease-related phenotypes. The concordant impact observed for same phenotypes across populations points to a process of adaptive introgression, whereby ancestry-enriched SNPs with specific functional utility appear to have been retained in modern populations by virtue of their effects on health and fitness. | NA | no | human | NA | no | no |
| 5414 | 2020 | 44112 | Nourani, L; Aliabadian, M; Mirshamsi, O; Djadid, ND | 2018 | Molecular detection and genetic diversity of avian haemosporidian parasites in Iran | Background The mobility of birds across or between continents exposes them to numerous vectors that have the potential to transmit pathogens and spread them into new regions. A combination of rich species diversity of birds along with the small amount of molecular studies in Iran makes observing the blood parasite distribution in wild avian populations indispensable for further estimation and administration of blood parasites. Methodology/Principal findings In order to evaluate the infection rate and molecular context of avian blood parasites, bird samples were collected (passerine = 316 and non-passerine = 14) in eight provinces of northern Iran between June to September 2015 and 2016. All bird samples were examined for haematoprotozoan infections by morphological screening using light microscope and mtDNA cytb gene amplification. A total of 115 birds were positive for blood parasites by molecular approach (34.84% overall infection). The infection rate of Haemoproteus, Plasmodium, and Leucocytozoon were 33.03%, 1.21%, and 0.6%, respectively. Sequences analysis has detected 43 lineages in Iranian birds’ hosts. Lineages were attributed to three genera Haemoproteus (n = 37), Plasmodium (n = 4), and Leucocytozoon (n = 2), of which 23 lineages fully matched previously recorded sequences in GenBank and MalAvi data reciprocities. Five lineages of ACDUM1, ACDUM2, PARUS1, PYERY01, and SISKIN1 were detected in multiple hosts’ species from dissimilar families. In Bayesian tree, all sequences were clustered in three main monophyletic clades as Haemoproteus, Plasmodium, and Leucocytozoon genera. Conclusions/Significance As the first study outlining the molecular detection of hematozoa of passerines from Iran, the current study has recorded 20 new lineages for three genera of Haemoproteus, Plasmodium, and Leucocytozoon. Additional investigations into these taxa in the avifauna for the other parts of Iran may provide extra information on blood parasites, hosts relationships and distribution patterns. | NA | no | gd of pathogen | NA | no | no |
| 5415 | 2020 | 44112 | Novillo, A; Gaibar, M; Romero-Lorca, A; Chaabani, H; Amir, N; Moral, P; Esteban, ME; Fernandez-Santander, A | 2018 | UDP-glucuronosyltransferase genetic variation in North African populations: a comparison with African and European data | Background: Genetic variation in glucuronosyltransferases (UGT) is crucial in drug metabolism and risk of some diseases. Aim: To examine genetic variation in UGT in North African populations. Subjects and methods: Allele frequencies of SNPs UGT1A4(24Thr), UGT1A4(48Val), UGT2B15(85Tyr), UGT2B15(523Thr) and UGT2B17 CNV deletion from Morocco, Algeria, Tunisia and Libya were compared to European and Sub-Saharan populations. Results: North Africans are the group with the highest genetic heterogeneity given by internal differences in the occurrence of UGT2B17 deletion, UGT1A4(48Val) and UGT1A4 haplotypes. UGT2B15 SNPs differentiate Sub-Saharans from the rest of the populations. Conclusion: North African populations show a high frequency of carriers of UGT2B15(523Thr), a variant linked to an increased risk of prostate cancer. High Atlas Moroccans and Algerians show low frequency of UGT2B17(del), a variant associated with high concentrations of testosterone and oestradiol. | NA | no | human | NA | no | no |
| 5416 | 2020 | 44111 | Nowicki, P; Deoniziak, K; Dziekanska, I; Kostro-Ambroziak, A; Plazio, E; Rutkowski, R; Sielezniew, M | 2019 | What keeps living dead’ alive: demography of a small and isolated population of Maculinea (=Phengaris) alcon | Small and isolated populations are prone to future extinctions and thus perceived as living dead’. Although generally considered to be of low conservation value, their existence can still enhance species survival at the landscape scale through improving the connectivity of other populations and facilitating some (even if little) gene flow. We investigated the demography and genetic status of a tiny and highly isolated local population of Maculinea (=Phengaris) alcon near its distribution margin with the aim of identifying the features that allow it to persist. The study comprised intensive mark-recapture, surveys of Gentiana pneumonanthe foodplants and butterfly eggs laid on them, as well as genetic analyses. The population has been found to be characterised by low genetic diversity and estimated at only a few tens of individuals. The foodplant availability turned out to be the most obvious factor limiting M. alcon abundance. Nevertheless, the life expectancy of adult butterflies is fairly long, and their flight period very short, implying that most individuals occur within the same time window. Together with the relatively little protandry observed, i.e. almost synchronous emergence of males and females, this increases the chances of random mating among the individuals. Moreover, the butterflies move freely across the core habitat fragment. All things concerned, the effective population size is presumably not much lower than the recorded population size. Our findings provide guidelines for pinpointing those among living dead’ populations that are likely to be the most persistent and thus worth conservation efforts aimed at preserving them. | NA | no | No good fitness measure and measure host GD | NA | no | no |
| 5417 | 2020 | 44111 | Nsibo, DL; Barnes, I; Kunene, NT; Berger, DK | 2019 | Influence of farming practices on the population genetics of the maize pathogen Cercospora zeina in South Africa | Gray leaf spot (GLS) is an important foliar disease of maize. This disease, caused by Cercospora zeina, is prevalent in both smallholder and commercial maize farms in South Africa. Notably, smallholder practices are geared towards conservation agriculture, planting diverse maize genotypes within a field and avoiding chemical control. This study examined the population genetic structure of 129 C. zeina isolates from three smallholder farm sites in KwaZulu-Natal in South Africa using 13 microsatellite markers. These were analysed, together with 239 isolates previously analysed from four commercial farms in the same province, to determine whether fanning systems influence the genetic diversity of C. zeina. In addition, we wanted to determine whether the smallholder fanning system harboured a greater diversity of C. zeina haplotypes due to lack of chemical spraying of these crops. Overall, farming systems exhibited partial, but significant, population differentiation, contributing 10% of the genetic variation observed. A 16% genetic variation conferred between KwaNxamalala (smallholder) and Cedara (commercial) areas that are in close proximity, confirmed this. Private alleles accounted for 29% of the 52 alleles observed in smallholder farms. Smallholder farms harboured a higher gene and genotypic diversity, with a clonal fraction of only 13% compared to 32% in commercial farms. Mating type ratios indicative of sexual recombination and lower linkage disequilibrium in most smallholder populations were consistent with higher levels of diversity. This study suggests that commercial fanning practices, such as fungicides and monoculture crop planting, may result in a narrower genetic diversity of the pathogen that is then propagated by asexual reproduction. In contrast, management of GLS disease in smallholder farms should consider the greater diversity of pathogen genotypes, especially if future research shows that this equates to a greater diversity of pathogenicity alleles. | NA | no | No good fitness measure and measure host GD | NA | no | no |
| 5418 | 2020 | 44042 | Nyouma, A; Bell, JM; Jacob, F; Cros, D | 2019 | From mass selection to genomic selection: one century of breeding for quantitative yield components of oil palm (Elaeis guineensis Jacq.) | More efficient methods are required to breed oil palm (Elaeis guineensis Jacq.) for yield maximization in order to meet the increased demand for palm oil while limiting environmental impacts. This review article analyzes the evolution of breeding schemes for oil palm yield and its quantative components and the changes expected to take place with genomic selection (GS). Genetic improvement of oil palm yield started in the 1920s through mass selection. Later, several disruptive improvements dramatically increased the rate of genetic progress: (1) understanding the heredity of fruit form and the adoption of tenera, with thicker mesocarp, in plantations; (2) the discovery of hybrid vigor and the adoption of modified reciprocal recurrent selection; and (3) clonal selection, exploiting intra-hybrid variability. In addition, the use of linear mixed models to estimate genetic values has made selection more efficient. Today, GS appears to be a new disruptive improvement that can speed up breeding schemes by avoiding field trials in some cycles and increase selection intensity by evaluating more candidates. The genetic potential for oil palm yield has increased considerably over one century of breeding. GS is expected to bring the rate of genetic progress to a previously unprecedented level. The future studies on oil palm GS will aim at making it efficient for all yield components. For this purpose, they should focus in particular on the optimization of training populations and on the improvement of prediction models. Minimizing environmental impacts will also require improvement in other aspects (resistance to diseases, cultural practices, etc.). | NA | no | dometicated host | NA | no | no |
| 5419 | 2020 | 44039 | O’Reilly, GD; Jabot, F; Gunn, MR; Sherwin, WB | 2020 | Predicting Shannon’s information for genes in finite populations: new uses for old equations | This study provides predictive equations for Shannon’s information in a finite population, which are intuitive and simple enough to see wide scale use in molecular ecology and population genetics. A comprehensive profile of genetic diversity contains three complementary components: numbers of allelic types, Shannon’s information and heterozygosity. Currently heterozygosity has greater resources than Shannon’s information, such as more predictive models and integration into more mainstream genetics software. However, Shannon’s information has several advantages over heterozygosity as a measure of genetic diversity, so it is important to develop Shannon’s information as a new tool for molecular ecology. Past efforts at making forecasts for Shannon’s information in specific molecular ecology scenarios mostly dealt with expectations for Shannon’s information at genetic equilibrium, but dynamic forecasts are also vital. In particular, we must be able to predict loss of genetic diversity when dealing with finite populations, because they risk losing genetic variability, which can have an adverse effect on their survival. We present equations for predicting loss of genetic diversity measured by Shannon’s information. We also provide statistical justification for these models by assessing their fit to data derived from simulations and managed, replicated laboratory populations. The predictive models will enhance the usefulness of Shannon’s information as a measure of genetic diversity; they will also be useful in pest control and conservation. | NA | no | No good fitness measure and measure host GD | NA | no | no |
| 5420 | 2020 | 44112 | Obodai, E; Odoom, JK; Adiku, T; Goka, B; Wolff, T; Biere, B; Schweiger, B; Reiche, J | 2018 | The significance of human respiratory syncytial virus (HRSV) in children from Ghana with acute lower respiratory tract infection: A molecular epidemiological analysis, 2006 and 2013-2014 | Background Acute lower respiratory tract infection (ALRI) is a leading cause of childhood morbidity and mortality in developing countries. Globally, human respiratory syncytial virus (HRSV) is the most common pathogen of ALRI in infants and children. However, age-stratified HRSV disease burden data are largely absent from Africa, which is a key gap in informing an evidence-based recommendation for the introduction of an HRSV vaccine by the WHO. Methods This study investigated the presence of HRSV in respiratory specimens from 552 children <5 years old with ALRI from Accra, Ghana in 2006 and 2013-2014 by real-time PCR. Of HRSV-positive samples the second hypervariable region of the viral G protein gene was sequenced and analyzed for phylogeny, characteristic amino acid substitutions, and potential glycosylation patterns. Further, HRSV infections have been characterized by age, symptoms and timely occurrence. Results HRSV was observed in 23% (127/552) of the children with ALRI, with the highest incidence in infants younger than one year (33%, 97/295, p = 0.013). Within the observed seasonal circulation time of HRSV from June (mid-wet season) to December (beginning of the dry season) the incidence of ALRI due to HRSV was as high as 46% (125/273). HRSV disease was significantly associated with (broncho-) pneumonia, bronchiolitis, LRTI, and difficulty in breathing. Phylogenetic characterization of HRSV strains from Ghana identified the circulation of the currently worldwide prevailing genotypes ON1 and BA9, and shows evidence of an independent molecular evolution of ON1 and BA9 strains in Ghana resulting in potentially new subgenotypes within ON1 and BA9, provisionally named ON1.5, ON1.6, and BA9-IV. Conclusion This study addresses important knowledge gaps in the forefront of introducing the HRSV vaccine by providing information on the molecular evolution and incidence of HRSV in Accra (Ghana, Africa). | NA | no | gd of pathogen | NA | no | no |
| 5421 | 2020 | 44112 | Oguz, AC; Olmez, F; Karakaya, A | 2019 | Genetic Diversity of Net Blotch Pathogens of Barley in Turkey | Pyrenophora teres f. maculata (Ptm) (spot form of net blotch) and Pyrenophora teres f. teres (Ptt) (net form of net blotch) are common leaf pathogens that cause net blotch disease of barley. From different regions of Turkey during the years 2012-2016 and 2017,48 Ptt and 49 Ptm single spore isolates were obtained from different Hordeum species W. vulgare (40 Ptm, 47 Ptt), H. spontaneum (7 Ptm, 1 Ptt) and H. bulbosum (2 Ptm)]. The genetic diversity of the isolates was accomplished using inter simple sequence repeat (ISSR) marker system. Using the species-specific and mating-type specific PCR primers, 48 Ptt and 49 Ptm isolates were identified as 30 MAT 1-1, 18 MAT1-2 and 27 MAT1-1, 22 MAT1-2 types, respectively. In order to determine genetic diversity of isolates, 10 ISSR primers were used and seven of these primers showed optimal band numbers and intensities. The most useful primers were UBC 825 and UBC 807 for Ptt population and UBC 825, UBC 807 and UBC 827 for Ptm population. The similarity rate of the Ptm isolates was found to be lower than that of Ptt isolates. There was no definite separation by year, mating type or geographical area. However, Ptt population was clustered more by location. The similarity rate of isolates obtained from wild barleys collected from Southeast Anatolia region of Turkey located in the Fertile Crescent region were low. Although some isolates from the same wild hosts and provinces showed low similarity, some isolates obtained from different wild hosts showed higher similarity to each other. This variation in isolates from wild barleys may contribute to the genetic diversity of the P. teres population. Genetic diversity of Ptt and Ptm populations in Turkey was assessed for the first time with this study. Investigation of pathogen biology and genetic structure will be useful for the development of disease control strategies. (C) 2019 Friends Science Publishers | NA | no | dometicated host | NA | no | no |
| 5422 | 2020 | 44112 | Okamiya, H; Kusano, T | 2018 | Lower genetic diversity and hatchability in amphibian populations isolated by urbanization | Many amphibian populations worldwide have declined rapidly and been threatened with extinction in the past few decades because of human impacts on the environment. It is well known that urbanization reduces the genetic diversity of isolated populations. The concept that a reduction in genetic diversity leads to lower reproductive fitness has been predominantly supported by studies involving laboratory organisms, but has rarely been tested in wild populations. Here we examined whether genetic diversity affected hatchability, a population parameter related to reproductive success, in the populations of two pond-breeding amphibian species (Hynobius tokyoensis and Rana ornativentris) affected by urbanization. We surveyed 81 populations in the southwestern Kanto region of Japan. Mean hatchability of the populations was determined and genetic diversities were estimated via mitochondrial or microsatellite DNA analyses. Firstly, Random Forests (an ensemble machine learning method) models were applied to clarify the effects of environmental factors on both hatchability and genetic diversity. Subsequently, the relationships among environmental factors, genetic diversity, and mean hatchability were evaluated using path analysis. Mean hatchability was significantly affected by both urbanization and genetic diversity. This result shows that loss of genetic diversity may decrease a population’s reproductive success in the field. | NA | no | No good fitness measure | NA | no | no |
| 5423 | 2020 | 44111 | Okamura, B; Hartikainen, H; Trew, J | 2019 | Waterbird-Mediated Dispersal and Freshwater Biodiversity: General Insights From Bryozoans | Freshwater environments are fragmented and heterogeneous in space and time. Long term persistence thus necessitates at least occasional dispersal of aquatic organisms to locate suitable habitats. However, the insubstantial movements of many require zoochory-hitchhiking a ride with more mobile animals. We review evidence for waterbird-mediated zoochory of freshwater bryozoans, a group that provides an excellent model for addressing this issue. The feasibility of long distance transport by waterbirds of bryozoan propagules (statoblasts) is evaluated in relation to statoblast resistance to extreme conditions and waterbird gut retention times, flight durations and distances. We highlight genetic evidence for colonization following waterbird-mediated transport. The consequences of zoochory for biodiversity are manifold. Taxa that release statoblasts show lower levels of genetic differentiation, genetic divergence and haplotype diversity than those whose statoblasts are retained in situ (hence less available for zoochory). Zoochory may also disseminate pathogens and parasites when infected host stages are transported. Such co-dispersal may explain some disease distributions and is supported by viability of infected statoblasts. Zoochory can also be expected to influence local and regional population and community dynamics, food web structure and stability, and organismal distributions, and abundances. Finally, zoochory may influence host-parasite coevolution and disease dynamics across the landscape with the benefits to parasites depending on their life history (e.g., simple vs. complex life cycles, generalists vs. specialists). Our synthesis highlights the complex ecological and evolutionary impacts of zoochory of freshwater organisms and raises questions for future research. | NA | no | No good fitness measure and measure host GD | NA | no | no |
| 5424 | 2020 | 44113 | Oklander, LI; Mino, CI; Fernandez, G; Caputo, M; Corach, D | 2017 | Genetic structure in the southernmost populations of black-and-gold howler monkeys (Alouatta caraya) and its conservation implications | Black-and-gold howler monkeys Alouatta caraya, are arboreal primates, inhabitants of Neotropical forests, highly susceptible to the yellow fever virus, considered early ‘sentinels’ of outbreaks, and thus, of major epidemiological importance. Currently, anthropogenic habitat loss and modifications threatens their survival. Habitat modification can prevent, reduce or change dispersal behavior, which, in turn, may influence patterns of gene flow. We explored past and contemporary levels of genetic diversity, elucidated genetic structure and identified its possible drivers, in ten populations (n = 138) located in the southernmost distribution range of the species in South America, in Argentina and Paraguay. Overall, genetic variability was moderate (ten microsatellites: 3.16 +/- 0.18 alleles per locus, allelic richness of 2.93 +/- 0.81, 0.443 +/- 0.025 unbiased expected heterozygosity; 22 haplotypes of 491-bp mitochondrial Control Region, haplotypic diversity of 0.930 +/- 0.11, and nucleotide diversity of 0.01 +/- 0.007). Significant evidence of inbreeding was found in a population that was, later, decimated by yellow fever. Population-based gene flow measures (FST = 0.13;. ST = 018), hierarchical analysis of molecular variance and Bayesian clustering methods revealed significant genetic structure, grouping individuals into four clusters. Shared haplotypes and lack of mitochondrial differentiation (non-significant. ST) among some populations seem to support the hypothesis of historical dispersal via riparian forests. Current resistance analyses revealed a significant role of landscape features in modeling contemporary gene flow: continuous forest and riparian forests could promote genetic exchange, whereas disturbed forests or crop/grassland fields may restrict it. Estimates of effective population size allow anticipating that the studied populations will lose 75% of heterozygosity in less than 50 generations. Our findings suggest that anthropogenic modifications on native forests, increasingly ongoing in Northeastern Argentina, Southern Paraguay and Southeastern Brazil, might prevent the dispersal of howlers, leading to population isolation. To ensure long-term viability and maintain genetic connectivity of A. caraya remnant populations, we recommend preserving and restoring habitat continuity. To conserve the species genetic pool, as well, the four genetic clusters identified here should be considered separate Management Units and given high conservation priority. In light of our findings and considering complementary non-genetic information, we suggest upgrading the international conservation status of A. caraya to Vulnerable. | NA | no | inbreeding | NA | no | no |
| 5425 | 2020 | 44039 | Olarte, O; Sanchez-Montes, G; Martinez-Solano, I | 2020 | Integrative demographic study of the Iberian painted frog (Discoglossus galganoi): inter-annual variation in the effective to census population size ratio, with insights on mating system and breeding success | In the face of worldwide amphibian declines, integrative studies combining individual-based information and genetic data represent a powerful approach to produce robust, reliable, and comparable assessments of demographic dynamics. The Iberian painted frog (Discoglossus galganoi) is endemic to Spain and Portugal and shows decreasing population trends across its range, but few studies have attempted to estimate census sizes or assess genetic diversity in wild populations, and little is known about their reproductive biology. We applied an integrative approach based on the combination of capture-mark-recapture data and multilocus genotypes to monitor a breeding population ofD. galganoiin central Spain during two consecutive breeding seasons, focusing on the estimation of demographic parameters and their temporal variation. Specifically, we estimated the number of adults (N-a), the effective population size (N-e), and the effective number of breeders (N-b), as well as survival and migration rates. We documented a >50% decrease in the estimated number of adults of both sexes between the breeding seasons of 2018 and 2019, probably associated with reduced rainfall in the latter. Estimates ofN(b)and theN(b)/N(a)ratio were low in both seasons, with a 20-30% decrease inN(b)and a 47% increase in theN(b)/N(a)ratio in 2019. Based on the reconstruction of pedigrees from larval and adult genotypes, we provide the first genetic evidence of polygamy in males and females ofD. galganoiand the first estimates of breeding success in the species. | NA | no | Only one pop? (but twp seasons) | NA | no | no |
| 5426 | 2020 | 44040 | Old, JM; Hermsen, EM; Young, LJ | 2020 | MHC Class II variability in bare-nosed wombats (Vombatus ursinus) | Studies of gene diversity are used to investigate population dynamics, including immunological fitness. Aside from the selection of an appropriate gene target, an important factor that underpins these studies is the ability to recover viable DNA samples from native animals that are protected, threatened or difficult to sample or locate such as the bare-nosed wombat (Vombatus ursinus). In this study, we used genomic DNA extracted from muscle tissue samples and also identified the optimal method to extract DNA from fresh wombat scat samples to enable further analyses to be performed using non-invasive techniques. The DNA was probed via the polymerase chain reaction using previously targeted marsupial Major Histocompatibility Complex (MHC) gene primers. These genes are highly variable and associated with binding and presentation of pathogens in the immune system. Twenty-three unique MHC Class II DAB V. ursinus gene sequences were translated to 21 unique predicted peptide sequences from 34 individual tissue or scat samples. Vombatus ursinus MHC Class II DAB gene and peptide sequences were most similar to other marsupial DNA and peptide sequences. Further analysis also indicated the likelihood of MHC Class II DAB family membership through motif identification. Additional sampling is required to assess the full level of diversity of MHC Class II DAB genes among V. ursinus populations; however, this study is the first to identify MHC genes in a wombat and will advance immunological and disease studies of the species. | NA | no | n | NA | no | no |
| 5427 | 2020 | 44040 | Oliveira, AS; dos Reis, EF; Nogueira, APO; Juliatti, FC | 2020 | INBREEDING DEPRESSION AND GENETIC VARIABILITY IN AGRICULTURAL TRAITS AND RESISTANCE TO PLANT PATHOGENS IN EXOTIC MAIZE POPULATIONS | The effect known as depression by inbreeding refers to the reduction on the average value of quantitative traits, related to plant reproduction and physiology, due to the homozygosis of deleterious alleles. This study evaluated the inbreeding depression and the genetic variability of agricultural traits and of the resistance to phytopathogens in inbred families of two exotic maize populations. The experiments were done in the experimental area of the Universidade Federal de Goias, Regional Jatai, in the second harvest 2015. Fifty and 40 F-1 of NAPS and NAP7 populations, respectively, were evaluated interplanting one row with a mixture of base population (family of Germanic siblings) at every ten plots to estimate depression by inbreeding of the traits evaluated. The experimental design was randomized blocks, with three replications. The following traits were evaluated: PH - plant height (cm), EH - ear height (cm), MF - male flowering (days), BP - number of broken plants, LP - number of lodged plants, KW - kernel weigh (kg plot(-1)), ET - resistance to Exserohilum turcicum, WS(White Spot) - resistance to Pantoea ananatis, CS(Corn Stunt) - resistance to the corn stunt, PP - resistance to Puccinia polysora and CZ - resistance to Cercospora zea-maydis. The greatest estimates of depression by inbreeding in the agricultural traits were observed for kernel production, with values of 51.2 and 38.9% for the populations NAPS and NAP7, respectively. Among the traits of resistance to phytopathogens, the greatest estimate was observed for the stunting complex, with values of -58.9% in NAPS and -74.2% in NAP7. Both populations under study presented genetic potential to be used in breeding programs with recurrent selection and, after some selection cycles, lineages with good agricultural standard and resistance to phytopathogens can be obtained. | NA | no | inbreeding | NA | no | no |
| 5428 | 2020 | 44111 | Oliveira, H; Sampaio, M; Melo, LDR; Dias, O; Pope, WH; Hatfull, GF; Azeredo, J | 2019 | Staphylococci phages display vast genomic diversity and evolutionary relationships | BackgroundBacteriophages are the most abundant and diverse entities in the biosphere, and this diversity is driven by constant predator-prey evolutionary dynamics and horizontal gene transfer. Phage genome sequences are under-sampled and therefore present an untapped and uncharacterized source of genetic diversity, typically characterized by highly mosaic genomes and no universal genes. To better understand the diversity and relationships among phages infecting human pathogens, we have analysed the complete genome sequences of 205 phages of Staphylococcus sp.ResultsThese are predicted to encode 20,579 proteins, which can be sorted into 2139 phamilies (phams) of related sequences; 745 of these are orphams and possess only a single gene. Based on shared gene content, these phages were grouped into four clusters (A, B, C and D), 27 subclusters (A1-A2, B1-B17, C1-C6 and D1-D2) and one singleton. However, the genomes have mosaic architectures and individual genes with common ancestors are positioned in distinct genomic contexts in different clusters. The staphylococcal Cluster B siphoviridae are predicted to be temperate, and the integration cassettes are often closely-linked to genes implicated in bacterial virulence determinants. There are four unusual endolysin organization strategies found in Staphylococcus phage genomes, with endolysins predicted to be encoded as single genes, two genes spliced, two genes adjacent and as a single gene with inter-lytic-domain secondary translational start site. Comparison of the endolysins reveals multi-domain modularity, with conservation of the SH3 cell wall binding domain.ConclusionsThis study provides a high-resolution view of staphylococcal viral genetic diversity, and insights into their gene flux patterns within and across different phage groups (cluster and subclusters) providing insights into their evolution. | NA | no | No good fitness measure and measure host GD | NA | no | no |
| 5429 | 2020 | 44111 | Olm, MAK; Marson, FAL; Athanazio, RA; Nakagawa, NK; Macchione, M; Loges, NT; Omran, H; Rached, SZ; Bertuzzo, CS; Stelmach, R; Saldiva, PHN; Ribeiro, JD; Jones, MH; Mauad, T | 2019 | Severe pulmonary disease in an adult primary ciliary dyskinesia population in Brazil | Primary Ciliary Dyskinesia (PCD) is underdiagnosed in Brazil. We enrolled patients from an adult service of Bronchiectasis over a two-year period in a cross-sectional study. The inclusion criteria were laterality disorders (LD), cough with recurrent infections and the exclusion of other causes of bronchiectasis. Patients underwent at least two of the following tests: nasal nitric oxide, ciliary movement and analysis of ciliary immunofluorescence, and genetic tests (31 PCD genes + CFTR gene). The clinical characterization included the PICADAR and bronchiectasis scores, pulmonary function, chronic Pseudomonas aeruginosa (cPA) colonization, exhaled breath condensate (EBC) and mucus rheology (MR). Forty-nine of the 500 patients were diagnosed with definite (42/49), probable (5/49), and clinical (2/49) PCD. Twenty-four patients (24/47) presented bi-allelic pathogenic variants in a total of 31 screened PCD genes. A PICADAR score > 5 was found in 37/49 patients, consanguinity in 27/49, LD in 28/49, and eight PCD sibling groups. FACED diagnosed 23/49 patients with moderate or severe bronchiectasis; FEV1 <= 50% in 25/49 patients, eight patients had undergone lung transplantation, four had been lobectomized and cPA+ was determined in 20/49. The EBC and MR were altered in all patients. This adult PCD population was characterized by consanguinity, severe lung impairment, genetic variability, altered EBC and MR. | NA | no | human | NA | no | no |
| 5430 | 2020 | 44111 | Ontiveros, ES; Hughes, S; Penedo, MCT; Grahn, RA; Stern, JA | 2019 | Genetic heterogeneity and diversity of North American golden retrievers using a low density STR marker panel | Thirty-three autosomal short tandem repeat (STR) markers were used to evaluate genetic heterogeneity and diversity in 525 golden retrievers (GRs). This breed was selected because of its popularity and artificial selection for conformation vs. performance phenotypes. Seven additional STRs were used to evaluate the highly polymorphic dog leukocyte antigen (DLA) class I and class II regions. From 3 to 13 alleles were found at each of the 33 loci (mean 7) and the average effective alleles (Ne) was 3.34. The observed heterozygosity was 0.65 and the expected heterozygosity was 0.68. The resulting fixation index was 0.035 indicating that the population was randomly breeding. We found that modern GRs retain 46% of genomic diversity present in all canids and 21/175 (12%) and 20/90 (22%) of the known DLA class I and class II haplotypes, respectively. Selection for performance or conformation led to a narrowing of genomic and DLA diversity with conformation having a greater effect than performance. A comparison was made between coefficient of inbreeding (COI) determined from 10 or 12 generation pedigrees and DNA based internal relatedness values. A weak but significant correlation was observed between IR score and 10 or 12 generation COI (r = 0.38, p<0.0001 and r = 0.40, p<0.0001, respectively). IR values were higher in conformation than performance lines but only significant at p = 0.17. This was supported by 10 and 12 generation COI values that were significantly (p<0.0001) higher in conformation than performance lines. We demonstrate herein that a low density of STR markers can be utilized to study the genetic makeup of GRs. | NA | no | dometicated host | NA | no | no |
| 5431 | 2020 | 44111 | Orlova, SY; Smirnova, MA; Stroganov, AN; Mukhametov, IN; Smirnov, AA; Tok, KS; Park, JH; Orlov, AM | 2019 | Population Structure and Microevolution of Pacific Cod Gadus macrocephalus Based on the Analysis of the Control Region (mtDNA) Polymorphism | We performed a population genetic analysis of 22 Pacific cod sample sets (n = 986) based on the mtDNA control region (599 bp) polymorphism, which made it possible to specify four distinguished groups of this species: (1) within the seaward parts of the Bering Sea and Sea of Okhotsk and northern part of the Pacific Ocean, affected by the Pacific Ocean gyres; (2) coastal areas of the East/Japan Sea; (3) northern part of the Sea of Okhotsk, more similar to the second group from the East/Japan Sea by haplotype sets than to the seaward groups, despite limited reproductive contacts, special hydrological conditions, and the history of formation of the sea areas; (4) fundamentally different Pacific cod from the Yellow Sea adapted to the conditions of the shallow water area in the subtropical region. | NA | no | No good fitness measure and measure host GD | NA | no | no |
| 5432 | 2020 | 44113 | Ortiz-Alvarez, R; Triado-Margarit, X; Camarero, L; Casamayor, EO; Catalan, J | 2018 | High planktonic diversity in mountain lakes contains similar contributions of autotrophic, heterotrophic and parasitic eukaryotic life forms | A rich eukaryotic planktonic community exists in high-mountain lakes despite the diluted, oligotrophic and cold, harsh prevailing conditions. Attempts of an overarching appraisal have been traditionally hampered by observational limitations of small, colorless, and soft eukaryotes. We aimed to uncover the regional eukaryotic biodiversity of a mountain lakes district to obtain general conclusions on diversity patterns, dominance, geographic diversification, and food-web players common to oligotrophic worldwide distributed freshwater systems. An unprecedented survey of 227 high-altitude lakes comprising large environmental gradients was carried out using Illumina massive tag sequencing of the 18S rRNA gene. We observed a large Chrysophyceae dominance in richness, abundance and novelty, and unveiled an unexpected richness in heterotrophic phagotrophs and parasites. In particular, Cercozoa and Chytridiomycota showed diversity features similar to the dominant autotrophic groups. The prominent beta-dispersion shown by parasites suggests highly specific interactions and a relevant role in food webs. Interestingly, the freshwater Pyrenean metacommunity contained more diverse specific populations than its closest marine oligotrophic equivalent, with consistently higher betadiversity. The relevance of unseen groups opens new perspectives for the better understanding of planktonic food webs. Mountain lakes, with remarkable environmental idiosyncrasies, may be suitable environments for the genetic diversification of microscopic eukaryotic life forms. | NA | no | No good fitness measure and measure host GD | NA | no | no |
| 5433 | 2020 | 44040 | Osorio-Guarin, JA; Berdugo-Cely, JA; Coronado-Silva, RA; Baez, E; Jaimes, Y; Yockteng, R | 2020 | Genome-Wide Association Study Reveals Novel Candidate Genes Associated with Productivity and Disease Resistance to Moniliophthora spp. in Cacao (Theobroma cacao L.) | Cacao (Theobroma cacao L.), the source of chocolate, is one of the most important commodity products worldwide that helps improve the economic livelihood of farmers. Diseases like frosty pod rot caused by Moniliophthora roreri and witches’ broom caused by Moniliophthora perniciosa limit the cacao productivity, this can be solved by using resistant varieties. In the current study, we sequenced 229 cacao accessions using genotyping-by-sequencing to examine the genetic diversity and population structure employing 9,003 and 8,131 single nucleotide polymorphisms recovered by mapping against two cacao genomes (Criollo B97-61/B2 v2 and Matina 1-6 v1.1). In the phenotypic evaluation, three promising accessions for productivity and 10 with good tolerance to the frosty pod rot and witches’ broom diseases were found. A genome-wide association study was performed on 102 accessions, discovering two genes associated with productivity and seven to disease resistance. The results enriched the knowledge of the genetic regions associated with important cacao traits that can have significant implications for conservation and breeding strategies like marker-assisted selection. | NA | no | No good fitness measure and measure host GD | NA | no | no |
| 5434 | 2020 | 44041 | Owings, AC; Fernandes, SB; Olatoye, MO; Fogleman, AJ; Zahnd, WE; Jenkins, WD; Malhi, RS; Lipka, AE | 2019 | Population Structure Analyses Provide Insight into the Source Populations Underlying Rural Isolated Communities in Illinois | We have previously hypothesized that relatively small and isolated rural communities may experience founder effects, defined as the genetic ramifications of small population sizes at the time of a community’s establishment. To explore this, we used an Illumina Infinium Omni2.5Exome-8 chip to collect data from 157 individuals from four Illinois communities, three rural and one urban. Genetic diversity estimates of 999,259 autosomal markers suggested that the reduction in heterozygosity due to shared ancestry was approximately 0, indicating a randomly mating population. An eigenanalysis, which is similar to a principal component analysis but run on a genetic coancestry matrix, conducted in the SNPRelate R package revealed that most of these individuals formed one cluster, with a few putative outliers obscuring population variation. An additional eigenanalysis on the same markers in a combined data set including the 2,504 individuals in the 1000 Genomes database found that most of the 157 Illinois individuals clustered into one group in close proximity to individuals of European descent. A final eigenanalysis of the Illinois individuals with the 503 individuals of European descent (within the 1000 Genomes Project) revealed two clusters of individuals and likely two source populations; one British and one consisting of multiple European subpopulations. We therefore demonstrate the feasibility of examining genetic relatedness across Illinois populations and assessing the number of source populations using publicly available databases. When assessed, population structure information can contribute to the understanding of genetic history in rural populations. | NA | no | human | NA | no | no |
| 5435 | 2020 | 44113 | Oyler-McCance, SJ; Fike, JA; Lukacs, PM; Sparks, DW; O’Shea, TJ; Whitaker, JO | 2018 | Genetic Mark-Recapture Improves Estimates of Maternity Colony Size for Indiana Bats | Genetic mark-recapture methods are increasingly being used to estimate demographic parameters in species where traditional techniques are problematic or imprecise. The federally endangered Indiana bat Myotis sodalis has declined dramatically and threats such as white-nose syndrome continue to afflict this species. To date, important demographic information for Indiana bats has been difficult to estimate precisely using traditional techniques such as emergence counts. Successful management and protection of Indiana bats requires better methods to estimate population sizes and survival rates throughout the year, particularly during summer when these bats reproduce and are widely dispersed away from their winter hibernacula. In addition, the familial makeup of maternity colonies is unknown, yet important for understanding local and regional population dynamics. We had four objectives in this study. For the first two objectives we investigated the potential use of DNA from fecal samples (fecal DNA) collected at roosts to obtain genetically based mark-recapture estimates of 1) colony size and 2) survival rates, for an Indiana bat maternity colony in Indianapolis, Indiana. The third objective was to compare our genetically based colony-size estimates with emergence counts conducted at the same roost tree to evaluate the genetic mark-recapture method. Our fourth objective was to use fecal DNA to estimate levels of relatedness among individuals sampled at the roost. In the summer of 2008, we collected fecal pellets and conducted emergence counts at a prominent roost tree during three time periods each lasting 7 or 8 d. We genotyped fecal DNA using five highly polymorphic microsatellite loci to identify individuals and used a robust-design mark-recapture approach to estimate survival rates as well as colony size at the roost tree. Emergence count estimates at the roost tree ranged from 100 to 215, whereas genetic mark-recapture estimates were higher, ranging from 122 to 266 and more precise. Apparent survival was 0.994 (SE = 0.04) between sampling periods suggesting that few bats died or permanently emigrated during the course of the study. Relatedness estimates, r, between all pairs of individuals averaged 0.055 ranging from 0 to 0.779, indicating that most individuals were not closely related. We demonstrate here the promise of using fecal DNA to estimate demographic information for Indiana bats and potentially other bat species. | NA | no | No good fitness measure and measure host GD | NA | no | no |
| 5436 | 2020 | 44111 | Ozkilinc, H; Can, CA | 2019 | The most recent status of genetic structure of Didymella rabiei (Ascochyta rabiei) populations in Turkey and the first genotype profile of the pathogen from the wild ancestor, Cicer reticulatum | Chickpea is an important legume crop cultivated in many locations of Turkey and wild relatives of chickpea naturally grow in the Southeastern Anatolia Region of Turkey. Ascochyta blight caused by Didymella rabiei is one of the most important limiting factors for chickpea production. In this study, we aimed to investigate the genetic structures of D. rabiei samples from wild and domesticated chickpea hosts from different geographical regions. For this purpose, D. rabiei was collected from different geographic regions of Turkey between March and June in 2014 and 2015. Besides, some isolates were obtained from the wild chickpea species, C. pinnatifidum and C. reticulatum. Total, one hundred and two isolates of D. rabiei were genotyped using six sequence tagged microsatellite (STMS) markers. According to the results of molecular variance, the pathogen isolates from different geographic regions showed a significant genetic variation, but, most of which were confined within the populations. The total genetic diversity was estimated to be 0.612 for D. rabiei isolates from chickpea over the country. For the first time, D. rabiei from C. reticulatum, which is the wild ancestor of domesticated chickpea was found in the world and the isolates from C. reticulatum were represented with a single genotype. Isolates from C. pinnatifidum were genetically closer to the isolates from Southeastern Anatolia Region. All the isolates were grouped as one genetic population according to the Bayesian algorithm and presented a mixed distribution based on the principal coordinate analysis (PCoA) of genetic distances. All the results represented and updated the information about the genetic structure of D. rabiei populations across the country by announcing new information about the isolates from wild relatives. In addition, STMS markers were utilized to test whether allele sizes of microstallite loci change during asexual reproduction in infection period within the host. Thus, the changes in pathogen genotype was traced from seed to seedling. To do tracing, chickpea seeds were treated with two different D. rabiei isolates with known STMS multilocus genotypes. Then, pathogen re- isolations were performed from the plants grown from the infected seeds and these re-isolates were genotyped with the same STMS markers. Allele sizes of re-isolates were different from the parental ones for some of the loci which indicated that microsatellite alleles may have changed during multiple cycles of asexual reproduction through host infection. | NA | no | dometicated host | NA | no | no |
| 5437 | 2020 | 44040 | Pabijan, M; Palomar, G; Antunes, B; Antol, W; Zielinski, P; Babik, W | 2020 | Evolutionary principles guiding amphibian conservation | The Anthropocene has witnessed catastrophic amphibian declines across the globe. A multitude of new, primarily human-induced drivers of decline may lead to extinction, but can also push species onto novel evolutionary trajectories. If these are recognized by amphibian biologists, they can be engaged in conservation actions. Here, we summarize how principles stemming from evolutionary concepts have been applied for conservation purposes, and address emerging ideas at the vanguard of amphibian conservation science. In particular, we examine the consequences of increased drift and inbreeding in small populations and their implications for practical conservation. We then review studies of connectivity between populations at the landscape level, which have emphasized the limiting influence of anthropogenic structures and degraded habitat on genetic cohesion. The rapid pace of environmental changes leads to the central question of whether amphibian populations can cope either by adapting to new conditions or by shifting their ranges. We gloomily conclude that extinction seems far more likely than adaptation or range shifts for most species. That said, conservation strategies employing evolutionary principles, such as selective breeding, introduction of adaptive variants through translocations, ecosystem interventions aimed at decreasing phenotype-environment mismatch, or genetic engineering, may effectively counter amphibian decline in some areas or for some species. The spread of invasive species and infectious diseases has often had disastrous consequences, but has also provided some premier examples of rapid evolution with conservation implications. Much can be done in terms of setting aside valuable amphibian habitat that should encompass both natural and agricultural areas, as well as designing protected areas to maximize the phylogenetic and functional diversity of the amphibian community. We conclude that an explicit consideration and application of evolutionary principles, although certainly not a silver bullet, should increase effectiveness of amphibian conservation in both the short and long term. | NA | no | inbreeding | NA | no | no |
| 5438 | 2020 | 44042 | Pacioni, C; Wayne, AF; Page, M | 2019 | Guidelines for genetic management in mammal translocation programs | Adequate levels of genetic diversity are important for the long-term success of translocated populations. Typically, population genetic theory and mathematical models are used to inform expected outcomes in different contexts. However, difficulties arise when trying to translate theoretical expectations into management actions. Providing practical guidelines on how to maximise the genetic diversity of translocated populations will help bridge this gap. In this study we develop guidelines for genetic management in translocation programs that consider genetic dynamics associated with population establishment, the harvest of founders from a source population, and the supplementation of an existing population over eight generations. Guidelines were informed by individual-based modelling. Given the nature of the modelling framework that we adopted, we report results in terms of the actual number of individuals and genetic diversity parameters as these are estimated in field-based studies. Our results demonstrate that 10 releases of 50 or two releases of 100 individuals should be carried out to establish a new population. Repeated harvests (each) of > 30% of a source population within a generation had a negative impact on its genetic diversity and demographics. The survival of > 20% of the supplemented individuals was needed for the supplementation program to be effective. Concurrently, the survival of resident animals also had a major effect. We make available a R utility to explore potential outcomes under different management scenarios. We considered our results to be directly applicable to polygamous, continuous breeder species, and generally informative for a wide range of vertebrate species. | NA | no | No good fitness measure and measure host GD | NA | no | no |
| 5439 | 2020 | 44043 | Packer, C | 2019 | The African Lion: A Long History of Interdisciplinary Research | Over the past 50 years, lion research has covered a wide range of interdisciplinary topics involving extensive collaborations with scientists from over a dozen different fields. These collaborations have not only led to greater scientific understanding of disease dynamics, impacts of sport hunting on lion populations, and the interactions of lions and their prey, but also resulted in a large-scale disease-control program in rural Tanzania, new hunting policies in several African countries, widespread adoption of camera-traps as a conservation-management tool, new statistical and economic approaches to broadscale conservation approaches, and innovative local-level conservation interventions. | NA | no | No good fitness measure and measure host GD | NA | no | no |
| 5440 | 2020 | 44112 | Palittapongarnpim, P; Ajawatanawong, P; Viratyosin, W; Smittipat, N; Disratthakit, A; Mahasirimongkol, S; Yanai, H; Yamada, N; Nedsuwan, S; Imasanguan, W; Kantipong, P; Chaiyasirinroje, B; Wongyai, J; Toyo-oka, L; Phelan, J; Parkhill, J; Clark, TG; Hibberd, ML; Ruengchai, W; Palittapongarnpim, P; Juthayothin, T; Tongsima, S; Tokunaga, K | 2018 | Evidence for Host-Bacterial Co-evolution via Genome Sequence Analysis of 480 Thai Mycobacterium tuberculosis Lineage 1 Isolates | Tuberculosis presents a global health challenge. Mycobacterium tuberculosis is divided into several lineages, each with a different geographical distribution. M. tuberculosis lineage 1 (L1) is common in the high-burden areas in East Africa and Southeast Asia. Although the founder effect contributes significantly to the phylogeographic profile, co-evolution between the host and M. tuberculosis may also play a role. Here, we reported the genomic analysis of 480 L1 isolates from patients in northern Thailand. The studied bacterial population was genetically diverse, allowing the identification of a total of 18 sublineages distributed into three major clades. The majority of isolates belonged to L1.1 followed by L1.2.1 and L1.2.2. Comparison of the single nucleotide variant (SNV) phylogenetic tree and the clades defined by spoligotyping revealed some monophyletic clades representing EAI2\_MNL, EAI2\_NTM and EAI6\_BGD1 spoligotypes. Our work demonstrates that ambiguity in spoligotype assignment could be partially resolved if the entire DR region is investigated. Using the information to map L1 diversity across Southeast Asia highlighted differences in the dominant strain-types in each individual country, despite extensive interactions between populations over time. This finding supported the hypothesis that there is co-evolution between the bacteria and the host, and have implications for tuberculosis disease control. | NA | no | gd of pathogen | NA | no | no |
| 5441 | 2020 | 44040 | Palmer, N; Smith, MJ; Ruykys, L; Jackson, C; Volck, G; Riessen, N; Thomasz, A; Moir, C; Palmer, B | 2020 | Wild-born versus captive-bred: a comparison of survival and refuge selection by translocated numbats (Myrmecobius fasciatus) | Context. To re-establish a population of the threatened numbat (Myrmecobius fasciatus) in a newly created safe haven at Mount Gibson Wildlife Sanctuary, in the semi-arid zone of Western Australia, wild-born and captive-bred individuals of both sexes were translocated. Aim. To compare: (1) the use of refuges by wild-born numbats pre- and post-translocation, and (2) the survival and refuge use of captive-bred numbats compared with wild-sourced numbats post-translocation. Methods. Monitoring via radio-tracking facilitated the gathering of information on survival and behaviour of numbats pre- and post-translocation, and allowed a comparison of how different factors (e.g. captive-bred or wild-born; male or female) influenced survival and establishment in a new environment. Key results. There were no significant differences in survival between sexes or between wild-born and captive-bred individuals. However, there were some differences in behaviour between sexes and source populations. Captive-bred numbats, regardless of sex, made greater use of tree hollows as nocturnal refuges than did their wild-born counterparts. Among wild-born numbats, there was a comparatively greater use of tree hollows and logs on the ground by males at Mount Gibson than at Scotia. The use of diurnal escape refuges did not vary between sexes or between captive-bred and wild-born individuals. Conclusions. On the basis of the information presented here, we conclude that, in the absence of predation by mammalian predators, and with suitable release habitat, captive-bred-to-wild translocations of numbats may be as likely to succeed as are wild-to-wild translocations, at least over the first few months post-translocation. Implications. Optimising the size and genetic diversity of the founding population by using animals from a variety of sources may not need to be heavily constrained by concerns about the ability of captive-bred numbats to adapt to and survive life in the wild. | NA | no | n | NA | no | no |
| 5442 | 2020 | 44111 | Pantoja-Gomez, LM; Correa, AS; de Oliveira, LO; Guedes, RNC | 2019 | Common Origin of Brazilian and Colombian Populations of the Neotropical Coffee Leaf Miner, Leucoptera coffeella (Lepidoptera: Lyonetiidae) | The coffee leaf miner, Leucoptera coffeella (Gurin-Mneville & Perrottet, 1842), probably infested coffee plants in Neotropical America during the 19th century. The species subsequently became a key pest of coffee plants in Brazil, but not in Colombia, the two main coffee producers in the region. The contrasting importance of the coffee leaf miner in Brazil and Colombia may be the result of the evolutionary and demographic history of this species. Therefore, our goal was to test two alternative hypotheses regarding the possible genetic origins of this species: 1) leaf miners in both countries share the same origin and 2) the leaf miner arrived in both countries independently from distinct sources and subsequently diversified without genetic exchange between countries. Thus, DNA sequence data of 21 populations were collected (Brazil, 16; Colombia, 5), and partial sequences of their cytochrome oxidase subunit I (COI), cytochrome b (Cytb), and the nuclear internal transcribed spacer (ITS) region were obtained to test these hypotheses. Both nuclear and mitochondrial molecular markers showed low nucleotide diversity. Analyses of molecular variance indicated higher variability within population in both concatenated mitochondrial genes and ITS region (70.57 and 84.01%, respectively). Finally, geno/haplotype networks showed each central geno/haplotypes that displayed high frequency and were distributed widely in both countries. Low-frequency geno/haplotypes were at tip positions connected to the central geno/haplotypes through single mutation steps, suggesting that the Neotropical coffee leaf miner in both Brazil and Colombia consists of a single species and exhibits a common and recent genetic origin. | NA | no | No good fitness measure and measure host GD | NA | no | no |
| 5443 | 2020 | 44113 | Parada-Rojas, CH; Quesada-Ocampo, LM | 2018 | Analysis of microsatellites from transcriptome sequences of Phytophthora capsici and applications for population studies | Phytophthora capsici is a devastating oomycete that affects solanaceous, cucurbitaceous, fabaceous, and other crops in the United States (US) and worldwide. The release of the P. capsici genome allows for design of robust markers for genetic studies. We identified and characterized microsatellites in the P. capsici transcriptome. A subset of 50 microsatellites were assayed in a diverse set of P. capsici isolates and evaluated for polymorphism. Polymorphic microsatellites were confirmed by fragment analysis, and 12 were used for population characterization of 50 P. capsici isolates from different states, hosts, and mating types. Analysis of genetic relationship among isolates revealed significant geographic structure by state. Our findings highlight the usefulness of these 12 microsatellites to characterize the population structure of P. capsici and potential transferability to closely-related Phytophthora spp. since markers are located in coding regions. Our markers will facilitate genetic characterization and complement phenotypic studies of P. capsici populations, which may assist in deployment of disease management strategies. | NA | no | No good fitness measure | NA | no | no |
| 5444 | 2020 | 44111 | Parau, LG; Frias-Soler, RC; Wink, M | 2019 | High Genetic Diversity among Breeding Red-Backed Shrikes Lanius collurio in the Western Palearctic | Revealing the genetic population structure in abundant avian species is crucial for understanding speciation, conservation, and evolutionary history. The Red-backed Shrike Lanius collurio, an iconic songbird renowned for impaling its prey, is widely distributed as a breeder across much of Europe, Asia Minor and western Asia. However, in recent decades, many populations have declined significantly, as a result of habitat loss, hunting along migration routes, decrease of arthropod food, and climate change e.g., severe droughts in Africa. Within this context, gene flow among different breeding populations becomes critical to ensure the survival of the species, but we still lack an overview on the genetic diversity of the species. In this paper, we analyzed the mitochondrial cytochrome b gene (mtDNA) and the cytochrome c oxidase subunit 1 gene (mtDNA) of 132 breeding Red-backed Shrikes from across the entire breeding range to address this knowledge gap. Our results revealed consistent genetic diversity and 76 haplotypes among the Eurasian populations. Birds are clustered in two major groups, with no clear geographical separation, as a direct consequence of Pleistocene glaciations and apparent lineage mixing in refugia. This has led to genetic panmixia. | NA | no | No good fitness measure and measure host GD | NA | no | no |
| 5445 | 2020 | 44111 | Paredes-Montero, JR; Hameed, U; Zia-Ur-Rehman, M; Rasool, G; Haider, MS; Herrmann, HW; Brown, JK | 2019 | Demographic Expansion of the Predominant Bemisia tabaci (Gennadius) (Hemiptera: Aleyrodidae) Mitotypes Associated With the Cotton Leaf Curl Virus Epidemic in Pakistan | The association between Bemisia tabaci mitotypes and cotton leaf curl outbreaks in Pakistan was investigated using the mitochondria cytochrome oxidase I gene (COI) as a molecular marker. The 3’-651 base fragment has been used to resolve B. tabaci phylogenies. However, the 5’-618 base fragment was nearly unexplored. Phylogenetic analysis for 829 whiteflies from 11 districts in two provinces of Pakistan, indicated all haplotypes grouped on the Asia II major clade, with Asia II-1 mitotype predominating, at 84%, compared to Asia II-5 and II-7, at similar to 16%, combined. The 3’- and 5’-fragment tree topologies were similar, while the concatenated topology was unique in some respects. Comparisons of segregating sites within the 3’- and 5’-loci, at third codon positions, 71 and 47, and of transitions to transversions (Ti/Tv) ratio of 2.93 and 5.9, respectively, showed the 3’-locus was most informative, while nucleotide diversity (pi) was highest for the 5’-end, indicating both fragments contributed to concatenated tree structure. The extent of haplotype diversity, measured by Tajima’s D, R-2, and Fu’s F analyses, revealed significant demographic expansion for Asia II-1 and II-7 mitotypes. The bottleneck that preceded the expansions was evident in the temporal changes in mtCOI polymorphisms beginning in similar to 1990s, a timeframe known to have coincided with the adoption of a high-yield whitefly-susceptible cultivar in 1988, followed by pesticide overuse. These two cooperating phenomena appear to have exerted selection on the cotton leaf curl disease (CLCuD)-whitefly complex, resulting in the emergence of a resistance-breaking begomovirus as the polyphagous Asia II-1 mitotype underwent a genetic expansion that led to ‘a perfect storm’. | NA | no | gd of pathogen | NA | no | no |
| 5446 | 2020 | 44040 | Paredes-Montero, JR; Zia-Ur-Rehman, M; Hameed, U; Haider, MS; Herrmann, HW; Brown, JK | 2020 | Genetic variability, community structure, and horizontal transfer of endosymbionts among three Asia II-Bemisia tabaci mitotypes in Pakistan | Endosymbionts associated with the whitefly Bemisia tabaci cryptic species are known to contribute to host fitness and environmental adaptation. The genetic diversity and population complexity were investigated for endosymbiont communities of B. tabaci occupying different micro-environments in Pakistan. Mitotypes of B. tabaci were identified by comparative sequence analysis of the mitochondria cytochrome oxidase I (mtCOI) gene sequence. Whitefly mitotypes belonged to the Asia II-1, -5, and -7 mitotypes of the Asia II major clade. The whitefly-endosymbiont communities were characterized based on 16S ribosomal RNA operational taxonomic unit (OTU) assignments, resulting in 43 OTUs. Most of the OTUs occurred in the Asia II-1 and II-7 mitotypes (r(2) = .9, p < .005), while the Asia II-5 microbiome was less complex. The microbiome OTU groups were mitotype-specific, clustering with a basis in phylogeographical distribution and the corresponding ecological niche of their whitefly host, suggesting mitotype-microbiome co-adaptation. The primary endosymbiont Portiera was represented by a single, highly homologous OTU (0%-0.67% divergence). Two of six Arsenophonus OTUs were uniquely associated with Asia II-5 and -7, and one occurred exclusively in Asia II-1, two only in Asia II-5, and one in both Asia II-1 and -7. Four other secondary endosymbionts, Cardinium, Hemipteriphilus, Rickettsia, and Wolbachia OTUs, were found at <= 29% frequencies. The most prevalent Arsenophonus OTU was found in all three Asia II mitotypes (55% frequency), whereas the same strain of Cardinium and Wolbachia was found in both Asia II-1 and -5, and a single Hemipteriphilus OTU occurred in Asia II-1 and -7. This pattern is indicative of horizontal transfer, suggestive of a proximity between mitotypes sufficient for gene flow at overlapping mitotype ecological niches. | NA | no | No good fitness measure and measure host GD | NA | no | no |
| 5447 | 2020 | 44113 | Park, AW; Farrell, MJ; Schmidt, JP; Huang, S; Dallas, TA; Pappalardo, P; Drake, JM; Stephens, PR; Poulin, R; Nunn, CL; Davies, TJ | 2018 | Characterizing the phylogenetic specialism - generalism spectrum of mammal parasites | The distribution of parasites across mammalian hosts is complex and represents a differential ability or opportunity to infect different host species. Here, we take a macroecological approach to investigate factors influencing why some parasites show a tendency to infect species widely distributed in the host phylogeny (phylogenetic generalism) while others infect only closely related hosts. Using a database on over 1400 parasite species that have been documented to infect up to 69 terrestrial mammal host species, we characterize the phylogenetic generalism of parasites using standard effect sizes for three metrics: mean pairwise phylogenetic distance (PD), maximum PD and phylogenetic aggregation. We identify a trend towards phylogenetic specialism, though statistically host relatedness is most often equivalent to that expected from a random sample of host species. Bacteria and arthropod parasites are typically the most generalist, viruses and helminths exhibit intermediate generalism, and protozoa are on average the most specialist. While viruses and helminths have similar mean pairwise PD on average, the viruses exhibit higher variation as a group. Close-contact transmission is the transmission mode most associated with specialism. Most parasites exhibiting phylogenetic aggregation (associating with discrete groups of species dispersed across the host phylogeny) are helminths and viruses. | NA | no | No good fitness measure and measure host GD | NA | no | no |
| 5448 | 2020 | 44113 | Parkinson, JE; Bartels, E; Devlin-Durante, MK; Lustic, C; Nedimyer, K; Schopmeyer, S; Lirman, D; LaJeunesse, TC; Baums, IB | 2018 | Extensive transcriptional variation poses a challenge to thermal stress biomarker development for endangered corals | As climate changes, sea surface temperature anomalies that negatively impact coral reef organisms continue to increase in frequency and intensity. Yet, despite widespread coral mortality, genetic diversity remains high even in those coral species listed as threatened. While this is good news in many ways, it presents a challenge for the development of biomarkers that can identify resilient or vulnerable genotypes. Taking advantage of three coral restoration nurseries in Florida that serve as long-term common garden experiments, we exposed over 30 genetically distinct Acropora cervicornis colonies to hot and cold temperature shocks seasonally and measured pooled gene expression responses using RNAseq. Targeting a subset of 20 genes, we designed a high-throughput qPCR array to quantify expression in all individuals separately under each treatment with the goal of identifying predictive and/or diagnostic thermal stress biomarkers. We observed extensive transcriptional variation in the population, suggesting abundant raw material is available for adaptation via natural selection. However, this high variation made it difficult to correlate gene expression changes with colony performance metrics such as growth, mortality and bleaching susceptibility. Nevertheless, we identified several promising diagnostic biomarkers for acute thermal stress that may improve coral restoration and climate change mitigation efforts in the future. | NA | no | No good fitness measure and measure host GD | NA | no | no |
| 5449 | 2020 | 44113 | Parmar, DR; Mitra, S; Bhadouriya, S; Rao, T; Kunteepuram, V; Gaur, A | 2017 | Characterization of major histocompatibility complex class I, and class II DRB loci of captive and wild Indian leopards (Panthera pardus fusca) | The major histocompatibility complex (MHC), in vertebrate animals, is a multi-genic protein complex that encodes various receptors. During a disease, MHC interacts with the antigen and triggers a cascade of adaptive immune responses to overcome a disease outbreak. The MHC is very important region from immunological point of view, but it is poorly characterized among Indian leopards. During this investigation, we examined genetic diversity for MHC class I (MHC-I) and MHC class II-DRB (MHC-II) among wild and captive Indian leopards. This study estimated a pool of 9 and 17 alleles for MHC-I and MHC-II, respectively. The wild group of individuals showed higher nucleotide diversity and amino acid polymorphism compared to the captive group. A phylogenetic comparison with other felids revealed a clustering in MHC-I and interspersed presence in MHC-II sequences. A test for selection also revealed a deviation from neutrality at MHC-II DRB loci and higher non-synonymous substitution rate (dN) among the individuals from wild group. Further, the wild individuals showed higher dN for both MHC I and II genes compared to the group that was bred under captive conditions. These findings suggest the role of micro-evolutionary forces, such as pathogen-mediated selection, to cause MHC variations among the two groups of Indian leopards, because the two groups have been bred in two different environments for a substantial period of time. Since, MHC diversity is often linked with the quality of immunological health; the results obtained from this study fill the gap of knowledge on disease predisposition among wild and captive Indian leopards. | NA | no | mhc diversity | NA | no | no |
| 5450 | 2020 | 44113 | Parra, GJ; Cagnazzi, D; Jedensjo, M; Ackermann, C; Frere, C; Seddon, J; Nikolic, N; Krutzen, M | 2018 | Low genetic diversity, limited gene flow and widespread genetic bottleneck effects in a threatened dolphin species, the Australian humpback dolphin | Numerous species of marine megafauna are at risk of extinction and understanding their genetic population structure and demographic history is essential for their conservation. We used mitochondrial DNA and 18 nuclear microsatellite loci, on the largest genetic dataset compiled to date on Australian humpback dolphins (eight sampling sites, 159 samples), to assess their genetic diversity, gene flow and past demographic history along the east coast of Queensland, Australia. Levels of genetic diversity were low (mtDNA: h = 0-0.52, pi = 0-0.007; nDNA: Ho = 0.27-0.41; AR = 1.7-2.4). Both mitochondrial (Phi(ST) = 0.49, P = 0.001) and nuclear markers (F-ST = 0.14, P = 0.001) showed strong genetic structure among sampling sites. Four putative populations were identified, with little contemporary gene flow (m = 0.017 to 0.047) among populations. Genetic divergence follows an isolation-by-distance model (r = 0.38, P = 0.0001), with an apparent restriction in gene flow occurring at scales of 382,509 km. Estimates of contemporary effective population size were low (N-e = 11.5-31.2), with signatures of genetic bottlenecks for all putative populations about 50-150 generations ago. The current low levels of genetic diversity, gene flow, and effective population size in Australian humpback dolphins indicate the effects of historical population bottlenecks and/or founder events during the late Holocene period (similar to 1250-3750 years ago); probably associated with sea level fall and increased intensity of El Nino Southern Oscillation-climatic events. Our results raise important conservation concerns and emphasize the vulnerability of Australian humpback dolphins to stochastic demographic, genetic and environmental processes. Conservation strategies should focus on promoting connectivity among local populations and reducing direct causes of human related mortality. | NA | no | No good fitness measure | NA | no | no |
| 5451 | 2020 | 44042 | Patgiri, SJ; Sarma, K; Sarmah, N; Bhattacharyya, N; Sarma, DK; Nirmolia, T; Bhattacharyya, DR; Mohapatra, PK; Bansal, D; Bharti, PK; Sehgal, R; Mahanta, J; Sultan, AA | 2019 | Characterization of drug resistance and genetic diversity of Plasmodium falciparum parasites from Tripura, Northeast India | Monitoring of anti-malarial drug resistance is vital in Northeast India as this region shares its international border with Southeast Asia. Genetic diversity of Plasmodium parasites regulates transmission dynamics, disease severity and vaccine efficacy. P. falciparum chloroquine resistance transporter (Pfcrt), multidrug resistance-1 (Pfmdr-1) and kelch 13 propeller (PfK-13) genes which govern antimalarial drug resistance and three genetic diversity markers, merozoite surface protein 1 and 2 (Pfmsp-1, Pfmsp-2) and glutamate rich protein (Pfglurp) were evaluated from Tripura, Northeast India using molecular tools. In the Pfcrt gene, 87% isolates showed triple mutations at codons M74I, N75E and K76T. 12.5% isolates in Pfmdr-1 gene showed mutation at N86Y. No polymorphism in PfK13 propeller was found. Polyclonal infections were observed in 53.85% isolates and more commonly in adults (p = 0.0494). In the Pfmsp-1 locus, the K1 allelic family was predominant (71.2%) followed by the 3D7/IC family (69.2%) in the Pfmsp-2 locus. RI I region of Pfglurp exhibited nine alleles with expected heterozygosity of 0.85. The multiplicity of infection for Pfmsp-1, Pfmsp-2 and Pfglurp were 1.56,1.31 and 1.06 respectively. Overall, the study demonstrated a high level of chloroquine resistance and extensive parasite diversity in the region, necessitating regular surveillance in this population group. | NA | no | gd of pathogen | NA | no | no |
| 5452 | 2020 | 44041 | Patton, AH; Margres, MJ; Stahlke, AR; Hendricks, S; Lewallen, K; Hamede, RK; Ruiz-Aravena, M; Ryder, O; McCallum, HI; Jones, ME; Hohenlohe, PA; Storfer, A | 2019 | Contemporary Demographic Reconstruction Methods Are Robust to Genome Assembly Quality: A Case Study in Tasmanian Devils | Reconstructing species’ demographic histories is a central focus of molecular ecology and evolution. Recently, an expanding suite of methods leveraging either the sequentially Markovian coalescent (SMC) or the site-frequency spectrum has been developed to reconstruct population size histories from genomic sequence data. However, few studies have investigated the robustness of these methods to genome assemblies of varying quality. In this study, we first present an improved genome assembly for the Tasmanian devil using the Chicago library method. Compared with the original reference genome, our new assembly reduces the number of scaffolds (from 35,975 to 10,010) and increases the scaffold N90 (from 0.101 to 2.164 Mb). Second, we assess the performance of four contemporary genomic methods for inferring population size history (PSMC, MSMC, SMC++, Stairway Plot), using the two devil genome assemblies as well as simulated, artificially fragmented genomes that approximate the hypothesized demographic history of Tasmanian devils. We demonstrate that each method is robust to assembly quality, producing similar estimates of Ne when simulated genomes were fragmented into up to 5,000 scaffolds. Overall, methods reliant on the SMC are most reliable between similar to 300 generations before present (gbp) and 100 kgbp, whereas methods exclusively reliant on the site-frequency spectrum are most reliable between the present and 30 gbp. Our results suggest that when used in concert, genomic methods for reconstructing species’ effective population size histories 1) can be applied to nonmodel organisms without highly contiguous reference genomes, and 2) are capable of detecting independently documented effects of historical geological events. | NA | no | No good fitness measure and measure host GD | NA | no | no |
| 5453 | 2020 | 44113 | Paul, M; Bhadra, A | 2018 | The great Indian joint families of free-ranging dogs | Cooperative breeding is an excellent example of cooperation in social groups. Domestic dogs have evolved from cooperatively hunting and breeding ancestors but have adapted to a facultatively social scavenging lifestyle on streets, and solitary living in human homes. Pets typically breed and reproduce under human supervision, but free-ranging dogs can provide insights into the natural breeding ecology of dogs. We conducted a five year-long field based behavioural study on parental care of free-ranging dogs in India. 23 mother-litter units, belonging to 15 groups were observed, which revealed the presence of widespread allo-parenting by both adult males and females. While all the females were known to be related to the pups receiving care, the relatedness with the males could not be determined. Hence, we coined the term putative father for caregiving males. Allomothers provided significantly less care than the mothers, but the putative fathers showed comparable levels of care with the mothers. Mothers invested more effort in nursing and allogrooming, while the putative fathers played and protected more. Our observations provide support for both the benefit-of-philopatry and assured fitness returns hypotheses. Free-ranging dogs are not cooperative breeders like wolves but are rather communal breeders; their breeding biology bearing interesting similarities with the human joint family system. This breeding strategy is likely to have played an important role in increasing pup survival in a stochastic environment and helping to adapt to living among humans during the domestication of dogs. | NA | no | No good fitness measure and measure host GD | NA | no | no |
| 5454 | 2020 | 44043 | Pawluk, RJ; de Leaniz, CG; Cable, J; Tiddeman, B; Consuegra, S | 2019 | Colour plasticity in response to social context and parasitic infection in a self-fertilizing fish | Many animal species rely on changes in body coloration to signal social dominance, mating readiness and health status to conspecifics, which can in turn influence reproductive success, social dynamics and pathogen avoidance in natural populations. Such colour changes are thought to be controlled by genetic and environmental conditions, but their relative importance is difficult to measure in natural populations, where individual genetic variability complicates data interpretation. Here, we studied shifts in melanin-related body coloration in response to social context and parasitic infection in two naturally inbred lines of a self-fertilizing fish to disentangle the relative roles of genetic background and individual variation. We found that social context and parasitic infection had a significant effect on body coloration that varied between genetic lines, suggesting the existence of genotype by environment interactions. In addition, individual variation was also important for some of the colour attributes. We suggest that the genetic background drives colour plasticity and that this can maintain phenotypic variation in inbred lines, an adaptive mechanism that may be particularly important when genetic diversity is low. | NA | no | No good fitness measure and measure host GD | NA | no | no |
| 5455 | 2020 | 44112 | Payne, AN; Rangel, J | 2018 | The effect of queen insemination volume on the growth of newly established honey bee (Apis mellifera) colonies | The number of female progeny that a honey bee (Apis mellifera) queen produces in her lifetime is directly dependent on the amount of semen she collects upon mating (i.e., insemination volume) and the number of viable sperm cells contained within the semen (i.e., sperm viability). Queen insemination volume has been shown to alter queen mandibular pheromone profiles, as well as worker behavior and physiology at the individual level. In order to determine if queen insemination volume has any colony-level effects, we compared the growth of newly established colonies headed by queens instrumentally inseminated with either a low volume (1.5 mu L) or a high volume (9.0 mu L) of pooled semen from May to October in 2013 and 2015. We did not find a significant effect of queen insemination volume on the production of worker comb, drone comb, stored food, worker population, or seasonal queen or colony survivorship. Therefore, we concluded that queen insemination volume does not seem to directly affect growth at the colony level, at least during a colony’s first year. | NA | no | no good gd measure host pop | NA | no | no |
| 5456 | 2020 | 44113 | Pearson, SK; Bull, CM; Gardner, MG | 2018 | Selection outweighs drift at a fine scale: Lack of MHC differentiation within a family living lizard across geographically close but disconnected rocky outcrops | The highly polymorphic genes of the major histocompatibility complex (MHC) are involved in disease resistance, mate choice and kin recognition. Therefore, they are widely used markers for investigating adaptive variation. Although selection is the key driver, gene flow and genetic drift also influence adaptive genetic variation, sometimes in opposing ways and with consequences for adaptive potential. To further understand the processes that generate MHC variation, it is helpful to compare variation at the MHC with that at neutral genetic loci. Differences in MHC and neutral genetic variation are useful for inferring the relative influence of selection, gene flow and drift on MHC variation. To date, such investigations have usually been undertaken at a broad spatial scale. Yet, evolutionary and ecological processes can occur at a fine spatial scale, particularly in small or fragmented populations. We investigated spatial patterns of MHC variation among three geographically close, naturally discrete, sampling sites of Egernia stokesii, an Australian lizard. The MHC of E. stokesii has recently been characterized, and there is evidence for historical selection on the MHC. We found E. stokesii MHC weakly differentiated among sites compared to microsatellites, suggesting selection, acting similarly at each site, has outweighed any effects of low gene flow or of genetic drift on E. stokesii MHC variation. Our findings demonstrate the strength of selection in shaping patterns of MHC variation or consistency at a fine spatial scale. | NA | no | mhc diversity | NA | no | no |
| 5457 | 2020 | 44042 | Pegot-Espagnet, P; Guillaume, O; Desprez, B; Devaux, B; Devaux, P; Henry, K; Henry, N; Willems, G; Goudemand, E; Mangin, B | 2019 | Discovery of interesting new polymorphisms in a sugar beet (elite x exotic) progeny by comparison with an elite panel | Selection of stable varieties producing the highest amount of extractable sugar per hectare (ha), resistant to diseases, and respecting environmental criteria is undoubtedly the main target for sugar beet breeding. As sodium, potassium, and alpha-amino nitrogen in sugar beets are the impurities that have the biggest negative impact on white sugar extraction, it is interesting to reduce their concentration in further varieties. However, domestication history and strong selection pressures have affected the genetic diversity needed to achieve this goal. In this study, quantitative trait locus (QTL) detection was performed on two populations, an (elite x exotic) sugar beet progeny and an elite panel, to find potentially new interesting regions brought by the exotic accession. The three traits linked with impurities content were studied. Some QTLs were detected in both populations, the majority in the elite panel because of most statistical power. Some of the QTLs were colocated and had favorable effect in the progeny since the exotic allele was linked with a decrease in the impurity content. A few number of favorable QTLs were detected in the progeny, only. Consequently, introgressing exotic genetic material into sugar beet breeding programs can allow the incorporation of new interesting alleles. | NA | no | No good fitness measure and measure host GD | NA | no | no |
| 5458 | 2020 | 44113 | Peischl, S; Dupanloup, I; Foucal, A; Jomphe, M; Bruat, V; Grenier, JC; Gouy, A; Gilbert, KJ; Gbeha, E; Bosshard, L; Hip-Ki, E; Agbessi, M; Hodgkinson, A; Vezina, H; Awadalla, P; Excoffier, L | 2018 | Relaxed Selection During a Recent Human Expansion | Humans have colonized the planet through a series of range expansions, which deeply impacted genetic diversity in newly settled areas and potentially increased the frequency of deleterious mutations on expanding wave fronts. To test this prediction, we studied the genomic diversity of French Canadians who colonized Quebec in the 17th century. We used historical information and records from approximate to 4000 ascending genealogies to select individuals whose ancestors lived mostly on the colonizing wave front and individuals whose ancestors remained in the core of the settlement. Comparison of exomic diversity reveals that: (i) both new and low-frequency variants are significantly more deleterious in front than in core individuals, (ii) equally deleterious mutations are at higher frequencies in front individuals, and (iii) front individuals are two times more likely to be homozygous for rare very deleterious mutations present in Europeans. These differences have emerged in the past six to nine generations and cannot be explained by differential inbreeding, but are consistent with relaxed selection mainly due to higher rates of genetic drift on the wave front. Demographic inference and modeling of the evolution of rare variants suggest lower effective size on the front, and lead to an estimation of selection coefficients that increase with conservation scores. Even though range expansions have had a relatively limited impact on the overall fitness of French Canadians, they could explain the higher prevalence of recessive genetic diseases in recently settled regions of Quebec. | NA | no | No good fitness measure and measure host GD | NA | no | no |
| 5459 | 2020 | 44040 | Pelerito, A; Nunes, A; Nuncio, MS; Gomes, JP | 2020 | Genome-scale approach to study the genetic relatedness among Brucella melitensis strains | Brucellosis is an important zoonotic disease that affects both humans and animals. To date, laboratory surveillance is still essentially based on the traditional MLVA-16 methodology and the associated epidemiological information is frequently scarce. Our goal was to contribute to the improvement of Brucella spp. surveillance through the implementation of a whole genome sequencing (WGS) approach. We created a curated ready-to-use species-specific wgMLST scheme enrolling a panel of 2656 targets (http://doi.org/10.5281/zenodo. 3575026) and used this schema to perform a retrospective analysis of the genetic relatedness among B. melitensis strains causing human infection in Portugal (a country where brucellosis is an endemic disease) from 2010 to 2018. The strains showed a phylogenetic clustering within genotype II (25 out of 36) and IV (4 out of 36), and shared clades with strains isolated from countries with which Portugal has intense food trading, tourism and similar eating habits, such as Spain, Italy and Greece. In addition, our results point to the identification of strong associations between B. melitensis strains, likely underlying missed outbreaks as 22 out of the 36 strains showed genetic linkage with others. In fact, the applied gene-by-gene approach grouped these strains into six genetic clusters each one containing putative epidemiological links. Nevertheless, more studies will be needed in order to define the appropriate range of cut-offs (probable non-static cut-offs) that best illustrate the association between genetic linkage and epidemiological information and may serve as alerts for the health authorities. The release of this freely available and scalable schema contributes to the required technological transition for laboratorial surveillance of brucellosis and will facilitate the assessment of ongoing and future outbreaks in order to prevent the transmission spread. | NA | no | No good fitness measure and measure host GD | NA | no | no |
| 5460 | 2020 | 44113 | Pemberton, TJ; Szpiech, ZA | 2018 | Relationship between Deleterious Variation, Genomic Autozygosity, and Disease Risk: Insights from The 1000 Genomes Project | Genomic regions of autozygosity (ROAs) represent segments of individual genomes that are homozygous for haplotypes inherited identical-by-descent (IBD) from a common ancestor. ROAs are nonuniformly distributed across the genome, and increased ROA levels are a reported risk factor for numerous complex diseases. Previously, we hypothesized that long ROAs are enriched for deleterious homozygotes as a result of young haplotypes with recent deleterious mutations-relatively untouched by purifying selection-being paired IBD as a consequence of recent parental relatedness, a pattern supported by ROA and whole-exome sequence data on 27 individuals. Here, we significantly bolster support for our hypothesis and expand upon our original analyses using ROA and whole-genome sequence data on 2,436 individuals from The 1000 Genomes Project. Considering CADD deleteriousness scores, we reaffirm our previous observation that long ROAs are enriched for damaging homozygotes worldwide. We show that strongly damaging homozygotes experience greater enrichment than weaker damaging homozygotes, while overall enrichment varies appreciably among populations. Mendelian disease genes and those encoding FDA-approved drug targets have significantly increased rates of gain in damaging homozygotes with increasing ROA coverage relative to all other genes. In genes implicated in eight complex phenotypes for which ROA levels have been identified as a risk factor, rates of gain in damaging homozygotes vary across phenotypes and populations but frequently differ significantly from non-disease genes. These findings highlight the potential confounding effects of population background in the assessment of associations between ROA levels and complex disease risk, which might underlie reported inconsistencies in ROA-phenotype associations. | NA | no | No good fitness measure | NA | no | no |
| 5461 | 2020 | 44112 | Penczykowski, RM; Parratt, SR; Barres, B; Sallinen, SK; Laine, AL | 2018 | Manipulating host resistance structure reveals impact of pathogen dispersal and environmental heterogeneity on epidemics | Understanding how variation in hosts, parasites, and the environment shapes patterns of disease is key to predicting ecological and evolutionary outcomes of epidemics. Yet in spatially structured populations, variation in host resistance may be spatially confounded with variation in parasite dispersal and environmental factors that affect disease processes. To tease apart these disease drivers, we paired surveys of natural epidemics with experiments manipulating spatial variation in host susceptibility to infection. We mapped epidemics of the wind-dispersed powdery mildew pathogen Podosphaera plantaginis in five populations of its plant host, Plantago lanceolata. At 15 replicate sites within each population, we deployed groups of healthy potted ‘sentinel’ plants from five allopatric host lines. By tracking which sentinels became infected in the field and measuring pathogen connectivity and microclimate at those sites, we could test how variation in these factors affected disease when spatial variation in host resistance and soil conditions was minimized. We found that the prevalence and severity of sentinel infection varied over small spatial scales in the field populations, largely due to heterogeneity in pathogen prevalence on wild plants and unmeasured environmental factors. Microclimate was critical for disease spread only at the onset of epidemics, where humidity increased infection risk. Sentinels were more likely to become infected than initially healthy wild plants at a given field site. However, in a follow-up laboratory inoculation study we detected no significant differences between wild and sentinel plant lines in their qualitative susceptibility to pathogen isolates from the field populations, suggesting that primarily non-genetic differences between sentinel and wild hosts drove their differential infection rates in the field. Our study leverages a multi-faceted experimental approach to disentangle important biotic and abiotic drivers of disease patterns within wild populations. | NA | no | no good gd measure host pop | NA | no | no |
| 5462 | 2020 | 44112 | Peng, Y; Tang, SM; Wang, D; Zhong, HZ; Jia, HJ; Cai, XH; Zhang, ZX; Xiao, MF; Yang, HM; Wang, J; Kristiansen, K; Xu, X; Li, JH | 2018 | MetaPGN: a pipeline for construction and graphical visualization of annotated pangenome networks | Pangenome analyses facilitate the interpretation of genetic diversity and evolutionary history of a taxon. However, there is an urgent and unmet need to develop new tools for advanced pangenome construction and visualization, especially for metagenomic data. Here, we present an integrated pipeline, named MetaPGN, for construction and graphical visualization of pangenome networks from either microbial genomes or metagenomes. Given either isolated genomes or metagenomic assemblies coupled with a reference genome of the targeted taxon, MetaPGN generates a pangenome in a topological network, consisting of genes (nodes) and gene-gene genomic adjacencies (edges) of which biological information can be easily updated and retrieved. MetaPGN also includes a self-developed Cytoscape plugin for layout of and interaction with the resulting pangenome network, providing an intuitive and interactive interface for full exploration of genetic diversity. We demonstrate the utility of MetaPGN by constructing Escherichia coli pangenome networks from five E. coli pathogenic strains and 760 human gut microbiomes, revealing extensive genetic diversity of E. coli within both isolates and gut microbial populations. With the ability to extract and visualize gene contents and gene-gene physical adjacencies of a specific taxon from large-scale metagenomic data, MetaPGN provides advantages in expanding pangenome analysis to uncultured microbial taxa. | NA | no | No good fitness measure and measure host GD | NA | no | no |
| 5463 | 2020 | 44041 | Pennington, PM; Marshall, KL; Capiro, DM; Howard, L; Durrant, BS | 2020 | Pregnancies following long luteal phases in southern white rhinoceros (Ceratotherium simum simum) | All extant species in the Rhinocerotidae family are experiencing escalating threats in the wild, making self-sustaining captive populations essential genetic reservoirs for species survival. Assisted reproductive technologies (ARTs) will become increasingly important for achieving and maintaining ex situ population sustainability and genetic diversity. Previous reports have shown that a large proportion of captive southern white rhinoceros (SWR) females are irregularly cyclic or acyclic, and that cycling females display two different estrous cycle lengths of approximately 30 or 70 days. It has been suggested that the longer estrous cycle length is infertile or subfertile, as no term pregnancies have been observed following long cycles. Here we report the achievement of two pregnancies following long luteal phases, using ovulation induction and artificial insemination with either fresh or frozen-thawed semen. One female SWR conceived on the first insemination attempt and gave birth to a live offspring. A second female conceived twice in consecutive long cycles although the first embryo was resorbed by 33 days post-insemination. A pregnancy from this female’s second insemination is ongoing with expected parturition in November 2019. Whether prolonged estrous cycles in SWR are subfertile or infertile in natural breeding situations remains unclear. However, our findings demonstrate that the application of ARTs following prolonged cycles can result the successful establishment of pregnancies in SWR. Therefore, with ARTs, female SWR otherwise considered nonreproductive due to long estrous cycles may still have the potential for representation and contribution to the ex situ population. | NA | no | No good fitness measure and measure host GD | NA | no | no |
| 5464 | 2020 | 44112 | Pereira, FL; Goncalves, LA; Tavares, GC; Soares, SD; Dorella, FA; de Carvalho, AF; Leibowitz, MP; Leal, CAG; Azevedo, VAD; Figueiredo, HCP | 2018 | Complete genome sequencing of sixteen Francisella noatunensis subsp. orientalis isolates: A genomic approach for molecular characterization and spread dynamics of this clonal population | Francisella noatunensis subsp. orientalis (FNO) is an important emerging pathogen associated with disease outbreaks in farm-raised Nile tilapia. FNO genetic diversity using PCR-based typing, no intra-species discrimination was achieved among isolates/strains from different countries, thus demonstrating a clonal behaviour pattern. In this study, we aimed to evaluate the population structure of FNO isolates by comparing whole-genome sequencing data. The analysis of recombination showed that Brazilian isolates group formed a clonal population; whereas other lineages are also supported by this analysis for isolates from foreign countries. The whole-genome multilocus sequence typing (wgMLST) analysis showed varying numbers of dissimilar alleles, suggesting that the Brazilian clonal population are in expansion. Each Brazilian isolate could be identified as a single node by high-resolution gene-by-gene approach, presenting slight genetic differences associated to mutational events. The common ancestry node suggests a single entry into the country before 2012, and the rapid dissemination of this infectious agent may be linked to market sales of infected fingerlings. | NA | no | No good fitness measure | NA | no | no |
| 5465 | 2020 | 44112 | Perez-Enriquez, R; Robledo, D; Houston, RD; Llera-Herrera, R | 2018 | SNP markers for the genetic characterization of Mexican shrimp broodstocks | Selective breeding of shrimp has major potential to enhance production traits, including growth and disease resistance. Genetic characterization of broodstock populations is a key element of breeding programs, as it enables decisions on inbreeding restrictions, family structure, and the potential use of genomic selection. Single Nucleotide Polymorphisms (SNPs) are suitable genetic markers for this purpose. A set of SNPs was developed to characterize commercial breeding stocks in Mexico. Individuals from local and imported lines were selected for sequencing using the nextRAD technique, resulting in the identification of 2619 SNPs. Genetic structure analysis showed three to five genetic groups of Ecuadorian and Mexican origins. A subset of 1231 SNPs has potential for stock identification and management. Further, three SNPs were identified as candidate sex-linked markers. The role of SNPs possibly associated with genes related to traits of importance to shrimp farming, such as growth and immune response, should be further investigated. | NA | no | No good fitness measure and measure host GD | NA | no | no |
| 5466 | 2020 | 44111 | Perez-Espona, S; Goodall-Copestake, WP; Savirina, A; Bobovikova, J; Molina-Rubio, C; Perez-Barberia, FJ | 2019 | First assessment of MHC diversity in wild Scottish red deer populations | Control and mitigation of disease in wild ungulate populations are one of the major challenges in wildlife management. Despite the importance of the major histocompatibility complex (MHC) genes for immune response, assessment of diversity on these genes is still rare for European deer populations. Here, we conducted the first assessment of variation at the second exon of the MHC DRB in wild populations of Scottish highland red deer, the largest continuous population of red deer in Europe. Allelic diversity at these loci was high, with 25 alleles identified. Selection analyses indicated c. 22% of amino acids encoded under episodic positive selection. Patterns of MHC allelic distribution were not congruent with neutral population genetic structure (estimated with 16 nuclear microsatellite markers) in the study area, the latter showing a marked differentiation between populations located at either side of the Great Glen. This study represents a first step towards building an immunogenetic map of red deer populations across Scotland to aid future management strategies for this ecologically and economically important species. | NA | no | mhC diversity | NA | no | no |
| 5467 | 2020 | 44111 | Perez-Irineo, G; Barrera, CB; Santos-Moreno, A | 2019 | Densidad, environmental suitability and ecological niche in four American feline species (Carnivora: Felidae) | Several wild species of the family Felidae are at risk, and population size is one of the most important ecological parameters to determine conservation actions. Population size is influenced by different biotic and abiotic factors that satisfy the requirements of the species to survive and reproduce, allowing the permanence of the species through time. Because population size is not homogeneous throughout the distribution, there are several hypotheses to explain their variations. One proposal considers that more abundant populations are located in regions with environmentally suitability, and population size decreases towards the less favorable ones. Another hypothesis considers that the population size is related to the internal structure of the ecological niche, where the largest populations are located in the centroid of the niche, and the size declines as the distance to this centroid increase. The objective of this study was to evaluate the relationship between population abundance, environmental suitability and ecological niche in four wild felids species of America. The density of Lynx rufus (bobcat) was correlated positively with the environmental suitability index, the highest density of Panthera onca (jaguar) was located in regions near the centroid of the niche, while the population densities of Leopardus pardalis (ocelot) and Puma concolor (cougar) did not conform to the predictions of either of the two hypotheses. The relationship between feline density and environmental characteristics was species-specific, with no general pattern. | NA | no | No good fitness measure | NA | no | no |
| 5468 | 2020 | 44043 | Perez-Valencia, LI; Michel, AP; Moya-Raygoza, G; Rodriguez, A | 2019 | Genetic variation and structure of Diaphorina citri (Hemiptera: Liviidae) in populations from Mexico | The Asian citrus psyllid, Diaphorina citri Kuwayama, is native to Asia but has recently invaded North America. Asian citrus psyllid is a significant pest of citrus crops by its direct feeding but, more importantly, as the vector of the bacterium Candidatus Liberibacter asiaticus’, which causes Huanglongbing disease. Asian citrus psyllid was first found in Mexico in 2001 and 2002 and, since then, has spread quickly across the country, suggesting rapid adaptation to new environments. Yet, we lack information on the genetic variation and structure that could facilitate or inhibit adaptation. Using six microsatellite markers, we analyzed genetic variation and structure among six localities in Mexico: three in western states near the Pacific coast and two in the Yucatan Peninsula near the Gulf of Mexico. We found low genetic diversity (no more than three alleles per locus) and intermediate differentiation between all populations. Asian citrus psyllid populations clustered into two genetic groups, but, surprisingly, these clusters were present in western populations. The first group included El Arenal, and the second group included Autlan de Navarro, Colima, and Tecoman. Interestingly, both of the Yucatan populations shared variation from the two clusters, suggesting admixture. We infer that reproductive isolation, barriers to gene flow, local selection, and the possibility of multiple invasions have influenced the current genetic structure of Asian citrus psyllid in Mexico. | NA | no | No good fitness measure | NA | no | no |
| 5469 | 2020 | 44113 | Perry, KD; Baker, GJ; Powis, KJ; Kent, JK; Ward, CM; Baxter, SW | 2018 | Cryptic Plutella species show deep divergence despite the capacity to hybridize | Background: Understanding genomic and phenotypic diversity among cryptic pest taxa has important implications for the management of pests and diseases. The diamondback moth, Plutella xylostella L., has been intensively studied due to its ability to evolve insecticide resistance and status as the world’s most destructive pest of brassicaceous crops. The surprise discovery of a cryptic species endemic to Australia, Plutella australiana Landry & Hebert, raised questions regarding the distribution, ecological traits and pest status of the two species, the capacity for gene flow and whether specific management was required. Here, we collected Plutella from wild and cultivated brassicaceous plants from 75 locations throughout Australia and screened 1447 individuals to identify mtDNA lineages and Wolbachia infections. We genotyped genome-wide SNP markers using RADseq in coexisting populations of each species. In addition, we assessed reproductive compatibility in crossing experiments and insecticide susceptibility phenotypes using bioassays. Results: The two Plutella species coexisted on wild brassicas and canola crops, but only 10% of Plutella individuals were P. australiana. This species was not found on commercial Brassica vegetable crops, which are routinely sprayed with insecticides. Bioassays found that P. australiana was 19-306 fold more susceptible to four commonly-used insecticides than P. xylostella. Laboratory crosses revealed that reproductive isolation was incomplete but directionally asymmetric between the species. However, genome-wide nuclear SNPs revealed striking differences in genetic diversity and strong population structure between coexisting wild populations of each species. Nuclear diversity was 1.5-fold higher in P. australiana, yet both species showed limited variation in mtDNA. Infection with a single Wolbachia subgroup B strain was fixed in P. australiana, suggesting that a selective sweep contributed to low mtDNA diversity, while a subgroup A strain infected just 1.5% of P. xylostella. Conclusions: Despite sympatric distributions and the capacity to hybridize, strong genomic and phenotypic divergence exists between these Plutella species that is consistent with contrasting colonization histories and reproductive isolation after secondary contact. Although P. australiana is a potential pest of brassicaceous crops, it is of secondary importance to P. xylostella. | NA | no | No good fitness measure | NA | no | no |
| 5470 | 2020 | 44112 | Pestana, JR; de Morais, TP; Nogueira, APO; Juliatti, FC | 2018 | REACTION OF BRAZILIAN COTTON GENOTYPES TO WHITE MOLD DEPENDS ON PATHOGEN AGGRESSIVENESS AND INCUBATION CONDITIONS | The expansion of cotton crop into irrigated and high lands of Brazilian Cerrado, despite the possibility of increasing fiber yield, led to the occurrence of diseases previously considered secondary, such as white mold [Sclerotinia sclerotiorum (Lib.) de Bary]. Host genetic resistance is of extreme importance in integrated strategies to manage this disease. Resistance of Brazilian cotton genotypes, challenged with different strains of S. sclerotiorum, under two incubation conditions for disease progress was evaluated. In addition, possible correlation between oxalic acid and straw test methods to rank the genotypes was evaluated. Artificial inoculation was done when cotton plants reached the V-2 phenological stage with fungi isolated from naturally infected soybean (ScS) or cotton (ScC) commercial crops. Control plants were inoculated with culture medium. After inoculation, plants were kept for one week either in a growth chamber or in greenhouse and evaluated for disease symptoms and severity. The oxalic acid test consisted of stem submersion of rootless cotton plants in a 2-cm layer of 20 or 40 mM solutions for 20, 44 or 68 h. A wilting scale was used to distinguish genotype’s sensibility to the acid. The data were submitted to individual, joint, and multivariate analysis, grouping cotton genotypes by the Scott-Knott’s test (p < 0.05), the hierarchical UPGMA and the non-hierarchical Tocher methods. Difference in aggressiveness between strains was identified, in which ScC led to greater disease severity. This result suggests a possible physiological specialization of S. sclerotiorum to different hosts. It was observed that the growth chamber environment provided more adequate conditions for S. sclerotiorum infection, thus allowing better selection of resistant cotton genotypes. UPGMA and Tocher grouping methods further confirmed that the evaluated genotypes differ from each other in resistance to white mold. No correlation between oxalic acid and straw test methods was observed. | NA | no | dometicated host | NA | no | no |
| 5471 | 2020 | 44111 | Peters, MJ; Suwannapong, G; Pelin, A; Corradi, N | 2019 | Genetic and Genome Analyses Reveal Genetically Distinct Populations of the Bee Pathogen Nosema ceranae from Thailand | The recent global decline in Western honeybee (Apis mellifera) populations is of great concern for pollination and honey production worldwide. Declining honeybee populations are frequently infected by the microsporidian pathogen Nosema ceranae. This species was originally described in the Asiatic honeybee (Apis cerana), and its identification in global A. mellifera hives could result from a recent host transfer. Recent genome studies have found that global populations of this parasite are polyploid and that humans may have fueled their global expansion. To better understand N. ceranae biology, we investigated its genetic diversity within part of their native range (Thailand) and among different hosts (A. mellifera, A. cerana) using both PCR and genome-based methods. We find that Thai N. ceranae populations share many SNPs with other global populations and appear to be clonal. However, in stark contrast with previous studies, we found that these populations also carry many SNPs not found elsewhere, indicating that these populations have evolved in their current geographic location for some time. Our genome analyses also indicate the potential presence of diploidy within Thai populations of N. ceranae. | NA | no | No good fitness measure | NA | no | no |
| 5472 | 2020 | 44043 | Petkar, A; Harris-Shultz, K; Wang, HL; Brewer, MT; Sumabat, L; Ji, PS | 2019 | Genetic and phenotypic diversity of Fusarium oxysporum f. sp. niveum populations from watermelon in the southeastern United States | Fusarium wilt of watermelon, caused by Fusarium oxysporum f. sp. niveum (FON), occurs worldwide and is responsible for substantial yield losses in watermelon-producing areas of the southeastern United States. Management of this disease largely relies on the use of integrated pest management (i.e., fungicides, resistant cultivars, crop rotation, etc.). Knowledge about race structure and genetic diversity of FON in the southeastern US is limited. To determine genetic diversity of the pathogen, FON isolates were collected from symptomatic watermelon plants in commercial fields in Georgia and Florida, USA, and identified based on morphological characteristics and PCR analysis using FON-specific primers. Discriminant analysis of principal components (DAPC) of 99 isolates genotyped with 15 simple sequence repeat (SSR) markers grouped the isolates in eight distinct clusters with two prominent clusters (clusters 1 and 8). Cluster 1 consisted of a total of 14 isolates, out of which 85.7% of the isolates were collected in Florida. However, most of the isolates (92.4%) in cluster 8 were collected in Georgia. Both DAPC and pairwise population differentiation analysis (Phi(PT)) revealed that the genetic groups were closely associated with geographical locations of pathogen collection. Three races of FON (races 0, 2 and 3) were identified in the phenotypic analysis; with race 3 identified for the first time in Georgia. Overall, 5.1%, 38.9% and 55.9% of the isolates were identified as race 0, race 2 and race 3, respectively. The majority of the isolates in cluster 1 and cluster 8 belonged to either race 2 (35.6%) or race 3 (45.8%). Additionally, no relationship between genetic cluster assignment and races of the isolates was observed. The information obtained on genotypic and phenotypic diversity of FON in the southeastern US will help in development of effective disease management programs to combat Fusarium wilt. | NA | no | gd of pathogen | NA | no | no |
| 5473 | 2020 | 44113 | Petrusic, M; Vidakovic, DO; Lazic, S; Radnovic, D; Knezevic, P | 2018 | Prevalence and genetic variability of Plesiomonas shigelloides in temperate climate surface waters of the Pannonian Plain | Plesiomonas shigelloides, a Gram-negative bacterium and the causative agent of intestinal diseases and extraintestinal infections in humans and animals, is most frequently found in aquatic environments in tropical or subtropical areas. The present study was designed to establish the prevalence and genetic variability of P. shigelloides in surface waters (lakes, rivers, ponds, inlets and canals) located in a temperate climate zone, namely the Pannonian Plain of the northern part of Serbia and southern part of Hungary. The strains were isolated directly by plating samples on inositol-brilliant green-bile agar with neutral red or phenol red as indicators. Our results indicate that phenol red effectively facilitates differentiation of P. shigelloides from other bacteria. A number of samples were enriched using alkaline peptone water broth, peptone inositolbile broth and tetrathionate broth. The recovery of the isolates was more successful with the first medium. Out of a total of 51 water samples collected from 28 different locations, 22 samples (43.1%) were found positive for P. shigelloides. Among the 37 isolated strains, 34 were from lakes (Satrinci, Ludas, Panonija, Krivaja, Pecs, Kapetanski rit, Pavlovci, Kovacsszenaja, Dobrodol, Vranjas, Borkovac, Hermann Otto, Sot, Selevrenac, Zobnatica, Palic, Orfui, Jarkovci, Conoplja) and 3 were from rivers (Danube, Sava). The strains were identified by phenotypic characteristic or by the VITEK2 system and confirmed by PCR using 23S rRNA species-specific oligos. The strains showed a high genetic variability, displaying a variety of RAPD profiles. Our results reveal for the first time a high prevalence of genetically diverse P. shigelloides populations in surface waters located in the temperate climate of central and southeastern Europe. | NA | no | No good fitness measure and measure host GD | NA | no | no |
| 5474 | 2020 | 44111 | Phelan, JE; Lim, DR; Mitarai, S; de Sessions, PF; Tujan, MAA; Reyes, LT; Medado, IAP; Palparan, AG; Naim, ANM; Jie, S; Segubre-Mercado, E; Simoes, B; Campino, S; Hafalla, JC; Murase, Y; Morishige, Y; Hibberd, ML; Kato, S; Ama, MCG; Clark, TG | 2019 | Mycobacterium tuberculosis whole genome sequencing provides insights into the Manila strain and drug-resistance mutations in the Philippines | The Philippines has a high incidence of tuberculosis disease (TB), with an increasing prevalence of multidrug-resistant Mycobacterium tuberculosis (MDR-TB) strains making its control difficult. Although the M. tuberculosis Manila ancient lineage 1 strain-type is thought to be prevalent in the country, with evidence of export to others, little is known about the genetic diversity of circulating strains. By whole genome sequencing (WGS) 178 isolates from the Philippines National Drug Resistance Survey, we found the majority (143/178; 80.3%) belonged to the lineage 1 Manila clade, with the minority belonging to lineages 4 (European-American; n = 33) and 2 (East Asian; n = 2). A high proportion were found to be multidrug-resistant (34/178; 19.1%), established through highly concordant laboratory drug susceptibility testing and in silico prediction methods. Some MDR-TB isolates had near identical genomic variation, providing potential evidence of transmission. By placing the Philippine isolates within a phylogeny of global M. tuberculosis (n > 17,000), we established that they are genetically similar to those observed outside the country, including a clade of Manila-like strain-types in Thailand. An analysis of the phylogeny revealed a set of similar to 200 SNPs that are specific for the Manila strain-type, and a subset can be used within a molecular barcode. Sixty-eight mutations known to be associated with 10 anti-TB drug resistance were identified in the Philippine strains, and all have been observed in other populations. Whilst nine putative streptomycin resistance conferring markers in gid (8) and rrs (1) genes appear to be novel and with functional consequences. Overall, this study provides an important baseline characterisation of M. tuberculosis genetic diversity for the Philippines, and will fill a gap in global datasets and aid the development of a nation-wide database for epidemiological studies and clinical decision making. Further, by establishing a molecular barcode for detecting Manila strains it will assist with the design of diagnostic tools for disease control activities. | NA | no | No good fitness measure | NA | no | no |
| 5475 | 2020 | 44113 | Phillips, S | 2018 | Differing mortality rates in two concurrently radio-tracked populations of koala (Phascolarctos cinereus) | Radio-tracking studies enable insights into factors that contribute to koala mortality. Two radio-tracking studies investigating the impacts of disturbance events on koalas were undertaken in different areas over the same period. Both studies employed similar techniques for koala capture, processing and monitoring. In one study, none of nine koalas died during a 5-month monitoring program following their translocation into a new habitat area, while in the second study 6 of 11 koalas died over the same period during an in situ impact-monitoring study. The two populations differed morphologically and genetically: that with the higher mortality rate notable for a smaller head and neck circumference and lower genetic diversity. Differing outcomes from the two studies lend support to a hypothesis that inbreeding and the loss of genetic information may predispose some individuals and/or populations of koalas to an elevated stress response and/or increased susceptibility to disease, the expression of which may become exacerbated in the presence of ongoing disturbance or novel stressors that can include research activities. If this is the case, the endocrinology and genetic structure of free-ranging koala populations needs to be afforded greater consideration in terms of predicting a given population’s immunological response to potential isolation and/or disturbance events. | NA | no | no good gd measure host pop | NA | no | no |
| 5476 | 2020 | 44113 | Piaskowski, J; Hardner, C; Cai, LC; Zhao, YY; Iezzoni, A; Peace, C | 2018 | Genomic heritability estimates in sweet cherry reveal non-additive genetic variance is relevant for industry-prioritized traits | Background: Sweet cherry is consumed widely across the world and provides substantial economic benefits in regions where it is grown. While cherry breeding has been conducted in the Pacific Northwest for over half a century, little is known about the genetic architecture of important traits. We used a genome-enabled mixed model to predict the genetic performance of 505 individuals for 32 phenological, disease response and fruit quality traits evaluated in the RosBREED sweet cherry crop data set. Genome-wide predictions were estimated using a repeated measures model for phenotypic data across 3 years, incorporating additive, dominance and epistatic variance components. Genomic relationship matrices were constructed with high-density SNP data and were used to estimate relatedness and account for incomplete replication across years. Results: High broad-sense heritabilities of 0.83, 0.77, and 0.76 were observed for days to maturity, firmness, and fruit weight, respectively. Epistatic variance exceeded 40% of the total genetic variance for maturing timing, firmness and powdery mildew response. Dominance variance was the largest for fruit weight and fruit size at 34% and 27%, respectively. Omission of non-additive sources of genetic variance from the genetic model resulted in inflation of narrow-sense heritability but minimally influenced prediction accuracy of genetic values in validation. Predicted genetic rankings of individuals from single-year models were inconsistent across years, likely due to incomplete sampling of the population genetic variance. Conclusions: Predicted breeding values and genetic values revealed many high-performing individuals for use as parents and the most promising selections to advance for cultivar release consideration, respectively. This study highlights the importance of using the appropriate genetic model for calculating breeding values to avoid inflation of expected parental contribution to genetic gain. The genomic predictions obtained will enable breeders to efficiently leverage the genetic potential of North American sweet cherry germplasm by identifying high quality individuals more rapidly than with phenotypic data alone. | NA | no | No good fitness measure and measure host GD | NA | no | no |
| 5477 | 2020 | 44113 | Piccinali, RV; Gaunt, MW; Gurtler, RE | 2018 | A Microsatellite-Based Analysis of House Infestation With Triatoma Infestans (Hemiptera: Reduviidae) After Insecticide Spraying in the Argentine Chaco | Prevention of vector-borne transmission of Chagas disease mainly relies on residual insecticide spraying. Despite significant success at a regional scale, house infestation with Triatoma infestans (Klug) (Hemiptera: Reduviidae) still persists in the Gran Chaco ecoregion. One key aspect is the identification of the sources of reinfestant triatomines. After detecting fine-scale genetic structure in two rural villages of Pampa del Indio, Argentine Chaco, we tested hypotheses on the putative origins of the triatomines collected at 4, 8, and 12 mo after insecticide house spraying. We genotyped 10 microsatellite loci in 262 baseline and 83 postspraying triatomines from different houses. Genetic variability was similar between baseline and postspraying populations, but 13 low-frequency alleles were not detected at postspraying. F(ST)s were not significant between insects collected before and after insecticide spraying at the same house in all but one case, and they clustered together in a neighbor-joining tree. A clustering algorithm detected seven genetic groups, four of them mainly composed of baseline and postspraying insects from the same house. Assignment tests suggested multiple putative sources (including the house of collection) for most postspraying insects but excluded a house located more than 9 km from the study area. The origin of three triatomines was attributed to immigration from other unaccounted sources. Our study is compatible with the hypothesis that house reinfestations in the Argentine Chaco are mostly related to residual foci (i.e., survival of insects within the same community), in agreement with field observations, spatial analysis, and morphometric studies previously published. | NA | no | No good fitness measure and measure host GD | NA | no | no |
| 5478 | 2020 | 44111 | Pierce, AA; de Man, TJB | 2019 | Antibiotic resistant pathogen outbreak investigation: an interdisciplinary module to teach fundamentals of evolutionary biology | The evolution of resistance to antibiotics provides a timely and relevant topic for teaching undergraduate students evolutionary biology. Here, we present a module incorporating modified sequencing data from eight antibiotic resistant pathogen outbreaks in hospital settings with bioinformatics and phylogenetic analyses. This module uses whole genome sequencing data from hospital outbreaks investigated by the Centers for Disease Control and Prevention to provide examples of antibiotic resistance spread. Students work in groups to analyze outbreak data to identify the bacterial species and antibiotic resistance genes, to infer a phylogenetic tree examining relatedness among isolates, and to determine a possible source of the outbreak. Students then compile their results in individual reports and provide recommendations for preventing the further spread of antibiotic resistant organisms. In addition to providing genomic outbreak data, we include a teaching concepts guide discussing three integral components of the module: how evolutionary biology concepts of natural selection and competition impact antibiotic resistance; outbreak investigation information to aid in phylogenetic analysis and creation of recommendations; and instructions for the bioinformatics protocol. Completion of this module provides students an opportunity to think critically about the evolution of resistance, practice bioinformatics techniques, and relate evolutionary biology to current events. | NA | no | No good fitness measure and measure host GD | NA | no | no |
| 5479 | 2020 | 44113 | Pierce, MD; Dzama, K; Muchadeyi, FC | 2018 | Genetic Diversity of Seven Cattle Breeds Inferred Using Copy Number Variations | Copy number variations (CNVs) comprise deletions, duplications, and insertions found within the genome larger than 50 bp in size. CNVs are thought to be primary role-players in breed formation and adaptation. South Africa boasts a diverse ecology with harsh environmental conditions and a broad spectrum of parasites and diseases that pose challenges to livestock production. This has led to the development of composite cattle breeds which combine the hardiness of Sanga breeds and the production potential of the Taurine breeds. The prevalence of CNVs within these respective breeds of cattle and the prevalence of CNV regions (CNVRs) in their diversity, adaptation and production is however not understood. This study therefore aimed to ascertain the prevalence, diversity, and correlations of CNVRs within cattle breeds used in South Africa. Illumina Bovine SNP50 data and PennCNV were utilized to identify CNVRs within the genome of 287 animals from seven cattle breeds representing Sanga, Taurine, Composite, and cross breeds. Three hundred and fifty six CNVRs of between 36 kb to 4.1 Mb in size were identified. The null hypothesis that one CNVR loci is independent of another was tested using the GENEPOP software. One hunded and two and seven of the CNVRs in the Taurine and Sanga/Composite cattle breeds demonstrated a significant (p <= 0.05) association. PANTHER overrepresentation analyses of correlated CNVRs demonstrated significant enrichment of a number of biological processes, molecular functions, cellular components, and protein classes. CNVR genetic variation between and within breed group was measured using phi FT which allows intra-individual variation to be suppressed and hence proved suitable for measuring binary CNVR presence/absence data. Estimate Phi FT within and between breed variance was 2.722 and 0.518 respectively. Pairwise population PhiPT values corresponded with breed type, with Taurine Holstem and Angus breeds demonstrating no between breed CNVR variation. Phylogenetic trees were drawn. CNVRs primarily clustered animals of the same breed type together. This study successfully identified, characterized, and analyzed 356 CNVRs within seven cattle breeds. CNVR correlations were evident, with many more correlations being present among the exotic Taurine breeds. CNVR genetic diversity of Sanga, Taurine and Composite breeds was ascertained with breed types exposed to similar selection pressures demonstrating analogous incidences of CNVRs. | NA | no | dometicated host | NA | no | no |
| 5480 | 2020 | 44111 | Pilczynska, J; Cocito, S; Boavida, J; Serrao, EA; Assis, J; Fragkopoulou, E; Queiroga, H | 2019 | Genetic diversity increases with depth in red gorgonian populations of the Mediterranean Sea and the Atlantic Ocean | Background. In the ocean, the variability of environmental conditions found along depth gradients exposes populations to contrasting levels of perturbation, which can be reflected in the overall patterns of species genetic diversity. At shallow sites, resource availability may structure large, persistent and well-connected populations with higher levels of diversity. In contrast, the more extreme conditions, such as thermal stress during heat waves, can lead to population bottlenecks and genetic erosion, inverting the natural expectation. Here we examine how genetic diversity varies along depth for a long-lived, important ecosystem-structuring species, the red gorgonian, Paramuricea clavata. Methods. We used five polymorphic microsatellite markers to infer differences in genetic diversity and differentiation, and to detect bottleneck signs between shallow and deeper populations across the Atlantic Ocean and the Mediterranean Sea. We further explored the potential relationship between depth and environmental gradients (temperature, ocean currents, productivity and slope) on the observed patterns of diversity by means of generalized linear mixed models. Results. An overall pattern of higher genetic diversity was found in the deeper sites of the Atlantic Ocean and the Mediterranean Sea. This pattern was largely explained by bottom temperatures, with a linear pattern of decreasing genetic diversity with increasing thermal stress. Genetic differentiation patterns showed higher gene flow within sites (i.e., shallow vs. deeper populations) than between sites. Recent genetic bottlenecks were found in two populations of shallow depths. Discussion. Our results highlight the role of deep refugial populations safeguarding higher and unique genetic diversity for marine structuring species. Theoretical regression modelling demonstrated how thermal stress alone may reduce population sizes and diversity levels of shallow water populations. In fact, the examination of time series on a daily basis showed the upper water masses repeatedly reaching lethal temperatures for P. clavata. Differentiation patterns showed that the deep richer populations are isolated. Gene flow was also inferred across different depths; however, not in sufficient levels to offset the detrimental effects of surface environmental conditions on genetic diversity. The identification of deep isolated areas with high conservation value for the red gorgonian represents an important step in the face of ongoing and future climate changes. | NA | no | No good fitness measure | NA | no | no |
| 5481 | 2020 | 44112 | Pinacho-Pinacho, CD; Garcia-Varela, M; Sereno-Uribe, AL; de Leon, GPP | 2018 | A hyper-diverse genus of acanthocephalans revealed by tree-based and non-tree-based species delimitation methods: Ten cryptic species of Neoechinorhynchus in Middle American freshwater fishes | The genus Neoechinorhynchus represents a hyper-diverse group of acanthocephalans, parasites of fresh and brackish water fish and freshwater turtles, with approximately 116 species described worldwide. Forty-nine species have been recorded in the Americas, nine of them in Middle America. Even though species delimitation methods using DNA sequences have been rarely used for parasitic helminths, the genetic library for species of Neoechinorhynchus has grown in the past few years, enhancing the possibility of using these methods for inferring evolutionary relationships and for establishing more robust species boundaries. In this study, we used non-treebased and tree-based methods through a coalescent approach to explore the species limits of specimens of Neoechinorhynchus collected in 57 localities across Middle America. We sequenced a large number of individuals to build a comprehensive dataset for three genes: the mitochondrial cytochrome c oxidase subunit I (352 individuals), the internal transcribed spacers (330 individuals), and the D2 + D3 domains of the large subunit (278 individuals). Several species delimitation methods were implemented, i.e., Automatic Barcode Gap Discovery (ABGD), General Mixed Yule-Coalescent Model (GMYC), Bayesian species delimitation (BPP) and species tree (\*BEAST). Additionally, we conducted a detailed morphological study of the diagnostic traits associated with the proboscis of 184 males and 169 females. Overall, our analyses allowed us to validate nine nominal species of Neoechinorhynchus and to identify 10 additional genetic lineages herein regarded as candidate species. This unexpected genetic diversity and the lack of reliable morphological traits show that the genus Neoechinorhynchus includes a group of cryptic species, at least in Middle America. | NA | no | No good fitness measure and measure host GD | NA | no | no |
| 5482 | 2020 | 44112 | Pinheiro, A; de Sousa-Pereira, P; Strive, T; Knight, KL; Woof, JM; Esteves, PJ; Abrantes, J | 2018 | Identification of a new European rabbit IgA with a serine-rich hinge region | In mammals, the most striking IgA system belongs to Lagomorpha. Indeed, 14 IgA sub-classes have been identified in European rabbits, 11 of which are expressed. In contrast, most other mammals have only one IgA, or in the case of hominoids, two IgA subclasses. Characteristic features of the mammalian IgA subclasses are the length and amino acid sequence of their hinge regions, which are often rich in Pro, Ser and Thr residues and may also carry Cys residues. Here, we describe a new IgA that was expressed in New Zealand White domestic rabbits of IGHVa1 allotype. This IgA has an extended hinge region containing an intriguing stretch of nine consecutive Ser residues and no Pro or Thr residues, a motif exclusive to this new rabbit IgA. Considering the amino acid properties, this hinge motif may present some advantage over the common IgA hinge by affording novel functional capabilities. We also sequenced for the first time the IgA14 CH2 and CH3 domains and showed that IgA14 and IgA3 are expressed. | NA | no | No good fitness measure and measure host GD | NA | no | no |
| 5483 | 2020 | 44112 | Pinto, FAS; Bager, A; Clevenger, AP; Grilo, C | 2018 | Giant anteater (Myrmecophaga tridactyla) conservation in Brazil: Analysing the relative effects of fragmentation and mortality due to roads | Road networks ran have serious ecological consequences for many species, mainly through habitat fragmentation and mortality due to collisions with vehicles. One example of a species impacted by roads is the giant anteater (Myrmecophaga tridactyla), currently listed as Vulnerable by IUCN. Here we analysed the relative effect of fragmentation and mortality due to roads on giant anteater populations and show the critical areas for their persistence in Brazil. We estimated minimum patch size and maximum road density to evaluate the impact of the road network and observed road-kills on this species. We explored different scenarios by varying values of dispersal capacity to estimate the minimum patch size, and also of population densities to estimate maximum road density for giant anteater persistence. Our findings indicated that the minimum patch size can be from 498 to 247 km(2) and the maximum road density can vary between 0.21 and 0.55 km/km(2) in pessimist and optimistic scenarios, respectively. In Brazil, habitat fragmentation seemed to have a major impact over giant anteater populations. Habitat fragmentation due to roads seemed to have a more negative effect than mortality due to collisions with vehicles. Critical areas for the species persistence can represent 32% of its range in the optimistic scenario with 18% of suitable patches below the minimum size and 0.1% above the maximum road density. This study provides insights and implications for road networks on giant anteater populations in Brazil and guidance on road density and patch size thresholds for land managers and road agencies charged with planning ecologically sustainable roads in Brazil. | NA | no | No good fitness measure | NA | no | no |
| 5484 | 2020 | 44113 | Piotrowska, MJ; Riddell, C; Hoebe, PN; Ennos, RA | 2018 | Planting exotic relatives has increased the threat posed by Dothistroma septosporum to the Caledonian pine populations of Scotland | To manage emerging forest diseases and prevent their occurrence in the future, it is essential to determine the origin(s) of the pathogens involved and identify the management practices that have ultimately caused disease problems. One such practice is the widespread planting of exotic tree species within the range of related native taxa. This can lead to emerging forest disease both by facilitating introduction of exotic pathogens and by providing susceptible hosts on which epidemics of native pathogens can develop. We used microsatellite markers to determine the origins of the pathogen Dothistroma septosporum responsible for the current outbreak of Dothistroma needle blight (DNB) on native Caledonian Scots pine (Pinus sylvestris) populations in Scotland and evaluated the role played by widespread planting of two exotic pine species in the development of the disease outbreak. We distinguished three races of D. septosporum in Scotland, one of low genetic diversity associated with introduced lodgepole pine (Pinus contorta), one of high diversity probably derived from the DNB epidemic on introduced Corsican pine (Pinus nigra subsp. laricio) in England and a third of intermediate diversity apparently endemic on Caledonian Scots pine. These races differed for both growth rate and exudate production in culture. Planting of exotic pine stands in the UK appears to have facilitated the introduction of two exotic races of D. septosporum into Scotland which now pose a threat to native Caledonian pines both directly and through potential hybridization and introgression with the endemic race. Our results indicate that both removal of exotic species from the vicinity of Caledonian pine populations and restriction of movement of planting material are required to minimize the impact of the current DNB outbreak. They also demonstrate that planting exotic species that are related to native species reduces rather than enhances the resilience of forests to pathogens. | NA | no | No good fitness measure and measure host GD | NA | no | no |
| 5485 | 2020 | 44042 | Pisarenko, SV; Eremenko, EI; Ryazanova, AG; Kovalev, DA; Buravtseva, NP; Aksenova, LY; Dugarzhapova, ZF; Evchenko, AY; Kravets, EV; Semenova, OV; Bobrisheva, OV; Kuznetsova, IV; Golovinskaya, TM; Volynkina, AS; Balakhonov, SV; Kulichenko, AN | 2019 | Phylogenetic analysis of Bacillus anthracis strains from Western Siberia reveals a new genetic cluster in the global population of the species | Background Anthrax is a zoonotic disease caused by the gram-positive bacterium Bacillus anthracis. The most anthrax-endemic regions of Russia are Siberia and North Caucasus. Previously, genotyping of Russian B.anthracis isolates was carried out using canSNP and MLVA data; these methods yield lower resolution results compared to whole genome SNP analysis (wgSNP). In this research, we have used wgSNP method for genotyping of 10 B.anthracis isolates, obtained during 1961-2016 in Russia on territory of Western Siberia. Results We have analyzed 185 B.anthracis genomes available in GenBank database and genomes of 10 isolates obtained in this study to determine the place of Russian isolates in the global phylogeny of B.anthracis. For the studied genomes we have detected 7203 SNPs, which were used for building a phylogenetic reconstruction with Maximum Likelihood Method. Results of the phylogenetic analysis indicate that Russian strains belong to three different genetic groups. Three strains belong to genetic group Ames, two strains - to STI group. Five strains belong to the main genetic line B, and four of them form a subcluster, described for the first time, which we have named Siberia. Conclusions In this study, the data on genetic diversity of B.anthracis strains on the territory of Western Siberia is presented for the first time. As a result of complex phylogenetic analysis, the place of these isolates was determined in the global phylogenetic structure of the B.anthracis population. We describe a new cluster in the main genetic line B for the first time. | NA | no | No good fitness measure and measure host GD | NA | no | no |
| 5486 | 2020 | 44113 | Pisarenko, SV; Kovalev, DA; Volynkina, AS; Ponomarenko, DG; Rusanova, DV; Zharinova, NV; Khachaturova, AA; Tokareva, LE; Khvoynova, IG; Kulichenko, AN | 2018 | Global evolution and phylogeography of Brucella melitensis strains | Background: Brucellosis is a bacterial zoonotic disease. Annually in the world more than 500,000 new cases of brucellosis in humans are registered. In this study, we propose an evolutionary model of the historical distribution of B. melitensis based on the full-genomic SNP analysis of 98 strains. Results: We performed an analysis of the SNP of the complete genomes of 98 B. melitensis strains isolated in different geographical regions of the world to obtain relevant information on the population structure, genetic diversity and the evolution history of the species. Using genomic sequences of 21 strains of B. melitensis isolated in Russia and WGS data from the NCBI database, it was possible to identify five main genotypes and 13 species genotypes for analysis. Data analysis based on the Bayesian Phylogenetics and Phylogeography method allowed to determine the regions of geographical origin and the expected pathways of distribution of the main lines (genotypes and subgenotypes) of the pathogen. Conclusions: Within the framework of our study, the model of global evolution and phylogeography of B. melitensis strains isolated in various regions of the planet was proposed for the first time. The sets of unique specific SNPs described in our study, for all identified genotypes and subgenotypes, can be used to develop new bacterial typing and identification systems for B. melitensis. | NA | no | gd of pathogen | NA | no | no |
| 5487 | 2020 | 44112 | Piubelli, C; Soleymanpoor, H; Giorli, G; Formenti, F; Buonfrate, D; Bisoffi, Z; Perandin, F | 2019 | Blastocystis prevalence and subtypes in autochthonous and immigrant patients in a referral centre for parasitic infections in Italy | In this study we characterized the presence and subtype (ST1-ST4) of Blastocystis in patients attended at a referral center for tropical diseases in Northern Italy. We also, evaluated the organism’s association with other intestinal parasites. Parasite screening was performed on 756 patients, from different geographical origins (namely, Italians, Africans, South Americans, Asian and non-Italian Europeans) in which Italians represented the largest group. Blastocystis was seen to be the most prevalent parasite in the study. Subtype 3 and 1 were the most frequently found in the Italians and Africans. Our data confirmed previous studies performed in Italy, in which ST3 proved to be the most prevalent subtype, but we highlighted also a high frequency of mixed subtypes, which were probably underestimated in former analyses. Interestingly, the mixed subtypes group was the most prevalent in all the analysed geographical areas. About half of our cases showed other co-infecting parasites and the most frequent was Dientamoeba fragilis. Our study confirms that, in Blastocystis infection, multiple subtypes and co-infecting parasites are very frequently present, in particular Dientamoeba fragilis. | NA | no | human | NA | no | no |
| 5488 | 2020 | 44111 | Plenk, K; Bardy, K; Hohn, M; Kropf, M | 2019 | Long-term survival and successful conservation? Low genetic diversity but no evidence for reduced reproductive success at the north-westernmost range edge of Poa badensis (Poaceae) in Central Europe | Many steppe species reach their (north)westernmost distribution limit in western Central Europe. This also applies to Poa badensis, a rare steppe plant of calcareous rock/sand vegetation. To explore potential differences in reproductive success and genetic composition of peripheral populations, we analysed the absolute (north)westernmost occurrences in Western Germany and populations at the western margin (Eastern Austria) and the centre (Central Hungary) of the Pannonicum, representing a part of the continuous range. Specifically, we discuss the genetic and reproductive constitution of the (north)westernmost exclave and draw conclusions on the species’ biogeographical and conservation history in this region. Therefore, we used two independent molecular marker systems (AFLPs, cpDNA sequences) and a set of performance parameters. Overall, lowest regional genetic diversity was found in Western Germany, which is mainly a result of the specific history of two populations. However, this low genetic diversity was not accompanied by reduced reproductive success. The Eastern Austrian populations showed reduced genetic diversity and predominantly reduced performance, interpreted as a consequence of small population sizes. Central Hungarian populations showed the overall highest genetic diversity and comparatively high performance values. We observed high admixture and haplotype sharing between Austrian and Hungarian populations, indicating gene flow among these regions. In contrast, we interpreted the increased population differentiation within, and the clear distinctiveness of the German exclave as a long-term isolation of these (north)westernmost occurrences. Our results, overall, prove the good constitution of these populations and, together with their particular biogeographical history, highlight their conservation value. | NA | no | No good fitness measure | NA | no | no |
| 5489 | 2020 | 44040 | Plenk, K; Willner, W; Demina, ON; Hohn, M; Kuzemko, A; Vassilev, K; Kropf, M | 2020 | Phylogeographic evidence for long-term persistence of the Eurasian steppe plant Astragalus onobrychis in the Pannonian region (eastern Central Europe) | Steppe grasslands represent important refuges for rare species in the Pannonian region of eastern Central Europe. Nevertheless, little is known about their regional continuity and potential (post)glacial in-situ survival. Biogeographical studies assume potential source areas for the postglacial (re)colonisation of the Pannonian region in the Western and Central Pontics including the Black Sea coast. However, south-exposed slopes surrounding the Hungarian plain may have also played an important role as small-scale refugia. Using cpDNA sequence markers (atpI-H, rpL16, ycf1) and 190 individuals representing 25 populations, we investigated the genetic diversity and biogeographical history of Astragalus onobrychis within different subregions of its European distribution. We aimed at answering questions about the glacial survival, (post)glacial expansion and/or recent decline of the species throughout this area. A TCS network showed 108 haplotypes, which were divided into two unconnected clusters, representing different ploidy levels. We found octoploid populations widely distributed from the Pannonian region and Transylvania to more eastern areas in the Western Pontics and the Northern Caucasus. On the contrary, di- and tetraploid populations occur in the Dobruja and the Northern Balkan Peninsula, but also in the Central Pontic and the Southern Pannonian subregions. Four main cpDNA haplotype groups basically showed a geographical pattern; the Pannonian region being best-defined. We found high haplotype and moderate to high nucleotide diversity within most populations/subregions. Equilibrating these two genetic diversity measures indicates a long-term - probably glacial - (diploid) persistence of A. onobrychis in the Southern Pannonian subregion, areas along the Black Sea coast, the Northern Balkan Peninsula and the Central Pontic subregion. However, the octoploid lineage likely originated more to the east and expanded to Western Pontic areas and the Pannonian Basin probably already during Pleistocene. | NA | no | No good fitness measure | NA | no | no |
| 5490 | 2020 | 44113 | Polimanti, R; Kayser, MH; Gelernter, J | 2018 | Local adaptation in European populations affected the genetics of psychiatric disorders and behavioral traits | Background: Recent studies have used genome-wide data to investigate evolutionary mechanisms related to behavioral phenotypes, identifying widespread signals of positive selection. Here, we conducted a genome-wide investigation to study whether the molecular mechanisms involved in these traits were affected by local adaptation. Methods: We performed a polygenic risk score analysis in a sample of 2455 individuals from 23 European populations with respect to variables related to geo-climate diversity, pathogen diversity, and language phonological complexity. The analysis was adjusted for the genetic diversity of European populations to ensure that the differences detected would reflect differences in environmental exposures. Results: The top finding was related to the association between winter minimum temperature and schizophrenia. Additional significant geo-climate results were also observed with respect to bipolar disorder (sunny daylight), depressive symptoms (precipitation rate), major depressive disorder (precipitation rate), and subjective well-being (relative humidity). Beyond geo-climate variables, we also observed findings related to pathogen diversity and language phonological complexity: openness to experience was associated with protozoan diversity; conscientiousness and extraversion were associated with language consonants. Conclusions: We report that common variation associated with psychiatric disorders and behavioral traits was affected by processes related to local adaptation in European populations. | NA | no | No good fitness measure | NA | no | no |
| 5491 | 2020 | 44041 | Polonca, S | 2020 | Environment Shapes the Intra-species Diversity of Bacillus subtilis Isolates | Cosmopolitan bacteria are those that are found practically everywhere in the world. One of them is Bacillus subtilis, which can travel around the world through dust storms rising from various deserts. Upon landing, bacterial survival is determined by the ability to adjust to the heterogonous environments and bacteria isolated from extremely different environments, such as desert and riverbank soil, are expected to be less related due to the environmental pressure of each region. However, little is known about the influence of soil and habitat on B. subtilis evolution. Here, we show that desert and riverbank B. subtilis strains differ in genetic relatedness and physiological traits, such as biofilm morphology and utilisation of carbon sources. Desert strains showed more diversity at the genetic level and were able to utilise more carbon sources than riverbank strains which were highly genetically conserved. Biofilm morphologies of desert and riverbank strains generally segregated and both groups formed different morphology clusters despite the astonishing diversity observed among riverbank strains. We also show that relatedness of B. subtilis strains does not decrease with distance inside the same habitat, which, together with diversity data implies that the difference in environmental selection pressures plays a fundamental role in the evolution of this species. | NA | no | No good fitness measure and measure host GD | NA | no | no |
| 5492 | 2020 | 44111 | Portanier, E; Garel, M; Devillard, S; Maillard, D; Poissant, J; Galan, M; Benabed, S; Poirel, MT; Duhayer, J; Itty, C; Bourgoin, G | 2019 | Both candidate gene and neutral genetic diversity correlate with parasite resistance in female Mediterranean mouflon | BackgroundParasite infections can have substantial impacts on population dynamics and are accordingly a key challenge for wild population management. Here we studied genetic mechanisms driving parasite resistance in a large herbivore through a comprehensive approach combining measurements of neutral (16 microsatellites) and adaptive (MHC DRB1 exon 2) genetic diversity and two types of gastrointestinal parasites (nematodes and coccidia).ResultsWhile accounting for other extrinsic and intrinsic predictors known to impact parasite load, we show that both neutral genetic diversity and DRB1 are associated with resistance to gastrointestinal nematodes. Intermediate levels of multi-locus heterozygosity maximized nematodes resistance, suggesting that both in- and outbreeding depression might occur in the population. DRB1 heterozygosity and specific alleles effects were detected, suggesting the occurrence of heterozygote advantage, rare-allele effects and/or fluctuating selection. On the contrary, no association was detected between genetic diversity and resistance to coccidia, indicating that different parasite classes are impacted by different genetic drivers.ConclusionsThis study provides important insights for large herbivores and wild sheep pathogen management, and in particular suggests that factors likely to impact genetic diversity and allelic frequencies, including global changes, arealso expected to impact parasite resistance. | NA | no | no good gd measure host pop | NA | no | no |
| 5493 | 2020 | 44112 | Pospisilova, P; Grange, PA; Grillova, L; Mikalova, L; Martinet, P; Janier, M; Vermersch, A; Benhaddou, N; Del Giudice, P; Alcaraz, I; Truchetet, F; Dupin, N; Smajs, D | 2018 | Multi-locus sequence typing of Treponema pallidum subsp pallidum present in clinical samples from France: Infecting treponemes are genetically diverse and belong to 18 allelic profiles | Treponema pallidum subsp. pallidum, the causative agent of sexually transmitted syphilis, detected in clinical samples from France, was subjected to molecular typing using the recently developed Multilocus Sequence Typing system. The samples (n = 133) used in this study were collected from 2010-2016 from patients with diagnosed primary or secondary syphilis attending outpatient centers or hospitals in several locations in France. Altogether, 18 different allelic profiles were found among the fully typed samples (n = 112). There were five allelic variants identified for TP0136, 12 for TP0548, and eight for TP0705. Out of the identified alleles, one, seven, and three novel alleles were identified in TP0136, TP0548, and TP0705, respectively. Partial allelic profiles were obtained from 6 samples. The majority of samples (n = 110) belonged to the SS14-like cluster of TPA isolates while 7 clustered with Nichols-like isolates. Patients infected with Nichols-like samples were more often older (p = 0.041) and more often diagnosed with secondary syphilis (p = 0.033) compared to patients infected with SS14-like samples. In addition, macrolide resistance caused by the A2058G mutation was found to be associated with allelic profile 1.3.1 or with strains belonging to the 1.3.1 lineage (p<0.001). The genetic diversity among TPA strains infecting the European population was surprisingly high, which suggests that additional studies are needed to reveal the full genetic diversity of TPA pathogens infecting humans. | NA | no | gd of pathogen | NA | no | no |
| 5494 | 2020 | 44113 | Potter, KM; Campbell, AR; Josserand, SA; Nelson, CD; Jetton, RM | 2017 | Population isolation results in unexpectedly high differentiation in Carolina hemlock (Tsuga caroliniana), an imperiled southern Appalachian endemic conifer | Carolina hemlock (Tsuga caroliniana Engelm.) is a rare conifer species that exists in small, isolated populations within a limited area of the Southern Appalachian Mountains of the USA. As such, it represents an opportunity to assess whether population size and isolation can affect the genetic diversity and differentiation of a species capable of longdistance gene flow via wind-dispersed pollen and seed. This information is particularly important in a gene conservation context, given that Carolina hemlock is experiencing mortality throughout its range as a result of infestation by hemlock wooly adelgid (Adelges tsugae Annand), an exotic insect. In this study, 439 Carolina hemlock trees from 29 areas (analyzed as populations) were sampled, representing an extensive range-wide sampling of the species. Data from 12 polymorphic nuclear microsatellite loci were collected and analyzed for these samples. The results show that populations of Carolina hemlock are extremely inbred (FIS = 0.713) and surprisingly highly differentiated from each other (FST = 0.473) with little gene flow (N-m = 0.740). Additionally, most populations contained at least one unique allele. This level of differentiation is unprecedented for a North American conifer species. Numerous genetic clusters were inferred using two different clustering approaches. The results clearly demonstrate that, existing as a limited number of small and isolated populations, Carolina hemlock has insufficient gene flow to avoid widespread genetic drift and inbreeding, despite having the capacity to disperse pollen and seed relatively long distances by wind. These results have important conservation implications for this imperiled species. | NA | no | No good fitness measure | NA | no | no |
| 5495 | 2020 | 44039 | Prathiviraj, R; Chellapandi, P | 2020 | Evolutionary genetic analysis of unassigned peptidase clan-associated microbial virulence and pathogenesis | Peptidase clan is the largest group of proteases with common ancestry as identified by structural homology. A peptidase with unknown catalytic type is referred to as an unassigned peptidase clan, which can be classified into 8 peptidase families (U32, U40, U49, U56, U57, U62, U69, and U72). The members of this clan are widely dispersed in diverse microbial pathogens and their apparent involvement in the microbial virulence is not yet known for antibiotic drug discovery. In the present study, we have analyzed their common structural and functional characteristics using evolutionary genetic analysis. As shown by our analysis, the molecular and functional characteristics of this clan diverged across the microbial pathogens. It also indicates that the members of each family might have evolved independently and a peptidase core converged to interconnect the unassigned peptidase clan. Several evolutionary constraints have been identified as selective measures from this clan that inferred on their functional evolution and divergence. Genetic diversity analysis described that many members of this clan have evolved as new molecular functions across the microbial pathogens by imposing the Darwinian positive selection. Structural analysis of this study indicates that members of this clan have a conserved fold, convergence in functional parts, and divergence in spatial structural arrangements. As the results of our study, the neofunctionalization of several unassigned peptidases provides a full virulence for microbial pathogens occupying at the different pathophysiological niche. | NA | no | No good fitness measure and measure host GD | NA | no | no |
| 5496 | 2020 | 44040 | Premier, J; Fickel, J; Heurich, M; Kramer-Schadt, S | 2020 | The boon and bane of boldness: movement syndrome as saviour and sink for population genetic diversity | Background Many felid species are of high conservation concern, and with increasing human disturbance the situation is worsening. Small isolated populations are at risk of genetic impoverishment decreasing within-species biodiversity. Movement is known to be a key behavioural trait that shapes both demographic and genetic dynamics and affects population survival. However, we have limited knowledge on how different manifestations of movement behaviour translate to population processes. In this study, we aimed to 1) understand the potential effects of movement behaviour on the genetic diversity of small felid populations in heterogeneous landscapes, while 2) presenting a simulation tool that can help inform conservation practitioners following, or considering, population management actions targeting the risk of genetic impoverishment. Methods We developed a spatially explicit individual-based population model including neutral genetic markers for felids and applied this to the example of Eurasian lynx. Using a neutral landscape approach, we simulated reintroductions into a three-patch system, comprising two breeding patches separated by a larger patch of differing landscape heterogeneity, and tested for the effects of various behavioural movement syndromes and founder population sizes. We explored a range of movement syndromes by simulating populations with various movement model parametrisations that range from ‘shy’ to ‘bold’ movement behaviour. Results We find that movement syndromes can lead to a higher loss of genetic diversity and an increase in between population genetic structure for both bold and shy movement behaviours, depending on landscape conditions, with larger decreases in genetic diversity and larger increases in genetic differentiation associated with bold movement syndromes, where the first colonisers quickly reproduce and subsequently dominate the gene pool. In addition, we underline the fact that a larger founder population can offset the genetic losses associated with subpopulation isolation and gene pool dominance. Conclusions We identified a movement syndrome trade-off for population genetic variation, whereby bold-explorers could be saviours - by connecting populations and promoting panmixia, or sinks - by increasing genetic losses via a ‘founder takes all’ effect, whereas shy-stayers maintain a more gradual genetic drift due to their more cautious behaviour. Simulations should incorporate movement behaviour to provide better projections of long-term population viability and within-species biodiversity, which includes genetic diversity. Simulations incorporating demographics and genetics have great potential for informing conservation management actions, such as population reintroductions or reinforcements. Here, we present such a simulation tool for solitary felids. | NA | no | No good fitness measure | NA | no | no |
| 5497 | 2020 | 44039 | Presotto, A; Hernandez, F; Casquero, M; Vercellino, R; Pandolfo, C; Poverene, M; Cantamutto, M | 2020 | Seed bank dynamics of an invasive alien species, Helianthus annuus L. | Aims The ability to form persistent seed banks is one of the best predictors of species’ potential to establish in new ranges. Wild sunflower is native to North America where the formation of persistent seed banks is promoted by disturbance and it plays a key role on the establishment and persistence of native populations. However, the role of the seed banks on the establishment and persistence of invasive populations has not been studied. Here, we evaluated the role of seed bank and disturbance on the establishment and fitness, and seed persistence in the soil in several sunflower biotypes collected in ruderal (wild Helianthus annuus) and agrestal (natural crop wild hybrid) habitats of Argentina as well as volunteer populations (progeny of commercial cultivars). Methods In a seed-bank experiment, we evaluated emergence, survival to reproduction, survival of emerged seedlings, inflorescences per plant and per plot under disturbed and undisturbed conditions over 2 years; in a seed-burial experiment, we evaluated seed persistence in the soil over four springs (6, 18, 30 and 42 months). Important Findings Overall, seedling emergence was early in the growing season (during winter), and it was promoted by disturbance, especially in the first year. Despite this, the number of inflorescences per plot was similar under both conditions, especially in ruderals. In the second year, emergence from the seed bank was much lower, but the survival rate was higher. In the seed-burial experiment, genetic differences were observed but seeds of ruderals arid agrestals persisted up to 42 months while seeds of the volunteer did not persist longer than 6 months. The agrestal biotype showed an intermediate behavior between ruderals and volunteers in both experiments. Our findings showed that wild and crop-wild sunflower can form persistent seed banks outside its native range and that disturbance may facilitate its establishment in new areas. | NA | no | No good fitness measure and measure host GD | NA | no | no |
| 5498 | 2020 | 44113 | Prosser, C; Meyer, W; Ellis, J; Lee, R | 2018 | Resistance screening and trend analysis of imported falciparum malaria in NSW, Australia ( 2010 to 2016) | Background The World Health Organization currently recommends artemisinin (along with a partner drug) as the global frontline treatment for Plasmodium falciparum malaria. Artemisinin resistant P. falciparum are now found throughout the greater Mekong subregion of South East Asia. Several polymorphisms in the parasite’s kelch gene have been demonstrated to confer artemisinin resistance. While genotypes within the greater Mekong subregion are thoroughly examined in the literature, P. falciparum populations within several areas that do not (yet) have endemic resistance are underrepresented. Results This investigation characterised the Pfkelch13 propeller domains from 153 blood samples of 140 imported cases of P. falciparum malaria in New South Wales from 2010 to 2016. A low level of propeller domain diversity was observed, including the C580 (Y) under bar coding mutation most strongly associated with artemisinin resistance in South East Asia. The resistance genotype was found in a sample originating in Papua New Guinea, where this mutation, or artemisinin treatment failure, have not been previously reported. Sequencing a panel of geographically informative polymorphisms within the organellar genomes identified the C580 (Y) under bar parasite as having Oceanic origins. Patient data analysis revealed that New South Wales, Australia, P. falciparum malaria cases often originated from regions with limited drug resistance screening. Conclusions The C580 (Y) under bar finding from outside of the greater Mekong subregion supports the consensus to upscale molecular surveillance of artemisinin resistance outside of South East Asia. The genetic screening results identify a risk of importing resistant falciparum malaria to Australia, supporting an ongoing surveillance protocol to pre-empt treatment failure and contribute to global data gathering. | NA | no | No good fitness measure and measure host GD | NA | no | no |
| 5499 | 2020 | 44040 | Pu, S; Qi, G; Yang, J; Wang, N; Guo, J; Zhang, P; Yang, X; Liu, L; Li, C | 2020 | COMPARATION OF SYMPTOM TYPE DIFFERENCES IN ENVIRONMENTAL PRICKLYASH RUST AND POPULATION GENETIC STRUCTURE OF COLEOSPORIUM | Pricklyash has important economic, medicinal and ecological value. Pricklyash rust is one of the main diseases of the plant, it often causes discoloration, necrosis and abscission of Pricklyash leaves. When the disease is serious, it can cause entire leaves of pricklyash to fall off and infect repeatedly, which can lead to the weakening of pricklyash, even death, and seriously affects its yield and quality. The spore morphology of pricklyash rust with different symptoms was observed and ITS sequence was analyzed and compared, the results showed that 60 samples of pricklyash rust collected from wild pricklyash, Qujing pricklyash, red pricklyash and green pricklyash belong to Basidio-mycotina, Teliomycetes, Uredinales, Coleosporaceae, Coleosporium, Coleosporium zanthoxyli. By comparing and analyzing the results of previous studies on Dendrobium rust with of pricklyash rust, it was found that Dendrobium rust and pricklyash rust were clustered in two populations and the genetic similarity coefficient of ISSR was 0.67. The genetic diversity of Dendrobium rust was richer than of pricklyash rust. | NA | no | No good fitness measure and measure host GD | NA | no | no |
| 5500 | 2020 | 44112 | Pugliese, M; Matic, S; Prethi, S; Gisi, U; Gullino, ML | 2018 | Molecular characterization and sensitivity to demethylation inhibitor fungicides of Aspergillus fumigatus from orange-based compost | Aspergillus fumigatus, the causal agent of human aspergilloses, is known to be non-pathogenic in plants. It is present as saprophyte in different types of organic matter and develops rapidly during the high-temperature phase of the composting process. Aspergilloses are treated with demethylation inhibitor (DMI) fungicides and resistant isolates have been recently reported. The present study aims to estimate the abundance, genetic diversity and DMI sensitivity of A. fumigatus during the composting process of orange fruits. Composting of orange fruits resulted in a 100-fold increase in A. fumigatus frequency already after 1 week, demonstrating that the degradation of orange fruits favoured the growth of A. fumigatus in compost. Most of A. fumigatus isolates belonged to mating type 2, including those initially isolated from the orange peel, whereas mating type 1 evolved towards the end of the composting process. None of the A. fumigatus isolates expressed simultaneously both mating types. The 52 investigated isolates exhibited moderate SSR polymorphisms by formation of one major (47 isolates) and one minor cluster (5 isolates). The latter included mating type 1 isolates from the last sampling and the DMI-resistant reference strains. Only few isolates showed cyp51A polymorphisms but were sensitive to DMIs as all the other isolates. None of the A. fumigatus isolates owned any of the mutations associated with DMI resistance. This study documents a high reproduction rate of A. fumigatus during the composting process of orange fruits, requesting specific safety precautions in compost handling. Furthermore, azole residue concentrations in orange-based compost were not sufficient to select A. fumigatus resistant genotypes. | NA | no | No good fitness measure and measure host GD | NA | no | no |
| 5501 | 2020 | 44039 | Purohit, D; Ram, MS; Pandey, VK; Pravalika, S; Deka, PJ; Narayan, G; Umapathy, G | 2020 | Cross-specific markers reveal retention of genetic diversity in captive-bred pygmy hog, a critically endangered suid | As part of the species recovery plan for the critically endangered pygmy hog (Porcula salvania), a conservation-breeding program was initiated, to bolster its wild population. For successful conservation-breeding, it is essential to maintain 90% of the founder genetic diversity over time. Therefore, in the present study, we assessed the genetic diversity of a captive population of pygmy hog across generations using a set of ten, cross-specific microsatellite markers. Our results indicated a genetically heterozygous captive population (H-E = 0.603), with stable expected heterozygosities across generations. However, the most recent generation showed a significant decrease in individual heterozygosities, implying possible genetic inbreeding. The current findings warrant a need for genetic evaluation to inform future conservation-breeding decisions. In addition, we also designed and tested primers for PCR-based species and sex-identification in the pygmy hog. The markers standardised in the present study would also help in evaluating the survival and ecology of the reintroduced populations. | NA | no | only one pop, no good fitness measure, maybe inbred? | NA | no | no |
| 5502 | 2020 | 44112 | Qasim, M; Wang, BH; Zou, HS; Lin, YW; Dash, CK; Bamisile, BS; Hussai, M; Zhao, ZW; Wang, LD | 2018 | Phylogenetic relationship and genetic diversity of citrus psyllid populations from China and Pakistan and their associated Candidatus bacterium | Asian citrus psyllid (ACP), Diaphorina citri Kuwayama (Hemiptera: Liviidae), is a severely devastating pest of Rutaceae plants, mainly citrus, across the globe, and causal agent of Huanglongbing (HLB) disease. To find out the genetic relationship and diversity among the populations of ACP and associated Candidatus Liberibacter asiaticus (CLas) from two countries (China and Pakistan), sequence data of three different genes, cytochrome oxidase subunit I (COI), Cu-transporting protein (ATOX1) and 16S rRNA, were used to characterize all populations. In the present study, MEGA-7 and statistical parsimony software (TCS-1.2) were used to depict the phylogenetic relationship among all populations under both genes, whereas diversity was calculated by DnaSP v5. All analyses were done for country wise and overall relationship among all populations. For ACP populations, both genes presented a significant strong intermingled relationship among all populations and put all population into a single haplotype (Dcit-2), which proved similarity between Chinese and Pakistani populations. Moreover, for CLas strains, 16S gene also presented strong relationship for all sampled populations. All three genes of ACP and CLas populations elucidated more than 95% resemblance to each other. On the other hand, a significant genetic variation was observed by three genes for overall populations, although, country wise variation was nonsignificant between all collected populations. ATOX1 gene presented higher diversity through Fu’s Fs test (pi = 0.01081, p < 0.003) whereas COI gene gave less diversity under Fu’s Fs and Tajima’s D test (pi = 0.00512, p < 0.000 and 0.05, respectively). Similarly, nucleotide mismatch distribution also had shown enough genetic variation among all ACP populations, under both genes. Our sequence data for both genes proved the invasion of the Chinese ACP population (Dcit-2) into Pakistan, through all phylogenetic relationship, which proved a similar genetic makeup among all ACP populations from both countries. Therefore, these results can be helpful to utilize any novel designed control measure equally for both countries. | NA | no | No good fitness measure and measure host GD | NA | no | no |
| 5503 | 2020 | 44112 | Qi, Y; Zhao, W; Huang, YJ; Wang, XN; Zhao, YY | 2019 | Correlation between Climatic Factors and Genetic Diversity of Phrynocephalus forsythii | Global climate change is a threat to animals in nearly all biomes and ecosystems, especially for ectotherm whose life activities highly depend on environmental thermal regime. Population genetic diversity which is essential for adaptation to environmental change is a useful index for long-term species survival. In this paper, genetic diversity of eight Phrynocephalus forsythii population which distributed in Tarim Basin, China, were evaluated based on three mtDNA gene and its correlation with environment factors were investigated using RDA. Our result revealed that, the level of genetic diversity of P. forsythii populations was related to its location but there was no significant correlation between genetic distances and geographic distances in P. forsythii. However, we find that mtDNA of P. forsythii was subjected to selection pressure during evolution and population genetic diversity was significantly positively related to variation coefficient of rainfall (VCR) and altitude (AL), while significantly negatively related to longitude (N) and annual average temperature (AAT). Our result supported the previous prediction that excessive ambient heat is a threat to R. forsythii. | NA | no | No good fitness measure | NA | no | no |
| 5504 | 2020 | 44113 | Qiang, ZZ; Wang, Y; Li, S; Wang, MW; Luo, XD; Li, X; Feng, XL; Li, CY | 2018 | Efficiency of ISSR marker in assessing the genetic diversity of wild and cultivated Hedysarum polybotrys Hand. Mazz | Genetic variation of cultivated and wild populations plays an important role in maintaining the population gene polymorphism and developing future management strategies. Hedysarum polybotrys is an important economic plant in Gansu Province, China. This paper was the first to investigate and analyse the genetic diversity between wild and cultivated H. polybotrys. The results of the ISSR-PCR revealed that 479 (98.97%) of the 484 ISSR loci tested by 17 primers were polymorphic. The change of PPL was 92.98-93.80%. The species level of the alleles, the effective number of alleles, the Nei’s gene diversity and the Shannon’s information index were 1.9897, 1.6006, 0.3502 and 0.5228, respectively. The high-low sequence of the genetic diversity of the wild population was greater than that of the cultivated population. The genetic distance and genetic identity between the cultivated and wild populations were 0.0488 and 0.9524. AMOVA analysis indicated that genetic variation mainly occurred among populations (8.09%, 6.47%, respectively). UPGMA analysis revealed that the 52 samples were clustered into two branches, I and II. Although most of the alleles from the wild populations were maintained in the gene pool of the cultivated populations, a small amount of the alleles remained in the wild population. PCoA and population structure analysis confirmed the partitioning results of the UPGMA clustering. The H. polybotrys populations had a high level of genetic diversity, and the genetic differentiation of two populations was low. Results provided a theoretical basis for the protection and utilisation of H. polybotrys resources. | NA | no | No good fitness measure | NA | no | no |
| 5505 | 2020 | 44113 | Qin, J; Song, QJ; Shi, AN; Li, S; Zhang, MC; Zhang, B | 2017 | Genome-wide association mapping of resistance to Phytophthora sojae in a soybean [Glycine max (L.) Merr.] germplasm panel from maturity groups IV and V | Phytophthora sojae, an oomycete pathogen of soybean, causes stem and root rot, resulting in annual economic loss up to $2 billion worldwide. Varieties with P. sojae resistance are environmental friendly to effectively reduce disease damages. In order to improve the resistance of P. sojae and broaden the genetic diversity in Southern soybean cultivars and germplasm in the U.S., we established a P. sojae resistance gene pool that has high genetic diversity, and explored genomic regions underlying the host resistance to P. sojae races 1, 3, 7, 17 and 25. A soybean germplasm panel from maturity groups (MGs) IV and V including 189 accessions originated from 10 countries were used in this study. The panel had a high genetic diversity compared to the 6,749 accessions from MGs IV and V in USDA Soybean Germplasm Collection. Based on disease evaluation dataset of these accessions inoculated with P. sojae races 1, 3, 7, 17 and 25, which are publically available, five accessions in this panel were resistant to all races. Genome-wide association analysis identified a total of 32 significant SNPs, which were clustered in resistance-associated genomic regions, among those, ss715619920 was only 3kb away from the gene Glyma. 14g087500, a subtilisin protease. Gene expression analysis showed that the gene was down-regulated more than 4 fold (log2 fold > 2.2) in response to P. sojae infection. The identified molecular markers and genomic regions that are associated with the disease resistance in this gene pool will greatly assist the U.S. Southern soybean breeders in developing elite varieties with broad genetic background and P. sojae resistance. | NA | no | dometicated host | NA | no | no |
| 5506 | 2020 | 44043 | Qiu, G; Rui, YP; Yi, BC; Liu, T; Hao, ZJ; Li, X; Zhang, LH; Huang, SC; Li, K; Han, ZQ | 2019 | Identification and Genomic Analysis of a Pathogenic Strain of Mycoplasma hyopneumoniae (TB1) Isolated from Tibetan Pigs | The present study aims to identify the species and strains of Mycoplasma hyopneumoniae isolated from Tibetan pigs (Mh TB1) at the genetic level for understanding the basis of its pathogenicity. Mh TB1 was isolated from the consolidated lungs of Tibetan pigs by liquid culture and agar plate colony method. Polymerase chain reaction (PCR) amplification of the 16S recombinant DNA (rDNA) conservative sequence and a species-specific gene (P36) of Mh provided species confirmation. PCR products were imaged on gels and shotgun sequencing was performed. DNA sequences were compared for assessing genetic similarity between Mh TB1 and Mh reference strains in the GenBank database. The isolated strains were >98% similar to the Mh reference strains. Genomic analysis revealed significant sequence conservation between Mh TB1 and the reference strains; however, differential genes were more prevalent in Mh TB1 than in other reported strains. Therefore, we concluded that Mh is a major pathogen of Tibetan pigs that cause enzootic pneumonia. The Mh TB1 strain harbors more genes and specific virulence factors, consistent with its plateau-related adaptability to hypoxia and virulence. Differential gene analysis revealed gene variations in the inclement plateau environment, enriched gene pool, and plateau adaptability of the Mh TB1 strain, which will be important for vaccine development. | NA | no | gd of pathogen | NA | no | no |
| 5507 | 2020 | 44039 | Quigley, KM; Bay, LK; van Oppen, MJH | 2020 | Genome-wide SNP analysis reveals an increase in adaptive genetic variation through selective breeding of coral | Marine heat waves are increasing in magnitude, duration, and frequency as a result of climate change and are the principal global driver of mortality in reef-building corals. Resilience-based genetic management may increase coral heat tolerance, but it is unclear how temperature responses are regulated at the genome level and thus how corals may adapt to warming naturally or through selective breeding. Here we combine phenotypic, pedigree, and genomic marker data from colonies sourced from a warm reef on the Great Barrier Reef reproductively crossed with conspecific colonies from a cooler reef to produce combinations of warm purebreds and warm-cool hybrid larvae and juveniles. Interpopulation breeding created significantly greater genetic diversity across the coral genome compared to breeding between populations and maintained diversity in key regions associated with heat tolerance and fitness. High-density genome-wide scans of single nucleotide polymorphisms (SNPs) identified alleles significantly associated with larval families reared at 27.5 degrees C (87-2,224 loci), including loci putatively associated with proteins involved in responses to heat stress (cell membrane formation, metabolism, and immune responses). Underlying genetics of these families explained 43% of PCoA multilocus variation in survival, growth, and bleaching responses at 27.5 degrees C and 31 degrees C at the juvenile stage. Genetic marker contribution to total variation in fitness traits (narrow-sense heritability) was high for survival but not for growth and bleaching in juveniles, with heritability of these traits being higher at 31 degrees C relative to 27.5 degrees C. While based on only a limited number of crosses, the mechanistic understanding presented here demonstrates that allele frequencies are affected by one generation of selective breeding, key information for the assessments of genetic intervention feasibility and modelling of reef futures. | NA | no | No good fitness measure and measure host GD | NA | no | no |
| 5508 | 2020 | 44042 | Rachlin, A; Kleinecke, M; Kaestli, M; Mayo, M; Webb, JR; Rigas, V; Shilton, C; Benedict, S; Dyrting, K; Currie, BJ | 2019 | A cluster of melioidosis infections in hatchling saltwater crocodiles (Crocodylus porosus) resolved using genome-wide comparison of a common north Australian strain of Burkholderia pseudomallei | Burkholderia pseudomallei is a Gram-negative saprophytic bacillus and the aetiological agent of melioidosis, a disease of publichealth importance throughout Southeast Asia and northern Australia. Infection can occur in humans and a wide array of animal species, though zoonotic transmission and case clusters are rare. Despite its highly plastic genome and extensive strain diversity, fine-scale investigations into the population structure of B. pseudomallei indicate there is limited geographical dispersal amongst sequence types (STs). In the ‘Top End’ of northern Australia, five STs comprise 90% of the overall abundance, the most prevalent and widespread of which is ST-109. In May 2016, ST-109 was implicated in two fatal cases of melioidosis in juvenile saltwater crocodiles at a wildlife park near Darwin, Australia. To determine the probable source of infection, we sampled the crocodile enclosures and analysed the phylogenetic relatedness of crocodile and culture-positive ST-109 environmental park isolates against an additional 135 ST-109 B. pseudomallei isolates from the Top End. Collectively, our whole-genome sequencing (WGS) and pathology findings confirmed B. pseudomallei detected in the hatchling incubator as the likely source of infection, with zero SNPs identified between clinical and environmental isolates. Our results also demonstrate little variation across the ST-109 genome. with SNPs in recombinogenic regions and one suspected case of ST homoplasy accounting for nearly all observed diversity. Collectively, this study supports the use of WGS for outbreak source attribution in highly recombinogenic pathogens, and confirms the epidemiological and phylogenetic insights that can be gained from high-resolution sequencing platforms. | NA | no | No good fitness measure and measure host GD | NA | no | no |
| 5509 | 2020 | 44040 | Rafiei, A; Baghlaninezhad, R; Koster, PC; Bailo, B; de Mingo, MH; Carmena, D; Panabad, E; Beiromvand, M | 2020 | Multilocus genotyping of Giardia duodenalis in Southwestern Iran. A community survey | Giardia duodenalis is one of the main enteric pathogens associated with diarrheal disease. In developing countries, giardiasis is a major public health concern, particularly in children under five years of age. This study aimed to evaluate the occurrence and genetic diversity of G. duodenalis causing human infections in Shushtar County, Southwestern Iran. Individual faecal specimens were collected from 1,163 individuals (male/female ratio: 0.9; age range 2-75 years) with (n = 258) and without (n = 905) gastrointestinal symptoms living in rural and urban settings during the period 2017-2018. Conventional (sucrose flotation and microscopy) methods were used for the initial detection of G. duodenalis cysts in faecal specimens. Microscopy-positive samples were confirmed by PCR amplification and sequencing of the small subunit rRNA (ssu rRNA) gene of the parasite. A multilocus genotyping (MLG) scheme targeting the triose phosphate isomerase (tpi), the glutamate dehydrogenase (gdh), and the beta-giardin (bg) genes was used for genotyping purposes. Giardia duodenalis cysts were detected in 7.7% (90/1,163) of samples by microscopy, of which 82 were confirmed by ssu-PCR. Successful amplification and sequencing results were obtained for 23.2% (19/82), 9.8% (8/82), and 8.5% (7/82) of the confirmed samples at the tpi, gdh, and bg loci, respectively. MLG data for the three loci were available for two samples only. Out of the 24 samples genotyped at any loci, 50% (12/24) were identified as assemblage A and the remaining half as assemblage B. Overall, AII was the most prevalent sub-assemblage detected (41.7%, 10/24), followed by BIII (25.0%, 6/24), discordant BIII/BIV (5/24) or AII/AIII (2/24) sequences, and BIV (1/24). No significant correlation was demonstrated between a given assemblage/sub-assemblage and the occurrence of clinical symptoms. No genotypes adapted to animal hosts other than humans (e.g. assemblages C-F) were found circulating in the investigated human population, suggesting that transmission of human giardiasis in this Iranian region is primarily of anthroponotic nature. Further molecular-based studies are needed to confirm and expand these results, and to ascertain the presence and public health relevance of the parasite in environmental (e.g. drinking water) samples. | NA | no | gd of pathogen | NA | no | no |
| 5510 | 2020 | 44111 | Rakov, AV; Mastriani, E; Liu, SL; Schifferli, DM | 2019 | Association of Salmonella virulence factor alleles with intestinal and invasive serovars | BackgroundThe role of Salmonella virulence factor (VF) allelic variation in modulating pathogenesis or host specificity has only been demonstrated in a few cases, mostly through serendipitous findings. Virulence factor (VF) alleles from Salmonella enterica subsp. enterica genomes were compared to identify potential associations with the host-adapted invasive serovars Typhi, Dublin, Choleraesuis, and Gallinarum, and with the broad host-range intestinal serovars Typhimurium, Enteritidis, and Newport.ResultsThrough a bioinformatics analysis of 500 Salmonella genomes, we have identified allelic variants of 70 VFs, many of which are associated with either one of the four host-adapted invasive Salmonella serovars or one of the three broad host-range intestinal serovars. In addition, associations between specific VF alleles and intra-serovar clusters, sequence types (STs) and/or host-adapted FimH adhesins were identified. Moreover, new allelic VF associations with non-typhoidal S. Enteritidis and S. Typhimurium (NTS) or invasive NTS (iNTS) were detected.ConclusionsBy analogy to the previously shown association of specific FimH adhesin alleles with optimal binding by host adapted Salmonella serovars, lineages or strains, we predict that some of the identified association of other VF alleles with host-adapted serovars, lineages or strains will reflect specific contributions to host adaptation and/or pathogenesis. The identification of these allelic associations will support investigations of the biological impact of VF alleles and better characterize the role of allelic variation in Salmonella pathogenesis. Most relevant functional experiments will test the potential causal contribution of the detected FimH-associated VF variants in host adapted virulence. | NA | no | No good fitness measure and measure host GD | NA | no | no |
| 5511 | 2020 | 44113 | Rangaraju, A; Anbarasu, K; Sridhar, MS; Putta, SR; Daga, S | 2017 | Novel mutations of ATP7B gene in Wilson’s disease patients of South Indian cohort | Wilson Disease is an autosomal recessive inherited disorder caused by abnormal copper metabolism. Mutations in P-type adenosine triphosphatase -ATP7B gene are known to result in excessive copper deposition in liver, brain and cornea leading to hepatic, and neuropsychiatric manifestations. Wide clinical and genetic heterogeneity is observed despite being a monogenic condition, making diagnosis difficult. Unlike other populations where a common causal mutation has been established, Indian population has revealed heterogeneous data attributing to its genetic diversity. Therefore we considered screening of ATP7B gene to identify any novel disease causing mutation and establish genotype-phenotype correlation. Genetic screening of the entire coding region of ATP7B gene was carried out in thirteen WD patients and nine of available family members by PCR based direct sequencing for hotspot exons 8, 13, 14, 15 and 18 and PCR based SSCP analysis for the rest of 16 exons. We report five novel mutations (3 missense, 1 frameshift and 1 intronic) and five reported mutations (2 missense, 1 silent, 1 intronic and 1 in 5’UTR). Mutations were mostly observed in hotspot exons 8, 13 and 18. Genotype-phenotype correlation revealed predominance of mutations in certain clinical subtype along with regional variation. Molecular genetic analysis has proved to be the most reliable method for confirmation of clinical diagnosis and identification of pre-symptomatic individuals, however screening of the entire gene can be time consuming and an expensive method. Since the hotspot exons: 8, 13, 14, 15, and 18 constitute for nearly 85% of the mutations, screening of these exons can be an efficient and an economical option. | NA | no | human | NA | no | no |
| 5512 | 2020 | 44113 | Rangel, J; Gonzalez, A; Stoner, M; Hatter, A; Traver, BE | 2018 | Genetic diversity and prevalence of Varroa destructor, Nosema apis, and N-ceranae in managed honey bee (Apis mellifera) colonies in the Caribbean island of Dominica, West Indies | Honey bees (Apis mellifera) are arguably the most important insect pollinators of major agricultural crops around the world. Therefore, it is important to assess the health of managed honey bees in every region, particularly in areas where this information remains unknown. In 2015 and 2016, several managed apiaries were surveyed on the Caribbean Island of Dominica, West Indies. We measured the levels of the ectoparasitic mite, Varroa destructor, and analyzed the levels of Nosema apis and Nosema ceranae using spore counts and qPCR. Colonies sampled in 2015 were also analyzed for subspecies composition using mitochondrial DNA. Varroa levels were low overall (1-7 mites per 100 bees), with many colonies exhibiting no mites. In apiaries where varroa counts were performed in both years, there were significantly more mites in 2016 than in 2015. Overall, colonies exhibited low Nosema spp. spore counts, with only 1.33% of the bees analyzed scoring positive for spores. qPCR revealed that of the 30 colonies analyzed, all but six (82.3% of the total) were infected with low levels of N. apis. The prevalence of N. ceranae infection was higher, with 97.1% of the bees analyzed being infected. The majority of colonies analyzed (84.4%) exhibited the M4 haplotype (A. m. mellifera), while the remaining (15.6%) exhibited the C1 haplotype (A. m. ligustica). We did not find any colonies that had the African A. m. scutellata haplotype. This study is the first survey conducted to assess the genetic structure and health status of managed honey bees in Dominica. | NA | no | no good gd measure host pop | NA | no | no |
| 5513 | 2020 | 44113 | Ranke, PS; Skjelseth, S; Parn, H; Herfindal, I; Pedersen, AAB; Stokke, BG; Kvalnes, T; Ringsby, TH; Saether, BE; Jensen, H | 2017 | Demographic influences of translocated individuals on a resident population of house sparrows | Translocation of individuals from source populations to augment small populations facing risk of extinction is an important conservation tool. Here we examine sex-specific differences between resident and translocated house sparrows Passer domesticus in reproductive success and survival, and the contribution of translocated individuals to the growth of a local population. We found evidence for assortative mating based on origin revealed by fewer parentages between translocated males and resident females than expected, and the total number of fledglings produced by such pairs was lower. The reproductive success of translocated males was positively related to the size of the throat badge (a sexual ornament), such that only translocated males with a large badge size were as successful as resident males. However, offspring with parents of different origin had higher survival than offspring with parents of the same origin, which suggests hybrid vigour. The contribution of resident and translocated individuals to the stochastic component of the long-run growth rate of the population was similar; neither the mean individual contributions in fitness nor the demographic variance differed between the two groups. Thus, this experiment shows that translocated individuals may have a similar demographic influence on the growth of local populations as resident individuals. Still, the intermixing of translocated and resident individuals was low, and fitness differed according to origin in relation to individual differences in a sexually selected trait. In addition, hybrid vigour with respect to offspring recruitment seemed to partially decrease the negative fitness consequences of the assortative mating based on origin. | NA | no | no good gd measure host pop | NA | no | no |
| 5514 | 2020 | 44039 | Rao, PL; Gandham, RK; Subbiah, M | 2020 | Molecular evolution and genetic variations of V and W proteins derived by RNA editing in Avian Paramyxoviruses | The newly assigned subfamily Avulavirinae in the family Paramyxoviridae includes avian paramyxoviruses (APMVs) isolated from a wide variety of avian species across the globe. Till date, 21 species of APMVs are reported and their complete genome sequences are available in GenBank. The APMV genome comprises of a single stranded, negative sense, non-segmented RNA comprising six transcriptional units (except APMV-6 with seven units) each coding for a structural protein. Additionally, by co-transcriptional RNA editing of phosphoprotein (P) gene, two mRNAs coding for accessory viral proteins, V and W, are generated along with unedited P mRNA. However, in APMV-11, the unedited mRNA codes for V protein while +2 edited mRNA translates to P protein, similar to members of subfamily Rubulavirinae in the same family. Such RNA editing in paramyxoviruses enables maximizing the coding capacity of their smaller genome. The three proteins of P gene: P, V and W, share identical N terminal but varied C terminal sequences that contribute to their unique functions. Here, we analyzed the P gene editing site, V and W sequences of all 21 APMV species known so far (55 viruses) by using bioinformatics and report their genetic variations and molecular evolution. The variations observed in the sequence and hexamer phase positions of the P gene editing sites is likely to influence the levels and relative proportions of P, V and W proteins’ expressions which could explain the differences in the pathogenicity of APMVs. The V protein sequences of APMVs had conserved motifs similar to V proteins of other paramyxoviruses including the seven cysteine residues involved in MDA5 interference, STAT1 degradation and interferon antagonism. Conversely, W protein sequences of APMVs were distinct. High sequence homology was observed in both V and W proteins between strains of the same species than between species except in APMV-3 which was the most divergent APMV species. The estimates of synonymous and non-synonymous substitution rates suggested negative selection pressure on the V and W proteins within species indicating their low evolution rate. The molecular clock analysis revealed higher conservation of V protein sequence compared to W protein indicating the important role played by V protein in viral replication, pathogenesis and immune evasion. However, we speculate the genetic diversity of W proteins could impact the degree of pathogenesis, variable interferon antagonistic activity and the wide host range exhibited by APMV species. Phylogenetically, V proteins of APMVs clustered into three groups similar to the recent classification of APMVs into three new genera while no such pattern could be deciphered in the analysis of W proteins except that strains of same species grouped together. This is the first comprehensive study describing in detail the genetic variations and the molecular evolution of P gene edited, accessory viral proteins of Avian paramyxoviruses. | NA | no | No good fitness measure and measure host GD | NA | no | no |
| 5515 | 2020 | 44113 | Rathmann, H; Reyes-Centeno, H; Ghirotto, S; Creanza, N; Hanihara, T; Harvati, K | 2017 | Reconstructing human population history from dental phenotypes | Dental phenotypic data are often used to reconstruct biological relatedness among past human groups. Teeth are an important data source because they are generally well preserved in the archaeological and fossil record, even when associated skeletal and DNA preservation is poor. Furthermore, tooth form is considered to be highly heritable and selectively neutral; thus, teeth are assumed to be an excellent proxy for neutral genetic data when none are available. However, to our knowledge, no study to date has systematically tested the assumption of genetic neutrality of dental morphological features on a global scale. Therefore, for the first time, this study quantifies the correlation of biological affinities between worldwide modern human populations, derived independently from dental phenotypes and neutral genetic markers. We show that population relationship measures based on dental morphology are significantly correlated with those based on neutral genetic data (on average r = 0.574, p < 0.001). This relatively strong correlation validates tooth form as a proxy for neutral genomic markers. Nonetheless, we suggest caution in reconstructions of population affinities based on dental data alone because only part of the dental morphological variation among populations can be explained in terms of neutral genetic differences. | NA | no | human | NA | no | no |
| 5516 | 2020 | 44042 | Raudsepp, T; Finno, CJ; Bellone, RR; Petersen, JL | 2019 | Ten years of the horse reference genome: insights into equine biology, domestication and population dynamics in the post-genome era | The horse reference genome from the Thoroughbred mare Twilight has been available for a decade and, together with advances in genomics technologies, has led to unparalleled developments in equine genomics. At the core of this progress is the continuing improvement of the quality, contiguity and completeness of the reference genome, and its functional annotation. Recent achievements include the release of the next version of the reference genome (EquCab3.0) and generation of a reference sequence for the Y chromosome. Horse satellite-free centromeres provide unique models for mammalian centromere research. Despite extremely low genetic diversity of the Y chromosome, it has been possible to trace patrilines of breeds and pedigrees and show that Y variation was lost in the past approximately 2300 years owing to selective breeding. The high-quality reference genome has led to the development of three different SNP arrays and WGSs of almost 2000 modern individual horses. The collection of WGS of hundreds of ancient horses is unique and not available for any other domestic species. These tools and resources have led to global population studies dissecting the natural history of the species and genetic makeup and ancestry of modern breeds. Most importantly, the available tools and resources, together with the discovery of functional elements, are dissecting molecular causes of a growing number of Mendelian and complex traits. The improved understanding of molecular underpinnings of various traits continues to benefit the health and performance of the horse whereas also serving as a model for complex disease across species. | NA | no | No good fitness measure and measure host GD | NA | no | no |
| 5517 | 2020 | 44111 | Ravenhall, M; Benavente, ED; Sutherland, CJ; Baker, DA; Campino, S; Clark, TG | 2019 | An analysis of large structural variation in global Plasmodium falciparum isolates identifies a novel duplication of the chloroquine resistance associated gene | The evolution of genetic mechanisms for host immune evasion and anti-malarial resistance has enabled the Plasmodium falciparum malaria parasite to inflict high morbidity and mortality on human populations. Most studies of P. falciparum genetic diversity have focused on single-nucleotide polymorphisms (SNPs), assisting the identification of drug resistance-associated loci such as the chloroquine related crt and sulfadoxine-pyrimethamine related dhfr. Whilst larger structural variants are known to impact adaptation, for example, mdr1 duplications with anti-malarial resistance, no largescale, genome-wide study on clinical isolates has been undertaken using whole genome sequencing data. By applying a structural variant detection pipeline across whole genome sequence data from 2,855 clinical isolates in 21 malaria-endemic countries, we identified > 70,000 specific deletions and > 600 duplications. Most structural variants are rare (48.5% of deletions and 94.7% of duplications are found in single isolates) with 2.4% of deletions and 0.2% of duplications found in > 5% of global isolates. A subset of variants was present at high frequency in drug-resistance related genes including mdr1, the gch1 promoter region, and a putative novel duplication of crt. Regional-specific variants were identified, and a companion visualisation tool has been developed to assist web-based investigation of these polymorphisms by the wider scientific community. | NA | no | gd of pathogen | NA | no | no |
| 5518 | 2020 | 44112 | Razo, CAP; Hernandez, ER; Ponce, SIR; Suazo, FM; Robbe-Austerman, S; Stuber, T; Alarcon, GJC | 2018 | Molecular epidemiology of cattle tuberculosis in Mexico through whole-genome sequencing and spoligotyping | Mycobacterium bovis infection in cattle persists in Mexico, posing a threat to human health. Control of bovine tuberculosis, through the National Program Against Bovine Tuberculosis, has led to the decrease of disease prevalence in most of the country, except for high dairy production regions. Genotyping of M. bovis has been performed mainly by spoligotyping and variable number tandem repeats (VNTR), but higher resolution power can be useful for a finer definition of the spread of the disease. Whole genome sequencing and spoligotyping was performed for a set of 322 M. bovis isolates from different sources in Mexico: Baja California, Coahuila, Estado de Mexico, Guanajuato, Hidalgo, Jalisco, Queretaro and Veracruz, from dairy and beef cattle, as well as humans. Twelve main genetic clades were obtained through WGS and genetic diversity analysis. A clear differentiation of the Baja California isolates was seen as they clustered together exclusively. However, isolates from the central states showed no specific clustering whatsoever. Although WGS proves to have higher resolving power than spoligotyping, and since there was concordance between WGS and spoligotyping results, we consider that the latter is still an efficient and practical method for monitoring bovine tuberculosis in developing countries, where resources for higher technology are scarce. | NA | no | gd of pathogen | NA | no | no |
| 5519 | 2020 | 44112 | Realpe-Quintero, M; Barba-Leon, J; Perez-Montano, JA; Pacheco-Gallardo, C; Gonzalez-Aguilar, D; Dominguez-Arias, RM; Cabrera-Diaz, E | 2018 | Genetic diversity and antimicrobial resistance of Salmonella serotypes recovered throughout the beef production chain and from patients with salmonellosis | Salmonella is one of the major foodborne pathogens worldwide. The antimicrobial resistance (AMR) of this foodborne pathogen has raised a great concern in recent years. Studies on the frequency and characterization of Salmonella serotypes can help to improve our knowledge on the epidemiology of this pathogen. The purpose of this study was to compare the serotypes, AMR and genetic profiles of Salmonella isolates recovered from raw beef throughout the beef production chain and from human feces associated with clinical cases of salmonellosis. The serotype, AMR and pulsed-field gel electrophoresis profile of 243 Salmonella enterica isolates recovered from beef carcasses (n=78), ground beef (n=135), and human feces from clinical cases of salmonellosis (n=30) were compared. Forty-three different Salmonella serotypes were identified and regardless of the source, the top five corresponded to Typhimurium, Give, Group B (partially serotyped), Infantis and Anatum. Twelve serotypes from beef carcasses were also found in ground beef, showing their presence throughout the beef production chain. Salmonella Typhimurium, Infantis, Anatum and Montevideo were the only serotypes identified in all sample types. Resistance to tetracyclines was the most frequent (41.2%) followed by resistance to aminoglycosides (37%), folate pathway inhibitors (21%), quinolones (20.2%), phenicols (17.1%), penicillins (15.6%) and cephems (7%). Multidrug resistance was observed in 28.8% of the isolates, and those from human feces showed resistance to a larger number of antimicrobials. Great concern arises from the resistance and reduced susceptibility observed to quinolones and cephalosporins because these drugs are the first line of treatment for invasive Salmonella infections. Twenty-seven distinct pulse-types were detected among 238 isolates. Clustering analysis for the most frequent serotypes identified groups of isolates with similar AMR profiles. Multidrug resistance spreading throughout the food production chain should be continually monitored and its importance emphasized. | NA | no | gd of pathogen | NA | no | no |
| 5520 | 2020 | 44112 | Rebaudet, S; Moore, S; Rossignol, E; Bogreau, H; Gaudart, J; Normand, AC; Laraque, MJ; Adrien, P; Boncy, J; Piarroux, R | 2019 | Epidemiological and molecular forensics of cholera recurrence in Haiti | Cholera has affected Haiti with damping waves of outbreaks since October 2010. However, mechanisms behind disease persistence during lull periods remain poorly understood. By mid 2014, cholera transmission seemed to only persist in the northern part of Haiti. Meanwhile, cholera appeared nearly extinct in the capital, Port-au-Prince, where it eventually exploded in September 2014. This study aimed to determine whether this outbreak was caused by local undetected cases or by re-importation of the disease from the north. Applying an integrated approach between November 2013 and November 2014, we assessed the temporal and spatial dynamics of cholera using routine surveillance data and performed population genetics analyses of 178 Vibrio cholerae O1 clinical isolates. The results suggest that the northern part of the country exhibited a persisting metapopulation pattern with roaming oligoclonal outbreaks that could not be effectively controlled. Conversely, undetected and unaddressed autochthonous low-grade transmission persisted in the Port-au-Prince area, which may have been the source of the acute outbreak in late-2014. Cholera genotyping is a simple but powerful tool to adapt control strategies based on epidemic specificities. In Haiti, these data have already yielded significant progress in cholera surveillance, which is a key component of the strategy to eventually eliminate cholera. | NA | no | No good fitness measure and measure host GD | NA | no | no |
| 5521 | 2020 | 44112 | Reher, D; Key, FM; Andres, AM; Kelso, J | 2019 | Immune Gene Diversity in Archaic and Present-day Humans | Genome-wide analyses of two Neandertals and a Denisovan have shown that these archaic humans had lower genetic heterozygosity than present-day people. A similar reduction in genetic diversity of protein-coding genes (gene diversity) was found in exome sequences of three Neandertals. Reduced gene diversity, particularly in genes involved in immunity, may have important functional consequences. In fact, it has been suggested that reduced diversity in immune genes may have contributed to Neandertal extinction. We therefore explored gene diversity in different human groups, and at different time points on the Neandertal lineage, with a particular focus on the diversity of genes involved in innate immunity and genes of the Major Histocompatibility Complex (MHC). We find that the two Neandertals and a Denisovan have similar gene diversity, all significantly lower than any present-day human. This is true across gene categories, with no gene set showing an excess decrease in diversity compared with the genome-wide average. Innate immune-related genes show a similar reduction in diversity to other genes, both in present-day and archaic humans. There is also no observable decrease in gene diversity over time in Neandertals, suggesting that there may have been no ongoing reduction in gene diversity in later Neandertals, although this needs confirmation with a larger sample size. In both archaic and present-day humans, genes with the highest levels of diversity are enriched for MHC-related functions. In fact, in archaic humans the MHC genes show evidence of having retained more diversity than genes involved only in the innate immune system. | NA | no | human | NA | no | no |
| 5522 | 2020 | 44112 | Reid-Anderson, S; Bilgmann, K; Stow, A | 2019 | Effective population size of the critically endangered east Australian grey nurse shark Carcharias taurus | Retaining genetic variation is central to species-level conservation, and knowledge of effective population sizes (N-e) can be used to predict rates at which genetic variation will be lost over generations. Here we used thousands of genome-wide single nucleotide polymorphisms to investigate N-e of the critically endangered grey nurse shark Carcharias taurus across 1400 km of its eastern Australian range. The eastern Australian grey nurse shark population has declined rapidly in number over the last few decades, has relatively low genetic diversity and is extremely susceptible to anthropogenic mortality. We found no evidence for any genetic structure, which is consistent with previous field observations that show widespread movement across the distribution. Estimates of the effective number of breeders (N-b) and N-e were around 400, using an approach based on proportions of siblings and another based on linkage disequilibrium. Forward simulations revealed that even if an N-e of 400 is maintained, the population will experience some loss of genetic diversity over the next 50 generations. The relatively low N-e highlights the importance of maintaining the population size of grey nurse sharks in order to retain genetic variation and therefore resilience to environmental change. | NA | no | No good fitness measure and measure host GD | NA | no | no |
| 5523 | 2020 | 44111 | Reid, CJ; DeMaere, MZ; Djordjevic, SP | 2019 | Australian porcine clonal complex 10 (CC10) Escherichia coli belong to multiple sublineages of a highly diverse global CC10 phylogeny | We recently identified clonal complex 10 (CC10) Escherichia coli as the predominant clonal group in two populations of healthy Australian food-production pigs. CC10 are highly successful, colonizing humans, food-production animals, fresh produce and environmental niches. Furthermore, E. coli within CC10 are frequently drug resistant and increasingly reported as human and animal extra-intestinal pathogens. In order to develop a high-resolution global phylogeny and determine the repertoire of antimicrobial-resistance genes, virulence-associated genes and plasmid types within this clonal group, we downloaded 228 publicly available CC10 short-read genome sequences for comparison with 20 porcine CC10 we have previously described. Core genome single nucleotide polymorphism phylogeny revealed a highly diverse global phylogeny consisting of multiple lineages that did not cluster by geography or source of the isolates. Australian porcine strains belonged to several of these divergent lineages, indicative that CC10 is present in these animals due to multiple colonization events. Differences in resistance gene and plasmid carriage between porcine strains and the global collection highlighted the role of lateral gene transfer in the evolution of CC10 strains. Virulence profiles typical of extra-intestinal pathogenic E. coli were present in both Australian porcine strains and the broader collection. As both the core phylogeny and accessory gene characteristics appeared unrelated to the geography or source of the isolates, it is likely that the global expansion of CC10 is not a recent event and may be associated with faecal carriage in humans. | NA | no | No good fitness measure and measure host GD | NA | no | no |
| 5524 | 2020 | 44112 | Reis-Cunha, JL; Baptista, RP; Rodrigues-Luiz, GF; Coqueiro-dos-Santos, A; Valdivia, HO; de Almeida, LV; Cardoso, MS; D’Avila, DA; Dias, FHC; Fujiwara, RT; Galvao, LMC; Chiari, E; Cerqueira, GC; Bartholomeu, DC | 2018 | Whole genome sequencing of Trypanosoma cruzi field isolates reveals extensive genomic variability and complex aneuploidy patterns within TcII DTU | Background: Trypanosoma cruzi, the etiologic agent of Chagas disease, is currently divided into six discrete typing units (DTUs), named TcI-TcVI. TcII is among the major DTUs enrolled in human infections in South America southern cone, where it is associated with severe cardiac and digestive symptoms. Despite the importance of TcII in Chagas disease epidemiology and pathology, so far, no genome-wide comparisons of the mitochondrial and nuclear genomes of TcII field isolates have been performed to track the variability and evolution of this DTU in endemic regions. Results: In the present work, we have sequenced and compared the whole nuclear and mitochondrial genomes of seven TcII strains isolated from chagasic patients from the central and northeastern regions of Minas Gerais, Brazil, revealing an extensive genetic variability within this DTU. A comparison of the phylogeny based on the nuclear or mitochondrial genomes revealed that the majority of branches were shared by both sequences. The subtle divergences in the branches are probably consequence of mitochondrial introgression events between TcII strains. Two T. cruzi strains isolated from patients living in the central region of Minas Gerais, S15 and S162a, were clustered in the nuclear and mitochondrial phylogeny analysis. These two strains were isolated from the other five by the Espinhaco Mountains, a geographic barrier that could have restricted the traffic of insect vectors during T. cruzi evolution in the Minas Gerais state. Finally, the presence of aneuploidies was evaluated, revealing that all seven TcII strains have a different pattern of chromosomal duplication/loss. Conclusions: Analysis of genomic variability and aneuploidies suggests that there is significant genomic variability within Minas Gerais TcII strains, which could be exploited by the parasite to allow rapid selection of favorable phenotypes. Also, the aneuploidy patterns vary among T. cruzi strains and does not correlate with the nuclear phylogeny, suggesting that chromosomal duplication/loss are recent and frequent events in the parasite evolution. | NA | no | gd of pathogen | NA | no | no |
| 5525 | 2020 | 44113 | Renan, S; Speyer, E; Ben-Nun, T; Ziv, A; Greenbaum, G; Templeton, AR; Bar-David, S; Bouskila, A | 2018 | Fission-fusion social structure of a reintroduced ungulate: Implications for conservation | In a reintroduced population, the social behavior of the species can strongly affect the long-term viability of the population through its effects on movement, information flow, disease spread and the population’s genetic variability. Therefore, information on the social behavior of a reintroduced population can contribute to conservation practices; however, its importance is often underestimated. The initial phase of the Asiatic wild ass’s (Equus hemionus) reintroduction in Israel has been considered a success, and the population is currently estimated at more than 250 individuals. However, the current social structure of the population remained unknown. We aimed to study this important population trait and to provide helpful information for efficient conservation and management protocols. The study was based on direct observations that were conducted over four consecutive years, and on the analyses of groups’ composition and female groups’ stability. Female groups accompanied by males constituted only 5% of the total 659 observations, males were observed to be mainly solitary or in groups of various sizes, and females were organized in non-stable groups, indicating that the reintroduced population exhibits a fission-fusion social structure. Identifying the social structure for the species in the expanding Negev population of the Asiatic wild ass can assist in implementing future reintroductions and can contribute to effective management decisions aimed at protecting the species. | NA | no | No good fitness measure and measure host GD | NA | no | no |
| 5526 | 2020 | 44040 | Repkin, EA; Maltseva, AL; Varfolomeeva, MA; Aianka, RV; Mikhailova, NA; Granovitch, AI | 2020 | Genetic and morphological variation of metacercariae of Microphallus piriformes (Trematoda, Microphallidae): Effects of paraxenia and geographic location | Host organism offers an environment for a parasite, and this environment is heterogenous within the host, variable among individual as well as between the hosts, and changing during the host’s lifetime. This heterogeneity may act as a prerequisite for parasite species divergence. Intraspecific variability related to a certain type of heterogeneity may indicate an initial stage of speciation, and thus poses an evolutionary importance. Here we analyzed genetic and morphologic variation of trematode metacercariae of Microphallus piriformes (Trematoda, Microphallidae). Genetic variability of trematodes was assessed from sequences of cytochrome c oxidase subunit 1 (COI) and internal transcribed spacer region (ITS-1). Morphological variation of metacercarial body shape was for the first time analyzed using geometric morphometrics. Parasites from the White Sea and the Barents Sea coasts demonstrated partial genetic divergence (according to COI sequence analysis) and had significantly different body shape. Neither genetic nor morphological variation of metacercariae was related to intermediate host species. We discuss possible causes of the observed genetic divergence of parasite populations in different geographic regions. | NA | no | No good fitness measure and measure host GD | NA | no | no |
| 5527 | 2020 | 44042 | Retel, C; Kowallik, V; Huang, WN; Werner, B; Kunzel, S; Becks, L; Feulner, PGD | 2019 | The feedback between selection and demography shapes genomic diversity during coevolution | Species interactions and coevolution are integral to ecological communities, but we lack empirical information on when and how these interactions generate and purge genetic diversity. Using genomic time series data from host-virus experiments, we found that coevolution occurs through consecutive selective sweeps in both species, with temporal consistency across replicates. Sweeps were accompanied by phenotypic change (resistance or infectivity increases) and expansions in population size. In the host, population expansion enabled rapid generation of genetic diversity in accordance with neutral processes. Viral molecular evolution was, in contrast, confined to few genes, all putative targets of selection. This study demonstrates that molecular evolution during species interactions is shaped by both eco-evolutionary feedback dynamics and interspecific differences in how genetic diversity is generated and maintained. | NA | no | No good fitness measure and measure host GD | NA | no | no |
| 5528 | 2020 | 44113 | Retshabile, G; Mlotshwa, BC; Williams, L; Mwesigwa, S; Mboowa, G; Huang, ZY; Rustagi, N; Swaminathan, S; Katagirya, E; Kyobe, S; Wayengera, M; Kisitu, GP; Kateete, DP; Wampande, EM; Maplanka, K; Kasvosve, I; Pettitt, ED; Matshaba, M; Nsangi, B; Marape, M; Tsimako-Johnstone, M; Brown, CW; Yu, FL; Kekitiinwa, A; Joloba, M; Mpoloka, SW; Mardon, G; Anabwani, G; Hanchard, NA | 2018 | Whole-Exome Sequencing Reveals Uncaptured Variation and Distinct Ancestry in the Southern African Population of Botswana | Large-scale, population-based genomic studies have provided a context for modern medical genetics. Among such studies, however, African populations have remained relatively underrepresented. The breadth of genetic diversity across the African continent argues for an exploration of local genomic context to facilitate burgeoning disease mapping studies in Africa. We sought to characterize genetic variation and to assess population substructure within a cohort of HIV-positive children from Botswana-a Southern African country that is regionally underrepresented in genomic databases. Using whole-exome sequencing data from 164 Batswana and comparisons with 150 similarly sequenced HIV-positive Ugandan children, we found that 13%-25% of variation observed among Batswana was not captured by public databases. Uncaptured variants were significantly enriched (p = 2.2 x 10(-16)) for coding variants with minor allele frequencies between 1% and 5% and included predicted-damaging non-synonymous variants. Among variants found in public databases, corresponding allele frequencies varied widely, with Botswana having significantly higher allele frequencies among rare (<1%) pathogenic and damaging variants. Batswana clustered with other Southern African populations, but distinctly from 1000 Genomes African populations, and had limited evidence for admixture with extra-continental ancestries. We also observed a surprising lack of genetic substructure in Botswana, despite multiple tribal ethnicities and language groups, alongside a higher degree of relatedness than purported founder populations from the 1000 Genomes project. Our observations reveal a complex, but distinct, ancestral history and genomic architecture among Batswana and suggest that disease mapping within similar Southern African populations will require a deeper repository of genetic variation and allelic dependencies than presently exists. | NA | no | human | NA | no | no |
| 5529 | 2020 | 44039 | Rhode, C; Badenhorst, R; Hull, KL; Greenwood, MP; Bester-van der Merwe, AE; Andere, AA; Picard, CJ; Richards, C | 2020 | Genetic and phenotypic consequences of early domestication in black soldier flies (Hermetia illucens) | The black soldier fly,Hermetia illucens, is an emerging biotechnological agent with its larvae being effective converters of organic waste into usable bio-products including protein and lipids. To date, most operations use unimproved commercial populations produced by mass rearing, without cognisance of specific breeding strategies. The genetic and phenotypic consequences of these commercial practices remain unknown and could have a significant impact on long-term population viability and productivity. The aim of this study was thus to assess the genetic and phenotypic changes during the early phases of colony establishment and domestication in the black soldier fly. An experimental colony was established from wild founder flies and a new microsatellite marker panel was developed to assess population genetic parameters along with the phenotypic characteristics of each generational cohort under captive breeding. The experimental colony was characterised by a small effective population size, subsequent loss of genetic diversity and rapid genetic and phenotypic differentiation between the generational cohorts. Ultimately, the population collapsed by the fifth generation, most likely owing to the adverse effect of inbreeding depression following the fixation of deleterious alleles. Species withr-selected life history characteristics (e.g. short life-span, high fecundity and low larval survival) are known to pose particular challenges for genetic management. The current study suggests that sufficient genetic and phenotypic variations exist in the wild population and that domestication and strain development could be achieved with careful population augmentation and selection during the early stages of colony establishment. | NA | no | only one group, no good gd or fitness measure | NA | no | no |
| 5530 | 2020 | 44113 | Riabovol, I; Riabovol, L; Diordiieva, I; Poltoretskyi, S; Lubchenko, A; Kononenko, L; Kryzhanovskiy, V | 2018 | Evaluation of resistance to diseases of soft winter wheat samples created by hybridization of ecologically and geographically remote forms | Genetic and breeding improvement of soft winter wheat varieties is one of the most effective methods of increasing yields, resistance against abiotic and biotic environment factors and energy-saving of wheat cultivation. Use of disease-resistant varieties is an economically effective and environmentally safe method of plant protection. The plant’s resistance is provided by the existing group of resistance genes, which are specific and act at the first determinant phase of plant and pathogen interaction. In wheat breeding the genes that provide varieties with stable resistance to disease regardless of genetic diversity of pathogens and weather conditions of cultivation are of greatest value. Such genes are often dominant mono-and oligogens. The objective of our research was to identify and select soft winter wheat samples resistant to main diseases and created by hybridization of ecologically and geographically remote forms in order to use them in breeding as donors of resistance. As a result of the research four samples of soft winter wheat 4075, 6151, 3872 and 6254 with complex resistance to diseases (resistance score 8-9) were selected. Created materials showed a significant advantage in resistance to powdery mildew, fusarium and septoria blight in comparison with standard variety Favoritka. Selected samples can be used as donors of resistance genes in the breeding process of high-yielding soft winter wheat varieties. | NA | no | dometicated host | NA | no | no |
| 5531 | 2020 | 44111 | Richardson, JL; Silveira, G; Medrano, IS; Arietta, AZ; Mariani, C; Pertile, AC; Pereira, TC; Childs, JE; Ko, AI; Costa, F; Caccone, A | 2019 | Significant Genetic Impacts Accompany an Urban Rat Control Campaign in Salvador, Brazil | Rats thrive in human-dominated landscapes and have expanded to a near global distribution. Norway rats (Rattus norvegicus) contaminate food, damage infrastructure, and are reservoirs for zoonotic pathogens that cause human diseases. To limit these negative impacts, entities around the world implement intervention and control strategies designed to quickly and drastically reduce the number of rats in a population. While the primary goal of these interventions is to reduce rat numbers and their detrimental activities, there are important, yet unexplored, population genetic implications for these rapid population declines. Here, we compare the population genetics of R. norvegicus before, immediately after, and several months following a rodenticide-based eradication campaign targeting rats in an urban slum of Salvador, Brazil. This slum has been the focus of long-term research designed to understand and reduce the risk of leptospirosis for people in this area. We also look for a clear source of rats contributing to population recovery, by either rebounding through breeding of local survivors, or by immigration/reinvasion of the site. We found evidence of severe genetic bottlenecks, with the effective population size dropping 85-91% after eradication, consistent with declines in population sizes. These rapid declines also led to a strong shift in the genetic structure of rats before and after the eradication campaign. Relatedness increased in two of the three study areas after eradication, suggesting reduced population sizes and an uneven impact of the campaign across colonies within the population. Lastly, dozens of low-frequency alleles (mean frequency of 0.037) observed before the campaign were undetected after the campaign, potentially lost from the population via drift or selection. We discuss the public health and ecological implications of these rapid genetic impacts of urban control efforts. Our data suggests that targeting the genetic viability of rat populations may be another important component for integrated pest management (IPM) strategies, designed to reduce urban rats. | NA | no | No good fitness measure and measure host GD | NA | no | no |
| 5532 | 2020 | 44111 | Riehl, C; Strong, MJ | 2019 | Social parasitism as an alternative reproductive tactic in a cooperatively breeding cuckoo | Cooperatively nesting birds are vulnerable to social parasites that lay their eggs in host nests but provide no parental care(1-4). Most previous research has focused on the co-evolutionary arms race between host defences and the parasites that attempt to circumvent them(5-9), but it remains unclear why females sometimes cooperate and sometimes parasitize, and how parasitic tactics arise in cooperative systems(10-12). Here we show that cooperative and parasitic reproductive strategies result in approximately equal fitness pay-offs in the greater ani (Crotophaga major), a long-lived tropical cuckoo, using an 11-year dataset and comprehensive genetic data that enable comparisons of the life-histories of individual females. We found that most females in the population nested cooperatively at the beginning of the breeding season; however, of those birds that had their first nests destroyed, a minority subsequently acted as reproductive parasites. The tendency to parasitize was highly repeatable, which indicates individual specialization. Across years, the fitness pay-offs of the two strategies were approximately equal: females who never parasitized (a ‘pure cooperative’ strategy) laid larger clutches and fledged more young from their own nests than did birds that both nested and parasitized (a ‘mixed’ strategy). Our results suggest that the success of parasites is constrained by reproductive trade-offs as well as by host defences, and illustrate how cooperative and parasitic tactics can coexist stably in the same population. | NA | no | No good fitness measure and measure host GD | NA | no | no |
| 5533 | 2020 | 44112 | Riesgo, A; Taboada, S; Perez-Portela, R; Melis, P; Xavier, JR; Blasco, G; Lopez-Legentil, S | 2019 | Genetic diversity, connectivity and gene flow along the distribution of the emblematic Atlanto-Mediterranean sponge Petrosia ficiformis (Haplosclerida, Demospongiae) | BackgroundKnowledge about the distribution of the genetic variation of marine species is fundamental to address species conservation and management strategies, especially in scenarios with mass mortalities. In the Mediterranean Sea, Petrosia ficiformis is one of the species most affected by temperature-related diseases. Our study aimed to assess its genetic structure, connectivity, and bottleneck signatures to understand its evolutionary history and to provide information to help design conservation strategies of sessile marine invertebrates.ResultsWe genotyped 280 individuals from 19 locations across the entire distribution range of P. ficiformis in the Atlanto-Mediterranean region at 10 microsatellite loci. High levels of inbreeding were detected in most locations (especially in the Macaronesia and the Western Mediterranean) and bottleneck signatures were only detected in Mediterranean populations, although not coinciding entirely with those with reported die-offs. We detected strong significant population differentiation, with the Atlantic populations being the most genetically isolated, and show that six clusters explained the genetic structure along the distribution range of this sponge. Although we detected a pattern of isolation by distance in P. ficiformis when all locations were analyzed together, stratified Mantel tests revealed that other factors could be playing a more prominent role than isolation by distance. Indeed, we detected a strong effect of oceanographic barriers impeding the gene flow among certain areas, the strongest one being the Almeria-Oran front, hampering gene flow between the Atlantic Ocean and the Mediterranean Sea. Finally, migration and genetic diversity distribution analyses suggest a Mediterranean origin for the species.ConclusionsIn our study Petrosia ficiformis showed extreme levels of inbreeding and population differentiation, which could all be linked to the poor swimming abilities of the larva. However, the observed moderate migration patterns are highly difficult to reconcile with such poor larval dispersal, and suggest that, although unlikely, dispersal may also be achieved in the gamete phase. Overall, because of the high genetic diversity in the Eastern Mediterranean and frequent mass mortalities in the Western Mediterranean, we suggest that conservation efforts should be carried out specifically in those areas of the Mediterranean to safeguard the genetic diversity of the species. | NA | no | No good fitness measure | NA | no | no |
| 5534 | 2020 | 44040 | Righi, T; Splendiani, A; Fioravanti, T; Casoni, E; Gioacchini, G; Carnevali, O; Barucchi, VC | 2020 | Loss of Mitochondrial Genetic Diversity in Overexploited Mediterranean Swordfish (Xiphias gladius, 1759) Population | Intense and prolonged mortality caused by over-exploitation could drive the decay of genetic diversity which may lead to decrease species’ resilience to environmental changes, thus increasing their extinction risk. Swordfish is a high commercial value species, especially in the Mediterranean Sea, where it is affected by high catch levels. Mediterranean swordfish consist of a population genetically and biologically distinct from Atlantic ones and therefore managed as a separate stock. The last Mediterranean swordfish stock assessment reported that in the last forty years Mediterranean swordfish has been overfished and, to date, it is still subject to overfishing. A comparison between an available mitochondrial sequence dataset and a homologous current sample was carried out to investigate temporal genetic variation in the Mediterranean swordfish population over near twenty years. Our study provides the first direct measure of reduced genetic diversity for Mediterranean swordfish during a short period, as measured both in the direct loss of mitochondrial haplotypes and reduction in haplotype diversity. A reduction of the relative females’ effective population size in the recent sample has been also detected. The possible relationship between fishery activities and the loss of genetic diversity in the Mediterranean swordfish population is discussed. | NA | no | No group level | NA | no | no |
| 5535 | 2020 | 44042 | Rivera-Leon, VE; Urban, J; Mizroch, S; Brownell, RL; Oosting, T; Hao, WS; Palsboll, PJ; Berube, M | 2019 | Long-term isolation at a low effective population size greatly reduced genetic diversity in Gulf of California fin whales | The Gulf of California, Mexico is home to many cetacean species, including a presumed resident population of fin whales, Balaenoptera physalus. Past studies reported very low levels of genetic diversity among Gulf of California fin whales and a significant level of genetic differentiation from con-specifics in the eastern North Pacific. The aim of the present study was to assess the degree and timing of the isolation of Gulf of California fin whales in a population genetic analysis of 18 nuclear microsatellite genotypes from 402 samples and 565 mitochondrial control region DNA sequences (including mitochondria) sequences retrieved from NCBI). The analyses revealed that the Gulf of California fin whale population was founded similar to 2.3 thousand years ago and has since remained at a low effective population size (similar to 360) and isolated from the eastern North Pacific (N(e)m between 0.89-1.4). The low effective population size and high degree of isolation implied that Gulf of California fin whales are vulnerable to the negative effects of genetic drift, human-caused mortality and habitat change. | NA | no | No good fitness measure and measure host GD | NA | no | no |
| 5536 | 2020 | 44111 | Roach, R; Mann, R; Gambley, CG; Chapman, T; Shivas, RG; Rodoni, B | 2019 | Genomic sequence analysis reveals diversity of Australian Xanthomonas species associated with bacterial leaf spot of tomato, capsicum and chilli | BackgroundThe genetic diversity in Australian populations of Xanthomonas species associated with bacterial leaf spot in tomato, capsicum and chilli were compared to worldwide bacterial populations. The aim of this study was to confirm the identities of these Australian Xanthomonas species and classify them in comparison to overseas isolates. Analysis of whole genome sequence allows for the investigation of bacterial population structure, pathogenicity and gene exchange, resulting in better management strategies and biosecurity.ResultsPhylogenetic analysis of the core genome alignments and SNP data grouped strains in distinct clades. Patterns observed in average nucleotide identity, pan genome structure, effector and carbohydrate active enzyme profiles reflected the whole genome phylogeny and highlight taxonomic issues in X. perforans and X. euvesicatoria. Circular sequences with similarity to previously characterised plasmids were identified, and plasmids of similar sizes were isolated. Potential false positive and false negative plasmid assemblies were discussed. Effector patterns that may influence virulence on host plant species were analysed in pathogenic and non-pathogenic xanthomonads.ConclusionsThe phylogeny presented here confirmed X. vesicatoria, X. arboricola, X. euvesicatoria and X. perforans and a clade of an uncharacterised Xanthomonas species shown to be genetically distinct from all other strains of this study. The taxonomic status of X. perforans and X. euvesicatoria as one species is discussed in relation to whole genome phylogeny and phenotypic traits. The patterns evident in enzyme and plasmid profiles indicate worldwide exchange of genetic material with the potential to introduce new virulence elements into local bacterial populations. | NA | no | gd of pathogen | NA | no | no |
| 5537 | 2020 | 44042 | Roberts, BR; Osborne, JL | 2019 | Testing the efficacy of a thermal camera as a search tool for locating wild bumble bee nests | Research into how bumble bee colonies respond to the stressors affecting their populations are currently studied in the laboratory using commercially reared Bombus terrestris colonies. Understanding how these stressors affect wild bumble bee colonies in the field would be a crucial step forward for the conservation of bumble bee species. Currently, visual cues are used to locate bumble bee nests, using human searchers looking for the worker nest traffic, but the limitations of this method mean that low numbers of nests are found and so a new method that looks to tackle these limitations is needed. Thermal cameras have been considered as a potential nest searching tool because they reduce the visual complexity of the environment by displaying a homogenized thermal landscape to the searcher. In this study, we compare the use of a thermal camera to human searches using two trials: (i) using inexperienced volunteers to search along the transect for a known bumble bee nest and (ii) using an experienced individual to search across a number of novel locations. We found thermal cameras are not a better nest detection technique than human searches, having low success rates across both trials. We discuss the limitations of thermal cameras as a technique and propose how the technology could be improved for future studies. | NA | no | No good fitness measure and measure host GD | NA | no | no |
| 5538 | 2020 | 44112 | Robinson, CV; de Leaniz, CG; James, J; Cable, J; Orozco-TerWengel, P; Consuegra, S | 2018 | Genetic diversity and parasite facilitated establishment of the invasive signal crayfish (Pacifastacus leniusculus) in Great Britain | Successful establishment of non-native species is strongly influenced, among other factors, by the genetic variation of founding populations, which can be enhanced by multiple introductions through admixture. Coexisting pathogens can also facilitate the establishment of non-native species by detrimentally impacting on the native fauna acting as novel weapons. The signal crayfish (Pacifastacus leniusculus) is a highly invasive species, which has caused mass declines of native crayfish in Europe through displacement and transmission of the oomycete Aphanomyces astaci (crayfish plague), which is typically lethal to native European crayfish. However, whether Aphanomyces astaci may have facilitated the invasion of the signal crayfish is not known. We estimated the genetic diversity at microsatellite DNA loci, effective population size, and potential origins of seven infected and noninfected signal crayfish populations in Europe and one founder population in North America. Approximate Bayesian computation analysis and population structuring suggested multiple host introductions from diverse source populations, as well as higher heterozygosity among infected than uninfected populations, which could reflect a fitness advantage. Low effective population size, moderate heterozygosity, and lack of isolation by distance suggest that some invasive signal crayfish populations may not be fully established or that their genetic diversity may have been reduced by eradication attempts. | NA | no | No good fitness measure | NA | no | no |
| 5539 | 2020 | 44112 | Rocha, JF; Martinez, R; Abuabara, Y | 2018 | Genetic diversity of three indigenous pig breeds in Colombia | Zungo (ZU), San Pedreno (SP) and Casco de Mula (CM) are Colombian indigenous pig breeds originated from European populations that are well adapted to tropical conditions. These breeds have a great potential to be used in pure and crossbred schemes due to their high reproductive performance and resistance to tropical diseases. We investigated the genetic diversity and genetic structure of these pig breeds using microsatellite molecular markers. Fifty-five ZU, SP and CM animals were genotyped for 10 microsatellites using capillary electrophoresis. The average number of alleles per marker ranged from 5.56 +/- 1.88 in ZU to 6.70 +/- 1.64 in SP. Observed heterozygosity ranged from 0.68 +/- 0.05 in ZU to 0.74 +/- 0.03 in SP. Polymorphic information content was high for most of the microsatellites in all breeds. Inbreeding coefficient (F-IS) values were low for all microsatellites, with an average of 0.035 +/- 0.037. Higher average values closer to and above 0.1 were observed for the fixation index of the global population (F-IT) (0.131 +/- 0.041) and the coefficient of relatedness between individuals from different populations (F-ST) (0.099 +/- 0.013). Principal components and structure analyses showed a high level of clustering per breed and very low admixture levels between them. The panel of markers used in this study proved to be useful to investigate genetic diversity and pedigree relationships in pig populations. The Colombian pig breeds that we evaluated had a high genetic variability and a well-differentiated genetic structure, which are key for decision making in conservation programs and for the implementation of future animal breeding plans in these populations. | NA | no | No good fitness measure | NA | no | no |
| 5540 | 2020 | 44113 | Rodger, YS; Greenbaum, G; Silver, M; Bar-David, S; Winters, G | 2018 | Detecting hierarchical levels of connectivity in a population of Acacia tortilis at the northern edge of the species’ global distribution: Combining classical population genetics and network analyses | Genetic diversity and structure of populations at the edge of the species’ spatial distribution are important for potential adaptation to environmental changes and consequently, for the long-term survival of the species. Here, we combined classical population genetic methods with newly developed network analyses to gain complementary insights into the genetic structure and diversity of Acacia tortilis, a keystone desert tree, at the northern edge of its global distribution, where the population is under threat from climatic, ecological, and anthropogenic changes. We sampled A. tortilis from 14 sites along the Dead Sea region and the Arava Valley in Israel and in Jordan. In addition, we obtained samples from Egypt and Sudan, the hypothesized origin of the species. Samples from all sites were genotyped using six polymorphic microsatellite loci. Our results indicate a significant genetic structure in A. tortilis along the Arava Valley. This was detected at different hierarchical levels from the basic unit of the subpopulation, corresponding to groups of trees within ephemeral rivers (wadis), to groups of subpopulations (communities) that are genetically more connected relative to others. The latter structure mostly corresponds to the partition of the major drainage basins in the area. Network analyses, combined with classical methods, allowed for the identification of key A. tortilis subpopulations in this region, characterized by their relatively high level of genetic diversity and centrality in maintaining gene flow in the population. Characterizing such key subpopulations may enable conservation managers to focus their efforts on certain subpopulations that might be particularly important for the population’s long-term persistence, thus contributing to species conservation within its peripheral range. | NA | no | No good fitness measure | NA | no | no |
| 5541 | 2020 | 44113 | Rodrigues, PT; Valdivia, HO; de Oliveira, TC; Alves, JMP; Duarte, AMRC; Cerutti, C; Buery, JC; Brito, CFA; de Souza, JC; Hirano, ZMB; Bueno, MG; Catao-Dias, JL; Malafronte, RS; Ladeia-Andrade, S; Mita, T; Santamaria, AM; Calzada, JE; Tantular, IS; Kawamoto, F; Raijmakers, LRJ; Mueller, I; Pacheco, MA; Escalante, AA; Felger, I; Ferreira, MU | 2018 | Human migration and the spread of malaria parasites to the New World | We examined the mitogenomes of a large global collection of human malaria parasites to explore how and when Plasmodium falciparum and P. vivax entered the Americas. We found evidence of a significant contribution of African and South Asian lineages to present-day New World malaria parasites with additional P. vivax lineages appearing to originate from Melanesia that were putatively carried by the Australasian peoples who contributed genes to Native Americans. Importantly, mitochondrial lineages of the P. vivax-like species P. simium are shared by platyrrhine monkeys and humans in the Atlantic Forest ecosystem, but not across the Amazon, which most likely resulted from one or a few recent human-to-monkey transfers. While enslaved Africans were likely the main carriers of P. falciparum mitochondrial lineages into the Americas after the conquest, additional parasites carried by Australasian peoples in pre-Columbian times may have contributed to the extensive diversity of extant local populations of P. vivax. | NA | no | human | NA | no | no |
| 5542 | 2020 | 44113 | Rodriguez-Nevado, C; Lam, TTY; Holmes, EC; Pagan, I | 2018 | The impact of host genetic diversity on virus evolution and emergence | Accumulating evidence indicates that biodiversity has an important impact on parasite evolution and emergence. The vast majority of studies in this area have only considered the diversity of species within an environment as an overall measure of biodiversity, overlooking the role of genetic diversity within a particular host species. Although theoretical models propose that host genetic diversity in part shapes that of the infecting parasite population, and hence modulates the risk of parasite emergence, this effect has seldom been tested empirically. Using Rabies virus (RABV) as a model parasite, we provide evidence that greater host genetic diversity increases both parasite genetic diversity and the likelihood of a host being a donor in RABV cross-species transmission events. We conclude that host genetic diversity may be an important determinant of parasite evolution and emergence. | NA | no | No good fitness measure and measure host GD | NA | no | no |
| 5543 | 2020 | 44042 | Rolim, GG; Arruda, LS; Torres, JB; Barros, EM; Fernandes, MG | 2019 | Susceptibility of Cotton Boll Weevil (Coleoptera: Curculionidae) to Spinosyns | The control of boll weevil, Anthonomus grandis grandis Boh., relies primarily on synthetic insecticides. Although insecticides are registered to spray cotton fields against boll weevils, only a few offer satisfactory control and most have broad-spectrum action. Alternatively, spinosyns have been recommended against lepidopteran pest species in cotton and are considered comparatively of reduced risk to nontargets. The susceptibility of nine populations of boll weevil to spinosad and spinetoram was determined through dried residue on squares and cotton leaves. Furthermore, control efficacy of spinosad and spinetoram compared with the standard organophosphate malathion was determined for caged adults at two different positions within the plant canopy or treated cotton leaves after different spray intervals. Boll weevil susceptibility varied across the nine populations and tested spinosyns. The lethal concentrations (LC(50)s and LC(90)s) varied from 8.62 to 32.25 and 49.86 to 281.70 mg a.i./l for spinosad and from 2.17 to 15.36 and 8.58 to 69.36 mg a.i./l for spinetoram. The location of boll weevil within the plant canopy affected the insecticide efficacy with higher mortality when caged on upper parts rather than on lower parts of cotton plants (>85% vs <45% of control) across all three insecticides. In addition, dried residues of spinosyns resulted in adult mortality >80% until the end of the evaluation period (8 d), while the standard malathion caused only 10% at this last evaluation time. Thus, we can conclude that both spinosyns promoted prolonged adult mortality using recommended field rates compared with the standard malathion. | NA | no | No good fitness measure and measure host GD | NA | no | no |
| 5544 | 2020 | 44113 | Romba, R; Gnankine, O; Drabo, SF; Tiendrebeogo, F; Henri, H; Mouton, L; Vavre, F | 2018 | Abundance of Bemisia tabaci Gennadius (Hemiptera: Aleyrodidae) and its parasitoids on vegetables and cassava plants in Burkina Faso (West Africa) | The whitefly Bemisia tabaci is a pest of many agricultural and ornamental crops worldwide and particularly in Africa. It is a complex of cryptic species, which is extremely polyphagous with hundreds of host plants identified around the world. Previous surveys in western Africa indicated the presence of two biotypes of the invasive MED species (MED-Q1 and MED-Q3) living in sympatry with the African species SSA and ASL. This situation constitutes one of the rare cases of local coexistence of various genetic entities within the B.tabaci complex. In order to study the dynamics of the distribution and abundance of genetic entities within this community and to identify potential factors that could contribute to coexistence, we sampled B.tabaci populations in Burkina Faso in 2015 and 2016 on various plants, and also their parasitoids. All four genetic entities were still recorded, indicating no exclusion of local species by the MED species. While B.tabaci individuals were found on 55 plant species belonging to eighteen (18) families showing the high polyphagy of this pest, some species/biotypes exhibited higher specificity. Two parasitoid species (Eretmocerus mundus and Encarsia vandrieschei) were also recorded with E.mundus being predominant in most localities and on most plants. Our data indicated that whitefly abundance, diversity, and rate of parasitism varied according to areas, plants, and years, but that parasitism rate was globally highly correlated with whitefly abundance suggesting density dependence. Our results also suggest dynamic variation in the local diversity of B.tabaci species/biotypes from 1year to the other, specifically with MED-Q1 and ASL species. This work provides relevant information on the nature of plant-B.tabaci-parasitoid interactions in West Africa and identifies that coexistence might be stabilized by niche differentiation for some genetic entities. However, MED-Q1 and ASL show extensive niche overlap, which could ultimately lead to competitive exclusion. | NA | no | gd of pathogen | NA | no | no |
| 5545 | 2020 | 44113 | Romero-Diaz, C; Breedveld, MC; Fitze, PS | 2017 | Climate Effects on Growth, Body Condition, and Survival Depend on the Genetic Characteristics of the Population | Climatic change is expected to affect individual life histories and population dynamics, potentially increasing vulnerability to extinction. The importance of genetic diversity has been highlighted for adaptation and population persistence. However, whether responses of life-history traits to a given environmental condition depend on the genetic characteristics of a population remains elusive. Here we tested this hypothesis in the lizard Zootoca vivipara by simultaneously manipulating habitat humidity, a major climatic predictor of Zootoca’s distribution, and adult male color morph frequency, a trait with genome-wide linkage. Interactive effects of humidity and morph frequency had immediate effects on growth and body condition of juveniles and yearlings, as well as on adult survival, and delayed effects on offspring size. In yearlings, higher humidity led to larger female body size and lower humidity led to higher male compared to female survival. In juveniles and yearlings, some treatment effects were compensated over time. The results show that individual responses to environmental conditions depend on the population’s color morph frequency, age class, and sex and that these affect intra- and inter-age class competition. Moreover, humidity affected the competitive environment rather than imposing trait-based selection on specific color morphs. This indicates that species’ responses to changing environments (e.g., to climate change) are highly complex and difficult to accurately reconstruct and predict without information on the genetic characteristics and demographic structure of populations. | NA | no | No good fitness measure and measure host GD | NA | no | no |
| 5546 | 2020 | 44113 | Ropars, J; Maufrais, C; Diogo, D; Marcet-Houben, M; Perin, A; Sertour, N; Mosca, K; Permal, E; Laval, G; Bouchier, C; Ma, L; Schwartz, K; Voelz, K; May, RC; Poulain, J; Battail, C; Wincker, P; Borman, AM; Chowdhary, A; Fan, S; Kim, SH; Le Pape, P; Romeo, O; Shin, JH; Gabaldon, T; Sherlock, G; Bougnoux, ME; d’Enfert, C | 2018 | Gene flow contributes to diversification of the major fungal pathogen Candida albicans | Elucidating population structure and levels of genetic diversity and recombination is necessary to understand the evolution and adaptation of species. Candida albicans is the second most frequent agent of human fungal infections worldwide, causing high-mortality rates. Here we present the genomic sequences of 182 C. albicans isolates collected worldwide, including commensal isolates, as well as ones responsible for superficial and invasive infections, constituting the largest dataset to date for this major fungal pathogen. Although, C. albicans shows a predominantly clonal population structure, we find evidence of gene flow between previously known and newly identified genetic clusters, supporting the occurrence of (para) sexuality in nature. A highly clonal lineage, which experimentally shows reduced fitness, has undergone pseudogenization in genes required for virulence and morphogenesis, which may explain its niche restriction. Candida albicans thus takes advantage of both clonality and gene flow to diversify. | NA | no | No good fitness measure and measure host GD | NA | no | no |
| 5547 | 2020 | 44113 | Roques, S; Berrebi, P; Rochard, E; Acolas, ML | 2018 | Genetic monitoring for the successful re-stocking of a critically endangered diadromous fish with low diversity | The success of conservation programs for genetically depleted species is challenging because survival and adaptive potential are thought to be promoted by genetic factors. Such programs are thus generally designed to minimize the risks associated with the loss of genetic diversity and inbreeding. However, few empirical studies have explored the outcomes of these strategies in released individuals once they are in the wild. The only remaining population of the critically endangered European sturgeon, Acipenser sturio, is located in the Gironde estuary (France) after continued demographic decline over the last century. A restoration program that was initiated 20 years ago has resulted in more than. one and a half million individuals being stocked in the last decade. Because the captive broodstock is composed of a small number of breeders (N = 78 in 2007, which decreased to N = 21 in early 2017), maintaining genetic diversity and minimizing inbreeding are crucial for the successful recovery of this species. Based on the analysis of 18 microsatellite markers, we examined the patterns of genetic diversity of fish from the last wild cohorts and broodstock as well as of captive-born fish recaptured after stocking. Our results support the hypothesis of a genetic bottleneck with low genetic diversity and a highly reduced effective population size in wild-born A. sturio. While the original diversity is depleted, clearly challenging conservation strategies, we found that genetic erosion has been limited during re-stocking (96% of the broodstock and 90% of the wild fish original diversity has been maintained in recaptured individuals). Results further suggest that the genetics of the parents is not the main factor explaining recapture probability: progeny issued from both related and not related parent pairs were found in recaptures years after their release. For broodstock management, kinship between parents is identified as a relevant parameter to be considered to avoid inbreeding. This study suggests that a multi-year stocking strategy associated with sufficiently high numbers of unrelated parent pairs may be the best strategy for the successful reintroduction and preservation of genetic diversity for A. sturio. These findings are encouraging for the species and further highlights the relevance of careful genetic monitoring for the long-term conservation of genetically depleted species, which are more vulnerable to genetic effects. | NA | no | No good fitness measure and measure host GD | NA | no | no |
| 5548 | 2020 | 44040 | Rose, C; Prange, IS; Landry, SM | 2020 | Extirpated, immigrated, genetically stratified-first demographic assessment of a recovering bobcat (Lynx rufus) population after a century of extinction | The bobcat (Lynx rufus) is a widespread mesocarnivore that declined in the USA as immigration and settlement proceeded. It was considered extirpated in Ohio (OH) around 1850, and generally extinct for a century. Listed as state-endangered in 1974, verified reports increased in the 1990s. Population clusters developed in 2 areas of OH, and genetically stratified. The eastern population increased more rapidly, and the southern was part of a multistate population including West Virginia (WV) and Kentucky (KY). To investigate the recovering bobcat’s population ecology, the State of OH collected carcasses of bobcats who died mostly from vehicle collision while state-listed. We processed specimens for demographic data, and supplemented the southern sample with western WV and eastern KY harvest data given genetic relatedness. We applied a vertical life table framework interpreting our population cross-section as a recovery period. We estimated age structure, fecundity, and population status of eastern OH bobcats, and compared fecundity between areas. Life-table analyses indicated an eastern OH population hovering around zero growth. Juvenile females contained most reproductive value, and most males died as young adults. Fecundity in eastern OH surpassed that of the multistate population overall where comparable. Fecundity increased through several years of life as bobcats matured. Our results suggest, after a rapid recovery of eastern OH bobcats, population growth slowed as they began to saturate their environment. OH delisted bobcats in 2014 and proposed a harvest season in 2018. With loosening regulation, we recommend more robust sampling for improved population modeling after expansion of the recovered population’s range. | NA | no | No good fitness measure and measure host GD | NA | no | no |
| 5549 | 2020 | 44040 | Rossanti, R; Morisada, N; Nozu, K; Kamei, K; Horinouchi, T; Yamamura, T; Minamikawa, S; Fujimura, J; Nagano, C; Sakakibara, N; Ninchoji, T; Kaito, H; Ito, S; Tanaka, R; Iijima, K | 2020 | Clinical and genetic variability of PAX2-related disorder in the Japanese population | Pathogenic variants of paired box gene 2 (PAX2) cause autosomal-dominant PAX2-related disorder, which includes renal coloboma syndrome (RCS). Patients with PAX2-related disorder present with renal and ophthalmological pathologies, as well as with other abnormalities, including developmental problems and hearing loss. We sequenced PAX2 in 457 patients with congenital anomalies of the kidney and urinary tract or with renal dysfunction of unknown cause and identified 19 different pathogenic variants in 38 patients from 30 families (6.5%). Thirty-four patients had renal hypodysplasia or chronic kidney disease of unknown cause, and three had focal segmental glomerulosclerosis. Although no obvious genotype-phenotype correlation was observed, six of the seven patients who developed end-stage renal disease in childhood had truncating variants. Twenty-three patients had ocular disabilities, mostly optic disc coloboma. Non-renal and non-ophthalmological manifestations included developmental disorder, electrolyte abnormality, and gonadal abnormalities. Two unrelated patients had congenital cystic adenomatoid malformations in their lungs. Six of ten probands with PAX2 mutation identified by next-generation sequencing did not show typical RCS manifestations. We conclude that PAX2-related disorder has a variable clinical presentation and can be diagnosed by next-generation sequencing even in the absence of typical RCS manifestations. | NA | no | human | NA | no | no |
| 5550 | 2020 | 44112 | Rottiers, H; Everaert, H; Boeckx, P; Limba, G; Baert, G; De Wever, J; Maebe, K; Smagghe, G; Dewettinck, K; Messens, K | 2018 | Unraveling the genetic background of the Yangambi Research Center cacao germplasm collection, DR Congo | The Democratic Republic of the Congo (DR Congo or DRC) has some of the most fertile soils in Africa to cultivate cacao, an important cash crop and source of income for many smallholder farmers. Although cacao is currently produced there on small scale, DRC has the potential to grow as a cacao-producing country, thereby supplying the increasing cacao demand in the global market. Since the introduction of cacao varieties in the late nineteenth century, selection and breeding experiments have been carried out based on phenotype, without any knowledge on the genetic background of the cultivars. Therefore, this study analyzes 62 Congolese accessions of the Centre de Recherche de Yangambi (CRY) and 51 accessions of international collections representing 10 reference groups and Trinitario cultivars using 14 microsatellite markers. Descriptive statistics revealed a high gene diversity and polymorphic information content (PIC) for most of the markers, of which mTcCIR 12, 37, and 60 were the most discriminative. Both Bayesian clustering and principal coordinate analysis (PCoA) revealed high-admixed ancestry of the CRY cultivars. The collection was divided in two clusters, of which the first formed a hybrid population, linked to Amelonado, Trinitario, MaraIOEn, and Nanay, and the second was assigned to predominantly Amelonado, followed by Nanay, Contamana, and Nacional. This high admixture level resulted from numerous hybridization and recombination events that took place in the previous century. The obtained knowledge is essential for efficient conservation, utilization, and selection of high-quality cacao cultivars, which are agronomical favored, disease resistant, and of superior flavor quality. | NA | no | No good fitness measure and measure host GD | NA | no | no |
| 5551 | 2020 | 44112 | Rowntree, JK; Craig, H | 2019 | The contrasting roles of host species diversity and parasite population genetic diversity in the infection dynamics of a keystone parasitic plant | 1. Diversity among species and genetic diversity within species are both important components of ecological communities that can determine the outcome of species interactions, especially between hosts and parasites. We sought to understand the impact of species diversity on host community resistance to infection by a keystone parasitic plant (Rhinanthus minor L.) and genetic diversity of the parasite on its successful establishment in a grassland community. 2. We used an experimental approach where large pots were planted with mixtures of mesotrophic grassland species at high and low species diversity. The parasitic plant was sown in a proportion of these with high and low genetic diversity treatments. Establishment of the parasite was monitored over 2 years and the pots harvested at the end of each growing season to determine the impact of infection on plant community biomass. 3. We found a strong effect of host plant species diversity on the establishment of the parasitic plant, with successful establishment considerably lower in the high species diversity treatment. Genetic diversity appeared to promote establishment of the parasite in the high species diversity treatment, and also facilitated longer term fitness in the low species diversity treatment. Host community structure was influenced by R. minor, with grass relative biomass decreasing and legume relative biomass increasing when the parasite was present. There was no direct impact of the presence of the parasite on the relative biomass of nonleguminous forbs. 4. Synthesis. Our data demonstrate the importance of host community species diversity in deterring the establishment of a generalist parasite. They also highlight the role of genetic diversity in determining the outcome of host-parasite interactions in multispecies communities. These findings, therefore, have important implications for the establishment and management of species-rich grasslands and provide insight into the community dynamics of parasitic plants and their hosts. | NA | no | no good gd measure host pop | NA | no | no |
| 5552 | 2020 | 44113 | Rubio-Mendez, G; Castillo-Gomez, HA; Hernandez-Sandoval, L; Espinosa-Reyes, G; De-Nova, JA | 2018 | Chronic Disturbance Affects the Demography and Population Structure of Beaucarnea inermis, a Threatened Species Endemic to Mexico | Beaucarnea inermis is a threatened plant of the seasonally dry tropical forests of the northeastern Sierra Madre Oriental mountains. It has been affected by habitat loss and fragmentation, mainly from changes in land use and poaching. The number of B. inermis plants, sexual proportion, and structural parameters were recorded in natural populations inside the Sierra del Abra Tanchipa Biosphere Reserve and unprotected sites. Effects of chronic disturbance on demography parameters and asymmetry coefficient were estimated. Average population density is 280 ind*ha(-1) inside the protected area and 186 ind*ha(-l) in unprotected sites. Life tables indicate a high seedling to juvenile mortality rate, but life expectancy increases in juveniles, suggesting a survival type III curve. Population size distribution skewness indicates differences associated with disturbance. Sex ratio was 0.93:1 (m:f) inside the protected area and 0.76:1 (m:f) in unprotected sites. Anthropogenic disturbance affects life expectancy and the survival and mortality rates mainly in early life classes; however, once the adult stage is reached, mortality rate is reduced and survival rate increases. The natural protected area represents a refuge for the species from the effects of anthropogenic disturbance and illegal poaching. Additional studies are needed to evaluate the genetic diversity in B. inermis in protected and unprotected sites, and how it is affected by disturbance. Also, it is important to highlight other species inside the protected area such as Dioon edule, Zamia fischeri, and Stanhopea tigrina, which are considered as endangered or threatened. | NA | no | No good fitness measure and measure host GD | NA | no | no |
| 5553 | 2020 | 44042 | Rubio, AV; Fredes, F; Simonetti, JA | 2019 | Exotic Pinus radiata Plantations do not Increase Andes Hantavirus Prevalence in Rodents | Andes south virus (ANDV) is the etiologic agent of hantavirus cardiopulmonary syndrome (HCPS) in Chile and southern Argentina. Farm and forestry workers have been identified as a group at high risk of acquiring HCPS caused by ANDV due to their close exposure to rodents or their secretions in rural areas. Therefore, investigation on the effect of landscape composition on ANDV in wild rodents becomes relevant for disease prevention and control. In this study, we analyzed the influence of Monterey pine (Pinus radiata) plantations, an important monoculture in the global forest industry, on small mammal assemblage and on ANDV seroprevalence and abundance of seropositive rodents from central Chile. Small mammals were sampled seasonally during 2 years in native forests, adult pine plantations and young pine plantations. A total of 1630 samples from seven rodent species were analyzed for antibody detection. ANDV seroprevalence and abundance of seropositive rodents were significantly higher in the native forest compared to pine plantations. Furthermore, Monterey pine plantations decrease the abundance and relative abundance of Oligoryzomys longicaudatus (the principal reservoir of ANDV) and do not change sex ratio and distribution of age classes of this rodent species, which are variables that are important for ANDV transmission. Our findings indicate that Monterey pine plantations would not pose a higher risk of human exposure to ANDV compared to the temperate native forest. Our results can be useful for hantavirus risks assessment in human-dominated areas where ANDV is endemic. | NA | no | gd of pathogen | NA | no | no |
| 5554 | 2020 | 44042 | Russell, RE; Tripp, DW; Rocke, TE | 2019 | Differential plague susceptibility in species and populations of prairie dogs | Laboratory trials conducted over the past decade at U.S. Geological Survey National Wildlife Health Center indicate that wild populations of prairie dogs (Cynomys spp.) display different degrees of susceptibility to experimental challenge with fully virulent Yersinia pestis, the causative agent of plague. We evaluated patterns in prairie dog susceptibility to plague to determine whether the historical occurrence of plague at location of capture was related to survival times of prairie dogs challenged with Y. pestis. We found that black-tailed prairie dogs (Cynomys ludovicianus) from South Dakota (captured prior to the detection of plague in the state), Gunnison’s prairie dogs (Cynomys gunnisoni) from Colorado, and Utah prairie dogs (Cynomys parvidens) from Utah were most susceptible to plague. Though the susceptibility of black-tailed prairie dogs in South Dakota compared with western locations supports our hypothesis regarding historical exposure, both Colorado and Utah prairie dogs have a long history of exposure to plague. It is possible that for these populations, genetic isolation/bottle necks have made them more susceptible to plague outbreaks. | NA | no | no good gd measure host | NA | no | no |
| 5555 | 2020 | 44042 | Russell, T; Lane, A; Clarke, J; Hogg, C; Morris, K; Keeley, T; Madsen, T; Ujvari, B | 2019 | Multiple paternity and precocial breeding in wild Tasmanian devils, Sarcophilus harrisii (Marsupialia: Dasyuridae) | Polyandry, a common reproductive strategy in various animal species, has potential female benefits, which include enhanced offspring fitness. Benefits can be direct, such as reduced risk of male infanticide of offspring, or indirect, such as increased genetic diversity of offspring and the acquisition of good genes’. Multiple paternity of litters has been recorded in numerous marsupial species but has not been reported in Tasmanian devils, Sarcophilus harrisii (Boitard). We investigated whether multiple paternity occurred in litters within a wild population of Tasmanian devils. Using major histocompatibility complex-linked and neutral microsatellite markers, the paternity of nine litters was analysed. We found multiple paternity in four out of nine litters and that yearling (> 1, < 2 years old) male devils were siring offspring. This is the first record of multiple paternity and of male precocial breeding in wild Tasmanian devils. To date, there are no data relating to the subsequent survival of devils from single- vs. multiple-sired litters; therefore, we do not know whether multiple paternity increases offspring survival in the wild. These results have implications for the Tasmanian devil captive insurance programme, because group housing can lead to multiple-sired litters, making the maintenance of genetic diversity over time difficult to manage. | NA | no | No good fitness measure | NA | no | no |
| 5556 | 2020 | 44113 | Russell, T; Lisovski, S; Olsson, M; Brown, G; Spindler, R; Lane, A; Keeley, T; Hibbard, C; Hogg, CJ; Thomas, F; Belov, K; Ujvari, B; Madsen, T | 2018 | MHC diversity and female age underpin reproductive success in an Australian icon; the Tasmanian Devil | Devil Facial Tumour Disease (DFTD), a highly contagious cancer, has decimated Tasmanian devil (Sarcophilus harrisii) numbers in the wild. To ensure its long-term survival, a captive breeding program was implemented but has not been as successful as envisaged at its launch in 2005. We therefore investigated the reproductive success of 65 captive devil pair combinations, of which 35 produced offspring (successful pairs) whereas the remaining 30 pairs, despite being observed mating, produced no offspring (unsuccessful pairs). The devils were screened at six MHC Class I-linked microsatellite loci. Our analyses revealed that younger females had a higher probability of being successful than older females. In the successful pairs we also observed a higher difference in total number of heterozygous loci, i.e. when one devil had a high total number of heterozygous loci, its partner had low numbers. Our results therefore suggest that devil reproductive success is subject to disruptive MHC selection, which to our knowledge has never been recorded in any vertebrate. In order to enhance the success of the captive breeding program the results from the present study show the importance of using young (2-year old) females as well as subjecting the devils to MHC genotyping. | NA | no | mhc diversity | NA | no | no |
| 5557 | 2020 | 44111 | Rutaihwa, LK; Menardo, F; Stucki, D; Gygli, SM; Ley, SD; Malla, B; Feldmann, J; Borrell, S; Beisel, C; Middelkoop, K; Carter, EJ; Diero, L; Ballif, M; Jugheli, L; Reitheru, K; Fenner, L; Brites, D; Gagneux, S | 2019 | Multiple Introductions of Mycobacterium tuberculosis Lineage 2-Beijing Into Africa Over Centuries | The Lineage 2-Beijing (L2-Beijing) sub-lineage of Mycobacterium tuberculosis has received much attention due to its high virulence, fast disease progression, and association with antibiotic resistance. Despite several reports of the recent emergence of L2-Beijing in Africa, no study has investigated the evolutionary history of this sub-lineage on the continent. In this study, we used whole genome sequences of 781 L2 clinical strains from 14 geographical regions globally distributed to investigate the origins and onward spread of this lineage in Africa. Our results reveal multiple introductions of L2-Beijing into Africa linked to independent bacterial populations from East- and Southeast Asia. Bayesian analyses further indicate that these introductions occurred during the past 300 years, with most of these events pre-dating the antibiotic era. Hence, the success of L2-Beijing in Africa is most likely due to its hypervirulence and high transmissibility rather than drug resistance. | NA | no | gd of pathogen | NA | no | no |
| 5558 | 2020 | 44111 | Rutaihwa, LK; Sasamalo, M; Jaleco, A; Hella, J; Kingazi, A; Kamwela, L; Kingalu, A; Malewo, B; Shirima, R; Doetsch, A; Feldmann, J; Reinhard, M; Borrell, S; Brites, D; Reither, K; Doulla, B; Fenner, L; Gagneux, S | 2019 | Insights into the genetic diversity of Mycobacterium tuberculosis in Tanzania | Background Human tuberculosis (TB) is caused by seven phylogenetic lineages of the Mycobacterium tuberculosis complex (MTBC), Lineage 1-7. Recent advances in rapid genotyping of MTBC based on single nucleotide polymorphisms (SNP), allow for phylogenetically robust strain classification, paving the way for defining genotype-phenotype relationships in clinical settings. Such studies have revealed that, in addition to host and environmental factors, strain variation in the MTBC influences the outcome of TB infection and disease. In Tanzania, such molecular epidemiological studies of TB however are scarce in spite of a high TB burden. Methods and findings Here we used SNP-typing to characterize a nationwide collection of 2,039 MTBC clinical isolates representative of 1.6% of all new and retreatment TB cases notified in Tanzania during 2012 and 2013. Four lineages, namely Lineage 1-4 were identified within the study population. The distribution and frequency of these lineages varied across regions but overall, Lineage 4 was the most frequent (n = 866, 42.5%), followed by Lineage 3 (n = 681, 33.4%) and 1 (n = 336, 16.5%), with Lineage 2 being the least frequent (n = 92, 4.5%). We found Lineage 2 to be independently associated with female sex (adjusted odds ratio [aOR] 2.14; 95% confidence interval [95% CI] 1.31 - 3.50, p = 0.002) and retreatment cases (aOR 1.67; 95% CI 0.95 - 2.84, p = 0. 065) in the study population. We found no associations between MTBC lineage and patient age or HIV status. Our sublineage typing based on spacer oligotyping on a subset of Lineage 1, 3 and 4 strains revealed the presence of mainly EAI, CAS and LAM families. Finally, we detected low levels of multidrug resistant isolates among a subset of 144 retreatment cases. Conclusions This study provides novel insights into the MTBC lineages and the possible influence of pathogen-related factors on the TB epidemic in Tanzania. | NA | no | gd of pathogen | NA | no | no |
| 5559 | 2020 | 44113 | Rutter, MT; Roles, AJ; Fenster, CB | 2018 | Quantifying natural seasonal variation in mutation parameters with mutation accumulation lines | Mutations create novel genetic variants, but their contribution to variation in fitness and other phenotypes may depend on environmental conditions. Furthermore, natural environments may be highly heterogeneous. We assessed phenotypes associated with survival and reproductive success in over 30,000 plants representing 100 mutation accumulation lines of Arabidopsis thaliana across four temporal environments at a single field site. In each of the four assays, environmental variance was substantially larger than mutational variance. For some traits, whether mutational variance was significantly varied between seasons. The founder genotype had mean trait values near the mean of the distribution of the mutation accumulation lines in all field experiments. New mutations also contributed more phenotypic variation than would be predicted, given phenotypic and sequence-level divergence among natural populations of A.thaliana. The combination of large environmental variance with a mean effect of mutation near zero suggests that mutations could contribute substantially to standing genetic variation. | NA | no | No good fitness measure and measure host GD | NA | no | no |
| 5560 | 2020 | 44042 | Ryabov, EV; Childers, AK; Lopez, D; Grubbs, K; Posada-Florez, F; Weaver, D; Girten, W; vanEngelsdorp, D; Chen, YP; Evans, JD | 2019 | Dynamic evolution in the key honey bee pathogen deformed wing virus: Novel insights into virulence and competition using reverse genetics | The impacts of invertebrate RNA virus population dynamics on virulence and infection outcomes are poorly understood. Deformed wing virus (DWV), the main viral pathogen of honey bees, negatively impacts bee health, which can lead to colony death. Despite previous reports on the reduction of DWV diversity following the arrival of the parasitic mite Varroa destructor, the key DWV vector, we found high genetic diversity of DWV in infested United States honey bee colonies. Phylogenetic analysis showed that divergent US DWV genotypes are of monophyletic origin and were likely generated as a result of diversification after a genetic bottleneck. To investigate the population dynamics of this divergent DWV, we designed a series of novel infectious cDNA clones corresponding to coexisting DWV genotypes, thereby devising a reverse-genetics system for an invertebrate RNA virus quasispecies. Equal replication rates were observed for all clone-derived DWV variants in single infections. Surprisingly, individual clones replicated to the same high levels as their mixtures and even the parental highly diverse natural DWV population, suggesting that complementation between genotypes was not required to replicate to high levels. Mixed clone-derived infections showed a lack of strong competitive exclusion, suggesting that the DWV genotypes were adapted to coexist. Mutational and recombination events were observed across clone progeny, providing new insights into the forces that drive and constrain virus diversification. Accordingly, our results suggest that Varroa influences DWV dynamics by causing an initial selective sweep, which is followed by virus diversification fueled by negative frequency-dependent selection for new genotypes. We suggest that this selection might reflect the ability of rare lineages to evade host defenses, specifically antiviral RNA interference (RNAi). In support of this hypothesis, we show that RNAi induced against one DWV strain is less effective against an alternate strain from the same population. | NA | no | No good fitness measure and measure host GD | NA | no | no |
| 5561 | 2020 | 44113 | Sabatini, A; Podda, C; Frau, G; Cani, MV; Musu, A; Serra, M; Palmas, F | 2018 | Restoration of native Mediterranean brown trout Salmo cettii Rafinesque, 1810 (Actinopterygii: Salmonidae) populations using an electric barrier as a mitigation tool | This study assessed the effectiveness of a novel conservation strategy for the native brown trout populations in Sardinia (Italy). The conservation measures included: (i) installation of an electric fish barrier; (ii) removal of all the resident introgressed brown trout upstream from the barrier; (iii) restocking of the upstream region with native Sardinian trout genetically characterised; and (iv) estimation of native trout population size with prediction of future growth. Introgressed brown trout were removed using multiple-pass electrofishing methods in a 2-km region upstream from the barrier. In two eradication campaigns, conducted in 2010 and 2012, a total of 1801 trout were captured, comprising a total biomass of 55,501 g. The capture efficiency analysis showed total capture rates of over 99% were achieved for parr (0+ age cohort), and 100% for adult trout. After the eradication campaigns we restocked this area with native brown trout (Adriatic lineage, AD, according to the mitochondrial DNA control region nomenclature). The monitoring campaigns showed good survival (S = 60%) and reproduction of 3.1 recruits/adult trout. Successful breeding was recorded starting from the summer of 2015 and a self-sustaining population resulted after only two restocking events. The results of this restoration project allow recommendations to be made for future conservation eradication and repopulation projects for restoration of typical Mediterranean streams. | NA | no | No good fitness measure and measure host GD | NA | no | no |
| 5562 | 2020 | 44112 | Sabella, E; Pierro, R; Luvisi, A; Panattoni, A; D’Onofrio, C; Scalabrelli, G; Nutricati, E; Aprile, A; De Bellis, L; Materazzi, A | 2018 | Phylogenetic analysis of viruses in Tuscan Vitis vinifera sylvestris (Gmeli) Hegi | The health status of the native grapevine Vitis vinifera subsp. sylvestris (Gmeli) Hegi in natural areas in Europe has received little attention. A survey was carried out on wild grapevines in Tuscany (Italy), where isolates of the Grapevine rupestris stem pitting virus (GRSPaV), Grapevine leafroll-associated virus 1 and 3 (GLRaV-1 and GLRaV-3) and Grapevine virus A (GVA) were detected. The complete coat protein (CP) region of these isolates was sequenced to investigate the relationship of the viral variants from Tuscan wild grapevines with isolates from different geographical origins. According to the phylogenetic analyses, GLRaV-1 and GLRaV-3 isolates from Tuscan wild grapevines clustered with isolates from cultivated grapevines with nucleotide sequence identities ranging from 66% to 87% and from 72.5% to 99% respectively, without any correlation between the distribution and geographical origin. Conversely, GRSPaV and GVA isolates clustered together with other Italian isolates from V. vinifera with nucleotide sequence identities ranging from 71.14% to 96.12% and from 73.5% to 92%, respectively. Our analysis of the whole amino acid sequences revealed a high conservation level for the studied proteins explained by a selective pressure on this genomic region, probably due to functional constraints imposed on CP, such as specific interactions with cellular receptors in the insect vectors necessary for successful transmission. In addition, analyses of genetic recombination suggest no significant point mutations that might play a significant role in genetic diversification. The dN/dS ratio also estimated a low number of non-silent mutations, highlighting the purifying selective pressure. The widespread distribution of the Rugose wood complex (GRSPaV and GVA associated disease) in comparison with the Grapevine Leafroll associated viruses (GLRaV-1 and -3) could explain the major geographical correlation found for the viral variants detected in Tuscany. | NA | no | gd of pathogen | NA | no | no |
| 5563 | 2020 | 44040 | Saeed, S; Ahmed, A; Laghari, SK; Ali, GM; Begum, S | 2020 | GENETIC AND CHEMICAL VARIATION OF SERIPHIDIUM QUETTENSE AN ENDEMIC TAXA OF TWO MOUNTAINOUS REGIONS OF QUETTA, PAKISTAN | Serriphidium quettense of the Asteraceae family is endemic to Balochistan, Pakistan. It has been used in traditional medicine for treating gastrointestinal diseases; while also having germicidal and antibacterial properties. However, no molecular and chemical characterization was demarcated earlier. Thus this study aimed to depict the genetic variability in two mountainous populations by molecular markers. Genetic variability assessment was carried out by polymorphic bands analysis to generate a dendrogram based on Sequential Agglomerative Hierarchical and Non-overlapping (SAHN) algorithm by Unweighted Pair Group Method of Arithmetic (UPGMA) means by using Numerical Taxonomy and Multivariate Analysis System (NTSYS) Pc version 2.01 for Cluster analysis. Chemical characterization was done by evaluating Total Phenolic Content (TPC) and flavonoids. A total of 68 amplified loci with 30 polymorphic were found with an average of 11.3 and 45% polymorphism. Cluster dendrogram based on similarity coefficient ranged from 0.285 to 0.785. Chemical vaiation was assesed by Agglomerative Hierarchical Clustering (AHC) based on dissimilarity matrix as indicated by two main clusters. Cluster 1 was represented by two sub clusters exhibited more chemical variation with in Zargoon population. Overall results obtained exhibited the diverse pattern of genetic and chemical variation. In conclusion, molecular marker profiling together with phytochemical variation of total phenolic and flavonoid content in all accessions of S. quettense and impact of ecological diversity on genetic and chemical variation can be used as base line study. Furthermore, implementation of conservation strategies of this endemic medicinal plant is highly recommended. | NA | no | No good fitness measure and measure host GD | NA | no | no |
| 5564 | 2020 | 44111 | Safner, T; Buzan, E; Rezic, A; Sprem, N | 2019 | Small-scale spatial genetic structure of Alpine chamois (Rupicapra rupicapra) in Northern Dinarides | Chamois (Rupicapra rupicapra) is a mountain-dwelling ungulate inhabiting predominantly rocky habitat with steep slopes. Since it mostly inhabits boreal habitats at high altitudes, low valleys tend to separate populations, thereby limiting gene flow. In the present study, we genotyped 54 georeferenced chamois using 20 SSR loci to test the influence of the Kupa River on the spatial genetic structure of the population in the bordering area between Croatia and Slovenia in the northern Dinaric Mountains. Both GENELAND and STRUCTURE assigned all individuals to one spatial cluster, indicating that the Kupa River does not represent a barrier to gene flow for chamois. Unfortunately, in 2015, a razor wire fence was constructed along the Croatian-Slovenian border at the Kupa River. This fence represents a major threat to the chamois population as it may cause mortality, obstruct seasonal dispersal, and reduce the effective population size. If the fence remains as it is, changes in the genetic structure and genetic diversity of the population due to the effect of drift and reduced effective population size can be predicted over the next generations. | NA | no | No good fitness measure and measure host GD | NA | no | no |
| 5565 | 2020 | 44111 | Sagonas, K; Runemark, A; Antoniou, A; Lymberakis, P; Pafilis, P; Valakos, ED; Poulakakis, N; Hansson, B | 2019 | Selection, drift, and introgression shape MHC polymorphism in lizards | The major histocompatibility complex (MHC) has long served as a model for the evolution of adaptive genetic diversity in wild populations. Pathogen-mediated selection is thought to be a main driver of MHC diversity, but it remains elusive to what degree selection shapes MHC diversity in complex biogeographical scenarios where other evolutionary processes (e.g. genetic drift and introgression) may also be acting. Here we focus on two closely related green lizard species, Lacerta trilineata and L. viridis, to address the evolutionary forces acting on MHC diversity in populations with different biogeographic structure. We characterized MHC class I exon 2 and exon 3, and neutral diversity (microsatellites), to study the relative importance of selection, drift, and introgression in shaping MHC diversity. As expected, positive selection was a significant force shaping the high diversity of MHC genes in both species. Moreover, introgression significantly increased MHC diversity in mainland populations, with a primary direction of gene flow from L. viridis to L. trilineata. Finally, we found significantly fewer MHC alleles in island populations, but maintained MHC sequence and functional diversity, suggesting that positive selection counteracted the effect of drift. Overall, our data support that different evolutionary processes govern MHC diversity in different biogeographical scenarios: positive selection occurs broadly while introgression acts in sympatry and drift when the population sizes decrease. | NA | no | mHC diversity | NA | no | no |
| 5566 | 2020 | 44113 | Saka, T; Nishita, Y; Masuda, R | 2018 | Low genetic variation in the MHC class II DRB gene and MHC-linked microsatellites in endangered island populations of the leopard cat (Prionailurus bengalensis) in Japan | Isolated populations of the leopard cat (Prionailurus bengalensis) on Tsushima and Iriomote islands in Japan are classified as subspecies P. b. euptilurus and P. b. iriomotensis, respectively. Because both populations have decreased to roughly 100, an understanding of their genetic diversity is essential for conservation. We genotyped MHC class II DRB exon 2 and MHC-linked microsatellite loci to evaluate the diversity of MHC genes in the Tsushima and Iriomote cat populations. We detected ten and four DRB alleles in these populations, respectively. A phylogenetic analysis showed DRB alleles from both populations to be closely related to those in other felid DRB lineages, indicating trans-species polymorphism. The MHC-linked microsatellites were more polymorphic in the Tsushima than in the Iriomote population. The MHC diversity of both leopard cat populations is much lower than in the domestic cat populations on these islands, probably due to inbreeding associated with founder effects, geographical isolation, or genetic drift. Our results predict low resistance of the two endangered populations to new pathogens introduced to the islands. | NA | no | mhc diversity | NA | no | no |
| 5567 | 2020 | 44041 | Salas-Martinez, MG; Saldana-Alvarez, Y; Cordova, EJ; Mendiola-Soto, DK; Cid-Soto, MA; Luckie-Duque, A; Vicenteno-Ayala, H; Barajas-Olmos, F; Contreras-Cubas, C; Garcia-Ortiz, H; Jimenez-Ruiz, JL; Centeno-Cruz, F; Martinez-Hernandez, A; Mendoza-Caamal, EC; Mirzaeicheshmeh, E; Orozco, L | 2019 | Genetic variability of five ADRB2 polymorphisms among Mexican Amerindian ethnicities and the Mestizo population | The Mexican population is characterized by high and particular admixture, and the picture of variants associated with disease remains unclear. Here we investigated the distribution of single nucleotide polymorphisms (SNPs) in the Mexican population. We focused on two nonsynonymous and three synonymous SNPs in the beta-2 adrenergic receptor gene (ADRB2), which plays key roles in energy balance regulation. These SNPs were genotyped in 2,011 Mexican Amerindians (MAs) belonging to 62 ethnic groups and in 1,980 geographically matched Mexican Mestizos (MEZs). The frequency distribution of all five ADRB2 variants significantly differed between MAs, MEZs, and other continental populations (CPs) from the 1000 Genomes database. Allele frequencies of the three synonymous SNPs rs1042717A, rs1042718A, and rs1042719C were significantly higher in Mexican individuals, particularly among MAs, compared to in the other analyzed populations (P<0.05). The non-synonymous ADRB2 Glu27 allele (rs1042714G), which is associated with several common conditions, showed the lowest frequency in MAs (0.03) compared to other populations worldwide. Among MEZs, this allele showed a frequency of 0.15, intermediate between that in MAs and in Iberians (0.43). Moreover, Glu27 was the only SNP exhibiting a geographic gradient within the MEZ population (from 0.22 to 0.11), reflecting admixed mestizo ancestry across the country. Population differentiation analysis demonstrated that Glu27 had the highest F-ST value in MAs compared with Europeans (CEU) (0.71), and the lowest between MAs and Japanese (JPT) (0.01), even lower than that observed between MAs and MEZs (0.08). This analysis demonstrated the genetic diversity among Amerindian ethnicities, with the most extreme FST value (0.34) found between the Nahuatls from Morelos and the Seris. This is the first study of ADRB2 genetic variants among MA ethnicities. Our findings add to our understanding of the genetic contribution to variability in disease susceptibility in admixed populations. | NA | no | human | NA | no | no |
| 5568 | 2020 | 44112 | Salim, B; Amin, M; Igarashi, M; Ito, K; Jongejan, F; Katakura, K; Sugimoto, C; Nakao, R | 2019 | Recombination and purifying and balancing selection determine the evolution of major antigenic protein 1 (map 1) family genes in Ehrlichia ruminantium | Heartwater is an economically important disease of ruminants caused by the tick-borne bacterium Ehrlichia ruminantium. The disease is present throughout sub-Saharan Africa as well as on several islands in the Caribbean, where it poses a risk of spreading onto the American mainland. The dominant immune response of infected animals is directed against the variable outer membrane proteins of E. ruminantium encoded by a polymorphic multigene family. Here, we examined the full-length sequence of the major antigenic protein 1 (map1) family genes in multiple E. ruminantium isolates from different African countries and the Caribbean, collected at different time points to infer the possible role of recombination breakpoint and natural selection. A high level of recombination was found particularly in map1 and map1-2. Evidence of strong negative purifying selection in map1 and balancing selection to maintain genetic variation across these samples from geographically distinct countries suggests host pathogen co-evolution. This co-evolution between the host and pathogen results in balancing selection by maintaining genetic diversity that could be explained by the demographic history of long-term pathogen pressure. This signifies the adaptive role and the molecular evolutionary forces underpinning E. ruminantium map1 multigene family antigenicity. | NA | no | No good fitness measure and measure host GD | NA | no | no |
| 5569 | 2020 | 44113 | Sallam, AH; Tyagi, P; Brown-Guedira, G; Muehlbauer, GJ; Hulse, A; Steffenson, BJ | 2017 | Genome-Wide Association Mapping of Stem Rust Resistance in Hordeum vulgare subsp spontaneum | Stem rust was one of the most devastating diseases of barley in North America. Through the deployment of cultivars with the resistance gene Rpg1, losses to stem rust have been minimal over the past 70 yr. However, there exist both domestic (QCCJB) and foreign (TTKSK aka isolate Ug99) pathotypes with virulence for this important gene. To identify new sources of stem rust resistance for barley, we evaluated the Wild Barley Diversity Collection (WBDC) (314 ecogeographically diverse accessions of Hordeum vulgare subsp. spontaneum) for seedling resistance to four pathotypes (TTKSK, QCCJB, MCCFC, and HKHJC) of the wheat stem rust pathogen (Puccinia graminis f. sp. tritici, Pgt) and one isolate (92-MN-90) of the rye stem rust pathogen (P. graminis f. sp. secalis, Pgs). Based on a coefficient of infection, the frequency of resistance in the WBDC was low ranging from 0.6% with HKHJC to 19.4% with 92-MN-90. None of the accessions was resistant to all five cultures of P. graminis. A genome-wide association study (GWAS) was conducted to map stem rust resistance loci using 50,842 single-nucleotide polymorphic markers generated by genotype-by-sequencing and ordered using the new barley reference genome assembly. After proper accounting for genetic relatedness and structure among accessions, 45 quantitative trait loci were identified for resistance to P. graminis across all seven barley chromosomes. Three novel loci associated with resistance to TTKSK, QCCJB, MCCFC, and 92-MN-90 were identified on chromosomes 5H and 7H, and two novel loci associated with resistance to HKHJC were identified on chromosomes 1H and 3H. These novel alleles will enhance the diversity of resistance available for cultivated barley. | NA | no | No good fitness measure and measure host GD | NA | no | no |
| 5570 | 2020 | 44111 | Salvo, MA; Aliota, MT; Moncla, LH; Velez, ID; Trujillo, AI; Friedrich, TC; Osorio, JE | 2019 | Tracking dengue virus type 1 genetic diversity during lineage replacement in an hyperendemic area in Colombia | Dengue virus (DENV) is a flavivirus responsible for the most common and burdensome arthropod-borne viral disease of humans[1]. DENV evolution has been extensively studied on broad geographic and time scales, using sequences from a single gene[2,3]. It is believed that DENV evolution in humans is dominated primarily by purifying selection due to the constraint of maintaining fitness in both humans and mosquitoes[4,5]. Few studies have explored DENV evolutionary dynamics using whole genome sequences, nor have they explored changes in viral diversity that occur during intra-epidemic periods. We used deep sequencing of the viral coding region to characterize DENV-1 evolution in a Colombian population sampled during two high-prevalence dengue seasons in which serotype dominance shifted. Our data demonstrate patterns of strain extinction and replacement within DENV-1 as its prevalence waned and DENV-3 became established. A comparison of whole-genome versus single-gene-based phylogenetic analyses highlights an important difference in evolutionary patterns. We report a trend of higher nonsynonymous to synonymous diversity ratios among non-structural (NS) genes, and statistically significantly higher values among these ratios in the NS1 gene after DENV-1 strain replacement. These results suggest that positive selection could be driving DENV evolution within individual communities. Signals of positive selection coming from distinct samples may be drowned out when combining multiple regions with differing patterns of endemic transmission as commonly done by large-scale geo-temporal assessments. Here, we frame our findings within a small, local transmission history which aids significance. Moreover, these data suggest that the NS1 gene, rather than the E gene, may be a target of positive selection, although not mutually exclusive, and potentially useful sentinel of adaptive changes at the population level. | NA | no | No good fitness measure and measure host GD | NA | no | no |
| 5571 | 2020 | 44112 | Sams, AJ; Boyko, AR | 2019 | Fine-Scale Resolution of Runs of Homozygosity Reveal Patterns of Inbreeding and Substantial Overlap with Recessive Disease Genotypes in Domestic Dogs | Inbreeding leaves distinct genomic traces, most notably long genomic tracts that are identical by descent and completely homozygous. These runs of homozygosity (ROH) can contribute to inbreeding depression if they contain deleterious variants that are fully or partially recessive. Several lines of evidence have been used to show that long (> 5 megabase) ROH are disproportionately likely to harbor deleterious variation, but the extent to which long vs. short tracts contribute to autozygosity at loci known to be deleterious and recessive has not been studied. In domestic dogs, nearly 200 mutations are known to cause recessive diseases, most of which can be efficiently assayed using SNP arrays. By examining genome-wide data from over 200,000 markers, including 150 recessive disease variants, we built high-resolution ROH density maps for nearly 2,500 dogs, recording ROH down to 500 kilobases. We observed over 678 homozygous deleterious recessive genotypes in the panel across 29 loci, 90% of which overlapped with ROH inferred by GERMLINE. Although most of these genotypes were contained in ROH over 5 Mb in length, 14% were contained in short (0.5 - 2.5 megabase) tracts, a significant enrichment compared to the genetic background, suggesting that even short tracts are useful for computing inbreeding metrics like the coefficient of inbreeding estimated from ROH (F-ROH). In our dataset, F-ROH differed significantly both within and among dog breeds. All breeds harbored some regions of reduced genetic diversity due to drift or selective sweeps, but the degree of inbreeding and the proportion of inbreeding caused by short vs. long tracts differed between breeds, reflecting their different population histories. Although only available for a few species, large genome-wide datasets including recessive disease variants hold particular promise not only for disentangling the genetic architecture of inbreeding depression, but also evaluating and improving upon current approaches for detecting ROH. | NA | no | dometicated host | NA | no | no |
| 5572 | 2020 | 44042 | Sanaei, E; Husemann, M; Seiedy, M; Rethwisch, M; Tuda, M; Toshova, TB; Kim, MJ; Atanasova, D; Kim, I | 2019 | Global genetic diversity, lineage distribution, and Wolbachia infection of the alfalfa weevil Hypera postica (Coleoptera: Curculionidae) | The alfalfa weevil (Hypera postica) is a well-known example of a worldwide-distributed pest with high genetic variation. Based on the mitochondrial genes, the alfalfa weevil clusters into two main mitochondrial lineages. However, there is no clear picture of the global diversity and distribution of these lineages; neither the drivers of its diversification are known. However, it appears likely that historic demographic events including founder effects played a role. In addition, Wolbachia, a widespread intracellular parasite/symbiont, likely played an important role in the evolution of the species. Wolbachia infection so far was only detected in the Western lineage of H. postica with no information on the infecting strain, its frequency, and its consequences on the genetic diversity of the host. We here used a combination of mitochondrial and nuclear sequences of the host and sequence information on Wolbachia to document the distribution of strains and the degree of infection. The Eastern lineage has a higher genetic diversity and is found in the Mediterranean, the Middle East, Eastern Europe, and eastern America, whereas the less diverse Western lineage is found in Central Europe and the western America. Both lineages are infected with the same common strain of Wolbachia belonging to Supergroup B. Based on neutrality tests, selection tests, and the current distribution and diversification of Wolbachia in H. postica, we suggested the Wolbachia infection did not shape genetic diversity of the host. The introduced populations in the United States are generally genetically less diverse, which is in line with founder effects. | NA | no | No good fitness measure and measure host GD | NA | no | no |
| 5573 | 2020 | 44113 | Sanchez, PR; Cobos, A; Navaro, M; Ramos, JT; Pagan, I; Holguin, A | 2017 | Impact of Clinical Parameters in the Intrahost Evolution of HIV-1 Subtype B in Pediatric Patients: A Machine Learning Approach | Determining the factors modulating the genetic diversity of HIV-1 populations is essential to understand viral evolution. This study analyzes the relative importance of clinical factors in the intrahost HIV-1 subtype B (HIV-1B) evolution and in the fixation of drug resistance mutations (DRM) during longitudinal pediatric HIV-1 infection. We recovered 162 partial HIV-1B pol sequences (from 3 to 24 per patient) from 24 perinatally infected patients from the Madrid Cohort of HIV-1 infected children and adolescents in a time interval ranging from 2.2 to 20.3 years. We applied machine learning classification methods to analyze the relative importance of 28 clinical/epidemiological/virological factors in the HIV-1B evolution to predict HIV-1B genetic diversity (d), nonsynonymous and synonymous mutations (d(N), d(S)) and DRM presence. Most of the 24 HIV-1B infected pediatric patients were Spanish (91.7%), diagnosed before 2000 (83.3%), and all were antiretroviral therapy experienced. They had from 0.3 to 18.8 years of HIV-1 exposure at sampling time. Most sequences presented DRM. The best-predictor variables for HIV-1B evolutionary parameters were the age of HIV-1 diagnosis for d, the age at first antiretroviral treatment for dN and the year of HIV-1 diagnosis for d(s). The year of infection (birth year) and year of sampling seemed to be relevant for fixation of both DRM at large and, considering drug families, to protease inhibitors (PI). This study identifies, for the first time using machine learning, the factors affecting more HIV-1B pol evolution and those affecting DRM fixation in HIV-1B infected pediatric patients. | NA | no | hiv | NA | no | no |
| 5574 | 2020 | 44111 | Sannikov, SN; Sannikova, NS; Petrova, IV; Egorov, EV | 2019 | The Hypothesis about the Lofoten Pleistocene Refugium for Pinus sylvestris L. | The dates of appearance of a representative portion of Pinus sylvestris L. pollen in palynospectra (20%) have been analyzed as a parameter reflecting the spread of leading-edge pine populations from central and eastern Europe to the Lofoten region via probable alternative routes of population immigration. It has been found that they could reach this region not earlier than 6500-7000 years BP, i.e., 2500-3000 years later than the factual palynological date, even at the maximum possible rate of their spread (300-500 km/1000 years). Therefore, the probability of the origin of Lofoten P. sylvestris populations from adventive populations that migrated to Scandinavia from adjacent European regions is excluded. The generalization of the results of studies on the chronopalynology and rate of Holocene expansion of P sylvestris and data on paleoglaciology, paleomarinology, paleobotany, and modern genogeography and ecology of Scots pine has confirmed the hypothesis about the survival of autochthonous refugial populations of this species on the Lofoten Islands during the last glacial phase and their expansion during the Holocene. | NA | no | No good fitness measure and measure host GD | NA | no | no |
| 5575 | 2020 | 44040 | Santacruz, A; Ornelas-Garcia, CP; De Leon, GPP | 2020 | Diversity of Rhabdochona mexicana (Nematoda: Rhabdochonidae), a parasite of Astyanax spp. (Characidae) in Mexico and Guatemala, using mitochondrial and nuclear genes, with the description of a new species | Among fish parasitic nematodes Rhabdochona is one of the most speciose genera, with c. 100 species. Twelve congeneric species occur in Mexican freshwater fishes, in a region located between the Nearctic and Neotropical biogeographical regions. Host association and biogeographical history have determined the high species richness of Rhabdochona in Mexico. One of these species, Rhabdochona mexicana, is highly specific to the characid genus Astyanax. Characids are a group of freshwater fish with Neotropical affinity. In this paper, we explore the genetic diversity of R. mexicana through samples obtained from populations of Astyanax spp. across river basins of Mexico and Guatemala. Sequences of one mitochondrial and two ribosomal genes were obtained from 38 individuals and analysed using Maximum Likelihood and Bayesian Inference analysis. Phylogenetic analyses using cox1, and a concatenated alignment of 18S + 28S + cox1 recovered two genetic lineages. One of them corresponded with R. mexicana sensu stricto; this lineage included three reciprocally monophyletic subgroups; the other lineage was highly divergent and represented a putative candidate species. A detailed morphological study was conducted to corroborate the molecular findings. We describe a new species herein and discuss the implications of using molecular tools to increase our knowledge about the diversity of a speciose genus such as Rhabdochona. | NA | no | No group level | NA | no | no |
| 5576 | 2020 | 44039 | Santos, AD; Barbosa, NCCP; Thomazini, TC; Morales, AC | 2020 | Genetic Variability of Ceraeochrysa cincta, Ceraeochrysa claveri, and Ceraeochrysa cubana (Neuroptera: Chrysopidae) Populations in Agroecosystems of Southeast Brazil | The species of the genus Ceraeochrysa, known as green lacewings or trash-carriers, are widely distributed along the Americas and its islands. In Brazil, 28 species are found, including Ceraeochrysa cincta (Schneider), Ceraeochrysa claveri (Naves), and Ceraeochrysa cubana (Hagen).These species are recorded on many crops, where they are often used for biological control. For this use, knowledge of the genetic features of the species is extremely important because they are associated to the species’ ability to withstand different conditions in new environments, such as variations of temperature and presence of pathogens. However, little is known about the genetic features of Ceraeochrysa species. Here, we analyze and compare the distribution of the genetic variability of C. cincta, C. claveri, and C. cubana in agroecosystem populations of southeast Brazil. We found a high genetic diversity in each of the three species, and no strong genetic structure was detected, such that genetic diversity is broadly shared among the crops and localities analyzed. We can conclude that there was a high gene flow among the sampled Ceraeochrysa populations (natural or driven by anthropic action) since the exchange of seedlings among crops can lead to the distribution of the specimens. | NA | no | No good fitness measure | NA | no | no |
| 5577 | 2020 | 44040 | Santos, J; Xavier, M; Cardoso, L; Nobre, SAM; Bacchi, RR; Cangussu, CHC; Almeida, AC; Leite, LN; Barreto, NAP; Xavier, AREO | 2020 | Identification and molecular analysis of yeasts found in domestic pigeon droppings in Montes Claros, MG, Brazil | Pigeon droppings carry microorganisms such as Candida spp., which have high genetic variability and are the most common opportunistic pathogens in humans. There is a need to establish fast, reliable and simple laboratory methods to differentiate pathogenic from non-pathogenic yeast species in pigeon droppings, as they can be a source of contamination for humans. We examined yeasts isolated from domestic pigeon droppings through a proteomic approach using matrix-assisted laser desorption ionization (MALDI-TOF). We also examined genetic characteristics of the isolates by restriction fragment length polymorphism (RFLP) PCR and enterobacterial repetitive intergenic consensus sequences PCR methods. Five colonies isolated from domestic pigeon droppings collected at three different sampling locations on the roof of a Brazilian university building were identified as Candida krusei and Candida rugosa by MALDI-TOF. Phylogenetic studies using the two PCR-based molecular methods were able to differentiate the species C. krusei and C. rugosa into different groups. These genomic methods gave similar results and proved useful for genetic diversity analysis and interspecific differentiation of Candida isolates. | NA | no | No good fitness measure and measure host GD | NA | no | no |
| 5578 | 2020 | 44112 | Santos, PSC; Mezger, M; Kolar, M; Michler, FU; Sommer, S | 2018 | The best smellers make the best choosers: mate choice is affected by female chemosensory receptor gene diversity in a mammal | The products of the genes of the major histocompatibility complex (MHC) are known to be drivers of pathogen resistance and sexual selection enhancing offspring genetic diversity. The MHC further influences individual odour types and social communication. However, little is known about the receptors and their volatile ligands that are involved in this type of chemical communication. Here, we have investigated chemosensory receptor genes that ultimately enable females to assess male genes through odour cues. As a model, we used an invasive population of North American raccoons (Procyon lotor) in Germany. We investigated the effect of two groups of chemosensory receptor genes-trace amine-associated receptors (TAARs) and olfactory receptors (ORs)-on MHC-dependent mate choice. Females with more alleles of the TAAR or OR loci were more likely to choose a male with a diverse MHC. We additionally found that MHC class I genes have a stronger effect on mate choice than the recently reported effect for MHC class II genes, probably because of their immunological relevance for viral resistance. Our study is among the first to show a genetic link between behaviour and chemosensory receptor genes. These results contribute to understanding the link between genetics, olfaction and associated life-history decisions. | NA | no | No good fitness measure and measure host GD | NA | no | no |
| 5579 | 2020 | 44111 | Santos, TTD; Amorim, VBD; dos Santos-Serejo, JA; Ledo, CAD; Haddad, F; Ferreira, CF; Amorim, EP | 2019 | Genetic variability among autotetraploid populations of banana plants derived from wild diploids through chromosome doubling using SSR and molecular markers based on retrotransposons | The development of banana cultivars resistant to diseases is the most viable way to minimize crop damages. However, this strategy is limited by the low fertility observed among commercial triploid cultivars, especially those of the Cavendish subgroup. Induced polyploidy has the potential to overcome sterility barriers and allows the development of triploid genotypes with fruit sensory profiles aligned with market demands. This work characterized agronomically five autotetraploid plant populations derived from diploids treated with colchicine. The experiment was conducted using an augmented block design, and 13 agronomic characteristics were evaluated during two production cycles. The sum of ranks proposed by Mulamba and Mock allowed the genotypes Pisang Lilin 8, Malbut 10, Niyarma Yik 2, Pisang Mas 3, and Thong Dok Mak 2 to be selected, which were superior for most of the agronomic characteristics evaluated. SSR and IRAP molecular markers detected variability in the autotetraploid populations, and the analysis of molecular variance supports that approximately 78% of identified variability was contained within the populations studied. Based on the results, it is inferred that the promising autotetraploids can be crossed with diploids to develop secondary triploid hybrids. | NA | no | No good fitness measure and measure host GD | NA | no | no |
| 5580 | 2020 | 44039 | Sapp, JR; Yost, J; Lyon, BE | 2020 | The socially parasitic antPolyergus mexicanushas host-associated genetic population structure and related neighbouring colonies | The genetic structure of populations can be both a cause and a consequence of ecological interactions. For parasites, genetic structure may be a consequence of preferences for host species or of mating behaviour. Conversely, genetic structure can influence where conspecific interactions among parasites lay on a spectrum from cooperation to conflict. We used microsatellite loci to characterize the genetic structure of a population of the socially parasitic dulotic (aka slave-making) ant (Polyergus mexicanus), which is known for its host-specificity and conspecific aggression. First, we assessed whether the pattern of host species use by the parasite has influenced parasite population structure. We found that host species use was correlated with subpopulation structure, but this correlation was imperfect: some subpopulations used one host species nearly exclusively, while others used several. Second, we examined the viscosity of the parasite population by measuring the relatedness of pairs of neighbouring parasitic ant colonies at varying distances from each other. Although natural history observations of local dispersal by queens suggested the potential for viscosity, there was no strong correlation between relatedness and distance between colonies. However, 35% of colonies had a closely related neighbouring colony, indicating that kinship could potentially affect the nature of some interactions between colonies of this social parasite. Our findings confirm that ecological forces like host species selection can shape the genetic structure of parasite populations, and that such genetic structure has the potential to influence parasite-parasite interactions in social parasites via inclusive fitness. | NA | no | No good fitness measure and measure host GD | NA | no | no |
| 5581 | 2020 | 44112 | Sarao, PS; Bentur, JS | 2018 | Quantification of antibiosis levels in nine different rice genotypes against Nilaparvata lugens (Homoptera: Delphacidae) | Studies on different parameters of antibiosis to simultaneously compare diverse genotypes with different resistance levels are lacking. Such studies are necessary to identify the genetic variability among resistant genotypes, which, ultimately, can lead to the identification of resistance genes with diverse mechanisms. The current study was undertaken to quantify antibiosis levels in nine rice genotypes against the brown planthopper (BPH), Nilaparvata lugens (Stal), under glasshouse conditions using nine parameters. Among these genotypes, RP2068-18-3-5 and Ptb33 displayed significantly better performance as compared to other genotypes in most of the parameters studied and did not differ from each other. Rathu Heenati was the best in unfertilised eggs laid and similar to the above two genotypes regarding percent of nymphal survival. All the test genotypes performed significantly better than the susceptible check TN1, though INRC3021 did not differentiate from the former in some tests. The overall damage score was positively and significantly correlated with the amount of honeydew excretion, percent nymphal survival and emergence, the proportion of brachypterous females, female population, and growth index, and were negatively and significantly correlated with percent unfertilised eggs laid and nymphal development period. Regression analysis revealed a significant linear relationship between damage score and nymphal emergence, nymphal survival percentage, and proportion of brachypterous females. RP2068-18-3-5 is the new source of BPH resistance identified through this study, which breeders can further use in resistance breeding. | NA | no | No good fitness measure and measure host GD | NA | no | no |
| 5582 | 2020 | 44113 | Sarkar, A; Stoneking, M; Nandineni, MR | 2017 | Unraveling the human salivary microbiome diversity in Indian populations | The importance of studying the salivary microbiome has been highlighted for its connection to health and disease and as a potential tool for supplementing human genetic diversity studies. While the salivary microbiome has been studied in various world populations, Indian populations have not been examined. We therefore analyzed microbiome diversity in saliva samples from 92 volunteers from eight different sampling locations in India by amplifying and sequencing variable regions (V1 and V2) of the bacterial 16S rRNA gene. The results showed immense bacterial richness in Indian populations; we identified 165 bacterial genera and 785 unique Operational Taxonomic Units (OTUs), with substantial sharing among the populations. There were small, but significant correlations in the abundance of bacterial genera in sampling locations from the same geographic region. Most of the core OTUs detected were also observed previously in other populations, but Solobacterium spp., Lachnoanaerobaculum spp. and Alloprevotella spp. were observed to be a component of the saliva microbiome unique to Indian populations. Importantly, nine bacterial genera were observed that were not listed in the Human Oral Microbiome Database (HOMD). These results highlight the importance of analyzing underrepresented populations like those of India. | NA | no | No good fitness measure and measure host GD | NA | no | no |
| 5583 | 2020 | 44040 | Sasseron, GR; Benchimol-Reis, LL; Perseguini, JMKC; Paulino, JFC; Bajay, MM; Carbonell, SAM; Chiorato, AF | 2020 | Fusarium oxysporum f. sp. phaseoli genetic variability assessed by new developed microsatellites | Fusarium oxysporum f . sp. phaseoli (Fop) J.B. Kendrich & W.C. Snyder is the causal agent of Fusarium wilt of common bean (Phaseolus vulgaris L.). The objective of this study was to develop microsatellite markers (SSRs) to characterize the genetic diversity of Fop. Two libraries enriched with SSRs were developed and a total of 40 pairs of SSRs were characterized. Out of these, 15 SSRs were polymorphic for 42 Fop isolates. The number of alleles varied from two to ten, with an average of four alleles per locus and an average PIC (Polymorphic Information Content) of 0.38. The genetic diversity assessed by microsatellites for Fop was low, as expected for an asexual fungus, and not associated with geographic origin, but they were able to detect enough genetic variability among isolates in order to differentiate them. Microsatellites are a robust tool widely used for genetic fingerprinting and population structure analyses. SSRs for Fop may be an efficient tool for a better understanding of the ecology, epidemiology and evolution of this pathogen. | NA | no | No good fitness measure and measure host GD | NA | no | no |
| 5584 | 2020 | 44113 | Savage, AE; Mulder, KP; Torres, T; Wells, S | 2018 | Lost but not forgotten: MHC genotypes predict overwinter survival despite depauperate MHC diversity in a declining frog | The amphibian disease chytridiomycosis, caused by the fungus Batrachochytrium dendrobatidis (Bd), has contributed to the decline of Chiricahua leopard frogs (Rana chiricahuensis), a federally threatened species native to the Southwestern United States. We characterized immunogenetic variability in R. chiricahuensis by sequencing an expressed Major Histocompatibility Complex (MHC) class II beta gene across 13 natural populations in Arizona, USA, as well as 283 individuals that were captive reared from two egg masses. We recovered a total of five class II beta MHC alleles compared to 84 alleles previously characterized in eight natural populations of the Arizona congener R. yavapaiensis, demonstrating reduced MHC diversity in R. chiricahuensis. One allele was fixed in five populations but none of the R. chiricahuensis alleles were closely related to R. yavapaiensis allele Q, which is significantly associated with chytridiomycosis resistance in laboratory trials. Nine of 13 R. chiricahuensis population localities were Bd positive, and bearing allele RachDRB*04 was the best genetic predictor of an individual being infected with Bd. A total of three class II beta alleles were recovered from captive reared individuals, which were released to two natural population localities followed by recapture surveys to assess MHC-based survival over winter, the time when chytridiomycosis outbreaks are most severe. At one site, all released animals were fixed for a single allele and MHC-based survival could not be assessed. At the second site, fewer than half of the released but all of the recaptured individuals were homozygous for RachDRB*05, indicating that MHC genotype is important in determining Bd survival under natural field conditions. We conclude that the limited MHC variation in R. chiricahuensis is likely the consequence rather than the cause of natural selection favoring alleles that promote survival in the face of Bd. Our study highlights that preserving even low levels of functional genetic variation may be essential for population persistence, and that local disease adaptation may present as a reduction in genetic diversity. These finding also suggest that for populations that have declined due to a specific infectious pathogen, MHC-based genetically-informed reintroduction approaches may enhance species recovery efforts. | NA | no | mhc diversity | NA | no | no |
| 5585 | 2020 | 44111 | Savage, AE; Muletz-Wolz, CR; Grant, EHC; Fleischer, RC; Mulder, KP | 2019 | Functional variation at an expressed MHC class II locus associates with Ranavirus infection intensity in larval anuran populations | Infectious diseases are causing catastrophic losses to global biodiversity. Iridoviruses in the genus Ranavirus are among the leading causes of amphibian disease-related mortality. Polymorphisms in major histocompatibility complex (MHC) genes are significantly associated with variation in amphibian pathogen susceptibility. MHC genes encode two classes of polymorphic cell-surface molecules that can recognize and bind to diverse pathogen peptides. While MHC class I genes are the classic mediators of viral-acquired immunity, larval amphibians do not express them. Consequently, MHC class II gene diversity may be an important predictor of Ranavirus susceptibility in larval amphibians, the life stage most susceptible to Ranavirus. We surveyed natural populations of larval wood frogs (Rana sylvatica), which are highly susceptible to Ranavirus, across 17 ponds and 2years in Maryland, USA. We sequenced the peptide-binding region of an expressed MHC class II locus and assessed allelic and genetic diversity. We converted alleles to functional supertypes and determined if supertypes or alleles influenced host responses to Ranavirus. Among 381 sampled individuals, 26% were infected with Ranavirus. We recovered 20 unique MHC class II alleles that fell into two deeply diverged clades and seven supertypes. MHC genotypes were associated with Ranavirus infection intensity, but not prevalence. Specifically, MHC heterozygotes and supertype ST1/ST7 had significantly lower Ranavirus infection intensity compared to homozygotes and other supertypes. We conclude that MHC class II functional genetic variation is an important component of Ranavirus susceptibility. Identifying immunogenetic signatures linked to variation in disease susceptibility can inform mitigation strategies for combatting global amphibian declines. | NA | no | MHC diversity | NA | no | no |
| 5586 | 2020 | 44113 | Sayed, SM; Ali, EF; El-Arnaouty, SA; Mahmoud, SF; Amer, SA | 2018 | Isolation, identification, and molecular diversity of indigenous isolates of Beauveria bassiana from Taif region, Saudi Arabia | The entomopathogenic fungus, Beauveria bassiana causes a disease known as the white muscadine disease in insects. Most of the entomopathogenic fungi are soil borne nature an attempt was made to collect the soil samples from various ecosystems. Therefore, the present study aimed to isolate native strains of B. bassiana and study their genetic diversity at Taif region, Saudi Arabia, using ITS of nuclear DNA and COI gene analysis by comparing of different isolates from occasional localities through the world. Ninety-four soil samples were collected from different regions at Taif. Only 11 samples had B. bassiana fungus with a ratio of 11.7%. Sequences of ITS (593 bp), and partial COI (437 bp) of these isolates were carried out and revealed that four differed in their genetics. These sequences were deposited in the DDBJ GenBank database with eight accession numbers. Consequently, the three analytical methods (MP, NJ, and ML) executed a single tree with identical topology. The tree identified the studied isolates into two main clusters; the first contained Uzbekistan and USA isolates. The Chinese isolate clustered with this group in the MP tree and was diverged as a single cluster in the NJ tree. The second clade included the newly studied four isolates from Taif and the Italian isolate. The pairwise genetic distances among the four studied isolates (D ranged between 0.002 and 0.008) showed that they were genetically closely related. Further studies are needed to indicate more differentiation among these isolates and to evaluate their efficacy against the important insect pests in Saudi Arabia. | NA | no | No good fitness measure and measure host GD | NA | no | no |
| 5587 | 2020 | 44039 | Sazzini, M; Abondio, P; Sarno, S; Gnecchi-Ruscone, GA; Ragno, M; Giuliani, C; De Fanti, S; Ojeda-Granados, C; Boattini, A; Marquis, J; Valsesia, A; Carayol, J; Raymond, F; Pirazzini, C; Marasco, E; Ferrarini, A; Xumerle, L; Collino, S; Mari, D; Arosio, B; Monti, D; Passarino, G; D’Aquila, P; Pettener, D; Luiselli, D; Castellani, G; Delledonne, M; Descombes, P; Franceschi, C; Garagnani, P | 2020 | Genomic history of the Italian population recapitulates key evolutionary dynamics of both Continental and Southern Europeans | Background The cline of human genetic diversity observable across Europe is recapitulated at a micro-geographic scale by variation within the Italian population. Besides resulting from extensive gene flow, this might be ascribable also to local adaptations to diverse ecological contexts evolved by people who anciently spread along the Italian Peninsula. Dissecting the evolutionary history of the ancestors of present-day Italians may thus improve the understanding of demographic and biological processes that contributed to shape the gene pool of European populations. However, previous SNP array-based studies failed to investigate the full spectrum of Italian variation, generally neglecting low-frequency genetic variants and examining a limited set of small effect size alleles, which may represent important determinants of population structure and complex adaptive traits. To overcome these issues, we analyzed 38 high-coverage whole-genome sequences representative of population clusters at the opposite ends of the cline of Italian variation, along with a large panel of modern and ancient Euro-Mediterranean genomes. Results We provided evidence for the early divergence of Italian groups dating back to the Late Glacial and for Neolithic and distinct Bronze Age migrations having further differentiated their gene pools. We inferred adaptive evolution at insulin-related loci in people from Italian regions with a temperate climate, while possible adaptations to pathogens and ultraviolet radiation were observed in Mediterranean Italians. Some of these adaptive events may also have secondarily modulated population disease or longevity predisposition. Conclusions We disentangled the contribution of multiple migratory and adaptive events in shaping the heterogeneous Italian genomic background, which exemplify population dynamics and gene-environment interactions that played significant roles also in the formation of the Continental and Southern European genomic landscapes. | NA | no | human | NA | no | no |
| 5588 | 2020 | 44112 | Schaefer, J; Kreiser, B; Flanagan, S | 2018 | Population genomics of Fundulus grandis exposed to oil from Deepwater Horizon | The April 2010 Deepwater Horizon (DWH) oil spill released over 4 million barrels of oil into northern Gulf of Mexico ecosystems. Due to the magnitude of the spill and observed effects from previous spills, the ecological impacts were predicted to be catastrophic. While negative effects of petroleum hydrocarbon exposure to fish have been documented at the organismal scale, most studies of fish assemblages have noted few, if any, DWH impacts. Our understanding of DWH effects is therefore limited by a disconnect in results reported at different organizational levels. We used population genomic tools to test hypotheses about population-level DWH impacts to Gulf Killifish (Fundulus grandis) in coastal Mississippi. Fundulus grandis is one of the most abundant marsh species in the northern Gulf of Mexico, and along with the sister species F. heteroclitus, has served as a model for toxicology research. We collected genotyping by sequencing data from four populations of F. grandis (two oiled and two not) to test predictions about DWH-related gene flow, demographics, genetic diversity and evidence of selection. Mississippi populations of F. grandis showed clear genetic structure, but there were few indicators (e.g. reduced genetic diversity, lower N-e estimates) consistent with large-scale mortality or migration related to DWH oil. Only one estimate of effective population size was reduced in oil-exposed populations and of the twenty outlier loci only two were in regions with potential adaptive significance (i.e., immune functions). While the toxic effects of oil on F. grandis at the organismal level is well established, the minimal impacts we detected to populations along the Mississippi coast likely reflects the complexity of the northem Gulf of Mexico and DWH oil spill. However, we do recognize that the patchiness of oil exposure and low genome coverage of the data limit our ability to conclude there were no DWH effects on these populations. | NA | no | No good fitness measure and measure host GD | NA | no | no |
| 5589 | 2020 | 44041 | Schaller, L; Lauschke, VM | 2019 | The genetic landscape of the human solute carrier (SLC) transporter superfamily | The human solute carrier (SLC) superfamily of transporters is comprised of over 400 membrane-bound proteins, and plays essential roles in a multitude of physiological and pharmacological processes. In addition, perturbation of SLC transporter function underlies numerous human diseases, which renders SLC transporters attractive drug targets. Common genetic polymorphisms in SLC genes have been associated with inter-individual differences in drug efficacy and toxicity. However, despite their tremendous clinical relevance, epidemiological data of these variants are mostly derived from heterogeneous cohorts of small sample size and the genetic SLC landscape beyond these common variants has not been comprehensively assessed. In this study, we analyzed Next-Generation Sequencing data from 141,456 individuals from seven major human populations to evaluate genetic variability, its functional consequences, and ethnogeographic patterns across the entire SLC superfamily of transporters. Importantly, of the 204,287 exonic single-nucleotide variants (SNVs) which we identified, 99.8% were present in less than 1% of analyzed alleles. Comprehensive computational analyses using 13 partially orthogonal algorithms that predict the functional impact of genetic variations based on sequence information, evolutionary conservation, structural considerations, and functional genomics data revealed that each individual genome harbors 29.7 variants with putative functional effects, of which rare variants account for 18%. Inter-ethnic variability was found to be extensive, and 83% of deleterious SLC variants were only identified in a single population. Interestingly, population-specific carrier frequencies of loss-of-function variants in SLC genes associated with recessive Mendelian disease recapitulated the ethnogeographic variation of the corresponding disorders, including cystinuria in Jewish individuals, type II citrullinemia in East Asians, and lysinuric protein intolerance in Finns, thus providing a powerful resource for clinical geneticists to inform about population-specific prevalence and allelic composition of Mendelian SLC diseases. In summary, we present the most comprehensive data set of SLC variability published to date, which can provide insights into inter-individual differences in SLC transporter function and guide the optimization of population-specific genotyping strategies in the bourgeoning fields of personalized medicine and precision public health. | NA | no | No good fitness measure and measure host GD | NA | no | no |
| 5590 | 2020 | 44113 | Schenekar, T; Weiss, S | 2017 | Selection and genetic drift in captive versus wild populations: an assessment of neutral and adaptive (MHC-linked) genetic variation in wild and hatchery brown trout (Salmo trutta) populations | Captive bred individuals are often released into natural environments to supplement resident populations. Captive bred salmonid fishes often exhibit lower survival rates than their wild brethren and stocking measures may have a negative influence on the overall fitness of natural populations. Stocked fish often stem from a different evolutionary lineage than the resident population and thus may be maladapted for life in the wild, but this phenomenon has also been linked to genetic changes that occur in captivity. In addition to overall loss of genetic diversity via captive breeding, adaptation to captivity has become a major concern. Altered selection pressure in captivity may favour alleles at adaptive loci like the Major Histocompatibility Complex (MHC) that are maladaptive in natural environments. We investigated neutral and MHC-linked genetic variation in three autochthonous and three hatchery populations of Austrian brown trout (Salmo trutta). We confirm a positive selection pressure acting on the MHC II beta locus, whereby the signal for positive selection was stronger in hatchery versus wild populations. Additionally, diversity at the MHC II beta locus was higher, and more uniform among hatchery samples compared to wild populations, despite equal levels of diversity at neutral loci. We postulate that this stems from a combination of stronger genetic drift and a weakening of positive selection at this locus in wild populations that already have well adapted alleles for their specific environments. | NA | no | No good fitness measure and measure host GD | NA | no | no |
| 5591 | 2020 | 44112 | Schindler, S; Radford, AN | 2018 | Factors influencing within-group conflict over defence against conspecific outsiders seeking breeding positions | In social species, groups face a variety of threats from conspecific outsiders. Defensive actions are therefore common, but there is considerable variation in which individuals contribute and to what extent. There has been some theoretical exploration of this variation when the defence is of shared resources, but the relative contributions when a single intruder threatens a particular breeding position have received less attention. Defensive actions are costly, both for the individual and dependent young, and contributions are likely to differ depending on individual sex, rank and size, current breeding stage, infanticide risk and relatedness levels. Here, we model analytically the relative fitness benefits of different group members to engaging in defence against individual intruders and determine when within-group conflicts of interest might arise over these defensive contributions. Conflicts of interest between the challenged breeder and other group members depend on relatedness to the brood and the potential relatedness reduction if an intruder acquires breeding status. Conflicts are more likely to occur when there is a low chance of winning the contest, low infanticide rates, inefficient defence from helpers, a long remaining brood-dependency period and high external (non-contest-related) mortality. Our work can help explain variation in defensive actions against out-group threats. | NA | no | No good fitness measure and measure host GD | NA | no | no |
| 5592 | 2020 | 44113 | Schmidt-Lebuhn, AN; Marshall, DJ; Dreis, B; Young, AG | 2018 | Genetic rescue in a plant polyploid complex: Case study on the importance of genetic and trait data for conservation management | Knowledge of the biology of rare plant species is indispensable to aid their survival and to inform efficient conservation actions, but in many cases relevant data are lacking. In addition, while studies of conservation genetics have provided a wealth of information on the considerations arising from inbreeding, mate limitation, or local adaptation, the impact of intraspecific polyploidy remains understudied. In this study, we examined the breeding system of the rare Australian daisy Rutidosis lanata (Asteraceae) and screened ten of its populations for their ploidy level to develop recommendations for management actions, in particular, with regard to seed sourcing and genetic rescue. We found R.lanata to represent a polyploid complex, with tetraploid, pentaploid and hexaploid individuals coexisting in the same species. Crossing experiments confirmed R.lanata to be self-incompatible. Mate availability varied from c. 49% to c. 76% across populations. Most populations showed mate availability of c. 50%-70%, suggesting that mate limitation resulting from a lack of local genetic diversity may cause or at least contribute to reduced seed set. Crossing between populations resulted in significantly higher reproductive success for all populations except one, suggesting the possibility of genetic rescue through population mixing. However, the crossing experiments also showed that pentaploids suffer from a severely reduced paternal reproductive fitness. Any additional hybrids between tetraploids and pentaploids, as would be created by mixing populations with different genome copy numbers during conservation work, would consequently exacerbate mate limitation and thus reduce population viability. We conclude that seed set and thus population viability can be maximized by mixing populations with the same number of genome copies, but that populations with different numbers should be kept spatially separated. The case of Rutidosis lanata provides an example and a potential template for examining the conservation genetics of other species that may constitute polyploid complexes. | NA | no | No good fitness measure | NA | no | no |
| 5593 | 2020 | 44112 | Schrandt, M; Powers, S; Rikard, FS; Thongda, W; Peatman, E | 2018 | Short-term low salinity mitigates effects of oil and dispersant on juvenile eastern oysters: A laboratory experiment with implications for oil spill response activities | Following the Deepwater Horizon oil spill, eastern oyster (Crassostrea virginica) reefs in the northern Gulf of Mexico were exposed to oil and various associated clean-up activities that may have compromised oyster reef health. Included in the exposure was oil, dispersant, and in some locales, atypical salinity regimes. Oil and dispersants can be detrimental to oysters and the effects of salinity depend on the level. In addition to these extrinsic factors, genetic diversity of oyster populations may help the oysters respond to stressors, as demonstrated in other systems. We used a 3x3x2 factorial design to experimentally examine the effects of oil/dispersed oil, intraspecific genetic diversity, and salinity on juvenile (ca. 25 mm shell height) oyster survivorship and growth during a 21-d exposure in a closed, recirculating system. The genetic effect was weak overall, oil and dispersed oil negatively affected juvenile oyster survivorship, and low salinity mitigated mortality in oil and dispersed oil treatments. Survivorship was about 40% greater in low-salinity than in mesohaline water for both oil and dispersed oil treatments, bringing survivorship in low salinity oil-only treatments to a similar level with low salinity controls (no oil). Oyster growth was minimal after 21 d but appeared to be negatively affected by oil and dispersed oil, and had a significant interaction with salinity. Our results may be informative for future decisions regarding oil spill response activities and suggest that a pulse of low salinity water may be a viable short-term mitigation option for oysters if filtration characteristics, exposure time, and water temperatures are all considered, in addition to weighing the costs and benefits of this type of response on other organisms and habitats. | NA | no | no good gd measure host pop | NA | no | no |
| 5594 | 2020 | 44043 | Schroeder, JW; Martin, JT; Angulo, DF; Arias-Del Razo, I; Barbosa, JM; Perea, R; Sebastian-Gonzalez, E; Dirzo, R | 2019 | Host plant phylogeny and abundance predict root-associated fungal community composition and diversity of mutualists and pathogens | Interactions between plants and their root-associated fungi (RAF) may influence the relative abundance of tree species and determine forest community diversity. Such plant-soil feedbacks in turn depend on the degree to which spatial distance and phylogenetic relatedness of host trees structure pathogen and mutualist communities, but research detailing these aspects of RAF communities is lacking. Here, we characterize plant-RAF associations across a diverse plant community, focusing on the degree to which RAF communities are structured by spatial distance, host phylogenetic relatedness, and host abundance. We compare results for different functional groups, including both putative mutualists and pathogens, an aspect poorly examined hitherto. We collected roots at regular intervals along ten 50 m by 2 m transects, then used DNA barcoding to identify host plants, and characterize the associated fungal community. Variance partitioning was used to measure the relative contributions of host phylogenetic relatedness and spatial distance to explaining RAF community composition. A weighted linear regression was used to measure the correlation between host abundance and RAF diversity. Phylogenetic distance among hosts was a better predictor of RAF community composition than spatial distance, but this relationship was stronger for putative pathogens than for mutualists, suggesting that pathogens show stronger host preference than mutualists. Across all functional groups, RAF showed similar levels of spatial structure. Additionally, RAF communities of locally abundant plants were less diverse than RAF communities of rare plants. Synthesis. We found that RAF communities are structured by the phylogenetic relatedness of hosts and, to a lesser extent, by spatial distance, with pathogens showing stronger host preference than mutualists. Abundant hosts had less diverse RAF communities than rare hosts, which is notable because abundant plants tend to experience weaker negative plant-soil feedback. Going forward, mechanisms underlying the host abundance-RAF diversity relationship warrant further investigation. Additionally, the survey approach presented here could be paired with experiments linking RAF community composition to plant recruitment. | NA | no | No good fitness measure and measure host GD | NA | no | no |
| 5595 | 2020 | 44113 | Schuler, KL; Baker, BB; Mayer, KA; Perez-Heydrich, C; Holahan, PM; Thomas, NJ; White, CL | 2018 | Refining Aging Criteria for Northern Sea Otters in Washington State | Measurement of skull ossification patterns is a standard method for aging various mammalian species and has been used to age sea otters Enhydra lutris from Russia, California, and Alaska. Cementum annuli counts have also been verified as an accurate aging method for sea otters in Alaska. In this study, we compared cementum annuli count results and skull ossification patterns as methods for aging the northern sea otter, E. I. kenyoni, in Washington State. We found significant agreement between the two methods, suggesting that either method could be used to age sea otters in Washington. We found that ossification of the squamosal-jugal suture at the ventral glenoid fossa can be used to differentiate male subadults from adults. To assist field biologists or others without access to cementum annuli or skull ossification analysis techniques, we analyzed a suite of morphologic, physiologic, and developmental characteristics to assess whether a set of these more easily accessible parameters could also predict age class. We identified tooth condition score, evidence of reproductive activity in females, and tooth eruption pattern as the most useful criteria for classifying sea otters in Washington. We created a simple decision tree based on characteristics accessible in the field or at necropsy, which can be used to reliably predict age class of Washington sea otters as determined by cementum annuli. These techniques offer field biologists and marine mammal stranding networks a replicable, cost-conscious methodology to gather useful biological information from sea otters. | NA | no | No good fitness measure and measure host GD | NA | no | no |
| 5596 | 2020 | 44039 | Schultz, AJ; Cristescu, RH; Hanger, J; Loader, J; de Villiers, D; Frere, CH | 2020 | Inbreeding and disease avoidance in a free-ranging koala population | Habitat destruction and fragmentation are increasing globally, forcing surviving species into small, isolated populations. Isolated populations typically experience heightened inbreeding risk and associated inbreeding depression and population decline; although individuals in these populations may mitigate these risks through inbreeding avoidance strategies. For koalas, as dietary specialists already under threat in the northern parts of their range, increased habitat fragmentation and associated inbreeding costs are of great conservation concern. Koalas are known to display passive inbreeding avoidance through sex-biased dispersal, although population isolation will reduce dispersal pathways. We tested whether free-ranging koalas display active inbreeding avoidance behaviours. We used VHF tracking data, parentage reconstruction, and veterinary examination results to test whether free-ranging female koalas avoid mating with (a) more closely related males; and (b) males infected with sexually transmittedChlamydia pecorum. We found no evidence that female koalas avoid mating with relatively more related available mates. In fact, as the relatedness of potential mates increases, so did inbreeding events. We also found no evidence that female koalas can avoid mating with males infected withC. pecorum. The absence of active inbreeding avoidance mechanisms in koalas is concerning from a conservation perspective, as small, isolated populations may be at even higher risk of inbreeding depression than expected. At risk koala populations may require urgent conservation interventions to augment gene flow and reduce inbreeding risks. Similarly, if koalas are not avoiding mating with individuals with chlamydial disease, populations may be at higher risk from disease than anticipated, further impacting population viability. | NA | no | No good fitness measure and measure host GD and inbreeding | NA | no | no |
| 5597 | 2020 | 44113 | Schultz, AJ; Cristescu, RH; Littleford-Colquhoun, BL; Jaccoud, D; Frere, CH | 2018 | Fresh is best: Accurate SNP genotyping from koala scats | Maintaining genetic diversity is a crucial component in conserving threatened species. For the iconic Australian koala, there is little genetic information on wild populations that is not either skewed by biased sampling methods (e.g., sampling effort skewed toward urban areas) or of limited usefulness due to low numbers of microsatellites used. The ability to genotype DNA extracted from koala scats using next-generation sequencing technology will not only help resolve location sample bias but also improve the accuracy and scope of genetic analyses (e.g., neutral vs. adaptive genetic diversity, inbreeding, and effective population size). Here, we present the successful SNP genotyping (1272 SNP loci) of koala DNA extracted from scat, using a proprietary DArTseq protocol. We compare genotype results from two-day-old scat DNA and 14-day-old scat DNA to a blood DNA template, to test accuracy of scat genotyping. We find that DNA from fresher scat results in fewer loci with missing information than DNA from older scat; however, 14-day-old scat can still provide useful genetic information, depending on the research question. We also find that a subset of 209 conserved loci can accurately identify individual koalas, even from older scat samples. In addition, we find that DNA sequences identified from scat samples through the DArTseq process can provide genetic identification of koala diet species, bacterial and viral pathogens, and parasitic organisms. | NA | no | No good fitness measure and measure host GD | NA | no | no |
| 5598 | 2020 | 44043 | Schunter, C; Pascual, M; Raventos, N; Garriga, J; Garza, JC; Bartumeus, F; Macpherson, E | 2019 | A novel integrative approach elucidates fine-scale dispersal patchiness in marine populations | Dispersal is one of the main determining factors of population structure. In the marine habitat, well-connected populations with large numbers of reproducing individuals are common but even so population structure can exist on a small-scale. Variation in dispersal patterns between populations or over time is often associated to geographic distance or changing oceanographic barriers. Consequently, detecting structure and variation in dispersal on a fine-scale within marine populations still remains a challenge. Here we propose and use a novel approach of combining a clustering model, early-life history trait information from fish otoliths, spatial coordinates and genetic markers to detect very fine-scale dispersal patterns. We collected 1573 individuals (946 adults and 627 juveniles) of the black-faced blenny across a small-scale (2 km) coastline as well as at a larger-scale area (< 50 kms). A total of 178 single nucleotide polymorphism markers were used to evaluate relatedness patterns within this well-connected population. In our clustering models we categorized SHORT-range dispersers to be potential local recruits based on their high relatedness within and low relatedness towards other spatial clusters. Local retention and/or dispersal of this potential local recruitment varied across the 2 km coastline with higher frequency of SHORT-range dispersers towards the southwest of the area for adults. An inverse pattern was found for juveniles, showing an increase of SHORT-range dispersers towards the northeast. As we rule out selective movement and mortality from one year to the next, this pattern reveals a complex but not full genetic mixing, and variability in coastal circulation is most likely the main driver of this fine-scale chaotic genetic patchiness within this otherwise homogeneous population. When focusing on the patterns within one recruitment season, we found large differences in temperatures (from approx. 17 degrees C to 25 degrees C) as well as pelagic larval duration (PLD) for juveniles from the beginning of the season and the end of the season. We were able to detect fine-scale differences in LONG-range juvenile dispersers, representing distant migrants, depending on whether they were born at the beginning of the season with a longer PLD, or at the end of the reproductive season. The ability to detect such fine-scale dispersal patchiness will aid in our understanding of the underlying mechanisms of population structuring and chaotic patchiness in a wide range of species even with high potential dispersal abilities. | NA | no | No good fitness measure and measure host GD | NA | no | no |
| 5599 | 2020 | 44112 | Schurink, A; da Silva, VH; Velie, BD; Dibbits, BW; Crooijmans, RPMA; Francois, L; Janssens, S; Stinckens, A; Blott, S; Buys, N; Lindgren, G; Ducro, BJ | 2018 | Copy number variations in Friesian horses and genetic risk factors for insect bite hypersensitivity | Background: Many common and relevant diseases affecting equine welfare have yet to be tested regarding structural variants such as copy number variations (CNVs). CNVs make up a substantial proportion of total genetic variability in populations of many species, resulting in more sequence differences between individuals than SNPs. Associations between CNVs and disease phenotypes have been established in several species, but equine CNV studies have been limited. Aim of this study was to identify CNVs and to perform a genome-wide association (GWA) study in Friesian horses to identify genomic loci associated with insect bite hypersensitivity (IBH), a common seasonal allergic dermatitis observed in many horse breeds worldwide. Results: Genotypes were obtained using the Axiom (R) Equine Genotyping Array containing 670,796 SNPs. After quality control of genotypes, 15,041 CNVs and 5350 CNV regions (CNVRs) were identified in 222 Friesian horses. Coverage of the total genome by CNVRs was 11.2% with 49.2% of CNVRs containing genes. 58.0% of CNVRs were novel (i.e. so far only identified in Friesian horses). A SNP- and CNV-based GWA analysis was performed, where about half of the horses were affected by IBH. The SNP-based analysis showed a highly significant association between the MHC region on ECA20 and IBH in Friesian horses. Associations between the MHC region on ECA20 and IBH were also detected based on the CNV-based analysis. However, CNVs associated with IBH in Friesian horses were not often in close proximity to SNPs identified to be associated with IBH. Conclusions: CNVs were identified in a large sample of the Friesian horse population, thereby contributing to our knowledge on CNVs in horses and facilitating our understanding of the equine genome and its phenotypic expression. A clear association was identified between the MHC region on ECA20 and IBH in Friesian horses based on both SNP- and CNV-based GWA studies. These results imply that MHC contributes to IBH sensitivity in Friesian horses. Although subsequent analyses are needed for verification, nucleotide differences, as well as more complex structural variations like CNVs, seem to contribute to IBH sensitivity. IBH should be considered as a common disease with a complex genomic architecture. | NA | no | dometicated host | NA | no | no |
| 5600 | 2020 | 44040 | Schwantes, JB; Quevedo, P; D’Avila, MF; Molento, MB; Graichen, DAS | 2020 | Fasciola hepatica in Brazil: genetic diversity provides insights into its origin and geographic dispersion | Fasciola hepatica is a trematode parasite that affects mammals, including humans. In Brazil, fascioliasis, a disease caused by the parasite, is of great importance. The disorder affects the welfare of the Brazilian population through impairing the agricultural production of cattle, where the disease causes weight loss as a result of liver damage. This study aimed to evaluate the genetic diversity of F. hepatica throughout Southern Brazil to determine its geographic origin and estimate the colonization route of the parasite. To accomplish these aims, flukes were collected from slaughterhouses in three endemic areas of Rio Grande do Sul and Parana states. DNA was isolated using the phenol-chloroform protocol from single flukes and two mitochondrial genes, cytochrome oxidase subunit I (COI) and nicotinamide dehydrogenase subunit 1 (Nad1), were amplified and sequenced. Ten haplotypes of COI were found from 75 isolated parasites and the total haplotype and nucleotide diversity observed were 0.475 and 0.002, respectively. Using the Nad1 gene, we found 24 haplotypes from 79 samples, resulting in haplotype and nucleotide diversity values of 0.756 and 0.004, respectively. An analysis of molecular variance showed that 57.4% and 77.5% of variation was within populations (F-ST), while 9.0 and 36.8% of variation was among groups (F-CT) when considering COI and Nad1 genes, respectively. For COI, the fixation index values of 0.425 and 0.368 were obtained for F-ST and F-CT, respectively, while analysis of Nad1 0.225 and 0.089 index values were obtained for F-ST and F-CT, respectively. We have determined that F. hepatica found in the two distinct areas originated from several geographical regions, since we found haplotypes that were shared with at least three different continents. These data are in accordance with the recent colonization of Brazil, and the recent import of cattle from South American, European and, possibly, some African countries. The observed F-ST and F-CT values for COI and Nad1 genes of F. hepatica may be a result of limited movement of animals within states and support the lack of geographical structure of the parasite in Brazil, which are in agreement with the observed cattle production systems in this region. | NA | no | gd of pathogen | NA | no | no |
| 5601 | 2020 | 44039 | Scribano, G; Balestrieri, A; Gazzola, A; Pellitteri-Rosa, D | 2020 | Strong behavioural defensive responses of endemic Rana latastei tadpoles induced by a native predator’s odour | Prey species must constantly acquire information on predator identity, abundance and dangerousness from the environment. In aquatic habitats, this information is mainly propagated by water-borne chemical signals, either predator-specific odours or prey alarm cues. Anuran larvae innately respond to conspecific alarm cues and are able to associate them to predator cues during their lifetime. In this study, we investigated the anti-predatory responses of endemic Italian agile frog (Rana latastei) tadpoles exposed to either conspecific or heterospecific alarm cues and a native predator’s (Anax imperator larvae) odour. Pre-and post-stimulus behaviours of each tadpole were recorded by a digital camera and analysed by a source executable software for image-based tracking. We found that Italian agile frog tadpoles responded to fasted dragonfly odour by strongly reducing their activity, both in terms of the amount of time they spent active and path length covered in comparison to control groups. Contrary to previous studies, predators’ diet had a negligible effect on tadpole response and our experiment did not bring any evidence of the phylogenetic-relatedness hypothesis. The innate or early-in-development recognition of dragonfly larvae is clearly adaptive and may increase tadpole survival with relatively low costs, but, at the same time, may increase the risk of ignoring novel potential threats. | NA | no | No good fitness measure and measure host GD | NA | no | no |
| 5602 | 2020 | 44042 | Sedaghatkish, A; Gossen, BD; Yu, FQ; Torkamaneh, D; McDonald, MR | 2019 | Whole-genome DNA similarity and population structure of Plasmodiophora brassicae strains from Canada | Background Clubroot is an important disease of brassica crops world-wide. The causal agent, Plasmodiophora brassicae, has been present in Canada for over a century but was first identified on canola (Brassica napus) in Alberta, Canada in 2003. Genetic resistance to clubroot in an adapted canola cultivar has been available since 2009, but resistance breakdown was detected in 2013 and new pathotypes are increasing rapidly. Information on genetic similarity among pathogen populations across Canada could be useful in estimating the genetic variation in pathogen populations, predicting the effect of subsequent selection pressure on changes in the pathogen population over time, and even in identifying the origin of the initial pathogen introduction to canola in Alberta. Results The genomic sequences of 43 strains (34 field collections, 9 single-spore isolates) of P. brassicae from Canada, the United States, and China clustered into five clades based on SNP similarity. The strains from Canada separated into four clades, with two containing mostly strains from the Prairies (provinces of Alberta, Saskatchewan, and Manitoba) and two that were mostly from the rest of Canada or the USA. Several strains from China formed a separate clade. More than one pathotype and host were present in all four Canadian clades. The initial pathotypes from canola on the Prairies clustered separately from the pathotypes on canola that could overcome resistance to the initial pathotypes. Similarly, at one site in central Canada where resistance had broken down, about half of the genes differed (based on SNPs) between strains before and after the breakdown. Conclusion Clustering based on genome-wide DNA sequencing demonstrated that the initial pathotypes on canola on the Prairies clustered separately from the new virulent pathotypes on the Prairies. Analysis indicated that these ‘new’ pathotypes were likely present in the pathogen population at very low frequency, maintained through balancing selection, and increased rapidly in response to selection from repeated exposure to host resistance. | NA | no | gd of pathogen | NA | no | no |
| 5603 | 2020 | 44111 | Sekamatte, M; Riad, MH; Tekleghiorghis, T; Linthicum, KJ; Britch, SC; Richt, JA; Gonzalez, JP; Scoglio, CM | 2019 | Individual-based network model for Rift Valley fever in Kabale District, Uganda | Rift Valley fever (RVF) is a zoonotic disease, that causes significant morbidity and mortality among ungulate livestock and humans in endemic regions. In East Africa, the causative agent of the disease is Rift Valley fever virus (RVFV) which is primarily transmitted by multiple mosquito species in Aedes and Mansonia genera during both epizootic and enzootic periods in a complex transmission cycle largely driven by environmental and climatic factors. However, recent RVFV activity in Uganda demonstrated the capability of the virus to spread into new regions through livestock movements, and underscored the need to develop effective mitigation strategies to reduce transmission and prevent spread among cattle populations. We simulated RVFV transmission among cows in 22 different locations of the Kabale District in Uganda using real world livestock data in a network-based model. This model considered livestock as a spatially explicit factor in different locations subjected to specific vector and environmental factors, and was configured to investigate and quantitatively evaluate the relative impacts of mosquito control, livestock movement, and diversity in cattle populations on the spread of the RVF epizootic. We concluded that cattle movement should be restricted for periods of high mosquito abundance to control epizootic spreading among locations during an RVF outbreak. Importantly, simulation results also showed that cattle populations with heterogeneous genetic diversity as crossbreeds were less susceptible to infection compared to homogenous cattle populations. | NA | no | No good fitness measure and measure host GD | NA | no | no |
| 5604 | 2020 | 44040 | Semerikova, SA; Isakov, IY; Semerikov, VL | 2020 | Chloroplast DNA Variation Shed Light on the History of Lime Tree (Tilia cordata s. l.) in the Eastern Part of the Range | The Quaternary history of European nemoral forests in the east of their distribution may differ significantly from the typical dynamics of populations and ranges of most deciduous species in Western and Central Europe, characterized by survival in the Mediterranean refugia in glacial phases and recolonization in the interglacials. This study focuses on the phylogeography of the small-leaved lime Tilia cordata Mill. s. s. (Malvaceae) and related taxa in Eastern Europe, the Urals, Siberia, and the Crimea. The variability of five chloroplast DNA fragments (CDt, HKt, DT, K1K2, and psbJ-petA) was studied in 29 populations using restriction analysis and sequencing. The deep divergence of the six identified haplotypes and poorly supported topology of the phylogenetic tree presumably correspond to the long existence of T. cordata in several areas of the western Palaearctic isolated from each other. The two haplotypes dominate on the Russian Plain, in the Urals, and in Siberia. Their comparison with the data of other authors shows their absence in Western and Central Europe. Several haplotypes found in western Russia, in Belarus, and in Western Ukraine correspond to haplotypes previously identified in Central Europe and the Carpathians. We assume that such a distribution of chloroplast DNA variability is due to the preservation of lime in the refugia in the east of the range during one or several of the last glacial intervals and recolonization in the Late Glacial on the Russian Plain. Comparison of the results of the study of the variability of chloroplast DNA in T. cordata s. l. and the results of the study of nuclear microsatellites suggest that the species that lived in the Urals and the Russian Plain in the Pleistocene is a Siberian lime (T. sibirica), later displaced by the European small-leaved lime and now occurring only in Southern Siberia. | NA | no | No good fitness measure and measure host GD | NA | no | no |
| 5605 | 2020 | 44113 | Semizer-Cuming, D; Kjaer, ED; Finkeldey, R | 2017 | Gene flow of common ash (Fraxinus excelsior L.) in a fragmented landscape | Gene flow dynamics of common ash (Fraxinus excelsior L.) is affected by several human activities in Central Europe, including habitat fragmentation, agroforestry expansion, controlled and uncontrolled transfer of reproductive material, and a recently introduced emerging infectious disease, ash dieback, caused by Hymenoscyphus fraxineus. Habitat fragmentation may alter genetic connectivity and effective population size, leading to loss of genetic diversity and increased inbreeding in ash populations. Gene flow from cultivated trees in landscapes close to their native counterparts may also influence the adaptability of future generations. The devastating effects of ash dieback have already been observed in both natural and managed populations in continental Europe. However, potential long-term effects of genetic bottlenecks depend on gene flow across fragmented landscapes. For this reason, we studied the genetic connectivity of ash trees in an isolated forest patch of a fragmented landscape in Rosenbeck, Germany. We applied two approaches to parentage analysis to estimate gene flow patterns at the study site. We specifically investigated the presence of background pollination at the landscape level and the degree of genetic isolation between native and cultivated trees. Local meteorological data was utilized to understand the effect of wind on the pollen and seed dispersal patterns. Gender information of the adult trees was considered for calculating the dispersal distances. We found that the majority of the studied seeds (55-64%) and seedlings (75-98%) in the forest patch were fathered and mothered by the trees within the same patch. However, we determined a considerable amount of pollen flow (260-45%) from outside of the study site, representing background pollination at the landscape level. Limited pollen flow was observed from neighbouring cultivated trees (2%). Both pollen and seeds were dispersed in all directions in accordance with the local wind directions. Whereas there was no positive correlation between pollen dispersal distance and wind speed, the correlation between seed dispersal distance and wind speed was significant (0.71, p < 0.001), indicating that strong wind favours long-distance dispersal of ash seeds. Finally, we discussed the implications of establishing gene conservation stands and the use of enrichment planting in the face of ash dieback. | NA | no | No good fitness measure and measure host GD | NA | no | no |
| 5606 | 2020 | 44043 | Semlali, A; Al Mutairi, M; Alanazi, IO; Aljohi, HA; Parine, NR; Alhadheq, A; Al-Jafari, AA; Mobeirek, AF; Al Amri, A; Shaik, JP; Filali, FZ; Alanazi, M | 2019 | Toll-like receptor 4 polymorphisms in Saudi population with cardiovascular diseases | Background Toll-like receptors play a substantial role in innate immunity and the effects of TLR4 genetic variants on cardiovascular diseases are still largely unknown. Therefore, we aimed to investigate the effects of TLR4 polymorphisms on cardiovascular diseases risk in the Saudi population. Methods Three tag single-nucleotide polymorphisms (rs2770150, rs10759931, and rs4986790) in TLR4 were studied on 222 unrelated patients with cardiovascular diseases and 190 healthy volunteers. Results We found that, in patients over 60 years old, the frequency of the TT genotype in rs2770150 and the variant allele G in rs10759931 were higher compared to the control group. Based on gender, the genotype frequency of rs2770150 increases the risk for cardiovascular diseases in female patients by 3.6-fold. The allele frequency for the G allele of rs10759931 increased the risk for CVDs in male patients by more than 1.5-fold. Furthermore, the genotype frequency of rs2770150 had a significant association with cardiovascular diseases in patients without hypertension and G allele of rs10759931 significantly increased the risk of cardiovascular diseases in patients that smoked. After Bonferroni correction only patients without hypertension showed significant risk of CVD with rs2770150. Conclusion A deeper understanding of the genetic variability of TLR4 will enable us to better identification of biomarkers for early detection and prognosis, and also enhance the decision-making process of treatments for cardiovascular diseases. | NA | no | human | NA | no | no |
| 5607 | 2020 | 44040 | Semo, A; Gaya-Vidal, M; Fortes-Lima, C; Alard, B; Oliveira, S; Almeida, J; Prista, A; Damasceno, A; Fehn, AM; Schlebusch, C; Rocha, J | 2020 | Along the Indian Ocean Coast: Genomic Variation in Mozambique Provides New Insights into the Bantu Expansion | The Bantu expansion, which started in West Central Africa around 5,000 BP, constitutes a major migratory movement involving the joint spread of peoples and languages across sub-Saharan Africa. Despite the rich linguistic and archaeological evidence available, the genetic relationships between different Bantu-speaking populations and the migratory routes they followed during various phases of the expansion remain poorly understood. Here, we analyze the genetic profiles of southwestern and southeastern Bantu-speaking peoples located at the edges of the Bantu expansion by generating genome-wide data for 200 individuals from 12 Mozambican and 3 Angolan populations using similar to 1.9 million autosomal single nucleotide polymorphisms. Incorporating a wide range of available genetic data, our analyses confirm previous results favoring a late split between West and East Bantu speakers, following a joint passage through the rainforest. In addition, we find that Bantu speakers from eastern Africa display genetic substructure, with Mozambican populations forming a gradient of relatedness along a North-South cline stretching from the coastal border between Kenya and Tanzania to South Africa. This gradient is further associated with a southward increase in genetic homogeneity, and involved minimum admixture with resident populations. Together, our results provide the first genetic evidence in support of a rapid North-South dispersal of Bantu peoples along the Indian Ocean Coast, as inferred from the distribution and antiquity of Early Iron Age assemblages associated with the Kwale archaeological tradition. | NA | no | human | NA | no | no |
| 5608 | 2020 | 44111 | Serna-Dominguez, MG; Andrade-Michel, GY; Rosas-Valdez, R; Castro-Felix, P; Arredondo-Bernal, HC; Gallou, A | 2019 | High genetic diversity of the entomopathogenic fungus Beauveria bassiana in Colima, Mexico | The entomopathogenic fungus Beauveria bassiana is used widely as a biological control agent against a wide range of insect pests globally. In this study, 44 Beauveria isolates from the state of Colima, Mexico harbored in the Coleccion de Hongos EntomopatOgenos of the Centro Nacional de Referencia de Control Biologico and from different substrates, insect-hosts, and localities were characterized with molecular markers. All isolates were identified using a Bayesian phylogenetic analysis of translation elongation factor 1-alpha (TEF) and nuclear intergenic Bloc region. Forty-three isolates were identified as B. bassiana and grouped into two sub-clades, i.e., AFNEO\_1 (n = 22; previously defined as a Glade with African and Neotropical origin) and Bb Glade (n = 21; closely associated with ex-type strain ARSEF 1564), and one isolate was identified as B. pseudobassiana. The fixation index (F-ST = 0.493) established the genetic differentiation between AFNEO\_1 and Bb Glades. High genotype richness and genetic diversity of AFNEO\_1 and Bb clades were revealed in sequence analysis of Bloc region and SSR genotyping. Moreover, the AFNEO\_1 and Bb clades were confirmed as two independent clonally structured assemblages. Finally, the AMOVA detected no significant association between any combination of substrate, insect-host or geographical origin. High genetic variation of B. bassiana in Colima, Mexico could suggest a functional diversity among isolates that may include those effective against a specific insect pest. | NA | no | gd of pathogen | NA | no | no |
| 5609 | 2020 | 44113 | Sgarlata, GM; Salmona, J; Aleixo-Pais, I; Rakotonanahary, A; Sousa, AP; Kun-Rodrigues, C; Ralantoharijaona, T; Jan, F; Zaranaina, R; Rasolondraibe, E; Zaonarivelo, JR; Andriaholinirina, NV; Chikhi, L | 2018 | Genetic Differentiation and Demographic History of the Northern Rufous Mouse Lemur (Microcebus tavaratra) Across a Fragmented Landscape in Northern Madagascar | Phylogeographic barriers, together with habitat loss and fragmentation, contribute to the evolution of a species’ genetic diversity by limiting gene flow and increasing genetic differentiation among populations. Changes in connectivity can thus affect the genetic diversity of populations, which may influence the evolutionary potential of species and the survival of populations in the long term. We studied the genetic diversity of the little known Northern rufous mouse lemur (Microcebus tavaratra), endemic to Northern Madagascar. We focused on the population of M. tavaratra in the Loky-Manambato region, Northern Madagascar, a region delimited by two permanent rivers and characterized by a mosaic of fragmented forests. We genotyped 148 individuals at three mitochondrial loci (D-loop, cytb, and cox2) in all the major forests of the study region. Our analyses suggest that M. tavaratra holds average genetic diversity when compared to other mouse lemur species, and we identified two to four genetic clusters in the study region, a pattern similar to that observed in another lemur endemic to the region (Propithecus tattersalli). The main cluster involved samples from the two mountain forests in the study region, which were connected until recently. However, the river crossing the study region does not appear to be a strict barrier to gene flow in M. tavaratra. Finally, the inferred demographic history of M. tavaratra suggests no detectable departure from stationarity over the last millennia. Comparisons with codistributed species (P. tattersalli and two endemic rodents, Eliurus spp.) suggest both differences and similarities in the genetic clusters identified (i.e., barriers to species dispersal) and in the inferred demographic history. These comparisons suggest that studies of codistributed species are important to understand the effects of landscape features on species and to reconstruct the history of habitat changes in a region. | NA | no | No good fitness measure and measure host GD | NA | no | no |
| 5610 | 2020 | 44040 | Shah, SHJ; Paredes-Montero, JR; Malik, AH; Brown, JK; Qazi, J | 2020 | Distribution of Bemisia tabaci (Gennadius) (Hemiptera: Aleyrodidae) mitotypes in commercial cotton fields in the Punjab province of Pakistan | The Bemisia tabaci (Gennadius) (Hemiptera: Aleyroridae) sibling species group is comprised of genetic variants defined by biological differences or a fragment of mitochondrial cytochrome oxidase I gene sequence (mitotype) that allows for phylogeographic affiliation. Some mitotypes may cause damage to crop plants by feeding and transmission of plant viruses. In Pakistan, cotton-vegetable agroecosystems are vulnerable to whitefly-transmitted virus (genus Begomovirus; family Geminiviridae) infection. The identity and distribution of the whitefly B. tabaci mitotypes associated with the cotton crop were studied in 8 districts in the Punjab Province from 2014 to 2016. Phylogenetic analysis of the 3(sic)-fragment of the mitochondrial cytochrome oxidase I gene indicated the predominant haplotypes belonged to the Asia II-1 mitotype, with pairwise distances ranging from 0.15 to 3.2%. Pairwise distances showed that B. tabaci haplotype diversity varied by district, with the Khanewal harboring the highest divergence at 1.37%, compared to the lowest at 0.50% in the Dera Ghazi Khan district. The median-joining network analysis showed genetic expansion, or a ‘recovery’ trend, following the declining genetic diversity that occurred during the late 1990s to the early 2000s. The Asia II-1 mitotype group was the predominant whitefly vector species in Punjab Province. The haplotype network provides documentation of continued genetic expansion among the B. tabaci populations in the Punjab, which is consistent with previously reported trends among whiteflies sampled in the same or nearby districts from 2012 to 2014. Genetic expansion varied among districts and could be explained by factors unique to each district, i.e., management practices that influence B. tabaci mitotype composition, whitefly susceptibility to cotton leaf curl disease complex, and cotton genotype | NA | no | No good fitness measure | NA | no | no |
| 5611 | 2020 | 44113 | Sharma, G; Maymon, M; Freeman, S | 2017 | Epidemiology, pathology and identification of Colletotrichum including a novel species associated with avocado (Persea americana) anthracnose in Israel | Anthracnose disease caused by Colletotrichum species is a major constraint for the shelf-life and marketability of avocado fruits. To date, only C. gloeosporioides sensu lato and C. aenigma have been reported as pathogens affecting avocado in Israel. This study was conducted to identify and characterize Colletotrichum species associated with avocado anthracnose and to determine their survival on different host-structures in Israel. The pathogen survived and over-wintered mainly on fresh and dry leaves, as well as fresh twigs in the orchard. A collection of 538 Colletotrichum isolates used in this study was initially characterized based on morphology and banding patterns generated according to arbitrarily primed PCR to assess the genetic diversity of the fungal populations. Thereafter, based on multi-locus phylogenetic analyses involving combinations of ITS, act, ApMat, cal, chs1, gapdh, gs, his3, tub2 gene/markers; eight previously described species (C. aenigma, C. alienum, C. fructicola, C. gloeosporioides sensu stricto, C. karstii, C. nupharicola, C. siamense, C. theobromicola) and a novel species (C. perseae) were identified, as avocado anthracnose pathogens in Israel; and reconfirmed after pathogenicity assays. Colletotrichum perseae sp. nov. and teleomorph of C. aenigma are described along with comprehensive morphological descriptions and illustrations, for the first time in this study. | NA | no | No good fitness measure and measure host GD | NA | no | no |
| 5612 | 2020 | 44112 | Sharma, RS; Karmakar, S; Kumar, P; Mishra, V | 2019 | Application of filamentous phages in environment: A tectonic shift in the science and practice of ecorestoration | Theories in soil biology, such as plant-microbe interactions and microbial cooperation and antagonism, have guided the practice of ecological restoration (ecorestoration). Below-ground biodiversity (bacteria, fungi, invertebrates, etc.) influences the development of above-ground biodiversity (vegetation structure). The role of rhizosphere bacteria in plant growth has been largely investigated but the role of phages (bacterial viruses) has received a little attention. Below the ground, phages govern the ecology and evolution of microbial communities by affecting genetic diversity, host fitness, population dynamics, community composition, and nutrient cycling. However, few restoration efforts take into account the interactions between bacteria and phages. Unlike other phages, filamentous phages are highly specific, nonlethal, and influence host fitness in several ways, which make them useful as target bacterial inocula. Also, the ease with which filamentous phages can be genetically manipulated to express a desired peptide to track and control pathogens and contaminants makes them useful in biosensing. Based on ecology and biology of filamentous phages, we developed a hypothesis on the application of phages in environment to derive benefits at different levels of biological organization ranging from individual bacteria to ecosystem for ecorestoration. We examined the potential applications of filamentous phages in improving bacterial inocula to restore vegetation and to monitor changes in habitat during ecorestoration and, based on our results, recommend a reorientation of the existing framework of using microbial inocula for such restoration and monitoring. Because bacterial inocula and biomonitoring tools based on filamentous phages are likely to prove useful in developing cost-effective methods of restoring vegetation, we propose that filamentous phages be incorporated into nature-based restoration efforts and that the tripartite relationship between phages, bacteria, and plants be explored further. Possible impacts of filamentous phages on native microflora are discussed and future areas of research are suggested to preclude any potential risks associated with such an approach. | NA | no | No good fitness measure and measure host GD | NA | no | no |
| 5613 | 2020 | 44112 | Sharma, U; Gupta, S; Venkatesh, S; Rai, A; Dhariwal, AC; Husain, M | 2018 | Comparative genetic variability in HIV-1 subtype C p24 Gene in early age groups of infants | It is important to study the molecular properties of vertically transmitted viruses in early infancy to understand disease progression. P24 having an important role in virus assembly and maturation was selected to explore the genotypic characteristics. Blood samples, obtained from 82 HIV-1 positive infants, were categorized into acute (<= 6 months) and early (> 6-18 months) age groups. Of the 82 samples, 79 gave amplification results for p24, which were then sequenced and analysed. Amino acid heterogeneity analysis showed that substitutions were more frequent. Several substitution mutations were present in some of the sequences of both the age groups in the functional motifs of the gene namely Beta hairpin, CyPA binding loop, residues L136 and L190, linker region and major homology region. In the acute age group, an insertion of Asparagine residue (N5NL6) was observed in the beta hairpin region in one of the sequences. This insertion was accompanied with analogous substitutions of N5Q, Q7L and G8R. In the early age group, a deletion of two residues; VK181-182, was observed at the C-terminal end in one of the sequences. These mutations may impair the structure of the protein leading to defective virus assembly. Protein variation effect analyzer software showed that deleterious mutations were more in the acute than the early age group. Variability analysis revealed that the amino acid heterogeneity was comparatively higher in the acute than the early age group. Variability in the virus was decreasing with the increasing age of the infants indicating that the virus is gradually evolving under positive selection pressure. HLA class 1 binding peptide analysis showed that the epitopes TPQDLNTML and RMYSPVSIL may be helpful in designing epitope based vaccine. | NA | no | hiv | NA | no | no |
| 5614 | 2020 | 44039 | Sheehan, MJ; Reeve, HK | 2020 | Evolutionarily stable investments in recognition systems explain patterns of discrimination failure and success | Many animals are able to perform recognition feats that astound us-such as a rodent recognizing kin it has never met. Yet in other contexts, animals appear clueless as when reed warblers rear cuckoo chicks that bear no resemblance to their own species. Failures of recognition when it would seem adaptive have been especially puzzling. Here, we present a simple tug-of-war game theory model examining how individuals should optimally invest in affecting the accuracy of discrimination between desirable and undesirable recipients. In the game, discriminating individuals (operators) and desirable and undesirable recipients (targets and mimics, respectively) can all invest effort into their own preferred outcome. We demonstrate that stable inaccurate recognition will arise when undesirable recipients have large fitness gains from inaccurate recognition relative to the pay-offs that the other two parties receive from accurate recognition. The probability of accurate recognition is often determined by just the relative pay-offs to the desirable and undesirable recipients, rather than to the discriminator. Our results provide a new lens on long-standing puzzles including a lack of nepotism in social insect colonies, tolerance of brood parasites and male birds caring for extra-pair young in their nests, which our model suggests should often lack accurate discrimination. This article is part of the theme issue ‘Signal detection theory in recognition systems: from evolving models to experimental tests’. | NA | no | No good fitness measure and measure host GD | NA | no | no |
| 5615 | 2020 | 44112 | Shehzadiand, I; Naz, S | 2019 | MORPHOLOGICAL, BIOCHEMICAL AND GENETIC CHARACTERIZATION OF CITRUS CANKER PATHOGEN (XANTHOMONASAXONOPODIS) FROM CITRUS CULTIVARS OF PUNJAB, PAKISTAN | The objective of the present study was to identify citrus canker pathogen (Xanthomonas axonopodis) and disease severity among different citrus cultivars collected from citrus growing area (CRI) of Punjab, Pakistan. Morphological, biochemical and molecular markers characterized the pathogen. Total 95 samples were collected from grapefruit (C. paradis), kumquats (C. japonica) and lemon and limes (C. limon) and subjected to biochemical analysis by API strip. Three different primer sets targeting 16SrDNA and internally transcribed spacer region (ITS) were used to check genetic variability by PCR. The infectious pathogen colonies were found to be small, yellow, mucoid and gram negative by gram staining The oxidase, urease tests were negative and casesin hydrolysis, citrate utilization, methyl red test, catalase tests were positive for the pathogen via API strips. All the collected samples were positive for the disease by PCR and the disease severity was also measured by percentage mean. The highest disease severity (82%) was observed in grapefruits followed by lemon and limes (70%) while the lowest (30%) was in kumquats. The present results revealed that citrus cultivars have variability towards the susceptibility of citrus canker and phylogenetic analysis revealed that A type citrus canker is prevalent in selected region of Pakistan. | NA | no | gd of pathogen | NA | no | no |
| 5616 | 2020 | 44040 | Shelenga, TV; Malyshev, LL; Kerv, YA; Diubenko, TV; Konarev, AV; Horeva, VI; Belousova, MK; Kolesova, MA; Chikida, NN | 2020 | Metabolomic approach to search for fungal resistant forms of Aegilops tauschii Coss. from the VIR collection | Broadening of the genetic diversity of donors of resistance to biotic environmental factors is a challenging problem concerning Triticum L., which can be solved by using wild relatives of wheat, in particular, Aegilops tauschii Coss., in breeding programs. This species, believed to be the donor of D genome of common wheat (T. aestivum L.), is a source of some traits important for breeding. This greatly facilitates the possibility of crossing Ae. tauschii with common wheat. Aegilops L. species are donors of effective genes for resistance to fungal diseases in wheat. For instance, genes that determine resistance to rust agents in common wheat were successfully introgressed from Ae. tauschii into the genome of T. aestivum L. The aim of our study was to identify differences in metabolomic profiles of Ae. tauschii forms (genotypes), resistant or susceptible to such fungal pathogens as Puccinia triticina f. sp. tritici and Erysiphe graminis f. sp. tritici. These indicators may be used as biochemical markers of resistance. A comparative analysis of groups of Ae. tauschii accessions showed that metabolomic profiles of the forms with or without resistance to fungal pathogens differed significantly in the contents of nonproteinogenic amino acids, polyols, phyto-sterols, acylglycerols, mono- and oligosaccharides, glycosides, phenolic compounds (hydroquinone, kempferol), etc. This fact was consistent with the previously obtained data on the relationship between Fusarium resistance in oats (Avena sativa L.) and certain components of the metabolomic profile, such as acylglycerols, nonproteinogenic amino acids, galactinol, etc. Thus, our studies once again confirmed the possibility and effectiveness of the use of metabolomic analysis for screening the genetic diversity of accessions in the VIR collection, of Ae. tauschii in particular, in order to identify forms with a set of compounds in their metabolomic profile, which characterize them as resistant. Ae. tauschii accessions with a high content of pipecolic acids, acylglycerols, galactinol, stigma-sterol, glycerol, azelaic and pyrogallic acids, campesterol, hydroquinone, etc., can be used for creating wheat and triticale cultivars with high resistance to fungal pathogens causing powdery mildew, brown rust, and yellow rust. | NA | no | No good fitness measure and measure host GD | NA | no | no |
| 5617 | 2020 | 44112 | Shemesh, H; Shani, G; Carmel, Y; Kent, R; Sapir, Y | 2018 | To mix or not to mix the sources of relocated plants? The case of the endangered Iris lortetii | Active management of endangered species is required for the persistence of many rare species. Species trans location, a common practice used to mitigate the negative effects of small population sizes, entails risks of outbreeding depression due to dilution of local adaptations, and therefore must be weighed against the costs of a hands-off conservation approach. Iris lortetii is an endangered rhizomatous plant, growing in a small number of isolated populations in northern Israel. We implemented a quasi-in-situ reintroduction program by planting 234 rhizomes from diverse origins in five new sites. All new sites were selected to be as similar as possible to those of the largest natural population. We recorded plant survival and flowering in the field after four years. Flowering plants were artificially crossed either with plants from the same population of origin (within population) or with plants from different origin (between populations). We found no differences in survival between populations of origin and only some indication of local adaptations in the form of increased flowering of the local population. Nonetheless, seed set was significantly higher (a 73% increase) in crosses between populations of origin, compared to within-population crosses, suggesting low genetic diversity within the natural populations. The ability to combine active conservation with rigid testing of theoretical hypothesis, while avoiding all risk to natural populations, highlights the value of the quasi-in-situ approach for restoration. Our results indicate that, in the case of Iris lortetii, active relocation of genotypes, seeds or pollen can enhance the survival of natural populations over time. | NA | no | no good gd measure host pop | NA | no | no |
| 5618 | 2020 | 44040 | Sherman, RM; Salzberg, SL | 2020 | Pan-genomics in the human genome era | Since the early days of the genome era, the scientific community has relied on a single ‘reference’ genome for each species, which is used as the basis for a wide range of genetic analyses, including studies of variation within and across species. As sequencing costs have dropped, thousands of new genomes have been sequenced, and scientists have come to realize that a single reference genome is inadequate for many purposes. By sampling a diverse set of individuals, one can begin to assemble a pan-genome: a collection of all the DNA sequences that occur in a species. Here we review efforts to create pan-genomes for a range of species, from bacteria to humans, and we further consider the computational methods that have been proposed in order to capture, interpret and compare pan-genome data. As scientists continue to survey and catalogue the genomic variation across human populations and begin to assemble a human pan-genome, these efforts will increase our power to connect variation to human diversity, disease and beyond. Although single reference genomes are valuable resources, they do not capture genetic diversity among individuals. Sherman and Salzberg discuss the concept of ‘pan-genomes’, which are reference genomes that encompass the genetic variation within a given species. Focusing particularly on large eukaryotic pan-genomes, they describe the latest progress, the varied methodological approaches and computational challenges, as well as applications in fields such as agriculture and human disease. | NA | no | No good fitness measure and measure host GD | NA | no | no |
| 5619 | 2020 | 44113 | Sherpa, S; Rioux, D; Goindin, D; Fouque, F; Francois, O; Despres, L | 2018 | At the Origin of a Worldwide Invasion: Unraveling the Genetic Makeup of the Caribbean Bridgehead Populations of the Dengue Vector Aedes aegypti | Human-driven global environmental changes have considerably increased the risk of biological invasions, especially the spread of human parasites and their vectors. Among exotic species that have major impacts on public health, the dengue fever mosquito Aedes aegypti originating from Africa has spread worldwide during the last three centuries. Although considerable progress has been recently made in understanding the history of this invasion, the respective roles of human and abiotic factors in shaping patterns of genetic diversity remain largely unexplored. Using a genome-wide sample of genetic variants (3,530 ddRAD SNPs), we analyzed the genetic structure of Ae. aegypti populations in the Caribbean, the first introduced territories in the Americas. Fourteen populations were sampled in Guyane and in four islands of the Antilles that differ in climatic conditions, intensity of urbanization, and vector control history. The genetic diversity in the Caribbean was low (He = 0.14-0.17), as compared with a single African collection from Benin (He = 0.26) and site-frequency spectrum analysis detected an ancient bottleneck dating back similar to 300 years ago, supporting a founder event during the introduction of Ae. aegypti. Evidence for a more recent bottleneck may be related to the eradication program undertaken on the American continent in the 1950s. Among 12 loci detected as F-ST-outliers, two were located in candidate genes for insecticide resistance (cytochrome P450 and voltage-gated sodium channel). Genome-environment association tests identified additional loci associated with human density and/or delta-methrin resistance. Our results highlight the high impact of human pressures on the demographic history and genetic variation of Ae. aegypti Caribbean populations. | NA | no | No good fitness measure and measure host GD | NA | no | no |
| 5620 | 2020 | 44040 | Shimozuru, M; Shirane, Y; Jimbo, M; Yamanaka, M; Nakanishi, M; Ishinazaka, T; Kasai, S; Nose, T; Fujimoto, Y; Tsuruga, H; Mano, T; Tsubota, T | 2020 | Male reproductive input, breeding tenure, and turnover in high-density brown bear (Ursus arctos yesoensis) populations on the Shiretoko Peninsula, Hokkaido, Japan | Male reproductive behavior has not been thoroughly investigated in large, non-social mammals. We studied male reproductive input using field observations and microsatellite DNA data for the brown bear (Ursus arctos yesoensis Lydekker, 1897) population in a special wildlife protection area on the Shiretoko Peninsula in Hokkaido, Japan. We identified the father of 82 offspring born between 2006 and 2018 from 54 litters produced by 20 females and revealed that they were sired by a total of 19 males. Overall, paternity was distributed relatively evenly among different males, although litter production tended to be skewed to a few bears in some years. Male breeding tenure varied among individuals, but most tenures were within the range of 1 to 6 years. Human-caused mortality, mostly occurring near human residential areas, induced male turnover, indicating that the special wildlife protection area located in the inner part of the Shiretoko Peninsula is not free from human impact. The frequency of successful reproduction was highest in 10- to 14-year-old bears, which is consistent with the period when males reach physical maturity. These results contribute to an understanding of breeding systems in large solitary mammals and to appropriate conservation and management strategies for brown bear populations. | NA | no | No good fitness measure and measure host GD | NA | no | no |
| 5621 | 2020 | 44111 | Shockey, AC; Dabney, J; Pepperell, CS | 2019 | Effects of Host, Sample, and in vitro Culture on Genomic Diversity of Pathogenic Mycobacteria | Mycobacterium tuberculosis (M. tb), an obligate human pathogen and the etiological agent of tuberculosis (TB), remains a major threat to global public health. Comparative genomics has been invaluable for monitoring the emergence and spread of TB and for gaining insight into adaptation of M. tb. Most genomic studies of M. tb are based on single bacterial isolates that have been cultured for several weeks in vitro. However, in its natural human host, M. tb comprises complex, in some cases massive bacterial populations that diversify over the course of infection and cannot be wholly represented by a single genome. Recently, enrichment via hybridization capture has been used as a rapid diagnostic tool for TB, circumventing culturing protocols and enabling the recovery of M. tb genomes directly from sputum. This method has further applicability to the study of M. tb adaptation, as it enables a higher resolution and more direct analysis of M. tb genetic diversity within hosts with TB. Here we analyzed genomic material from M. tb and Mycobacterium bovis populations captured directly from sputum and from cultured samples using metagenomic and Pool-Seq approaches. We identified effects of sampling, patient, and sample type on bacterial genetic diversity. Bacterial genetic diversity was more variable and on average higher in sputum than in culture samples, suggesting that manipulation in the laboratory reshapes the bacterial population. Using outlier analyses, we identified candidate bacterial genetic loci mediating adaptation to these distinct environments. The study of M. tb in its natural human host is a powerful tool for illuminating host pathogen interactions and understanding the bacterial genetic underpinnings of virulence. | NA | no | gd of pathogen | NA | no | no |
| 5622 | 2020 | 44112 | Shwab, EK; Saraf, P; Zhu, XQ; Zhou, DH; McFerrin, BM; Ajzenberg, D; Schares, G; Hammond-Aryee, K; van Helden, P; Higgins, SA; Gerhold, RW; Rosenthal, BM; Zhao, XP; Dubey, JP; Su, CL | 2018 | Human impact on the diversity and virulence of the ubiquitous zoonotic parasite Toxoplasma gondii | A majority of emerging infectious diseases in humans are zoonoses. Understanding factors that influence the emergence and transmission of zoonoses is pivotal for their prevention and control. Toxoplasma gondii is one of the most widespread zoonotic pathogens known today. Whereas only a few genotypes of T. gondii dominate in the Northern Hemisphere, many genotypes coexist in South America. Furthermore, T. gondii strains from South America are more likely to be virulent than those from the Northern Hemisphere. However, it is not clear what factor(s) shaped modern-day genetic diversity and virulence of T. gondii. Here, our analysis suggests that the rise and expansion of farming in the past 11,000 years established the domestic cat/mouse transmission cycle for T. gondii, which has undoubtedly played a significant role in the selection of certain linages of T. gondii. Our mathematical simulations showed that within the domestic transmission cycle, intermediately mouse-virulent T. gondii genotypes have an adaptive advantage and eventually become dominant due to a balance between lower host mortality and the ability to superinfect mice previously infected with a less virulent T. gondii strain. Our analysis of the global type II lineage of T. gondii suggests its Old World origin but recent expansion in North America, which is likely the consequence of global human migration and trading. These results have significant implications concerning transmission and evolution of zoonotic pathogens in the rapidly expanding anthropized environment demanded by rapid growth of the human population and intensive international trading at present and in the future. | NA | no | No good fitness measure and measure host GD | NA | no | no |
| 5623 | 2020 | 44112 | Sigal, D; Reid, JNS; Wahl, LM | 2018 | Effects of Transmission Bottlenecks on the Diversity of Influenza A Virus | We investigate the fate of de novo mutations that occur during the in-host replication of a pathogenic virus, predicting the probability that such mutations are passed on during disease transmission to a new host. Using influenza A virus as a model organism, we develop a life-history model of the within-host dynamics of the infection, deriving a multitype branching process with a coupled deterministic model to capture the population of available target cells. We quantify the fate of neutral mutations and mutations affecting five life-history traits: clearance, attachment, budding, cell death, and eclipse phase timing. Despite the severity of disease transmission bottlenecks, our results suggest that in a single transmission event, several mutations that appeared de novo in the donor are likely to be transmitted to the recipient. Even in the absence of a selective advantage for these mutations, the sustained growth phase inherent in each disease transmission cycle generates genetic diversity that is not eliminated during the transmission bottleneck. | NA | no | No good fitness measure and measure host GD | NA | no | no |
| 5624 | 2020 | 44113 | Signor, S | 2017 | Population genomics of Wolbachia and mtDNA in Drosophila simulans from California | Wolbachia pipientis is an intracellular endosymbiont infecting many arthropods and filarial nematodes. Little is known about the short-term evolution of Wolbachia or its interaction with its host. Wolbachia is maternally inherited, resulting in co-inheritance of mitochondrial organelles such as mtDNA. Here I explore the evolution of Wolbachia, and the relationship between Wolbachia and mtDNA, using a large inbred panel of Drosophila simulans. I compare this to the only other large population genomic Wolbachia dataset from D. melanogaster. I find reduced diversity relative to expectation in both Wolbachia and mtDNA, but only mtDNA shows evidence of a recent selective sweep or population bottleneck. I estimate Wolbachia and mtDNA titre in each genotype, and I find considerable variation in both phenotypes, despite low genetic diversity in Wolbachia and mtDNA. A phylogeny of Wolbachia and of mtDNA suggest a recent origin of the infection derived from a single origin. Using Wolbachia and mtDNA titre as a phenotype, I perform the first association analysis using this phenotype with the nuclear genome and find several implicated regions, including one which contains four CAAX-box protein processing genes. CAAX-box protein processing can be an important part of host-pathogen interactions in other systems, suggesting interesting directions for future research. | NA | no | gd of pathogen | NA | no | no |
| 5625 | 2020 | 44113 | Silva, AF; Escada-Rebelo, S; Amaral, S; Tavares, RS; Schlatt, S; Ramalho-Santos, J; Mota, PC | 2018 | Can we induce spermatogenesis in the domestic cat using an in vitro tissue culture approach? | The reduced number of animals in most wild felid populations implies a loss of genetic diversity. The death of juveniles, prior to the production of mature sperm, represents a loss of potential genetic contribution to future populations. Since 2011 mouse testicular organ culture has introduced an alternative mechanism to produce sperm in vitro from immature tissue. However, extension of this technology to other species has remained limited. We have used the domestic cat (Felis catus) as a model for wild felids to investigate spermatogenesis initiation and regulation, with the mouse serving as a control species. Testicular tissue fragments were cultured in control medium or medium supplemented with knockout serum replacement (KSR), AlbuMax, beta-estradiol or AlbuMax plus beta-estradiol. Contrary to expectations, and unlike results obtained in mouse controls, no germ cell differentiation could be detected. The only germ cells observed after six weeks of culture were spermatogonia regardless of the initial stage of tubule development in the donor tissue. Moreover, the number of spermatogonia decreased with time in culture in all media tested, especially in the medium supplemented with KSR, while AlbuMax had a slight protective effect. The combination of AlbuMax and beta-estradiol led to an increase in the area occupied by seminiferous tubules, and thus to an increase in total number of spermatogonial cells. Considering all the media combinations tested the stimulus for felid germ cell differentiation in this type of system seems to be different from the mouse. Studies using other triggers of differentiation and tissue survival factors should be performed to pursue this technology for the genetic diversity preservation in wild felids. | NA | no | dometicated host | NA | no | no |
| 5626 | 2020 | 44113 | Silva, PHM; Brune, A; Pupin, S; Moraes, MLT; Sebbenn, AM; de Paula, RC | 2018 | Maintenance of genetic diversity in Eucalyptus urophylla S. T. Blake populations with restriction of the number of trees per family | Our aim was to verify the effect on gain and genetic diversity through the restriction of the number of trees per family in selection, in order to compose an elite population of Eucalyptus urophylla in two trials under distinct management levels. We studied 166 open-pollinated families of E. urophylla in Anhembi, Sao Paulo State, Brazil under commercial practices, and the same families in Selviria, Mato Grosso do Sul State, Brazil under lower management level (mainly no mineral fertilization). Mortality, height and diameter at breast height (DBH) were measured. DBH was analyzed by the REML/BLUP to select the best 25 trees, with four levels of tree restriction per family (no restriction; 1; 2 and 3 per family). We evaluated heritability; genetic gain and effective size of number of total and private alleles; observed and expected heterozygosity; coancestry and fixation index. A large difference in survival (48 and 83 %) and productivity (MAI of 26 and 44 m(3)ha(-1)y(-1)) was observed between trials due to the different levels of management applied. The highest restriction in number of individuals per family caused a small decrease in gain, corresponding to 7 % in the more productive trial and 3 % in the less productive one. Observed and expected heterozygosity, coancestry and fixation index were not significantly (lower than 5 %) affected by the restriction in both sites. The restriction of one tree per family allowed different alleles to be kept in the selected population and higher effective population size in order to insure variation for the next generations. | NA | no | No good fitness measure | NA | no | no |
| 5627 | 2020 | 44040 | Silveira, L; Garner, SR; Neff, BD | 2020 | Similarity at the major histocompatibility complex class II does not influence mating patterns in bluegill (Lepomis macrochirus) | In many species, individuals prefer mates that are genetically dissimilar at the major histocompatibility complex (MHC), likely because it improves offspring resistance to pathogens. Here, we provide the first characterization of the MHC class II peptide binding region in bluegill (Lepomis macrochirus) and examine its effect on mating patterns. We captured female and male bluegill during spawning and sequenced these fish at the MHC. We found strong evidence that positive selection promotes genetic diversity at the MHC in bluegill, with a 5:2 ratio of non-synonymous to synonymous mutations. However, we found no evidence that the MHC led to disassortative mating between females and parental males. Extra-pair mating and the presence of specialized cuckolder males may have an important, albeit still unresolved, role in shaping mating patterns at the MHC in bluegill. | NA | no | No group level | NA | no | no |
| 5628 | 2020 | 44113 | Silver, BP; Hudson, JM; Smith, CT; Lujan, K; Brown, M; Whitesel, TA | 2018 | An urban stream can support a healthy population of coastal cutthroat trout | Urbanization presents numerous challenges to aquatic species. For fish, typical threats include altered flow dynamics, poor water quality, and degraded food webs. These threats can be detrimental to fish and whether urban environments can support healthy populations is unclear. Tryon Creek is one of the largest urban watersheds in Portland, Oregon where developed land accounts for 55.6% of total land use. We used demographic, life history, disease, and genetic indices to assess the overall health of the Coastal Cutthroat Trout (Oncorhynchus clarkii) population in Tryon Creek. Results of these population indicators were within ranges for streams with Coastal Cutthroat Trout populations not influenced by urbanization and considered healthy. Coastal Cutthroat Trout in Tryon Creek exhibited a mean condition factor of 1.07 (95% CI 0.95-1.19, range 0.94-1.24), four size-classes, an estimated density of 0.01-0.03 individuals/m(2), and migratory behavior. The population did not exhibit excessive deviation from Hardy-Weinberg equilibrium. Genetic diversity in Tryon Creek (H-e = 0.76; allelic richness = 6.6) was comparable to that observed in non-urbanized streams in the Mt. Hood National Forest (H-e = 0.72; allelic richness = 5.6). Moreover, allele frequencies appeared stable, and no genetic divergence was detected among generations (F (ST) similar to 0.00; non-significant allele frequency heterogeneity tests). The population tested negative for 12 different pathogens and results for Renibacterium salmoninarum were inconclusive. The characteristics of Coastal Cutthroat Trout in Tryon Creek were similar to populations not influenced by urbanization which suggests an urban stream can support a healthy fish population. | NA | no | no good gd measure host pop | NA | no | no |
| 5629 | 2020 | 44111 | Simkanin, C; Carlton, JT; Steves, B; Fofonoff, P; Nelson, JC; Murray, CC; Ruiz, GM | 2019 | Exploring potential establishment of marine rafting species after transoceanic long-distance dispersal | Aim On 11 March 2011, the Great East Japan Earthquake triggered a massive tsunami that resulted in the largest known rafting event in recorded history. By spring 2012, marine debris began washing ashore along the Pacific coast of the United States and Canada with a wide range of Asian coastal species attached. We used this unique dataset, where the source region, date of dislodgment and landing location are known, to assess the potential for species invasions by transoceanic rafting on marine debris. Location Northeast Pacific from 20 to 60 degrees N. Time period Current. Major taxa studied Forty-eight invertebrate and algal species recorded on Japanese tsunami marine debris (JTMD). Methods We developed maximum entropy (MaxEnt) species distribution models for 48 species recorded on JTMD to predict establishment potential along the Pacific coast from 20 to 60 degrees N. Models were compared within the context of historical marine introductions from Japan to this region to validate the emergence of marine debris as a novel vector for species transfer. Results Overall, 27% (13 species) landed with debris at locations with suitable environmental conditions for establishment and survival, indicating that these species may be able to establish new populations or introduce greater genetic diversity to already established non-native populations. A further 21 species have an environmental match to areas where tsunami debris likely landed, but was not extensively sampled. Nearly 100 Japanese marine species previously invaded the northeastern Pacific, demonstrating this region’s environmental suitability for rafting Japanese biota. Historical invasions from Japan are highest in California and largely known from bays and harbours. Main conclusions Marine debris is a novel and growing vector for non-native species introduction. By utilizing a unique dataset of JTMD species, our predictive models show capacity for new transoceanic invasions and can focus monitoring priorities to detect successful long-distance dispersal across the world’s oceans. | NA | no | No good fitness measure and measure host GD | NA | no | no |
| 5630 | 2020 | 44113 | Simonis, JL; Harrison, RB; Long, ST; Rabon, DR; Waddell, WT; Faust, LJ | 2018 | Managed movement increases metapopulation viability of the endangered red wolf | Movement connects otherwise isolated populations, influencing demographic persistence and promoting gene flow. We evaluated the effect of movement on the genetic and demographic viability of the red wolf (Canis rufus) and tested the application of metapopulation theory to management of this endangered predator. The establishment of a captive subpopulation in the 1970s allowed persistence of the species and facilitated a reintroduction program that supported 2 wild subpopulations, one of which persists today. We assessed the effect of historical and potential future movement between the wild and captive subpopulations on the genetic and demographic viability of the metapopulation. We analyzed approximately 30 years of individual-level data to quantify the effects of historical movement among subpopulations and constructed an individual-based metapopulation model to predict the effects of future potential movement (i.e., releases of captive wolves) on the species’ persistence and genetic diversity. Counter to theory, increased movement has had positive demographic effects, with higher per capita movement rates leading to increased metapopulation growth and decreased subpopulation synchrony. These counter-theoretical results are likely due to differences in reproduction and survival rates among subpopulations and the small size of the metapopulation. Furthermore, higher rates of movement did not increase retention of genetic diversity, likely because of the active pedigree-based breeding management of the species already maximizing gene retention. Our model indicates that future releases of captive wolves are necessary, but not sufficient, for the survival of the species, and must be combined with changes to demographic rates in both the captive and northeastern North Carolina subpopulations. Our results highlight the need for models and field data that more adequately describe the viability of small metapopulations. (c) 2017 The Wildlife Society. Historical movement of red wolves between captive and wild subpopulations has promoted metapopulation viability and future releases of captive wolves to the wild subpopulation are necessary for the long-term viability of the red wolf metapopulation but must be combined with reductions in wild wolf mortality rates to have maximum effect. | NA | no | No good fitness measure and measure host GD | NA | no | no |
| 5631 | 2020 | 44039 | Singh, H; Kaur, J; Bala, R; Srivastava, P; Bains, NS | 2020 | Virulence and genetic diversity of Puccinia striiformis f. sp. tritici isolates in sub-mountainous area of Punjab, India | Information on the pathogen virulence profile and diversity across locations is crucial for host germplasm improvement and deployment. The rapid acquisition of virulence to host resistance by the wheat yellow/stripe rust pathogen (Puccinia striiformis f.sp. tritici: Pst), makes it crucial to know about its virulence and pathotype diversity. To assess the status of virulence and pathotype diversity in the sub-mountainous areas of Punjab, 27 stripe rust infected wheat samples from initial foci developed early in crop season were collected, multiplied and pathotyped using differential sets carrying known resistance genes. All the Pst isolates were virulent on lines carrying genes Yr2, Yr3, Yr4, Yr6, Yr7, Yr8, Yr9 and were avirulent on genes Yr1, Yr5, Yr10, Yr15 and Yr24/Yr26. Out of 27 isolates, only 4 pathotypes were detected indicating low pathotype diversity in Punjab. Two pathotypes i.e. 46S119 and 110S119 were most prevalent in Punjab. Other pathotypes recorded were; 238S119 and 110S84 but their prevalence was less. Three Pst pathotypes (46S119, 110S119 and 238S119) were tested for aggressiveness and it is found that 238S119 was more aggressive followed by 110S119 as spore production and uredial density was higher for this pathotype. Genetic diversity of the isolates was studied by using 74 SSR markers developed by different researchers. Scatter plot generated from the molecular data revealed the high genetic diversity among the isolates of Punjab. None of the pathotype showed any location specificity. Low pathotype diversity observed can be due to the cultivation of cultivars carrying only 1 or 2 major genes and high genetic diversity arised due to local adaptation, evolution for increased aggressiveness, mutation or parasexuality. | NA | no | Gd of pathogen | NA | no | no |
| 5632 | 2020 | 44042 | Singh, J; Khan, A | 2019 | Distinct patterns of natural selection determine sub-population structure in the fire blight pathogen, Erwinia amylovorad | The fire blight pathogen, Erwinia amylovora (EA), causes significant economic losses in rosaceae fruit crops. Recent genome sequencing efforts have explored genetic variation, population structure, and virulence levels in EA strains. However, the genomic aspects of population bottlenecks and selection pressure from geographical isolation, host range, and management practices are yet unexplored. We conducted a comprehensive analysis of whole genome sequences of 41 strains to study genetic diversity, population structure, and the nature of selection affecting sub-population differentiation in EA. We detected 72,741 SNPs and 2,500 Indels, representing about six-fold more diversity than previous reports. Moreover, nonsynonymous substitutions were identified across the effector regions, suggesting a role in defining virulence of specific strains. EA plasmids had more diversity than the chromosome sequence. Population structure analysis identified three distinct sub-groups in EA strains, with North American strains displaying highest genetic diversity. A five kilobase genomic window scan showed differences in genomic diversity and selection pressure between these three sub-groups. This analysis also highlighted the role of purifying and balancing selection in shaping EA genome structure. Our analysis provides novel insights into the genomic diversity and selection forces accompanying EA population differentiation. | NA | no | No good fitness measure and measure host GD | NA | no | no |
| 5633 | 2020 | 44113 | Singh, N; Mallick, J; Sagolsem, D; Mandal, N; Bhattacharyya, S | 2018 | Mapping of molecular markers linked with MYMIV and yield attributing traits in mungbean | The present study employed recombinant inbred lines (RILs) derived from the cross between a susceptible cultivar Sonali and resistant wild relative of mungbean (Vigna radiata var. sublobota to map molecular markers linked with mungbean yellow mosaic Indian virus (MYMIV) resistance and yield attributing traits in mungbean. Resistance to the virus was evaluated in RIL population under field conditions during two consecutive years 2013 and 2014. A set of 224 molecular markers were employed for the identification of polymorphism between parents. Only 46 markers showed polymorphism between Sonali and V. radiata var. sublobota. Twenty two polymorphic markers were used to construct a linkage map comprising 11 linkage groups. QTL analysis identified molecular markers linked with MYMIV resistance and agronomic traits viz., no. of pods per plant, no. of seeds per pod and 100-seed weight. Molecular markers identified to be linked with MYMIV were confirmed in 93 diverse mungbean accessions screened for yellow mosaic disease. The molecular markers linked to the MYMIV and yield attributing traits identified in this study will be useful in marker assisted breeding for development of high yielding mungbean varieties resistant to MYMIV. | NA | no | No good fitness measure and measure host GD | NA | no | no |
| 5634 | 2020 | 44113 | Singh, RK; Rai, N; Singh, AK; Kumar, P; Chaubey, T; Singh, B; Singh, SN | 2018 | Elucidation of diversity among F-1 hybrids to examine heterosis and genetic inheritance for horticultural traits and ToLCV resistance in tomato | Genetic diversity during prebreeding or postbreeding programme, is the key pillar to characterize the valuable traits and gene of interest. Whereas, superior or inferior heterotic performance of F-1 depend on the diverse nature of their pedigree. Therefore, the aim of this study was to see the diversity between the interspecific crosses and effect of heterosis, and inheritance for the morphological traits and ToLCV resistance. All the 24 F-1 interspecific crosses were classified into four clusters on the basis of morphological traits as well as simple sequence repeat (SSR) markers. Among the F-1 hybrids, 23 were grouped into clusters II, III and IV with different phylogeny, while PBC x EC 521080 was individual with cluster I. On the basis of visual observation of fruit colour, deep red and green colours in the crosses of S. pimpinellifolium (EC 521080) and S. habrochaites (EC 520061) exhibited dominant effects. In context of heterosis breeding, the crosses which were made using Solanum pimpinellifolium (EC 521080), S. chmielewskii (EC 520049) and S. cerasiforme (EC 528372) were better for yield capacity and the crosses of S. habrochaites (EC 520061) exhibited low and negative heterosis for ToLCV resistance. The F-2 progenies were segregated in various Mendelian ratio as follows 3:1, 1:2:1, 1:3, 13:3, 15:1, 12:3:1 and 9:6:1 for ToLCV disease reaction of incidence, plant growth habit and fruit colour appearance, respectively. Therefore, these interspecific crosses can be utilized for developing high yield, impressive fruit colour combiners and resistant hybrids/varieties of tomato. | NA | no | No good fitness measure and measure host GD | NA | no | no |
| 5635 | 2020 | 44112 | Siqueira, JD; Dominguez-Bello, MG; Contreras, M; Lander, O; Caballero-Arias, H; Deng, XT; Noya-Alarcon, O; Delwart, E | 2018 | Complex virome in feces from Amerindian children in isolated Amazonian villages | The number of viruses circulating in small isolated human populations may be reduced by viral extinctions and rare introductions. Here we used viral metagenomics to characterize the eukaryotic virome in feces from healthy children from a large urban center and from three Amerindian villages with minimal outside contact. Numerous human enteric viruses, mainly from the Picornaviridae and Caliciviridae families, were sequenced from each of the sites. Multiple children from the same villages shed closely related viruses reflecting frequent transmission clusters. Feces of isolated villagers also contained multiple viral genomes of unknown cellular origin from the Picornavirales order and CRESS-DNA group and higher levels of nematode and protozoan DNA. Despite cultural and geographic isolation, the diversity of enteric human viruses was therefore not reduced in these Amazonian villages. Frequent viral introductions and/or increased susceptibility to enteric infections may account for the complex fecal virome of Amerindian children in isolated villages. | NA | no | human | NA | no | no |
| 5636 | 2020 | 44112 | Sison-Mangus, MP; Metzger, CMJA; Ebert, D | 2018 | Host genotype-specific microbiota do not influence the susceptibility of D. magna to a bacterial pathogen | Host-associated microbiota have been claimed to play a role in hosts’ responses to parasitic infections, often protecting the hosts from infection. We tested for such a role in the crustacean Daphnia and the parasitic bacterium Pasteuria ramosa, a widely used model system for host-parasite interactions. We first determined the infection phenotype (i.e., resistotype) of eight clonal D. magna genotypes against four strains of P. ramosa by attachment test, followed by 16 S rDNA amplicon sequencing to determine if their genotype or their parasite resistotype influences the composition of their microbiome. We then reciprocally transplanted the microbiota of two host genotypes with opposite resistotypes to four P. ramosa isolates, followed by a reassessment of their resistotype after transplantation. We found significant differences in microbiome composition and structure between Daphnia genotypes and between Daphnia resistotypes to specific P. ramosa strains. Reciprocal microbiota exchange or making the Daphnia hosts bacteria-free, however, did not influence the resistotypes of the hosts. Thus, in contrary to what has been observed in some taxa, our results suggest that D. magna susceptibility to P. ramosa is strongly dictated by the genetic differences of the hosts and is still dependent on Daphnia’s first line of immune defense against the esophageal attachment of P. ramosa, which appears to be uninfluenced by the host’s microbiota. | NA | no | No good fitness measure and measure host GD | NA | no | no |
| 5637 | 2020 | 44111 | Sitterle, E; Maufrais, C; Sertour, N; Palayret, M; d’Enfert, C; Bougnoux, ME | 2019 | Within-Host Genomic Diversity of Candida albicans in Healthy Carriers | Genomic variations in Candida albicans, a major fungal pathogen of humans, have been observed upon exposure of this yeast to different stresses and experimental infections, possibly contributing to subsequent adaptation to these stress conditions. Yet, little is known about the extent of genomic diversity that is associated with commensalism, the predominant lifestyle of C. albicans in humans. In this study, we investigated the genetic diversity of C. albicans oral isolates recovered from healthy individuals, using multilocus sequencing typing (MLST) and whole genome sequencing. While MLST revealed occasional differences between isolates collected from a single individual, genome sequencing showed that they differed by numerous single nucleotide polymorphisms, mostly resulting from short-range loss-of-heterozygosity events. These differences were shown to have occurred upon human carriage of C. albicans rather than subsequent in vitro manipulation of the isolates. Thus, C. albicans intra-sample diversity appears common in healthy individuals, higher than that observed using MLST. We propose that diversifying lineages coexist in a single human individual, and this diversity can enable rapid adaptation under stress exposure. These results are crucial for the interpretation of longitudinal studies evaluating the evolution of the C. albicans genome. | NA | no | gd of pathogen | NA | no | no |
| 5638 | 2020 | 44040 | Sitters, H; Di Stefano, J | 2020 | Integrating functional connectivity and fire management for better conservation outcomes | Globally, the mean abundance of terrestrial animals has fallen by 50% since 1970, and populations face ongoing threats associated with habitat loss, fragmentation, climate change, and disturbance. Climate change can influence the quality of remaining habitat directly and indirectly by precipitating increases in the extent, frequency, and severity of natural disturbances, such as fire. Species face the combined threats of habitat clearance, changing climates, and altered disturbance regimes, each of which may interact and have cascading impacts on animal populations. Typically, conservation agencies are limited in their capacity to mitigate rates of habitat clearance, habitat fragmentation, or climate change, yet fire management is increasingly used worldwide to reduce wildfire risk and achieve conservation outcomes. A popular approach to ecological fire management involves the creation of fire mosaics to promote animal diversity. However, this strategy has 2 fundamental limitations: the effect of fire on animal movement within or among habitat patches is not considered and the implications of the current fire regime for long-term population persistence are overlooked. Spatial and temporal patterns in fire history can influence animal movement, which is essential to the survival of individual animals, maintenance of genetic diversity, and persistence of populations, species, and ecosystems. We argue that there is rich potential for fire managers to manipulate animal movement patterns; enhance functional connectivity, gene flow, and genetic diversity; and increase the capacity of populations to persist under shifting environmental conditions. Recent methodological advances, such as spatiotemporal connectivity modeling, spatially explicit individual-based simulation, and fire-regime modeling can be integrated to achieve better outcomes for biodiversity in human-modified, fire-prone landscapes. Article impact statement: Land managers may conserve populations by using fire to sustain or enhance functional connectivity. | NA | no | No good fitness measure and measure host GD | NA | no | no |
| 5639 | 2020 | 44042 | Sivasubbu, S; Scaria, V | 2019 | Genomics of rare genetic diseases-experiences from India | Home to a culturally heterogeneous population, India is also a melting pot of genetic diversity. The population architecture characterized by multiple endogamous groups with specific marriage patterns, including the widely prevalent practice of consanguinity, not only makes the Indian population distinct from rest of the world but also provides a unique advantage and niche to understand genetic diseases. Centuries of genetic isolation of population groups have amplified the founder effects, contributing to high prevalence of recessive alleles, which translates into genetic diseases, including rare genetic diseases in India. Rare genetic diseases are becoming a public health concern in India because a large population size of close to a billion people would essentially translate to a huge disease burden for even the rarest of the rare diseases. Genomics-based approaches have been demonstrated to accelerate the diagnosis of rare genetic diseases and reduce the socio-economic burden. The Genomics for Understanding Rare Diseases: India Alliance Network (GUaRDIAN) stands for providing genomic solutions for rare diseases in India. The consortium aims to establish a unique collaborative framework in health care planning, implementation, and delivery in the specific area of rare genetic diseases. It is a nation-wide collaborative research initiative catering to rare diseases across multiple cohorts, with over 240 clinician/scientist collaborators across 70 major medical/research centers. Within the GUaRDIAN framework, clinicians refer rare disease patients, generate whole genome or exome datasets followed by computational analysis of the data for identifying the causal pathogenic variations. The outcomes of GUaRDIAN are being translated as community services through a suitable platform providing low-cost diagnostic assays in India. In addition to GUaRDIAN, several genomic investigations for diseased and healthy population are being undertaken in the country to solve the rare disease dilemma. In summary, rare diseases contribute to a significant disease burden in India. Genomics-based solutions can enable accelerated diagnosis and management of rare diseases. We discuss how a collaborative research initiative such as GUaRDIAN can provide a nation-wide framework to cater to the rare disease community of India. | NA | no | human | NA | no | no |
| 5640 | 2020 | 44111 | Skrlec, I; Milic, J; Cilensek, I; Petrovic, D; Wagner, J; Peterlin, B | 2019 | Circadian clock genes and myocardial infarction in patients with type 2 diabetes mellitus | Disruption of circadian clock may trigger the onset of diabetes mellitus and myocardial infarction. Type 2 diabetes mellitus (T2DM) is well-known risk factors for cardiovascular diseases and myocardial infarction. We performed a case-control study, where we explored the possible association between single nucleotide polymorphisms in three circadian rhythm genes (ARNTL, CLOCK, and PER2) and myocardial infarction in 657 patients with T2DM. The study group consisted of 231 patients with myocardial infarction and T2DM and a control group of 426 T2DM patients. We hypothesized that variations in the circadian rhythm genes in patients with T2DM could be an additional risk factor for myocardial infarction. The statistically significant difference was found in allelic (p = 1.1 x 10^-5^) and genotype distribution (p = 1.42 x 10^-4^) between two groups of the rs12363415 at the ARNTL gene locus. We provide evidence that genetic variability in the ARNTL gene might be associated with myocardial infarction in patients with T2DM. | NA | no | human | NA | no | no |
| 5641 | 2020 | 44042 | Skuse, B; Phillippi, A; Batchelder, E | 2019 | The Absence of Nosema bombi in Bumblebees (Bombus spp.) on Farms in Michigan | The fungal pathogen Nosema bombi impacts bumblebee fitness and is a factor in population declines throughout North America. Pesticides are also thought to contribute to declines, because they harm the innate immune system of insects, making them more susceptible to disease. A total of 232 bumblebees were collected at nine different farms across Michigan (U.S.A.), including farms that used pesticides and those that did not, in summer 2017. The bees were identified and tested for Nosema bombi. All bees were found to be free front N. bombi infection, suggesting this microsporidian may not yet have a biologically important impact on bumblebee populations in Michigan. | NA | no | no good gd measure host | NA | no | no |
| 5642 | 2020 | 44113 | Slade, JWG; Watson, MJ; MacDougall-Shackleton, EA | 2017 | Birdsong signals individual diversity at the major histocompatibility complex | The major histocompatibility complex (MHC) plays a key role in vertebrate immunity, and pathogen-mediated selection often favours certain allelic combinations. Assessing potential mates’ MHC profiles may provide receivers with genetic benefits (identifying MHC-compatible mates and producing optimally diverse offspring) and/or material benefits (identifying optimally diverse mates capable of high parental investment). Oscine songbirds learn songs during early life, such that song repertoire content can reflect population of origin while song complexity can reflect early life condition. Thus birdsong may advertise the singer’s genetic dissimilarity to others in the population (and, presumably, compatibility with potential mates), or individual genetic diversity (and thus condition-dependent material benefits). We tested whether song repertoire content and/or complexity signal MHC class IIb dissimilarity and/or diversity in male song sparrows (Melospiza melodia). Pairwise dissimilarity in repertoire content did not predict MHC dissimilarity between males, suggesting that locally rare songs do not signal rare MHC profiles. Thus, geographical variation in song may not facilitate MHC-mediated inbreeding or outbreeding. Larger repertoires were associated with intermediate MHC diversity, suggesting intermediate rather than maximal MHC diversity is optimal. This could reflect trade-offs between resisting infection and autoimmune disorders. Song complexity may advertise optimal MHC diversity, a trait affecting disease resistance and capacity for parental care. | NA | no | gd of pathogen | NA | no | no |
| 5643 | 2020 | 44040 | Slynko, EE; Slynko, YV; Rabushko, VI | 2020 | Adaptive strategy of Rapana venosa (Gastropoda, Muricidae) in the invasive population of the Black Sea | We conducted molecular-genetic and morphological studies on the veined rapa whelk Rapana venosa (Valenciennes, 1846) in the Crimean waters of the Black Sea in order to determine possible reasons of the invasive success of this mollusk. Molecular-genetic tests were performed using COI gene; the surveyed samples reliably identified to R. venosa species. We compared the data on initial (from the Far-Eastern seas) and some invasive populations. In the natural conditions of the Yellow, East China Seas, and the Sea of Japan, the genetic diversity of rapa whelk is high (Hd = 0.933, pi = 0.002). In all the invasive populations of rapa whelk (Black Sea, European and North American), an extremely low level of haplotypic and nucleotide diversity was determined (Hd = 0.0, p = 0.0). Despite low values of genetic diversity, the invasive populations of rapa whelk are characterized by ecological success. We noted stable growth of populations, tolerance to diseases and parasites, effective reproduction, high fertility. This contradicts the main provisions of the population genetics theory of formation of edge of range populations, because usually only a small number of specimens of rapa whelk were introduced. The explanations of the unique condition of the invasive populations of rapa whelk, both from an ecological perspective (high resistance to fluctuations of hydrological factors and chemical pollution, absence of enemies, high fertility) and genetic perspective (high selective value of separate haplotypes settled in the new water areas) have not been confirmed. We determined that a very important factor for the naturalization of the rapa whelk at low genetic diversity is the intra-species morpho-ecological divergence. In the Crimean water area of the Black Sea, R. venosa was found to have two morpho-ecological forms associated with the peculiarities of the development of the proportions of the shell. Study of successful invasive species would allow timely and adequate reaction to new cases of introduction. | NA | no | No good fitness measure and measure host GD | NA | no | no |
| 5644 | 2020 | 44042 | Small, ST; Labbe, F; Coulibaly, YI; Nutman, TB; King, CL; Serre, D; Zimmerman, PA | 2019 | Human Migration and the Spread of the Nematode Parasite Wuchereria bancrofti | The human disease lymphatic filariasis causes the debilitating effects of elephantiasis and hydrocele. Lymphatic filariasis currently affects the lives of 90 million people in 52 countries. There are three nematodes that cause lymphatic filariasis, Brugia malayi, Brugia timori, and Wuchereria bancrofti, but 90% of all cases of lymphatic filariasis are caused solely by W. bancrofti (Wb). Here we use population genomics to reconstruct the probable route and timing of migration of Wb strains that currently infect Africa, Haiti, and Papua New Guinea (PNG). We used selective whole genome amplification to sequence 42 whole genomes of single Wb worms from populations in Haiti, Mali, Kenya, and PNG. Our results are consistent with a hypothesis of an Island Southeast Asia or East Asian origin of Wb. Our demographic models support divergence times that correlate with the migration of human populations. We hypothesize that PNG was infected at two separate times, first by the Melanesians and later by the migrating Austronesians. The migrating Austronesians also likely introduced Wb to Madagascar where later migrations spread it to continental Africa. From Africa, Wb spread to the New World during the transatlantic slave trade. Genome scans identified 17 genes that were highly differentiated among Wb populations. Among these are genes associated with human immune suppression, insecticide sensitivity, and proposed drug targets. Identifying the distribution of genetic diversity in Wb populations and selection forces acting on the genome will build a foundation to test future hypotheses and help predict response to current eradication efforts. | NA | no | human | NA | no | no |
| 5645 | 2020 | 44041 | Smith, LM; Gore, JA; de Torrez, ECB; Webb, E; Ridgley, F; Tornwall, B | 2019 | High Incidence of Hypopigmented Marks in the Endangered Florida Bonneted Bat | Aberrant patches of white skin or fur known as hypopigmented marks have been observed in many mammal species worldwide, but they are typically limited to only a few individuals in a population. Hypopigmented marks were documented in only two museum specimens of the federally endangered Florida bonneted bat (Eumops floridanus) as early as 1950, but recent observations suggest that these marks may be more common in this species than previously believed. To better understand the occurrence and persistence of hypopigmented marks in Florida bonneted bats, we evaluated the variation in frequency, compared effects on survival, and assessed spatial and temporal differences of specimens at an intensively studied site and across the species range. From 2014 through 2017, we regularly captured Florida bonneted bats for demographic studies at Babcock-Webb Wildlife Management Area in Charlotte County and we observed hypopigmented marks on 172 (80.8%) of 213 individuals, the highest incidence known for bats. The proportion of hypopigmented marks did not differ with sex, age at first capture, or reproductive status, and importantly, we documented the persistence of hypopigmented marks in individuals over multiple months. We also found no difference in survival or capture probability between hypopigmented and solid-colored individuals. Using data from concurrent mist-net studies, we assessed the proportion of Florida bonneted bats with hypopigmented marks across the species range and found a higher proportion of individuals with hypopigmented marks in the two northern counties (80.8% in Charlotte County and 61.5% in Polk County) compared with the two southern counties (21.1% in Miami-Dade County and 16.7% in Collier County). Additionally, we compared the proportion of hypopigmented individuals in Miami-Dade County between recently captured/collected bats (post-2004) and historically (pre-1965) collected museum specimens and found more hypopigmented marks in recently captured bats. The persistence of hypopigmented marks over time, the presence of marks on bats from different locations, and our finding of no effect of age on the presence of marks all strongly suggest that the hypopigmented marks in Florida bonneted bats are the result of genetic rather than environmental factors. Further study is required to understand the mechanism underlying the high incidence of hypopigmented marks and to determine whether the prevalence of hypopigmented marks indicates low genetic diversity that could threaten Florida bonneted bats. | NA | no | No good fitness measure and measure host GD | NA | no | no |
| 5646 | 2020 | 44111 | Snoek, BL; Volkers, RJM; Nijveen, H; Petersen, C; Dirksen, P; Sterken, MG; Nakad, R; Riksen, JAG; Rosenstiel, P; Stastna, JJ; Braeckman, BP; Harvey, SC; Schulenburg, H; Kammenga, JE | 2019 | A multi-parent recombinant inbred line population of C. elegans allows identification of novel QTLs for complex life history traits | BackgroundThe nematode Caenorhabditis elegans has been extensively used to explore the relationships between complex traits, genotypes, and environments. Complex traits can vary across different genotypes of a species, and the genetic regulators of trait variation can be mapped on the genome using quantitative trait locus (QTL) analysis of recombinant inbred lines (RILs) derived from genetically and phenotypically divergent parents. Most RILs have been derived from crossing two parents from globally distant locations. However, the genetic diversity between local C. elegans populations can be as diverse as between global populations and could thus provide means of identifying genetic variation associated with complex traits relevant on a broader scale.ResultsTo investigate the effect of local genetic variation on heritable traits, we developed a new RIL population derived from 4 parental wild isolates collected from 2 closely located sites in France: Orsay and Santeuil. We crossed these 4 genetically diverse parental isolates to generate a population of 200 multi-parental RILs and used RNA-seq to obtain sequence polymorphisms identifying almost 9000 SNPs variable between the 4 genotypes with an average spacing of 11kb, doubling the mapping resolution relative to currently available RIL panels for many loci. The SNPs were used to construct a genetic map to facilitate QTL analysis. We measured life history traits such as lifespan, stress resistance, developmental speed, and population growth in different environments, and found substantial variation for most traits. We detected multiple QTLs for most traits, including novel QTLs not found in previous QTL analysis, including those for lifespan and pathogen responses. This shows that recombining genetic variation across C. elegans populations that are in geographical close proximity provides ample variation for QTL mapping.ConclusionTaken together, we show that using more parents than the classical two parental genotypes to construct a RIL population facilitates the detection of QTLs and that the use of wild isolates facilitates the detection of QTLs. The use of multi-parent RIL populations can further enhance our understanding of local adaptation and life history trade-offs. | NA | no | No good fitness measure | NA | no | no |
| 5647 | 2020 | 44041 | Soares, L; Latta, SC; Ricklefs, RE | 2020 | Neotropical migratory and resident birds occurring in sympatry during winter have distinct haemosporidian parasite assemblages | Aim We compared the parasite assemblages of over-wintering migratory birds and permanent residents on the wintering grounds. We determined whether parasite sharing between migratory and resident birds is influenced by host phylogenetic relatedness. We then inferred whether migratory birds transport haemosporidian parasite lineages between the breeding and wintering grounds. Location Sierra de Bahoruco National Park, Dominican Republic, Hispaniola. Taxa Migratory and resident birds (primarily Aves: Passeriformes) and their haemosporidian parasites (order Haemosporida, Plasmodium, Haemoproteus and Parahaemoproteus). Methods We used mist nets to capture birds in thorn scrub, broadleaf dry forests and pine forests during midwinter. We used molecular methods to recognize haemosporidian parasites in blood samples, and genotyped infections based on the nucleotide differences in a region of the parasite cytochrome b gene. Results and Main Conclusion We identified 505 infections by 32 haemosporidian parasite lineages in 1,780 blood samples from 37 resident species, and in 901 blood samples from 14 overwintering migratory species, over five years at the same sites. Infection prevalence varied among migratory species from zero to 13%, whereas infection prevalence among resident species ranged up to 77%. Host relatedness did not predict parasite assemblage similarity. We discuss four hypotheses for the rarity of haemosporidian infections in migratory birds during winter, and for the infrequency of parasite sharing between migratory and resident birds: (a) relative abundance and host preferences of dipteran vectors lower parasite transmission to migratory birds; (b) parasite lineages adapted to infect endemic Caribbean hosts are unable to infect migratory species; (c) the physiology of migratory birds after migration and during winter reduces parasite survival; and (d) infected individuals suffer more pronounced mortality rates during migration. We highlight the link between host-parasite coevolution and the physiological adaptations associated with avian seasonal migration. | NA | no | No good fitness measure and measure host GD | NA | no | no |
| 5648 | 2020 | 44113 | Sochor, M; Sarhanova, P; Pfanzelt, S; Travnicek, B | 2017 | Is evolution of apomicts driven by the phylogeography of the sexual ancestor? Insights from European and Caucasian brambles (Rubus, Rosaceae) | AimApomixis, i.e. asexual reproduction via seeds, occurs in many plant taxa of diverse phylogenetic origins and has resulted in a high abundance and wide distribution of some groups. When and where apomicts arose and how their evolution is linked to their sexual ancestors is poorly understood. We aimed at detecting phylogeographical patterns in Rubus ulmifolius-R. sanctus agg., a diploid sexual species aggregate from Rubus series Discolores (Rosaceae), and asked where and when its polyploid apomictic descendants originated. LocationEurope and adjacent regions (Caucasus, Macaronesia, Morocco). MethodsNext-generation sequencing of 10 nuclear microsatellite loci, Sanger sequencing of two plastid loci and ecological niche modelling. ResultsThe data reveal strong, continental-scale genetic structuring within Rubus ulmifolius-R. sanctus agg. Geographical patterns of genetic diversity and ecological niche models indicate its survival mainly on the Iberian Peninsula and in Morocco during the Last Glacial Maximum, as well as population bottlenecks in the eastern Mediterranean and the Caucasus, whereas low allelic diversity in north-western Europe stems from post-glacial re-colonization from southern refugia. The distribution of alleles among diploids and polyploids indicates that the first allopolyploidization events occurred prior to the last glaciation, but also reflects post-glacial gene flow from diploids to polyploids. Main conclusionsPolyploid apomicts both preserve ancestral alleles lost in their diploid ancestors because of ice-age bottlenecks and harbour also derived, i.e. younger, alleles obtained from diploid taxa via recent gene flow. Diversification of apomicts as a result of the diploid’s genetic deterioration is hypothesized. Then, geographical parthenogenesis in Rubus could also be explained by genetic diversity patterns in the diploid, sexual ancestor. | NA | no | No good fitness measure and measure host GD | NA | no | no |
| 5649 | 2020 | 44113 | Soe, MJ; Nejsum, P; Seersholm, FV; Fredensborg, BL; Habraken, R; Haase, K; Hald, MM; Simonsen, R; Hojlund, F; Blanke, L; Merkyte, I; Willerslev, E; Kapel, CMO | 2018 | Ancient DNA from latrines in Northern Europe and the Middle East (500 BC-1700 AD) reveals past parasites and diet | High-resolution insight into parasitic infections and diet of past populations in Northern Europe and the Middle East (500 BC-1700 AD) was obtained by pre-concentration of parasite eggs from ancient latrines and deposits followed by shotgun sequencing of DNA. Complementary profiling of parasite, vertebrate and plant DNA proved highly informative in the study of ancient health, human-animal interactions as well as animal and plant dietary components. Most prominent were finding of soil-borne parasites transmitted directly between humans, but also meat-borne parasites that require consumption of raw or undercooked fish and pork. The detection of parasites for which sheep, horse, dog, pig, and rodents serves as definitive hosts are clear markers of domestic and synanthropic animals living in closer proximity of the respective sites. Finally, the reconstruction of full mitochondrial parasite genomes from whipworm (Ascaris lumbricoides) and roundworm species (Trichuris trichiura and Trichuris muris) and estimates of haplotype frequencies elucidates the genetic diversity and provides insights into epidemiology and parasite biology. | NA | no | No good fitness measure and measure host GD | NA | no | no |
| 5650 | 2020 | 44112 | Song, MF; Guan, YH; Li, HT; Wei, SG; Zhang, LX; Zhang, ZL; Ma, XJ | 2018 | The effects of genetic variation and environmental factors on rhynchophylline and isorhynchophylline in Uncaria macrophylla Wall. from different populations in China | Uncaria macrophylla Wall. is an important Chinese medicinal herb. Rhynchophylline (RIN) and isorhynchophylline (IRN) are its major active compounds. We investigated the influence of genetic differentiation and environmental factors on the RIN and IRN to find the main influencing factors of their contents and lay the foundation for the following cultivation and breeding. We used inter-simple sequence repeat (ISSR) markers to investigate the genetic diversity, and high-performance liquid chromatography (HPLC) to measure the contents of RIN and IRN in 200 samples of U. macrophylla obtained from nine natural populations, and then to analyze the correlation between genetic differentiation, environmental factors of sampling sites and the contents of RIN and IRN. We found that High intra-population (80.05%) and low inter-population (19.95%) genetic diversity existed in the samples of U. macrophylla. To some extent, genetic differentiation and the contents of RIN and IRN had correlation in individual populations (such as JH, MH, XM, and ML). The RIN and IRN contents were significant negatively correlated with the precipitation in May (R-IRN = -0.771, p = 0.015) and June (R-RIN = -0.814, p = 0.008; R-IRN = -0.921, p = 0.000), indicating that precipitation was the main affecting factor of their contents. Interestingly, the analysis results showed that the RIN content had a significant positive correlation (r = 0.585, p = 0.000) with the IRN content (they are isomers); the proportion of RIN had a significant negative correlation with the sum of the two (r = -0.390, p<0.0001), while the proportion of IRN had a significant positive correlation (r = 0.390, p<0.0001). It meant that, with the total quantity of the two compounds increased, the proportion of RIN decreased and the proportion of IRN increased, illustrating that their conversion exist some regularity. Moreover, the content ratio of RIN and IRN was significant positively correlated with the January precipitation (r = 0.716, p = 0.030), implying that January may be the key period for the mutual transformation of RIN and IRN. | NA | no | No good fitness measure and measure host GD | NA | no | no |
| 5651 | 2020 | 44113 | Souza-Shibatta, L; Kotelok-Diniz, T; Ferreira, DG; Shibatta, OA; Sofia, SH; de Assumpcao, L; Pini, SFR; Makrakis, S; Makrakis, MC | 2018 | Genetic Diversity of the Endangered Neotropical Cichlid Fish (Gymnogeophagus setequedas) in Brazil | Gymnogeophagus setequedas is a rare and rheophilic species of tribe Geophagini, considered endangered in Brazilian red lists. Its previously known geographical distribution range was the Parana River basin, in Paraguay, and a tributary of the Itaipu Reservoir in Brazil. Since its description no specimens have been collected in the original known distribution area. However, recent records of G. setequedas in the lower Iguacu River, in a region considered highly endemic for the ichthyofauna, extended the known geographical distribution and may represent one of the last remnants of the species. The aim of this study was to estimate the genetic diversity and population structure of G. setequedas, using microsatellite markers and mitochondrial haplotypes, in order to test the hypothesis of low genetic diversity in this restricted population. Muscular tissue samples of 86 specimens were obtained from nine locations in the Lower Iguacu River basin, between upstream of the Iguacu Falls and downstream of the Salto Caxias Reservoir. Seven microsatellites loci were examined and a total of 120 different alleles were obtained. The number of alleles per locus (N-A) was 17.429, effective alleles (N-E) 6.644, expected heterozygosity (H-E) 0.675, observed (H-O) heterozygosity 0.592, and inbreeding coefficient (F-IS) 0.128. Twelve haplotypes in the D-Loop region were revealed, with values of h (0.7642) and pi (0.00729), suggesting a large and stable population with a long evolutionary history. Thus, both molecular markers revealed high levels of genetic diversity and indicated the occurrence of a single G. setequedas population distributed along a stretch of approximately 200 km. The pattern of mismatch distribution was multimodal, which is usually ascribed to populations in demographic equilibrium. Nevertheless, the construction of a new hydroelectric power plant, already underway between the Salto Caxias Reservoir and Iguacu Falls, could fragment this population, causing loss of genetic diversity and population decline, and for this reason it is necessary to maintain the Iguacu River tributaries and downstream area from the Lower Iguacu Reservoir free of additional dams, to guarantee the survival of this species. | NA | no | No good fitness measure and measure host GD | NA | no | no |
| 5652 | 2020 | 44040 | Souza, AIS; Saraiva, MMS; Casas, MRT; Oliveira, GM; Cardozo, MV; Benevides, VP; Barbosa, FO; Freitas, OC; Almeida, AM; Berchieri, A | 2020 | High occurrence of beta-lactamase-producing Salmonella Heidelberg from poultry origin | Salmonella Heidelberg is commonly reported in foodborne outbreaks around the world, and chickens and poultry products are known as important source of these pathogen. Multidrug-resistant S. Heidelberg strains are disseminated into poultry production chair, which can lead to severe clinical infections in humans and of difficult to treat. This study aimed at evaluating the beta-lactam susceptibility and genotypic relatedness of Salmonella Heidelberg at Brazilian poultry production chain. Sixty-two S. Heidelberg strains from poultry production chain (poultry, poultry meat and poultry farm) were used. All strains were evaluated to antimicrobial susceptibility by diffusion disk test, as well as beta-lactam resistance genes. Genotypic relatedness was assessed by Pulsed-Field Gel Eletrophoresis, using Xba1 restriction enzyme. Forty-one strains were characterized as multidrug-resistant according to phenotype characterization. The resistance susceptibility revealed 31 distinct profiles, with higher prevalence of streptomycin (61/62), nalidixic acid (50/62), tetracycline (43/62) and beta-lactam drugs (37/62). bla(CMY-2) was the more frequent beta-lactamase gene found (38/62); other resistance genes found were bla(CTX-M) (2/62), bla(SHV) (3/62) and bla(TEM-1) (38/62). No carbapenemase genes was found. The Pulsed-Field Gel Electrophoresis showed 58 different profiles. Strains with a larger number of antimicrobial resistance were grouped into ten major clusters apart from others. The spread of resistance by ampC continues to rise, thereby turning concern to public health, since the beta-lactam antimicrobials are used as a therapeutic treatment in humans. | NA | no | No good fitness measure and measure host GD | NA | no | no |
| 5653 | 2020 | 44111 | Spaan, RS; Epps, CW; Ezenwa, VO; Jolles, AE | 2019 | Why did the buffalo cross the park? Resource shortages, but not infections, drive dispersal in female African buffalo (Syncerus caffer) | Dispersal facilitates population health and maintains resilience in species via gene flow. Adult dispersal occurs in some species, is often facultative, and is poorly understood, but has important management implications, particularly with respect to disease spread. Although the role of adult dispersal in spreading disease has been documented, the potential influence of disease on dispersal has received little attention. African buffalo (Syncerus caffer) are wide-ranging and harbor many pathogens that can affect nearby livestock. Dispersal of adult buffalo has been described, but ecological and social drivers of buffalo dispersal are poorly understood. We investigated drivers of adult buffalo dispersal during a 4-year longitudinal study at Kruger National Park, South Africa. We monitored the spatial movement of 304 female buffalo in two focal areas using satellite and radio collars, capturing each buffalo every 6 months to assess animal traits and disease status. We used generalized linear mixed models to determine whether likelihood of dispersal for individual female buffalo was influenced by animal traits, herd identity, environmental variables, gastrointestinal parasites, or microparasite infections. The likelihood and drivers of buffalo dispersal varied by herd, area, and year. In the Lower Sabie herd, where resources were abundant, younger individuals were more likely to disperse, with most dispersal occurring in the early wet season and during an unusually dry year, 2009. In the resource-poor Crocodile Bridge area, buffalo in poor condition were most likely to disperse. Our findings suggest that dispersal of female buffalo is driven by either seasonal (Lower Sabie) or perhaps social (Crocodile Bridge) resource restriction, indicating resource limitation and dispersal decisions are tightly linked for this social ungulate. We found no direct effects of infections on buffalo dispersal, assuaging fears that highly infectious individuals might be more prone to dispersing, which could accelerate the spatial spread of infectious diseases. | NA | no | No good fitness measure and measure host GD | NA | no | no |
| 5654 | 2020 | 44112 | Spitzweg, C; Hofmeyr, MD; Fritz, U; Vamberger, M | 2019 | Leopard tortoises in southern Africa have greater genetic diversity in the north than in the south (Testudinidae) | In contrast to mammals, little is known about the phylogeographic structuring of widely distributed African reptile species. With the present study, we contribute data for the leopard tortoise (Stigmochelys pardalis). It ranges from the Horn of Africa southward to South Africa and westwards to southern Angola. However, its natural occurrence is disputed for some southern regions. To clarify the situation, we used mtDNA sequences and 14 microsatellite loci from 204 individuals mainly from southern Africa. Our results retrieved five mitochondrial clades; one in the south and two in the north-west and north-east of southern Africa, respectively, plus two distributed further north. Using microsatellites, the southern clade matched with a well-defined southern nuclear cluster, whilst the two northern clades from southern Africa corresponded to another nuclear cluster with three subclusters. One subcluster had a western and central distribution, another occurred mostly in the north-east, and the third in a small eastern region (Maputaland), which forms part of a biodiversity hotspot. Genetic diversity was low in the south and high in the north of our study region, particularly in the north-east. Our results refuted that translocations influenced the genetic structure of leopard tortoises substantially. We propose that Pleistocene climatic fluctuations caused leopard tortoises to retract to distinct refugia in southern and northern regions and ascribe the high genetic diversity in the north of southern Africa to genetic structuring caused by the survival in three refuges and subsequent admixture, whereas tortoises in the south seem to have survived in only one continuous coastal refuge. | NA | no | No good fitness measure | NA | no | no |
| 5655 | 2020 | 44111 | Splendiani, A; Giovannotti, M; Righi, T; Fioravanti, T; Cerioni, PN; Lorenzoni, M; Carosi, A; La Porta, G; Barucchi, VC | 2019 | Introgression despite protection: the case of native brown trout in Natura 2000 network in Italy | Mediterranean brown trout is subject to several serious threats such as pollution, water abstraction, habitat alteration and especially genetic introgression with domestic strains used for stocking activities. Despite this latter issue has largely been debated by scientists, local managers and stakeholders for decades, official stocking practices with domestic trout still persists in several countries (Italy included), even if there are laws explicitly prohibiting introduction of organisms of non-local origin. Probably, the last opportunity to conserve native brown trout populations is represented by protected areas. Therefore, in the present study, we aimed to verify the role of the Nature 2000 network and a national park as valid tools to guarantee the survival of native brown trout in the Apennines. Partial mitochondrial DNA control region sequence analysis and genotypingof the locus LDH-C1\* and 11 microsatellites were used to investigate the genetic diversity of three rivers from central Italy. For all rivers investigated a temporal analysis of introgression was also carried out. The genetic diversity of three domestic stocks was included in the sampling design for comparison. The main results of this study indicated that: (i) the genetic diversity of brown trout in central Italy is very complex and (ii) its conservation is seriously threatened by genetic introgression phenomena still ongoing. The only samples showing no introgression or a decrease in genetic introgression were those isolated by the presence of natural and/or artificial barriers to fish movements rather than protected by inhabiting rivers withinthe Natura 2000 network. This observation prompts an important reflection on issues concerning fluvial continuity restoration and suggests that barrier removal should be undertaken with caution in order to avoid the concrete risk of domestic trout spreading that could promote additional loss of native brown trout biodiversity. | NA | no | No good fitness measure | NA | no | no |
| 5656 | 2020 | 44112 | Srivastava, A; Srivastava, KR; Hebbar, M; Galada, C; Kadavigrere, R; Su, FY; Cao, XH; Chinnaiyan, AM; Girisha, KM; Shukla, A; Bielas, SL | 2018 | Genetic diversity of NDUFV1-dependent mitochondrial complex I deficiency | Medical genomics research performed in diverse population facilitates a better understanding of the genetic basis of developmental disorders, with regional implications for community genetics. Autosomal recessive mitochondrial complex I deficiency (MCID) accounts for a constellation of clinical features, including encephalopathies, myopathies, and Leigh Syndrome. Using whole-exome sequencing, we identified biallelic missense variants in NDUFV1 that encodes the 51-kD subunit of complex I (NADH dehydrogenase) NDUFV1. Mapping the variants on published crystal structures of mitochondrial complex I demonstrate that the novel c.1118T > C (p.(Phe373Ser)) variant is predicted to diminish the affinity of the active pocket of NDUFV1 for FMN that correlates to an early onset of debilitating MCID symptoms. The c.1156C > T (p.(Arg386Cys)) variant is predicted to alter electron shuttling required for energy production and correlate to a disease onset in childhood. NDUFV1 c.1156C > T (p.(Arg386Cys)) represents a founder variant in South Asian populations that have value in prioritizing this variant in a population-specific manner for genetic diagnostic evaluation. In conclusion, our results demonstrate the advantage of analyzing population-specific sequences to understand the disease pathophysiology and prevalence of inherited risk variants in the underrepresented populations. | NA | no | No good fitness measure and measure host GD | NA | no | no |
| 5657 | 2020 | 44113 | Srivastava, D; Gayatri, MC; Sarangi, SK | 2018 | In vitro mutagenesis and characterization of mutants through morphological and genetic analysis in orchid Aerides crispa Lindl. | Mutation breeding techniques combined with tissue culture and molecular marker methods provide a powerful tool for improvement of slow growing plants such as orchids. In this study, we developed a protocol for in vitro mutagenic studies in a medicinal orchid of Western Ghats, Aerides crispa Lindl., commonly called the Curled Aerides. In vitro grown 60 day old protocorms treated with low concentrations (0.025-0.03%) of colchicine, ethyl methane sulphonate (EMS), and low doses (1-4Gy) of gamma radiations, and 27 combinations resulted in healthy multiple shoot bud formation with 40-60% survival frequency. Higher singly or combined dose treatments caused protocorm or shoot initials death, after 20-30 days. Healthy 2500 in vitro seedlings were screened for phenotypic changes in shoots and roots owing to a mutagenic effect. In total, 206 in vitro seedlings of 52 variant lines were identified on the basis of their unique leaf shape, colour, white stripes, thickness, length, and width and their root length and thickness. These variant lines were multiplied, established in an orchidarium, and compared with the control for genetic variability by using Random Amplified Polymorphic DNA method. Only 15 genetically distinct mutant lines were identified, which exhibited disparity in growth rate, leaf shape, leaf length, width, chlorophyll variegation, stomatal density, and/or pigment contents. | NA | no | No good fitness measure and measure host GD | NA | no | no |
| 5658 | 2020 | 44113 | Staples, J; Maxwell, EK; Gosalia, N; Gonzaga-Jauregui, C; Snyder, C; Hawes, A; Penn, J; Ulloa, R; Bai, XD; Lopez, AE; Van Hout, CV; O’Dushlaine, C; Teslovich, TM; McCarthy, SE; Balasubramanian, S; Kirchner, HL; Leader, JB; Murray, MF; Ledbetter, DH; Shuldiner, AR; Yancoupolos, GD; Dewey, FE; Carey, DJ; Overton, JD; Baras, A; Habegger, L; Reid, JG | 2018 | Profiling and Leveraging Relatedness in a Precision Medicine Cohort of 92,455 Exomes | Large-scale human genetics studies are ascertaining increasing proportions of populations as they continue growing in both number and scale. As a result, the amount of cryptic relatedness within these study cohorts is growing rapidly and has significant implications on downstream analyses. We demonstrate this growth empirically among the first 92,455 exomes from the DiscovEHR cohort and, via a custom simulation framework we developed called SimProgeny, show that these measures are in line with expectations given the underlying population and ascertainment approach. For example, within DiscovEHR we identified similar to 66,000 close (first-and second-degree) relationships, involving 55.6% of study participants. Our simulation results project that >70% of the cohort will be involved in these close relationships, given that DiscovEHR scales to 250,000 recruited individuals. We reconstructed 12,574 pedigrees by using these relationships (including 2,192 nuclear families) and leveraged them for multiple applications. The pedigrees substantially improved the phasing accuracy of 20,947 rare, deleterious compound heterozygous mutations. Reconstructed nuclear families were critical for identifying 3,415 de novo mutations in similar to 1,783 genes. Finally, we demonstrate the segregation of known and suspected disease-causing mutations, including a tandem duplication that occurs in LDLR and causes familial hypercholesterolemia, through reconstructed pedigrees. In summary, this work highlights the prevalence of cryptic relatedness expected among large healthcare population-genomic studies and demonstrates several analyses that are uniquely enabled by large amounts of cryptic relatedness. | NA | no | No good fitness measure and measure host GD | NA | no | no |
| 5659 | 2020 | 44111 | Stenzel, T; Dziewulska, D; Smialek, M; Tykalowski, B; Kowalczyk, J; Koncicki, A | 2019 | Comparison of the immune response to vaccination with pigeon circovirus recombinant capsid protein (PiCV rCP) in pigeons uninfected and subclinically infected with PiCV | Infections with immunosuppressive pigeon circovirus (PiCV) pose the most severe health problem to the global pigeon breeding. The vaccination with immunogenic PiCV recombinant capsid protein (PiCV rCP) is a potential tool for disease control. Because of the high prevalence of PiCV asymptomatic infections, the subclinically infected pigeons will be vaccinated in practice. The aim of this study was to answer a question if vaccination of asymptomatic, infected with PiCV pigeons induces a similar immune response to PiCV rCP as in uninfected birds. One hundred and twenty 6-week-old carrier pigeons were divided into 4 groups (2 groups of naturally infected and uninfected with PiCV individuals). Birds from groups V and V1 were vaccinated twice with PiCV rCP mixed with an adjuvant, whereas pigeons from groups C and C1 were immunized with an adjuvant only. The expression of genes encoding IFN-gamma., CD4, and CD8 T lymphocyte receptors; the number of anti-PiCV rCP IgY-secreting B cells (SBC) and anti-PiCV rCP IgY were evaluated 2, 21, 39 and 46 days post vaccination (dpv). Study results showed that the expression of CD8 and IFN-gamma. genes was higher in both groups of infected pigeons than in the uninfected birds, irrespective of vaccination. In the uninfected birds, the expression of these genes was insignificantly higher in the vaccinated pigeons. The anti-PiCV rCP IgY-SBC were detected on 2 and 23 dpv and seroconversion was noted on 23 and 39 dpv in V and V1 groups, respectively. In the light of the results obtained, it could be concluded that pigeon circovirus recombinant capsid protein elicits the immune response in both naturally infected and uninfected pigeons, but its rate varies depending on PiCV infectious status. The infection with PiCV masks the potential cellular immune response to the vaccination with PiCV rCP and leads to the suppression of humoral immunity. | NA | no | No good fitness measure and measure host GD | NA | no | no |
| 5660 | 2020 | 44043 | Stephens, PR; Altizer, S; Ezenwa, VO; Gittleman, JL; Moan, E; Han, B; Huang, S; Pappalardo, P | 2019 | Parasite sharing in wild ungulates and their predators: Effects of phylogeny, range overlap, and trophic links | 1. Understanding factors that facilitate interspecific pathogen transmission is a central issue for conservation, agriculture, and human health. Past work showed that host phylogenetic relatedness and geographical proximity can increase cross-species transmission, but further work is needed to examine the importance of host traits, and species interactions such as predation, in determining the degree to which parasites are shared between hosts. 2. Here we consider the factors that predict patterns of parasite sharing across a diverse assemblage of 116 wild ungulates (i.e., hoofed mammals in the Artiodactyla and Perissodactyla) and nearly 900 species of micro- and macroparasites, controlling for differences in total parasite richness and host sampling effort. We also consider the effects of trophic links on parasite sharing between ungulates and carnivores. 3. We tested for the relative influence of range overlap, phylogenetic distance, body mass, and ecological dissimilarity (i.e., the distance separating species in a Euclidean distance matrix based on standardized traits) on parasite sharing. We also tested for the effects of variation in study effort as a potential source of bias in our data, and tested whether carnivores reported to feed on ungulates have more ungulate parasites than those that use other resources. 4. As in other groups, geographical range overlap and phylogenetic similarity predicted greater parasite community similarity in ungulates. Ecological dissimilarity showed a weak negative relationship with parasite sharing. Counter to our expectations, differences, not similarity, in host body mass predicted greater parasite sharing between pairs of ungulate hosts. Pairs of well-studied host species showed higher overlap than poorly studied species, although including sampling effort did not reduce the importance of biological traits in our models. Finally, carnivores that feed on ungulates harboured a greater richness of ungulate helminths. 5. Overall, we show that the factors that predict parasite sharing in wild ungulates are similar to those known for other mammal groups, and demonstrate the importance of controlling for heterogeneity in host sampling effort in future analyses of parasite sharing. We also show that ecological interactions, in this case trophic links via predation, can allow sharing of some parasite species among distantly related host species. | NA | no | No good fitness measure and measure host GD | NA | no | no |
| 5661 | 2020 | 44113 | Storfer, A; Epstein, B; Jones, M; Micheletti, S; Spear, SF; Lachish, S; Fox, S | 2017 | Landscape genetics of the Tasmanian devil: implications for spread of an infectious cancer | Emerging infectious diseases are increasingly recognized in species’ declines and extinctions. Landscape genetics can be used as a tool to predict disease emergence and spread. The Tasmanian devil is threatened with extinction by a nearly 100% fatal transmissible cancer, which has spread across 95% of the species’ geographic range in 20 years. Here, we present a landscape genetic analysis in the last remaining uninfected parts of the Tasmanian devil’s geographic range to: describe population genetic structure, characterize genetic diversity, and test the influence of landscape variables on Tasmanian devil gene flow to assess the potential for disease spread. In contrast to previous genetic studies on Tasmanian devils showing evidence for two genetic populations island-wide, our genetic based assignment tests and spatial principal components analyses suggest at least two, and possibly three, populations in a study area that is approximately 15% of the size of the overall species’ geographic range. Positive spatial autocorrelation declined at about 40 km, in contrast to 80 km in eastern populations, highlighting the need for range-wide genetic studies. Strong genetic structure was found between devils in the northern part of the study area and those found south of Macquarie Harbor, with weaker structure found between the northeastern and northwestern portion of our study area. Consistent with previous work, we found low overall genetic diversity, likely owing to a combination of founder effects and extreme weather events thousands of years ago that likely caused large-scale population declines. We also found possible signs of recent bottlenecks, perhaps resulting from forest clearing for dairy farming in the central part of the study area. This human disturbance also may have contributed to weak genetic structuring detected between the northeastern and northwestern part of the study area. Individual-based least cost path modeling showed limited influence of landscape variables on gene flow, with weak effects of variation in elevation in the northeast. In the northwest, however, landscape genetic models did not perform better than the null isolation-by-distance model. At the larger spatial scale of the northern part of the study area, elevation and temperatures were negatively correlated with gene flow, consistent with low dispersal suitability of higher elevation habitats that have lower temperatures and dense, wet vegetation. Overall, Tasmanian devils are a highly vagile species for which dispersal and gene flow appear to be influenced little by landscape features, and spread of devil facial tumor disease to the remaining portion of the devil’s geographic range seems imminent. Nonetheless, strong genetic structure found between the northern and southern portions of our study area, combined with low densities and limited possible colonization of DFTD from the east suggest there is some time for implementation of management strategies. | NA | no | No good fitness measure and measure host GD | NA | no | no |
| 5662 | 2020 | 44039 | Stowell, SLM; Gagne, RB; McWhirter, D; Edwards, W; Ernest, HB | 2020 | Bighorn Sheep Genetic Structure in Wyoming Reflects Geography and Management | Aligning wildlife management boundaries with accurate biological units promotes effective conservation and management practices that reflect ecological and evolutionary processes. Neutral genetic markers allow for quantitative delineation of population structure without a priori assumptions or biases. In the United States, bighorn sheep (Ovis canadensis) are a charismatic component of Wyoming’s biodiversity and a species that provides important viewing and hunting opportunities. Bighorn sheep abundances are relatively stable throughout Wyoming, and the species is managed by administrative units identified using expert knowledge, distribution and movement data, and geographic and administrative boundaries. We used a panel of 38 variable microsatellite loci and 512 base pairs of mitochondrial DNA sequence to identify the genetic structure throughout the state and in translocation source herds, quantify the extent of genetic diversity within each genetic cluster, and estimate the degree of gene flow among herds using blood and tissue samples collected 1989-2017. We identified genetic structure of Rocky Mountain bighorn sheep in the major mountain ranges of Wyoming, with strong support for >= 5 genetic clusters using microsatellite loci. These genetic clusters generally aligned with current management units, whereas mitochondrial data showed a more complex mosaic that was not geographically patterned. Genetic variation estimated from both markers was high within each herd and comparable among herds. The assignment of individuals reflected a combination of geographic isolation and translocation, which has been extensive. Our results provide a state-wide assessment of genetic diversity and structure that will enhance management by understanding the outcomes of translocation, identifying the source of unknown individuals, and parameterizing disease ecology models. (c) 2020 The Wildlife Society. | NA | no | No good fitness measure | NA | no | no |
| 5663 | 2020 | 44112 | Stronen, AV; Iacolina, L; Pertoldi, C; Tokarska, M; Sorensen, BS; Bahrndorff, S; Olenski, K; Kaminski, S; Nikolskiy, P | 2019 | Genomic variability in the extinct steppe bison (Bison priscus) compared to the European bison (Bison bonasus) | In 2009, a frozen mummy of the steppe bison (SB) (Bison priscus) was discovered between the lower Kolyma River and the Alazeya River in northeast Siberia, Russia. The specimen was dated with C-14 and estimated to have lived more than 48,000 C-14 years before present (BP). The relationship between SB and the European Bison (EB) (Bison bonasus), also known as wisent or European wood bison, is unresolved and it is unclear whether the SB and EB overlapped in space and time. The aim of our study was to compare genetic variability between the SB specimen and modern EB. We expected higher SB variability due to substantial bottlenecks in the EB approximately one century ago when it became extinct in the wild. The EB (n=167) and the SB specimen were genotyped with the Illumina BovineHD BeadChip with 777,962 single-nucleotide polymorphism (SNP) markers. Steppe bison DNA was extracted and genotyped six times to account for genotyping errors due to low-quality DNA. We obtained a final set of 7786 SNPs. The mean number of private alleles in EB was 0.027 (+/- 0.0002) and in SB, it was 0.288 (+/- 0.0006). This could be explained by factors including differences between species, spatiotemporal divergence, and bottleneck effects. Investigation of historic EB samples could help resolve phylogenetic relationships, the role of the recent bottleneck, and provide information for conservation management to reduce the incidence of disease in the population and maintain its evolutionary potential. | NA | no | No good fitness measure and measure host GD | NA | no | no |
| 5664 | 2020 | 44040 | Stuart, P; Yalcindag, E; Ali, IKM; Peckova, R; Nurcahyo, W; Morrogh-Bernard, H; Foitova, I | 2020 | Entamoeba histolytica infections in wild and semi-wild orangutans in Sumatra and Kalimantan | Key to the success of orangutan conservation management practices is the prevention of the introduction of infectious diseases to the remaining populations. Previous reports of Entamoeba spp. positive orangutans are of concern as Entamoeba spp. infection has been linked to morbidity and mortality in primates. It remains to be determined if the Entamoeba species infecting orangutans is the pathogenic Entamoeba histolytica. Orangutan fecal samples have been collected from orangutans from sites in Sumatra (Bukit Lawang, Ketambe, and Suaq, 241 samples from 64 individuals), and two sites in Kalimantan (Sebangau and Tuanan, 129 samples from 39 individuals). All samples were from wild orangutans except for a proportion from Sumatra which were from semi-wild (108 samples, 10 individuals). E. histolytica-specific nested PCR assays were carried out on the fecal samples. A total of 36 samples from 17 individuals tested positive for E. histolytica. When compared with published sequences using NCBI BLAST the E. histolytica positive samples showed a 98-99% concordance. The majority (76%, n = 36) of the positive isolates came from semi-wild orangutans in Bukit Lawang. This study supports the growing body of evidence that contact with humans is an important risk factor for infection of wild primates with E. histolytica. | NA | no | No good measureGD of host | NA | no | no |
| 5665 | 2020 | 44112 | Sumabat, LG; Kemerait, RC; Kim, DK; Mehta, YR; Brewer, MT | 2018 | Clonality and geographic structure of host-specialized populations of Corynespora cassiicola causing emerging target spot epidemics in the southeastern United States | Corynespora cassiicola is a destructive plant-pathogenic fungus causing widespread target spot epidemics, including outbreaks on cotton, soybean, and tomato in the southeastern United States. Previous studies revealed that populations from the three hosts are genetically distinct and host specialized. Although variation in aggressiveness to cotton and tomato were observed, no genetic diversity was detected within populations sampled from each of these hosts. We aimed to gain a better understanding of the emerging target spot epidemics by developing microsatellite markers for C. cassiicola to assess genetic variation, population structure, and to infer modes of reproduction and mechanisms of dispersal. Two hundred sixty-five isolates from cotton, soybean, tomato, and other host plants were genotyped with 13 microsatellite markers. Genotypic diversity revealed genetic variation within each of the populations collected from different hosts, with the population from cotton dominated by clonal genotypes and showing the least genetic diversity. In addition, C. cassiicola populations on different host species were genetically distinct and structured based on host species. No association between genetic and geographic distances was identified in the tomato populations, and the association in cotton populations was low. However, significant regional geographic structure was detected in the soybean populations of C. cassiicola. These results further support previous findings of introduced host specialized isolates or the evolution of more aggressive strains on each host. The lack of geographic structure suggests that the clones on cotton and tomato spread rapidly, or similar founder populations were established by human-mediated dispersal, and that dispersal is not limited. However, regional geographic structure of populations on soybean suggests limited dispersal among more established populations of C. cassiicola, or genetic differences in founder populations that colonized different geographic areas. | NA | no | gd of pathogen | NA | no | no |
| 5666 | 2020 | 44042 | Sumasgutner, P; Terraube, J; Coulon, A; Villers, A; Chakarov, N; Kruckenhauser, L; Korpimaki, E | 2019 | Landscape homogenization due to agricultural intensification disrupts the relationship between reproductive success and main prey abundance in an avian predator | Background Selecting high-quality habitat and the optimal time to reproduce can increase individual fitness and is a strong evolutionary factor shaping animal populations. However, few studies have investigated the interplay between land cover heterogeneity, limitation in food resources, individual quality and spatial variation in fitness parameters. Here, we explore how individuals of different quality respond to possible mismatches between a cue for prey availability (land cover heterogeneity) and the actual fluctuating prey abundance. Results We analyse timing of breeding and reproductive success in a migratory population of Eurasian kestrels (Falco tinnunculus) breeding in nest-boxes, over a full three-year abundance cycle of main prey (voles), and consider several components of individual quality, including body condition, blood parasite infection, and genetic diversity (n = 448 adults) that act on different time scales. Older individuals, and kestrel parents in higher body condition started egg-laying earlier than younger birds and those in lower body condition. Additionally, egg-laying was initiated earlier during the increase and decrease phases (2011 and 2012) than during the low phase of the vole cycle (2013). Nestling survival (ratio of eggs that fledged successfully) was higher in early nests and in heterogeneous landscapes (i.e., mosaic of different habitat types), which was evident during the increase and decrease phases of the vole cycle, but not during the low vole year. Conclusions We found a strong positive effect of landscape heterogeneity on nestling survival, but only when voles were relatively abundant, whereas a difference in the timing of breeding related to territory landscape heterogeneity was not evident. Therefore, landscape heterogeneity appeared as the main driver of high reproductive performance under favourable food conditions. Our results show that landscape homogenization linked to agricultural intensification disrupts the expected positive effect of vole abundance on reproductive success of kestrels. | NA | no | No good fitness measure and measure host GD | NA | no | no |
| 5667 | 2020 | 44040 | Sun, YQ; Zhao, W; Xu, CQ; Xu, YL; El-Kassaby, YA; De La Torre, AR; Mao, JF | 2020 | Genetic Variation Related to High Elevation Adaptation Revealed by Common Garden Experiments in Pinus yunnanensis | Local adaptation, adaptation to specialized niches and environmental clines have been extensively reported for forest trees. Investigation of the adaptive genetic variation is crucial for forest resource management and breeding, especially in the context of global climate change. Here, we utilized a Pinus yunnanensis common garden experiments established at high and low elevation sites to assess the differences in growth and survival among populations and between the two common garden sites. The studied traits showed significant variation between the two test sites and among populations, suggesting adaptive divergence. To detect genetic variation related to environment, we captured 103,608 high quality SNPs based on RNA sequencing, and used them to assess the genetic diversity and population structure. We identified 321 outlier SNPs from 131 genes showing significant divergence in allelic frequency between survival populations of two sites. Functional categories associated with adaptation to high elevation were found to be related to flavonoid biosynthesis, response to UV, DNA repair, response to reactive oxygen species, and membrane lipid metabolic process. Further investigation of the outlier genes showed overrepresentation of the flavonoid biosynthesis pathway, suggesting that this pathway may play a key role in P. yunnanensis adaptation to high elevation environments. The outlier genes identified, and their variants, provide a basic reference for advanced investigations. | NA | no | No good measureGD of host | NA | no | no |
| 5668 | 2020 | 44112 | Sundaresh, A; Oliveira, J; Chinnadurai, RK; Rajkumar, RP; Hani, L; Krishnamoorthy, R; Leboyer, M; Negi, VS; Tamouza, R | 2019 | IL6/IL6R genetic diversity and plasma IL6 levels in bipolar disorder: An Indo-French study | Reports of association of genetic variants of IL6 and its receptor (IL6R) with psychiatric disorders are inconsistent, and there are few population-based studies thus far in bipolar disorder (BD). We genotyped the IL6 rs1800795 and IL6R rs2228145 polymorphisms in two independent sets of patients exposed to different environmental stimuli such as climatic conditions or specific infectious burden - a French sample and a south Indian Tamil sample of BD with quantitation of circulating plasma IL-6 levels in the latter sub-sample. In both populations, allele and genotype frequencies did not differ significantly between cases and controls for either polymorphism. Upon stratifying based on age at onset, we found no associations with the IL6 rs1800795 variant. However, the IL6R rs2228145 C allele and CC genotype were associated with early onset of disease in the French sample when compared to late onset BD. A similar trend was observed in the Indian population where we also found that plasma IL-6 levels were significantly higher in BD and also in patients who were in residual phase or remission both as compared to controls. Our findings are in favour of a possible trans-ethnic implication of the IL6R genetic diversity in BD and reinforce the notion that IL-6 is an important marker of the operating inflammatory processes in the disease. | NA | no | No good fitness measure and measure host GD | NA | no | no |
| 5669 | 2020 | 44112 | Sunny, A; Monroy-Vilchis, O; Zarco-Gonzalez, MM | 2018 | Genetic diversity and structure of Crotalus triseriatus, a rattlesnake of central Mexico | The isolated and fragmented populations are highly susceptible to stochastic events, increasing the extinction risk because of the decline in putative adaptive potential and individual fitness. The population has high heterozygosity values and a moderate allelic diversity, the heterozygosity values are higher than in most other Crotalus species and snake studies. Possibly these high levels of genetic diversity can be related to a large founder size, high effective population size, multiple paternity and overlapping generations. We did not find the genetic structuring but the effective number of alleles (Ne) was 138.1. We found evidence of bottlenecks and the majority of rattlesnakes were unrelated, despite the small sample size, endemic status, the isolated and fragmented habitat. The genetic information provided in this study can be useful as a first approach to try to make informed conservation efforts for this species and also, important to preserve the habitat of this species; the endangered Abies-Pinus forest of the Nevado the Toluca Volcano. | NA | no | No good fitness measure | NA | no | no |
| 5670 | 2020 | 44112 | Suraj, PG; Nagabhushana, K; Kamalakannan, R; Varghese, M | 2019 | Impact of fertility variation on genetic diversity and phenotypic traits in second generation seed production areas and clonal seed orchards of Eucalyptus camaldulensis | Fertility and gene diversity were estimated in three second generation (F-2) seed stands (SPA 1-3) and two clone trials (CSO 1&2) of Eucalyptus camaldulensis to assess the impact oNseed crop. F-2 seedlots were evaluated in comparison to native provenances, ten commercial clones and interspecific hybrids at diverse sites. SPA 1&2 were genetic gain trials of five first generation (F-1) orchard seedlots, SPA 3 a plantation of one F-1 orchard seedlot, and CSOs were clone trials of 21 commercial clones established at two contrasting sites. Fertility variation, as indicated by sibling coefficient, was high (psi, 9-14) in the SPAs as only about 26 % trees were fertile compared to 81 % trees in CSOs. Effective populatioNsize was higher iNsPA 1 and 2 (N-s, 95 and 74, respectively) thaNsPA 3 (N-s = 39). Fertility was highly skewed in CSO 2 resulting in low effective populatio size (N-s = 2) compared to CSO 1 (N-s = 11). Constant seed collection enabled 3-fold increase in relative populatioNsize and 22 % higher predicted gene diversity in CSO 2. Genetic diversity (He) estimated using SSR markers was higher iNsPA 1&2 and native provenances (NAT), compared to SPA 3 and CSO 1, whereas CSO 2 and clones had lower values. There was a high positive correlation between estimated H-e and predicted gene diversity values of SPAs and CSOs. H-e was positively correlated to mean field survival and negatively correlated to kraft pulp yield (KPY), evaluated at three years in progeny trials across three locations. Number of alleles per locus was higher iNsPAs and native provenances compared to CSOs and clones. Discriminant principal component analysis clustered CSO, NAT and SPA seedlots in different groups while commercial E. camaldulensis clones clustered close to NAT. Multilocus outcrossing rate was generally high (t(m), 91-100 %), though selfing was observed in two families of SPA 3 and CSO 2. Selected interspecific hybrid families of commercial E. camaldulensis clones (with E. urophylla and E. pellita) evaluated at two of the sites had higher H-e and KPY than clones at three years. | NA | no | dometicated host | NA | no | no |
| 5671 | 2020 | 44112 | Susi, H; Filloux, D; Frilander, MJ; Roumagnac, P; Laine, AL | 2019 | Diverse and variable virus communities in wild plant populations revealed by metagenomic tools | Wild plant populations may harbour a myriad of unknown viruses. As the majority of research efforts have targeted economically important plant species, the diversity and prevalence of viruses in the wild has remained largely unknown. However, the recent shift towards metagenomics-based sequencing methodologies, especially those targeting small RNAs, is finally enabling virus discovery from wild hosts. Understanding this diversity of potentially pathogenic microbes in the wild can offer insights into the components of natural biodiversity that promotes long-term coexistence between hosts and parasites in nature, and help predict when and where risks of disease emergence are highest. Here, we used small RNA deep sequencing to identify viruses in Plantago lanceolata populations, and to understand the variation in their prevalence and distribution across the Aland Islands, South-West Finland. By subsequent design of PCR primers, we screened the five most common viruses from two sets of P. lanceolata plants: 164 plants collected from 12 populations irrespective of symptoms, and 90 plants collected from five populations showing conspicuous viral symptoms. In addition to the previously reported species Plantago lanceolata latent virus (PlLV), we found four potentially novel virus species belonging to Caulimovirus, Betapartitivirus, Enamovirus, and Closterovirus genera. Our results show that virus prevalence and diversity varied among the sampled host populations. In six of the virus infected populations only a single virus species was detected, while five of the populations supported between two to five of the studied virus species. In 20% of the infected plants, viruses occurred as coinfections. When the relationship between conspicuous viral symptoms and virus infection was investigated, we found that plants showing symptoms were usually infected (84%), but virus infections were also detected from asymptomatic plants (44%). Jointly, these results reveal a diverse virus community with newly developed tools and protocols that offer exciting opportunities for future studies on the eco-evolutionary dynamics of viruses infecting plants in the wild. | NA | no | no good gd measure host pop | NA | no | no |
| 5672 | 2020 | 44040 | Suurvali, J; Whiteley, AR; Zheng, YC; Gharbi, K; Leptin, M; Wiehe, T | 2020 | The Laboratory Domestication of Zebrafish: From Diverse Populations to Inbred Substrains | We know from human genetic studies that practically all aspects of biology are strongly influenced by the genetic background, as reflected in the advent of personalizedmedicine. Yet, with few exceptions, this is not taken into account when using laboratory populations as animal model systems for research in these fields. Laboratory strains of zebrafish (Danio rerio) are widely used for research in vertebrate developmental biology, behavior, and physiology, for modeling diseases, and for testing pharmaceutic compounds in vivo. However, all of these strains are derived from artificial bottleneck events and therefore are likely to represent only a fraction of the genetic diversity present within the species. Here, we use restriction site-associated DNA sequencing to genetically characterize wild populations of zebrafish from India, Nepal, and Bangladesh, and to compare themto previously published data on four common laboratory strains. We measured nucleotide diversity, heterozygosity, and allele frequency spectra, and find that wild zebrafish are much more diverse than laboratory strains. Further, in wild zebrafish, there is a clear signal of GC-biased gene conversion that is missing in laboratory strains. We also find that zebrafish populations in Nepal and Bangladesh are most distinct from all other strains studied, making theman attractive subject for future studies of zebrafish population genetics andmolecular ecology. Finally, isolates of the same strains kept in different laboratories show a pattern of ongoing differentiation into genetically distinct substrains. Together, our findings broaden the basis for future genetic, physiological, pharmaceutic, and evolutionary studies in Danio rerio. | NA | no | inbreeding | NA | no | no |
| 5673 | 2020 | 44039 | Swart, Y; van Eeden, G; Spark, A; Uren, C; Moller, M | 2020 | Prospective avenues for human population genomics and disease mapping in southern Africa | Population substructure within human populations is globally evident and a well-known confounding factor in many genetic studies. In contrast, admixture mapping exploits population stratification to detect genotype-phenotype correlations in admixed populations. Southern Africa has untapped potential for disease mapping of ancestry-specific disease risk alleles due to the distinct genetic diversity in its populations compared to other populations worldwide. This diversity contributes to a number of phenotypes, including ancestry-specific disease risk and response to pathogens. Although the 1000 Genomes Project significantly improved our understanding of genetic variation globally, southern African populations are still severely underrepresented in biomedical and human genetic studies due to insufficient large-scale publicly available data. In addition to a lack of genetic data in public repositories, existing software, algorithms and resources used for imputation and phasing of genotypic data (amongst others) are largely ineffective for populations with a complex genetic architecture such as that seen in southern Africa. This review article, therefore, aims to summarise the current limitations of conducting genetic studies on populations with a complex genetic architecture to identify potential areas for further research and development. | NA | no | No good fitness measure and measure host GD | NA | no | no |
| 5674 | 2020 | 44112 | Swiderska, Z; Smidova, A; Buchtova, L; Bryjova, A; Fabianova, A; Munclinger, P; Vinkler, M | 2018 | Avian Toll-like receptor allelic diversity far exceeds human polymorphism: an insight from domestic chicken breeds | Immune genes show remarkable levels of adaptive variation shaped by pathogen-mediated selection. Compared to humans, however, population polymorphism in animals has been understudied. To provide an insight into immunogenetic diversity in birds, we sequenced complete protein-coding regions of all Toll-like receptor (TLR) genes with direct orthology between mammals and birds (TLR3, TLR4, TLR5 and TLR7) in 110 domestic chickens from 25 breeds and compared their variability with a corresponding human dataset. Chicken TLRs (chTLRs) exhibit on average nine-times higher nucleotide diversity than human TLRs (hTLRs). Increased potentially functional non-synonymous variability is found in chTLR ligand-binding ectodomains. While we identified seven sites in chTLRs under positive selection and found evidence for convergence between alleles, no selection or convergence was detected in hTLRs. Up to six-times more alleles were identified in fowl (70 chTLR4 alleles vs. 11 hTLR4 alleles). In chTLRs, high numbers of alleles are shared between the breeds and the allelic frequencies are more equal than in hTLRs. These differences may have an important impact on infectious disease resistance and host-parasite co-evolution. Though adaptation through high genetic variation is typical for acquired immunity (e.g. MHC), our results show striking levels of intraspecific polymorphism also in poultry innate immune receptors. | NA | no | dometicated host | NA | no | no |
| 5675 | 2020 | 44112 | Symonds, JE; Clarke, SM; King, N; Walker, SP; Blanchard, B; Sutherland, D; Roberts, R; Preece, MA; Tate, M; Buxton, P; Dodds, KG | 2019 | Developing Successful Breeding Programs for New Zealand Aquaculture: A Perspective on Progress and Future Genomic Opportunities | Over the past 40 years New Zealand (NZ) aquaculture has grown into a significant primary industry. Tonnage is small on a global scale, but the industry has built an international reputation for the supply of high quality seafood to many overseas markets. Since the early 1990s the industry has recognized the potential gains from selective breeding and the challenge has been to develop programs that can overcome biological obstacles (such as larval rearing and mortality) and operate cost-effectively on a relatively small scale while still providing significant gains in multiple traits of economic value. This paper provides an overview of the current status, and a perspective on genomic technology implementation, for the family based genetic improvement programs established for the two main species farmed in NZ: Chinook (king) salmon (Oncorhynchus tshawytscha) and Greenshell (TM) mussel (Perna canakulus). These programs have provided significant benefit to the industry in which we are now developing genomic resources based on genotyping-by-sequencing to complement the breeding programs, enable evaluation of the genetic diversity and identify the potential benefits of genomic selection. This represents an opportunity to increase genetic gain and more effectively utilize the potential for within family selection. | NA | no | No good fitness measure and measure host GD | NA | no | no |
| 5676 | 2020 | 44042 | Szpiech, ZA; Mak, ACY; White, MJ; Hu, DL; Eng, C; Burchard, EG; Hernandez, RD | 2019 | Ancestry-Dependent Enrichment of Deleterious Homozygotes in Runs of Homozygosity | Runs of homozygosity (ROH) are important genomic features that manifest when an individual inherits two haplotypes that are identical by descent. Their length distributions are informative about population history, and their genomic locations are useful for mapping recessive loci contributing to both Mendelian and complex disease risk. We have previously shown that ROH, and especially long ROH that are likely the result of recent parental relatedness, are enriched for homozygous deleterious coding variation in a worldwide sample of outbred individuals. However, the distribution of ROH in admixed populations and their relationship to deleterious homozygous genotypes is understudied. Here we analyze whole-genome sequencing data from 1,441 unrelated individuals from self-identified African American, Puerto Rican, and Mexican American populations. These populations are three-way admixed between European, African, and Native American ancestries and provide an opportunity to study the distribution of deleterious alleles partitioned by local ancestry and ROH. We re-capitulate previous findings that long ROH are enriched for deleterious variation genome-wide. We then partition by local ancestry and show that deleterious homozygotes arise at a higher rate when ROH overlap African ancestry segments than when they overlap European or Native American ancestry segments of the genome. These results suggest that, while ROH on any haplotype background are associated with an inflation of deleterious homozygous variation, African haplotype backgrounds may play a particularly important role in the genetic architecture of complex diseases for admixed individuals, highlighting the need for further study of these populations. | NA | no | human | NA | no | no |
| 5677 | 2020 | 44111 | Takawale, PS; Jade, SS; Bahulikar, RA; Desale, JS | 2019 | Diversity in Lucerne (Medicago sativa L.) germplasm for morphology, yield and molecular markers and their correlations | Lucerne is an important legume forage grown worldwide due to its high nutritive value, yield potential, quality and survival under highly contrasting environments. Estimation of genetic diversity in germplasm is an important criterion in breeding programme and is done based on phenotypic characters, biochemical and molecular markers. Thirty one accessions of Lucerne collected from Maharashtra and Gujarat were evaluated for morphological, yield characters, molecular markers and were compared with national check RL-88. The diversity analysis of morphological and yield characters indicated wide range of variation within the accessions. The accession RLG 08-1 recorded significant differences for plant height, number of tillers per plant, internodal length, green fodder, dry matter and crude protein yield (P<0.05) over RL-88. Green fodder yield was significantly correlated with plant height (0.72**), no. of tillers per plant (0.71**) and internodal length (0.71**). Dry matter yield was found significantly correlated with plant height, no. of tillers per plant, internodal length and crude protein yield (0.70**, 0.69**, 0.70** and 0.99\*\*). Leaf stem ratio was not linearly correlated with green fodder, dry matter and crude protein yield. Principal component analysis (PCA) demonstrated that the first two PCs contributed to 93.7% of total variance among the accessions. The accession RLG 08-1 was found superior among all the accessions. Thirteen Inter Simple Sequence Repeats (ISSRs) markers showed 82.8% polymorphism. The dendrogram revealed slight geographical structuring and RLG 08-1 was found genetically distinct from other accessions.Mantel correlation was not observed between morphological and yield characters as well as molecular markers. | NA | no | dometicated host | NA | no | no |
| 5678 | 2020 | 44113 | Takeshima, S; Corbi-Botto, C; Giovambattista, G; Aida, Y | 2018 | Genetic diversity of BoLA-DRB3 in South American Zebu cattle populations | Background: Bovine leukocyte antigens (BoLAs) are used extensively as markers of disease and immunological traits in cattle. However, until now, characterization of BoLA gene polymorphisms in Zebu breeds using high resolution typing methods has been poor. Here, we used a polymerase chain reaction sequence-based typing (PCR-SBT) method to sequence exon 2 of the BoLA class II DRB3 gene from 421 cattle (116 Bolivian Nellore, 110 Bolivian Gir, and 195 Peruvian Nellore-Brahman). Data from 1416 Taurine and Zebu samples were also included in the analysis. Results: We identified 46 previously reported alleles and no novel variants. Of note, 1/3 of the alleles were detected only in Zebu cattle. Comparison of the degree of genetic variability at the population and sequence levels with genetic distance in the three above mentioned breeds and nine previously reported breeds revealed that Zebu breeds had a gene diversity score higher than 0.86, a nucleotide diversity score higher than 0.06, and a mean number of pairwise differences greater than 16, being similar to those estimated for other cattle breeds. A neutrality test revealed that only Nellore-Brahman cattle showed the even gene frequency distribution expected under a balanced selection scenario. The F-ST index and the exact G test showed significant differences across all cattle populations (F-ST = 0..057; p < 0.001). Neighbor-joining trees and principal component analysis identified two major clusters: one comprising mainly European Taurine breeds and a second comprising Zebu breeds. This is consistent with the historical and geographical origin of these breeds. Some of these differences may be explained by variation of ammo acid motifs at antigen-binding sites. Conclusions: The results presented herein show that the historical divergence between Taurine and Zebu cattle breeds is a result of origin, selection, and adaptation events, which would explain the observed differences in BoLA DRB3 gene diversity between the two major bovine types. This allelic information will be important for investigating the relationship between the major histocompatibility complex and disease, and contribute to an ongoing effort to catalog bovine MHC allele frequencies according to breed and location. | NA | no | dometicated host | NA | no | no |
| 5679 | 2020 | 44112 | Talagrand-Reboul, E; Latif-Eugenin, F; Beaz-Hidalgo, R; Colston, S; Figueras, MJ; Graf, J; Jumas-Bilak, E; Lamy, B | 2018 | Genome-driven evaluation and redesign of PCR tools for improving the detection of virulence-associated genes in aeromonads | Many virulence factors have been described for opportunistic pathogens within the genus Aeromonas. Polymerase Chain Reactions (PCRs) are commonly used in population studies of aeromonads to detect virulence-associated genes in order to better understand the epidemiology and emergence of Aeromonas from the environment to host, but their performances have never been thoroughly evaluated. We aimed to determine diagnostic sensitivity and specificity of PCR assays for the detection of virulence-associated genes in a collection of Aeromonas isolates representative for the genetic diversity in the genus. Thirty-nine Aeromonas strains belonging to 27 recognized species were screened by published PCR assays for virulence-associated genes (act, aerA, aexT, alt, ascFG, ascV, ast, lafA, lip, ser, stx1, stx2A). In parallel, homologues of the 12 putative virulence genes were searched from the genomes of the 39 strains. Of the 12 published PCR assays for virulence factors, the comparison of PCR results and genome analysis estimated diagnostic sensitivities ranging from 34% to 100% and diagnostic specificities ranged from 71% to 100% depending upon the gene. To improve the detection of virulence-associated genes in aeromonads, we have designed new primer pairs for aerA/act, ser, lafA, ascFG and ascV, which showed excellent diagnostic sensitivity and specificity. Altogether, the analysis of high quality genomic data, which are more and more easy to obtain, provides significant improvements in the genetic detection of virulence factors in bacterial strains. | NA | no | No good fitness measure and measure host GD | NA | no | no |
| 5680 | 2020 | 44113 | Talbot, B; Vonhof, MJ; Broders, HG; Fenton, B; Keyghobadi, N | 2018 | Host association influences variation at salivary protein genes in the bat ectoparasite Cimex adjunctus | Parasite-host relationships create strong selection pressures that can lead to adaptation and increasing specialization of parasites to their hosts. Even in relatively loose host-parasite relationships, such as between generalist ectoparasites and their hosts, we may observe some degree of specialization of parasite populations to one of the multiple potential hosts. Salivary proteins are used by blood-feeding ectoparasites to prevent hemostasis in the host and maximize energy intake. We investigated the influence of association with specific host species on allele frequencies of salivary protein genes in Cimex adjunctus, a generalist blood-feeding ectoparasite of bats in North America. We analysed two salivary protein genes: an apyrase, which hydrolyses ATP at the feeding site and thus inhibits platelet aggregation, and a nitrophorin, which brings nitrous oxide to the feeding site, inhibiting platelet aggregation and vasoconstriction. We observed more variation at both salivary protein genes among parasite populations associated with different host species than among populations from different spatial locations associated with the same host species. The variation in salivary protein genes among populations on different host species was also greater than expected under a neutral scenario of genetic drift and gene flow. Finally, host species was an important predictor of allelic divergence in genotypes of individual C.adjunctus at both salivary protein genes. Our results suggest differing selection pressures on these two salivary protein genes in C.adjunctus depending on the host species. | NA | no | No good fitness measure and measure host GD | NA | no | no |
| 5681 | 2020 | 44111 | Tang, H; Hood, ME; Ren, ZX; Li, HD; Zhao, YH; Wolfe, LM; Li, DZ; Wang, H | 2019 | Specificity and seasonal prevalence of anther smut disease Microbotryum on sympatric Himalayan Silene species | Host sympatry provides opportunities for cross-species disease transmission and compounded disease effects on host population and community structure. Using the Silene-Microbotryum interaction (the castrating anther smut disease), eleven Himalayan Silene species were assessed in regions of high host diversity to ascertain levels of pathogen specificity. We also investigated disease prevalence, seasonal dynamics of infection and flowering patterns in five co-blooming Silene species. We identified several new Microbotryum lineages with varying degrees of specialization that is likely influenced by degrees of host divergence and ecological similarities (i.e. shared pollinator guilds). Affected species had 15%-40% of plants infected by anther smut. Flowering was seasonally overlapping among host species (except for the species pair S. asclepiadea and S. atrocastanea), but diseased flowering onset was earlier than healthy plants, leading to dramatic seasonal shifts in observed disease prevalence. Overlapping distributions and flowering provides opportunities for floral pathogen movement between host species, but host specialization may be constrained by the plant phylogenetic relatedness, adaptation to micro-habitats and difference in pollinator/vector guilds. | NA | no | No good fitness measure and measure host GD | NA | no | no |
| 5682 | 2020 | 44040 | Tang, L; Zhou, YJ; Zhu, SL; Liang, GD; Zhuang, H; Zhao, MF; Chang, XY; Li, HN; Liu, Z; Guo, ZR; Liu, WQ; He, XY; Wang, CX; Zhao, DD; Li, JJ; Mu, XQ; Yao, BQ; Li, X; Li, YG; Duo, LB; Wang, L; Johnston, RN; Zhou, J; Zhao, JB; Liu, GR; Liu, SL | 2020 | E. coli diversity: low in colorectal cancer | Background Escherichia coli are mostly commensals but also contain pathogenic lineages. It is largely unclear whether the commensal E. coli as the potential origins of pathogenic lineages may consist of monophyletic or polyphyletic populations, elucidation of which is expected to lead to novel insights into the associations of E. coli diversity with human health and diseases. Methods Using genomic sequencing and pulsed field gel electrophoresis (PFGE) techniques, we analyzed E. coli from the intestinal microbiota of three groups of healthy individuals, including preschool children, university students, and seniors of a longevity village, as well as colorectal cancer (CRC) patients, to probe the commensal E. coli populations for their diversity. Results We delineated the 2280 fresh E. coli isolates from 185 subjects into distinct genome types (genotypes) by PFGE. The genomic diversity of the sampled E. coli populations was so high that a given subject may have multiple genotypes of E. coli, with the general diversity within a host going up from preschool children through university students to seniors. Compared to the healthy subjects, the CRC patients had the lowest diversity level among their E. coli isolates. Notably, E. coli isolates from CRC patients could suppress the growth of E. coli bacteria isolated from healthy controls under nutrient-limited culture conditions. Conclusions The coexistence of multiple E. coli lineages in a host may help create and maintain a microbial environment that is beneficial to the host. As such, the low diversity of E. coli bacteria may be associated with unhealthy microenvironment in the intestine and hence facilitate the pathogenesis of diseases such as CRC. | NA | no | No good fitness measure and measure host GD | NA | no | no |
| 5683 | 2020 | 44039 | Tatiya-aphiradee, N; Chatuphonprasert, W; Jarukamjorn, K | 2020 | Oxidative stress exacerbates dextran sulfate sodium-induced ulcerative colitis in ICR mice | Ulcerative colitis (UC) is a complex, multifactorial disorder which can be aggravated by oxidative stress. Dextran sulfate sodium (DSS)-induced UC in inbred mice is the most commonly used animal-model. However, these populations lack genetic variability therefore this study aimed to establish a DSS-induced UC model in outbred ICR mice and to assess its association with the oxidant-antioxidant system. Male ICR mice were administered a 40 kDa-DSS solution (6 g/kg/day; 0.25 mL 21% DSS 4 times per day) intragastrically for 4 and 7 consecutive days (n = 5). Disease activity index (DAI) was determined daily from body weight and stool characteristics. At the end of the study, colons were collected to examine histology, myeloperoxidase activity, and expression of superoxide dismutase 1 and 2 (Sod1 and Sod2) and catalase (Cat). DSS-treated mice demonstrated daily increases in DAI score with significantly shortened colons, an indirect marker of inflammation in UC. Histological examination of DSS-treated colons revealed goblet cell loss, crypt damage, and epithelial erosion, followed by infiltration of mast cells to the mucosa with mucin depletion. Myeloperoxidase activity was elevated in the DSS-treated mice while Sod1, Sod2, and Cat were suppressed compared to controls. A DSS-induced UC model was established in ICR mice with associated oxidative stress triggering. This outbred-mouse model could prove useful for investigating the underlying pathophysiological mechanisms of UC in terms of oxidant-antioxidant balance. | NA | no | No group level | NA | no | no |
| 5684 | 2020 | 44039 | Tay, GK; Henschel, A; Elbait, GD; Al Safar, HS | 2020 | Genetic Diversity and Low Stratification of the Population of the United Arab Emirates | With high consanguinity rates on the Arabian Peninsula, it would not have been unexpected if the population of the United Arab Emirates (UAE) was shown to be relatively homogenous. However, this study of 1000 UAE nationals provided a contrasting perspective, one of a relatively heterogeneous population. Located at the apex of Europe, Asia, and Africa, the observed diversity could be explained by a plethora of migration patterns since the first Out-of-Africa movement. A strategy to explore the extent of genetic variation of the population of the UAE is presented. The first step involved a comprehensive population stratification study that was instructive for subsequent whole genome sequencing (WGS) of suitable representatives (which is described elsewhere). When these UAE data were compared to previous smaller studies from the region, the findings were consistent with a population that is a diverse and admixed group of people. However, rather than sharp and distinctive clusters, cluster analysis reveals low levels of stratification throughout the population. UAE emirates exhibit high within-Emirate-distance/among-Emirate distance ratios. Supervised admixture analysis showed a continuous gradient of ancestral populations, suggesting that admixture on the south eastern tip of the Arabian Peninsula occurred gradually. When visualized using a unique technique that combined admixture ratios and principal component analysis (PCA), unappreciated diversity was revealed while mitigating projection bias of conventional PCA. We observe low population stratification in the UAE in terms of homozygosity versus separation cluster coefficients. This holds for the UAE in a global context as well as for isolated cluster analysis of the Emirati birthplaces. However, the subtle clustering observed in the Emirates reflects geographic proximity and historic migration events. The analytical strategy used here highlights the complementary nature of data from genotype array and WGS for anthropological studies. Specifically, genotype array data were instructive to select representative subjects for WGS. Furthermore, from the 2.3 million allele frequencies obtained from genotype arrays, we identified 46,481 loci with allele frequencies that were significantly different with respect to other world populations. This comparison of allele frequencies facilitates variant prioritization in common diseases. In addition, these loci bear great potential as biomarkers in anthropological and forensic studies. | NA | no | Human | NA | no | no |
| 5685 | 2020 | 44042 | Taylor, AR; Jacob, PE; Neafsey, DE; Buckee, CO | 2019 | Estimating Relatedness Between Malaria Parasites | Understanding the relatedness of individuals within or between populations is a common goal in biology. Increasingly, relatedness features in genetic epidemiology studies of pathogens. These studies are relatively new compared to those in humans and other organisms, but are important for designing interventions and understanding pathogen transmission. Only recently have researchers begun to routinely apply relatedness to apicomplexan eukaryotic malaria parasites, and to date have used a range of different approaches on an ad hoc basis. Therefore, it remains unclear how to compare different studies and which measures to use. Here, we systematically compare measures based on identity-by-state (IBS) and identity-by-descent (IBD) using a globally diverse data set of malaria parasites, Plasmodium falciparum and P. vivax, and provide marker requirements for estimates based on IBD. We formally show that the informativeness of polyallelic markers for relatedness inference is maximized when alleles are equifrequent. Estimates based on IBS are sensitive to allele frequencies, which vary across populations and by experimental design. For portability across studies, we thus recommend estimates based on IBD. To generate estimates with errors below an arbitrary threshold of 0.1, we recommend similar to 100 polyallelic or 200 biallelic markers. Marker requirements are immediately applicable to haploid malaria parasites and other haploid eukaryotes. C.I.s facilitate comparison when different marker sets are used. This is the first attempt to provide rigorous analysis of the reliability of, and requirements for, relatedness inference in malaria genetic epidemiology. We hope it will provide a basis for statistically informed prospective study design and surveillance strategies. | NA | no | gd of pathogen | NA | no | no |
| 5686 | 2020 | 44113 | Taylor, AR; Schaffner, SF; Cerqueira, GC; Nkhoma, SC; Anderson, TJC; Sriprawat, K; Phyo, AP; Nosten, F; Neafsey, DE; Buckee, CO | 2017 | Quantifying connectivity between local Plasmodium falciparum malaria parasite populations using identity by descent | With the rapidly increasing abundance and accessibility of genomic data, there is a growing interest in using population genetic approaches to characterize fine-scale dispersal of organisms, providing insight into biological processes across a broad range of fields including ecology, evolution and epidemiology. For sexually recombining haploid organisms such as the human malaria parasite P. falciparum, however, there have been no systematic assessments of the type of data and methods required to resolve fine scale connectivity. This analytical gap hinders the use of genomics for understanding local transmission patterns, a crucial goal for policy makers charged with eliminating this important human pathogen. Here we use data collected from four clinics with a catchment area spanning approximately 120 km of the Thai-Myanmar border to compare the ability of divergence (F-ST) and relatedness based on identity by descent (IBD) to resolve spatial connectivity between malaria parasites collected from proximal clinics. We found no relationship between inter-clinic distance and FST, likely due to sampling of highly related parasites within clinics, but a significant decline in IBD-based relatedness with increasing inter-clinic distance. This association was contingent upon the data set type and size. We estimated that approximately 147 single-infection whole genome sequenced parasite samples or 222 single-infection parasite samples geno-typed at 93 single nucleotide polymorphisms (SNPs) were sufficient to recover a robust spatial trend estimate at this scale. In summary, surveillance efforts cannot rely on classical measures of genetic divergence to measure P. falciparum transmission on a local scale. Given adequate sampling, IBD-based relatedness provides a useful alternative, and robust trends can be obtained from parasite samples genotyped at approximately 100 SNPs. | NA | no | No good fitness measure and measure host GD | NA | no | no |
| 5687 | 2020 | 44113 | Teeling, EC; Vernes, SC; Davalos, LM; Ray, DA; Gilbert, MTP; Myers, E | 2018 | Bat Biology, Genomes, and the Bat1K Project: To Generate Chromosome-Level Genomes for All Living Bat Species | Bats are unique among mammals, possessing some of the rarest mammalian adaptations, including true self-powered flight, laryngeal echolocation, exceptional longevity, unique immunity, contracted genomes, and vocal learning. They provide key ecosystem services, pollinating tropical plants, dispersing seeds, and controlling insect pest populations, thus driving healthy ecosystems. They account for more than 20% of all living mammalian diversity, and their crown-group evolutionary history dates back to the Eocene. Despite their great numbers and diversity, many species are threatened and endangered. Here we announce Bat1K, an initiative to sequence the genomes of all living bat species (n similar to 1,300) to chromosome-level assembly. The Bat1K genome consortium unites bat biologists (>148 members as of writing), computational scientists, conservation organizations, genome technologists, and any interested individuals committed to a better understanding of the genetic and evolutionary mechanisms that underlie the unique adaptations of bats. Our aim is to catalog the unique genetic diversity present in all living bats to better understand the molecular basis of their unique adaptations; uncover their evolutionary history; link genotype with phenotype; and ultimately better understand, promote, and conserve bats. Here we review the unique adaptations of bats and highlight how chromosome-level genome assemblies can uncover the molecular basis of these traits. We present a novel sequencing and assembly strategy and review the striking societal and scientific benefits that will result from the Bat1K initiative. | NA | no | No good fitness measure and measure host GD | NA | no | no |
| 5688 | 2020 | 44112 | Teerlink, CC; Bernhisel, R; Cannon-Albright, LA; Rollins, MD | 2018 | A genealogical assessment of familial clustering of anorectal malformations | Familial recurrence of anorectal malformations (ARMs) has been reported in single institution case series and in two population-based studies. Here, we investigate the familial aggregation of ARMs using well-established, unbiased methods in a population genealogy of Utah. Study subjects included 255 ARM cases identified from among the two largest healthcare providers in Utah with linked genealogy data using International Classification of Diseases, Ninth Revision (ICD-9) diagnosis codes. The genealogical index of familiality (GIF) statistic, which compares the average pair-wise relatedness of cases to sets of matched controls, was used to test excess familial clustering. We also estimated relative risks (RRs) for ARM and associated phenotypes in relatives of cases adjusting for age-, sex-, and birthplace. Significant excess familial clustering was observed for all ARM subjects (GIF p < 1e-3). Significant RR estimates for ARM (RR = 15.6, p = 3.3e-6), and for almost all co-morbid birth defects previously associated with ARM, were observed among first-degree relatives of ARM case subjects. This genealogically-based population survey of familial aggregation of ARMs confirms the presence of a heritable component to ARMs and provides unbiased risk estimates to relatives of cases, which may have clinical utility. | NA | no | No good fitness measure and measure host GD | NA | no | no |
| 5689 | 2020 | 44113 | Telford, M; Navarro, A; Santpere, G | 2018 | Whole genome diversity of inherited chromosomally integrated HHV-6 derived from healthy individuals of diverse geographic origin | Human herpesviruses 6-A and -B (HHV-6A, HHV-6B) are ubiquitous in human populations worldwide. These viruses have been associated with several diseases such as multiple sclerosis, Hodgkin’s lymphoma or encephalitis. Despite of the need to understand the genetic diversity and geographic stratification of these viruses, the availability of complete viral sequences from different populations is still limited. Here, we present nine new inherited chromosomally integrated HHV-6 sequences from diverse geographical origin which were generated through target DNA enrichment on lymphoblastoid cell lines derived from healthy individuals. Integration with available HHV-6 sequences allowed the assessment of HHV-6A and -6B phylogeny, patterns of recombination and signatures of natural selection. Analysis of the intra-species variability showed differences between A and B diversity levels and revealed that the HHV-6B reference (Z29) is an uncommon sequence, suggesting the need for an alternative reference sequence. Signs of geographical variation are present and more defined in HHV-6A, while they appear partly masked by recombination in HHV-6B. Finally, we conducted a scan for signatures of selection in protein coding genes that yielded at least 6 genes (4 and 2 respectively for the A and B species) showing significant evidence for accelerated evolution, and 1 gene showing evidence of positive selection in HHV-6A. | NA | no | human | NA | no | no |
| 5690 | 2020 | 44112 | Tensen, L | 2018 | Species characteristics of felids and canids, and the number of articles published for each species between 2013 and 2017 | The data presented are related to the research article entitled Biases in wildlife and conservation research, using felids and canids as a case study available at https://doi.org/10.1016/j.gecco.2018.e00423. This data article lists species characteristics of two families of the order Carnivora, the Felidae and Canidae, and quantitatively categorizes research output for each species. The species characteristics that were included in the dataset are body size (in kg), geographic range size, IUCN species status, population trend, likelihood of being a keystone species, number of species per genus, the Evolutionary Distinctiveness (ED) score, and the Evolutionary Distinct and Globally Endangered (EDGE) score. All scientific articles that were published on felid and canid species between 2013 and 2017 were listed and subdivided into the following research topics: (1) ecology and behaviour, (2) conservation and wildlife management, (3) anatomy and physiology, (4) diseases and other health issues, (5) captive housing and artificial reproduction, (6) genetic diversity and phylogenetic structure, and (7) taxonomy and palaeoecology. All the data is made publically available. (C) 2018 The Authors. Published by Elsevier Inc. | NA | no | No good fitness measure and measure host GD | NA | no | no |
| 5691 | 2020 | 44112 | Tensen, L; Drouilly, M; van Vuljren, BJ | 2018 | Genetic structure and diversity within lethally managed populations of two mesopredators in South Africa | Jackals (Canis mesomelas) and caracals (Caracal caracal) are considered major predators of small livestock on farms across South Africa, with both species being subjected to lethal control. Lethal management (i.e., culling) can result in differential responses in the population dynamics and demography of mesopredators. We examined the potential impacts that high population turnover, due to lethal management, may have on the genetic structure and diversity of jackal and caracal populations. We included mitochondrial markers to assess the movement of maternal lineages across the landscape as a proxy for dispersal. We further employed variable microsatellite markers to quantify levels of genetic diversity and relatedness among individuals. We found that high population turnover in both species may promote compensatory dispersal, which is consistent with the high levels of genetic diversity observed in both species. Structure analysis showed that the jackal population was comprised of 2 genetic clusters, while all the caracals belonged to a single nuclear genetic cluster. The weak differentiation between the jackal clusters (FST = 0.08), and the high level of inbreeding (FIS = 0.112) in the Central Karoo cluster, suggests that they likely represent demic populations. These data can be used as a baseline for ongoing population monitoring. Temporal monitoring of wildlife populations that are exposed to lethal management is important for a better understanding of the long-term effects on species ecology and survival success. | NA | no | no good gd measure host pop | NA | no | no |
| 5692 | 2020 | 44113 | Tensen, L; Groom, RJ; Khuzwayo, J; van Vuuren, BJ | 2018 | The genetic tale of a recovering lion population (Panthera leo) in the Save Valley region (Zimbabwe): A better understanding of the history and managing the future | The rapid decline of the African lion (Panthera leo) has raised conservation concerns. In the Save A Valley Conservancy (SVC), in the Lowveld of Zimbabwe, lions were presumably reduced to approximately 5 to 10 individuals. After ten lions were reintroduced in 2005, the population has recovered to over 200 lions in 2016. Although the increase of lions in the SVC seems promising, a question remains whether the population is genetically viable, considering their small founding population. In this study, we document the genetic diversity in the SVC lion population using both mitochondrial and nuclear genetic markers, and compare our results to literature from other lion populations across Africa. We also tested whether genetic diversity is spatially structured between lion populations residing on several reserves in the Lowveld of Zimbabwe. A total of 42 lions were genotyped successfully for 11 microsatellite loci. We confirmed that the loss of allelic richness (probably resulting from genetic drift and small number of founders) has resulted in low genetic diversity and inbreeding. The SVC lion population was also found to be genetically differentiated from surrounding population, as a result of genetic drift and restricted natural dispersal due to anthropogenic barriers. From a conservation perspective, it is important to avoid further loss of genetic variability in the SVC lion population and maintain evolutionary potential required for future survival. Genetic restoration through the introduction of unrelated individuals is recommended, as this will increase genetic heterozygosity and improve survival and reproductive fitness in populations. | NA | no | No good fitness measure | NA | no | no |
| 5693 | 2020 | 44113 | Tewes, LJ; Michling, F; Koch, MA; Muller, C | 2018 | Intracontinental plant invader shows matching genetic and chemical profiles and might benefit from high defence variation within populations | Whereas many studies have revealed mechanisms driving plant invasions between continents, research on intracontinental range expanders is scarce. Therefore, we studied genetic, chemical and ecological traits of a range-expanding Brassicaceae, assuming that high genetic diversity should maintain chemical variation, which potentially benefits the invasion success. Moreover, we expected that within-individual defence diversity plays an essential role in biotic interactions. We compared Bunias orientalis L. plants from 16 populations of native, invasive or exotic non-invasive origin. The genetic structure was investigated by analysing the plastid DNA and amplified fragment length polymorphisms. For characterisation of the leaf chemistry, metabolic fingerprinting and profiling of glucosinolates as defence compounds were performed. The plant defence potential was tested using the generalist herbivore Mamestra brassicae. We found two major genetic lineages, which were mirrored in distinct chemical fingerprints of the plants. Genetic differentiation patterns point to a multiple introduction history of B.orientalis underlying the range expansion. Moreover, the genetic distance between individuals was correlated with the distance in chemical features. Genetic diversity tended to be reduced in potential leading edge (exotic) populations and was positively associated with quantitative metabolic diversity. Concentrations of indole glucosinolates were elevated in non-native populations, and high glucosinolate diversity was associated with low herbivore survival. Synthesis. This study suggests that the invasion success of this species may be facilitated by high chemical variation within populations. Moreover, high defence diversity within individuals of a population might be the main factor reducing herbivory and can be more important than the total concentration of defences. The combination of genetic and chemical analyses combined with bioassays revealed to be a powerful tool to study the differentiation between native and non-native populations and should be applied more often to explore intraspecific divergence. | NA | no | No good fitness measure | NA | no | no |
| 5694 | 2020 | 44042 | Thanchomnang, T; Intapan, PM; Sanpool, O; Rodpai, R; Sadaow, L; Phosuk, I; Somboonpatarakun, C; Laymanivong, S; Tourtip, S; Maleewong, W | 2019 | First molecular identification of Strongyloides fuelleborni in long-tailed macaques in Thailand and Lao People’s Democratic Republic reveals considerable genetic diversity | Strongyloides fuelleborni is a soil-transmitted nematode parasite of non-human primates. The worm is prevalent also in human populations in Africa and South-East Asia. In this study, we amplified and sequenced a portion of the 18S ribosomal RNA gene (rRNA) and of the mitochondrial cytochrome c oxidase subunit 1 (cox1) gene of Strongyloides adult males recovered from faecal samples from long-tailed macaques (Macaca fascicularis) in Thailand and Lao PDR. The prevalence in Thailand was 31.1% (55/177) and in Lao PDR it was 62.1% (41/66), with an overall prevalence of 39.5% (96/243). All 18S rRNA sequences that we obtained (n = 96) showed 100% identity with published S. fuelleborni sequences. The 96 cox1 sequences that we obtained represented 32 new haplotypes. When included with the 17 previously known haplotypes from S. fuelleborni, the cox1 sequences fell into four clusters, which had clear geographical structure. This is the first molecular confirmation of S. fuelleborni in long-tailed macaques in Thailand and Lao PDR. Clearly, awareness needs to be raised of the zoonotic potential of S. fuelleborni. A monitoring programme should be organized, taking into account the role of reservoir hosts (i.e. monkeys) in the natural background of human strongyloidiasis caused by S. fuelleborni. | NA | no | no good gd measure host | NA | no | no |
| 5695 | 2020 | 44113 | Thapa, K; Manandhar, S; Bista, M; Shakya, J; Sah, G; Dhakal, M; Sharma, N; Llewellyn, B; Wultsch, C; Waits, LP; Kelly, MJ; Hero, JM; Hughes, J; Karmacharya, D | 2018 | Assessment of genetic diversity, population structure, and gene flow of tigers (Panthera tigris tigris) across Nepal’s Terai Arc Landscape | With fewer than 200 tigers (Panthera tigris tigris) left in Nepal, that are generally confined to five protected areas across the Terai Arc Landscape, genetic studies are needed to provide crucial information on diversity and connectivity for devising an effective country-wide tiger conservation strategy. As part of the Nepal Tiger Genome Project, we studied landscape change, genetic variation, population structure, and gene flow of tigers across the Terai Arc Landscape by conducting Nepal’s first comprehensive and systematic scat-based, non-invasive genetic survey. Of the 770 scat samples collected opportunistically from five protected areas and six presumed corridors, 412 were tiger (57%). Out of ten microsatellite loci, we retain eight markers that were used in identifying 78 individual tigers. We used this data set to examine population structure, genetic variation, contemporary gene flow, and potential population bottlenecks of tigers in Nepal. We detected three genetic clusters consistent with three demographic sub-populations and found moderate levels of genetic variation (H-e = 0.61, A(R) = 3.51) and genetic differentiation (F-ST = 0.14) across the landscape. We detected 3-7 migrants, confirming the potential for dispersal-mediated gene flow across the landscape. We found evidence of a bottleneck signature likely caused by large-scale land-use change documented in the last two centuries in the Terai forest. Securing tiger habitat including functional forest corridors is essential to enhance gene flow across the landscape and ensure long-term tiger survival. This requires cooperation among multiple stakeholders and careful conservation planning to prevent detrimental effects of anthropogenic activities on tigers. | NA | no | No good fitness measure and measure host GD | NA | no | no |
| 5696 | 2020 | 44112 | Thareja, G; Mathew, S; Mathew, LS; Mohamoud, YA; Suhre, K; Malek, JA | 2018 | Genotyping-by-sequencing identifies date palm clone preference in agronomics of the State of Qatar | Understanding the genetic diversity in a crop population is key to its targeted breeding for desired traits, such as higher yields, better fruit quality and resistance to disease and changing climates. Date fruits represent a major crop in the Middle East and are key to achieving future food independence in arid countries like Qatar. We previously determined the genome of the date palm Phoenix dactylifera and showed that date palm trees world-wide divide into two distinct subpopulations of Eastern and Western origins. Here we applied a resource of SNPs from 179 commercially available date fruits to assess the genetic diversity of date palm trees grown in the State of Qatar. We found that palm trees in Qatar are mainly of Eastern origin, and that their genetic diversity doesn’t associate with regions of the State. Together with targeted genetic assays, our resource can be used in the future for date palm cultivar identification, to aid selecting suitable cultivars for targeted breeding, to improve a country’s date palm genetic diversity, and to certify the origin of date fruits and trees. | NA | no | dometicated host | NA | no | no |
| 5697 | 2020 | 44042 | Themi, PK; Chimusa, ER | 2019 | Population Structure and Implications on the Genetic Architecture of HIV-1 Phenotypes Within Southern Africa | The interesting history of Southern Africa has put the region in the spotlight for population medical genetics. Major events including the Bantu expansion and European colonialism have imprinted unique genetic signatures within autochthonous populations of Southern Africa, this resulting in differential allele frequencies across the region. This genetic structure has potential implications on susceptibility and resistance to infectious diseases such as human immunodeficiency virus (HIV) infection. Southern Africa is the region affected worst by HIV. Here, we discuss advances made in genome-wide association studies (GWAS) of HIV-1 in the past 12 years and dissect population diversity within Southern Africa. Our findings accentuate that a plethora of factors such as migration, language and culture, admixture, and natural selection have profiled the genetics of the people of Southern Africa. Genetic structure has been observed among the Khoe-San, among Bantu speakers, and between the Khoe-San, Coloureds, and Bantu speakers. Moreover, Southern African populations have complex admixture scenarios. Few GWAS of HIV-1 have been conducted in Southern Africa, with only one of these identifying two novel variants (HCG22rs2535307 and CCNG1kgp22385164) significantly associated with HIV-1 acquisition and progression. High genetic diversity, multi-wave genetic mixture and low linkage disequilibrium of Southern African populations constitute a challenge in identifying genetic variants with modest risk or protective effect against HIV-1. We therefore posit that it is compelling to assess genome-wide contribution of ancestry to HIV-1 infection. We further suggest robust methods that can pin-point population-specific variants that may contribute to the control of HIV-1 in Southern Africa. | NA | no | hiv | NA | no | no |
| 5698 | 2020 | 44112 | Theuns, S; Vanmechelen, B; Bernaert, Q; Deboutte, W; Vandenhole, M; Beller, L; Matthijnssens, J; Maes, P; Nauwynck, HJ | 2018 | Nanopore sequencing as a revolutionary diagnostic tool for porcine viral enteric disease complexes identifies porcine kobuvirus as an important enteric virus | Enteric diseases in swine are often caused by different pathogens and thus metagenomics are a useful tool for diagnostics. The capacities of nanopore sequencing for viral diagnostics were investigated here. First, cell culture-grown porcine epidemic diarrhea virus and rotavirus A were pooled and sequenced on a MinION. Reads were already detected at 7 seconds after start of sequencing, resulting in high sequencing depths (19.2 to 103.5X) after 3 h. Next, diarrheic feces of a one-week-old piglet was analyzed. Almost all reads (99%) belonged to bacteriophages, which may have reshaped the piglet’s microbiome. Contigs matched Bacteroides, Escherichia and Enterococcus phages. Moreover, porcine kobuvirus was discovered in the feces for the first time in Belgium. Suckling piglets shed kobuvirus from one week of age, but an association between peak of viral shedding (10(6.42)-10(7.01) copies/swab) and diarrheic signs was not observed during a follow-up study. Retrospective analysis showed the widespread (n = 25, 56.8% positive) of genetically moderately related kobuviruses among Belgian diarrheic piglets. MinION enables rapid detection of enteric viruses. Such new methodologies will change diagnostics, but more extensive validations should be conducted. The true enteric pathogenicity of porcine kobuvirus should be questioned, while its subclinical importance cannot be excluded. | NA | no | No good fitness measure and measure host GD | NA | no | no |
| 5699 | 2020 | 44111 | Thongpan, I; Suntronwong, N; Vichaiwattana, P; Wanlapakorn, N; Vongpunsawad, S; Poovorawan, Y | 2019 | Respiratory syncytial virus, human metapneumovirus, and influenza virus infection in Bangkok, 2016-2017 | Children and adults residing in densely populated urban centers around the world are at risk of seasonal influenza-like illness caused by respiratory viruses such as influenza virus, human metapneumovirus (hMPV), and respiratory syncytial virus (RSV). In a large metropolitan of Thailand’s capital city Bangkok, most respiratory infections are rarely confirmed by molecular diagnostics. We therefore examined the frequency of RSV, hMPV, and influenza virus in 8,842 patients who presented influenza-like illness and sought medical care at a large hospital in Bangkok between 2016 and 2017. Using a multiplex real-time reverse-transcription polymerase chain reaction (RT-PCR), 30.5% (2,699/8,842) of nasopharyngeal (NP) swab samples tested positive for one or more of these viruses. Influenza virus comprised 17.3% (1,528/8,842), of which the majority were influenza A/H3N2. Such infection was most prevalent among adults and the elderly. RSV was identified in 11.4% (1,011/8,842) and were mostly ON1 and BA9 genotypes. Of the hMPV-positive samples (3.6%, 318/8,842), genotypes A2, B1, and B2 were detected. A small number of individuals experienced co-infections (1.8%, 155/8,842), most commonly between RSV and influenza A/H3N2. RSV and hMPV co-infections were also found, but mainly in young children. Viral respiratory tract infection peaked locally in the rainy season (June to September). These findings support the utility of rapid nucleic acid testing of RSV, hMPV, and influenza virus in patients with ILI. | NA | no | gd of pathogen | NA | no | no |
| 5700 | 2020 | 44112 | Tian-Bi, YNT; Konan, JNK; Sangare, A; Ortega-Abboud, E; Utzinger, J; N’Goran, EK; Jarne, P | 2019 | Spatio-temporal population genetic structure, relative to demographic and ecological characteristics, in the freshwater snail Biomphalaria pfeifferiin Man, western Cote d’Ivoire | Combining the analysis of spatial and temporal variation when investigating population structure enhances our capacity for unravelling the biotic and abiotic factors responsible for microevolutionary change. This work aimed at measuring the spatial and temporal genetic structure of populations of the freshwater snail Biomphalaria pfeifferi (the intermediate host of the trematode Schistosoma mansoni) in relation to the mating system (self-fertilization), demography, parasite prevalence and some ecological parameters. Snail populations were sampled four times in seven human-water contact sites in the Man region, western Cote d’Ivoire, and their variability was measured at five microsatellite loci. Limited genetic diversity and high selfing rates were observed in the populations studied. We failed to reveal an effect of demographic and ecological parameters on within-population diversity, perhaps as a result of a too small number of populations. A strong spatial genetic differentiation was detected among populations. The temporal differentiation within populations was high in most populations, though lower than the spatial differentiation. All estimates of effective population size were lower than seven suggesting a strong effect of genetic drift. However, the genetic drift was compensated by high gene flow. The genetic structure within and among populations reflected that observed in other selfing snail species, relying on high selfing rates, low effective population sizes, environmental stochasticity and high gene flow. | NA | no | No good fitness measure | NA | no | no |
| 5701 | 2020 | 44113 | Tihon, E; Imamura, H; Dujardin, JC; Van Den Abbeele, J; Van den Broeck, F | 2017 | Discovery and genomic analyses of hybridization between divergent lineages of Trypanosoma congolense, causative agent of Animal African Trypanosomiasis | Hybrid populations and introgressive hybridization remain poorly documented in pathogenic micro-organisms, as such that genetic exchange has been argued to play a minor role in their evolution. Recent work demonstrated the existence of hybrid microsatellite profiles in Trypanosoma congolense, a parasitic protozoan with detrimental effects on livestock productivity in sub-Saharan Africa. Here, we present the first population genomic study of T.congolense, revealing a remarkable number of single nucleotide polymorphisms (SNPs), small insertions/deletions (indels) and gene deletions among 56 parasite genomes from ten African countries. One group of parasites from Zambia was particularly diverse, displaying a substantial number of heterozygous SNP and indel sites compared to T.congolense parasites from the nine other sub-Saharan countries. Genomewide 5-kb phylogenetic analyses based on phased SNP data revealed that these parasites were the product of hybridization between phylogenetically distinct T.congolense lineages. Other parasites within the same region in Zambia presented a mosaic of haplotypic ancestry and genetic variability, indicating that hybrid parasites persisted and recombined beyond the initial hybridization event. Our observations challenge traditional views of trypanosome population biology and encourage future research on the role of hybridization in spreading genes for drug resistance, pathogenicity and virulence. | NA | no | No good fitness measure and measure host GD | NA | no | no |
| 5702 | 2020 | 44040 | Toktay, H; Evlice, E; Imren, M; Ozer, G; Ali, MA; Dababat, A | 2020 | Characterization of Potato Golden Cyst Nematode Populations (Globodera rostochiensis) in Turkey | Golden potato cyst nematode, Globodera rostochiensis (Wollenweber) Behrens, is one of the most important soilborne pathogens causing economic losses in potato. The nematode is known to occurin several countries including Turkey and has a worldwide regulatory concern. In this study, identification and genetic diversity of G. rostochiensis specimens obtained from the main potato producing areas of Turkey were determined. Twenty -five of 35 soil samples collected from the provinces of Izmir, Nevsehir and Nigde were found to contain G. rostochiensis. The variation between G. rostochiensis populations was determined when examined according to ecological and pathogenic characteristics of nematode in Turkey. The cysts of G. rostochiensis were identified by measuring the morphological characters using perennial patterns, vulval cone, vulval basin and juveniles. Phylogenetic analysis of the Large Sub Unit (LSU) region of rDNA sequences was used to assess the inter or intra phylogenetic relationships between the nematode populations. The phylogenetic analyses demonstrated that the nematode specimens from Turkey cluster with Globodera spp. and signified the presence of single species of G. rostochiensis. As a result, morphological, morphometric and molecular methods were successfully combined for identification and characterization of G. rostochiensis. The frequency of G. rostochiensis in regulatory samples frompotato -producing areas is becoming increasingly important. The morphological characterization has several complications in the detection of this quarantine nematode, using of this combination is beneficial for a reliable and quick diagnostic for these nematodes which is crucial for regulatory services and growers. The results might help to investigate different ecotypes of G. rostochiensis for comprehensive understanding about physiology, ecology , and biology of the genus Globodera for its effective management in Turkey. (C) 2020 Friends Science Publishers | NA | no | gd of pathogen | NA | no | no |
| 5703 | 2020 | 44042 | Toledo, AV; Franco, MEE; Medina, R; Lenicov, AMMD; Balatti, PA | 2019 | Assessment of the genetic diversity of Argentinean isolates of Beauveria bassiana (Ascomycota: Hypocreales) using ISSR markers | Recombination mediated through a parasexual cycle is a potential source of genetic diversity among mitosporic fungi such as Beauveria bassiana. Knowledge of this diversity is critical to understand the structure of the population. Therefore, the aim of this study was to analyze the genetic diversity within 36 Argentinean isolates of B. bassiana collected from different geographical regions and insect hosts; and to assess by means of ITS sequences the preliminary taxonomic position of the isolates that might be related to the phenotypic and genotypic differences. The results demonstrate that the fingerprints generated with four ISSR markers were enough to detect significant genetic diversity among Argentinean isolates, which was unrelated neither with the geographical origin nor with insect hosts. (C) 2018 The Authors. Production and hosting by Elsevier B.V. on behalf of King Saud University. | NA | no | No good fitness measure and measure host GD | NA | no | no |
| 5704 | 2020 | 44112 | Torquet, N; Marti, F; Campart, C; Tolu, S; Nguyen, C; Oberto, V; Benallaoua, M; Naude, J; Didienne, S; Debray, N; Jezequel, S; Le Gouestre, L; Hannesse, B; Mariani, J; Mourot, A; Faure, P | 2018 | Social interactions impact on the dopaminergic system and drive individuality | Individuality is a striking feature of animal behavior. Individual animals differ in traits and preferences which shape their interactions and their prospects for survival. However, the mechanisms underlying behavioral individuation are poorly understood and are generally considered to be genetic-based. Here, we devised a large environment, Souris City, in which mice live continuously in large groups. We observed the emergence of individual differences in social behavior, activity levels, and cognitive traits, even though the animals had low genetic diversity (inbred C57BL/6J strain). We further show that the phenotypic divergence in individual behaviors was mirrored by developing differences in midbrain dopamine neuron firing properties. Strikingly, modifying the social environment resulted in a fast re-adaptation of both the animal’s traits and its dopamine firing pattern. Individuality can rapidly change upon social challenges, and does not just depend on the genetic status or the accumulation of small differences throughout development. | NA | no | No good fitness measure and measure host GD | NA | no | no |
| 5705 | 2020 | 44042 | Toth, EG; Tremblay, F; Housset, JM; Bergeron, Y; Carcaillet, C | 2019 | Geographic isolation and climatic variability contribute to genetic differentiation in fragmented populations of the long-lived subalpine conifer Pinus cembra L. in the western Alps | Background Genetic processes shape the modern-day distribution of genetic variation within and between populations and can provide important insights into the underlying mechanisms of evolution. The resulting genetic variation is often unequally partitioned within species’ distribution range and especially large differences can manifest at the range limit, where population fragmentation and isolation play a crucial role in species survival. Despite several molecular studies investigating the genetic diversity and differentiation of European Alpine mountain forests, the climatic and demographic constrains which influence the genetic processes are often unknown. Here, we apply non-coding microsatellite markers to evaluate the sporadic peripheral and continuous populations of cembra pine (Pinus cembra L.), a long-lived conifer species that inhabits the subalpine treeline ecotone in the western Alps to investigate how the genetic processes contribute to the modern-day spatial distribution. Moreover, we corroborate our findings with paleoecological records, micro and macro-remains, to infer the species’ possible glacial refugia and expansion scenarios. Results Four genetically distinct groups were identified, with Bayesian and F-ST based approaches, across the range of the species, situated in the northern, inner and south-western Alps. We found that genetic differentiation is substantially higher in marginal populations than at the center of the range, and marginal stands are characterized by geographic and genetic isolation due to spatial segregation and restricted gene flow. Moreover, multiple matrix regression approaches revealed effects of climatic heterogeneity in species’ spatial genetic pattern. Also, population stability tests indicated that all populations had experienced a severe historical bottleneck, no heterozygosity excess was detected, suggesting that more recently population sizes have remained relatively stable. Conclusions Our study demonstrated that cembra pine might have survived in multiple glacial refugia and subsequently recolonized the Alps by different routes. Modern-day marginal populations, at the edge of the species’ range, could maintain stable sizes over long periods without inbreeding depression and preserve high amounts of genetic variation. Moreover, our analyses indicate that climatic variability has played a major role in shaping differentiation, in addition to past historical events such as migration and demographic changes. | NA | no | No good fitness measure | NA | no | no |
| 5706 | 2020 | 44039 | Touchon, M; Perrin, A; de Sousa, JAM; Vangchhia, B; Burn, S; O’Brien, CL; Denamur, E; Gordon, D; Rocha, EP | 2020 | Phylogenetic background and habitat drive the genetic diversification ofEscherichia coli | Escherichia coliis mostly a commensal of birds and mammals, including humans, where it can act as an opportunistic pathogen. It is also found in water and sediments. We investigated the phylogeny, genetic diversification, and habitat-association of 1,294 isolates representative of the phylogenetic diversity of more than 5,000 isolates from the Australian continent. Since many previous studies focused on clinical isolates, we investigated mostly other isolates originating from humans, poultry, wild animals and water. These strains represent the species genetic diversity and reveal widespread associations between phylogroups and isolation sources. The analysis of strains from the same sequence types revealed very rapid change of gene repertoires in the very early stages of divergence, driven by the acquisition of many different types of mobile genetic elements. These elements also lead to rapid variations in genome size, even if few of their genes rise to high frequency in the species. Variations in genome size are associated with phylogroup and isolation sources, but the latter determine the number of MGEs, a marker of recent transfer, suggesting that gene flow reinforces the association of certain genetic backgrounds with specific habitats. After a while, the divergence of gene repertoires becomes linear with phylogenetic distance, presumably reflecting the continuous turnover of mobile element and the occasional acquisition of adaptive genes. Surprisingly, the phylogroups with smallest genomes have the highest rates of gene repertoire diversification and fewer but more diverse mobile genetic elements. This suggests that smaller genomes are associated with higher, not lower, turnover of genetic information. Many of these genomes are from freshwater isolates and have peculiar traits, including a specific capsule, suggesting adaptation to this environment. Altogether, these data contribute to explain why epidemiological clones tend to emerge from specific phylogenetic groups in the presence of pervasive horizontal gene transfer across the species. Author summary Previous large scale studies on the evolution ofE.colifocused on clinical isolates emphasizing virulence and antibiotic resistance in medically important lineages. Yet, mostE.colistrains are either human commensals or not associated with humans at all. Here, we analyzed a large collection of non-clinical isolates of the species to assess the mechanisms of gene repertoire diversification in the light of isolation sources and phylogeny. We show that gene repertoires evolve so rapidly by the high turnover of mobile genetic elements that epidemiologically indistinguishable strains can be phenotypically extremely heterogeneous, illustrating the velocity of bacterial adaptation and the importance of accounting for the information on the whole genome at the epidemiological scale. Phylogeny and habitat shape the genetic diversification ofE.colito similar extents. Surprisingly, freshwater strains seem specifically adapted to this environment, breaking the paradigm thatE.colienvironmental isolates are systematically fecal contaminations. As a consequence, the evolution of this species is also shaped by environmental habitats, and it may diversify by acquiring genes and mobile elements from environmental bacteria (and not just from gut bacteria). This may facilitate the acquisition of virulence factors and antibiotic resistance in the strains that become pathogenic. | NA | no | No good fitness measure and measure host GD | NA | no | no |
| 5707 | 2020 | 44111 | Townsend, AK; Taff, CC; Jones, ML; Getman, KH; Wheeler, SS; Hinton, MG; Logsdon, RM | 2019 | Apparent inbreeding preference despite inbreeding depression in the American crow | Although matings between relatives can have negative effects on offspring fitness, apparent inbreeding preference has been reported in a growing number of systems, including those with documented inbreeding depression. Here, we examined evidence for inbreeding depression and inbreeding preference in two populations (Clinton, New York, and Davis, California, USA) of the cooperatively breeding American crow (Corvus brachyrhynchos). We then compared observed inbreeding strategies with theoretical expectations for optimal, adaptive levels of inbreeding, given the inclusive fitness benefits and population-specific magnitude of inbreeding depression. We found that low heterozygosity at a panel of 33 microsatellite markers was associated with low survival probability (fledging success) and low white blood cell counts among offspring in both populations. Despite these costs, our data were more consistent with inbreeding preference than avoidance: The observed heterozygosity among 396 sampled crow offspring was significantly lower than expected if local adults were mating by random chance. This pattern was consistent across a range of spatial scales in both populations. Adaptive levels of inbreeding, given the magnitude of inbreeding depression, were predicted to be very low in the California population, whereas complete disassortative mating was predicted in the New York population. Sexual conflict might have contributed to the apparent absence of inbreeding avoidance in crows. These data add to an increasing number of examples of an inbreeding paradox, where inbreeding appears to be preferred despite inbreeding depression. | NA | no | dometicated host | NA | no | no |
| 5708 | 2020 | 44112 | Townsend, AK; Taff, CC; Wheeler, SS; Weis, AM; Hinton, MG; Jones, ML; Logsdon, RM; Reisen, WK; Freund, D; Sehgal, RNM; Saberi, M; Suh, YH; Hurd, J; Boyce, WM | 2018 | Low heterozygosity is associated with vector-borne disease in crows | Infectious diseases can have devastating impacts on wildlife populations and are of particular concern for small, inbred populations. Identifying specific pathogens that are linked to morbidity and mortality in inbred individuals is a priority for the conservation of small populations, but opportunities to examine them in the wild are rare. Here, we examined the relationship between heterozygosity and infectious disease in American crows (Corvus brachyrhynchos), a species that engages in close inbreeding, focusing on three pathogens common in Davis, California, USA: West Nile virus (WNV), Plasmodium spp. (avian malaria), and Campylobacter jejuni. We found that low heterozygosity at a panel of 33 microsatellite loci was associated with two vector-borne infectious diseases (WNV and avian malaria), but not with infection by the bacterial gut pathogen C. jejuni. Reasons for this association with vector-borne pathogens are unclear, but might include behavioral factors and immunological differences associated with inbreeding. Overall, these data are consistent with the idea that inbred individuals may be more susceptible to both novel and endemic vector-borne pathogens, underscoring the importance of protecting genetic diversity within populations and buffering small populations against infectious diseases. | NA | no | No good fitness measure and measure host GD | NA | no | no |
| 5709 | 2020 | 44112 | Traore, K; Konate, S; Thera, MA; Niangaly, A; Ba, A; Niare, A; Arama, C; Di Cristofaro, J; Baby, M; Picot, S; Chiaroni, J; Boetsch, G; Doumbo, OK | 2019 | Genetic polymorphisms with erythrocyte traits in malaria endemic areas of Mali | African populations are characterized by high degree of genetic diversity. This high genetic diversity could result from the natural selection pressure. Several studies have described an association between some genetic diversities and difference of susceptibility to infectious diseases like malaria. It seems therefore important to consider genetic diversity impact when interpreting results of clinical trials in malaria endemic areas. This study aimed to determine the genetic polymorphism with erythrocyte traits in different populations of malaria endemic area in Mali. The cross-sectional surveys were carried out in different ethnic groups living in malaria endemic areas in Mali. Six milliliters of whole blood were collected in EDTA vials from each participant after informed consent has been obtained. The ABO, RH, Kell, MNSs, Kidd and Duffy systems phenotypes were assessed by the technique of gel filtration. A total of 231 subjects were included from six villages. The blood groups phenotypes O (40.7%) and A (31.2%) were more frequent with respective allele frequencies of 0.65 and 0.21. In the RH system the haplotypes R0 (0.55), r (0.20) and R1 (0.13) were the most frequent. Seven percent (7%) of Duffy positive and 4% of Glycophorin B deficiency (S-s-) were observed among participants. All participants were Kell negative. ABO and RH systems were polymorphic in these ethnic groups in Mali. Their implication in susceptibility to malaria should be taken into account in clinical trials interpretation, and for prevention of blood transfusion risks during anemia frequently caused by malaria in children. | NA | no | No good fitness measure and measure host GD | NA | no | no |
| 5710 | 2020 | 44112 | Troianou, E; Huisman, J; Pemberton, JM; Walling, CA | 2018 | Estimating selection on the act of inbreeding in a population with strong inbreeding depression | Inbreeding depression is widely regarded as a driving force in the evolution of dispersal, mate choice and sperm selection. However, due to likely costs of inbreeding avoidance, which are poorly understood, it is unclear to what extent selection to avoid inbreeding is expected in nature. Moreover, there are currently very few empirical estimates of the strength of selection against the act of inbreeding (mating with a relative), as opposed to the fitness costs of being inbred. Here, we use data from the individual-based study of red deer on the Scottish island of Rum, a strongly polygynous system which harbours a large inbreeding load, to estimate selection against the act of inbreeding for each sex. We use pedigree and genomic estimates of relatedness between individuals and measure fitness using both lifetime breeding success (number of calves born) and lifetime reproductive success (number of calves surviving to independence), with the latter incorporating inbreeding depression in calf survival. We find for both sexes that the repeatability of the act of inbreeding was low (< 0.1), suggesting little among-individual variation for this trait on which selection can act. Using the genomic measures, there was significant selection against the act of inbreeding in males, but not in females, and there was considerable uncertainty in the estimate in both sexes. We discuss possible explanations for these patterns and their implications for understanding the evolution of inbreeding avoidance in natural populations. | NA | no | no good gd measure host pop | NA | no | no |
| 5711 | 2020 | 44113 | Troupin, C; Picard-Meyer, E; Dellicour, S; Casademont, I; Kergoat, L; Lepelletier, A; Dacheux, L; Baele, G; Monchatre-Leroy, E; Cliquet, F; Lemey, P; Bourhy, H | 2017 | Host Genetic Variation Does Not Determine Spatio-Temporal Patterns of European Bat 1 Lyssavirus | The majority of bat rabies cases in Europe are attributed to European bat 1 lyssavirus (EBLV-1), circulating mainly in serotine bats (Eptesicus serotinus). Two subtypes have been defined (EBLV-1a and EBLV-1b), each associated with a different geographical distribution. In this study, we undertake a comprehensive sequence analysis based on 80 newly obtained EBLV-1 nearly complete genome sequences from nine European countries over a 45-year period to infer selection pressures, rates of nucleotide substitution, and evolutionary time scale of these two subtypes in Europe. Our results suggest that the current lineage of EBLV-1 arose in Europe similar to 600 years ago and the virus has evolved at an estimated average substitution rate of similar to 4.19 x 10(-5) subs/site/year, which is among the lowest recorded for RNA viruses. In parallel, we investigate the genetic structure of French serotine bats at both the nuclear and mitochondrial level and find that they constitute a single genetic cluster. Furthermore, Mantel tests based on interindividual distances reveal the absence of correlation between genetic distances estimated between viruses and between host individuals. Taken together, this indicates that the genetic diversity observed in our E. serotinus samples does not account for EBLV-1a and -1b segregation and dispersal in Europe. | NA | no | No good fitness measure and measure host GD | NA | no | no |
| 5712 | 2020 | 44113 | Tuanthap, S; Phupolphan, C; Luengyosluechakul, S; Duang-in, A; Theamboonlers, A; Wattanaphansak, S; Vongpunsawad, S; Amonsin, A; Poovorawan, Y | 2018 | Porcine rotavirus C in pigs with gastroenteritis on Thai swine farms, 2011-2016 | Swine are economically important food animals, but highly contagious porcine epidemic diarrhea virus (PEDV) and rotavirus can afflict pig herds and contribute significantly to piglet morbidity and mortality. While there have been studies on rotavirus group A (RVA) in Thailand, reports of rotavirus group C (RVC) are limited. Here, we aimed to identify the prevalence of RVC circulating on Thai commercial swine farms. We analyzed 769 feces and intestine mucosal contents of pigs affected with diarrhea between 2011 and 2016 using RT-PCR specific for the PEDV spike (S), rotavirus glycoprotein (G) VP7, and protease-sensitive protein (P) VP4 genes. We found that 6.6% (51/769) of samples tested positive for RVC, of which 11 samples were co-infected with RVA and four samples were co-infected with PEDV. Three samples tested positive for all three viruses. Phylogenetic analysis of the VP7 gene showed that the most frequent RVC genotype was G1, which grouped with the prototypic RVC Cowden strain. While G6 and G9 were also common, G3 was relatively rare. Analysis of the VP4 gene revealed that the most common P type was P[5], followed by P[4], P[7], and P[1]. In all, there were six G/P combinations (G6P[5], G1P[1], G1P[4], G1P[5], G9P[4], and G9P[7]), of which G6P[5] was the most predominant. | NA | no | dometicated host | NA | no | no |
| 5713 | 2020 | 44112 | Tuanthap, S; Vongpunsawad, S; Luengyosluechakul, S; Sakkaew, P; Theamboonlers, A; Amonsin, A; Poovorawan, Y | 2019 | Genome constellations of 24 porcine rotavirus group A strains circulating on commercial Thai swine farms between 2011 and 2016 | Rotavirus A (RVA) infection is a major cause of diarrhea-related illness in young children. RVA is also one of the most common enteric viruses detected on pig farms and contributes to substantial morbidity and mortality in piglets. Long-term multi-site surveillance of RVA on Thai swine farms to determine the diversity of RVA strains in circulation is currently lacking. In this study, we characterized the 11 segments of the RVA genome from 24 Thai porcine RVA strains circulating between 2011 and 2016. We identified G9 (15/24) and P[13] (12/24) as the dominant genotypes. The dominant G and P combinations were G9P[13] (n = 6), G9P[23] (n = 6), G3P[13] (n = 5), G9P[19] (n = 3), G4P[6] (n = 2), G4P[19] (n = 1), and G5P [13] (n = 1). Genome constellation of the Thai strains showed the predominance of Wa-like genotype (Gx-P[x]-11/15-R1-C1-M1-A8-N1-T1/T7-E1/E9-H1) with evidence of reassortment between the porcine and human RVA strains (e.g., G4-P[6]-I1-R1-C1-M1-A8-N1-T1-E1-H1 and G9-P[19]-15-R1-C1-M1-A8-N1-T7-E9-H1). To assess the potential effectiveness of rotavirus vaccination, the Thai RVA strains were compared to the RVA strains represented in the swine rotavirus vaccine, which showed residue variations in the antigenic epitope on VP7 and shared amino acid identity below 90% for G4 and G5 strain. Several previous studies suggested these variations might effect on virus neutralization specificity and vaccine efficacy. Our study illustrates the importance of RVA surveillance beyond the G/P genotyping on commercial swine farms, which is crucial for controlling viral transmission. | NA | no | gd of pathogen | NA | no | no |
| 5714 | 2020 | 44112 | Tuncay, SS; Roth, S; Bardakci, F; Jehle, R | 2018 | Genetic diversity of common toads (Bufo bubo) along the Norwegian coast: disjunct distribution of locally dominant haplotypes | Little is known about the phylogeographic history of amphibian populations along the western Fennoscandinavian coast. In the present study, we focus on the common toad (Bufo bufo) and document the spatial distribution of mitochondrial DNA (cytb) haplotypes at 20 localities along its coastal Norwegian range. Two common haplotypes (out of eight haplotypes in total) were represented by 142 out of the 154 (92%) investigated individuals. However, they were shared at only three localities and clustered at two separate geographic regions each. The most common haplotype (55% of individuals) has previously been found to be abundant across central and eastern Europe, whereas the second most common haplotype (37% of individuals) has so far only been recorded in Sweden. The disjunct distribution of genetic lineages is in line with an assumption that the Norwegian coastline was postglacially colonised both from the south as well as across mountain passes from the east. Our data support previous studies on the phylogeography of Fennoscandinavia that revealed that post-glacial recolonisation patterns led to a pronounced spatial structure of local populations. | NA | no | No good fitness measure | NA | no | no |
| 5715 | 2020 | 44113 | Tunstall, T; Kock, R; Vahala, J; Diekhans, M; Fiddes, I; Armstrong, J; Paten, B; Ryder, OA; Steiner, CC | 2018 | Evaluating recovery potential of the northern white rhinoceros from cryopreserved somatic cells | The critically endangered northern white rhinoceros is believed to be extinct in the wild, with the recent death of the last male leaving only two remaining individuals in captivity. Its extinction would appear inevitable, but the development of advanced cell and reproductive technologies such as cloning by nuclear transfer and the artificial production of gametes via stem cells differentiation offer a second chance for its survival. In this work, we analyzed genome-wide levels of genetic diversity, inbreeding, population history, and demography of the white rhinoceros sequenced from cryopreserved somatic cells, with the goal of informing how genetically valuable individuals could be used in future efforts toward the genetic rescue of the northern white rhinoceros. We present the first sequenced genomes of the northern white rhinoceros, which show relatively high levels of heterozygosity and an average genetic divergence of 0.1% compared with the southern subspecies. The two white rhinoceros subspecies appear to be closely related, with low genetic admixture and a divergent time <80,000 yr ago. Inbreeding, as measured by runs of homozygosity, appears slightly higher in the southern than the northern white rhinoceros. This work demonstrates the value of the northern white rhinoceros cryopreserved genetic material as a potential gene pool for saving this subspecies from extinction. | NA | no | No good fitness measure and measure host GD | NA | no | no |
| 5716 | 2020 | 44039 | Turzhanova, A; Khapilina, ON; Tumenbayeva, A; Shevtsov, V; Raiser, O; Kalendar, R | 2020 | Genetic diversity of Alternaria species associated with black point in wheat grains | The genus Alternaria is a widely distributed major plant pathogen that can act as a saprophyte in plant debris. Fungi of this genus frequently infect cereal crops and cause such diseases as black point and wheat leaf blight, which decrease the yield and quality of cereal products. A total of 25 Alternaria sp. isolates were collected from germ grains of various wheat cultivars from different geographic regions in Kazakhstan. We investigated the genetic relationships of the main Alternaria species related to black point disease of wheat in Kazakhstan, using the inter-primer binding site (iPBS) DNA profiling technique. We used 25 retrotransposon-based iPBS primers to identify the differences among and within Alternaria species populations, and analyzed the variation using clustering (UPGMA) and statistical approaches (AMOVA). Isolates of Alternaria species clustered into two main genetic groups, with species of A. alternata and A. tennuissima forming one cluster, and isolates of A. infectoria forming another. The genetic diversity found using retrotransposon profiles was strongly correlated with geographic data. Overall, the iPBS fingerprinting technique is highly informative and useful for the evaluation of genetic diversity and relationships of Alternaria species. | NA | no | No good fitness measure and measure host GD | NA | no | no |
| 5717 | 2020 | 44111 | Ueki, M; Fujihara, J; Kimura-Kataoka, K; Yamada, K; Takinami, Y; Takeshita, H; Iida, R; Yasuda, T | 2019 | Low genetic heterogeneity of copy number variations (CNVs) in the genes encoding the human deoxyribonucleases 1-like 3 and II potentially relevant to autoimmunity | Deoxyribonucleases (DNases) might play a role in prevention of autoimmune conditions such as systemic lupus erythematosus through clearance of cell debris resulting from apoptosis and/ or necrosis. Previous studies have suggested that variations in the in vivo activities of DNases I-like 3(1L3) and II have an impact on autoimmune-related conditions. The genes for these DNases are known to show copy number variations (CNVs) whereby copy loss leads to a reduction of the in vivo activities of the enzymes, thereby possibly affecting the pathophysiological background of autoimmune diseases. Using a simple newly developed quantitative real-time PCR method, we investigated the distributions of the CNVs for DNASE1L3 and DNASE2 in Japanese and German populations. It was found that only 2 diploid copy numbers for all of these DNASE CNVs was distributed in both of the study populations; no copy loss or gain was evident for any of the autoimmune-related DNase genes. Therefore, it was demonstrated that these human autoimmune-related DNase genes show low genetic diversity of CNVs resulting in alterations of the in vivo levels of DNase activity. | NA | no | No good fitness measure and measure host GD | NA | no | no |
| 5718 | 2020 | 44112 | Ul Haq, I; Ijaz, S | 2019 | ASSESSMENT OF GENETIC DIVERSITY BASED ON ISSR MARKERS IN NEOPESTALOTIOPSIS SPECIES COLLECTED FROM GUAVA (PSIDIUM GUAJAVA L.) PLANTS AFFECTED WITH CANKER DISEASE IN PAKISTAN | Guava is susceptible to various phytopathogenic fungi that could be associated with canker diseases. In Pakistan, farmer’s economy is heavily affected by this devastating disease. One of the several strategies to managing it is to develop canker disease resistant guava varieties. For the complete understanding of the associated pathogen and its detailed characterization are required. In this study, Neopestalotiopsis spp accessions of Fungal Molecular Biology Culture Collection UAF (FMB-CC-UAF) isolated from canker affected guava plants were investigated to find genetic diversity among them based on ISSR markers. Eleven (11) ISSR primers were used to analyze eight (8) Neopestalotiopsis accessions. Genetic variation among these fungal accessions were estimated using unweighted pair group method with arithmetic mean (UPGMA) based genetic diversity analysis and Nei’s genetic diversity. Among them FMB-Guv-JF2 and FMB-Guv-JF7 showed 13.01% genetic difference to each other. However, the maximum genetic distance was estimated to be 82.32% between FMB-Guv-B2A and FMB-Guv-B1. Generated dendrogram revealed the grouping of investigated fungal accessions into distinct clusters with considerable genetic diversity. It is also supported by principal coordinate analysis (PCoA) which showed dispersed pattern of these fungal accessions indicating diverse genetic base. | NA | no | gd of pathogen | NA | no | no |
| 5719 | 2020 | 44112 | Ul Haq, I; Ijaz, S; Latif, MZ | 2019 | MULTILOCUS SEQUENCE TYPING (MLST) BASED GENETIC VARIATION ANALYSIS OF SHISHAM DIEBACK ASSOCIATED STRAINS OF CERATOCYSTIS FIMBRIATA SENSU LATO SPECIES COMPLEX IN PAKISTAN | Ceratocystis fimbriata sensu lato species complex is a group of highly diverse cryptic species known to be associated with different diseases in a range of host plants. For the accurate identification of fungal pathogens belonging to this species complex, molecular approaches have been employed in juxtaposition with morphological characterization. Different DNA based methods have become the routine practice of mycologist community throughout the world. Among them, multilocus sequence typing (MLST) approach is gaining popularity. Four fungal taxonomy groups have been established MLST scheme for few fungi, to date. However, in Ceratocystis fimbriata species complex, no report on MLST based genotyping is available yet. Therefore, by considering this perspective, we attempted MLST method for genotyping and diversity analysis of this species complex. We investigated ITS and TEF1-alpha loci, for two multilocus sequence typing scheme. Allelic profile of each locus gave 23 sequence types (STs) and 22 Allele types (ATs). However, the typing efficiency of ITS region was high as compared to TEF1-alpha. The phylogenetic network parallelograms based on neighbor net algorithm also showed genetic diversity in studied isolates. This relationship study was further supported by generating phylogram, which resolved these isolates in three different clades showing unequivocal phylogenetic diversity and relatedness. | NA | no | No good fitness measure and measure host GD | NA | no | no |
| 5720 | 2020 | 44113 | Urazaliev, R; Yessimhekova, M; Mukin, K; Chirkin, A; Ismagulova, G | 2018 | Monitoring of Aegilops L. local species genetic diversity of Kazakhstan’s flora | Cereal Crop Wild Relatives (CWR) are a very important gene pool for cereal/wheat improvement. New genes for resistance to diseases and pests are urgently needed to avoid using pesticides and to raise adaptivity to the environmental stresses caused by global climate change. In this regard, the study is aimed at ex situ conservation of Aegilops L genus local ecotypes’ genetic diversity, which is very relevant and promising for breeding. In order to establish breeding utility and form an ex situ collection reflecting the intra- and inter-specific diversity, the phenotypic screening of Kazakhstan’s local populations of Aegilops L. genus (Ae. cylindrica, Ae. tauschii, Ae. triuncialis and Ae. crassa) was conducted on the basis of multiple indicators. For the first time molecular-genetic analysis of 50 representatives of Aegilops L. genus from Kazakhstan’s flora was performed.The microsatellite analysis with the use of 11 EST-SSR markers revealed eight of them to be most effective. For each marker, allele frequency and average heterozygosity was calculated. For the most informative markers the presence of 5 and 6 respective allelic variations was found. A bank of genomic DNA was created and kept in ex situ storage (-70 degrees C, long-term) in the IMBB of the MES of RK. | NA | no | No good fitness measure | NA | no | no |
| 5721 | 2020 | 44042 | Urbaniak, L; Wojnicka-Poltorak, A; Celinski, K; Lesiczka, P; Pawlaczyk, E; Aucina, A | 2019 | Genetic resources of relict populations of Pinus sylvestris (L.) in Western Carpathians assessed by chloroplast microsatellites | The relict character of the Scots pine populations from the Tatra Mts. (Western Carpathians) was formed on scarcely accessible, ecologically extreme habitats, in areas which were not under human activity. An exhaustive genetic analysis of the Scots pine populations from the refugial locations in the Tatra Mts. (Poland) had not yet been. In this study, we characterize the genetic variation and differentiation of the relict Scots pine populations from the Tatra Mts., to provide information on their genetic resources and the conservation implications. Eight paternally inherited chloroplast microsatellite loci were used to investigate the genetic structure of 4 relict populations, which were compared with the natural populations from the Baltic region (5 populations from Lithuania, Latvia and Estonia) and to a northern range from Russia (5 populations from the Kola Peninsula and Arkhangelsk Oblast). The four relict pine populations were characterized by a lower level of genetic variation when compared to the Baltic and northern populations, which was expressed in lower values of particular genetic parameters: numbers of alleles and haplotypes, haplotype genetic diversity, haplotype differentiation, and mean genetic distance between individuals in the population. Our results revealed a very high and significant genetic differentiation between all the analyzed populations, as well as between the three analyzed regions (PhiPT = 8%). Furthermore, the relict populations within a close geographic location showed higher differentiation (PhiPT = 5%) compared to the northern Russian and Baltic populations that were separated by tens and hundreds of kilometers. The relict populations of P. sylvestris from the Tatra Mts. deserve conservation efforts due to the fact that they are an important component if the species-poor Tatra forests. | NA | no | No good fitness measure | NA | no | no |
| 5722 | 2020 | 44042 | Utsunomiya, YT; Milanesi, M; Fortes, MRS; Porto-Neto, LR; Utsunomiya, ATH; Silva, MVGB; Garcia, JF; Ajmone-Marsan, P | 2019 | Genomic clues of the evolutionary history of Bos indicus cattle | Together with their sister subspecies Bos taurus, zebu cattle (Bos indicus) have contributed to important socioeconomic changes that have shaped modern civilizations. Zebu cattle were domesticated in the Indus Valley 8000 years before present (YBP). From the domestication site, they expanded to Africa, East Asia, southwestern Asia and Europe between 4000 and 1300 YBP, intercrossing with B. taurus to form clinal variations of zebu ancestry across the landmass of Afro-Eurasia. In the past 150 years, zebu cattle reached the Americas and Oceania, where they have contributed to the prosperity of emerging economies. The zebu genome is characterized by two mitochondrial haplogroups (I1 and I2), one Y chromosome haplogroup (Y3) and three major autosomal ancestral groups (Indian-Pakistani, African and Chinese). Phenotypically, zebu animals are recognized by their hump, large ears and excess skin. They are rustic, resilient to parasites and capable of bearing the hot and humid climates of the tropics. Many resources are available to study the zebu genome, including commercial arrays of SNP, reference assemblies and publicly available genotypes and whole-genome sequences. Nevertheless, many of these resources were initially developed to support research and subsidize industrial applications in B. taurus, and therefore they can produce bias in data analysis. The combination of genomics with precision agriculture holds great promise for the identification of genetic variants affecting economically important traits such as tick resistance and heat tolerance, which were naturally selected for millennia and played a major role in the evolution of B. indicus cattle. | NA | no | dometicated host | NA | no | no |
| 5723 | 2020 | 44039 | Uusi-Heikkila, S | 2020 | Implications of size-selective fisheries on sexual selection | Fisheries often combine high mortality with intensive size selectivity and can, thus, be expected to reduce body size and size variability in exploited populations. In many fish species, body size is a sexually selected trait and plays an important role in mate choice and mate competition. Large individuals are often preferred as mates due to the high fecundity and resources they can provide to developing offspring. Large fish are also successful in competition for mates. Fisheries-induced reductions in size and size variability can potentially disrupt mating systems and lower average reproductive success by decreasing opportunities for sexual selection. By reducing population sizes, fisheries can also lead to an increased level of inbreeding. Some fish species avoid reproducing with kin, and a high level of relatedness in a population can further disrupt mating systems. Reduced body size and size variability can force fish to change their mate preferences or reduce their choosiness. If mate preference is genetically determined, the adaptive response to fisheries-induced changes in size and size variability might not occur rapidly. However, much evidence exists for plastic adjustments of mate choice, suggesting that fish might respond flexibly to changes in their social environment. Here, I first discuss how reduced average body size and size variability in exploited populations might affect mate choice and mate competition. I then consider the effects of sex-biased fisheries on mating systems. Finally, I contemplate the possible effects of inbreeding on mate choice and reproductive success and discuss how mate choice might evolve in exploited populations. Currently, little is known about the mating systems of nonmodel species and about the interplay between size-selective fisheries and sexual selection. Future studies should focus on how reduced size and size variability and increased inbreeding affect fish mating systems, how persistent these effects are, and how this might in turn affect population demography. | NA | no | No good fitness measure and measure host GD and inbreeding | NA | no | no |
| 5724 | 2020 | 44112 | Valenca-Barbosa, C; do Bomfim, TCB; Teixeira, BR; Gentile, R; Neto, SFD; Magalhaes, BSN; Balthazar, DD; da Silva, FA; Biot, R; Levy, CMD; Santos, HLC | 2019 | Molecular epidemiology of Blastocystis isolated from animals in the state of Rio de Janeiro, Brazil | The enteric protist Blastocystis is one of the most frequently reported parasites infecting both humans and many other animal hosts worldwide. A remarkable genetic diversity has been observed in the species, with 17 different subtypes (STs) on a molecular phylogeny based on small subunit RNA genes (SSU rDNA). Nonetheless, information regarding its distribution, diversity and zoonotic potential remains still scarce, especially in groups other than primates. In Brazil, only a few surveys limited to human isolates have so far been conducted on Blastocystis STs. The aim of this study is to determine the occurrence of Blastocystis subtypes in non-human vertebrate and invertebrate animal groups in different areas of the state of Rio de Janeiro, Brazil. A total of 334 stool samples were collected from animals representing 28 different genera. Blastocystis cultivated samples were subtyped using nuclear small subunit ribosomal DNA (SSU rDNA) sequencing. Phylogenetic analyses and BLAST searches revealed six subtypes: ST5 (28.8%), ST2 (21.1%), ST1 and ST8 (19.2%), ST3 (7.7%) and ST4 (3.8%). Our findings indicate a considerable overlap between STs in humans and other animals. This highlights the importance of investigating a range of hosts for Blastocystis to understand the eco-epidemiological aspects of the parasite and its host specificity. | NA | no | gd of pathogen | NA | no | no |
| 5725 | 2020 | 44043 | Van De Kerk, M; Onorato, DP; Hostetler, JA; Bolker, BM; Oli, MK | 2019 | Dynamics, Persistence, and Genetic Management of the Endangered Florida Panther PopulationDinamicas, Persistencia y Manejo Genetico de la Poblacion en Peligro de Extincion de Pantera de Florida | Abundant evidence supports the benefits accrued to the Florida panther (Puma concolor coryi) population via the genetic introgression project implemented in South Florida, USA, in 1995. Since then, genetic diversity has improved, the frequency of morphological and biomedical correlates of inbreeding depression have declined, and the population size has increased. Nevertheless, the panther population remains small and isolated and faces substantial challenges due to deterministic and stochastic forces. Our goals were 1) to comprehensively assess the demographics of the Florida panther population using long-term (1981-2015) field data and modeling to gauge the persistence of benefits accrued via genetic introgression and 2) to evaluate the effectiveness of various potential genetic management strategies. Translocation and introduction of female pumas (Puma concolor stanleyana) from Texas, USA, substantially improved genetic diversity. The average individual heterozygosity of canonical (non-introgressed) panthers was 0.386 +/- 0.012 (SE); for admixed panthers, it was 0.615 +/- 0.007. Survival rates were strongly age-dependent (kittens had the lowest survival rates), were positively affected by individual heterozygosity, and decreased with increasing population abundance. Overall annual kitten survival was 0.32 +/- 0.09; sex did not have a clear effect on kitten survival. Annual survival of subadult and adult panthers differed by sex; regardless of age, females exhibited higher survival than males. Annual survival rates of subadult, prime adult, and old adult females were 0.97 +/- 0.02, 0.86 +/- 0.03, and 0.78 +/- 0.09, respectively. Survival rates of subadult, prime adult, and old adult males were 0.66 +/- 0.06, 0.77 +/- 0.05, and 0.65 +/- 0.10, respectively. For panthers of all ages, genetic ancestry strongly affected survival rate, where first filial generation (F1) admixed panthers of all ages exhibited the highest rates and canonical (mostly pre-introgression panthers and their post-introgression descendants) individuals exhibited the lowest rates. The most frequently observed causes of death of radio-collared panthers were intraspecific aggression and vehicle collision. Cause-specific mortality analyses revealed that mortality rates from vehicle collision, intraspecific aggression, other causes, and unknown causes were generally similar for males and females, although males were more likely to die from intraspecific aggression than females. The probability of reproduction and the annual number of kittens produced varied by age; evidence that ancestry or abundance influenced these parameters was weak. Predicted annual probabilities of reproduction were 0.35 +/- 0.08, 0.50 +/- 0.05, and 0.25 +/- 0.06 for subadult, prime adult, and old adult females, respectively. The number of kittens predicted to be produced annually by subadult, prime adult, and old adult females were 2.80 +/- 0.75, 2.67 +/- 0.43, and 2.28 +/- 0.83, respectively. The stochastic annual population growth rate estimated using a matrix population model was 1.04 (95% CI = 0.72-1.41). An individual-based population model predicted that the probability that the population would fall below 10 panthers within 100 years (quasi-extinction) was 1.4% (0-0.8%) if the adverse effects of genetic erosion were ignored. However, when the effect of genetic erosion was considered, the probability of quasi-extinction within 100 years increased to 17% (0-100%). Mean times to quasi-extinction, conditioned on going quasi-extinct within 100 years, was 22 (0-75) years when the effect of genetic erosion was considered. Sensitivity analyses revealed that the probability of quasi-extinction and expected time until quasi-extinction were most sensitive to changes in kitten survival parameters. Without genetic management intervention, the Florida panther population would face a substantially increased risk of quasi-extinction. The question, therefore, is not whether genetic management of the Florida panther population is needed but when and how it should be implemented. Thus, we evaluated genetic and population consequences of alternative genetic introgression strategies to identify optimal management actions using individual-based simulation models. Releasing 5 pumas every 20 years would cost much less ($200,000 over 100 years) than releasing 15 pumas every 10 years ($1,200,000 over 100 years) yet would reduce the risk of quasi-extinction by comparable amount (44-59% vs. 40-58%). Generally, releasing more females per introgression attempt provided little added benefit. The positive effects of the genetic introgression project persist in the panther population after 20 years. We suggest that managers contemplate repeating genetic introgression by releasing 5-10 individuals from other puma populations every 20-40 years. We also recommend that managers continue to collect data that will permit estimation and monitoring of kitten, adult, and subadult survival. We identified these parameters via sensitivity analyses as most critical in terms of their impact on the probability of and expected times to quasi-extinction. The continuation of long-term monitoring should permit the adaptation of genetic management strategies as necessary while collecting data that have proved essential in assessing the genetic and demographic health of the population. The prospects for recovery of the panther will certainly be improved by following these guidelines. (c) 2019 The Authors. Wildlife Monographs published by Wiley Periodicals, Inc. on behalf of The Wildlife Society. | NA | no | no good gd measure host | NA | no | no |
| 5726 | 2020 | 44043 | van den Berg, RR; Dissel, S; Rapallini, MLBA; van Der Weijden, CC; Wit, B; Heymans, R | 2019 | Characterization and whole genome sequencing of closely related multidrug-resistant Salmonella enterica serovar Heidelberg isolates from imported poultry meat in the Netherlands | Multidrug-resistant Salmonella enterica serovar Heidelberg isolates are frequently recovered in the Netherlands from poultry meat imported from South America. Our aim was to retrospectively assess the characteristics of the antimicrobial determinants, gene content and the clonal relatedness of 122 unique S. Heidelberg isolates from chicken meat from Brazil (n = 119) and Argentina (n = 3) that were imported between 2010 and 2015. These isolates were subjected to antimicrobial susceptibility testing, PCR and Illumina HiSeq2500 whole genome sequencing. Draft genomes were assembled to assess the gene content, and the phylogenetic relationships between isolates were determined using single nucleotide polymorphisms. Ciprofloxacin-resistance was identified in 98.4% of the isolates and 83.7% isolates showed resistance to the extended-spectrum cephalosporins cefotaxime and ceftazidime (83.6% and 82.8% respectively). Of the latter, 97.1% exhibited an AmpC phenotype and contained bla(CMY-2), whereas the remaining three isolates contained an extended spectrum beta-lactamase. Of the 99 extended-spectrum cephalosporins-resistant isolates harboring CMY-2 plasmids, 56.6% contained the incompatibility group I1 replicon. Phylogenetic cluster analysis showed that all isolates from Brazil clustered together, with 49% occurring in clusters larger than 5 isolates that revealed intra-cluster similarities based on geographical location and/or resistance profiles. The remaining isolates were classified in smaller clusters or as singletons, highlighting the large diversity of S. Heidelberg in the poultry chain in Brazil that was revealed by this study. Considering the potential public health risk associated with multidrug-resistant S. Heidelberg in imported poultry, collaborative whole genome sequencing-based surveillance is needed to monitor the spread, pathogenic properties and epidemiological distribution of these isolates. | NA | no | No good fitness measure and measure host GD | NA | no | no |
| 5727 | 2020 | 44113 | van den Burg, MP; Meirmans, PG; van Wagensveld, TP; Kluskens, B; Madden, H; Welch, ME; Breeuwer, JAJ | 2018 | The Lesser Antillean Iguana (Iguana delicatissima) on St. Eustatius: Genetically Depauperate and Threatened by Ongoing Hybridization | The Lesser Antillean Iguana (Iguana delicatissima) is an endangered species threatened by habitat loss and hybridization with non-native Green Iguanas li iguana). Iguana delicatissima has been extirpated on several islands, and the Green Iguana has invaded most islands with extant populations. Information is essential to protect this species from extinction. We collected data on 293 iguanas including 17 juveniles from St. Eustasius, one of the few remaining I. delicatissima strongholds. Genetic data were leveraged to test for hybridization presence with the Green Iguana using both mitochondrial and nuclear genes, including 16 microsatellite loci. The microsatellites were also analyzed to estimate genetic diversity, population structure, and effective population size. Using molecular and morphological data, we identified 286 I. delicatissima individuals captured during our first fieldwork effort, and 7 non-native iguanas captured during a second effort, showing hybridization occurs within this population. Comparing homologous microsatellites used in studies on Dominica and Chancel, the I. delicatissima population on St. Eustatius has extremely low genetic diversity (H-O = 0(.)051; H-E = 0.057), suggesting this population is genetically depauperate. Furthermore, there is significant evidence for inbreeding (F-IS= 0.12) and weak spatial genetic structure (F-ST = 0.021, P= 0.002) within this population. Besides immediate threats including hybridization, this population’s lo’ genetic diversity, presence of physiological abnormalities and low recruitment could indicate presence of inbreeding depression that threatens its long-term survival. We conclude there is a continued region-wide threat to I. delicatissima and highlight the need for immediate conservation action to stop the continuing spread of Green Iguanas and to eliminate hybridization from St. Eustatius. | NA | no | No good fitness measure and measure host GD | NA | no | no |
| 5728 | 2020 | 44112 | van der Valk, T; Diez-del-Molino, D; Marques-Bonet, T; Guschanski, K; Dalen, L | 2019 | Historical Genomes Reveal the Genomic Consequences of Recent Population Decline in Eastern Gorillas | Many endangered species have experienced severe population declines within the last centuries [1, 2]. However, despite concerns about negative fitness effects resulting from increased genetic drift and inbreeding, there is a lack of empirical data on genomic changes in conjunction with such declines [3-7]. Here, we use whole genomes recovered from century-old historical museum specimens to quantify the genomic consequences of small population size in the critically endangered Grauer’s and endangered mountain gorillas. We find a reduction of genetic diversity and increase in inbreeding and genetic load in the Grauer’s gorilla, which experienced severe population declines in recent decades. In contrast, the small but relatively stable mountain gorilla population has experienced little genomic change during the last century. These results suggest that species histories as well as the rate of demographic change may influence how population declines affect genome diversity. | NA | no | No good fitness measure and measure host GD | NA | no | no |
| 5729 | 2020 | 44040 | van der Valk, T; Gonda, CM; Silegowa, H; Almanza, S; Sifuentes-Romero, I; Hart, TB; Hart, JA; Detwiler, KM; Guschanski, K | 2020 | The Genome of the Endangered Dryas Monkey Provides New Insights into the Evolutionary History of the Vervets | Genomic data can be a powerful tool for inferring ecology, behavior, and conservation needs of highly elusive species, particularly, when other sources of information are hard to come by. Here, we focus on the Dryas monkey (Cercopithecus dryas), an endangered primate endemic to the Congo Basin with cryptic behavior and possibly <250 remaining adult individuals. Using whole-genome sequencing data, we show that the Dryas monkey represents a sister lineage to the vervets (Chlorocebus sp.) and has diverged from them similar to 1.4 Ma with additional bidirectional gene flow similar to 750,000-similar to 500,000years ago that has likely involved the crossing of the Congo River. Together with evidence of gene flow across the Congo River in bonobos and okapis, our results suggest that the fluvial topology of the Congo River might have been more dynamic than previously recognized. Despite the presence of several homozygous loss-of-function mutations in genes associated with sperm mobility and immunity, we find high genetic diversity and low levels of inbreeding and genetic load in the studied Dryas monkey individual. This suggests that the current population carries sufficient genetic variability for long-term survival and might be larger than currently recognized. We thus provide an example of how genomic data can directly improve our understanding of highly elusive species. | NA | no | No good fitness measure and measure host GD | NA | no | no |
| 5730 | 2020 | 44113 | van der Valk, T; Sandoval-Castellanos, E; Caillaud, D; Ngobobo, U; Binyinyi, E; Nishuli, R; Stoinski, T; Gilissen, E; Sonet, G; Semal, P; Kalthoff, DC; Dalen, L; Guschanski, K | 2018 | Significant loss of mitochondrial diversity within the last century due to extinction of peripheral populations in eastern gorillas | Species and populations are disappearing at an alarming rate as a direct result of human activities. Loss of genetic diversity associated with population decline directly impacts species’ long-term survival. Therefore, preserving genetic diversity is of considerable conservation importance. However, to assist in conservation efforts, it is important to understand how genetic diversity is spatially distributed and how it changes due to anthropogenic pressures. In this study, we use historical museum and modern faecal samples of two critically endangered eastern gorilla taxa, Grauer’s (Gorilla beringei graueri) and mountain gorillas (Gorilla beringei beringei), to directly infer temporal changes in genetic diversity within the last century. Using over 100 complete mitochondrial genomes, we observe a significant decline in haplotype and nucleotide diversity in Grauer’s gorillas. By including historical samples from now extinct populations we show that this decline can be attributed to the loss of peripheral populations rather than a decrease in genetic diversity within the core range of the species. By directly quantifying genetic changes in the recent past, our study shows that human activities have severely impacted eastern gorilla genetic diversity within only four to five generations. This rapid loss calls for dedicated conservation actions, which should include preservation of the remaining peripheral populations. | NA | no | No good fitness measure | NA | no | no |
| 5731 | 2020 | 44112 | van Elst, T; Gadau, J | 2018 | Temporal variation in social structure and worker reproduction in the temporary social parasite Lasius fuliginosus (Hymenoptera: Formicidae) | Ant societies exhibit striking diversity in their social systems, including variation in the number of queens and mating partners. Knowledge on the number of breeders in a colony is crucial for a better understanding of the evolution of social insect life history traits such as reproductive skew or worker reproduction. Little is known about the breeding system of the formicine ant Lasius fuliginosus (LATREILLE, 1798), even though it is widely distributed in the Palearctic and able to compete ecologically with dominant genera like Formica. Moreover, L. fuliginosus has a particularly interesting life history in that it is a temporary social parasite of several Lasius species, which themselves are temporary social parasites. We determined the number of (reproductive) queens and mating partners of L. fuliginosus colonies and queens, respectively, from a population in Minster, Germany. Workers from 33 colonies and males from 12 of these colonies were genotyped for four polymorphic microsatellite markers. Our results show that 29 of these colonies were monogynous and monandrous and that two colonies were monogynous and polyandrous. Workers of the remaining two colonies were derived from multiple queens, possibly due to adoption of unrelated queens after the original queen’s death. Furthermore, genotyping of male offspring provided evidence for worker reproduction in three colonies, potentially also in response to queen orphanage in two of these. We estimated the mutation rate at one microsatellite locus in L. fuliginosus to be 1.46 x 10(-3) mutations per generation, which is similar to what has been observed in Apis mellifera LINNAEUS, 1758 and Drosophila melanogaster MEIGEN, 1830. To our knowledge, this is the first study to provide molecular insights into the breeding system of L. fuliginosus, which appears to be characterized by facultative polyandry and monogyny. In addition, L. fuliginosus now represents the second species in the genus Lasius for which worker reproduction has been documented. | NA | no | No good fitness measure | NA | no | no |
| 5732 | 2020 | 44113 | van Hooft, P; Keet, DF; Brebner, DK; Bastos, ADS | 2018 | Genetic insights into dispersal distance and disperser fitness of African lions (Panthera leo) from the latitudinal extremes of the Kruger National Park, South Africa | Background: Female lions generally do not disperse far beyond their natal range, while males can disperse distances of over 200 km. However, in bush-like ecosystems dispersal distances less than 25 km are reported. Here, we investigate dispersal in lions sampled from the northern and southern extremes of Kruger National Park, a bush-like ecosystem in South Africa where bovine tuberculosis prevalence ranges from low to high across a north-south gradient. Results: A total of 109 individuals sampled from 1998 to 2004 were typed using 11 microsatellite markers, and mitochondrial RS-3 gene sequences were generated for 28 of these individuals. Considerable north-south genetic differentiation was observed in both datasets. Dispersal was male-biased and generally further than 25 km, with long-distance male gene flow (75-200 km, detected for two individuals) confirming that male lions can travel large distances, even in bush-like ecosystems. In contrast, females generally did not disperse further than 20 km, with two distinctive RS-3 gene clusters for northern and southern females indicating no or rare long-distance female dispersal. However, dispersal rate for the predominantly non-territorial females from southern Kruger (fraction dispersers >= 0.68) was higher than previously reported. Of relevance was the below-average body condition of dispersers and their low presence in prides, suggesting low fitness. Conclusions: Large genetic differences between the two sampling localities, and low relatedness among males and high dispersal rates among females in the south, suggestive of unstable territory structure and high pride turnover, have potential implications for spread of diseases and the management of the Kruger lion population. | NA | no | No good fitness measure and measure host GD | NA | no | no |
| 5733 | 2020 | 44113 | van Schaik, J; Dekeukeleire, D; Gazaryan, S; Natradze, I; Kerth, G | 2018 | Comparative phylogeography of a vulnerable bat and its ectoparasite reveals dispersal of a non-mobile parasite among distinct evolutionarily significant units of the host | Knowledge about phylogeographical structuring and genetic diversity is of key importance for the conservation of endangered species. Comparative phylogeography of a host and its parasite has the potential to reveal cryptic dispersal and behaviour in both species, and can thus be used to guide conservation management. In this study, we investigate the phylogeographic structure of the Bechstein’s bat, Myotis bechsteinii, and its ectoparasitic bat fly, Basilia nana, at 12 sites across their entire distribution. For both species, a mitochondrial sequence fragment (ND1 and COI respectively) and nuclear microsatellite genotypes (14 and 10 loci respectively) were generated and used to compare the phylogeography of host and parasite. Our findings confirm the presence of three distinct genetic subpopulations of the Bechstein’s bat in (1) Europe, (2) the Caucasus and (3) Iran, which remain isolated from one another. The genetic distinctiveness of host populations in the Caucasus region and Iran emphasize that these populations must be managed as distinct evolutionarily significant units. This phylogeographical pattern is however not reflected in its parasite, B. nana, which shows evidence for more recent dispersal between host subpopulations. The discordant genetic pattern between host and parasite suggest that despite the long-term genetic isolation of the different host subpopulations, long-range dispersal of the parasite has occurred more recently, either as the result of secondary contact in the primary host or via secondary host species. This indicates that a novel pathogenic threat to one host subpopulation may be able to disperse, and thus have important consequences for all subpopulations. | NA | no | No good fitness measure | NA | no | no |
| 5734 | 2020 | 44113 | van Treuren, R; Hoekstra, R; van Hintum, TJL | 2017 | Inventory and prioritization for the conservation of crop wild relatives in The Netherlands under climate change | Crop-related wild plant species are a rich source of genetic diversity and are potentially useful in plant breeding for the development of varieties with novel traits. However, many crop wild relatives are poorly represented in gene banks, while their continued survival in situ is by no means ensured. Here we introduced a methodology to inventory relevant taxa and to assess their threat levels for continued survival in situ, including the expected effects of climate change, and applied it to crop wild relatives in The Netherlands. A total number of 214 taxa of wild relatives of economically important agricultural and horticultural crops were identified, of which 53 are included in the Dutch red list of plant species. The group of 53 red list species was studied in more detail to prioritize species for conservation. Based on recent distribution data, the number of Dutch populations consisting of at least 50 individuals varied strongly among the red list species. The majority of these ‘large’ populations were found to be located in protected areas. Furthermore, niche modelling was used to study the expected effects of climate change on the future distribution of the red list species. These analyses predicted a reduced distribution area for the majority of species, although also positive effects of climate change were observed for several species. Similar patterns of change were observed when only protected areas were considered. Results of the study were used to prioritize the conservation of crop wild relatives in The Netherlands. | NA | no | No good fitness measure and measure host GD | NA | no | no |
| 5735 | 2020 | 44112 | Van Wyk, AM; Kotze, A; Grobler, JP; Van Vuuren, BJ; Barrow, LN; Dalton, DL | 2018 | Isolation and characterization of species-specific microsatellite markers for blue and black wildebeest (Connochaetes taurinus and C-gnou) | The blue wildebeest (Connochaetes taurinus) is distributed throughout southern and east Africa while the black wildebeest (Connochaetes gnou) is endemic to South Africa and was driven to near extinction in the early 1900s due to hunting pressure and disease outbreaks. Extensive translocation of both species throughout South Africa is threatening the genetic integrity of blue and black wildebeest. To effectively manage these species, genetic tools that can be used to detect hybrid individuals, identify genetically unique subpopulations and determine the levels of genetic diversity are required. In this study, 11 microsatellite markers were developed for wildebeest through next-generation sequencing. The microsatellite loci displayed 2.00-4.14 alleles, unbiased heterozygosity values ranged from 0.32 to 0.60 and observed heterozygosity values ranged from 0.26 to 0.52. The comparatively high level of polymorphism observed in the microsatellite markers indicates that these markers can contribute significantly to our knowledge of population genetic structure, relatedness, genetic diversity and hybridization in these species. | NA | no | No good fitness measure | NA | no | no |
| 5736 | 2020 | 44112 | Vanden Broeck, A; Cox, K; Melosik, I; Maes, B; Smets, K | 2018 | Genetic diversity loss and homogenization in urban trees: the case of Tiliaxeuropaea in Belgium and the Netherlands | Urban trees form a vital component of sustainable cities but the use of a restricted range of species and genotypes may pose a risk to global biodiversity. Despite several studies investigating tree species diversity, intraspecific genetic diversity of urban trees remains largely unexplored. Here, we characterized the genetic diversity of Tiliaxeuropaea, one of the most widely planted urban tree species in Northwest Europe. We compared the genotypic diversity of historical plantings of Tilia spp. from the 17th century with the genotypic diversity of currently available planting stock in Belgium and the Netherlands. In total, 129 trees were sampled and genotyped with 14 microsatellite loci and 150 polymorphic Amplified Fragment Length Polymorphism markers. In Northwest Europe, homogenization of urban T.xeuropaea plantings already started at the 17th century. Genetic diversity within contemporary commercial planting stocks was extremely narrow and consisted mainly of two clones, sold under the name Pallida’ and Zwarte linde’. The genetic diversity found within the historical plantings was about four times higher than in the current commercial planting stocks. We recommend that tree nurseries should enlarge the genetic diversity of T.xeuropaea commercial planting stocks. The old clones have shown long-term disease resistance and could provide tree breeders with the valuable new genetic material. The range of available Tilia species and genotypes needs to be explored in future urban tree planning to optimize desired ecosystem services. | NA | no | No good fitness measure | NA | no | no |
| 5737 | 2020 | 44113 | Vander Haegen, WM; Orth, GR; Johnston, AN; Linders, MJ | 2018 | Endemic Diseases Affect Population Dynamics of Tree Squirrels in Contrasting Landscapes | Habitat loss and fragmentation can have detrimental effects on wildlife populations and where pervasive can create population isolates that may experience reduced genetic diversity and lower persistence. Diseases that cause epizootics also can reduce wildlife populations and may have disproportionate effects on small populations. We studied survival of radio-marked western gray squirrels (Sciurus griseus) using known-fate models in Program MARK and we quantified annual reproductive success by following females through the breeding season and counting young at natal nests. We used data on survival and productivity to model population growth rate and associated parameters using deterministic and stochastic approaches. Populations of western gray squirrels that we studied in an extensive, forested landscape and in a highly fragmented, urbanizing landscape in Washington, USA differed in their modeled growth rate. Adult survival was similar between populations although both were strongly affected by different endemic diseases with high epizootic potential. The demographic parameters that differed most between these 2 populations were related to productivity; litter size was marginally smaller and reproductive success was significantly lower in the urbanizing Puget Trough compared to rural Klickitat County. Results of our demographic modeling suggest that the larger Klickitat population is robust to immediate threats, whereas the smaller Puget Trough population is at risk because of its small size and low fecundity. Periodic outbreaks of notoedric mange in the Klickitat population reduce adult survival, although our models suggest that these epizootics would need to occur more frequently than observed to be of significant risk to the population. Continued degradation and fragmentation of western gray squirrel habitat in the Klickitat region along with mild winters resulting from climate change could increase the frequency and severity of mange epizootics and further threaten this population. Actions to retain and improve habitat resources may help ameliorate the effects of future mange epizootics and maintaining quality habitat should be a management priority in this region. The insular Puget Trough population experienced mortality due to tularemia each year of our study with infection rates 14%. Lack of characteristic histological indications in some affected squirrels may lead to underreporting of tularemia in animals submitted for routine necropsy and could complicate assessment of mortality risks in wildlife population studies. Given its small size and isolation, the Puget Trough population should be monitored closely for indications of decline in number or occupancy; this small population may need periodic augmentation to maintain genetic diversity. Increasing suitable habitat and maintaining connectivity between currently occupied range and potential habitat in the surrounding landscape will be crucial to the long-term viability of this population but will be challenging in the urbanizing landscape of the Puget Trough. (C) 2017 The Wildlife Society. | NA | no | no good gd measure host pop | NA | no | no |
| 5738 | 2020 | 44111 | Vanek, JP; Glowacki, GA | 2019 | Assessing the Impacts of Urbanization on Sex Ratios of Painted Turtles (Chrysemys picta) | Turtles are particularly susceptible to the negative impacts of urbanization due to low mobility and a life history strategy emphasizing long generation times and high adult survival. In addition to declines directly through habitat loss, urbanization has been hypothesized to limit populations of aquatic turtles through changes in population structure, as adult females are disproportionally killed on and near roads, leading to male-biased populations, which can lead to population declines or local extirpations. The purpose of this study was to better understand how urbanization impacts the sex ratios of painted turtles (Chrysemys picta) in an urban ecosystem, as empirical results linking male-biased turtle populations to roads and urbanization are mixed. Using eight years of trapping data from a long-term monitoring program in a suburb of Chicago, IL, USA, we report one of the most male-biased populations of turtles in the USA, consistent with prevailing road mortality hypotheses. However, we found no evidence that male-biased populations were related to road density or the amount of protected area around a sampling location and found that impervious surface (a metric of urbanization) was weakly related to less male-biased populations. Our results highlight the importance of replicating ecological studies across space and time and the difficulty in assessing population structure in aquatic turtles. We suggest that active conservation measures may be warranted for the continued persistence of urban turtle populations. | NA | no | No good fitness measure and measure host GD | NA | no | no |
| 5739 | 2020 | 44040 | Vasilyeva, YS; Zhulanov, AA; Boronnikova, SV; Yanbaev, YA | 2020 | Genetic structure of ural populations of Larix sibirica Ledeb. on the base of analysis of nucleotide polymorphism | Research on the state and dynamics of the gene pool (an important natural resource that determines the potential fitness of living organisms and, ultimately, their long-term survival) becomes an important problem in the context of increased anthropogenic environmental impact. They are especially important for key species of ecosystems of a global scale importance. Larix sibirica Ledeb., which spreads from the Western Siberia to the Russian North-West, is one of such forest tree species. We identified patterns of genetic structure of populations on the example of the species’ Western race on the Middle and Northern Urals. The analysis of nucleotide polymorphism of genes of ABA-inducible protein, MADS-box-transcription factor and of 4-kumarat: CoA ligase (a part of the gene) was used as a method. Evidences were obtained that a part of the populations previously formed a single large population. At the same time, populations with different gene pools were found. As a result, differences between populations within the region were more pronounced (fixation index F-ST = -0.021 - 0.260, total haplotype diversity Hd = 0.636 - 0.911; nucleotide diversity pi = 0.005 - 0.009; number of mutations theta(w) = 0.005 - 0.012) than in other parts of the race. Causes of this phenomenon are discussed. It was concluded that the larch forests with a unique gene pool and/or high genetic diversity should be objects of population-oriented forestry and conservation. | NA | no | No good fitness measure | NA | no | no |
| 5740 | 2020 | 44112 | Veeravechsukij, N; Krailas, D; Namchote, S; Wiggering, B; Neiber, MT; Glaubrecht, M | 2018 | Molecular phylogeography and reproductive biology of the freshwater snail Tarebia granifera in Thailand and Timor (Cerithioidea, Thiaridae): morphological disparity versus genetic diversity | The freshwater thiarid gastropod Tarebia granifera (Lamarck, 1816), including taxa considered either congeneric or conspecific by earlier authors, is widespread and abundant in various lentic and lotic water bodies in mainland and insular Southeast Asia, with its range extending onto islands in the Indo-West-Pacific. This snail is, as one of the most frequent and major first intermediate host, an important vector for digenic trematodes causing several human diseases. As a typical thiarid T. granifera is viviparous and parthenogenetic, with various embryonic stages up to larger shelled juveniles developing within the female’s subhemocoelic (i.e non-uterine) brood pouch. Despite the known conchological disparity in other thiarids as well as this taxon, in Thailand Tarebia has been reported with the occurrence of one species only. In light of the polytypic variations found in shell morphology of freshwater snails in general and this taxon in particular, the lack of a modern taxonomic-systematic revision, using molecular genetics, has hampered more detailed insights to date, for example, into the locally varying trematode infection rates found in populations of Tarebia from across its range in Thailand as well as neighboring countries and areas. Here, we integrate evidence from phylogeographical analyses based on phenotypic variation (shell morphology, using biometry and geometric morphometrics) with highly informative and heterogeneous mtDNA sequence data (from the gene fragments cytochrome c oxidase subunit 1 and 16 S rRNA). We evaluate both the morphological and molecular genetic variation (using several phylogenetic analyses, including haplotype networks and a dated molecular tree), in correlation with differences in the reproductive biology among populations of Tarebia from various water bodies in the north, northwest, central, and south of Thailand, supplementing our respective analyses of parasite infections of this thiarid by cercaria of 15 trematode species, reported in a parallel study. Based on the comparison of topotypical material from the island of Timor, with specimens from 12 locations as reference, we found significant, albeit not congruent variation of both phenotype and genotype in Tarebia granifera, based on 1,154 specimens from 95 Thai samples, representing a geographically wide-ranging, river-based cross-section of this country. Our analyses indicate the existence of two genetically distinct clades and hint at possible species differentiation within what has been traditionally considered as T. granifera. These two lineages started to split about 5 mya, possibly related to marine transgressions forming what became known as biogeographical barrier north of the Isthmus of Kra. Grounded on the site-by-site analysis of individual Tarebia populations, our country-wide chorological approach focussing on the conchologically distinct and genetically diverse lineages of Tarebia allows to discuss questions of this either reflecting subspecific forms versus being distinct species within a narrowly delimited species complex. Our results, therefore, provide the ground for new perspectives on the phylogeography, evolution and parasitology of Thai freshwater gastropods, exemplified here by these highly important thiarids. | NA | no | No good fitness measure | NA | no | no |
| 5741 | 2020 | 44111 | Veltjen, E; Asselman, P; Rodriguez, MH; Bejerano, AP; Lozano, ET; Torres, LRG; Goetghebeur, P; Larridon, I; Samain, MS | 2019 | Genetic patterns in Neotropical Magnolias (Magnoliaceae) using de novo developed microsatellite markers | Conserving tree populations safeguards forests since they represent key elements of the ecosystem. The genetic characteristics underlying the evolutionary success of the tree growth form: high genetic diversity, extensive gene flow and strong species integrity, contribute to their survival in terms of adaptability. However, different biological and landscape contexts challenge these characteristics. This study employs 63 de novo developed microsatellite or SSR (Single Sequence Repeat) markers in different datasets of nine Neotropical Magnolia species. The genetic patterns of these protogynous, insect-pollinated tree species occurring in fragmented, highly-disturbed landscapes were investigated. Datasets containing a total of 340 individuals were tested for their genetic structure and degree of inbreeding. Analyses for genetic structure depicted structuring between species, i.e. strong species integrity. Within the species, all but one population pair were considered moderate to highly differentiated, i.e. no indication of extensive gene flow between populations. No overall correlation was observed between genetic and geographic distance of the pairwise species’ populations. In contrast to the pronounced genetic structure, there was no evidence of inbreeding within the populations, suggesting mechanisms favouring cross pollination and/or selection for more genetically diverse, heterozygous offspring. In conclusion, the data illustrate that the Neotropical Magnolias in the context of a fragmented landscape still have ample gene flow within populations, yet little gene flow between populations. | NA | no | No good fitness measure and measure host GD | NA | no | no |
| 5742 | 2020 | 44040 | Vera, M; Mora, G; Rodriguez-Guitian, MA; Blanco, A; Casanova, A; Real, C; Romero, R; Bouza, C | 2020 | Living at the edge: population differentiation in endangered Arnica montana from NW Iberian Peninsula | Arnica montana (Asteraceae) is a herbaceous species with high interest for pharmaceutical industry due its anti-inflammatory properties. However, commercial collection in wild, habitat loss, abandonment of land, reforestation and the ongoing climate change threaten the conservation of the species throughout its natural distribution. Galicia (north-western Spain) is one of the main flower suppliers, but no legislation on A. montana exploitation is currently applied. In order to assess the genetic status and population structure in this region, 27 locations were genotyped for 14 microsatellite loci and sequenced for 2 plastid DNA (cpDNA) markers. The relationship of environmental, biochemical and morphological variables with genetic differentiation was also evaluated. Two different cpDNA groups previously described were found. One of them was widely distributed while the other was restricted to heathlands of the O Courel Mountains. The percentage of clonality was low (< 10%). Genetic diversity based on microsatellites (He = 0.245) was lower than in Central European populations, with high levels of population differentiation (F-ST = 0.441), suggesting strong effects of genetic drift. Bayesian clustering methods revealed the presence of four genetically distinct population units, one of them corresponding to the O Courel Mountains group described by cpDNA analysis. Environmental and biochemical factors were related to genetic differentiation, suggesting local adaptation. Necessary conservation measures include the overdue implementation of the Habitats Directive by the Galician Regional Administration, establishing sustainable harvesting regimes, and aim to conserve genetic variation in these endangered populations at the southern range edge. | NA | no | No good fitness measure | NA | no | no |
| 5743 | 2020 | 44113 | Verma, K; Sharma, S; Sharma, A; Dalal, J; Bhardwaj, T | 2018 | Data on haplotype diversity in the hypervariable region I, II and III of mtDNA amongst the Brahmin population of Haryana | Human mitochondrial DNA (mtDNA) is routinely analysed for pathogenic mutations, evolutionary studies, estimation of time of divergence within or between species, phylogenetic studies and identification of degraded remains. The data on various regions of human mtDNA has added enormously to the knowledge pool of population genetics as well as forensic genetics. The displacement-loop (D-loop) in the control region of mtDNA is rated as the most rapidly evolving part, due to the presence of variations in this region. The control region consists of three hypervariable regions. These hypervariable regions (HVI, HVII and HVIII) tend to mutate 5-10 times faster than nuclear DNA. The high mutation rate of these hypervariable regions is used in population genetic studies and human identity testing. In the present data, potentially informative hypervariable regions of mitochondrial DNA (mtDNA) i.e. HVI (np 16024-16365), HVII (np 73-340) and HVIII (np 438-576) were estimated to understand the genetic diversity amongst Brahmin population of Haryana. Blood samples had been collected from maternally unrelated individuals from the different districts of Haryana. An array of parameters comprising of polymorphic sites, transitions, transversions, deletions, gene diversity, nucleotide diversity, pairwise differences, Tajima’s D test, Fu’s Fs test, mismatch observed variance and expected heterozygosity were estimated. The observed polymorphisms with their respective haplogroups in comparison to rCRS were assigned. (C) 2018 The Authors. Published by Elsevier Inc. | NA | no | human | NA | no | no |
| 5744 | 2020 | 44113 | Versteeg, B; Bruisten, SM; Pannekoek, Y; Jolley, KA; Maiden, MCJ; van der Ende, A; Harrison, OB | 2018 | Genomic analyses of the Chlamydia trachomatis core genome show an association between chromosomal genome, plasmid type and disease | Background: Chlamydia trachomatis (Ct) plasmid has been shown to encode genes essential for infection. We evaluated the population structure of Ct using whole-genome sequence data (WGS). In particular, the relationship between the Ct genome, plasmid and disease was investigated. Results: WGS data from 157 Ct isolates deposited in the Chlamydiales pubMLST database (http:// pubMLST.org/chlamydiales/) were annotated with 902 genes including the core and accessory genome. Plasmid associated genes were annotated and a plasmid MLST scheme was defined allowing plasmid sequence types to be determined. Plasmid allelic variation was investigated. Phylogenetic relationships were examined using the Genome Comparator tool available in pubMLST. Phylogenetic analyses identified four distinct Ct core genome clusters and six plasmid clusters, with a strong association between the chromosomal genotype and plasmid. This in turn was linked to ompA genovars and disease phenotype. Horizontal genetic transfer of plasmids was observed for three urogenital-associated isolates, which possessed plasmids more commonly found in isolates resulting from ocular infections. The pgp3 gene was identified as the most polymorphic plasmid gene and pgp4 was the most conserved. Conclusion: A strong association between chromosomal genome, plasmid type and disease was observed, consistent with previous studies. This suggests co-evolution of the Ct chromosome and their plasmids, but we confirmed that plasmid transfer can occur between isolates. These data provide a better understanding of the genetic diversity occurring across the Ct genome in association with the plasmid content. | NA | no | No good fitness measure and measure host GD | NA | no | no |
| 5745 | 2020 | 44042 | Vicentini, C; Mafficini, A; Simbolo, M; Fassan, M; Delfino, P; Lawlor, RT; Rusev, B; Scarpa, A; Corbo, V | 2019 | Comparative Lesions Analysis Through a Targeted Sequencing Approach | Assessing intra-tumoral heterogeneity (ITH) is of paramount importance to anticipate failure of targeted therapies and design accordingly effective anti-tumor strategies. Although concerns are frequently raised due to differences in sample processing and depth of coverage, next-generation sequencing of solid tumors have unraveled a highly variable degree of ITH across tumor types. Capturing the genetic relatedness between primary and metastatic lesions through the identification of clonal and subclonal populations is critical to the design of therapies for advance-stage diseases. Here, we report a method for comparative lesions analysis that allows for the identification of clonal and subclonal populations among different specimens from the same patient. The experimental approach described here integrates three well-established approaches: histological analysis, high-coverage multi-lesion sequencing, and immunophenotypic analyses. In order to minimize the effects on the detection of subclonal events by inappropriate sample processing, we subjected tissues to careful pathological examination and neoplastic cell enrichment. Quality controlled DNA from neoplastic lesions and normal tissues was then subjected to high coverage sequencing, targeting the coding regions of 409 relevant cancer genes. While only looking at a limited genomic space, our approach enables evaluating the extent of heterogeneity among somatic alterations (single-nucleotide mutations and copy-number variations) in distinct lesions from a given patient. Through comparative analysis of sequencing data, we were able to distinguish clonal vs. subclonal alterations. The majority of ITH is often ascribed to passenger mutations; therefore, we also used immunohistochemistry to predict functional consequences of mutations. While this protocol has been applied to a specific tumor type, we anticipate that the methodology described here is broadly applicable to other solid tumor types. | NA | no | No good fitness measure and measure host GD | NA | no | no |
| 5746 | 2020 | 44111 | Vignal, A; Boitard, S; Thebault, N; Dayo, GK; Yapi-Gnaore, V; Karim, IYA; Berthouly-Salazar, C; Palinkas-Bodzsar, N; Guemene, D; Thibaud-Nissen, F; Warren, WC; Tixier-Boichard, M; Rognon, X | 2019 | A guinea fowl genome assembly provides new evidence on evolution following domestication and selection in galliformes | The helmeted guinea fowl Numida meleagris belongs to the order Galliformes. Its natural range includes a large part of sub-Saharan Africa, from Senegal to Eritrea and from Chad to South Africa. Archaeozoological and artistic evidence suggest domestication of this species may have occurred about 2,000 years BP in Mali and Sudan primarily as a food resource, although villagers also benefit from its capacity to give loud alarm calls in case of danger, of its ability to consume parasites such as ticks and to hunt snakes, thus suggesting its domestication may have resulted from a commensal association process. Today, it is still farmed in Africa, mainly as a traditional village poultry, and is also bred more intensively in other countries, mainly France and Italy. The lack of available molecular genetic markers has limited the genetic studies conducted to date on guinea fowl. We present here a first-generation whole-genome sequence draft assembly used as a reference for a study by a Pool-seq approach of wild and domestic populations from Europe and Africa. We show that the domestic populations share a higher genetic similarity between each other than they do to wild populations living in the same geographical area. Several genomic regions showing selection signatures putatively related to domestication or importation to Europe were detected, containing candidate genes, most notably EDNRB2, possibly explaining losses in plumage coloration phenotypes in domesticated populations. | NA | no | No good fitness measure | NA | no | no |
| 5747 | 2020 | 44112 | Villabona-Arenas, CJ; Ayouba, A; Esteban, A; D’arc, M; Ngole, EM; Peeters, M | 2018 | Noninvasive western lowland gorilla’s health monitoring: A decade of simian immunodeficiency virus surveillance in southern Cameroon | Simian immunodeficiency virus (SIVgor) causes persistent infection in critically endangered western lowland gorillas (Gorilla gorilla gorilla) from west central Africa. SIVgor is closely related to chimpanzee and human immunodeficiency viruses (SIVcpz and HIV-1, respectively). We established a noninvasive method that does not interfere with gorillas’ natural behaviour to provide wildlife pathogen surveillance and health monitoring for conservation. A total of 1,665 geo-referenced fecal samples were collected at regular intervals from February 2006 to December 2014 (123 sampling days) in the Campo-Ma’an National Park (southwest Cameroon). Host genotyping was performed using microsatellite markers, SIVgor infection was identified by serology and genetic amplification was attempted on seropositive individuals. We identified at least 125 distinct gorillas, 50 were resampled (observed 3.5 times in average) and 38 were SIVgor+ (seven individuals were seroconverters). Six groups of gorillas were identified based on the overlapping occurrence of individuals with apparent high rates of gene flow. We obtained SIVgor genetic sequences from 25 of 38 seropositive genotyped gorillas and showed that the virus follows exponential growth dynamics under a strict molecular clock. Different groups shared SIVgor lineages demonstrating intergroup viral spread and recapture of positive individuals illustrated intra-host viral evolution. Relatedness and relationship genetic analysis of gorillas together with Bayesian phylogenetic inference of SIVgor provided evidence suggestive of vertical transmission. In conclusion, we provided insights into gorilla social dynamics and SIVgor evolution and emphasized the utility of noninvasive sampling to study wildlife health populations. These findings contribute to prospective planning for better monitoring and conservation. | NA | no | no good gd measure host pop | NA | no | no |
| 5748 | 2020 | 44111 | Villalba-Mouco, V; van de Loosdrecht, MS; Posth, C; Mora, R; Martinez-Moreno, J; Rojo-Guerra, M; Salazar-Garcia, DC; Royo-Guillen, JI; Kunst, M; Rougier, H; Crevecoeur, I; Arcusa-Magallon, H; Tejedor-Rodriguez, C; de Lagran, IGM; Garrido-Pena, R; Alt, KW; Jeong, C; Schiffels, S; Utrilla, P; Krause, J; Haak, W | 2019 | Survival of Late Pleistocene Hunter-Gatherer Ancestry in the Iberian Peninsula | The Iberian Peninsula in southwestern Europe represents an important test case for the study of human population movements during prehistoric periods. During the Last Glacial Maximum (LGM), the peninsula formed a periglacial refugium [1] for hunter-gatherers (HGs) and thus served as a potential source for the re-peopling of northern latitudes [2]. The post-LGM genetic signature was previously described as a cline from Western HG (WHG) to Eastern HG (EHG), further shaped by later Holocene expansions from the Near East and the North Pontic steppes [ 3-9]. Western and central Europe were dominated by ancestry associated with the similar to 14,000-year-old individual from Villabruna, Italy, which had largely replaced earlier genetic ancestry, represented by 19,000-15,000-year-old individuals associated with the Magdalenian culture [2]. However, little is known about the genetic diversity in southern European refugia, the presence of distinct genetic clusters, and correspondence with geography. Here, we report new genome-wide data from 11 HGs and Neolithic individuals that highlight the late survival of Paleolithic ancestry in Iberia, reported previously in Magdalenian-associated individuals. We show that all Iberian HGs, including the oldest, a similar to 19,000-year-old individual from El Miran in Spain, carry dual ancestry from both Villabruna and the Magdalenian-related individuals. Thus, our results suggest an early connection between two potential refugia, resulting in a genetic ancestry that survived in later Iberian HGs. Our new genomic data from Iberian Early and Middle Neolithic individuals show that the dual Iberian HG genomic legacy pertains in the peninsula, suggesting that expanding farmers mixed with local HGs. | NA | no | No good fitness measure and measure host GD | NA | no | no |
| 5749 | 2020 | 44113 | Villanova, VL; Hughes, PT; Hoffman, EA | 2017 | Combining genetic structure and demographic analyses to estimate persistence in endangered Key deer (Odocoileus virginianus clavium) | Recent improvements in genetic analyses have paved the way in using molecular data to answer questions regarding evolutionary history, genetic structure, and demography. Key deer are a federally endangered subspecies assumed to be genetically unique, homogeneous, and have a female-biased population of approximately 900 deer. We used 985 bp of the mitochondrial cytochrome b gene and 12 microsatellite loci to test two hypotheses: (1) that Key deer are isolated and have reduced diversity compared to mainland deer and (2) that isolation of the Florida Keys has led to a small population size and a high risk of extinction. Our results indicate that Key deer are indeed genetically isolated from mainland white-tailed deer and that there is a lack of genetic substructure between islands. While Key deer exhibit reduced levels of genetic diversity compared to their mainland counterparts, they contain enough diversity to uniquely identify individual deer. Based on genetic identification, we estimated a census size of around 1000 individuals with a heavily skewed female-biased adult sex ratio. Furthermore, our genetic and contemporary demographic data were used to generate a species persistence model of the Key deer. Sensitivity tests within the population viability analysis brought to light the importance of fetal sex ratio and female survival as the primary factors at risk of driving the subspecies to extinction. This study serves as a prime example of how persistence models can be used to evaluate population viability in natural populations of endangered organisms. | NA | no | No good fitness measure and measure host GD | NA | no | no |
| 5750 | 2020 | 44043 | Villero-Wolf, Y; Mattar, S; Puerta-Gonzalez, A; Arrieta, G; Muskus, C; Hoyos, R; Pinzon, H; Pelaez-Carvajal, D | 2019 | Genomic epidemiology of Chikungunya virus in Colombia reveals genetic variability of strains and multiple geographic introductions in outbreak, 2014 | Chikungunya virus (CHIKV) is considered a public health problem due to its rapid spread and high morbidity. This study aimed to determine the genetic diversity and phylogenetic relationships of CHIKVs in Colombia. A descriptive and retrospective study was carried out using sera of patients infected with Chikungunya during the outbreak in Colombia. The whole genomes of CHIKV (n = 16) were sequenced with an Illumina Hi-seq 2500 and were assembled using the Iterative Virus Assembler software. A Bayesian inference phylogenetic analysis was carried out with 157 strains of worldwide origin. The Colombian CHIKV sequences were grouped in the Asian genotype; however, three independent phylogenetic subclades were observed, probably the result of three separate introductions from Panama, Nicaragua, and St. Barts. Each subclade showed several different non-synonymous mutations (nsP2-A153V; nsp2-Y543H; nsp2-G720A; nsP3-L458P; Capside R78Q), that may have functional consequences for CHIKV biology and pathogenesis. These same mutations may affect the efficacy of potential CHIKV vaccines. | NA | no | No good fitness measure and measure host GD | NA | no | no |
| 5751 | 2020 | 44041 | Virdi, JS; Singhal, N; Maurya, AK; Ramamurthy, T; Singh, P; Singhvi, N | 2019 | Medical Microbiology in India: The Recent Developments in the Basic Research, Diagnostics and Vaccines | Medical microbiology being the largest subset under microbiology brings out the benefits of the research in the field of microbiology for the welfare of the masses through applied medical science. Researchers around the globe are working tirelessly in this field in order to prevent, diagnose and treat infectious diseases well in time. India being a tropical country with poor socio-economic conditions and slack healthcare schemes has wide range and burden of the infectious diseases. This has led to the researchers here to take up the challenging tasks of medical microbiology research, they are actively working and the present article reviews the exemplary contribution of these researchers from India in the last decade. This enlists the basic research carried to assess the infections caused by Rotavirus, Human Immunodeficiency Virus (HIV), Pathogenic Vibrios, Mycobacterium tuberculosis, M. leprae, Salmonella enterica servovar typhi, Helicobacter pylori, Yersinia enterocolitica, Leptospires, Leishmania, Plasmodium falciparum, P. vivax, Wuchereria bancrofti, Entamoeba histolytica and some other pathogens. The knowledge gathered through the basic research has been applied for the development of certain vaccines and diagnostic tools and/or techniques to tackle infectious diseases. Notably, Mycobacterium Indicus Prani vaccine (against certain Mycobacterial infections), Rotavac (against rotavirus), JENVAC (against Japanese encephalitis), recombinant vaccine DENVs (against dengue virus) etc. have been developed by Indian research groups and have found wide application. The present article briefly reviews such breakthrough developments in the field of applied microbiology along with a concise account of the infectious diseases outbreaks that resulted from inadequate containments of the vectors. | NA | no | No good fitness measure and measure host GD | NA | no | no |
| 5752 | 2020 | 44113 | Vitale, AA; McKinney, ST; Linden, DW | 2018 | Maternal effect and interactions with philopatry in subadult female American black bear, Ursus americanus, den selection | The selection of suitable locations to fulfil life history requisites is an important process that can influence the survival and development of individuals and the persistence of populations. The impacts of site selection are well documented across taxa, but the driving forces behind site selection, especially regarding transgenerational behaviour, are less well understood. Philopatry (the tendency for an individual to remain near its natal home range) and maternal effect (a nongenetic transfer of phenotypes from mother to offspring) are two factors often thought to influence birth site selection behaviours. We investigated black bear, Ursus americanus, transgenerational den type selection behaviour by analysing a long-term data set containing den selection data of 168 2-year-old subadult females during 1984-2013 at four study sites in Maine, U.S.A. to determine whether there is evidence of philopatry and maternal effect on den type selection. Using logistic regression and model selection, we found evidence that both mechanisms play a role in subadult den type selection. Bears that spent the last winter with their mother in a high-protection den were more likely to select that den type as a subadult. Furthermore, in instances of differences in transgenerational den type selection, most subadults (79%) transitioned from a yearling den of lower protection to a subadult den of higher protection. Transitional behaviour might indicate that subadult black bears also account for environmental plasticity and mediate their den selection accordingly, without the benefit of prior independent denning experience. Future research should evaluate the relationship between den type and offspring recruitment to examine whether an individual-level maternal effect is manifested at the population level. Our results suggest that behavioural maternal effects, in part, influence transgenerational den type selection in black bears, and by extension may influence the behavioural phenotypes of other species with extended periods of maternal care. (c) 2018 The Association for the Study of Animal Behaviour. Published by Elsevier Ltd. All rights reserved. | NA | no | No good fitness measure and measure host GD | NA | no | no |
| 5753 | 2020 | 44040 | Volkova, PA; Burlakov, YA; Schanzer, IA | 2020 | Genetic variability of Prunus padus (Rosaceae) elaborates a new Eurasian phylogeographical paradigm | The extent of glacial survival of woody plants in temperate Asia is still poorly known. A reliable way to clarify this issue in the absence of sufficient paleontological data is a phylogeographic analysis of contemporary populations. A recent study of Juniperus communis genetic diversity in Eurasia suggested that this species with wide ecological tolerance survived the glaciation in many periglacial microrefugia at high latitudes and subsequently spread to new areas during interglacials (Hantemirova et al. in J Biogeogr 44:271-282, 2017. 10.1111/jbi.12867). This pattern was termed a new Eurasian phylogeographical paradigm as opposed to survival in few major refugia. We have tested the proposed paradigm with another hardy species with wide Eurasian area, Prunus padus, to find out if any general phylogeographic patterns may exist for cold-tolerant Eurasian arboreal plant species. We interpret the observed genetic structure [nuclear (ITS) and plastid DNA] of the Eurasian populations of P. padus as plausibly resulted from at least two cycles of glacial survivals in refugia followed by post-glacial colonization events. The species likely originated in East Asia and subsequently spread across all Eurasia. Its continuous range had been fragmented by early-Pleistocene glaciations, when the species survived in the Caucasian and Far Eastern refugia as well as in northern periglacial microrefugia with an active gene flow between them. The known major glacial refugia, such as Iberian Peninsula, the Colchis, the Southern Urals, and the Beringia, played little role as a source of the species post-glacial expansion. | NA | no | No good fitness measure and measure host GD | NA | no | no |
| 5754 | 2020 | 44111 | von Crautlein, M; Leinonen, PH; Korpelainen, H; Helander, M; Vare, H; Saikkonen, K | 2019 | Postglacial colonization history reflects in the genetic structure of natural populations of Festuca rubra in Europe | We conducted a large-scale population genetic survey of genetic diversity of the host grass Festuca rubra s.l., which fitness can be highly dependent on its symbiotic fungus Epichloe festucae, to evaluate genetic variation and population structure across the European range. The 27 studied populations have previously been found to differ in frequencies of occurrence of the symbiotic fungus E. festucae and ploidy levels. As predicted, we found decreased genetic diversity in previously glaciated areas in comparison with nonglaciated regions and discovered three major maternal genetic groups: southern, northeastern, and northwestern Europe. Interestingly, host populations from Greenland were genetically similar to those from the Faroe Islands and Iceland, suggesting gene flow also between those areas. The level of variation among populations within regions is evidently highly dependent on the postglacial colonization history, in particular on the number of independent long-distance seed colonization events. Yet, also anthropogenic effects may have affected the population structure in F. rubra. We did not observe higher fungal infection rates in grass populations with lower levels of genetic variability. In fact, the fungal infection rates of E. festucae in relation to genetic variability of the host populations varied widely among geographical areas, which indicate differences in population histories due to colonization events and possible costs of systemic fungi in harsh environmental conditions. We found that the plants of different ploidy levels are genetically closely related within geographic areas indicating independent formation of polyploids in different maternal lineages. | NA | no | gd of pathogen | NA | no | no |
| 5755 | 2020 | 44041 | Vung, NN; Choi, YS; Kim, I | 2020 | High resistance to Sacbrood virus disease in Apis cerana (Hymenoptera: Apidae) colonies selected for superior brood viability and hygienic behavior | Sacbrood virus (SBV) caused significant colony collapse in Korean Apis cerana. Therefore, breeding of resistant bees to counter this viral disease is urgently needed. Considering that hygienic behavior in honey bees confers colony-level resistance against brood diseases, we utilized this trait for selecting A. cerana colonies. In addition, the brood survival rate was evaluated after colonies were SBV-inoculated. Over four selective generations, dead brood removal and brood survivorship in selected colonies were higher than those in the controls, which were not selectively bred for those traits (P < 0.01, 99.3 vs. 89.9% for removal of pin-killed pupae; P < 0.01, 99.0 vs. 63.9% for removal of SBV-killed larvae; and P < 0.01, 70.0 vs. 9.2% for brood survivorship). Following SBV inoculation, selected colonies showed an increase in the number of surviving pupae and adults, whereas control colonies collapsed mostly. Our results confirm the feasibility of selecting SBV-resistant A. cerana. | NA | no | selectivley bred colonies, no good gd or fitness comp | NA | no | no |
| 5756 | 2020 | 44112 | Vychodilova, L; Necesankova, M; Albrechtova, K; Hlavac, J; Modry, D; Janova, E; Vyskocil, M; Mihalca, AD; Kennedy, LJ; Horin, P | 2018 | Genetic diversity and population structure of African village dogs based on microsatellite and immunity-related molecular markers | The village and street dogs represent a unique model of canine populations. In the absence of selective breeding and veterinary care, they are subject mostly to natural selection. Their analyses contribute to understanding general mechanisms governing the genetic diversity, evolution and adaptation. In this study, we analyzed the genetic diversity and population structure of African village dogs living in villages in three different geographical areas in Northern Kenya. Data obtained for neutral microsatellite molecular markers were compared with those computed for potentially non-neutral markers of candidate immunity-related genes. The neutral genetic diversity was similar to other comparable village dog populations studied so far. The overall genetic diversity in microsatellites was higher than the diversity of European pure breeds, but it was similar to the range of diversity observed in a group composed of many European breeds, indicating that the African population has maintained a large proportion of the genetic diversity of the canine species as a whole. Microsatellite marker diversity indicated that the entire population is subdivided into three genetically distinct, although closely related subpopulations. This genetical partitioning corresponded to their geographical separation and the observed gene flow well correlated with the communication patterns among the three localities. In contrast to neutral microsatellites, the genetic diversity in immunity-related candidate SNP markers was similar across all three subpopulations and to the European group. It seems that the genetic structure of this particular population of Kenyan village dogs is mostly determined by geographical and anthropogenic factors influencing the gene flow between various subpopulations rather than by biological factors, such as genetic contribution of original migrating populations and/or the pathogenmediated selection. On the other hand, the study of oldest surviving dogs suggested a biological mechanism, i.e. a possible advantage of the overal heterozygosity marked by the the microsatellite loci analyzed. | NA | no | No good fitness measure and measure host GD | NA | no | no |
| 5757 | 2020 | 44111 | Vyskocilova, S; Seal, S; Colvin, J | 2019 | Relative polyphagy of Mediterranean cryptic Bemisia tabaci whitefly species and global pest status implications | The Bemisia tabaci whitefly was previously considered a single, highly polyphagous species, but is now accepted as a group of cryptic biological species. We investigated the host plant relations of two sister species, the Mediterranean (MED) composed of the Q1 and Q2 mitochondrial groups and the ASL species (formerly considered a MED group), to discover whether polyphagy was related to the global pest status. We compared their performance by measuring the oviposition rate, survival, fecundity and proportion of female offspring on 13 host plants from nine families. In addition, oviposition preference was compared among leaves of different ages. Significant (P<0.05) differences were found between populations in all parameters, particularly in adult survival and fecundity. Females preferred strongly to oviposit on the oldest true leaf on tobacco and tomato and on young leaves of sweet potato and pepper. The greatest differences in fecundity occurred on bean, okra, squash, pepper and tobacco. Hosts favourable for all four populations were cotton and sweet potato; no offspring were produced on cassava, chard or tomato. Host ranges of Q1 populations from Europe and sub-Saharan Africa differed despite their close genetic relatedness at the mitogenome level. Discrepancies between the parental and offspring fitness were observed. Our findings show that (1) the species have differing but overlapping host plant ranges and (2) the Q1 is the most polyphagous and can utilise tobacco, which predisposes it to evolving resistance to neonicotinoids. Our findings contribute to the understanding of ecology of this pest species complex and aid the development of efficient pest control strategies. | NA | no | No good fitness measure and measure host GD | NA | no | no |
| 5758 | 2020 | 44112 | Wacker, S; Larsen, BM; Jakobsen, P; Karlsson, S | 2018 | High levels of multiple paternity in a spermcast mating freshwater mussel | Multiple paternity is an important characteristic of the genetic mating system and common across a wide range of taxa. Multiple paternity can increase within-population genotypic diversity, allowing selection to act on a wider spectre of genotypes, and potentially increasing effective population size. While the genetic mating system has been studied in many species with active mating behavior, little is known about multiple paternity in sessile species releasing gametes into the water. In freshwater mussels, males release sperm into the water, while eggs are retained and fertilized inside the female (spermcast mating). Mature parasitic glochidia are released into the water and attach to the gills of fish where they are encapsulated until settling in the bottom substrate. We used 15 microsatellite markers to detect multiple paternity in a wild population of the freshwater pearl mussel (Margaritifera margaritifera). We found multiple paternity in all clutches for which more than two offspring were genotyped, and numbers of sires were extremely high. Thirty-two sires had contributed to the largest clutch (43 offspring sampled). This study provides the first evidence of multiple paternity in the freshwater pearl mussel, a species that has experienced dramatic declines across Europe. Previous studies on other species of freshwater mussels have detected much lower numbers of sires. Multiple paternity in freshwater pearl mussels may be central for maintaining genetic variability in small and fragmented populations and for their potential to recover after habitat restoration and may also be important in the evolutionary arms race with their fish host with a much shorter generation time. | NA | no | No good fitness measure | NA | no | no |
| 5759 | 2020 | 44043 | Wacker, S; Larsen, BM; Karlsson, S; Hindar, K | 2019 | Host specificity drives genetic structure in a freshwater mussel | Parasites often depend on their hosts for long distance transport, and genetic population structure can be strongly affected by host specificity and dispersal. Freshwater pearl mussel (Margaritifera margaritifera) populations have previously been found to naturally infest either primarily Atlantic salmon (‘salmon-mussel’) or exclusively brown trout (‘trout-mussel’) across a wide geographic range. Here, we experimentally test whether this intraspecific variation in natural infestation can be explained by host specificity in freshwater pearl mussel. Our experiments show that when both host species were exposed to larvae from salmon-and trout-mussel respectively, salmon-mussel larvae almost never infested brown trout and vice versa. This suggests that host specificity can explain variation in natural infestation among the studied freshwater pearl mussel populations. Host specificity provides a link to the species’ variable population genetic structure, as mussel populations limited to Atlantic salmon, the host with stronger dispersal, show higher genetic diversity and weaker differentiation than populations limited to brown trout as host. | NA | no | No good fitness measure and measure host GD | NA | no | no |
| 5760 | 2020 | 44041 | Walas, L; Ganatsas, P; Iszkulo, G; Thomas, PA; Dering, M | 2019 | Spatial genetic structure and diversity of natural populations of Aesculus hippocastanum L. in Greece | Horse-chestnut (Aesculus hippocastanum L.) is an endemic and relict species from the Mediterranean biodiversity hotspot and a popular ornamental tree. Knowledge about the evolutionary history of this species remains scarce. Here, we ask what historical and ecological factors shaped the pattern of genetic diversity and differentiation of this species. We genotyped 717 individuals from nine natural populations using microsatellite markers. The influence of distance, topography and habitat variables on spatial genetic structure was tested within the approaches of isolation-by-distance and isolation-by-ecology. Species niche modeling was used to project the species theoretical range through time and space. The species showed high genetic diversity and moderate differentiation for which topography, progressive range contraction through the species’ history and long-term persistence in stable climatic refugia are likely responsible. A strong geographic component was revealed among five genetic clusters that are connected with very limited gene flow. The environmental variables were a significant factor in the spatial genetic structure. Modeling results indicated that future reduction of the species range may affect its survival. The possible impact of climate changes and high need of in situ conservation are discussed. | NA | no | No good fitness measure | NA | no | no |
| 5761 | 2020 | 44040 | Waldvogel, AM; Feldmeyer, B; Rolshausen, G; Exposito-Alonso, M; Rellstab, C; Kofler, R; Mock, T; Schmid, K; Schmitt, I; Bataillon, T; Savolainen, O; Bergland, A; Flatt, T; Guillaume, F; Pfenninger, M | 2020 | Evolutionary genomics can improve prediction of species’ responses to climate change | Global climate change (GCC) increasingly threatens biodiversity through the loss of species, and the transformation of entire ecosystems. Many species are challenged by the pace of GCC because they might not be able to respond fast enough to changing biotic and abiotic conditions. Species can respond either by shifting their range, or by persisting in their local habitat. If populations persist, they can tolerate climatic changes through phenotypic plasticity, or genetically adapt to changing conditions depending on their genetic variability and census population size to allow for de novo mutations. Otherwise, populations will experience demographic collapses and species may go extinct. Current approaches to predicting species responses to GCC begin to combine ecological and evolutionary information for species distribution modelling. Including an evolutionary dimension will substantially improve species distribution projections which have not accounted for key processes such as dispersal, adaptive genetic change, demography, or species interactions. However, eco-evolutionary models require new data and methods for the estimation of a species’ adaptive potential, which have so far only been available for a small number of model species. To represent global biodiversity, we need to devise large-scale data collection strategies to define the ecology and evolutionary potential of a broad range of species, especially of keystone species of ecosystems. We also need standardized and replicable modelling approaches that integrate these new data to account for eco-evolutionary processes when predicting the impact of GCC on species’ survival. Here, we discuss different genomic approaches that can be used to investigate and predict species responses to GCC. This can serve as guidance for researchers looking for the appropriate experimental setup for their particular system. We furthermore highlight future directions for moving forward in the field and allocating available resources more effectively, to implement mitigation measures before species go extinct and ecosystems lose important functions. | NA | no | No good fitness measure and measure host GD | NA | no | no |
| 5762 | 2020 | 44112 | Walugembe, M; Bertolini, F; Dematawewa, CMB; Reis, MP; Elbeltagy, AR; Schmidt, CJ; Lamont, SJ; Rothschild, MF | 2019 | Detection of Selection Signatures Among Brazilian, Sri Lankan, and Egyptian Chicken Populations Under Different Environmental Conditions | Extreme environmental conditions are a major challenge in livestock production. Changes in climate, particularly those that contribute to weather extremes like drought or excessive humidity, may result in reduced performance and reproduction and could compromise the animal’s immune function. Animal survival within extreme environmental conditions could be in response to natural selection and to artificial selection for production traits that over time together may leave selection signatures in the genome. The aim of this study was to identify selection signatures that may be involved in the adaptation of indigenous chickens from two different climatic regions (Sri Lanka = Tropical; Egypt = Arid) and in non-indigenous chickens that derived from human migration events to the generally tropical State of Sao Paulo, Brazil. To do so, analyses were conducted using fixation index (Fst) and hapFLK analyses. Chickens from Brazil (n = 156), Sri Lanka (n = 92), and Egypt (n = 96) were genotyped using the Affymetrix AxiomR (R) 600k Chicken Genotyping Array. Pairwise Fst analyses among countries did not detect major regions of divergence between chickens from Sri Lanka and Brazil, with ecotypes/breeds from Brazil appearing to be genetically related to Asian-Indian (Sri Lanka) ecotypes. However, several differences were detected in comparisons of Egyptian with either Sri Lankan or Brazilian populations, and common regions of difference on chromosomes 2, 3 and 8 were detected. The hapFLK analyses for the three separate countries suggested unique regions that are potentially under selection on chromosome 1 for all three countries, on chromosome 4 for Sri Lankan, and on chromosomes 3, 5, and 11 for the Egyptian populations. Some of identified regions under selection with hapFLK analyses contained genes such as TLR3, SOCS2, EOMES, and NFAT5 whose biological functions could provide insights in understanding adaptation mechanisms in response to arid and tropical environments. | NA | no | No good fitness measure and measure host GD | NA | no | no |
| 5763 | 2020 | 44111 | Wang, BS; Mojica, JP; Perera, N; Lee, CR; Lovell, JT; Sharma, A; Adam, C; Lipzen, A; Barry, K; Rokhsar, DS; Schmutz, J; Mitchell-Olds, T | 2019 | Ancient polymorphisms contribute to genome-wide variation by long-term balancing selection and divergent sorting in Boechera stricta | BackgroundGenomic variation is widespread, and both neutral and selective processes can generate similar patterns in the genome. These processes are not mutually exclusive, so it is difficult to infer the evolutionary mechanisms that govern population and species divergence. Boechera stricta is a perennial relative of Arabidopsis thaliana native to largely undisturbed habitats with two geographic and ecologically divergent subspecies. Here, we delineate the evolutionary processes driving the genetic diversity and population differentiation in this species.ResultsUsing whole-genome re-sequencing data from 517 B. stricta accessions, we identify four genetic groups that diverged around 30-180 thousand years ago, with long-term small effective population sizes and recent population expansion after the Last Glacial Maximum. We find three genomic regions with elevated nucleotide diversity, totaling about 10% of the genome. These three regions of elevated nucleotide diversity show excess of intermediate-frequency alleles, higher absolute divergence (d(XY)), and lower relative divergence (F-ST) than genomic background, and significant enrichment in immune-related genes, reflecting long-term balancing selection. Scattered across the genome, we also find regions with both high F-ST and d(XY) among the groups, termed F-ST-islands. Population genetic signatures indicate that F-ST-islands with elevated divergence, which have experienced directional selection, are derived from divergent sorting of ancient polymorphisms.ConclusionsOur results suggest that long-term balancing selection on disease resistance genes may have maintained ancestral haplotypes across different geographical lineages, and unequal sorting of balanced polymorphisms may have generated genomic regions with elevated divergence. This study highlights the importance of ancestral balanced polymorphisms as crucial components of genome-wide variation. | NA | no | No good fitness measure | NA | no | no |
| 5764 | 2020 | 44041 | Wang, C; Scott, SM; Subramanian, K; Loguercio, S; Zhao, P; Hutt, DM; Farhat, NY; Porter, FD; Balch, WE | 2019 | Quantitating the epigenetic transformation contributing to cholesterol homeostasis using Gaussian process | To understand the impact of epigenetics on human misfolding disease, we apply Gaussian-process regression (GPR) based machine learning (ML) (GPR-ML) through variation spatial profiling (VSP). VSP generates population-based matrices describing the spatial covariance (SCV) relationships that link genetic diversity to fitness of the individual in response to histone deacetylases inhibitors (HDACi). Niemann-Pick C1 (NPC1) is a Mendelian disorder caused by >300 variants in the NPC1 gene that disrupt cholesterol homeostasis leading to the rapid onset and progression of neurodegenerative disease. We determine the sequence-to-function-to-structure relationships of the NPC1 polypeptide fold required for membrane trafficking and generation of a tunnel that mediates cholesterol flux in late endosomal/lysosomal (LE/Ly) compartments. HDACi treatment reveals unanticipated epigenomic plasticity in SCV relationships that restore NPC1 functionality. GPR-ML based matrices capture the epigenetic processes impacting information flow through central dogma, providing a framework for quantifying the effect of the environment on the healthspan of the individual. | NA | no | No good fitness measure and measure host GD | NA | no | no |
| 5765 | 2020 | 44040 | Wang, C; Scott, SM; Sun, SH; Zhao, P; Hutt, DM; Shao, H; Gestwicki, JE; Balch, WE | 2020 | Individualized management of genetic diversity in Niemann-Pick C1 through modulation of the Hsp70 chaperone system | Genetic diversity provides a rich repository for understanding the role of proteostasis in the management of the protein fold in human biology. Failure in proteostasis can trigger multiple disease states, affecting both human health and lifespan. Niemann-Pick C1 (NPC1) disease is a rare genetic disorder triggered by mutations in NPC1, a multi-spanning transmembrane protein that is trafficked through the exocytic pathway to late endosomes (LE) and lysosomes (Ly) (LE/Ly) to globally manage cholesterol homeostasis. Defects triggered by >300 NPC1 variants found in the human population inhibit export of NPC1 protein from the endoplasmic reticulum (ER) and/or function in downstream LE/Ly, leading to cholesterol accumulation and onset of neurodegeneration in childhood. We now show that the allosteric inhibitor JG98, that targets the cytosolic Hsp70 chaperone/co-chaperone complex, can significantly improve the trafficking and post-ER protein level of diverse NPC1 variants. Using a new approach to model genetic diversity in human disease, referred to as variation spatial profiling, we show quantitatively how JG98 alters the Hsp70 chaperone/co-chaperone system to adjust the spatial covariance (SCV) tolerance and set-points on an amino acid residue-by-residue basis in NPC1 to differentially regulate variant trafficking, stability, and cholesterol homeostasis, results consistent with the role of BCL2-associated athanogene family co-chaperones in managing the folding status of NPC1 variants. We propose that targeting the cytosolic Hsp70 system by allosteric regulation of its chaperone/co-chaperone based client relationships can be used to adjust the SCV tolerance of proteostasis buffering capacity to provide an approach to mitigate systemic and neurological disease in the NPC1 population. | NA | no | human | NA | no | no |
| 5766 | 2020 | 44040 | Wang, X; Bernhardsson, C; Ingvarsson, PK | 2020 | Demography and Natural Selection Have Shaped Genetic Variation in the Widely Distributed Conifer Norway Spruce (Picea abies) | Under the neutral theory, species with larger effective population size are expected to harbor higher genetic diversity. However, across a wide variety of organisms, the range of genetic diversity is orders of magnitude more narrow than the range of effective population size. This observation has become known as Lewontin’s paradox and although aspects of this phenomenon have been extensively studied, the underlying causes for the paradox remain unclear. Norway spruce (Picea abies) is a widely distributed conifer species across the northern hemisphere, and it consequently plays a major role in European forestry. Here, we use whole-genome resequencing data from 35 individuals to perform population genomic analyses in P. abies in an effort to understand what drives genome-wide patterns of variation in this species. Despite having a very wide geographic distribution and an corresponding enormous current population size, our analyses find that genetic diversity of P. abies is low across a number of populations (pi = 0.0049 in Central-Europe, pi = 0.0063 in Sweden-Norway, pi = 0.0063 in Finland). To assess the reasons for the low levels of genetic diversity, we infer the demographic history of the species and find that it is characterized by several reoccurring bottlenecks with concomitant decreases in effective population size can, at least partly, provide an explanation for low polymorphism we observe in P. abies. Further analyses suggest that recurrent natural selection, both purifying and positive selection, can also contribute to the loss of genetic diversity in Norway spruce by reducing genetic diversity at linked sites. Finally, the overall low mutation rates seen in conifers can also help explain the low genetic diversity maintained in Norway spruce. | NA | no | No good fitness measure and measure host GD | NA | no | no |
| 5767 | 2020 | 44113 | Wang, XL; Philip, VM; Ananda, G; White, CC; Malhotra, A; Michalski, PJ; Karuturi, KRM; Chintalapudi, SR; Acklin, C; Sasner, M; Bennett, DA; De Jager, PL; Howell, GR; Carter, GW | 2018 | A Bayesian Framework for Generalized Linear Mixed Modeling Identifies New Candidate Loci for Late-Onset Alzheimer’s Disease | Recent technical and methodological advances have greatly enhanced genome-wide association studies (GWAS). The advent of low-cost, whole-genome sequencing facilitates high-resolution variant identification, and the development of linear mixed models (LMM) allows improved identification of putatively causal variants. While essential for correcting false positive associations due to sample relatedness and population stratification, LMMs have commonly been restricted to quantitative variables. However, phenotypic traits in association studies are often categorical, coded as binary case-control or ordered variables describing disease stages. To address these issues, we have devised a method for genomic association studies that implements a generalized LMM (GLMM) in a Bayesian framework, called Bayes-GLMM. Bayes-GLMM has four major features: (1) support of categorical, binary, and quantitative variables; (2) cohesive integration of previous GWAS results for related traits; (3) correction for sample relatedness by mixed modeling; and (4) model estimation by both Markov chain Monte Carlo sampling and maximal likelihood estimation. We applied Bayes-GLMM to the whole-genome sequencing cohort of the Alzheimer’s Disease Sequencing Project. This study contains 570 individuals from 111 families, each with Alzheimer’s disease diagnosed at one of four confidence levels. Using Bayes-GLMM we identified four variants in three loci significantly associated with Alzheimer’s disease. Two variants, rs140233081 and rs149372995, lie between PRKAR1B and PDGFA. The coded proteins are localized to the glial-vascular unit, and PDGFA transcript levels are associated with Alzheimer’s disease-related neuropathology. In summary, this work provides implementation of a flexible, generalized mixed-model approach in a Bayesian framework for association studies. | NA | no | No good fitness measure and measure host GD | NA | no | no |
| 5768 | 2020 | 44041 | Wang, Y; Luo, LJ; Li, Q; Wang, H; Wang, YQ; Sun, H; Xu, JG; Lan, RT; Ye, CY | 2019 | Genomic dissection of the most prevalent Listeria monocytogenes clone, sequence type ST87, in China | Background: Listeria monocytogenes consists of four lineages that occupy a wide variety of ecological niches. Sequence type (ST) 87 (serotype 1/2b), belonging to lineage I, is one of the most common STs isolated from food products, food associated environments and sporadic listeriosis in China. Here, we performed a comparative genomic analysis of the L. monocytogenes ST87 clone by sequencing 71 strains representing a diverse range of sources, different geographical locations and isolation years. Results: The core genome and pan genome of ST87 contained 2667 genes and 3687 genes respectively. Phylogenetic analysis based on core genome SNPs divided the 71 strains into 10 clades. The clinical strains were distributed among multiple clades. Four clades contained strains from multiple geographic regions and showed high genetic diversity. The major gene content variation of ST87 genomes was due to putative prophages, with eleven hotspots of the genome that harbor prophages. All strains carry an intact CRISRP/Cas system. Two major CRISPR spacer profiles were found which were not clustered phylogenetically. A large plasmid of about 90 Kb, which carried heavy metal resistance genes, was found in 32.4% (23/71) of the strains. All ST87 strains harbored the Listeria pathogenicity island (LIPI)-4 and a unique 10-open read frame (ORF) genomic island containing a novel restriction-modification system. Conclusion: Whole genome sequence analysis of L. monocytogenes ST87 enabled a clearer understanding of the population structure and the evolutionary history of ST87 L. monocytogenes in China. The novel genetic elements identified may contribute to its virulence and adaptation to different environmental niches. Our findings will be useful for the development of effective strategies for the prevention and treatment of listeriosis caused by this prevalent clone. | NA | no | No good fitness measure and measure host GD | NA | no | no |
| 5769 | 2020 | 44112 | Wang, YJ; Jiao, AX; Chen, HC; Ma, XD; Cui, D; Han, B; Ruan, RC; Xue, DY; Han, LZ | 2018 | Status and factors influencing on-farm conservation of Kam Sweet Rice (Oryza sativa L.) genetic resources in southeast Guizhou Province, China | BackgroundKam Sweet Rice (KSR) is a special kind of rice landrace that has been cultivated for thousands of years in the borders of Guizhou, Hunan, and Guangxi Provinces of China, and is mainly distributed in southeast Guizhou Province of China currently. KSR has many unique qualities, including strong resistance to diseases, pests, and adverse abiotic conditions, difficulty of threshing, and well glutinous features. KSR germplasm resources are an indispensable material and cultural symbol in the production and daily life and customs of the Dong people. Related studies showed that numerous traditional KSR varieties and cultivation area of KSR decreased sharply from the Qing dynasty to 2015, but many KSR varieties are still conserved in Dong villages of southeast Guizhou Province compared to other areas. However, the number of KSR varieties that are conserved on farms in southeast Guizhou Province and factors influencing the erosion and conservation of KSR genetic resources is unclear. Therefore, this study was an on-farm conservation investigation of KSR genetic resource in China’s major KSR producing areasLiping, Congjiang, and Rongjiang counties in Guizhou Province and influencing factors analysis of KSR abandonment and conservation.MethodsThe information of KSR conservation status and variety characteristics, typical villages, Dong’s cultural customs, and factors influencing KSR abandonment and conservation was obtained using ethno-biology methods, mainly through field research interviews, including participatory observation, semi-structured interviews, key informant interviews, focus group discussions, and cultural anthropology. The altitude, plant height, awn color and length, hull color, and rice color of 156 KSR accessions in 28 villages were recorded. The variety quantity and cultivation area of KSR were investigated in 33 ethnic villages. Questionnaire surveys were conducted in typical Dong villages to obtain local farmers’ attitudes toward cultivation and protection of KSR. We randomly selected 26 farmers from Sizhai village and 30 farmers from Huanggang village and chose 3 social characteristics including age, gender, and education levels of farmers, and adopted the method of face-to-face interviewing to complete the questionnaires. Then, we analyzed the correlation and determined the significance between farmers with different social characteristics and farmers’ attitudes to KSR development and protection using SPSS 17.0 software.Results(1) On-farm conservation status of KSR: a total of 156 KSR varieties were collected from 28 ethnic minority villages from 13 townships (accounting for 21% of three counties) in Liping, Congjiang, and Rongjiang counties. KSR accessions accounted for more than 90% of local rice varieties in each village. According to local farmers, although the quantity of KSR varieties decreased more than 50% in the investigated villages compared to the past 10-20years, some Dong villages have still cultivated KSR, accounting for more than 50% of the rice field area in 10 villages. This result showed that many KSR varieties are still conserved by in Dong villages, and these KSR varieties have a high genetic diversity of phenotypes. (2) Typical villages investigation: the cultivation area of KSR in Congjiang was the highest, 6.7 times larger than Liping and eight times larger than Rongjiang. In addition, the cultivation area of KSR in Dong villages was larger than that in other ethnic villages, and villages that had a higher planting area of KSR had more KSR accessions. (3) Farmers’ attitude toward the development and conservation of KSR: Dong farmers hold the negative attitudes concerning the development of KSR resources, but they thought it is necessary to protect KSR landraces. Especially, a high level of education and female, young, and old farmers played more important roles in the cultivation and protection of KSR.ConclusionsUntil now, some Dong ethnic villages have still cultivated KSR for thousands of years in Qiandongnan area, although the number of varieties and the planting area of KSR have been greatly reduced. In addition, ethnic traditional culture and social customs were the main influencing factors of KSR conservation; economic, management, and policy factors were the main influencing factors of KSR abandonment. Through the analysis of the correlation between farmers with different social characteristics and their attitudes toward the cultivation, reasons for conservation and abandonment, development tendency, and protection of KSR, we found that a high level of education and female, young, and old farmers play more important role in the cultivation and protection of KSR. Therefore, in order to promote the protection and sustainable utilization of KSR, it is necessary to build on-farm conservation of KSR and improve the position of female farmers and the education level of young people, and encourage the old people to educate the middle-aged to conserve and protect KSR as well as Dong’s traditional culture and social customs. This study is of great significance to promote better protection and optimal utilization of KSR and enable the public, government, and related researchers pay more attention to conserving ethnic traditional cultures. | NA | no | No good fitness measure and measure host GD | NA | no | no |
| 5770 | 2020 | 44112 | Wang, YJ; Lu, JM; Beattie, GAC; Islam, MR; Om, N; Dao, HT; Nguyen, LV; Zaka, SM; Guo, J; Tian, MY; Deng, XL; Tan, SY; Holford, P; He, YR; Cen, YJ | 2018 | Phylogeography of Diaphorina citri (Hemiptera: Liviidae) and its primary endosymbiont, ‘Candidatus Carsonella ruddii’: an evolutionary approach to host-endosymbiont interaction | BACKGROUNDIn insects, little is known about the co-evolution between their primary endosymbionts and hosts at the intraspecific level. This study examined co-diversification between the notorious agricultural pest Diaphorina citri and its primary endosymbionts (P-endosymbiont), Candidatus Carsonella ruddii’ at the population level. RESULTSMaximum likelihood, haplotype network, principal components and Bayesian clustering identified three lineages for D. citri and its P-endosymbiont: a Western clade containing individuals from Pakistan, Bhutan (Phuentsholing), Vietnam (Son La), USA, Myanmar and China (Ruili, Yunnan); a Central clade, with accessions originating from Southwest China, Bhutan (Tsirang) and Bangladesh; and an Eastern clade containing individuals from Southeast Asia, and East and South China. A more diverse genetic structure was apparent in the host mitochondrial DNA than their P-endosymbionts; however, the two sets of data were strongly congruent. CONCLUSIONThis study provides evidence for the co-diversification of D. citri and its P-endosymbiont during the migration from South Asia to East and Southeast Asia. We also suggest that the P-endosymbiont may facilitate investigations into the genealogy and migration history of the host. The biogeography of D. citri and its P-endosymbiont indicated that D. citri colonized and underwent a secondary dispersal from South Asia to East and Southeast Asia. (c) 2018 Society of Chemical Industry | NA | no | No good fitness measure and measure host GD | NA | no | no |
| 5771 | 2020 | 44113 | Wang, YJ; Xu, CB; Tian, MY; Deng, XL; Cen, YJ; He, YR | 2017 | Genetic diversity of Diaphorina citri and its endosymbionts across east and south-east Asia | BACKGROUND: Diaphorina citri is the vector of Candidatus Liberibacter asiaticus’, the most widespread pathogen associated huanglongbing, the most serious disease of citrus. To enhance our understanding of the distribution and origin of the psyllid, we investigated the genetic diversity and population structures of 24 populations in Asia and one from Florida based on the mtCOI gene. Simultaneously, genetic diversity and population structures of the primary endosymbiont (P-endosymbiont) Candidatus Carsonella ruddii’ and secondary endosymbiont (S-endosymbiont) Candidatus Profftella armatura’ of D. citri were determined with the housekeeping genes. RESULT: AMOVA analysis indicated that populations of D. citri and its endosymbionts in east and south-east Asia were genetically distinct from populations in Pakistan and Florida. Furthermore, P-endosymbiont populations displayed a strong geographical structure across east and south-east Asia, while low genetic diversity indicated the absence of genetic structure among the populations of D. citri and its S-endosymbiont across these regions. CONCLUSION: The Ca. C. ruddii’ is more diverse and structured than the D. citri and the Ca. P. armatura’ across east and south-east Asia. Multiple introductions of the psyllid have occurred in China. Management application for controlling the pest is proposed based on the genetic information of D. citri and its endosymbionts. (c) 2017 Society of Chemical Industry | NA | no | No good fitness measure and measure host GD | NA | no | no |
| 5772 | 2020 | 44039 | Ware, M; Garfield, EN; Nedimyer, K; Levy, J; Kaufman, L; Precht, W; Winters, RS; Miller, SL | 2020 | Survivorship and growth in staghorn coral (Acropora cervicornis) outplanting projects in the Florida Keys National Marine Sanctuary | Significant population declines in Acropora cervicornis and A. palmata began in the 1970s and now exceed over 90%. The losses were caused by a combination of coral disease and bleaching, with possible contributions from other stressors, including pollution and predation. Reproduction in the wild by fragment regeneration and sexual recruitment is inadequate to offset population declines. Starting in 2007, the Coral Restoration Foundation (TM) evaluated the feasibility of outplanting A. cervicornis colonies to reefs in the Florida Keys to restore populations at sites where the species was previously abundant. Reported here are the results of 20 coral outplanting projects with each project defined as a cohort of colonies outplanted at the same time and location. Photogrammetric analysis and in situ monitoring (2007 to 2015) measured survivorship, growth, and condition of 2419 colonies. Survivorship was initially high but generally decreased after two years. Survivorship among projects based on colony counts ranged from 4% to 89% for seven cohorts monitored at least five years. Weibull survival models were used to estimate survivorship beyond the duration of the projects and ranged from approximately 0% to over 35% after five years and 0% to 10% after seven years. Growth rate averaged 10 cm/year during the first two years then plateaued in subsequent years. After four years, approximately one-third of surviving colonies were >= 50 cm in maximum diameter. Projects used three to sixteen different genotypes and significant differences did not occur in survivorship, condition, or growth. Restoration times for three reefs were calculated based on NOAA Recovery Plan (NRP) metrics (colony abundance and size) and the findings from projects reported here. Results support NRP conclusions that reducing stressors is required before significant population growth and recovery will occur. Until then, outplanting protects against local extinction and helps to maintain genetic diversity in the wild. | NA | no | No good measureGD of host | NA | no | no |
| 5773 | 2020 | 44113 | Wedrowicz, F; Mosse, J; Wright, W; Hogan, FE | 2018 | Genetic structure and diversity of the koala population in South Gippsland, Victoria: a remnant population of high conservation significance | In the Australian state of Victoria, the history of koalas and their management has resulted in the homogenisation and reduction of genetic diversity in many contemporary populations. Decreased genetic diversity may reduce a species’ ability to adapt to future environmental pressures such as climate change or disease. The South Gippsland koala population is considered to be unique in Victoria, as it is believed to be a remnant population, not originating from managed populations that have low genetic variation. This study investigated genetic structure and diversity of koalas in South Gippsland, with comparison to other populations in Victoria (French Island/Cape Otway, FI and Raymond Island, RI), New South Wales and south east Queensland. Population analyses were undertaken using both microsatellite genotype and mitochondrial DNA sequence data. Non-invasive sampling of koala scats was used to source koala DNA, allowing 222 South Gippsland koalas to be genotyped. Using nuclear data the South Gippsland koala population was found to be significantly differentiated (D-jost 95% CI SG-RI = 0.03-0.06 and SG-FI = 0.08-012) and more diverse (A(R) 95% CI SG = 4.7-5.6, RI = 3.1-3.3, FI = 3.0-3.3; p = 0.001) than other Victorian koala populations, supporting the premise that koalas in the South Gippsland region are part of a remnant population, not derived from translocated island stock. These results were also supported by mitochondrial data where eight haplotypes (Pc4, Pc17, Pc26, Pc27, and Pc56-Pc59) were identified in South Gippsland while a single haplotype (Pc27) was found in all island koalas tested. Compared to other Victorian koala populations, greater genetic diversity found in South Gippsland koalas, may provide this population with a greater chance of survival in the face of future environmental pressures. The South Gippsland koala population is, therefore, of high conservation significance, warranting the implementation of strategies to conserve this population and its diversity into the future. | NA | no | No good fitness measure | NA | no | no |
| 5774 | 2020 | 44112 | Wei, M; Feng, M; Guan, Y; Guo, C; Zhou, H; Fu, YF; Tachibana, H; Cheng, XJ | 2018 | Correlation of genetic diversity between hosts and parasites in Entamoeba nuttalli isolates from Tibetan and rhesus macaques in China | Entamoeba nuttalli infection is prevalent in captive and wild macaques. Recent studies have suggested that genotypes of E. nuttalli isolates are correlated with the geographical distribution of host macaques. Correlation of amoebic genotypes with genetic diversity of host macaques was analyzed in present study. Sixty fresh stool samples were obtained from wild Tibetan macaques living in Mount Huang (HS) of the An-hui Province in China. PCR analysis revealed that the most prevalent Entamoeba species was E. chattoni (E. polecki ST2) (86.7%) followed by E. nuttalli (58.3%) and E. coli (25%). Six E. nuttalli HS isolates were successfully cultured. The tRNA-linked short tandem repeat (STR) loci and serine-rich protein gene of E. nuttalli isolates from four different regions of China (Mount Long-hu, Gui-yang, Mount E-mei, and HS, the former three isolates were obtained in previous studies) were studied and high numbers of polymorphisms were detected. When genetic diversity of different populations of E. nuttalli isolates was compared with geographical distance, an r(2) value of 0.919 was assigned by a Mantel test based on the tRNA-STR loci. In host macaques, the mtDNA HVS-I gene was also highly polymorphic in each of the genomes. Multiple regression analysis using E. nuttalli tRNA-STR loci genetic, macaque mtDNA HVS-I gene, and geographic distances showed an r(2) value of 0.943, indicating that a higher relevance was demonstrated when geographic and host gene factors were considered. Analysis of genetic factor of host would benefit for better understanding of the evolution of E. nuttalli. | NA | no | no good gd measure host pop | NA | no | no |
| 5775 | 2020 | 44041 | Wein, T; Dagan, T | 2019 | The Effect of Population Bottleneck Size and Selective Regime on Genetic Diversity and Evolvability in Bacteria | Population bottlenecks leading to a drastic reduction of the population size are common in the evolutionary dynamics of natural populations; their occurrence is known to have implications for genome evolution due to genetic drift, the consequent reduction in genetic diversity, and the rate of adaptation. Nevertheless, an empirical characterization of the effect of population bottleneck sizeon evolutionary dynamics of bacteria is currently lacking. In this study, we show that selective conditions have a stronger effect on the evolutionary history of bacteria in comparison to population bottlenecks. We evolved Escherichia coli populations under three different population bottleneck sizes (small, medium, and large) in two temperature regimes (37 degrees C and 20 degrees C). We find a high genetic diversity in the large in comparison to the small bottleneck size. Nonetheless, the cold temperature led to reduced genetic diversity regardless the bottleneck size; hence, the temperature has a stronger effect on the genetic diversity in comparison to the bottleneck size. A comparison of the fitness gain among the evolved populations reveals a similar pattern where the temperature has a significant effect on the fitness. Our study demonstrates that population bottlenecks are an important determinant of bacterial evolvability; their consequences depend on the selective conditions and are best understood via their effect on the standing genetic variation. | NA | no | No good fitness measure | NA | no | no |
| 5776 | 2020 | 44111 | Weiss, E; Spicher, C; Haas, R; Fischer, W | 2019 | Excision and transfer of an integrating and conjugative element in a bacterial species with high recombination efficiency | Horizontal transfer of mobile genetic elements, such as integrating and conjugative elements (ICEs), plays an important role in generating diversity and maintaining comprehensive pan-genomes in bacterial populations. The human gastric pathogen Helicobacter pylori, which is known for its extreme genetic diversity, possesses highly efficient transformation and recombination systems to achieve this diversity, but it is unclear to what extent these systems influence ICE physiology. In this study, we have examined the excision/integration and horizontal transfer characteristics of an ICE (termed ICEHptfs4) in these bacteria. We show that transfer of ICEHptfs4 DNA during mating between donor and recipient strains is independent of its conjugation genes, and that homologous recombination is much more efficient than site-specific integration into the recipient chromosome. Nevertheless, ICEHptfs4 excision by site-specific recombination occurs permanently in a subpopulation of cells and involves relocation of a circularization-dependent promoter. Selection experiments for excision indicate that the circular form of ICEHptfs4 is not replicative, but readily reintegrates by site-specific recombination. Thus, although ICEHptfs4 harbours all essential transfer genes, and typical ICE functions such as site-specific integration are active in H. pylori, canonical ICE transfer is subordinate to the more efficient general DNA uptake and homologous recombination machineries in these bacteria. | NA | no | gd of pathogen | NA | no | no |
| 5777 | 2020 | 44112 | Weldenegodguad, M; Popov, R; Pokharel, K; Ammosov, L; Ming, Y; Ivanova, Z; Kantanen, J | 2019 | Whole-Genome Sequencing of Three Native Cattle Breeds Originating From the Northernmost Cattle Farming Regions | Northern Fennoscandia and the Sakha Republic in the Russian Federation represent the northernmost regions on Earth where cattle farming has been traditionally practiced. In this study, we performed whole-genome sequencing to genetically characterize three rare native breeds Eastern Finncattle, Western Finncattle and Yakutian cattle adapted to these northern Eurasian regions. We examined the demographic history, genetic diversity and unfolded loci under natural or artificial selection. On average, we achieved 13.01-fold genome coverage after mapping the sequencing reads on the bovine reference genome (UMD 3.1) and detected a total of 17.45 million single nucleotide polymorphisms (SNPs) and 1.95 million insertions-deletions (indels). We observed that the ancestral species (Bos primigenius) of Eurasian taurine cattle experienced two notable prehistorical declines in effective population size associated with dramatic climate changes. The modern Yakutian cattle exhibited a higher level of within-population variation in terms of number of SNPs and nucleotide diversity than the contemporary European taurine breeds. This result is in contrast to the results of marker-based cattle breed diversity studies, indicating assortment bias in previous analyses. Our results suggest that the effective population size of the ancestral Asiatic taurine cattle may have been higher than that of the European cattle. Alternatively, our findings could indicate the hybrid origins of the Yakutian cattle ancestries and possibly the lack of intensive artificial selection. We identified a number of genomic regions under selection that may have contributed to the adaptation to the northern and subarctic environments, including genes involved in disease resistance, sensory perception, cold adaptation and growth. By characterizing the native breeds, we were able to obtain new information on cattle genomes and on the value of the adapted breeds for the conservation of cattle genetic resources. | NA | no | dometicated host | NA | no | no |
| 5778 | 2020 | 44041 | Werdel, TJ; Jenks, JA; Besser, TE; Kanta, JT; Lehman, CP; Frink, TJ | 2020 | Restoration of a bighorn sheep population impeded by Mycoplasma ovipneumoniae exposure | Bighorn sheep (Ovis canadensis) were once extirpated from the Black Hills region of South Dakota, U.S.A., mirroring declining populations throughout North America. Since the 1960s, several reintroductions have occurred in the Black Hills to reestablish populations, with varying success. We translocated 26 bighorn sheep from Alberta, Canada, to the Black Hills (February 2015) to restore bighorn sheep to their historic range. Due to prior examinations of cause-specific survival, subsequent genetic diversity and disease prevalence analyses were required to evaluate success of the restoration effort. We measured a mean allelic diversity of 5.23 (SE = 0.44 [mean number of alleles]) and an observed heterozygosity of 0.71 (SE = 0.06; expected = 0.64 +/- 0.05) in the translocated individuals. Translocated bighorn sheep tested negative for Mycoplasma ovipneumoniae at capture. An autogenous vaccine was administered prior to release in an attempt to safeguard the translocated bighorn sheep from infection with a strain known to be resident in adjacent bighorn sheep populations. However, the year following the translocation, a different strain of M. ovipneumoniae was associated with a pneumonia outbreak that resulted in 57.9% mortality. Our results suggest that allelic diversity and heterozygosity were sufficient for long-term herd establishment, reducing the potential for founder effects. However, the overwhelming mortality associated with pneumonia, via the transfer of M. ovipneumoniae from an unknown source, limited the success or our reintroduction efforts. Successful attempts to restore bighorn sheep to their historic ranges must consider and mitigate potential routes for M. ovipneumoniae transmission pre- and post-reintroduction. | NA | no | No good fitness measure and measure host GD | NA | no | no |
| 5779 | 2020 | 44113 | West, R; Potter, S; Taggart, D; Eldridge, MDB | 2018 | Looking back to go forward: genetics informs future management of captive and reintroduced populations of the black-footed rock-wallaby Petrogale lateralis | Active management is essential to the survival of many threatened species globally. Captive breeding programmes can play an important role in facilitating the supplementation, translocation and reintroduction of wild populations. However, understanding the genetic dynamics within and among wild and captive populations is crucial to the planning and implementation of ex situ management, as adaptive potential is, in part, driven by genetic diversity. Here, we use 14 microsatellite loci and mitochondrial Control Region sequence to examine the population genetics of both wild populations and captive colonies of the endangered warru (the MacDonnell Ranges race of the black-footed rock-wallaby Petrogale lateralis) in central Australia, to understand how historical evolutionary processes have shaped current diversity and ensure effective ex situ management. Whilst microsatellite data reveal significant contemporary differentiation amongst remnant warru populations, evidence of contemporary dispersal and relatively weak isolation by distance, as well as a lack of phylogeographic structure suggests historical connectivity. Genetic diversity within current captive populations is lower than in the wild source populations. Based on our genetic data and ecological observations, we predict outbreeding depression is unlikely and hence make the recommendation that captive populations be managed as one genetic group. This will increase genetic diversity within the captive population and as a result increase the adaptive potential of reintroduced populations. We also identify a new site in the Musgrave Ranges which contains unique alleles but also connectivity with a population 6 km away. This novel genetic diversity could be used as a future source for supplementation. | NA | no | No good fitness measure and measure host GD | NA | no | no |
| 5780 | 2020 | 44112 | Westergaard, KB; Zemp, N; Bruederle, LP; Stenoien, HK; Widmer, A; Fior, S | 2019 | Population genomic evidence for plant glacial survival in Scandinavia | Quaternary glaciations have played a major role in shaping the genetic diversity and distribution of plant species. Strong palaeoecological and genetic evidence supports a postglacial recolonization of most plant species to northern Europe from southern, eastern and even western glacial refugia. Although highly controversial, the existence of small in situ glacial refugia in northern Europe has recently gained molecular support. We used genomic analyses to examine the phylogeography of a species that is critical in this debate. Carex scirpoidea Michx subsp. scirpoidea is a dioecious, amphi-Atlantic arctic-alpine sedge that is widely distributed in North America, but absent from most of Eurasia, apart from three extremely disjunct populations in Norway, all well within the limits of the Weichselian ice sheet. Range-wide population sampling and variation at 5,307 single nucleotide polymorphisms show that the three Norwegian populations comprise unique evolutionary lineages divergent from Greenland with high between-population divergence. The Norwegian populations have low within-population genetic diversity consistent with having experienced genetic bottlenecks in glacial refugia, and host private alleles that probably accumulated in long-term isolated populations. Demographic analyses support a single, pre-Weichselian colonization into Norway from East Greenland, and subsequent divergence of the three populations in separate refugia. Other refugial areas are identified in North-east Greenland, Minnesota/Michigan, Colorado and Alaska. Admixed populations in British Columbia and West Greenland indicate postglacial contact. Taken together, evidence from this study strongly indicates in situ glacial survival in Scandinavia. | NA | no | No good fitness measure | NA | no | no |
| 5781 | 2020 | 44111 | Whitney, KD; Mudge, J; Natvig, DO; Sundararajan, A; Pockman, WT; Bell, J; Collins, SL; Rudgers, JA | 2019 | Experimental drought reduces genetic diversity in the grassland foundation species Bouteloua eriopoda | Understanding the resistance and resilience of foundation plant species to climate change is a critical issue because the loss of these species would fundamentally reshape communities and ecosystem processes. High levels of population genetic diversity may buffer foundation species against climate disruptions, but the strong selective pressures associated with climatic shifts may also rapidly reduce such diversity. We characterized genetic diversity and its responsiveness to experimental drought in the foundation plant, black grama grass (Bouteloua eriopoda), which dominates many western North American grasslands and shrublands. Previous studies suggested that in arid ecosystems, black grama reproduces largely asexually via stolons, and thus is likely to have low genetic variability, which might limit its potential to respond to climate disruptions. Using genotyping-by-sequencing, we demonstrated unexpectedly high genetic variability among black grama plants in a 1ha site within the Sevilleta National Wildlife Refuge in central New Mexico, suggesting some level of sexual reproduction. Three years of experimental, growing season drought reduced black grama survival and biomass (the latter by 96%), with clear genetic differentiation (higher F-ST) between plantssuccumbing to drought and those remaining alive. Reduced genetic variability in the surviving plantsin drought plots indicated that the experimental drought had forced black grama populations through selection bottlenecks. These results suggest that foundation grass species, such as black grama, may experience rapid evolutionary change if future climates include more severe droughts. | NA | no | No good fitness measure and measure host GD | NA | no | no |
| 5782 | 2020 | 44041 | Whitney, TD; Gandhi, KJK; Lucardi, RD | 2019 | Native or non-native? Historical biogeography of an emergent forest pest, <it>Matsucoccus macrocicatrices</it> | Aim: A historically benign insect herbivore, Matsucoccus macrocicatrices, has recently been linked to dieback and mortality of eastern white pine (Pinus strobus L.). Previous reports indicated that its native range was restricted to New England, USA and southeastern Canada. Now, the insect occurs throughout an area extending from the putative native range, southward to Georgia, and westward to Wisconsin. Our goal was to evaluate whether its current distribution was due to recent introductions consistent with invasion processes. We considered two hypotheses: (a) if recent expansion into adventive regions occurred, those populations would have reduced genetic diversity due to founder effect(s); alternatively (b) if M. macrocicatrices is native and historically co-occurred with its host tree throughout the North American range, then populations would have greater overall genetic diversity and a population structure indicative of past biogeographical influences. Location: Eastern North America. Methods: We developed nine M. macrocicatrices-specific microsatellite markers de novo and genotyped 390 individuals from 22 populations sampled across the range of eastern white pine in the USA. We assessed genetic variability, relatedness, and population structure. Results: There were no signatures of founder effects. The only differences in genetic diversity occurred latitudinally, where the number of rare alleles and observed heterozygosity was highest in the southern range extent. Analyses of population structure indicated three distinct genetic clusters separated by the Great Lakes and the Blue Ridge Mountains. Main Conclusions: The seemingly sudden ecological shift from benign herbivore to significant pest led us to suspect that M. macrocicatrices was non-native. However, our findings suggest that this insect is native and has likely co-occurred with its host tree since the last glacial maximum. Our study demonstrates the importance of historical biogeographical reconstruction to inform how to approach an emergent pest. | NA | no | No good fitness measure and measure host GD | NA | no | no |
| 5783 | 2020 | 44112 | Whittingham, LA; Dunn, PO; Freeman-Gallant, CR; Taff, CC; Johnson, JA | 2018 | Major histocompatibility complex variation and blood parasites in resident and migratory populations of the common yellowthroat | Genes of the major histocompatibility complex (MHC) are a critical part of the adaptive immune response, and the most polymorphic genes in the vertebrate genome, especially in passerine birds. This diversity is thought to be influenced by exposure to pathogens which can vary in relation to numerous factors. Migratory behaviour may be a particularly important trait to consider because migratory birds are exposed to a greater number of different pathogens and parasites at both breeding (i.e. temperate) and overwintering (i.e. tropical and subtropical) areas, as well as at stopover sites during migration. Thus, migrants are predicted to have greater MHC diversity than residents. We compared MHC variation, at both class I and II, and levels of haemosporidian infection between one resident and two migratory populations of the common yellowthroat (Geothlypis trichas). We found that residents were less likely to be infected with haemosporidian parasites and had lower MHC diversity at class I; however, variation at MHC class II was greater in residents than migrants, contrary to our prediction. These patterns were not likely to be caused by differences in population demography as genomewide heterozygosity (based on 9225 single nucleotide polymorphisms) was high in all three populations and not correlated with MHC variation. Our different results for MHC class I and II suggest that studies of immune gene variation in relation to life history need to consider that there could be different selection pressures arising from intracellular (class I) and extracellular (class II) pathogens in different populations. | NA | no | mhc diversity | NA | no | no |
| 5784 | 2020 | 44040 | Wieczorek, K; Wolkowicz, T; Osek, J | 2020 | MLST-based genetic relatedness of Campylobacter jejuni isolated from chickens and humans in Poland | Campylobacter jejuni infection is one of the most frequently reported foodborne bacterial diseases worldwide. The main transmission route of these microorganisms to humans is consumption of contaminated food, especially of chicken origin. The aim of this study was to analyze the genetic relatedness of C. jejuni from chicken sources (feces, carcasses, and meat) and from humans with diarrhea as well as to subtype the isolates to gain better insight into their population structure present in Poland. C. jejuni were genotyped using multilocus sequence typing (MLST) and sequence types (STs) were assigned in the MLST database. Among 602 isolates tested, a total of 121 different STs, including 70 (57.9%) unique to the isolates’ origin, and 32 STs that were not present in the MLST database were identified. The most prevalent STs were ST464 and ST257, with 58 (9.6%) and 52 (8.6%) C. jejuni isolates, respectively. Isolates with some STs (464, 6411, 257, 50) were shown to be common in chickens, whereas others (e.g. ST21 and ST572) were more often identified among human C. jejuni. It was shown that of 47 human sequence types, 26 STs (106 isolates), 23 STs (102 isolates), and 29 STs (100 isolates) were also identified in chicken feces, meat, and carcasses, respectively. These results, together with the high and similar proportional similarity indexes (PSI) calculated for C. jejuni isolated from patients and chickens, may suggest that human campylobacteriosis was associated with contaminated chicken meat or meat products or other kinds of food cross-contaminated with campylobacters of chicken origin. The frequency of various sequence types identified in the present study generally reflects of the prevalence of STs in other countries which may suggest that C. jejuni with some STs have a global distribution, while other genotypes may be more restricted to certain countries. | NA | no | gd of pathogen | NA | no | no |
| 5785 | 2020 | 44042 | Wildt, D; Miller, P; Koepfli, KP; Pukazhenthi, B; Palfrey, K; Livingston, G; Beetem, D; Shurter, S; Gregory, J; Takacs, M; Snodgrass, K | 2019 | Breeding Centers, Private Ranches, and Genomics for Creating Sustainable Wildlife Populations | Human-induced changes to environments are causing species declines. Beyond preserving habitat (in situ), insurance (ex situ) populations are essential to prevent species extinctions. The Conservation Centers for Species Survival (C2S2) is leveraging space of breeding centers and private ranches to produce source populations-genetically diverse reservoirs that also support research and reintroductions. The initial focus is on four African antelopes. C2S2 has developed a program, the Source Population Alliance, that emphasizes animals living in spacious, naturalistic conditions in greater numbers than can be accommodated by urban zoos. Simulation modeling demonstrates how herds can rapidly increase population abundance and retain genetic diversity. Advances in genomics and resulting DNA data allow monitoring of genetic diversity and parentage as well as refined decision-making. This approach, neither pure in situ nor ex situ, but rather sorta situ, is an innovative way of linking public and private sector resources to ensure that endangered species survive. | NA | no | No good fitness measure and measure host GD | NA | no | no |
| 5786 | 2020 | 44113 | Williams, D; Fothergill, JL; Evans, B; Caples, J; Haldenby, S; Walshaw, MJ; Brockhurst, MA; Winstanley, C; Paterson, S | 2018 | Transmission and lineage displacement drive rapid population genomic flux in cystic fibrosis airway infections of a Pseudomonas aeruginosa epidemic strain | Pseudomonas aeruginosa chronic infections of cystic fibrosis (CF) airways are a paradigm for within-host evolution with abundant evidence for rapid evolutionary adaptation and diversification. Recently emerged transmissible strains have spread globally, with the Liverpool Epidemic Strain (LES) the most common strain infecting the UK CF population. Previously we have shown that highly divergent lineages of LES can be found within a single infection, consistent with super-infection among a cross-sectional cohort of patients. However, despite its clinical importance, little is known about the impact of transmission on the genetic structure of these infections over time. To characterize this, we longitudinally sampled a meta-population of 15 genetic lineages within the LES over 13 months among seven chronically infected CF patients by genome sequencing. Comparative genome analyses of P. aeruginosa populations revealed that the presence of coexisting lineages contributed more to genetic diversity within an infection than diversification in situ. We observed rapid and substantial shifts in the relative abundance of lineages and replacement of dominant lineages, likely to represent super-infection by repeated transmissions. Lineage dynamics within patients led to rapid changes in the frequencies of mutations across suites of linked loci carried by each lineage. Many loci were associated with important infection phenotypes such as antibiotic resistance, mucoidy and quorum sensing, and were repeatedly mutated in different lineages. These findings suggest that transmission leads to rapid shifts in the genetic structure of CF infections, including in clinically important phenotypes such as antimicrobial resistance, and is likely to impede accurate diagnosis and treatment. | NA | no | gd of pathogen | NA | no | no |
| 5787 | 2020 | 44042 | Williams, MKF; Tripodi, AD; Szalanski, AL | 2019 | Molecular survey for the honey bee (Apis mellifera L.) trypanosome parasites Crithidia mellificae and Lotmaria passim | Honey bee populations in the United States have been fluctuating in recent years. Although this has not been attributed to any one cause, recent studies have shown that multiple interactions among mircoorganisms may contribute to their decline. Several honey bee internal parasites have been overlooked as probable causes of decline; these include two different species of trypanosomes Crithidia mellificae and Lotmaria passim. Both parasites have been understudied in honey bee populations and could contribute to population disease dynamics. This study explored a molecular diagnostic survey for C. mellificae and L. passim using multiplex-PCR. Honey bee samples from both feral and managed populations were collected from eight states. This is the first national honey bee trypanosome survey in the United States. A total of 1,360 samples were surveyed during this study in which, 11% were positive for L. passim; while no cases of C. mellificae were detected using multiplex-PCR analysis. Infection rates of states positive for L. passim ranged from 17% (New York) to 0.70% (Utah). Only one state (Mississippi) was not positive for L. passim. The proportion of positive L. passim samples was significantly different between managed and feral honey bee colonies. Results revealed that the honey bee parasitic trypanosome L. passim has a widespread distribution in the United States and should be considered as a potential contributor to honey bee health decline. | NA | no | No good fitness measure and measure host GD | NA | no | no |
| 5788 | 2020 | 44040 | Winnicki, SK; Munguia, SM; Williams, EJ; Boyle, WA | 2020 | Social interactions do not drive territory aggregation in a grassland songbird | Understanding the drivers of animal distributions is a fundamental goal of ecology and informs habitat management. The costs and benefits of colonial aggregations in animals are well established, but the factors leading to aggregation in territorial animals remain unclear. Territorial animals might aggregate to facilitate social behavior such as (1) group defense from predators and/or parasites, (2) cooperative care of offspring, (3) extra-pair mating, and/or (4) mitigating costs of extra-pair mating through kin selection. Using experimental and observational methods, we tested predictions of all four hypotheses in a tallgrass prairie in northeast Kansas, United States. Grasshopper Sparrow (Ammodramus savannarum) males formed clumps of territories in some parts of the site while leaving other apparently suitable areas unoccupied. Despite substantial sampling effort (653 territories and 223 nests), we found no support for any hypothesized social driver of aggregation, nor evidence that aggregation increases nest success. Our results run counter to previous evidence that conspecific interactions shape territory distributions. These results suggest one of the following alternatives: (1) the benefits of aggregation accrue to different life-history stages, or (2) the benefits of territory aggregation may be too small to detect in short-term studies and/or the consequences of aggregation are sufficiently temporally and spatially variable that they do not always appear to be locally adaptive, perhaps exacerbated by changing landscape contexts and declining population sizes. | NA | no | No good fitness measure and measure host GD | NA | no | no |
| 5789 | 2020 | 44039 | Wohlfeil, CK; Godfrey, SS; Leu, ST; Clayton, J; Gardner, MG | 2020 | Spatial proximity and asynchronous refuge sharing networks both explain patterns of tick genetic relatedness among lizards, but in different years | A major question for understanding the ecology of parasite infections and diseases in wildlife populations concerns the transmission pathways among hosts. Network models are increasingly used to model the transmission of infections among hosts - however, few studies have integrated host behaviour and genetic relatedness of the parasites transmitted between hosts. In a study of the Australian sleepy lizard Tiliqua rugosa and its three-host ixodid tick (Bothriocroton hydrosauri), we asked if patterns of genetic relatedness among ticks were best explained by spatial proximity or the host transmission network. Using synchronous GPS locations of over 50 adult lizards at 10 min intervals across the three-month activity period, over 2 years, we developed two alternative parasite transmission networks. One alternative was based on the spatial proximity of lizards (at the centre of their home ranges), and the other was based on the frequency of asynchronous shared refuge use between pairs of lizards. In each year, adult ticks were removed from lizards and their genotypes were determined at four polymorphic microsatellite loci. Adult ticks collected from the same host were more related to each other than ticks from different hosts. Similarly, adult ticks collected from different lizards had a higher relatedness if those lizards had a shorter path length connecting them on each of the two networks we explored. The predictors of tick relatedness differed between years. In the first year, the asynchronous shared refuges network was the stronger predictor of tick relatedness, whereas in year two, the spatial proximity-based network was the stronger predictor of tick relatedness. We speculate on how changing environmental conditions might change the relative importance of alternative processes driving the transmission of parasites. | NA | no | No good fitness measure and measure host GD | NA | no | no |
| 5790 | 2020 | 44042 | Wong, CKF; Zulperi, D; Vadamalai, G; Saidi, NB; Teh, CY | 2019 | Phylogenetic Analysis of Fusarium oxysporum f. sp. cubense Associated with Fusarium Wilt of Bananas from Peninsular Malaysia | Fusarium wilt of bananas caused by Fusarium oxysporum f. sp. cubense (Foc) has been known to hamper banana production. In Malaysia, most of the edible banana cultivars are susceptible to the fungal strain Foc tropical race 4 (Foc TR4). To date, molecular characterisation of Foc TR4 in this country remains elusive .Thus, this study aimed to characterise the diversity of Foc TR4 in Peninsular Malaysia based on the phylogenetic analysis of transcription elongation factor (TEF-1 alpha) sequences. From March 2016 to December 2016, 17 isolates of Foc were isolated from nine outbreak states in Peninsular Malaysia consisting of Penang, Perak, Selangor, Negeri Sembilan, Melaka, Johor, Pahang, Kelantan and Terengganu, with disease incidence exceeding 70% in several plantations. Morphological, molecular identification using TR4 specific primers and pathogenicity assay confirmed the identity of the fungal pathogen. Pathogenicity assays indicated that the aggressiveness level varies among the isolates. Phylogenetic analyses of TEF1-alpha sequences showed that there was no genetic variation among the Foc isolates by clustering them into four vegetative compatibility groups (VCGs) of 01213 (TR4), 01216 (TR4), 01213/16 (TR4) and 0121 (R4). PCR amplification using VCG specific primers have further categorised 16 Foc isolates as VCG01213 and a single isolate as VCG01213/16. In addition, no correlation between TEF-1 alpha sequences and aggressiveness of isolates could be established. | NA | no | gd of pathogen | NA | no | no |
| 5791 | 2020 | 44113 | Wosula, EN; Chen, WB; Fei, ZJ; Legg, JP | 2017 | Unravelling the Genetic Diversity among Cassava Bemisia tabaci Whiteflies Using NextRAD Sequencing | Bemisia tabaci threatens production of cassava in Africa through vectoring viruses that cause cassava mosaic disease (CMD) and cassava brown streak disease (CBSD). B. tabaci sampled from cassava in eight countries in Africa were genotyped using NextRAD sequencing, and their phylogeny and population genetics were investigated using the resultant single nucleotide polymorphism (SNP) markers. SNP marker data and short sequences of mitochondrial DNA cytochrome oxidase I (mtCOI) obtained from the same insect were compared. Eight genetically distinct groups were identified based on mtCOI, whereas phylogenetic analysis using SNPs identified six major groups, which were further confirmed by PCA and multidimensional analyses. STRUCTURE analysis identified four ancestral B. tabaci populations that have contributed alleles to the six SNP-based groups. Significant gene flows were detected between several of the six SNP-based groups. Evidence of gene flow was strongest for SNP-based groups occurring in central Africa. Comparison of the mtCOI and SNP identities of sampled insects provided a strong indication that hybrid populations are emerging in parts of Africa recently affected by the severe CMD pandemic. This study reveals that mtCOI is not an effective marker at distinguishing cassava-colonizing B. tabaci haplogroups, and that more robust SNP-based multilocus markers should be developed. Significant gene flows between populations could lead to the emergence of haplogroups that might alter the dynamics of cassava virus spread and disease severity in Africa. Continuous monitoring of genetic compositions of whitefly populations should be an essential component in efforts to combat cassava viruses in Africa. | NA | no | gd of pathogen | NA | no | no |
| 5792 | 2020 | 44111 | Wren, CD; Burke, A | 2019 | Habitat suitability and the genetic structure of human populations during the Last Glacial Maximum (LGM) in Western Europe | Human populations in Western Europe during the Last Glacial Maximum were geographically constrained to glacial refugia by the severity of the climate and ecological risk factors. In this research we use an agent-based model of human mobility and interaction, based on ethnographic and archaeological data, to explore the impact of ecological risk on human population structure via a reconstructed landscape of habitat suitability. The agent-based model allows us to evaluate the size and location of glacial refugia, the size of the populations occupying them and the degree of genetic relatedness between people occupying these areas. To do this, we model the probability of an agent foraging groups’ survival as a function of habitat suitability. The model’s simulated genomes (composed of regionally specific genetic markers) allow us to track long-term trends of inter-regional interaction and mobility. The results agree with previous archaeological studies situating a large glacial refugium spanning southern France and northeastern Spain, but we expand on those studies by demonstrating that higher rates of population growth in this central refugium led to continuous out-migration and therefore genetic homogeneity across Western Europe, with the possible exception of the Italian peninsula. These results concur with material culture data from known archaeological sites dating to the Last Glacial Maximum and make predictions for future ancient DNA studies. | NA | no | human | NA | no | no |
| 5793 | 2020 | 44042 | Wu, XX; Xu, XF; Ma, DX; Chen, RZ; Li, TY; Cao, YY | 2019 | Virulence structure and its genetic diversity analyses of Blumeria graminis f. sp. tritici isolates in China | Background: Blumeria graminis f. sp. tritici (Bgt), the causal agent of wheat powdery mildew severely affects yield security wheat production in China. Understanding the virulence structure and genetic variations of this pathogen is important for breeding wheat lines resistant to wheat powdery mildew. However, information related to genes controlling resistance remains elusive. This study analyzes the virulence structure and the genetic diversity of pathogenic Bgt populations isolated from northeastern (Liaoning, Heilongjiang) and northwestern (Gansu) China, two representative wheat producing areas, on 37 wheat cultivars each carrying a known powdery mildew resistance (Pm) gene. Results: Bgt isolates from northeastern China show higher frequencies of virulence genes than populations from Gansu Province. Many of the known Pm genes failed to provide resistance in this study. However, Pm21 provided 100% resistance to all isolates from all three provinces, obtained during two consecutive years, while Pm13 provided 100% resistance in Gansu. Pm13, Pm16, Pm18, and Pm22 also showed partial resistance in northeastern China, while Pm16, Pm18, Pm22, Pm5 + 6 and Pm2 + 6 +? maintained some resistance in Gansu. Genetic diversity among populations in different regions was detected by cluster analyses using expressed sequence tag-simple sequence repeat (EST-SSR). When the genetic similarity coefficient is relatively high, populations from the same regional origin are mostly clustered into one group while populations from different regions exhibit large genetic differences. Conclusion: Pm21 remains the best choice for breeding programs to maintain resistance to Bgt. Only 58% of the isolates tested show a clear correlation between EST-SSR genetic polymorphisms and frequency of virulence gene data. | NA | no | gd of pathogen | NA | no | no |
| 5794 | 2020 | 44112 | Wurster, JI; Bispo, PJM; Van Tyne, D; Cadorette, JJ; Boody, R; Gilmore, MS | 2018 | Staphylococcus aureus from ocular and otolaryngology infections are frequently resistant to clinically important antibiotics and are associated with lineages of community and hospital origins | Staphylococcus aureus is an important human pathogen that causes serious antibiotic-resistant infections. Its population structure is marked by the appearance and dissemination of successful lineages across different settings. To begin understanding the population structure of S. aureus causing ocular and otolaryngology infections, we characterized 262 isolates by antimicrobial sensitivity testing and multilocus sequence typing (MLST). Methicillin-resistant S. aureus were subjected to SCCmec typing and Panton-Valentine leukocidin (PVL) screening. Although we detected a high level of genetic diversity among methicillin-sensitive (MSSA) isolates, (63 sequence types-STs), the population was dominated by five lineages: ST30, ST5, ST8, ST15 and ST97. Resistance to penicillin, erythromycin and clindamycin was common among the major MSSA lineages, with fluctuations markedly impacted by genetic background. Isolates belonging to the predominant lineage, ST30, displayed high rates of resistance to penicillin (100%), erythromycin (71%), and clindamycin (63%). Overall, 21% of the isolates were methicillin-resistant (MRSA), with an apparent enrichment among otitis and orbital cellulitis isolates (>40%). MRSA isolates belonged to 14 STs grouped in 5 clonal complexes (CC), however, CC5 (56.1%) and CC8 (38.6%) dominated the population. Most CC5 strains were SCCmec type II, and resembled the hospital-adapted USA100 clone. CC8 strains were SCCmec type IV, and 86% were positive for the PVL toxin, common features of the community-acquired clone USA300. CC5 strains harboring a SCCmec type IV, typical for the USA800 clone, comprised 15.5% of the population. USA100 strains were highly resistant to clindamycin, erythromycin and levofloxacin (100%), while USA300 strains were frequently resistant to erythromycin (89%) but displayed lower rates of resistance to levofloxacin (39%) and clindamycin (17%). Our data demonstrate that the ocular and otolaryngology S. aureus populations are composed of strains that are commonly resistant to clinically relevant antibiotics, and are associated with the major epidemic clonal complexes of both community and hospital origins. | NA | no | gd of pathogen | NA | no | no |
| 5795 | 2020 | 44113 | Wymant, C; Hall, M; Ratmann, O; Bonsall, D; Golubchik, T; de Cesare, M; Gall, A; Cornelissen, M; Fraser, C | 2018 | PHYLOSCANNER: Inferring Transmission from Within- and Between-Host Pathogen Genetic Diversity | A central feature of pathogen genomics is that different infectious particles (virions and bacterial cells) within an infected individual may be genetically distinct, with patterns of relatedness among infectious particles being the result of both within-host evolution and transmission from one host to the next. Here, we present a new software tool, phyloscanner, which analyses pathogen diversity from multiple infected hosts. phyloscanner provides unprecedented resolution into the transmission process, allowing inference of the direction of transmission from sequence data alone. Multiply infected individuals are also identified, as they harbor subpopulations of infectious particles that are not connected by within-host evolution, except where recombinant types emerge. Low-level contamination is flagged and removed. We illustrate phyloscanner on both viral and bacterial pathogens, namely HIV-1 sequenced on Illumina and Roche 454 platforms, HCV sequenced with the Oxford Nanopore MinION platform, and Streptococcus pneumoniae with sequences from multiple colonies per individual. phyloscanner is available from https://github.com/BDI-pathogens/phyloscanner. | NA | no | No good fitness measure and measure host GD | NA | no | no |
| 5796 | 2020 | 44040 | Xu, ML; Li, XZ; Mo, X; Tu, SY; Cui, YH; Yang, DH | 2020 | Studies on the cold tolerance of ratoon ‘Chaling’ common wild rice | Background Rice is the staple food of many people around the world. However, most rice varieties, especially widely grown indica varieties and hybrids, are sensitive to cold stress. In order to provide a basis for the utilization of a common wild rice (CWR, Oryza rufipogon Griff.) named ‘Chaling’ CWR in cold-tolerant rice breeding and deepen the understanding of rice cold tolerance, the cold tolerance of ratoon ‘Chaling’ CWR was studied under the stress of the natural low temperature in winter in Changsha, Hunan province, China, especially under the stress of abnormal natural low temperature in Changsha in 2008, taking other ratoon CWR accessions and ratoon cultivated rice phenotypes as control. Results The results showed that ratoon ‘Chaling’ CWR can safely overwinter under the natural conditions in Changsha (28 degrees 22 ’ N), Hunan province, China, which is a further and colder northern place than its habitat, even if it suffers a long-term low temperature stress with ice and snow. In 2008, an extremely cold winter appeared in Changsha, i.e., the average daily mean temperature of 22 consecutive days from January 13 to February 3 was - 1.0 degrees C, and the extreme low temperature was - 4.7 degrees C. After subjected to this long-term cold stress, the overwinter survival rate of ratoon ‘Chaling’ CWR was 100%, equals to that of ratoon ‘Dongxiang’ CWR which is northernmost distribution in the word among wild rice populations, higher than those of ratoon ‘Fusui’ CWR, ratoon ‘Jiangyong’ CWR, and ratoon ‘Liujiang’ CWR (63.55-83.5%) as well as those of ratoon ‘Hainan’ CWR, ratoon ‘Hepu’ CWR, and all the ratoon cultivated rice phenotypes including 3 japonica ones, 3 javanica ones, and 5 indica ones (0.0%). Conclusions The results indicate that ratoon ‘Chaling’ CWR possesses strong cold tolerance and certain freezing tolerance. | NA | no | No good fitness measure and measure host GD | NA | no | no |
| 5797 | 2020 | 44039 | Xu, W; Zhou, XP; Fang, WZ; Chen, XL | 2020 | Genetic diversity of toll-like receptor genes in the vulnerable Chinese egret (Egretta eulophotes) | Toll-like receptor (TLR) genes have recently been employed to assess genetic diversity, as they can be used to infer both demographic history and adaptation to environments with different pathogen pressure. Here, we sampled 120 individuals of the Chinese egret (Egretta eulophotes), a globally vulnerable species, from four breeding populations across China. We assessed the levels of genetic diversity, selection pressure, and population differentiation at seven TLR loci (TLR1LB, TLR2A, TLR3, TLR4, TLR5, TLR7, and TLR15). Using a variety of metrics (SNPs, heterozygosity, nucleotides, haplotypes), our analyses showed that genetic diversity was lower at 4 of the 7 TLR loci in the vulnerable Chinese egret compared to the more common little egret (Egretta garzetta). The selection test indicated TLRs, except for TLR5, were under purifying selection in TLR evolution, suggesting that low TLR genetic diversity in the Chinese egret may be caused by purifying selection. Moreover, analysis of molecular variance indicated low but significant population differentiation among four populations at all of the TLR loci in this egret. However, some comparisons based on fixation index analyses did not show significant population differentiation, and Bayesian clustering showed admixture. Our finding suggested that these four populations of the Chinese egret in China may be considered a single unit for conservation planning. These results, the new report of TLR genetic diversity in a long-distance migratory vulnerable Ardeid species, will provide fundamental TLR information for further studies on the conservation genetics of the Chinese egret and other Ardeids. | NA | no | No good fitness measure | NA | no | no |
| 5798 | 2020 | 44111 | Xu, Z; Sun, H; Zhang, Z; Zhao, QB; Olasege, BS; Li, QM; Yue, Y; Ma, PP; Zhang, XZ; Wang, QS; Pan, YC | 2019 | Assessment of Autozygosity Derived From Runs of Homozygosity in Jinhua Pigs Disclosed by Sequencing Data | Jinhua pig, a well-known Chinese indigenous breed, has evolved as a pig breed with excellent meat quality, greater disease resistance, and higher prolificacy. The reduction in the number of Jinhua pigs over the past years has raised concerns about inbreeding. Runs of homozygosity (ROH) along the genome have been applied to quantify individual autozygosity to improve the understanding of inbreeding depression and identify genes associated with traits of interest. Here, we investigated the occurrence and distribution of ROH using next-generation sequencing data to characterize autozygosity in 202 Jinhua pigs, as well as to identify the genomic regions with high ROH frequencies within individuals. The average inbreeding coefficient, based on ROH longer than 1 Mb, was 0.168 +/- 0.052. In total, 18,690 ROH were identified in all individuals, among which shorter segments (1-5 Mb) predominated. Individual ROH autosome coverage ranged from 5.32 to 29.14% in the Jinhua population. On average, approximately 16.8% of the whole genome was covered by ROH segments, with the lowest coverage on SSC11 and the highest coverage on SSC17. A total of 824 SNPs (about 0.5%) and 11 ROH island regions were identified (occurring in over 45% of the samples). Genes associated with reproduction (HOXA3, HOXA7, HOXA10, and HOXA11), meat quality (MYOD1, LPIN3, and CTNNBL1), appetite (NUCB2) and disease resistance traits (MUC4, MUC13, MUC20, LMLN, ITGB5, HEG1, SLC12A8, and MYLK) were identified in ROH islands. Moreover, several quantitative trait loci for ham weight and ham fat thickness were detected. Genes in ROH islands suggested, at least partially, a selection for economic traits and environmental adaptation, and should be subject of future investigation. These findings contribute to the understanding of the effects of environmental and artificial selection in shaping the distribution of functional variants in the pig genome. | NA | no | dometicated host | NA | no | no |
| 5799 | 2020 | 44043 | Yadav, MK; Aravindan, S; Ngangkham, U; Prabhukarthikeyan, SR; Keerthana, U; Raghu, S; Pramesh, D; Banerjee, A; Roy, S; Sanghamitra, P; Adak, T; Priyadarshinee, P; Jena, M; Kar, MK; Rath, PC | 2019 | Candidate screening of blast resistance donors for rice breeding | Rice blast is one of the most serious diseases in the world. The use of resistant cultivars is the most preferred means to control this disease. Resistance often breaks down due to emergence of new races; hence identification of novel resistance donors is indispensable. In this study, a panel of 80 released varieties from National Rice Research Institute, Cuttack was genotyped with 36 molecular markers that were linked to 36 different blast resistance genes, to investigate the varietal genetic diversity and molecular marker-trait association with blast resistance. The polymorphism information content of 36 loci varied from 0.11 to 0.37 with an average of 0.34. The cluster analysis and population structure categorized the 80 National Rice Research Institute released varieties (NRVs) into three major genetic groups. The principal co-ordinate analysis displays the distribution of resistant and moderately resistant NRVs into different groups. Analysis of molecular variance result demonstrated maximum (97%) diversity within populations and minimum (3%) diversity between populations. Among tested markers, two markers (RM7364 and pi21\_79-3) corresponding to the blast resistance genes (Pi56(t) and pi21) were significantly associated and explained a phenotypic variance of 4.9 to 5.1% with the blast resistance. These associated genes could be introgressed through marker-assisted to develop durable blast resistant rice varieties. The selected resistant NRVs could be good donors for the blast resistance in rice crop improvement research. | NA | no | dometicated host | NA | no | no |
| 5800 | 2020 | 44112 | Yadav, MK; Aravindan, S; Ngangkham, U; Raghu, S; Prabhukarthikeyan, SR; Keerthana, U; Marndi, BC; Adak, T; Munda, S; Deshmukh, R; Pramesh, D; Samantaray, S; Rath, PC | 2019 | Blast resistance in Indian rice landraces: Genetic dissection by gene specific markers | Understanding of genetic diversity is important to explore existing gene in any crop breeding program. Most of the diversity preserved in the landraces which are well known reservoirs of important traits for biotic and abiotic stresses. In the present study, the genetic diversity at twenty-four most significant blast resistance gene loci using twenty-eight gene specific markers were investigated in landraces originated from nine diverse rice ecologies of India. Based on phenotypic evaluation, landraces were classified into three distinct groups: highly resistant (21), moderately resistant (70) and susceptible (70). The landraces harbour a range of five to nineteen genes representing blast resistance allele with the frequency varied from 4.96% to 100%. The cluster analysis grouped entire 161 landraces into two major groups. Population structure along with other parameters was also analyzed to understand the evolution of blast resistance gene in rice. The population structure analysis and principal coordinate analysis classified the landraces into two sub populations. Analysis of molecular variance showed maximum (93%) diversity within the population and least (7%) between populations. Five markers viz; K3957, Pikh, Pi2-i, RM212and RM302 were strongly associated with blast disease with the phenotypic variance of 1.4% to 7.6%. These resistant land races will serve as a valuable genetic resource for future genomic studies, host pathogen interaction, identification of novel R genes and rice improvement strategies. | NA | no | dometicated host | NA | no | no |
| 5801 | 2020 | 44040 | Yair, Y; Borovok, I; Mikula, I; Falk, R; Fox, LK; Gophna, U; Lysnyansky, I | 2020 | Genomics-based epidemiology of bovine Mycoplasma bovis strains in Israel | Background Mycoplasma bovis is an important etiologic agent of bovine mycoplasmosis affecting cattle production and animal welfare. In the past in Israel, M. bovis has been most frequently associated with bovine respiratory disease (BRD) and was rarely isolated from mastitis. This situation changed in 2008 when M. bovis-associated mastitis emerged in Israel. The aim of this study was to utilize whole genome sequencing to evaluate the molecular epidemiology and genomic diversity of M. bovis mastitis-associated strains and their genetic relatedness to M. bovis strains isolated from BRD in local feedlot calves and those imported to Israel from different European countries and Australia. Results Phylogeny based on total single nucleotide polymorphism (SNP) analysis of 225 M. bovis genomes clearly showed clustering of isolates on the basis of geographical origin: strains isolated from European countries clustered together and separately from Australian and Chinese isolates, while Israeli isolates were found in the both groups. The dominant genotype was identified among local mastitis-associated M. bovis isolates. This genotype showed a close genomic relatedness to M. bovis strains isolated from calves imported to Israel from Australia, to original Australian M. bovis strains, as well as to strains isolated in China. Conclusions This study represents the first comprehensive high-resolution genome-based epidemiological analysis of M. bovis in Israel and illustrates the possible dissemination of the pathogen across the globe by cattle trade. | NA | no | gd of pathogen | NA | no | no |
| 5802 | 2020 | 44041 | Yamamoto, N; Wang, YR; Lin, RM; Liang, YY; Liu, Y; Zhu, J; Wang, LX; Wang, SQ; Liu, HN; Deng, QM; Li, SC; Li, P; Zheng, AP | 2019 | Integrative transcriptome analysis discloses the molecular basis of a heterogeneous fungal phytopathogen complex, Rhizoctonia solani AG-1 subgroups | Rhizoctonia solani is a fungal species complex that causes necrotrophic crop diseases. It comprises several anastomosis groups, some of which include intra-subgroups, such as AG-1 IA and AG-1 IB, exhibiting varying pathogenicity. Owing to its heterozygous and multinucleate features, genomic analyses of R. solani are still challenging, and understanding of its genetic diversity and genic components is limited. In this study, in order to elucidate the molecular basis of this phytopathogen complex, an integrated transcriptome analysis was undertaken for three subgroups of AG-1, i.e. AG-1 IA, AG-1 IB, and AG-1 IC. Sequence variations suggested substantial evolutionary distances within AG-1. Transcript simple sequence repeats showed comparable characteristics among AG-1, but contained polymorphic sites. Intra-subgroup polymorphisms suggested varying genic heterozygosity within AG-1, suggesting their independent evolutionary trajectory. Sequences of pathogenic factors, phytotoxin biosynthesis pathway enzymes, secreted lignocellulosic enzymes, secreted reactive oxygen species detoxification enzymes, apoplastic/cytoplasmic effector candidates, were conserved among those subgroups. d(N)/d(S) ratios of a secretome subset suggested core secreted proteins in AG-1 and distinct evolution of Cys-rich small secreted proteins after differentiation of AG-1 subgroups. Identification of likely pathogenic factors including allergen protein homologues, oxidative phosphorylation and ethylene biosynthesis pathways, and diversification of polysaccharide monooxygenases provides molecular insight into key genomic components that play a role in R. solani pathogenesis. | NA | no | No good fitness measure and measure host GD | NA | no | no |
| 5803 | 2020 | 44112 | Yang, ML; Han, NY; Li, H; Meng, LH | 2018 | Transcriptome Analysis and Microsatellite Markers Development of a Traditional Chinese Medicinal Herb Halenia elliptica D. Don (Gentianaceae) | Halenia elliptica is a popular Chinese medicinal herb that is used to treat jaundice disease and virus hepatitis. and its wild populations have been reduced significantly due to overharvesting recently. However, effective conservation could not be implemented because of the lack of genomic information and genetic markers. In this study. a de novo transcriptome of H elliptica was sequenced using the NGS Illumina, and 132695 unigenes with the length >200 bp (base pairs) were obtained. Among them, a total of 32109 unigenes were scanned to develop simple sequence repeats (SSRs). Based on NCBI (National Center for Biotechnology Information) nonredundant database (Nr), these SSR sequences were annotated and assigned into gene ontology categories. In addition, we designed 126 pairs of SSR primers for polymerase chain reaction amplification, of which 12 pairs were identified to be polymorphic among 40 individuals from 8 populations. We then used the 12 polymorphic SSRs to construct a UPGMA dendrogram of the 40 individuals. In addition, a significant correlation between the genetic relationship and the geographic distance was found, suggesting a phylogeographic structure in H Moreover, 2 of these SSRs were also successfully amplified in a related species Veratrilla baillonii, suggesting their cross-species transferability. Generally, the SSR markers with high polymorphisms identified in this study provide valuable genetic resources and represent an initial step for exploring the genetic diversity and population histories of H elliptica and its related species. | NA | no | No good fitness measure and measure host GD | NA | no | no |
| 5804 | 2020 | 44042 | Yangui, I; Boutiti, MZ; Vettraino, AM; Bruni, N; Vannini, A; Ben Jamaa, ML; Boussaid, M; Messaoud, C | 2019 | Biscogniauxia mediterranea associated with cork oak (Quercus suber) in Tunisia: Relationships between phenotypic variation, genetic diversity and ecological factors | Biscogniauxia mediterranea is a xylariaceous ascomycete responsible for ‘charcoal disease’, the most frequent disease of cork oak in the Mediterranean basin. However, little is known regarding this fungus in North Africa. This is the first report on the morphological and genetic diversity of B. mediterranea populations in Tunisia. A total of forty-eight isolates from cork oak trees, corresponding to three different sites and three tree parts, were identified as B. mediterranea based on morphological and molecular diagnosis. Variability among the isolates was evaluated using morphological traits and microsatellite-primed PCR (MSP-PCR). B. mediterranea isolates showed high variability in culture, specifically in colony color. A high level of genetic diversity within populations and within tree parts was detected (H = 0.417, 1 = 0.605, and H = 0.415, 1=0.601 respectively). Significant genetic differentiation among populations (G(ST) = 0.116 and Phi(ST) = 0.137; p < 0.001) was determined. However, a low genetic differentiation among tree parts (G(ST) = 0.066 and (1) Phi(ST) = 0.018: p = 0.07) was observed. Principal coordinate analysis showed that genetic divergence was partially related to the geographical origin. Cluster analysis based on morphological traits showed that the grouping of the isolates occurs independently of the geographic location and the tree part. The effective number of alleles (Ne), the Nei’s gene diversity index (H), the Shannon’s information index (I), the colony color and the presence of exudates were significantly correlated with the altitude and the rainfall. The number of alleles (Na), the percentage of polymorphic loci (PPL), the texture and the density of the mycelium were significantly correlated with the temperature. These results provide a deeper understanding of population genetics of B. mediterranea and its adaptation to environmental conditions which could help to develop control strategies against charcoal canker disease. (C) 2019 Elsevier Ltd and British Mycological Society. All rights reserved. | NA | no | gd of pathogen | NA | no | no |
| 5805 | 2020 | 44113 | Yano, H; Iwamoto, T; Nishiuchi, Y; Nakajima, C; Starkova, DA; Mokrousov, I; Narvskaya, O; Yoshida, S; Arikawa, K; Nakanishi, N; Osaki, K; Nakagawa, I; Ato, M; Suzuki, Y; Maruyama, F | 2017 | Population Structure and Local Adaptation of MAC Lung Disease Agent Mycobacterium avium subsp hominissuis | Mycobacterium avium subsp. hominissuis (MAH) is one of the most common nontuberculous mycobacterial species responsible for chronic lung disease in humans. Despite increasing worldwide incidence, little is known about the genetic mechanisms behind the population evolution of MAH. To elucidate the local adaptation mechanisms of MAH, we assessed genetic population structure, the mutual homologous recombination, and gene content for 36 global MAH isolates, including 12 Japanese isolates sequenced in the present study. We identified five major MAH lineages and found that extensive mutual homologous recombination occurs among them. Two lineages (MahEastAsia1 and MahEastAsia2) were predominant in the Japanese isolates. We identified alleles unique to these two East Asian lineages in the loci responsible for trehalose biosynthesis (treS and mak) and in one mammalian cell entry operon, which presumably originated from as yet undiscovered mycobacterial lineages. Several genes and alleles unique to East Asian strains were located in the fragments introduced via recombination between East Asian lineages, suggesting implication of recombination in local adaptation. These patterns of MAH genomes are consistent with the signature of distribution conjugative transfer, a mode of sexual reproduction reported for other mycobacterial species. | NA | no | No good fitness measure and measure host GD | NA | no | no |
| 5806 | 2020 | 44042 | Yano, H; Suzuki, H; Maruyama, F; Iwamoto, T | 2019 | The recombination-cold region as an epidemiological marker of recombinogenic opportunistic pathogen Mycobacterium avium | Background The rapid identification of lineage remains a challenge in the genotyping of clinical isolates of recombinogenic pathogens. The chromosome of Mycobacterium avium subsp. hominissuis (MAH), an agent of Mycobacterium avium complex (MAC) lung disease, is often mosaic and is composed of chromosomal segments originating from different lineages. This makes it difficult to infer the MAH lineage in a simple experimental set-up. To overcome this difficulty, we sought to identify chromosomal marker genes containing lineage-specific alleles by genome data mining. Results We conducted genetic population structure analysis, phylogenetic analysis, and a survey of historical recombination using data from 125 global MAH isolates. Six MAH lineages (EA1, EA2, SC1, SC2, SC3, and SC4) were identified in the current dataset. One P-450 gene (locus\_tag MAH\_0788/MAV\_0940) in the recombination-cold region was found to have multiple alleles that could discriminate five lineages. By combining the information about allele type from one additional gene, the six MAH lineages as well as other M. avium subspecies were distinguishable. A recombination-cold region of 116 kb contains an insertion hotspot and is flanked by a mammalian cell-entry protein operon where allelic variants have previously been reported to occur. Hence, we speculate that the acquisition of lineage- or strain-specific insertions has introduced homology breaks in the chromosome, thereby reducing the chance of interlineage recombination. Conclusions The allele types of the newly identified marker genes can be used to predict major lineages of M. avium. The single nucleotide polymorphism typing approach targeting multiallelic loci in recombination-cold regions will facilitate the epidemiological study of MAC, and may also be useful for equivalent studies of other nontuberculous mycobacteria potentially carrying mosaic genomes. | NA | no | No good fitness measure and measure host GD | NA | no | no |
| 5807 | 2020 | 44042 | Ye, R; Tian, YN; Huang, YF; Zhang, YL; Wang, J; Sun, XD; Zhou, HN; Zhang, DM; Pan, WQ | 2019 | Genome-Wide Analysis of Genetic Diversity in Plasmodium falciparum Isolates From China-Myanmar Border | Plasmodium falciparum isolates from China-Myanmar border (CMB) have experienced regional special selective pressures and adaptive evolution. However, the genomes of P. falciparum isolates from this region to date are poorly characterized. Herein, we performed whole-genome sequencing of 34 P. falciparum isolates from CMB and a series of genome-wide sequence analyses to reveal their genetic diversity, population structures, and comparisons with the isolates from other epidemic regions (Thai-Cambodia border, Thai-Myanmar border, and West Africa). Totally 59,720 high-quality single-nucleotide polymorphisms (SNPs) were identified in the P. falciparum isolates from CMB, with average nucleotide diversity (p = 4.59 x 10^-4^) and LD decay (132 bp). The Tajima’s D and Fu and Li’s D values of the CMB isolates were -0.8 (p < 0.05) and -0.84 (p < 0.05), respectively, suggesting a demographic history of recent population expansion or purifying selection. Moreover, 78 genes of the parasite were identified that could be under positive selection, including those genes conferring drug resistance such as pfubp1. In addition, 33 SNPs were identified for tracing the source of the parasites with a high accuracy by analysis of the most differential SNPs among the four epidemic regions. Collectively, our data demonstrated high diversity of the CMB isolates’ genomes forming a distinct population, and the identification of 33-SNP barcode provides a valuable surveillance of parasite migration among the regions. | NA | no | No good fitness measure and measure host GD | NA | no | no |
| 5808 | 2020 | 44113 | Yin, FY; Liu, ZJ; Liu, JL; Liu, AH; Salih, DA; Li, YQ; Liu, GY; Luol, JX; Guan, GQ; Yin, H | 2018 | Population Genetic Analysis of Theileria annulata from Six Geographical Regions in China, Determined on the Basis of Micro- and Mini-satellite Markers | Theileria annulata, a tick-borne apicomplexan protozoan, causes a lymphoproliferative disease of cattle with high prevalence in tropical and sub-tropical regions. Understanding the genetic diversity and structure of local populations will provide more fundamental knowledge for the population genetics and epidemics of protozoa. In this study, 78 samples of T. annulata collected from cattle/yaks representing 6 different geographic populations in China were genotyped using eight micro- and mini-satellite markers. High genetic variation within population, moderate genetic differentiation, and high level of diversity co-occurring with significant linkage disequilibrium were observed, which indicates there is gene flow between these populations in spite of the existence of reproductive and geographical barriers among populations. Furthermore, some degree of genetic differentiation was also found between samples from China and Oman. These findings provide a first glimpse of the genetic diversity of the T. annulata populations in China, and might contribute to the knowledge of distribution, dynamics, and epidemiology of T. annulata populations and optimize the management strategies for control. | NA | no | gd of pathogen | NA | no | no |
| 5809 | 2020 | 44111 | Yin, JW; Zhu, DJ; Li, Y; Lv, D; Yu, HJ; Liang, CM; Luo, XD; Xu, XS; Fu, JW; Yan, HF; Dai, Z; Zhou, X; Wen, X; Xiong, SS; Lin, ZX; Lin, JD; Zhao, B; Wang, YJ; Li, KS; Ma, GD | 2019 | Genetic Variability of TCF4 in Schizophrenia of Southern Chinese Han Population: A Case-Control Study | Objective: Schizophrenia is thought to be a neurodevelopmental disorder. As a key regulator in the development of the central nervous system, transcription factor 4 (TCF4) has been shown to be involved in the pathogenesis of schizophrenia. The aim of our study was to assay the association of TCF4 single nucleotide polymorphisms (SNPs) with schizophrenia and the effect of these SNPs on phenotypic variability in schizophrenia in Southern Chinese Han Population. Methods: Four SNPs (rs9960767, rs2958182, rs4309482, and rs12966547) of TCF4 were genotyped in 1137 schizophrenic patients and 1035 controls in a Southern Chinese Han population using the improved multiplex ligation detection reaction (iMLDR) technique. For patients with schizophrenia, the severity of symptom phenotypes was analyzed by the five-factor model of the Positive and Negative Symptom Scale (PANSS). Cognitive function was assessed using the Brief Assessment of Cognition in Schizophrenia (BACS) scale. Results: The results showed that the genotypes and alleles of the three SNPs (rs2958182, rs4309482, and rs12966547) were not significantly different between the control group and the case group (all P > 0.05). rs9960767 could not be included in the statistics for the extremely low minor allele frequency. However, the genotypes of rs4309482 shown a potential risk in the positive symptoms (P = 0.04) and excitement symptoms (P = 0.04) of the five-factor model of PANSS, but not survived in multiple test correction. The same potential risk was shown in the rs12966547 in positive symptoms of the PANSS (P = 0.03). Conclusion: Our results failed to find the associations of SNPs (rs2958182, rs4309482, and rs12966547) in TCF4 with schizophrenia in Southern Chinese Han Population. | NA | no | human | NA | no | no |
| 5810 | 2020 | 44041 | Yordy, J; Kraus, C; Hayward, JJ; White, ME; Shannon, LM; Creevy, KE; Promislow, DEL; Boyko, AR | 2020 | Body size, inbreeding, and lifespan in domestic dogs | Inbreeding poses a real or potential threat to nearly every species of conservation concern. Inbreeding leads to loss of diversity at the individual level, which can cause inbreeding depression, and at the population level, which can hinder ability to respond to a changing environment. In closed populations such as endangered species and ex situ breeding programs, some degree of inbreeding is inevitable. It is therefore vital to understand how different patterns of breeding and inbreeding can affect fitness in real animals. Domestic dogs provide an excellent model, showing dramatic variation in degree of inbreeding and in lifespan, an important aspect of fitness that is known to be impacted by inbreeding in other species. There is a strong negative correlation between body size and lifespan in dogs, but it is unknown whether the higher rate of aging in large dogs is due to body size per se or some other factor associated with large size. We used dense genome-wide SNP array data to calculate average inbreeding for over 100 dog breeds based on autozygous segment length and found that large breeds tend to have higher coefficients of inbreeding than small breeds. We then used data from the Veterinary medical Database and other published sources to estimate life expectancies for pure and mixed breed dogs. When controlling for size, variation in inbreeding was not associated with life expectancy across breeds. When comparing mixed versus purebred dogs, however, mixed breed dogs lived about 1.2 years longer on average than size-matched purebred dogs. Furthermore, individual pedigree coefficients of inbreeding and lifespans for over 9000 golden retrievers showed that inbreeding does negatively impact lifespan at the individual level. Registration data from the American Kennel Club suggest that the molecular inbreeding patterns observed in purebred dogs result from specific breeding practices and/or founder effects and not the current population size. Our results suggest that recent inbreeding, as reflected in variation within a breed, is more likely to affect fitness than historic inbreeding, as reflected in variation among breeds. Our results also indicate that occasional outcrosses, as in mixed breed dogs, can have a substantial positive effect on fitness. | NA | no | dometicated host | NA | no | no |
| 5811 | 2020 | 44112 | Yu, LJ; Nie, YG; Yan, L; Hu, YB; Wei, FW | 2018 | No evidence for MHC-based mate choice in wild giant pandas | Major histocompatibility complex genes (MHC), a gene cluster that controls the immune response to parasites, are regarded as an important determinant of mate choice. However, MHC-based mate choice studies are especially rare for endangered animals. The giant panda (Ailuropoda melanoleuca), a flagship species, has suffered habitat loss and fragmentation. We investigated the genetic variation of three MHC class II loci, including DRB1, DQA1, and DQA2, for 19 mating-pairs and 11 parent-pairs of wild giant pandas based on long-term field behavior observations and genetic samples. We tested four hypotheses of mate choice based on this MHC variation. We found no supporting evidence for the MHC-based heterosis, genetic diversity, genetic compatibility and good gene hypotheses. These results suggest that giant pandas may not use MHC-based signals to select mating partners, probably because limited mating opportunities or female-biased natal dispersal restricts selection for MHC-based mate choice, acknowledging the caveat of the small sample size often encountered in endangered animal studies. Our study provides insight into the mate choice mechanisms of wild giant pandas and highlights the need to increase the connectivity and facilitate dispersal among fragmented populations and habitats. | NA | no | No good fitness measure and measure host GD | NA | no | no |
| 5812 | 2020 | 44042 | Zare, S; Derakhshandeh, A; Haghkhah, M; Naziri, Z; Broujeni, AM | 2019 | Molecular typing of Staphylococcus aureus from different sources by RAPD-PCR analysis | Staphylococcus aureus is an opportunistic bacterium which is carried as a normal flora organism but has a major role in the epidemiology and pathogenesis of different staphylococcal infections in humans and animals. Fifty S. aureus isolated from banknotes, foods, human infections and bovine mastitis were subjected to DNA fingerprinting by randomly amplified polymorphic DNA (RAPD) analysis to examine their genotypic polymorphism and investigate the amount of genetic relatedness among these various isolates. At 100% RAPD profile similarity level, isolates were classified into four, five and seven groups of the same clone, according to the RAPDPCR with OLP6, OLP11 and OLP13 primers, respectively. Amplification of the isolates resulted in several polymorphic bands ranged from >50 to >1500 bp in size. Maximum number of bands was obtained by primer OLP13 which produced seven bands in bovine mastitis isolates. Most polymorphisms were observed in isolates of bovine mastitis and the lowest were associated with human infections isolates. There was no relationship between the RAPD patterns and the sources of isolates, except the three clusters which showed host specificity and only included the strains from the same sources. The results confirm the wide genotypic diversity of the studied S. aureus strains. RAPD-PCR technique can be a valuable tool for assessing the genetic relationship, detection of polymorphism in S. aureus and tracing the sources and management of S. aureus infections. | NA | no | No good fitness measure and measure host GD | NA | no | no |
| 5813 | 2020 | 44113 | Zaviezo, T; Retamal, R; Urvois, T; Fauvergue, X; Blin, A; Malausa, T | 2018 | Effects of inbreeding on a gregarious parasitoid wasp with complementary sex determination | Inbreeding and inbreeding depression are processes in small populations of particular interest for a range of human activities such as animal breeding, species conservation, or pest management. In particular, biological control programs should benefit from a thorough understanding of the causes and consequences of inbreeding because natural enemies experience repetitive bottlenecks during importation, laboratory rearing, and introduction. Predicting the effect of inbreeding in hymenopteran parasitoid wasps, frequently used in biological control programs, is nonetheless a difficult endeavor. In haplodiploid parasitoids, the purge of deleterious alleles via haploid males should reduce genetic load, but if these species also have complementary sex determination (CSD), abnormal diploid males will be produced, which may jeopardize the success of biological control introductions. Mastrus ridens is such a parasitoid wasp with CSD, introduced to control the codling moth, Cydia pomonella (L.). We studied its life history traits in the laboratory under two conditions: inbred (full-sib) and outbred (nonsib) crosses, across five generations, to examine the consequences of inbreeding in this species. We found that in inbred lines, nonreproducing females live less, the number of daughters produced was lower, and sex ratio (proportion of males) and proportion of diploid males were higher. Diploid males were able to produce fertile daughters, but fewer than haploid males. Lineage survival was similar for inbred and outbred lines across the five generations. The most significant decrease in fitness was thus a consequence of the production of diploid males, but this effect was not as extreme as in most other species with CSD, due to the fertility of diploid males. This study highlights the importance of determining the type of sex determination in parasitoid wasps used for biological control, and the importance of maintaining genetic diversity in species with CSD when importation or augmentation is the goal. | NA | no | inbreeding | NA | no | no |
| 5814 | 2020 | 44113 | Zawierucha, K; Stec, D; Lachowska-Cierlik, D; Takeuchi, N; Li, ZQ; Michalczyk, L | 2018 | HIGH MITOCHONDRIAL DIVERSITY IN A NEW WATER BEAR SPECIES (TARDIGRADA: EUTARDIGRADA) FROM MOUNTAIN GLACIERS IN CENTRAL ASIA, WITH THE ERECTION OF A NEW GENUS CRYOCONICUS | Glaciers and ice sheets are considered a biome with unique organism assemblages. Tardigrada (water bears) are micrometazoans that play the function of apex consumers on glaciers. Cryoconite samples with the dark-pigmented tardigrade Cryoconicus gen. nov. kaczmareki sp. nov. were collected from four locations on glaciers in China and Kyrgyzstan. The erection of the new genus is based on a unique combination of morphological traits as well as on phylogenetic analyses. The analysis of COI sequences in the new species revealed high genetic differentiation with 9 haplotypes shared among 13 sequenced individuals from three sequenced populations. There was no apparent geographic structure in COI haplotype diversity, which might indicate effective dispersal abilities of the new species. A recovery of numerous live individuals from a sample that was frozen for 11 years suggests high survival rates in the natural environment. The ability to withstand low temperatures, combined with dark pigmentation that is hypothesised to protect from intense UV radiation, could explain how the new taxon is able to dwell in an extreme glacial habitat. We also found that a rare mountain tardigrade Ramazzottius cataphractus (Maucci, 1974) is morphologically similar to the new species, therefore we propose to transfer it to the new genus. Our study indicates that glacier invertebrate fauna is still poorly known and requires intense research. | NA | no | No good fitness measure and measure host GD | NA | no | no |
| 5815 | 2020 | 44111 | Zayasu, Y; Suzuki, G | 2019 | Comparisons of population density and genetic diversity in artificial and wild populations of an arborescent coral, Acropora yongei: implications for the efficacy of artificial spawning hotspots | We are developing techniques to restore coral populations by enhancing larval supply using artificial spawning hotspots that aggregate conspecific adult corals. However, no data were available regarding how natural larval supply from wild coral populations is influenced by fertilization rate and how this is in turn affected by local population density and genetic diversity. Therefore, we assessed population density and genetic diversity of a wild, arborescent coral, Acropora yongei, and compared these parameters with those of an artificially established A. yongei population in the field. The population density of wild arborescent corals was only 0.27% of that in the artificial population, even in a high-coverage area. Genetic diversity was also low in the wild population compared with the artificial population, and approximately 10% of all wild colonies were clones. Based on these results, the larval supply in the artificial population was estimated to be at least 1,400 times higher than that in wild A. yongei populations for the same area of adult population. | NA | no | No good fitness measure | NA | no | no |
| 5816 | 2020 | 44042 | Zeb, J; Szekeres, S; Takacs, N; Kontschan, J; Shams, S; Ayaz, S; Hornok, S | 2019 | Genetic diversity, piroplasms and trypanosomes in Rhipicephalus microplus and Hyalomma anatolicum collected from cattle in northern Pakistan | The two most important tick species in Pakistan are Rhipicephalus microplus and Hyalomma anatolicum. When associated with cattle, these have one or three host life cycles, respectively, with potential implications for their population genetics and for their vector role in the transmission of pathogens. To compare the two tick species in this context with molecular-phylogenetic methods, during the present study 123 ticks were collected from cattle in northern Pakistan. Two mitochondrial markers of 36 ticks were molecularly analyzed. All 11 R. microplus specimens had identical cox1 haplotypes, whereas the 25 H. anatolicum specimens had nine cox1 haplotypes. The latter belonged to two distinct phylogenetic lineages with high support. However, in the 16S rRNA gene these differences were less evident. Among the 113 ticks molecularly analyzed for tick-borne protozoa, the sequence of Babesia occultans was successfully amplified from two specimens of H. anatolicum. Theileria annulata was present in both R. microplus (10.4%) and H. anatolicum (27.3%), with significantly higher prevalence rate in the latter species. Only one tick, a H. anatolicum female, was positive in the PCR detecting Trypanosoma spp. Sequencing revealed the presence of a new genotype, with the closest phylogenetic relationship to stercorarian trypanosomes (in particular, to a tick-associated Trypanosoma sp. from Japan). In conclusion, the above differences between R. microplus and H. anatolicum may be partly related to their life cycles involving one host or three hosts, respectively. Among the others, host switching (reducing chances of inbreeding) and shorter periods spent on-host (reducing gene flow between cattle herds) are supposed to be important drivers of cox1 gene diversification in case of H. anatolicum as a three host tick species. These results highlight the importance of studying differences in intraspecific genetic diversity and piroplasm burdens between one host and three host ticks in the local scale. In addition, a Trypanosoma sp. molecularly identified in H. anatolicum is reported here for the first time from South Asia, deserving further evaluation concerning its host and vector species. | NA | no | dometicated host | NA | no | no |
| 5817 | 2020 | 44113 | Zhan, XY; Zhu, QY | 2017 | Molecular evolution of virulence genes and non-virulence genes in clinical, natural and artificial environmental Legionella pneumophila isolates | Background. L. pneumophila is the main causative agent of Legionnaires’ disease. Free-living amoeba in natural aquatic environments is the reservoir and shelter for L. pneumophila, From natural water sources, L. pneumophila can colonize artificial environments such as cooling towers land hot-water systems, and then spread in aerosols, infecting the susceptible person. Therefore, molecular phylogeny land genetic variability of L. pneumophila from different sources (natural water, artificial water, and human lung tissue) might be distinct because of the selection pressure in different. environments. Several studies researched genetic differences between L. pneumophtla clinical isolates and environmental isolates at the nucleotide sequence level. These reports mainly focused on the analysis of virulence genes, and rarely distinguished artificial and natural isolates. Methods. We have used 1139 L pneumophila isolates to study their genetic variability and molecular phylogeny. These isolates include 51 artificial isolates, 59 natural isolates, and 129 clinical isolates. The nucleotide sequences of two representative non-virulence (NV) genes (trpA, cca) and three representative virulence genes (icmK, IspE, lssD) were obtained using PCR and DNA sequencing and were analyzed. Results. Levels of genetic variability including haplotypes, haplotype diversity, nucleotide diversity nucleotide difference and the total number of mutations in the virulence loci were higher in the natural isolates. In contrast, levels of genetic variability including polymorphic sites, theta from. polymorphic sites and the total number of mutations in the NV loci were higher clinical isolates. A phylogenetic analysis of each individual gene. tree showed three to six main groups, but. not comprising. the same L. L. pneumophila isolates. We detected recombination events in every virulence loci of natural isolates, but only’detected.them in the cca locus of clinical isolates. Neutrality tests showed that variations in the virulence genes of clinical and environmental isolates were under neutral evolution. TrpA and cca loci of clinical isolates showed significantly negative values of Tajima’s D, Fu and Li’s D\* and F\*, suggesting the presence of negative selection in NV genes of clinical isolate Discussion. Our findings reinforced the point that the natural environments were the primary training place for L. pneumophila virulence, and intragenic recombination was an important strategy in the adaptive evolution of virulence gene. Our study also suggested the selection pressure had unevenly affected these genes and contributed to the different evolutionary patterns existed between NV genes and virulence genes. This work provides clues for future work on population-level and genetics-level questions about ecology and molecular evolution of L. pneumophila, as well as genetic differences of NV genes and virulence genes between this host-range pathogen with different lifestyles. | NA | no | No good fitness measure and measure host GD | NA | no | no |
| 5818 | 2020 | 44042 | Zhang, C; Gao, Y; Ning, ZL; Lu, Y; Zhang, XX; Liu, JJ; Xie, B; Xue, Z; Wang, XJ; Yuan, K; Ge, XL; Pan, YW; Liu, C; Tian, L; Wang, YC; Lu, DS; Hoh, BP; Xu, SH | 2019 | PGG.SNV: understanding the evolutionary and medical implications of human single nucleotide variations in diverse populations | Despite the tremendous growth of the DNA sequencing data in the last decade, our understanding of the human genome is still in its infancy. To understand the implications of genetic variants in the light of population genetics and molecular evolution, we developed a database, PGG.SNV (https://www.pggsnv.org), which gives much higher weight to previously under-investigated indigenous populations in Asia. PGG.SNV archives 265 million SNVs across 220,147 present-day genomes and 1018 ancient genomes, including 1009 newly sequenced genomes, representing 977 global populations. Moreover, estimation of population genetic diversity and evolutionary parameters is available in PGG.SNV, a unique feature compared with other databases. | NA | no | No good fitness measure and measure host GD | NA | no | no |
| 5819 | 2020 | 44111 | Zhang, CY; Lin, D; Wang, YZ; Peng, DZ; Li, HF; Fei, J; Chen, KW; Yang, N; Hu, XX; Zhao, YQ; Li, N | 2019 | Widespread introgression in Chinese indigenous chicken breeds from commercial broiler | Chinese indigenous chickens (CICs) constitute world-renowned genetic resources due to their excellent traits, including early puberty, good meat quality and strong resistance to disease. Unfortunately, the introduction of a large number of commercial chickens in the past two decades has had an adverse effect on CICs. Using the chicken 60 K single nucleotide polymorphism chip, we assessed the genetic diversity and population structure of 1,187 chickens, representing eight Chinese indigenous chicken breeds, two hybrid chicken breeds, two ancestral chicken breeds, two commercial populations and additional red jungle fowl. By investigating haplotype similarity, we found extensive gene introgression from commercial broiler to almost all CICs. Approximately 15% of the genome, on average, of CICs was introgressed, ranging from 0.64% for Tibetan chicken to 21.52% for Huiyang Bearded chicken. Further analysis revealed signals consistent with positive selection in the introgression loci. For the first time, we systematically mapped and quantified introgression from commercial broiler to CICs at the whole genome level. Our data provided a usable resource for chicken genetic diversity, and our findings indicated a dire need for protecting the genetic resources of CICs. | NA | no | No good fitness measure and measure host GD | NA | no | no |
| 5820 | 2020 | 44112 | Zhang, JM; Lopez-Pujol, J; Gong, X; Wang, HF; Vilatersana, R; Zhou, SL | 2018 | Population genetic dynamics of Himalayan-Hengduan tree peonies, Paeonia subsect. Delavayanae | According to the present taxonomical treatment, Paeonia subsect. Delavayanae consists of only two species (P. delavayi and P. ludlowii) endemic to the Himalayan-Hengduan Mountains. Although P. ludlowii can be distinguished from P. delavayi on the basis of a series of morphological characters, the species delimitation remains controversial because the more widespread one, P. delavayi, exhibits considerable morphological diversity. Both chloroplast DNA markers and nuclear microsatellites or simple sequence repeats (nSSR) are used herein to reveal genetic diversity and relationships of the two taxa included in this subsection, and ecological niche modeling (ENM) is employed to get insights into their paleodistribution. Our results show that genetic boundaries between the two currently recognized species are unclear, probably due to recent divergence. Paeonia ludlowii is budding from P. delavayi, probably by genetic isolation but also by shifting its niche to the harsher upland Tibetan conditions. Paeonia delavayi itself would be, however, under active speciation, showing significant genetic differentiation and morphological diversity. Whereas P. ludlowii would have endured the Pleistocene glacial periods by in situ persistence in local, small refugia, a ‘dual’ model seems to apply for P. delavayi (in situ persistence and retreat to refugia). The rarity of P. ludlowii and high evolutionary potential of P. delavayi imply high priority for in situ conservation of both taxa. The Himalayan-Hengduan Mountains are an ideal arena for differentiation within subsect. Delavayanae of Paeonia, by means of expansions/contractions/displacements, vertical migrations, and local survival/extinctions in response to the Neogene climate fluctuations and geological changes. | NA | no | No good fitness measure and measure host GD | NA | no | no |
| 5821 | 2020 | 44112 | Zhang, JX; Liu, ZG; Zou, YW; Zhang, NXZ; Wang, DL; Tu, D; Yang, LC; Deng, ZB; Yang, Y; Jiang, P; Wang, ND | 2018 | First molecular detection of porcine circovirus type 3 in dogs in China | Porcine circovirus type 3 (PCV3) has recently been isolated from diseased pigs within the USA. The objective was to detect the presence of PCV3 in dogs. Nested polymerase chain reactions (PCR) with PCV3-specific primers for the capsid gene were used to detect PCV3 genomic DNA in serum samples from dogs (n = 44) in China. There was PCV3 DNA detected in 4 of 44 dogs [all were negative for PCV2 and canine circovirus (CanineCV)]. Based on sequence analysis, positive sequences were grouped into PCV3 genotypes. However, these isolates had close evolutionary relationships with FoxCV (KP941114) and CanineCV (JQ821392). Further investigations of the epidemiology, evolutionary biology, and pathobiology of PCV3 to dogs are warranted. | NA | no | dog | NA | no | no |
| 5822 | 2020 | 44112 | Zhang, M; Peng, WF; Hu, XJ; Zhao, YX; Lv, FH; Yang, J | 2018 | Global genomic diversity and conservation priorities for domestic animals are associated with the economies of their regions of origin | Domestic animals play a key role in human survival and the development of civilization. However, the genetic resources of domestic animals are facing an alarming rate of erosion due to socioeconomic changes, economic globalization and financial constraints. In this study, through genome-wide SNP analysis, we estimated the heterozygosity, inbreeding coefficient, effective population size, and runs of homozygosity to identify the breeds facing the risk of extinction for sheep and cattle across the world. In particular, we quantified the contribution of 97 sheep breeds and 53 cattle breeds to genomic diversity (within-breed, between-breed and total) and prioritized the breeds for conservation. Additionally, we compared the average values of genomic diversity between breeds from regions (or countries) in different economic categories (underdeveloped, developing and developed), and found that breeds in developed regions exhibit significantly higher levels of total genomic diversity than those in underdeveloped and developing regions. Altogether, our results suggested that conservation priority should be given to breeds in developed regions to secure the future genomic diversity hotspots of domestic animal resources. | NA | no | dometicated host | NA | no | no |
| 5823 | 2020 | 44113 | Zhang, P; Huang, K; Zhang, BY; Dunn, DW; Chen, D; Li, F; Qi, XG; Guo, ST; Li, BG | 2018 | High polymorphism in MHC-DRB genes in golden snub-nosed monkeys reveals balancing selection in small, isolated populations | Background: Maintaining variation in immune genes, such as those of the major histocompatibility complex (MHC), is important for individuals in small, isolated populations to resist pathogens and parasites. The golden snub-nosed monkey (Rhinopithecus roxellana), an endangered primate endemic to China, has experienced a rapid reduction in numbers and severe population fragmentation over recent years. For this study, we measured the DRB diversity among 122 monkeys from three populations in the Qinling Mountains, and estimated the relative importance of different agents of selection in maintaining variation of DRB genes. Results: We identified a total of 19 DRB sequences, in which five alleles were novel. We found high DRB variation in R. roxellana and three branches of evidence suggesting that balancing selection has contributed to maintaining MHC polymorphism over the long term in this species: i) different patterns of both genetic diversity and population differentiation were detected at MHC and neutral markers; ii) an excess of non-synonymous substitutions compared to synonymous substitutions at antigen binding sites, and maximum-likelihood-based random-site models, showed significant positive selection; and iii) phylogenetic analyses revealed a pattern of trans-species evolution for DRB genes. Conclusions: High levels of DRB diversity in these R. roxellana populations may reflect strong selection pressure in this species. Patterns of genetic diversity and population differentiation, positive selection, as well as trans-species evolution, suggest that pathogen-mediated balancing selection has contributed to maintaining MHC polymorphism in R. roxellana over the long term. This study furthers our understanding of the role pathogen-mediated balancing selection has in maintaining variation in MHC genes in small and fragmented populations of free-ranging vertebrates. | NA | no | mhc diversity | NA | no | no |
| 5824 | 2020 | 44112 | Zhang, S; Chen, HF; Liu, JX; Liu, DM; Yan, HL; Wang, FG | 2019 | Effects of drought on Physiological Responses of Bretschneidera sinensis | Bretschneidera sinensis Hemsl. is a rare and endangered species in China. Understanding the effects of drought on its physiological responses is required for the introduction and protection of this species with future climate change. In order to know the drought effects on plasma membrane permeability (PMP), malondialdehyde (MDA), proline, superoxide dismutase (SOD) and chlorophyll (chl) in B. sinensis, 100 biennial seedlings of B. sinensis were divided into five groups and subjected to different treatments, i.e., a reduction in the amount of water applied on a daily basis. The soluble sugar, soluble starch and nonstructural carbohydrate (NSC) contents were also measured prior to the death of B. sinensis. The results indicated that, as the drought continued and intensified, the proline and SOD contents changed slightly, while the PMP and the MDA content increased constantly, especially under severe drought. The chl content also showed a decreasing trend. Consequently, B. sinensis became highly sensitive to the drought because the repair mechanisms could not keep pace with the damage. When close to death, the B. sinensis seedlings did not use high levels of NSCs. Although, NSCs did not decline, but gradually transferred from leaf to stem and then to roots. Meanwhile, the composition of NSCs changed i.e., soluble starch was degraded to soluble sugar, which indicates an adaptive mechanism in a long-term drought environment. It was concluded that B. sinensis is highly sensitive to the drought because of several physiological changes in B. sinensis, however, carbon starvation would not occur in B. sinensis during drought. (C) 2019 Friends Science Publishers | NA | no | No good fitness measure and measure host GD | NA | no | no |
| 5825 | 2020 | 44112 | Zhang, SK; Shu, JP; Xue, HJ; Zhang, W; Wang, YD; Liu, YN; Wang, HJ | 2018 | Genetic diversity in the camellia weevil, Curculio chinensis Chevrolat (Coleptera: Curculionidae) and inferences for the impact of host plant and human activity | The camellia weevil, Curculio chinensis (Coleoptera: Curculionidae), is a host-specific parasite of Camellia seeds that causes huge economic losses in China. Despite its economic impact, little is known about the species’ genetic structure and current distribution status, which are important to provide critical insights into establishing a control strategy for this pest. Here, genetic diversity and the phylogenetic structure of C. chinensis were inferred using the sequences of COI and EF1-alpha among different geographical populations. Twenty-two haplotypes for the COI gene and 26 haplotypes for EF1-alpha in 215 individuals from 16 sample regions in China were found. A phylogenetic analysis revealed four distinct clades within C. chinensis. The amova analysis showed significant genetic differentiation among all the populations sampled, however, the genetic differentiation among the residual populations was not significant when JX (Jiangxi) and TC (Tengchong) populations were excluded. The M (M = Nm for haploid data, M = 2 Nm for diploid data) value analysis suggested that JX and TC populations were significantly different from other populations. Thus, these results imply that human activity and host plant could have a significant impact on C. chinensis population diversity and the current geographical population pattern. | NA | no | gd of pathogen | NA | no | no |
| 5826 | 2020 | 44113 | Zhang, W; Manawasinghe, IS; Zhao, WS; Xu, JP; Brooks, S; Zhao, XY; Hyde, KD; Chethana, KWT; Liu, JH; Li, XH; Yan, JY | 2017 | Multiple gene genealogy reveals high genetic diversity and evidence for multiple origins of Chinese Plasmopara viticola population | Downy mildew caused by Plasmopara viticola is one of the most devastating diseases of grapevines worldwide. So far, the genetic diversity and origin of the Chinese P. viticola population are unclear. In the present study, 103 P. viticola isolates were sequenced at four gene regions: internal transcribed spacer one (ITS), large subunit of ribosomal RNA (LSU), actin gene (ACT) and beta-tubulin (TUB). The sequences were analyzed to obtain polymorphism and diversity information of the Chinese population as well as to infer the relationships between Chinese and American isolates. High genetic diversity was observed for the Chinese population, with evidence of sub-structuring based on climate. Phylogenetic analysis and haplotype networks showed evidence of close relationships between some American and Chinese isolates, consistent with recent introduction from America to China via planting materials. However, there is also evidence for endemic Chinese P. viticola isolates. Our results suggest that the current Chinese Plasmopara viticola population is an admixture of endemic and introduced isolates. | NA | no | No good fitness measure and measure host GD | NA | no | no |
| 5827 | 2020 | 44040 | Zhang, Y; Colli, L; Barker, JSF | 2020 | Asian water buffalo: domestication, history and genetics | The domestic Asian water buffalo (Bubalus bubalis) is found on all five continents, with a global population of some 202 million. The livelihoods of more people depend on this species than on any other domestic animal. The two distinct types (river and swamp) descended from different wild Asian water buffalo (Bubalus arnee) populations that diverged some 900 kyr BP and then evolved in separate geographical regions. After domestication in the western region of the Indian subcontinent (ca. 6300 years BP), the river buffalo spread west as far as Egypt, the Balkans and Italy. Conversely, after domestication in the China/Indochina border region ca. 3000-7000 years BP, swamp buffaloes dispersed through south-east Asia and China as far as the Yangtze River valley. Molecular and morphological evidence indicates that swamp buffalo populations have strong geographic genetic differentiation and a lack of gene flow, but strong phenotypic uniformity. In contrast, river buffalo populations show a weaker phylogeographic structure, but higher phenotypic diversity (i.e. many breeds). The recent availability of a high-quality reference genome and of a medium-density marker panel for genotyping has triggered a number of genome-wide investigations on diversity, evolutionary history, production traits and functional elements. The growing molecular knowledge combined with breeding programmes should pave the way to improvements in production, environmental adaptation and disease resistance in water buffalo populations worldwide. | NA | no | dometicated host | NA | no | no |
| 5828 | 2020 | 44041 | Zhang, YQ; Million, WC; Ruggeri, M; Kenkel, CD | 2019 | Family matters: Variation in the physiology of brooded Porites astreoides larvae is driven by parent colony effects | The planktonic larval phase of scleractinian coral life-history represents a crucial stage when dispersal takes place and genetic diversity among populations is maintained. Understanding the dynamics influencing larval survival is especially relevant in the context of climate change, as larvae may be more vulnerable to environmental disturbances than adults. Several physiological parameters of coral larvae have been shown to vary by release time and past environmental history. However, the contribution of parental or genetic effects is largely unknown. To investigate these potential familial effects, we collected adult Porites astreoides colonies in April 2018 from two reef zones in the lower Florida Keys and quantified physiological traits and thermal tolerance of the newly released larvae. Family accounted for more variation than day of release and reef origin, with > 60% of the variation in chlorophyll a and protein content explained by family. The survivorship of larvae under 36 degrees C acute temperature stress was also tightly linked to what parent colony they were released from. During a 32 degrees C moderate temperature stress experiment, inshore larvae tended to bleach less than offshore larvae, mirroring the enhanced bleaching resistance previously observed in inshore adult coral populations. The significant familial effects identified in the present study suggest that researchers should be cautious when interpreting results of studies which pool larvae among families, and that future studies should take care to account for this variation. | NA | no | No good fitness measure and measure host GD | NA | no | no |
| 5829 | 2020 | 44039 | Zheng, X; Li, JJ; Sheng, J; Dai, Y; Wang, Y; Liu, JB; Xu, Y | 2020 | Haplotypes of the Mutated SIRT2 Promoter Contributing to Transcription Factor Binding and Type 2 Diabetes Susceptibility | Genetic variability is an important causative factor for susceptibility and pathogenesis of type 2 diabetes (T2D). Histone deacetylase, sirtuin 2 (SIRT2), plays regulatory roles in glucose metabolism and insulin sensitivity. However, whether the SIRT2 variants or haplotypes contribute to T2D risk remain to be elucidated. In this study, we first detected three novel polymorphisms (P-MU1, P-MU2, and P-MU3) in the promoter of SIRT2 in the Chinese population. All pairwise sets of the three loci were strongly in linkage disequilibrium. Next, we constructed the haplotype block structure, and found H1-GGC and H2-CCA accounted for the most (total 91.8%) in T2D. The haplotype combination H1-H1-GGGGCC displayed a high risk for T2D (OR = 2.03, 95% CI = 1.12-3.72). By association analysis, we found the individuals carrying H1-H1-GGGGCC had significantly higher fasting plasma glucose and glycated hemoglobin. The haplotype H1-GGC presented a 6.74-fold higher promoter activity than H2-CCA, which was consistent with the correlation results. Furthermore, we clarified the mechanism whereby the C allele of both the P-MU1 and P-MU2 loci disrupted the signal transducer and activator of transcription 1 (STAT1) binding sites, leading to the attenuation of the SIRT2 transcription. Together, these data suggest that the linked haplotype GGC could be considered as a promising marker for T2D diagnosis and therapy assessment. | NA | no | No good fitness measure and measure host GD | NA | no | no |
| 5830 | 2020 | 44040 | Zhong, YZ; De, TNM; Alarcon, C; Park, CS; Lec, B; Perera, MA | 2020 | Discovery of novel hepatocyte eQTLs in African Americans | African Americans (AAs) are disproportionately affected by metabolic diseases and adverse drug events, with limited publicly available genomic and transcriptomic data to advance the knowledge of the molecular underpinnings or genetic associations to these diseases or drug response phenotypes. To fill this gap, we obtained 60 primary hepatocyte cultures from AA liver donors for genome-wide mapping of expression quantitative trait loci (eQTL) using LAMatrix. We identified 277 eGenes and 19,770 eQTLs, of which 67 eGenes and 7,415 eQTLs are not observed in the Genotype-Tissue Expression Project (GTEx) liver eQTL analysis. Of the eGenes found in GTEx only 25 share the same lead eQTL. These AA-specific eQTLs are less correlated to GTEx eQTLs. in effect sizes and have larger Fst values compared to eQTLs found in both cohorts (overlapping eQTLs). We assessed the overlap between GWAS variants and their tagging variants with AA hepatocyte eQTLs and demonstrated that AA hepatocyte eQTLs can decrease the number of potential causal variants at GWAS loci. Additionally, we identified 75,002 exon QTLs of which 48.8% are not eQTLs in AA hepatocytes. Our analysis provides the first comprehensive characterization of AA hepatocyte eQTLs and highlights the unique discoveries that are made possible due to the increased genetic diversity within the African ancestry genome. Author summary Precision medicine has enabled more accurate diagnoses, treatments and outcomes with the implementation ongoing at many US hospitals. However, much of these efforts are based on genetic associations conducted in European cohorts. The discovery of disease associated genetic variants is lacking in minority populations, leading to a growing disparity in precision medicine. Our study is the first to map eQTLs in hepatocytes obtained exclusively from African Americans. The liver is a clinically relevant organ linked to many diseases, including cardiovascular and lipid traits, in addition to its importance in drug metabolism. Our analysis replicated previously identified eQTLs within the GTEx and uncovered novel regulatory variants. Many of these novel eQTLs were discovered due to the higher allele frequency of these variants in African ancestry populations, thereby demonstrating the potential increase in power with the use of African ancestry genomes. We decreased the number of potential causal variants at GWAS loci that overlap with our eQTLs. This is important as fine-mapping of causal variants has primarily relied on functional validation. Thus, narrowing the number of potential causal variants expedites this process. Lastly, we show that by identifying both population-specific and hepatocyte-specific eQTLs in African Americans, we can identify potential genes that drive disease. | NA | no | human | NA | no | no |
| 5831 | 2020 | 44111 | Zhong, YZ; Perera, MA; Gamazon, ER | 2019 | On Using Local Ancestry to Characterize the Genetic Architecture of Human Traits: Genetic Regulation of Gene Expression in Multiethnic or Admixed Populations | Understanding the nature of the genetic regulation of gene expression promises to advance our understanding of the genetic basis of disease. However, the methodological impact of the use of local ancestry on high-dimensional omics analyses, including, most prominently, expression quantitative trait loci (eQTL) mapping and trait heritability estimation, in admixed populations remains critically underexplored. Here, we develop a statistical framework that characterizes the relationships among the determinants of the genetic architecture of an important class of molecular traits. We provide a computationally efficient approach to local ancestry analysis in eQTL mapping while increasing control of type I and type II error over traditional approaches. Applying our method to National Institute of General Medical Sciences (NIGMS) and Genotype-Tissue Expression (GTEx) datasets, we show that the use of local ancestry can improve eQTL mapping in admixed and multiethnic populations, respectively. We estimate the trait variance explained by ancestry by using local admixture relatedness between individuals. By using simulations of diverse genetic architectures and degrees of confounding, we show improved accuracy in estimating heritability when accounting for local ancestry similarity. Furthermore, we characterize the sparse versus polygenic components of gene expression in admixed individuals. Our study has important methodological implications for genetic analysis of omics traits across a range of genomic contexts, from a single variant to a prioritized region to the entire genome. Our findings highlight the importance of using local ancestry to better characterize the heritability of complex traits and to more accurately map genetic associations. | NA | no | human | NA | no | no |
| 5832 | 2020 | 44112 | Zhu, LF; Deng, C; Zhao, X; Ding, JJ; Huang, HS; Zhu, SL; Wang, ZW; Qin, SS; Ding, YH; Lu, GQ; Yang, ZS | 2018 | Endangered Pere David’s deer genome provides insights into population recovering | The Milu (Pere David’s deer, Elaphurus davidianus) were once widely distributed in the swamps (coastal areas to inland areas) of East Asia. The dramatic recovery of the Milu population is now deemed a classic example of how highly endangered animal species can be rescued. However, the molecular mechanisms that underpinned this population recovery remain largely unknown. Here, different approaches (genome sequencing, resequencing, and salinity analysis) were utilized to elucidate the aforementioned molecular mechanisms. The comparative genomic analyses revealed that the largest recovered Milu population carries extensive genetic diversity despite an extreme population bottleneck. And the protracted inbreeding history might have facilitated the purging of deleterious recessive alleles. Seventeen genes that are putatively related to reproduction, embryonic (fatal) development, and immune response were under high selective pressure. Besides, SCNN1A, a gene involved in controlling reabsorption of sodium in the body, was positively selected. An additional 29 genes were also observed to be positively selected, which are involved in blood pressure regulation, cardiovascular development, cholesterol regulation, glycemic control, and thyroid hormone synthesis. It is possible that these genetic adaptations were required to buffer the negative effects commonly associated with a high-salt diet. The associated genetic adaptions are likely to have enabled increased breeding success and fetal survival. The future success of Milu population management might depend on the successful reintroduction of the animal to historically important distribution regions. | NA | no | No good fitness measure and measure host GD | NA | no | no |
| 5833 | 2020 | 44043 | Zhu, SJ; Hendry, JA; Almagro-Garcia, J; Pearson, RD; Amato, R; Miles, A; Weiss, DJ; Lucas, TCD; Nguyen, M; Gething, PW; Kwiatkowski, D; McVean, G | 2019 | The origins and relatedness structure of mixed infections vary with local prevalence of P. falciparum malaria | Individual malaria infections can carry multiple strains of Plasmodium falciparum with varying levels of relatedness. Yet, how local epidemiology affects the properties of such mixed infections remains unclear. Here, we develop an enhanced method for strain deconvolution from genome sequencing data, which estimates the number of strains, their proportions, identity-by-descent (IBD) profiles and individual haplotypes. Applying it to the Pf3k data set, we find that the rate of mixed infection varies from 29% to 63% across countries and that 51% of mixed infections involve more than two strains. Furthermore, we estimate that 47% of symptomatic dual infections contain sibling strains likely to have been co-transmitted from a single mosquito, and find evidence of mixed infections propagated over successive infection cycles. Finally, leveraging data from the Malaria Atlas Project, we find that prevalence correlates within Africa, but not Asia, with both the rate of mixed infection and the level of IBD. | NA | no | gd of pathogen | NA | no | no |
| 5834 | 2020 | 44111 | Zhu, Y; Zhong, ZW; Pages, JF; Finke, D; Wang, D; Ma, QH; Hassan, N; Zhu, H; Wang, L | 2019 | Negative effects of vertebrate on invertebrate herbivores mediated by enhanced plant nitrogen content | Classic theory holds that the main interaction within the herbivore guild is competition, based on research focused on co-occurring, similarly sized species that reduce the quantity of shared plant resources. However, plant quality may also be crucial in mediating herbivore interspecific interactions. This is especially true when competition occurs between distantly related herbivore species, given that small terrestrial herbivores (e.g., insect herbivores) appear to be more sensitive to alterations of plant quality than plant quantity. In this study, we first tested in the field whether large vertebrate herbivores (cattle Bos taurus) exerted a negative effect on smaller insect herbivores (grasshopper Euchorthippus unicolor) through their overlapping foraging preferences for a dominant grass Leymus chinensis. We measured changes in grass quantity, grass quality, and microclimatic conditions in response to vertebrate grazing and conducted additional manipulative studies in the field and the laboratory to identify potential mechanisms underlying the interaction. Our results showed that grazing by large herbivores caused a significant decline in grasshopper population density and individual performance (survival, size, and weight of both female and male E. unicolor), despite a 38% increase in grass nitrogen (N) content in grazed plots. Experiments manipulating N levels of L. chinensis in the field and the laboratory confirmed that enriching plant N had a negative effect on grasshopper individual performance and population size. Therefore, enhanced quality (N content) of plant resources appears to be an important driver in mediating the negative effect of vertebrate grazing on grasshoppers. Synthesis. We document that phylogenetic relatedness and trait similarity can be poor predictors of interaction strength in some cases, since distantly related herbivores of disparate size can interact indirectly via changes in plant quality. Counter-intuitively, the observed negative effect of cattle on grasshoppers was mediated, at least in part, by an increase in plant quality in cattle grazed areas. The implication is that light to moderate grazing, a common management strategy, may contribute to suppression of grasshoppers in the Eurasian steppe grassland system by altering plant nutrient supplies. | NA | no | no good gd measure host pop | NA | no | no |
| 5835 | 2020 | 44112 | Ziadie, MA; Ebot-Ojong, F; McKinney, EC; Moore, AJ | 2019 | Evolution of Personal and Social Immunity in the Context of Parental Care | Social immunity moderates the spread of pathogens in social groups and is especially likely in groups structured by genetic relatedness. The extent to which specific immune pathways are used is unknown. Here, we investigate the expression and social role of three functionally separate immune genes (pgrp-sc2, thaumatin, and defensin) during parental care in the beetle Nicrophorus vespilloides. These genes reside in different immune pathways, allowing us to test whether specific components of the immune system are targeted for social immunity. To test for the evolution of specificity, we manipulated the influence of social context and timing on gene expression and quantified the covariance of maternal immune gene expression and offspring fitness. Larvae reduced expression of all three genes in the presence of parents. Parental pgrp-sc2 and thaumatin increased during direct parenting, while defensin was upregulated before larvae arrived. Parental expression of pgrp-sc2 and thaumatin responded similarly to experimental manipulation of timing and presence of larvae, which differed from the response of defensin. We found a positive covariance between maternal expression and offspring fitness for pgrp-sc2 and thaumatin but not defensin. We suggest that social immunity can involve specific genes and pathways, reflecting evolution as an interacting phenotype during parenting. | NA | no | no good gd measure host pop | NA | no | no |
| 5836 | 2020 | 44112 | Zou, X; Wang, Z; He, GL; Wang, MG; Su, YD; Liu, J; Chen, PY; Wang, SY; Gao, B; Li, Z; Hou, YP | 2018 | Population Genetic Diversity and Phylogenetic Characteristics for High-Altitude Adaptive Kham Tibetan Revealed by DNATyper (TM) 19 Amplification System | Tibetans residing in the high-altitude inhospitable environment have undergone significant natural selection of their genetic architecture. Recently, highly mutational autosomal short tandem repeats were widely used not only in the anthropology and population genetics to investigate the genetic structure and relationships, but also in the medical genetics to explore the pathogenesis of multiple genetic diseases and in the forensic science to identify individual and parentage relatedness. However, genetic variants and forensic efficiency of DNATyper (TM) 19 amplification system and genetic background of Kham Tibetan remain uncharacterized. Thus, we genotyped 19 forensic genetic markers in 11,402 Kham Tibetans to gain insight into the genetic diversity of Chinese high-altitude adaptive population. Highly discriminating and polymorphic forensic measures were observed, which indicated that this new-developed DNATyper 19 PCR amplification is suitable for routine forensic identification purposes and Chinese national DNA database establishment. Pairwise genetic distances among the comprehensive population comparisons suggested that this high-altitude adaptive Kham Tibetan has genetically closer relationships with lowlanders of Tibeto-Burmanspeaking populations (Chengdu Tibetan, Liangshan Tibetan, and Liangshan Yi). Genetic substructure analyses via phylogenetic reconstruction, principal component analysis, and multidimensional scaling analysis in both nationwide and worldwide contexts suggested that the genetic proximity exists along the linguistic, ethnic, and continental geographical boundary. Further studies with whole-genome sequencing of modern or archaic Kham Tibetans would be useful in reconstructing the Tibetan population history. | NA | no | human | NA | no | no |
| 5837 | 2020 | 44040 | Zozomova-Lihova, J; Melicharkova, A; Svitok, M; Spaniel, S | 2020 | Pleistocene range disruption and postglacial expansion with secondary contacts explain the genetic and cytotype structure in the western Balkan endemic Alyssum austrodalmaticum (Brassicaceae) | The western Balkans represents an area of significant topographic and environmental heterogeneity, harbouring high species and intraspecific diversity. Similar genetic and distributional splits observed in unrelated species have suggested some common features in their glacial response and biogeographic history. Here, we studied the western Balkan endemic Alyssum austrodalmaticum with the aim to explore and understand its intraspecific structure and processes that shaped the present patterns. We employed data from AFLPs, two low-copy nuclear genes, genome size, morphometrics and species distribution modelling. Four genetic lineages were identified within the species, which were geographically structured and showed cytotype-specific patterns. The observed phylogeographic structure is congruent with the predicted species distribution during the Last Glacial Maximum. Two allopatric diploid lineages (referred to as N2x and S2x) reflect glacial range disruption and survival in distinct refugia inferred in north-western (Istria, Kvarner) and south-eastern Adriatic areas (northern Adriatic palaeo-coastline and southern Dinarides). AFLP analyses with in silico-generated polyploid genotypes and nuclear genes proved that the two tetraploid lineages (C4x, S4x) were genetic allopolyploids and arose independently. The central tetraploids (C4x) originated through secondary contacts of the two diploid lineages. The origin of the southern tetraploids (S4x) is somewhat ambiguous. Apart from the southern diploids (S2x), the involvement, either direct or through later introgression, of the central tetraploids (C4x) or even other Balkan relatives is possible. Our study highlights the role of glacial range dynamics and secondary contacts, triggering introgression and polyploid evolution, in the formation of genetic diversity and intraspecific patterns in the western Balkans. | NA | no | No good fitness measure and measure host GD | NA | no | no |
| 5838 | 2020 | 44039 | Zuercher, ME; Monson, TA; Dvoretzky, RR; Ravindramurthy, S; Hlusko, LJ | 2020 | Dental Variation in Megabats (Chiroptera: Pteropodidae): Tooth Metrics Correlate with Body Size and Tooth Proportions Reflect Phylogeny | Variation in the dentition yields insight into the evolutionary history of Mammalia. However, to date, there has been limited research on the dental variation in Pteropodidae, a family of bats found throughout sub-Saharan Africa, Southeast Asia, and Oceania. Most species are large, diurnal, non-echolocating, and eat fruit or nectar. Pteropodids are of significant concern in conservation due to rapidly declining populations resulting from habitat loss, climate change, and their impacts on agriculture and disease. We collected dental metrics from the mandibular postcanine teeth of 101 pteropodid specimens spanning six species within the family to test three hypotheses: H1) dental metrics are significantly different across pteropodid species; H2) variation in pteropodid dental metrics is associated with variation in body size; and H3) variation in pteropodid dental proportions is associated with phylogenetic relatedness. We find that dental linear metrics vary significantly across pteropodids and are significantly associated with body size. In contrast, dental proportions of pteropodids reflect phylogenetic relationships. We propose that the combination of approaches for quantifying postcanine dental variation can elucidate and refine our understanding of the various selective forces that shaped the Pteropodidae radiation. | NA | no | No good fitness measure and measure host GD | NA | no | no |
| 5839 | 2020 | 44113 | Zurn, JD; Rouse, MN; Chao, S; Aoun, M; Macharia, G; Hiebert, CW; Pretorius, ZA; Bonman, JM; Acevedo, M | 2018 | Dissection of the multigenic wheat stem rust resistance present in the Montenegrin spring wheat accession PI 362698 | Background: Research to identify and characterize stem rust resistance genes in common wheat, Triticum aestivum, has been stimulated by the emergence of Ug99-lineage races of the wheat stem rust pathogen, Puccinia graminis f. sp. tritici (Pgt), in Eastern Africa. The Montenegrin spring wheat landrace PI 362698 was identified as a source of Pgt resistance. This accession exhibits resistance to multiple Ug99-lineage and North American Pgt races at seedling and adult-plant stages. A recombinant inbred population was developed by crossing the susceptible line LMPG-6 with a single plant selection of PI 362698. A genetic map was constructed using the Illumina iSelect 90 K wheat assay and the markers csLv34, NB-LRR3, and wMAS000003 and quantitative trait locus (QTL) analysis was performed. Results: QTL analysis identified five significant QTLs (alpha = 0.05) on chromosomes 2B, 3B, 6A, 6D, and 7A associated with wheat stem rust resistance. The QTL on chromosome 3B was identified using both field data from Kenya (Pgt Ug99-lineage races) and seedling data from Pgt race MCCF. This QTL potentially corresponds to Sr12 or a new allele of Sr12. The multi-pathogen resistance gene Sr57 located on chromosome 7D is present in PI 362698 according to the diagnostic markers csLv34 and wMAS000003, however a significant QTL was not detected at this locus. The QTLs on chromosomes 2B, 6A, and 6D were identified during seedling trials and are thought to correspond to Sr16, Sr8a, and Sr5, respectively. The QTL identified on chromosome 7A was detected using MCCF seedling data and may be Sr15 or a potentially novel allele of recently detected Ug99 resistance QTLs. Conclusions: The combination of resistance QTLs found in PI 362698 is like the resistance gene combination present in the broadly resistant cultivar Thatcher. As such, PI 362698 may not be a landrace as previously thought. PI 362698 has been crossed with North Dakota wheat germplasm for future breeding efforts. Additional work is needed to fully understand why the combination of genes present in PI 362698 and ‘Thatcher’ provide such durable resistance. | NA | no | No good fitness measure and measure host GD | NA | no | no |
| 5840 | 2020 | 44112 | Guiraud, BSHB; Tahi, MG; Fouet, O; Trebissou, CI; Pokou, D; Rivallan, R; Argout, X; Koffi, KK; Kone, B; Zoro, BIA; Lanaud, C | 2018 | Assessment of genetic diversity and structure in cocoa trees (Theobroma cacao L.) in Cte d’Ivoire with reference to their susceptibility to Cocoa swollen shoot virus disease (CSSVD) | Resistance to Cocoa Swollen Shoot Virus disease (CSSVD) is becoming an increasingly important criterion for selection of new cocoa cultivars in Cte-d’Ivoire, where the disease resurfaced since 2003. This virus can seriously affect the yield of trees with a loss of 25%, 1 year after infection, to around 100% 3 years after. In order to find tolerant plant material, 337 farm accessions have been collected on fields affected by CSSVD, according to the status of accessions potentially tolerant (APT) or susceptible (APS). Both phenotypic groups were genotyped using 30 microsatellite markers (SSR) in the presence of representative clones of the ten genetic groups of cocoa. This study revealed 214 alleles with the set of primer pairs used. The number of alleles per locus was between 3 and 16 with an average of 7.13 alleles per locus. The results showed a high contribution of genetic diversity within population (H (s) = 0.51) to the total genetic diversity (H (t) = 0.53) for the two studied groups. There was no significant difference between tolerant and susceptible groups (Fst = 0.05). These results suggest that APT could be a potential genetic reservoir for other traits of interest associated with virus resistance. The phylogenetic tree, as the STRUCTURE analysis of Ivorian cocoa population, showed a distribution of individuals following four groups marked by a high contribution of group 4 (Nanay, Maranon, Guiana) followed by group 2 (Criollo), and group 1 (Amelonado), and a lower contribution of group 3 (Iquitos, Purus, Nacional, Curaray, Contamana). | NA | probably no | “domesticated host? Not good gd of”“groups”“?” | Comparison of tolerant susceptible individuals | no | no |
| 5841 | 2020 | 44039 | Lado, P; Luan, B; Allerdice, MEJ; Paddock, CD; Karpathy, SE; Klompen, H | 2020 | Integrating population genetic structure, microbiome, and pathogens presence data in Dermacentor variabilis | Tick-borne diseases (TBDs) continue to emerge and re-emerge in several regions of the world, highlighting the need for novel and effective control strategies. The development of effective strategies requires a better understanding of TBDs ecology, and given the complexity of these systems, interdisciplinary approaches are required. In recent years, the microbiome of vectors has received much attention, mainly because associations between native microbes and pathogens may provide a new promising path towards the disruption of pathogen transmission. However, we still do not fully understand how host genetics and environmental factors interact to shape the microbiome of organisms, or how pathogenic microorganisms affect the microbiome and vice versa. The integration of different lines of evidence may be the key to improve our understanding of TBDs ecology. In that context, we generated microbiome and pathogen presence data for Dermacentor variabilis, and integrated those data sets with population genetic data, and metadata for the same individual tick specimens. Clustering and multivariate statistical methods were used to combine, analyze, and visualize data sets. Interpretation of the results is challenging, likely due to the low levels of genetic diversity and the high abundance of a few taxa in the microbiome. Francisella was dominant in almost all ticks, regardless of geography or sex. Nevertheless, our results showed that, overall, ticks from different geographic regions differ in their microbiome composition. Additionally, DNA of Rickettsia rhipicephali, R. montanensis, R. bellii, and Anaplasma spp., was detected in D. variabilis specimens. This is the first study that successfully generated microbiome, population genetics, and pathogen presence data from the same individual ticks, and that attempted to combine the different lines of evidence. The approaches and pre-processing steps used can be applied to a variety of taxa, and help better understand ecological processes in biological systems. | NA | Probably no | No good fitness measure and measure host GD | No good fitness measure and measure host GD | no | no |
| 5842 | 2020 | 44113 | Pais, AL; Li, X; Xiang, QY | 2018 | Discovering variation of secondary metabolite diversity and its relationship with disease resistance in Cornus florida L. | Understanding intraspecific relationships between genetic and functional diversity is a major goal in the field of evolutionary biology and is important for conserving biodiversity. Linking intraspecific molecular patterns of plants to ecological pressures and trait variation remains difficult due to environment-driven plasticity. Next-generation sequencing, untargeted liquid chromatography-mass spectrometry (LC-MS) profiling, and interdisciplinary approaches integrating population genomics, metabolomics, and community ecology permit novel strategies to tackle this problem. We analyzed six natural populations of the disease-threatened Cornus florida L. from distinct ecological regions using genotype-by-sequencing markers and LC-MS-based untargeted metabolite profiling. We tested the hypothesis that higher genetic diversity in C.florida yielded higher chemical diversity and less disease susceptibility (screening hypothesis), and we also determined whether genetically similar subpopulations were similar in chemical composition. Most importantly, we identified metabolites that were associated with candidate loci or were predictive biomarkers of healthy or diseased plants after controlling for environment. Subpopulation clustering patterns based on genetic or chemical distances were largely congruent. While differences in genetic diversity were small among subpopulations, we did observe notable similarities in patterns between subpopulation averages of rarefied-allelic and chemical richness. More specifically, we found that the most abundant compound of a correlated group of putative terpenoid glycosides and derivatives was correlated with tree health when considering chemodiversity. Random forest biomarker and genomewide association tests suggested that this putative iridoid glucoside and other closely associated chemical features were correlated to SNPs under selection. | NA | probably no | ok gd, pop level? And fitness measure of group/pop (disease susxeptibility?) | No direct relationship between genetic diversity and disease | no | no |
| 5843 | 2020 | 44040 | White, PS; Choi, A; Pandey, R; Menezes, A; Penley, M; Gibson, AK; de Roode, J; Morran, L | 2020 | Host heterogeneity mitigates virulence evolution | Parasites often infect genetically diverse host populations, and the evolutionary trajectories of parasite populations may be shaped by levels of host heterogeneity. Mixed genotype host populations, compared to homogeneous host populations, can reduce parasite prevalence and potentially reduce rates of parasite adaptation due to trade-offs associated with adapting to specific host genotypes. Here, we used experimental evolution to select for increased virulence in populations of the bacterial parasite Serratia marcescens exposed to either heterogeneous or homogeneous populations of Caenorhabditis elegans. We found that parasites exposed to heterogeneous host populations evolved significantly less virulence than parasites exposed to homogeneous host populations over several hundred bacterial generations. Thus, host heterogeneity impeded parasite adaptation to host populations. While we detected trade-offs in virulence evolution, parasite adaptation to two specific host genotypes also resulted in modestly increased virulence against the reciprocal host genotypes. These results suggest that parasite adaptation to heterogeneous host populations may be impeded by both trade-offs and a reduction in the efficacy of selection as different host genotypes exert different selective pressures on a parasite population. | NA | yes | focus on parasite, but maybe some way of getting an effect size that works comparing the homogenous and heterogenous host pop groups? | experimental evolution where parasites selected to be more virulent. Ancestral values potentially relevant (figure S3), but no present to mixed and homogeneous populations. | no | no |
| 5844 | 2020 | 44041 | Yates, MC; Bowles, E; Fraser, DJ | 2019 | Small population size and low genomic diversity have no effect on fitness in experimental translocations of a wild fish | Little empirical work in nature has quantified how wild populations with varying effective population sizes and genetic diversity perform when exposed to a gradient of ecologically important environmental conditions. To achieve this, juvenile brook trout from 12 isolated populations or closed metapopulations that differ substantially in population size and genetic diversity were transplanted to previously fishless ponds spanning a wide gradient of ecologically important variables. We evaluated the effect of genome-wide variation, effective population size (N-e), pond habitat, and initial body size on two fitness correlates (survival and growth). Genetic variables had no effect on either fitness correlate, which was determined primarily by habitat (pond temperature, depth, and pH) and initial body size. These results suggest that some vertebrate populations with low genomic diversity, low N-e, and long-term isolation can represent important sources of variation and are capable of maintaining fitness in, and ultimately persisting and adapting to, changing environments. Our results also reinforce the paramount importance of improving available habitat and slowing habitat degradation for species conservation. | NA | yes | looks promising - OK gd measure? | no clear measure of group/population level genetic diversity | no | no |
| 5845 | 2020 | 44042 | Cabalzar, AP; Fields, PD; Kato, Y; Watanabe, H; Ebert, D | 2019 | Parasite-mediated selection in a natural metapopulation of Daphnia magna | Parasite-mediated selection varying across time and space in metapopulations is expected to result in host local adaptation and the maintenance of genetic diversity in disease-related traits. However, nonadaptive processes like migration and extinction-(re)colonization dynamics might interfere with adaptive evolution. Understanding how adaptive and nonadaptive processes interact to shape genetic variability in life-history and disease-related traits can provide important insights into their evolution in subdivided populations. Here we investigate signatures of spatially fluctuating, parasite-mediated selection in a natural metapopulation of Daphnia magna. Host genotypes from infected and uninfected populations were genotyped at microsatellite markers, and phenotyped for life-history and disease traits in common garden experiments. Combining phenotypic and genotypic data a Q(ST)-F-ST-like analysis was conducted to test for signatures of parasite mediated selection. We observed high variation within and among populations for phenotypic traits, but neither an indication of host local adaptation nor a cost of resistance. Infected populations have a higher gene diversity (Hs) than uninfected populations and Hs is strongly positively correlated with fitness. These results suggest a strong parasite effect on reducing population level inbreeding. We discuss how stochastic processes related to frequent extinction-(re)colonization dynamics as well as host and parasite migration impede the evolution of resistance in the infected populations. We suggest that the genetic and phenotypic patterns of variation are a product of dynamic changes in the host gene pool caused by the interaction of colonization bottlenecks, inbreeding, immigration, hybrid vigor, rare host genotype advantage and parasitism. Our study highlights the effect of the parasite in ameliorating the negative fitness consequences caused by the high drift load in this metapopulation. | NA | yes | comp inf pop with uninfected? GD measure of pop OK? | No way to match up data on geneticd diversity/relatedness with mortality or parasite load. Only relative fitness measured which includes effects on reproduction¬† | no | no |
| 5846 | 2020 | 44042 | Fraser, DJ; Walker, L; Yates, MC; Marin, K; Wood, JLA; Bernos, TA; Zastavniouk, C | 2019 | Population correlates of rapid captive-induced maladaptation in a wild fish | Understanding the extent to which captivity generates maladaptation in wild species can inform species recovery programs and elucidate wild population responses to novel environmental change. Although rarely quantified, effective population size (N-e) and genetic diversity should influence the magnitude of plastic and genetic changes manifested in captivity that reduce wild fitness. Sexually dimorphic traits might also mediate consequences of captivity. To evaluate these relationships, we generated >600 full- and half-sibling families from nine wild brook trout populations, reared them for one generation under common, captive environmental conditions and contrasted several fitness-related traits in wild versus captive lines. We found substantial variation in lifetime success (lifetime survival and reproductive success) and life history traits among wild populations after just one captive generation (fourteen- and threefold ranges across populations, respectively). Populations with lower heterozygosity showed lower captive lifetime success, suggesting that captivity generates maladaptation within one generation. Greater male-biased mortality in captivity occurred in populations having disproportionately higher growth rates in males than females. Wild population N-e and allelic diversity had little or no influence on captive trait expression and lifetime success. Our results have four conservation implications: (i) Trait values and lifetime success were highly variable across populations following one generation of captivity. (ii) Maladaptation induced by captive breeding might be particularly intense for the very populations practitioners are most interested in conserving, such as those with low heterozygosity. (iii) Maladaptive sex differences in captivity might be associated with population-dependent growth costs of reproduction. (iv) Heterozygosity can be a good indicator of short-term, intraspecific responses to novel environmental change. | NA | yes | gd of group ok? | Within individual genetic diversity | no | no |
| 5847 | 2020 | 44039 | Common, J; Walker-Sunderhauf, D; van Houte, S; Westra, ER | 2020 | Diversity in CRISPR-based immunity protects susceptible genotypes by restricting phage spread and evolution | Diversity in host resistance often associates with reduced pathogen spread. This may result from ecological and evolutionary processes, likely with feedback between them. Theory and experiments on bacteria-phage interactions have shown that genetic diversity of the bacterial adaptive immune system can limit phage evolution to overcome resistance. Using the CRISPR-Cas bacterial immune system and lytic phage, we engineered a host-pathogen system where each bacterial host genotype could be infected by only one phage genotype. With this model system, we explored how CRISPR diversity impacts the spread of phage when they can overcome a resistance allele, how immune diversity affects the evolution of the phage to increase its host range and if there was feedback between these processes. We show that increasing CRISPR diversity benefits susceptible bacteria via a dilution effect, which limits the spread of the phage. We suggest that this ecological effect impacts the evolution of novel phage genotypes, which then feeds back into phage population dynamics. | NA | yes | Looks promising | Excluded because artificial engineered system. Similar rationale used for excluding domestic species | no | no |
| 5848 | 2020 | 44040 | Fratini, S; Natali, C; Zanet, S; Iannucci, A; Capizzi, D; Sinibaldi, I; Sposimo, P; Ciofi, C | 2020 | Assessment of rodenticide resistance, eradication units, and pathogen prevalence in black rat populations from a Mediterranean biodiversity hotspot (Pontine Archipelago) | Biological invasions are a growing threat to biodiversity. The black rat, one of the worst pest in the world, is responsible for extensive population decline of many autochthonous and endemic species, particularly in island ecosystems. A number of rat eradication campaigns have been conducted, however, such endeavors do not always result in a complete removal of the pest. This may be due to the occurrence of individuals resistant to common rodenticides and/or a re-invasion of the same environment from interconnected areas when appropriate eradication units are not defined before starting an eradication campaign. Our study is a multidisciplinary approach whereby genetic and epidemiological methods were used to provide background information for successful eradication of black rats. We investigated the occurrence of mutations in the VKORC1 gene known to confer resistance to rodenticides and evaluated the spread of zoonoses across three islands of the Pontine Archipelago, an Italian hotspot of endemic Mediterranean biodiversity and a possible mainland source of invasion. As part of an eradication campaign, we also assessed patterns of genetic diversity at 10 microsatellite loci in order to identify eradication units. We recorded a strong population structure and revealed at least two distinct eradication units. Some degree of admixture was recorded on Ponza, the largest island and likely the main source of rats invading the other two islands. We did not record the occurrence of rats resistant to anticoagulants, but we revealed transmission of vector-borne pathogens in commensal habitats of the Archipelago. | NA | yes | looks like GD and fitness data can be collected from 3 populations(islands) - make sure on group level and not ind level? | Limited number of comparisons (r for 4 pops, parasite data for 3) with very similar relatedness values "Average pairwise relatedness was r = 0.074 for | no | no |
| 5849 | 2020 | 44039 | Bolnick, DI; Resetarits, EJ; Ballare, K; Stuart, YE; Stutz, WE | 2020 | Host patch traits have scale-dependent effects on diversity in a stickleback parasite metacommunity | Many metacommunities are distributed across habitat patches that are themselves aggregated into groups. Perhaps the clearest example of this nested metacommunity structure comes from multi-species parasite assemblages, which occupy individual hosts that are aggregated into host populations. At both spatial scales, we expect parasite community diversity in a given patch (either individual host or population) to depend on patch characteristics that affect colonization rates and species sorting. But, are these patch effects consistent across spatial scales? Or, do different processes govern the distribution of parasite community diversity among individual hosts, versus among host patches? To answer these questions, we document the distribution of parasite richness among host individuals and among populations in a metapopulation of threespine sticklebackGasterosteus aculeatus. We find some host traits (host size, gape width) are associated with increased parasite richness at both spatial scales. Other patch characteristics affect parasite richness only among individuals (sex), or among populations (lake size, lake area, elevation and population mean heterozygosity). These results demonstrate that some rules governing parasite richness in this metacommunity are shared across scales, while others are scale-specific. | NA | yes | looks promising | looks at parasite diversity not host diversity | no | no |
| 5850 | 2020 | 44040 | Williams, ST; Haas, CA; Roberts, JH; Taylor, SS | 2020 | Depauperate major histocompatibility complex variation in the endangered reticulated flatwoods salamander (Ambystoma bishopi) | Reticulated flatwoods salamander (Ambystoma bishopi) populations began decreasing dramatically in the 1900s. Contemporary populations are small, isolated, and may be susceptible to inbreeding and reduced adaptive potential because of low genetic variation. Genetic variation at immune genes is especially important as it influences disease susceptibility and adaptation to emerging infectious pathogens, a central conservation concern for declining amphibians. We collected samples from across the extant range of this salamander to examine genetic variation at major histocompatibility complex (MHC) class I alpha and II beta exons as well as the mitochondrial control region. We screened tail or toe tissue for ranavirus, a pathogen associated with amphibian declines worldwide. Overall, we found low MHC variation when compared to other amphibian species and did not detect ranavirus at any site. MHC class I alpha sequencing revealed only three alleles with a nucleotide diversity of 0.001, while MHC class II beta had five alleles with a with nucleotide diversity of 0.004. However, unique variation still exists across this species’ range with private alleles at three sites. Unlike MHC diversity, mitochondrial variation was comparable to levels estimated for other amphibians with nine haplotypes observed, including one haplotype shared across all sites. We hypothesize that a combination of a historic disease outbreak and a population bottleneck may have contributed to low MHC diversity while maintaining higher levels of mitochondrial DNA variation. Ultimately, MHC data indicated that the reticulated flatwoods salamander may be at an elevated risk from infectious diseases due to low levels of immunogenetic variation necessary to combat novel pathogens. | NA | yes | any good fitness measure to combine with diversity measure? See tables | no measure of relatednes | no | no |
| 5851 | 2020 | 44112 | Ferrari, M; Lindholm, AK; Konig, B | 2019 | Fitness Consequences of Female Alternative Reproductive Tactics in House Mice (Mus musculus domesticus) | Alternative reproductive tactics (ARTs) are defined as discrete differences in morphological, physiological, and/or behavioral traits associated with reproduction that occur within the same sex and population. House mice provide a rare example of ARTs in females, which can rear their young either solitarily or together with one or several other females in a communal nest. We assessed the fitness consequences of communal and solitary breeding in a wild population to understand how the two tactics can be evolutionarily stable. Females switched between the two tactics (with more than 50% of all females having two or more litters using both tactics), pointing toward communal and solitary breeding being two tactics within a single strategy and not two genetically determined strategies. Communal breeding resulted in reduced pup survival and negatively impacted female reproductive success. Older and likely heavier females more often reared their litters solitarily, indicating that females use a condition-dependent strategy. Solitary breeding seems the more successful tactic, and only younger and likely less competitive females might opt for communal nursing, even at the cost of increased pup mortality. This study emphasizes the importance of analyzing phenotypic plasticity and its role in cooperation in the context of female ARTs. | NA | yes | can the group gd be compared as low and high? | Only look at relatedness between 2 communally nesting females and offspring survival | no | no |
| 5852 | 2020 | 44039 | Abdi, MK; Lupi, D; Hardy, ICW | 2020 | Co-foundress confinement elicits kinship effects in a naturally sub-social parasitoid | Kinship among interacting individuals is often associated with sociality and also with sex ratio effects. Parasitoids in the bethylid genus Goniozus are sub-social, with single foundress females exhibiting post-ovipositional maternal care via short-term aggressive host and brood defence against conspecific females. Due to local mate competition (LMC) and broods normally being produced by a single foundress, sex ratios are female-biased. Contests between adult females are, however, not normally fatal, and aggression is reduced when competing females are kin, raising the possibility of multi-foundress reproduction on some hosts. Here, we screen for further life-history effects of kinship by varying the numbers and relatedness of foundresses confined together with a host resource and also by varying the size of host. We confined groups of 1-8 Goniozus nephantidis females together with a host for 5+ days. Multi-foundress groups were either all siblings or all nonsiblings. Our chief expectations included that competition for resources would be more intense among larger foundress groups but diminished by both larger host size and closer foundress relatedness, affecting both foundress mortality and reproductive output. From classical LMC theory, we expected that offspring group sex ratios would be less female-biased when there were more foundresses, and from extended LMC theory, we expected that sex ratios would be more female-biased when foundresses were close kin. We found that confinement led to the death of some females (11% overall) but only when host resources were most limiting. Mortality of foundresses was less common when foundresses were siblings. Developmental mortality among offspring was considerably higher in multi-foundress clutches but was unaffected by foundress relatedness. Groups of sibling foundresses collectively produced similar numbers of offspring to nonsibling groups. There was little advantage for individual females to reproduce in multi-foundress groups: single foundresses suppressed even the largest hosts presented and had the highest per capita production of adult offspring. Despite single foundress reproduction being the norm, G. nephantidis females in multi-foundress groups appear to attune sex allocation according to both foundress number and foundress relatedness: broods produced by sibling foundresses had sex ratios similar to broods produced by single foundresses (ca. 11% males), whereas the sex ratios of broods produced by nonsibling females were approximately 20% higher and broadly increased with foundress number. We conclude that relatedness and host size may combine to reduce selection against communal reproduction on hosts and that, unlike other studied parasitoids, G. nephantidis sex ratios conform to predictions of both classical and extended LMC theories. | NA | yes | Looks promising | NA | no | yes |
| 5853 | 2020 | 44040 | de Morais, CT; Kettle, CJ; Philipson, CD; Maycock, CR; Burslem, DFRP; Khoo, E; Ghazoul, J | 2020 | Exploring the role of genetic diversity and relatedness in tree seedling growth and mortality: A multispecies study in a Bornean rainforest | Where conspecific seedlings occur at high densities, density-dependent processes tend to depress their performance and survival relative to co-occurring heterospecifics. We extend this observation to within-species genetic diversity and relatedness. We posit that seedling growth and survival increase where there is higher genetic diversity, and lower relatedness, among seedling populations, under the expectation that increased genetic dissimilarity among conspecific seedlings affords greater resistance to pathogens. We used estimates of individual seedling genetic diversity (multilocus heterozygosity (sMLH)) and genetic relatedness among conspecific seedlings (pairwise kinship coefficients (LRI)), under high and low conspecific seedling density, coupled with censuses of seedling growth and mortality over 30 months to explore the role of genetic diversity and relatedness on growth and mortality of 1,485 seedlings of four dipterocarp species in an undisturbed Bornean tropical rainforest. We hypothesized that more genetically diverse and less related co-occurring seedlings would display higher survival and growth rates. In three of our four species, lower genetic diversity increased the probability of mortality over 30 months. We observed no effect of genetic diversity on relative growth rates. Contrary to our expectations, only one species showed a negative effect of increased relatedness on mortality. In two of the four species studied, seedlings that were more genetically related to neighbouring conspecific seedlings in the sample plot were less likely to die, but grew more slowly. Synthesis. Our results confirm that genetic diversity and relatedness among seedlings shape survival probabilities differentially across species. In contrast, we found no differences in mortality and growth rates between plots with low and high seedling density. Our results suggest that a greater abundance of pollen donors could contribute to cohort seedling survival in some species, though not all, and the specific mechanisms by which genetic relatedness determines seedling demography remain uncertain. These results have conservation and management implications in view of changing patterns of gene flow in fragmented, exploited and degraded tropical rainforests, which might differentially influence natural regeneration among species. | NA | yes | Is the relatedness they use OK measure? | NA | no | yes |
| 5854 | 2020 | 44112 | Parsche, S; Lattorff, HMG | 2018 | The relative contributions of host density and genetic diversity on prevalence of a multi-host parasite in bumblebees | The role of population and demographic factors in influencing the transmission and establishment parasites infecting multiple hosts is poorly understood. We assessed the effects of these factors on parasite prevalence in a model system - the intestinal protozoan Crithidia bombi (Trypanosomatidae) infecting a range of bumblebee species (Bombus spp.). We used microsatellite markers and sibship reconstruction to infer genetic diversity and the density of host populations to infer their relative contributions to parasite prevalence. We established the prevalence, type (single-vs. multiple-strain infection) and intensity of C. bombi infections in workers and males of three common bumblebee species (B. terrestris, B. lapidarius and B. pascuorum) at 30 locations across Germany. We found evidence that colony density promoted prevalence, while increased genetic diversity lowered prevalence in B. terrestris. The effect size for genetic diversity was much larger than for colony density. Thus, genetic factors affected the prevalence, while demographic and life-history traits (e.g. population density and seasonal cycle of development) were additional factors shaping the spread and establishment of a multi-host parasite. Bombus lapidarius possessed characteristics that indicate it might act as a reservoir species spreading disease to other species. | NA | yes | Looks promising | NA | no | yes |
| 5855 | OwnStudy | NA | Bensch, H; Cornwallis, C. K. | NA | H bensch Master’s thesis: unpub data, see suppdata table S18 | NA | NA | yes | NA | NA | no | yes |
| 5858 | references | from ref check | Strauss, Alexander; Hite, Jessica; Shocket, Marta; Caceres, Carla; Duffy, Meghan; Hall, Spencer | 2017 | Rapid evolution rescues hosts from competition and disease but—despite a dilution effect—increases the density of infected hosts | Virulent parasites can depress the densities of their hosts. Taxa that reduce disease via dilution effects might alleviate this burden. However, ‘diluter’ taxa can also depress host densities through competition for shared resources. The combination of disease and interspecific competition could even drive hosts extinct. Then again, genetically variable host populations can evolve in response to both competitors and parasites. Can rapid evolution rescue host density from the harm caused by these ecological enemies? How might such evolution influence dilution effects or the size of epidemics? In a mesocosm experiment with planktonic hosts, we illustrate the joint harm of competition and disease: hosts with constrained evolutionary ability (limited phenotypic variation) suffered greatly from both. However, populations starting with broader phenotypic variation evolved stronger competitive ability during epidemics. In turn, enhanced competitive ability—driven especially by parasites—rescued host densities from the negative impacts of competition, disease, and especially their combination. Interspecific competitors reduced disease (supporting dilution effects) even when hosts rapidly evolved. However, this evolutionary response also elicited a potential problem. Populations that evolved enhanced competitive ability and maintained robust total densities also supported higher densities of infections. Thus, rapid evolution rescued host densities but also unleashed larger epidemics. | NA | yes | from Ekroth paper | NA | no | yes |
| 5859 | references | from ref check | Agashe, Deepa | 2009 | The Stabilizing Effect of Intraspecific Genetic Variation on Population Dynamics in Novel and Ancestral Habitats | Recent studies show that intraspecific genetic variation in asexual species may have large effects on community and ecosystem functions, increasing their stability, productivity, and species richness. However, major questions regarding its population‐level impact remain empirically unanswered: (a) How does intraspecific genetic diversity affect the ecological characteristics of sexual species, in which recombination can alter the outcome of causal mechanisms such as selection and niche diversification? (b) Does genetic diversity increase population dynamic stability? (c) Is the impact of genetic diversity dependent on the selective environment? To answer these questions, I founded replicate flour beetle (Tribolium castaneum) populations with different degrees of ecologically relevant, heritable trait variation and monitored their dynamics for approximately eight generations. I show that population stability and persistence increased with greater genetic variation but that the stabilizing effect was independent of the selective habitat (different proportions of ancestral and novel resources). Alleles from a single founding strain underwent a selective sweep in the homogeneous ancestral habitat but not in a novel heterogeneous habitat. These results expand current understanding of the ecological impacts of genetic diversity by showing that genetically more diverse sexual populations persist longer and are more stable but that the selective environment determines the mechanistic basis of increased stability | NA | yes | from ref check | NA | no | yes |
| 5860 | references | from ref check | Anton, Christian; Zeisset, Inga; Musche, Martin; et al. | 2007 | Population structure of a large blue butterfly and its specialist parasitoid in a fragmented landscape | Habitat fragmentation may interrupt trophic interactions if herbivores and their specific parasitoids respond differently to decreasing connectivity of populations. Theoretical models predict that species at higher trophic levels are more negatively affected by isolation than lower trophic level species. By combining ecological data with genetic information from microsatellite markers we tested this hypothesis on the butterfly Maculinea nausithous and its specialist hymenopteran parasitoid Neotypus melanocephalus. We assessed the susceptibility of both species to habitat fragmentation by measuring population density, rate of parasitism, overall genetic differentiation (θST) and allelic richness in a large metapopulation. We also simulated the dynamics of genetic differentiation among local populations to asses the relative effects of migration rate, population size, and haplodiploid (parasitoid) and diploid (host) inheritance on metapopulation persistence. We show that parasitism by N. melanocephalus is less frequent at larger distances to the nearest neighbouring population of M. nausithous hosts, but that host density itself is not affected by isolation. Allelic richness was independent of isolation, but the mean genetic differentiation among local parasitoid populations increased with the distance between these populations. Overall, genetic differentiation in the parasitoid wasp was much greater than in the butterfly host and our simulations indicate that this difference is due to a combination of haplodiploidy and small local population sizes. Our results thus support the hypothesis that Neotypus parasitoid wasps are more sensitive to habitat fragmentation than their Maculinea butterfly hosts. | NA | yes | from ref check | NA | no | yes |
| 5861 | references | from ref check | Cook-Patton, Susan C.; McArt, Scott H.; Parachnowitsch, Amy L.; et al. | 2011 | A direct comparison of the consequences of plant genotypic and species diversity on communities and ecosystem function | Biodiversity loss is proceeding at an unprecedented rate, yet we lack a thorough understanding of the consequences of losing diversity at different scales. While species diversity is known to impact community and ecosystem processes, genotypic diversity is assumed to have relatively smaller effects. Nonetheless, a few recent studies suggest that genotypic diversity may have quantitatively similar ecological consequences compared to species diversity. Here we show that increasing either genotypic diversity of common evening primrose (Oenothera biennis) or species diversity of old‐field plant species resulted in nearly equivalent increases (∼17%) in aboveground primary production. The predominant mechanism explaining this effect, niche complementarity, was similar for each type of diversity. Arthropod species richness also increased with both types of plant diversity, but the mechanisms leading to this effect differed: abundance‐driven accumulation of arthropod species was important in plant genotypic polycultures, whereas resource specialization was important in plant species polycultures. Thus, similar increases in primary productivity differentially impacted higher trophic levels in response to each type of plant diversity. These results highlight important ecological similarities and differences between genotypic and species diversity and suggest that genotypic diversity may play a larger role in community and ecosystem processes than previously realized. | NA | yes | from ref check | NA | no | yes |
| 5862 | references | from ref check | Crutsinger, Gregory M.; Collins, Michael D.; Fordyce, James A.; et al. | 2006 | Plant genotypic diversity predicts community structure and governs an ecosystem process | Theory predicts, and recent empirical studies have shown, that the diversity of plant species determines the diversity of associated herbivores and mediates ecosystem processes, such as aboveground net primary productivity (ANPP). However, an often-overlooked component of plant diversity, namely population genotypic diversity, may also have wide-ranging effects on community structure and ecosystem processes. We showed experimentally that increasing population genotypic diversity in a dominant old-field plant species, Solidago altissima, determined arthropod diversity and community structure and increased ANPP. The effects of genotypic diversity on arthropod diversity and ANPP were comparable to the effects of plant species diversity measured in other studies. | NA | yes | from ref check | NA | no | yes |
| 5863 | references | from ref check | Crutsinger, Gregory M.; Reynolds, W. Nicholas; Classen, Aimee T.; et al. | 2008 | Disparate effects of plant genotypic diversity on foliage and litter arthropod communities | IntraspeciWc diversity can inXuence the structure of associated communities, though whether litter-based and foliage-based arthropod communities respond to intraspeciWc diversity in similar ways remains unclear. In this study, we compared the eVects of host-plant genotype and genotypic diversity of the perennial plant, Solidago altissima, on the arthropod community associated with living plant tissue (foliage-based community) and microarthropods associated with leaf litter (litter-based community). We found that variation among host-plant genotypes had strong eVects on the diversity and composition of foliagebased arthropods, but only weak eVects on litter-based microarthropods. Furthermore, host-plant genotypic diversity was positively related to the abundance and diversity of foliage-based arthropods, and within the herbivore and predator trophic levels. In contrast, there were minimal eVects of plant genotypic diversity on litter-based microarthropods in any trophic level. Our study illustrates that incorporating communities associated with living foliage and senesced litter into studies of community genetics can lead to very diVerent conclusions about the importance of intraspeciWc diversity than when only foliage-based community responses are considered in isolation. | NA | yes | from ref check | NA | no | yes |
| 5864 | references | from ref check | Crutsinger, Gregory M.; Reynolds, W. Nicholas; Classen, Aimee T.; et al. | 2008 | Temporal dynamics in non-additive responses of arthropods to host-plant genotypic diversity | Genotypic diversity within host‐plant populations has been linked to the diversity of associated arthropod communities, but the temporal dynamics of this relationship, along with the underlying mechanisms, are not well understood. In this study, we employed a common garden experiment that manipulated the number of genotypes within patches of Solidago altissima, tall goldenrod, to contain 1, 3, 6 or 12 genotypes m−2 and measured both host‐plant and arthropod responses to genotypic diversity throughout an entire growing season. Despite substantial phenological changes in host plants and in the composition of the arthropod community, we detected consistent positive responses of arthropod diversity to host‐plant genotypic diversity throughout all but the end of the growing season. Arthropod richness and abundance increased with genotypic diversity by up to∼65%. Furthermore, arthropod responses were non‐additive for most of the growing season, with up to 52% more species occurring in mixtures than the number predicted by summing the number of arthropods associated with component genotypes in monoculture. Non‐additive arthropod responses were likely driven by concurrent non‐additive increases in host‐plant aboveground biomass. Qualitative differences among host‐plant genotypes were also important early in the season, when specialist herbivores dominated the arthropod community. Neither arthropod diversity nor flower number was associated with genotypic diversity at the end of the growing season, when generalist floral‐associated herbivores dominated. Taken together, these results show that focusing on the temporal dynamics in the quantity and quality of co‐occurring host‐plant genotypes and associated community composition can help uncover the mechanisms that link intraspecific host‐plant diversity to the structure of arthropod communities. Furthermore, consistent non‐additive effects in genotypically diverse plots may limit the predictability of the arthropod community based solely on the genetic make‐up of a host‐plant patch. | NA | yes | from ref check | NA | no | yes |
| 5865 | references | from ref check | Ellison, Amy; Cable, Jo; Consuegra, Sofia | 2011 | Best of both worlds? Association between outcrossing and parasite loads in a selfing fish | Mixed‐mating strategies (i.e., intermediate levels of self‐fertilization and outcrossing in hermaphrodites) are relatively common in plants and animals, but why self‐fertilization (selfing) rates vary so much in nature has proved difficult to explain. We tested the hypothesis that parasites help maintain mixed‐mating using a partially selfing fish (Kryptolebias marmoratus) as a model. We show that outcrossed progeny in the wild are genetically more diverse and less susceptible to multiple parasite infections than their selfed counterparts. Given that outcrossing in K. marmoratus can only be attained by male‐hermaphrodite matings, our data provide an explanation for the coexistence of males and hermaphrodites in androdioecious species where hermaphrodites are unable to outcross among themselves. Moreover, our study provides evidence that parasites contribute to maintaining mixed‐mating in a natural animal population. | NA | yes | from ref check | NA | no | yes |
| 5866 | references | from ref check | Hughes, AR; Stachowicz, JJ | 2004 | Genetic diversity enhances the resistance of a seagrass ecosystem to disturbance | Motivated by recent global reductions in biodiversity, empirical and theoretical research suggests that more species-rich systems exhibit enhanced productivity, nutrient cycling, or resistance to disturbance or invasion relative to systems with fewer species. In contrast, few data are available to assess the potential ecosystem-level importance of genetic diversity within species known to play a major functional role. Using a manipulative field experiment, we show that increasing genotypic diversity in a habitat-forming species (the seagrass Zostera marina) enhances community resistance to disturbance by grazing geese. The time required for recovery to near predisturbance densities also decreases with increasing eelgrass genotypic diversity. However, there is no effect of diversity on resilience, measured as the rate of shoot recovery after the disturbance, suggesting that more rapid recovery in diverse plots is due solely to differences in disturbance resistance. Genotypic diversity did not affect ecosystem processes in the absence of disturbance. Thus, our results suggest that genetic diversity, like species diversity, may be most important for enhancing the consistency and reliability of ecosystems by providing biological insurance against environmental change. | NA | yes | from ref check | NA | no | yes |
| 5867 | references | from ref check | Johnson, MTJ; Lajeunesse, MJ; Agrawal, AA | 2006 | Additive and interactive effects of plant genotypic diversity on arthropod communities and plant fitness | Recent research suggests that genetic diversity in plant populations can shape the diversity and abundance of consumer communities. We tested this hypothesis in a field experiment by manipulating patches of Evening Primrose (Oenothera biennis) to contain one, four or eight plant genotypes. We then surveyed 92 species of naturally colonizing arthropods. Genetically diverse plant patches had 18% more arthropod species, and a greater abundance of omnivorous and predacious arthropods, but not herbivores, compared with monocultures. The effects of genotypic diversity on arthropod communities were due to a combination of interactive and additive effects among genotypes within genetically diverse patches. Greater genetic diversity also led to a selective feedback, as mean genotype fitness was 27% higher in diverse patches than in monocultures. A comparison between our results and the literature reveals that genetic diversity and species diversity can have similar qualitative and quantitative effects on arthropod communities. Our findings also illustrate the benefit of preserving genetic variation to conserve species diversity and interactions within multitrophic communities. | NA | yes | from ref check | NA | no | yes |
| 5868 | references | from ref check | Liersch and Schmid-Hempel | 1998 | Genetic variation within social insect colonies reduces parasite load | In colonies of social Hymenoptera (ants, bees and wasps), workers are often not very closely related to each other, because queens mate with several different males (polyandry) or because several functional queens are present (polygyny). Both characteristics increase genetic variation among the queens’ reproductive and worker offspring, but the benefits of this increased variation remain obscure. Here we report an experiment where genetically homogeneous and genetically heterogeneous colonies of the bumble bee, Bombus terrestris, have been exposed to parasitism under field conditions. Colonies of high or low genetic variation were achieved by adding and removing brood from donor colonies. The results showed a consistent effect in that genetically variable colonies experienced reduced parasite loads, i.e. lower prevalence, intensity and parasite species richness, for a range of protozoa, nematodes, mites or parasitoids affecting the workers. We therefore propose that polyandry and/or polygyny of social insects may be beneficial under parasitism. | NA | yes | from ref check | NA | no | yes |
| 5869 | references | from ref check | McLeod, Laura; Marshall, Dustin J. | 2009 | Do genetic diversity effects drive the benefits associated with multiple mating? A test in a marine invertebrate | Background Mothers that mate with multiple males often produce higher quality offspring than mothers that mate with a single male. By engaging in polyandry, mothers may increase their chances of mating with a compatible male or promote sperm competition - both of which act to increase maternal fitness via the biasing of the paternity of offspring. Surprisingly, mating with multiple males, can carry benefits without biasing paternity and may be due simply to differences in genetic diversity between monandrous and polyandrous clutches but role of genetic diversity effects in driving the benefits of polyandry remains poorly tested. Disentangling indirect, genetic benefits from genetic diversity effects is challenging but crucial if we are to understand the selection pressures acting to promote polyandry.      Methodology/Principal Findings Here, we examine the post-fertilisation benefits of accessing the sperm of multiple males in an externally fertilising polychaete worm. Accessing the sperm of multiple males increases offspring performance but this benefit was driven entirely by genetic diversity effects and not by the biasing of paternity at fertilisation.  Conclusions/Significance Previous studies on polyandry should be interpreted cautiously as genetic diversity effects alone can explain the benefits of polyandry yet these diversity effects may be difficult to disentangle from other mechanisms. We suggest that future studies use a modified experimental design in order to discriminate between genetic diversity effects and indirect, genetic benefits. Background Mothers that mate with multiple males often produce higher quality offspring than mothers that mate with a single male. By engaging in polyandry, mothers may increase their chances of mating with a compatible male or promote sperm competition - both of which act to increase maternal fitness via the biasing of the paternity of offspring. Surprisingly, mating with multiple males, can carry benefits without biasing paternity and may be due simply to differences in genetic diversity between monandrous and polyandrous clutches but role of genetic diversity effects in driving the benefits of polyandry remains poorly tested. Disentangling indirect, genetic benefits from genetic diversity effects is challenging but crucial if we are to understand the selection pressures acting to promote polyandry.  Methodology/Principal Findings Here, we examine the post-fertilisation benefits of accessing the sperm of multiple males in an externally fertilising polychaete worm. Accessing the sperm of multiple males increases offspring performance but this benefit was driven entirely by genetic diversity effects and not by the biasing of paternity at fertilisation.  Conclusions/Significance Previous studies on polyandry should be interpreted cautiously as genetic diversity effects alone can explain the benefits of polyandry yet these diversity effects may be difficult to disentangle from other mechanisms. We suggest that future studies use a modified experimental design in order to discriminate between genetic diversity effects and indirect, genetic benefits. Background Mothers that mate with multiple males often produce higher quality offspring than mothers that mate with a single male. By engaging in polyandry, mothers may increase their chances of mating with a compatible male or promote sperm competition - both of which act to increase maternal fitness via the biasing of the paternity of offspring. Surprisingly, mating with multiple males, can carry benefits without biasing paternity and may be due simply to differences in genetic diversity between monandrous and polyandrous clutches but role of genetic diversity effects in driving the benefits of polyandry remains poorly tested. Disentangling indirect, genetic benefits from genetic diversity effects is challenging but crucial if we are to understand the selection pressures acting to promote polyandry.  Methodology/Principal Findings Here, we examine the post-fertilisation benefits of accessing the sperm of multiple males in an externally fertilising polychaete worm. Accessing the sperm of multiple males increases offspring performance but this benefit was driven entirely by genetic diversity effects and not by the biasing of paternity at fertilisation.  Conclusions/Significance Previous studies on polyandry should be interpreted cautiously as genetic diversity effects alone can explain the benefits of polyandry yet these diversity effects may be difficult to disentangle from other mechanisms. We suggest that future studies use a modified experimental design in order to discriminate between genetic diversity effects and indirect, genetic benefits. Background Mothers that mate with multiple males often produce higher quality offspring than mothers that mate with a single male. By engaging in polyandry, mothers may increase their chances of mating with a compatible male or promote sperm competition - both of which act to increase maternal fitness via the biasing of the paternity of offspring. Surprisingly, mating with multiple males, can carry benefits without biasing paternity and may be due simply to differences in genetic diversity between monandrous and polyandrous clutches but role of genetic diversity effects in driving the benefits of polyandry remains poorly tested. Disentangling indirect, genetic benefits from genetic diversity effects is challenging but crucial if we are to understand the selection pressures acting to promote polyandry.  Methodology/Principal Findings Here, we examine the post-fertilisation benefits of accessing the sperm of multiple males in an externally fertilising polychaete worm. Accessing the sperm of multiple males increases offspring performance but this benefit was driven entirely by genetic diversity effects and not by the biasing of paternity at fertilisation. Conclusions/Significance Previous studies on polyandry should be interpreted cautiously as genetic diversity effects alone can explain the benefits of polyandry yet these diversity effects may be difficult to disentangle from other mechanisms. We suggest that future studies use a modified experimental design in order to discriminate between genetic diversity effects and indirect, genetic benefits. | NA | yes | from ref check | NA | no | yes |
| 5870 | references | from ref check | Page Jr et al. | 1995 | Effects of worker genotypic diversity on honey bee colony development and behavior (Apis mellifera L.) | There have been numerous reports of genetic influences on division of labor in honey bee colonies, but the effects of worker genotypic diversity on colony behavior are unclear. We analyzed the effects of worker genotypic diversity on the phenotypes of honey bee colonies during a critical phase of colony development, the “nest initiation” phase. Five groups of colonies were studied (n = 5–11 per group); four groups had relatively low genotypic diversity compared to the fifth group. Colonies were derived from queens that were instrumentally inseminated with the semen of four different drones according to one of the following mating schemes: group A, 4 A-source drones; group B, 4 B-source drones; group C, 4 C-source drones; group D, 4 D-source drones; and group E, 1 drone of each of the A-D drone sources. There were significant differences between colonies in groups A-D for 8 out of 19 colony traits. Because the queens in all of these colonies were super sisters, the observed differences between groups were primarily a consequence of differences in worker genotypes. There were very few differences (2 out of 19 traits) between colonies with high worker genotypic diversity (group E) and those with low diversity (groups A-D combined). This is because colonies with greater diversity tended to have phenotypes that were average relative to colonies with low genotypic diversity. We hypothesize that the averaging effect of genotypic variability on colony phenotypes may have selective advantages, making colonies less likely to “fail” because of inappropriate colony responses to changing environmental conditions. | NA | yes | from ref check | NA | no | yes |
| 5871 | references | from ref check | Parker, John D.; Salminen, Juha-Pekka; Agrawal, Anurag A. | 2010 | Herbivory enhances positive effects of plant genotypic diversity | Both plant diversity and vertebrate herbivores can impact plant fitness and ecosystem functioning, however their interactions have not been explicitly tested. We manipulated plant genotypic diversity of the native plant Oenothera biennis and monitored its survivorship and lifetime fitness with and without one of its major vertebrate consumers, white‐tailed deer Odocoileus virginianus. Intense but unmanipulated herbivory by meadow voles Microtus pennsylvanicus killed over 70% of nearly 4000 experimental plants. However, plants grown in genotypically diverse patches suffered fewer vole attacks and had higher survival and reproductive output than plants in monoculture. Moreover, positive effects of genotypic diversity were enhanced by the presence of deer, indicating a non‐additive interaction between diversity and trophic‐level complexity. Genetic selection analyses showed that the selective value of ecologically important traits depended on plant diversity and exposure to deer, demonstrating that community complexity can promote fitness through multiple ecologically and evolutionarily important feedbacks. | NA | yes | from ref check | NA | yes | yes |
| 5872 | references | from ref check | Robinson, John D. Et al. | 2013 | Extinction hazards in experimental Daphnia magna populations: Effects of genotype diversity and environmental variation | Extinction is ubiquitous in natural systems and the ultimate fate of all biological populations. However, the factors that contribute to population extinction are still poorly understood, particularly genetic diversity and composition. A laboratory experiment was conducted to examine the influences of environmental variation and genotype diversity on persistence in experimental Daphnia magna populations. Populations were initiated in two blocks with one, two, three, or six randomly selected and equally represented genotypes, fed and checked for extinction daily, and censused twice weekly over a period of 170 days. Our results show no evidence for an effect of the number of genotypes in a population on extinction hazard. Environmental variation had a strong effect on hazards in both experimental blocks, but the direction of the effect differed between blocks. In the first block, variable environments hastened extinction, while in the second block, hazards were reduced under variable food input. This occurred despite greater fluctuations in population size in variable environments in the second block of our experiment. Our results conflict with previous studies, where environmental variation consistently increased extinction risk. They are also at odds with previous studies in other systems that documented significant effects of genetic diversity on population persistence. We speculate that the lack of sexual reproduction, or the phenotypic similarity among our experimental lines, might underlie the lack of a significant effect of genotype diversity in our study. | NA | yes | from ref check | NA | no | yes |
| 5873 | references | from ref check | Trouve, S; Degen, L; Renaud, F; et al. | 2003 | Evolutionary implications of a high selfing rate in the freshwater snail Lymnaea truncatula | Extinction is ubiquitous in natural systems and the ultimate fate of all biological populations. However, the factors that contribute to population extinction are still poorly understood, particularly genetic diversity and composition. A laboratory experiment was conducted to examine the influences of environmental variation and genotype diversity on persistence in experimental Daphnia magna populations. Populations were initiated in two blocks with one, two, three, or six randomly selected and equally represented genotypes, fed and checked for extinction daily, and censused twice weekly over a period of 170 days. Our results show no evidence for an effect of the number of genotypes in a population on extinction hazard. Environmental variation had a strong effect on hazards in both experimental blocks, but the direction of the effect differed between blocks. In the first block, variable environments hastened extinction, while in the second block, hazards were reduced under variable food input. This occurred despite greater fluctuations in population size in variable environments in the second block of our experiment. Our results conflict with previous studies, where environmental variation consistently increased extinction risk. They are also at odds with previous studies in other systems that documented significant effects of genetic diversity on population persistence. We speculate that the lack of sexual reproduction, or the phenotypic similarity among our experimental lines, might underlie the lack of a significant effect of genotype diversity in our study. | NA | yes | from ref check | NA | no | yes |

## **Table S2:** Data used for analysis of the effect of within-group relatedness on mortality and pathogen abundance (r).

| Study | Author | Year | Title | Journal | Species | Data source | Analysis | Effect size Rscript reference | Pathogen present | Pathogen manipulated | Relatedness manipulated | Measurement | Life stage | Relatedness measure | Kin structure | Lab | Possible Inbreeding | NlowR | NhighR | Ntotal | r |
| --- | --- | --- | --- | --- | --- | --- | --- | --- | --- | --- | --- | --- | --- | --- | --- | --- | --- | --- | --- | --- | --- |
| 1 | Cook-Patton, Susan C.; McArt, Scott H.; Parachnowitsch, Amy L.; et al. | 2011 | A direct comparison of the consequences of plant genotypic and species diversity on communities and ecosystem function | Ecology | Oenothera biennis | fig 2 and appendix | ANOVA | NA | yes | obs | exp | Parasitism | adult | genotypes | Kin | no | no | 69 | 46 | 115 | -0.190 |
| 1 | Cook-Patton, Susan C.; McArt, Scott H.; Parachnowitsch, Amy L.; et al. | 2011 | A direct comparison of the consequences of plant genotypic and species diversity on communities and ecosystem function | Ecology | Oenothera biennis | fig 2 and appendix | ANOVA | NA | yes | obs | exp | Parasitism | adult | genotypes | Kin | no | no | 69 | 46 | 115 | -0.213 |
| 2 | Johnson, MTJ; Lajeunesse, MJ; Agrawal, AA | 2006 | Additive and interactive effects of plant genotypic diversity on arthropod communities and plant fitness | Ecology Letters | Oenothera biennis | table 2 herbivores | ANOVA | NA | yes | obs | exp | Parasitism | adult | genotypes | Kin | no | no | NA | NA | 50 | -0.085 |
| 2 | Johnson, MTJ; Lajeunesse, MJ; Agrawal, AA | 2006 | Additive and interactive effects of plant genotypic diversity on arthropod communities and plant fitness | Ecology Letters | Oenothera biennis | table 2 herbivores | ANOVA | NA | yes | obs | exp | Parasitism | adult | genotypes | Kin | no | no | NA | NA | 50 | 0.077 |
| 3 | Ganz, HH; Ebert, D | 2010 | Benefits of host genetic diversity for resistance to infection depend on parasite diversity | Ecology | Daphnia magna | text exp 3 results | Logistic\_Regression | NA | yes | exp | exp | Parasitism | adult | relatedness | Kin | yes | no | 64 | 64 | 128 | 0.221 |
| 4 | Mattila, HR; Rios, D; Walker-Sperling, VE; Roeselers, G; Newton, ILG | 2012 | Characterization of the Active Microbiotas Associated with Honey Bees Reveals Healthier and Broader Communities when Colonies are Genetically Diverse | Plos One | Apis mellifera | table 2 | ChiSq | NA | yes | obs | exp | Parasitism | adult | relatedness | Kin | no | no | 12 | 10 | 22 | 0.283 |
| 4 | Mattila, HR; Rios, D; Walker-Sperling, VE; Roeselers, G; Newton, ILG | 2012 | Characterization of the Active Microbiotas Associated with Honey Bees Reveals Healthier and Broader Communities when Colonies are Genetically Diverse | Plos One | Apis mellifera | table 2 | ChiSq | NA | yes | obs | exp | Parasitism | adult | relatedness | Kin | no | no | 12 | 10 | 22 | 0.283 |
| 4 | Mattila, HR; Rios, D; Walker-Sperling, VE; Roeselers, G; Newton, ILG | 2012 | Characterization of the Active Microbiotas Associated with Honey Bees Reveals Healthier and Broader Communities when Colonies are Genetically Diverse | Plos One | Apis mellifera | table 2 | ChiSq | NA | yes | obs | exp | Parasitism | adult | relatedness | Kin | no | no | 12 | 10 | 22 | 0.169 |
| 4 | Mattila, HR; Rios, D; Walker-Sperling, VE; Roeselers, G; Newton, ILG | 2012 | Characterization of the Active Microbiotas Associated with Honey Bees Reveals Healthier and Broader Communities when Colonies are Genetically Diverse | Plos One | Apis mellifera | table 2 | ChiSq | NA | yes | obs | exp | Parasitism | adult | relatedness | Kin | no | no | 12 | 10 | 22 | 0.283 |
| 5 | Dagan, Y; Liljeroos, K; Jokela, J; Ben-Ami, F | 2013 | Clonal diversity driven by parasitism in a freshwater snail | Journal of Evolutionary Biology | Melanoides tuberculata | table 1 | Pearson\_Correlation | NA | yes | obs | obs | Parasitism | adult | allozyme | Kin | no | yes | NA | NA | 22 | -0.455 |
| 6 | Keeney, DB; King, TM; Rowe, DL; Poulin, R | 2009 | Contrasting mtDNA diversity and population structure in a direct-developing marine gastropod and its trematode parasites | Molecular Ecology | Zeacumantus subcarinatus | table 1 | Pearson\_Correlation | NA | yes | obs | obs | Parasitism | adult | microsatellites | Kin | no | no | NA | NA | 11 | -0.030 |
| 6 | Keeney, DB; King, TM; Rowe, DL; Poulin, R | 2009 | Contrasting mtDNA diversity and population structure in a direct-developing marine gastropod and its trematode parasites | Molecular Ecology | Zeacumantus subcarinatus | table 1 | Pearson\_Correlation | NA | yes | obs | obs | Parasitism | adult | microsatellites | Kin | no | no | NA | NA | 11 | 0.004 |
| 8 | Crutsinger, Gregory M.; Reynolds, W. Nicholas; Classen, Aimee T.; et al. | 2008 | Disparate effects of plant genotypic diversity on foliage and litter arthropod communities | Oecologia | Solidago altissima | table 1 | ANOVA | NA | yes | obs | exp | Parasitism | adult | relatedness | Kin | no | no | NA | NA | 63 | -0.250 |
| 8 | Crutsinger, Gregory M.; Reynolds, W. Nicholas; Classen, Aimee T.; et al. | 2008 | Disparate effects of plant genotypic diversity on foliage and litter arthropod communities | Oecologia | Solidago altissima | table 1 | ANOVA | NA | yes | obs | exp | Parasitism | adult | relatedness | Kin | no | no | NA | NA | 63 | -0.240 |
| 9 | McLeod, Laura; Marshall, Dustin J. | 2009 | Do genetic diversity effects drive the benefits associated with multiple mating? A test in a marine invertebrate | Plos One | Galeolaria caespitosa | exp 2 results fig 3b larvae | Means and SD | NA | no | obs | exp | Mortality | juvenile | relatedness | Non-kin | yes | no | 4 | 4 | 8 | 0.245 |
| 9 | McLeod, Laura; Marshall, Dustin J. | 2009 | Do genetic diversity effects drive the benefits associated with multiple mating? A test in a marine invertebrate | Plos One | Galeolaria caespitosa | exp 1 results fig 2c larvae | ANOVA | NA | no | obs | exp | Mortality | juvenile | relatedness | Non-kin | yes | no | NA | NA | 8 | 0.723 |
| 10 | Hughes, WOH; Boomsma, JJ | 2006 | Does genetic diversity hinder parasite evolution in social insect colonies? | Journal of Evolutionary Biology | Acromyrmex echinatior | from fig 2 | ChiSq | NA | yes | exp | exp | Parasitism | adult | relatedness | Kin | yes | no | 12 | 12 | 24 | 0.092 |
| 10 | Hughes, WOH; Boomsma, JJ | 2006 | Does genetic diversity hinder parasite evolution in social insect colonies? | Journal of Evolutionary Biology | Acromyrmex echinatior | from fig 6b | Means and SD | NA | yes | exp | exp | Parasitism | adult | relatedness | Kin | yes | no | 12 | 12 | 24 | 0.083 |
| 11 | Aguirre, JD; Marshall, DJ | 2012 | Does genetic diversity reduce sibling competition? | Evolution | Ciona intestinalis | fig 2b and text | ANCOVA | NA | yes | obs | exp | Mortality | adult | relatedness | Kin | no | no | NA | NA | 24 | 0.403 |
| 12 | Johansson, M; Primmer, CR; Merila, J | 2007 | Does habitat fragmentation reduce fitness and adaptability? A case study of the common frog (Rana temporaria) | Molecular Ecology | Rana temporaria | text results | Pearson\_Correlation | NA | yes | obs | obs | Mortality | juvenile | microsatellites | Non-kin | no | yes | NA | NA | 8 | 0.700 |
| 13 | WALLS, SC; BLAUSTEIN, AR | 1994 | Does Kinship Influence Density Dependence in a Larval Salamander? | Oikos | Ambystoma opacum | fig 1a high density | Means and SD | NA | yes | obs | exp | Mortality | juvenile | relatedness | Non-kin | no | no | 5 | 5 | 10 | 0.343 |
| 13 | WALLS, SC; BLAUSTEIN, AR | 1994 | Does Kinship Influence Density Dependence in a Larval Salamander? | Oikos | Ambystoma opacum | fig 1a low density | Means and SD | NA | yes | obs | exp | Mortality | juvenile | relatedness | Non-kin | no | no | 5 | 5 | 10 | 0.376 |
| 14 | Byrne, PG; Roberts, JD | 2000 | Does multiple paternity improve fitness of the frog Crinia georgiana? | Evolution | Crinia georgiana | table 1. Wet | Means and SD | NA | no | obs | exp | Mortality | juvenile | relatedness | Non-kin | yes | no | 8 | 8 | 16 | -0.099 |
| 14 | Byrne, PG; Roberts, JD | 2000 | Does multiple paternity improve fitness of the frog Crinia georgiana? | Evolution | Crinia georgiana | table 4. Constant | Means and SD | NA | no | obs | exp | Mortality | juvenile | relatedness | Non-kin | yes | no | 6 | 7 | 13 | -0.285 |
| 14 | Byrne, PG; Roberts, JD | 2000 | Does multiple paternity improve fitness of the frog Crinia georgiana? | Evolution | Crinia georgiana | table 4. Fluctuating | Means and SD | NA | no | obs | exp | Mortality | juvenile | relatedness | Non-kin | yes | no | 5 | 6 | 11 | 0.273 |
| 14 | Byrne, PG; Roberts, JD | 2000 | Does multiple paternity improve fitness of the frog Crinia georgiana? | Evolution | Crinia georgiana | table 1. Dry | Means and SD | NA | no | obs | exp | Mortality | juvenile | relatedness | Non-kin | yes | no | 6 | 8 | 14 | -0.016 |
| 15 | Thonhauser, KE; Raveh, S; Thoss, M; Penn, DJ | 2016 | Does multiple paternity influence offspring disease resistance? | Journal of Evolutionary Biology | Mus musculus | fig 3 and text | Mann\_Whitney | NA | yes | exp | exp | Parasitism | juvenile | relatedness | Kin | yes | no | 15 | 15 | 30 | -0.133 |
| 16 | Byrne, PG; Whiting, MJ | 2011 | Effects of simultaneous Polyandrous on offspring fitness in an African tree frog | Behavioral Ecology | Chiromantis xerampelina | figure 2 | ANOVA | NA | yes | obs | obs | Mortality | juvenile | relatedness | Non-kin | no | no | NA | NA | 20 | 0.491 |
| 17 | Weyrauch, SL; Grubb, TC | 2006 | Effects of the interaction between genetic diversity and UV-B radiation on wood frog fitness | Conservation Biology | Rana sylvatica | fig 2a, table 3 | ANOVA | NA | no | obs | obs | Mortality | juvenile | RAPD | Non-kin | yes | yes | NA | NA | 12 | 0.528 |
| 17 | Weyrauch, SL; Grubb, TC | 2006 | Effects of the interaction between genetic diversity and UV-B radiation on wood frog fitness | Conservation Biology | Rana sylvatica | fig 2b, table 3 | ANOVA | NA | no | obs | obs | Mortality | juvenile | RAPD | Non-kin | yes | yes | NA | NA | 12 | 0.593 |
| 18 | Page Jr, et al. | 1995 | Effects of worker genotypic diversity on honey bee colony development and behavior (Apis mellifera L.) | Behavioral Ecology and Sociobiology | Apis mellifera | table 1 | ANOVA | NA | yes | obs | exp | Parasitism | adult | relatedness | Kin | no | no | 10 | 34 | 44 | 0.116 |
| 18 | Page Jr, et al. | 1995 | Effects of worker genotypic diversity on honey bee colony development and behavior (Apis mellifera L.) | Behavioral Ecology and Sociobiology | Apis mellifera | table 1 | ANOVA | NA | yes | obs | exp | Mortality | adult | relatedness | Kin | no | no | 10 | 34 | 44 | -0.166 |
| 19 | Trouve, S; Degen, L; Renaud, F; et al. | 2003 | Evolutionary implications of a high selfing rate in the freshwater snail Lymnaea truncatula | Evolution | Lymnaea truncatula | result text | Pearson\_Correlation | NA | yes | obs | obs | Parasitism | adult | microsatellites | Kin | no | yes | NA | NA | 13 | 0.470 |
| 20 | Baer, B; Schmid-Hempel, P | 1999 | Experimental variation in Polyandrous affects parasite loads and fitness in a bumble-bee | Nature | Bombus terrestris | from fig 1a | Mann\_Whitney | NA | yes | obs | exp | Parasitism | adult | relatedness | Kin | no | no | 7 | 12 | 19 | 0.632 |
| 20 | Baer, B; Schmid-Hempel, P | 1999 | Experimental variation in Polyandrous affects parasite loads and fitness in a bumble-bee | Nature | Bombus terrestris | from fig 1a | Mann\_Whitney | NA | yes | obs | exp | Parasitism | adult | relatedness | Kin | no | no | 7 | 12 | 19 | 0.619 |
| 20 | Baer, B; Schmid-Hempel, P | 1999 | Experimental variation in Polyandrous affects parasite loads and fitness in a bumble-bee | Nature | Bombus terrestris | from fig 2 | Mann\_Whitney | NA | yes | obs | exp | Parasitism | adult | relatedness | Kin | no | no | 7 | 12 | 19 | 0.549 |
| 20 | Baer, B; Schmid-Hempel, P | 1999 | Experimental variation in Polyandrous affects parasite loads and fitness in a bumble-bee | Nature | Bombus terrestris | from fig 1b | Mann\_Whitney | NA | yes | obs | exp | Parasitism | adult | relatedness | Kin | no | no | 7 | 12 | 19 | 0.581 |
| 20 | Baer, B; Schmid-Hempel, P | 1999 | Experimental variation in Polyandrous affects parasite loads and fitness in a bumble-bee | Nature | Bombus terrestris | from fig 1b | Mann\_Whitney | NA | yes | obs | exp | Parasitism | adult | relatedness | Kin | no | no | 7 | 12 | 19 | 0.352 |
| 20 | Baer, B; Schmid-Hempel, P | 1999 | Experimental variation in Polyandrous affects parasite loads and fitness in a bumble-bee | Nature | Bombus terrestris | from fig 2 | Mann\_Whitney | NA | yes | obs | exp | Parasitism | adult | relatedness | Kin | no | no | 7 | 12 | 19 | 0.453 |
| 21 | Reber, A; Castella, G; Christe, P; Chapuisat, M | 2008 | Experimentally increased group diversity improves disease resistance in an ant species | Ecology Letters | Formica selysi | from fig 2 and text | Repeated measures ANOVA | NA | no | exp | exp | Mortality | adult | relatedness | Kin | yes | no | 8 | 32 | 40 | -0.062 |
| 21 | Reber, A; Castella, G; Christe, P; Chapuisat, M | 2008 | Experimentally increased group diversity improves disease resistance in an ant species | Ecology Letters | Formica selysi | from fig 2 and text | Repeated measures ANOVA | NA | yes | exp | exp | Mortality | adult | relatedness | Kin | yes | no | 8 | 32 | 49 | 0.154 |
| 21 | Reber, A; Castella, G; Christe, P; Chapuisat, M | 2008 | Experimentally increased group diversity improves disease resistance in an ant species | Ecology Letters | Formica selysi | from fig 1 and text | Repeated measures ANOVA | NA | no | exp | obs | Mortality | adult | microsatellites | Kin | yes | no | 28 | 27 | 55 | -0.127 |
| 21 | Reber, A; Castella, G; Christe, P; Chapuisat, M | 2008 | Experimentally increased group diversity improves disease resistance in an ant species | Ecology Letters | Formica selysi | from fig 1 and text | Repeated measures ANOVA | NA | yes | exp | obs | Mortality | adult | microsatellites | Kin | yes | no | 28 | 27 | 55 | -0.183 |
| 22 | Robinson, John D. Et al. | 2013 | Extinction hazards in experimental Daphnia magna populations: Effects of genotype diversity and environmental variation | Ecology and Evolution | Daphnia magna | table 2 A-1.2 | Coxs\_ProportionalHazard | NA | no | obs | exp | Mortality | adult | genotypes | Kin | yes | no | NA | NA | 80 | 0.053 |
| 22 | Robinson, John D. Et al. | 2013 | Extinction hazards in experimental Daphnia magna populations: Effects of genotype diversity and environmental variation | Ecology and Evolution | Daphnia magna | table 2 A-2 | Coxs\_ProportionalHazard | NA | no | obs | exp | Mortality | adult | genotypes | Kin | yes | no | NA | NA | 80 | 0.078 |
| 23 | Dobelmann, J; Loope, KJ; Wilson-Rankin, E; Quinn, O; Baty, JW; Gruber, MAM; Lester, PJ | 2017 | Fitness in invasive social wasps: the role of variation in viral load, immune response and paternity in predicting nest size and reproductive output | Oikos | Vespula vulgaris | table A2 = polyandry, fig 1b = viral load | Pearson\_Correlation | 2a | yes | obs | obs | Parasitism | adult | microsatellites | Kin | no | no | NA | NA | 6 | -0.198 |
| 24 | Tarpy, DR; vanEngelsdorp, D; Pettis, JS | 2013 | Genetic diversity affects colony survivorship in commercial honey bee colonies | Naturwissenschaften | Apis mellifera | table 1 | Linear\_Regression | NA | yes | obs | obs | Parasitism | adult | microsatellites | Kin | no | no | NA | NA | 78 | -0.032 |
| 24 | Tarpy, DR; vanEngelsdorp, D; Pettis, JS | 2013 | Genetic diversity affects colony survivorship in commercial honey bee colonies | Naturwissenschaften | Apis mellifera | table 1 | Linear\_Regression | NA | yes | obs | obs | Parasitism | adult | microsatellites | Kin | no | no | NA | NA | 78 | -0.130 |
| 24 | Tarpy, DR; vanEngelsdorp, D; Pettis, JS | 2013 | Genetic diversity affects colony survivorship in commercial honey bee colonies | Naturwissenschaften | Apis mellifera | table 1 | Linear\_Regression | NA | yes | obs | obs | Parasitism | adult | microsatellites | Kin | no | no | NA | NA | 78 | 0.063 |
| 24 | Tarpy, DR; vanEngelsdorp, D; Pettis, JS | 2013 | Genetic diversity affects colony survivorship in commercial honey bee colonies | Naturwissenschaften | Apis mellifera | table 1 | Linear\_Regression | NA | yes | obs | obs | Parasitism | adult | microsatellites | Kin | no | no | NA | NA | 78 | 0.110 |
| 24 | Tarpy, DR; vanEngelsdorp, D; Pettis, JS | 2013 | Genetic diversity affects colony survivorship in commercial honey bee colonies | Naturwissenschaften | Apis mellifera | text | Ttest | NA | yes | obs | obs | Mortality | adult | microsatellites | Kin | no | no | NA | NA | 78 | 0.111 |
| 24 | Tarpy, DR; vanEngelsdorp, D; Pettis, JS | 2013 | Genetic diversity affects colony survivorship in commercial honey bee colonies | Naturwissenschaften | Apis mellifera | table 1 | Linear\_Regression | NA | yes | obs | obs | Parasitism | adult | microsatellites | Kin | no | no | NA | NA | 78 | 0.126 |
| 24 | Tarpy, DR; vanEngelsdorp, D; Pettis, JS | 2013 | Genetic diversity affects colony survivorship in commercial honey bee colonies | Naturwissenschaften | Apis mellifera | table 1 | Linear\_Regression | NA | yes | obs | obs | Parasitism | adult | microsatellites | Kin | no | no | NA | NA | 78 | 0.077 |
| 25 | Hughes, WOH; Boomsma, JJ | 2004 | Genetic diversity and disease resistance in leaf-cutting ant societies | Evolution | Acromyrmex echinatior | text. Fig 4 | Repeated measures ANOVA | NA | yes | exp | exp | Mortality | adult | relatedness | Kin | yes | no | 80 | 80 | 160 | -0.064 |
| 25 | Hughes, WOH; Boomsma, JJ | 2004 | Genetic diversity and disease resistance in leaf-cutting ant societies | Evolution | Acromyrmex echinatior | text. Fig 4 | Repeated measures ANOVA | NA | no | exp | exp | Mortality | adult | relatedness | Kin | yes | no | 80 | 80 | 160 | -0.050 |
| 25 | Hughes, WOH; Boomsma, JJ | 2004 | Genetic diversity and disease resistance in leaf-cutting ant societies | Evolution | Acromyrmex echinatior | text. Fig 4 | Repeated measures ANOVA | NA | no | exp | exp | Mortality | adult | relatedness | Kin | yes | no | 80 | 80 | 160 | -0.038 |
| 25 | Hughes, WOH; Boomsma, JJ | 2004 | Genetic diversity and disease resistance in leaf-cutting ant societies | Evolution | Acromyrmex echinatior | text. Fig 4 | Repeated measures ANOVA | NA | yes | exp | exp | Mortality | adult | relatedness | Kin | yes | no | 80 | 80 | 160 | 0.247 |
| 26 | Field, SG; Lange, M; Schulenburg, H; Velavan, TP; Michiels, NK | 2007 | Genetic diversity and parasite defense in a fragmented urban metapopulation of earthworms | Animal Conservation | Lumbricus terrestris | from fig 5 | Pearson\_Correlation | NA | yes | obs | obs | Parasitism | adult | microsatellites | Kin | no | yes | NA | NA | 14 | 0.150 |
| 27 | Hughes, AR; Stachowicz, JJ | 2004 | Genetic diversity enhances the resistance of a seagrass ecosystem to disturbance | Proceedings of the National Academy of Sciences | Zostera marina | from fig 1b, table 1 | Linear\_Regression | NA | yes | obs | exp | Mortality | adult | microsatellites | Kin | no | no | NA | NA | 24 | 0.800 |
| 28 | Aguirre, JD; Marshall, DJ | 2012 | Genetic diversity increases population productivity in a sessile marine invertebrate | Ecology | Bugula neritina | fig 1A + text exp 1, 6 weeks | ANOVA | NA | yes | obs | exp | Mortality | adult | relatedness | Kin | no | no | 9 | 9 | 18 | 0.708 |
| 29 | Altermatt, F; Ebert, D | 2008 | Genetic diversity of Daphnia magna populations enhances resistance to parasites | Ecology Letters | Daphnia magna | from fig 2a summer 2005 | Means and SD | NA | yes | exp | exp | Parasitism | adult | genotypes | Kin | yes | no | 15 | 15 | 30 | 0.134 |
| 29 | Altermatt, F; Ebert, D | 2008 | Genetic diversity of Daphnia magna populations enhances resistance to parasites | Ecology Letters | Daphnia magna | from fig 2b autumn 2007 | Means and SD | NA | yes | exp | exp | Parasitism | adult | genotypes | Kin | yes | no | 15 | 15 | 30 | 0.225 |
| 29 | Altermatt, F; Ebert, D | 2008 | Genetic diversity of Daphnia magna populations enhances resistance to parasites | Ecology Letters | Daphnia magna | from fig 2a summer 2006 | Means and SD | NA | yes | exp | exp | Parasitism | adult | genotypes | Kin | yes | no | 15 | 15 | 30 | 0.550 |
| 29 | Altermatt, F; Ebert, D | 2008 | Genetic diversity of Daphnia magna populations enhances resistance to parasites | Ecology Letters | Daphnia magna | from fig 2b autumn 2005 | Means and SD | NA | yes | exp | exp | Parasitism | adult | genotypes | Kin | yes | no | 15 | 15 | 30 | 0.402 |
| 29 | Altermatt, F; Ebert, D | 2008 | Genetic diversity of Daphnia magna populations enhances resistance to parasites | Ecology Letters | Daphnia magna | from fig 2a spring 2006 | Means and SD | NA | yes | exp | exp | Parasitism | adult | genotypes | Kin | yes | no | 15 | 15 | 30 | 0.420 |
| 29 | Altermatt, F; Ebert, D | 2008 | Genetic diversity of Daphnia magna populations enhances resistance to parasites | Ecology Letters | Daphnia magna | from fig 2b spring 2006 | Means and SD | NA | yes | exp | exp | Parasitism | adult | genotypes | Kin | yes | no | 15 | 15 | 30 | 0.153 |
| 29 | Altermatt, F; Ebert, D | 2008 | Genetic diversity of Daphnia magna populations enhances resistance to parasites | Ecology Letters | Daphnia magna | fig3 high parasite diversity | Means and SD | NA | yes | exp | exp | Mortality | adult | genotypes | Kin | yes | no | 15 | 15 | 30 | 0.504 |
| 29 | Altermatt, F; Ebert, D | 2008 | Genetic diversity of Daphnia magna populations enhances resistance to parasites | Ecology Letters | Daphnia magna | from fig 2a spring 2007 | Means and SD | NA | yes | exp | exp | Parasitism | adult | genotypes | Kin | yes | no | 15 | 15 | 30 | -0.222 |
| 29 | Altermatt, F; Ebert, D | 2008 | Genetic diversity of Daphnia magna populations enhances resistance to parasites | Ecology Letters | Daphnia magna | from fig 2b autumn 2006 | Means and SD | NA | yes | exp | exp | Parasitism | adult | genotypes | Kin | yes | no | 15 | 15 | 30 | 0.329 |
| 29 | Altermatt, F; Ebert, D | 2008 | Genetic diversity of Daphnia magna populations enhances resistance to parasites | Ecology Letters | Daphnia magna | from fig 2b summer 2006 | Means and SD | NA | yes | exp | exp | Parasitism | adult | genotypes | Kin | yes | no | 15 | 15 | 30 | 0.581 |
| 29 | Altermatt, F; Ebert, D | 2008 | Genetic diversity of Daphnia magna populations enhances resistance to parasites | Ecology Letters | Daphnia magna | from fig 2a autumn 2007 | Means and SD | NA | yes | exp | exp | Parasitism | adult | genotypes | Kin | yes | no | 15 | 15 | 30 | 0.459 |
| 29 | Altermatt, F; Ebert, D | 2008 | Genetic diversity of Daphnia magna populations enhances resistance to parasites | Ecology Letters | Daphnia magna | from fig 2b summer 2005 | Means and SD | NA | yes | exp | exp | Parasitism | adult | genotypes | Kin | yes | no | 15 | 15 | 30 | 0.109 |
| 29 | Altermatt, F; Ebert, D | 2008 | Genetic diversity of Daphnia magna populations enhances resistance to parasites | Ecology Letters | Daphnia magna | from fig 2a autumn 2005 | Means and SD | NA | yes | exp | exp | Parasitism | adult | genotypes | Kin | yes | no | 15 | 15 | 30 | 0.064 |
| 29 | Altermatt, F; Ebert, D | 2008 | Genetic diversity of Daphnia magna populations enhances resistance to parasites | Ecology Letters | Daphnia magna | fig3 low parasite diversity | Means and SD | NA | yes | exp | exp | Mortality | adult | genotypes | Kin | yes | no | 15 | 15 | 30 | 0.174 |
| 29 | Altermatt, F; Ebert, D | 2008 | Genetic diversity of Daphnia magna populations enhances resistance to parasites | Ecology Letters | Daphnia magna | from fig 2b spring 2007 | Means and SD | NA | yes | exp | exp | Parasitism | adult | genotypes | Kin | yes | no | 15 | 15 | 30 | 0.098 |
| 29 | Altermatt, F; Ebert, D | 2008 | Genetic diversity of Daphnia magna populations enhances resistance to parasites | Ecology Letters | Daphnia magna | fig3 control | Means and SD | NA | no | exp | exp | Mortality | adult | genotypes | Kin | yes | no | 15 | 15 | 30 | -0.178 |
| 29 | Altermatt, F; Ebert, D | 2008 | Genetic diversity of Daphnia magna populations enhances resistance to parasites | Ecology Letters | Daphnia magna | from fig 2a autumn 2006 | Means and SD | NA | yes | exp | exp | Parasitism | adult | genotypes | Kin | yes | no | 15 | 15 | 30 | 0.263 |
| 30 | Desai, SD; Currie, RW | 2015 | Genetic diversity within honey bee colonies affects pathogen load and relative virus levels in honey bees, Apis mellifera L | Behav Ecol and Sociobiol | Apis mellifera | fig 5b | ANOVA | NA | yes | obs | exp | Mortality | adult | relatedness | Kin | no | no | 24 | 12 | 36 | -0.189 |
| 30 | Desai, SD; Currie, RW | 2015 | Genetic diversity within honey bee colonies affects pathogen load and relative virus levels in honey bees, Apis mellifera L | Behav Ecol and Sociobiol | Apis mellifera | fig 7a fall | Means and SD | NA | yes | obs | exp | Parasitism | adult | relatedness | Kin | no | no | 24 | 11 | 35 | 0.231 |
| 30 | Desai, SD; Currie, RW | 2015 | Genetic diversity within honey bee colonies affects pathogen load and relative virus levels in honey bees, Apis mellifera L | Behav Ecol and Sociobiol | Apis mellifera | fig 7a fall | Means and SD | NA | yes | obs | exp | Parasitism | adult | relatedness | Kin | no | no | 24 | 11 | 35 | 0.162 |
| 30 | Desai, SD; Currie, RW | 2015 | Genetic diversity within honey bee colonies affects pathogen load and relative virus levels in honey bees, Apis mellifera L | Behav Ecol and Sociobiol | Apis mellifera | fig 7b spring | Means and SD | NA | yes | obs | exp | Parasitism | adult | relatedness | Kin | no | no | 24 | 11 | 35 | 0.009 |
| 30 | Desai, SD; Currie, RW | 2015 | Genetic diversity within honey bee colonies affects pathogen load and relative virus levels in honey bees, Apis mellifera L | Behav Ecol and Sociobiol | Apis mellifera | fig 7b spring | Means and SD | NA | yes | obs | exp | Parasitism | adult | relatedness | Kin | no | no | 24 | 11 | 35 | 0.076 |
| 30 | Desai, SD; Currie, RW | 2015 | Genetic diversity within honey bee colonies affects pathogen load and relative virus levels in honey bees, Apis mellifera L | Behav Ecol and Sociobiol | Apis mellifera | fig 7b spring | Means and SD | NA | yes | obs | exp | Parasitism | adult | relatedness | Kin | no | no | 24 | 11 | 35 | -0.346 |
| 30 | Desai, SD; Currie, RW | 2015 | Genetic diversity within honey bee colonies affects pathogen load and relative virus levels in honey bees, Apis mellifera L | Behav Ecol and Sociobiol | Apis mellifera | fig 8a N. Apis | Fishers\_Exact | NA | yes | obs | exp | Parasitism | adult | relatedness | Kin | no | no | 15 | 8 | 23 | 0.159 |
| 30 | Desai, SD; Currie, RW | 2015 | Genetic diversity within honey bee colonies affects pathogen load and relative virus levels in honey bees, Apis mellifera L | Behav Ecol and Sociobiol | Apis mellifera | fig 4 | ANOVA | NA | yes | obs | exp | Mortality | adult | relatedness | Kin | no | no | 24 | 11 | 35 | -0.241 |
| 30 | Desai, SD; Currie, RW | 2015 | Genetic diversity within honey bee colonies affects pathogen load and relative virus levels in honey bees, Apis mellifera L | Behav Ecol and Sociobiol | Apis mellifera | fig 1b fall | Means and SD | NA | yes | obs | exp | Parasitism | adult | relatedness | Kin | no | no | 24 | 11 | 35 | -0.555 |
| 30 | Desai, SD; Currie, RW | 2015 | Genetic diversity within honey bee colonies affects pathogen load and relative virus levels in honey bees, Apis mellifera L | Behav Ecol and Sociobiol | Apis mellifera | fig 7a fall | Means and SD | NA | yes | obs | exp | Parasitism | adult | relatedness | Kin | no | no | 24 | 11 | 35 | 0.330 |
| 30 | Desai, SD; Currie, RW | 2015 | Genetic diversity within honey bee colonies affects pathogen load and relative virus levels in honey bees, Apis mellifera L | Behav Ecol and Sociobiol | Apis mellifera | fig 1b spring | Means and SD | NA | yes | obs | exp | Parasitism | adult | relatedness | Kin | no | no | 24 | 11 | 35 | -0.106 |
| 30 | Desai, SD; Currie, RW | 2015 | Genetic diversity within honey bee colonies affects pathogen load and relative virus levels in honey bees, Apis mellifera L | Behav Ecol and Sociobiol | Apis mellifera | fig 8b N. Ceranae | Fishers\_Exact | NA | yes | obs | exp | Parasitism | adult | relatedness | Kin | no | no | 15 | 8 | 23 | 0.452 |
| 31 | Tarpy, DR | 2003 | Genetic diversity within honeybee colonies prevents severe infections and promotes colony growth | Proc. R. Soc. Lond. B. | Apis mellifera | from fig 2a | Means and SD | NA | yes | obs | exp | Parasitism | adult | relatedness | Kin | no | no | 24 | 24 | 48 | -0.272 |
| 32 | SHYKOFF, JA; SCHMIDHEMPEL, P | 1991 | Genetic relatedness and eusociality parasite mediated selection on the genetic composition of groups | Behavioral Ecology and Sociobiology | Bombus terrestris | table 3 | Ttest | NA | yes | exp | exp | Parasitism | adult | relatedness | Kin | yes | no | 15 | 15 | 30 | 0.361 |
| 33 | Bichet, C; Moodley, Y; Penn, DJ; Sorci, G; Garnier, S | 2015 | Genetic structure in insular and mainland populations of house sparrows (Passer domesticus) and their hemosporidian parasites | Ecology and Evolution | Passer domesticus | table 1 | Pearson\_Correlation | 6a | yes | obs | obs | Parasitism | adult | microsatellites | Non-kin | no | yes | NA | NA | 12 | -0.114 |
| 33 | Bichet, C; Moodley, Y; Penn, DJ; Sorci, G; Garnier, S | 2015 | Genetic structure in insular and mainland populations of house sparrows (Passer domesticus) and their hemosporidian parasites | Ecology and Evolution | Passer domesticus | table 1 | Pearson\_Correlation | 6d | yes | obs | obs | Parasitism | adult | microsatellites | Non-kin | no | yes | NA | NA | 12 | -0.097 |
| 34 | Liersch, and Schmid-Hempel | 1998 | Genetic variation within social insect colonies reduces parasite load | Proc. R. Soc. Lond. B. | Bombus terrestris | from fig 1 | Ttest | NA | yes | obs | exp | Parasitism | adult | relatedness | Kin | no | no | 10 | 7 | 17 | 0.528 |
| 34 | Liersch, and Schmid-Hempel | 1998 | Genetic variation within social insect colonies reduces parasite load | Proc. R. Soc. Lond. B. | Bombus terrestris | from fig 1 | Ttest | NA | yes | obs | exp | Parasitism | adult | relatedness | Kin | no | no | 10 | 7 | 17 | 0.507 |
| 34 | Liersch, and Schmid-Hempel | 1998 | Genetic variation within social insect colonies reduces parasite load | Proc. R. Soc. Lond. B. | Bombus terrestris | from fig 1 | Ttest | NA | yes | obs | exp | Parasitism | adult | relatedness | Kin | no | no | 10 | 7 | 17 | 0.433 |
| 35 | Cook-Patton, SC; Hastings, AP; Agrawal, AA | 2017 | Genotypic diversity mitigates negative effects of density on plant performance: a field experiment and life cycle analysis of common evening primrose Oenothera biennis | Journal of Ecology | Oenothera biennis | fig 2a + text | Logistic\_Regression | NA | yes | obs | exp | Mortality | adult | microsatellites | Kin | no | no | 100 | 120 | 220 | 0.000 |
| 36 | Parker, John D.; Salminen, Juha-Pekka; Agrawal, Anurag A. | 2010 | Herbivory enhances positive effects of plant genotypic diversity | Ecology Letters | Oenothera biennis | figure 1a | ANOVA | NA | yes | obs | exp | Mortality | adult | genotypes | Kin | no | no | NA | NA | 476 | 0.094 |
| 37 | He, TH; Lamont, BB | 2010 | High microsatellite genetic diversity fails to predict greater population resistance to extreme drought | Conservation Genetics | Banksia hookeriana | fig 2a | Pearson\_Correlation | NA | yes | obs | obs | Mortality | adult | microsatellites | Kin | no | yes | NA | NA | 15 | -0.383 |
| 38 | Kapranas, A; Maher, AMD; Griffin, CT | 2016 | Higher relatedness mitigates mortality in a nematode with lethal male fighting | JOURNAL OF EVOLUTIONARY BIOLOGY | Steinernema longicaudum | exp 3 text | GLM | NA | no | obs | exp | Mortality | adult | relatedness | Kin | yes | no | 27 | 27 | 54 | -0.279 |
| 38 | Kapranas, A; Maher, AMD; Griffin, CT | 2016 | Higher relatedness mitigates mortality in a nematode with lethal male fighting | JOURNAL OF EVOLUTIONARY BIOLOGY | Steinernema longicaudum | exp 1, fig 1, 48 h | GLM | NA | no | obs | exp | Mortality | adult | relatedness | Kin | yes | no | NA | NA | 144 | -0.337 |
| 38 | Kapranas, A; Maher, AMD; Griffin, CT | 2016 | Higher relatedness mitigates mortality in a nematode with lethal male fighting | JOURNAL OF EVOLUTIONARY BIOLOGY | Steinernema longicaudum | exp 2, fig 2, 48 h | Means and SD | NA | no | obs | exp | Mortality | adult | relatedness | Kin | yes | no | 18 | 18 | 36 | -0.175 |
| 38 | Kapranas, A; Maher, AMD; Griffin, CT | 2016 | Higher relatedness mitigates mortality in a nematode with lethal male fighting | JOURNAL OF EVOLUTIONARY BIOLOGY | Steinernema longicaudum | exp 2, fig 2, 48 h | Means and SD | NA | no | obs | exp | Mortality | adult | relatedness | Kin | yes | no | 18 | 18 | 36 | -0.187 |
| 39 | Gardner, MG; Schonrogge, K; Elmes, GW; Thomas, JA | 2007 | Increased genetic diversity as a defence against parasites is undermined by social parasites: Microdon mutabilis hoverflies infesting Formica lemani ant colonies | Proc. R. Soc. Lond. B. | Formica lemani | text section d Burren colonies | Spearman\_Correlation | NA | yes | obs | obs | Parasitism | adult | microsatellites | Kin | no | no | NA | NA | 30 | 0.071 |
| 39 | Gardner, MG; Schonrogge, K; Elmes, GW; Thomas, JA | 2007 | Increased genetic diversity as a defence against parasites is undermined by social parasites: Microdon mutabilis hoverflies infesting Formica lemani ant colonies | Proc. R. Soc. Lond. B. | Formica lemani | fig 1 and text, Mull pop | Means and SD | NA | yes | obs | obs | Parasitism | adult | microsatellites | Kin | no | no | 22 | 19 | 41 | -0.714 |
| 39 | Gardner, MG; Schonrogge, K; Elmes, GW; Thomas, JA | 2007 | Increased genetic diversity as a defence against parasites is undermined by social parasites: Microdon mutabilis hoverflies infesting Formica lemani ant colonies | Proc. R. Soc. Lond. B. | Formica lemani | text (b) Burren | Means and SD | NA | yes | obs | obs | Parasitism | adult | microsatellites | Kin | no | no | 20 | 10 | 30 | -0.546 |
| 39 | Gardner, MG; Schonrogge, K; Elmes, GW; Thomas, JA | 2007 | Increased genetic diversity as a defence against parasites is undermined by social parasites: Microdon mutabilis hoverflies infesting Formica lemani ant colonies | Proc. R. Soc. Lond. B. | Formica lemani | text section d mull colonies | Spearman\_Correlation | NA | yes | obs | obs | Parasitism | adult | microsatellites | Kin | no | no | NA | NA | 41 | -0.060 |
| 40 | Gamfeldt, L; Kallstrom, B | 2007 | Increasing intraspecific diversity increases predictability in population survival in the face of perturbations | Oikos | Gammarus duebeni | fig 1 + text | Linear\_Regression | NA | no | obs | exp | Mortality | adult | relatedness | Non-kin | yes | no | NA | NA | 4 | -0.013 |
| 40 | Gamfeldt, L; Kallstrom, B | 2007 | Increasing intraspecific diversity increases predictability in population survival in the face of perturbations | Oikos | Gammarus duebeni | fig 1 + text | Linear\_Regression | NA | no | obs | exp | Mortality | adult | relatedness | Non-kin | yes | no | NA | NA | 4 | -0.100 |
| 41 | LAMBIN, X; KREBS, CJ | 1993 | Influence of Female Relatedness on the Demography of Townsend’s Vole Populations in Spring | Brittish Ecological Society | Microtus townsendii | fig3female pooled | Means and SD | NA | yes | obs | exp | Mortality | adult\_juvenile | relatedness | Kin | no | no | 11 | 51 | 62 | -0.112 |
| 43 | Tarpy, DR; Seeley, TD | 2006 | Lower disease infections in honeybee (Apis mellifera) colonies headed by polyandrous vs monandrous queens | Naturwissenschaften | Apis mellifera | fig 1 | Ttest | NA | yes | obs | exp | Parasitism | adult | relatedness | Kin | no | no | 10 | 10 | 20 | 0.520 |
| 44 | Franklin, MT; Ritland, CE; Myers, JH; Cory, JS | 2012 | Multiple Mating and Family Structure of the Western Tent Caterpillar, Malacosoma californicum pluviale: Impact on Disease Resistance | Plos One | Malacosoma californicum pluviale | from text | Pearson\_Correlation | NA | yes | obs | obs | Parasitism | adult\_juvenile | microsatellites | Kin | no | yes | NA | NA | 12 | 0.295 |
| 45 | Vanpe, C; Kjellander, P; Gaillard, JM; Cosson, JF; Galan, M; Hewison, AJM | 2009 | Multiple paternity occurs with low frequency in the territorial roe deer, Capreolus capreolus | Biological Journal of the Linnean Society | Capreolus capreolus | text, survival first year | ChiSq | NA | yes | obs | obs | Mortality | juvenile | microsatellites | Kin | no | yes | 5 | 28 | 33 | 0.400 |
| 46 | Schmidt, AM; Linksvayer, TA; Boomsma, JJ; Pedersen, JS | 2011 | No benefit in diversity? The effect of genetic variation on survival and disease resistance in a polygynous social insect | Ecological Entomology | Monomorium pharaonis | fig3b mixed control vs U3 | Means and SD | NA | no | exp | exp | Mortality | juvenile | relatedness | Kin | yes | no | 6 | 18 | 35 | -0.634 |
| 46 | Schmidt, AM; Linksvayer, TA; Boomsma, JJ; Pedersen, JS | 2011 | No benefit in diversity? The effect of genetic variation on survival and disease resistance in a polygynous social insect | Ecological Entomology | Monomorium pharaonis | fig3b mixed control vs U1 | Means and SD | NA | yes | exp | exp | Mortality | juvenile | relatedness | Kin | yes | no | 6 | 18 | 35 | -0.916 |
| 46 | Schmidt, AM; Linksvayer, TA; Boomsma, JJ; Pedersen, JS | 2011 | No benefit in diversity? The effect of genetic variation on survival and disease resistance in a polygynous social insect | Ecological Entomology | Monomorium pharaonis | fig3a mixed infected vs U3 | Means and SD | NA | yes | exp | exp | Mortality | adult | relatedness | Kin | yes | no | 4 | 16 | 28 | -0.341 |
| 46 | Schmidt, AM; Linksvayer, TA; Boomsma, JJ; Pedersen, JS | 2011 | No benefit in diversity? The effect of genetic variation on survival and disease resistance in a polygynous social insect | Ecological Entomology | Monomorium pharaonis | fig3a mixed infected vs U5 | Means and SD | NA | yes | exp | exp | Mortality | adult | relatedness | Kin | yes | no | 4 | 12 | 24 | -0.041 |
| 46 | Schmidt, AM; Linksvayer, TA; Boomsma, JJ; Pedersen, JS | 2011 | No benefit in diversity? The effect of genetic variation on survival and disease resistance in a polygynous social insect | Ecological Entomology | Monomorium pharaonis | fig3b mixed infected vs U3 | Means and SD | NA | no | exp | exp | Mortality | juvenile | relatedness | Kin | yes | no | 4 | 16 | 28 | -0.816 |
| 46 | Schmidt, AM; Linksvayer, TA; Boomsma, JJ; Pedersen, JS | 2011 | No benefit in diversity? The effect of genetic variation on survival and disease resistance in a polygynous social insect | Ecological Entomology | Monomorium pharaonis | fig3a mixed control vs U1 | Means and SD | NA | yes | exp | exp | Mortality | adult | relatedness | Kin | yes | no | 6 | 18 | 35 | 0.441 |
| 46 | Schmidt, AM; Linksvayer, TA; Boomsma, JJ; Pedersen, JS | 2011 | No benefit in diversity? The effect of genetic variation on survival and disease resistance in a polygynous social insect | Ecological Entomology | Monomorium pharaonis | fig3b mixed infected vs U5 | Means and SD | NA | yes | exp | exp | Mortality | juvenile | relatedness | Kin | yes | no | 4 | 12 | 24 | -0.742 |
| 46 | Schmidt, AM; Linksvayer, TA; Boomsma, JJ; Pedersen, JS | 2011 | No benefit in diversity? The effect of genetic variation on survival and disease resistance in a polygynous social insect | Ecological Entomology | Monomorium pharaonis | fig3b mixed control vs U5 | Means and SD | NA | no | exp | exp | Mortality | juvenile | relatedness | Kin | yes | no | 6 | 9 | 26 | -0.824 |
| 46 | Schmidt, AM; Linksvayer, TA; Boomsma, JJ; Pedersen, JS | 2011 | No benefit in diversity? The effect of genetic variation on survival and disease resistance in a polygynous social insect | Ecological Entomology | Monomorium pharaonis | fig3a mixed infected vs U1 | Means and SD | NA | no | exp | exp | Mortality | adult | relatedness | Kin | yes | no | 4 | 11 | 23 | 0.123 |
| 46 | Schmidt, AM; Linksvayer, TA; Boomsma, JJ; Pedersen, JS | 2011 | No benefit in diversity? The effect of genetic variation on survival and disease resistance in a polygynous social insect | Ecological Entomology | Monomorium pharaonis | fig3b mixed infected vs U1 | Means and SD | NA | yes | exp | exp | Mortality | juvenile | relatedness | Kin | yes | no | 4 | 11 | 23 | -0.706 |
| 46 | Schmidt, AM; Linksvayer, TA; Boomsma, JJ; Pedersen, JS | 2011 | No benefit in diversity? The effect of genetic variation on survival and disease resistance in a polygynous social insect | Ecological Entomology | Monomorium pharaonis | fig3a mixed control vs U5 | Means and SD | NA | no | exp | exp | Mortality | adult | relatedness | Kin | yes | no | 6 | 9 | 26 | -0.592 |
| 46 | Schmidt, AM; Linksvayer, TA; Boomsma, JJ; Pedersen, JS | 2011 | No benefit in diversity? The effect of genetic variation on survival and disease resistance in a polygynous social insect | Ecological Entomology | Monomorium pharaonis | fig3a mixed control vs U3 | Means and SD | NA | no | exp | exp | Mortality | adult | relatedness | Kin | yes | no | 6 | 18 | 35 | -0.062 |
| 49 | King, K.C., Jokela, J. & Lively, C.M. | 2011 | Parasites, sex, and clonal diversity in natural snail populations | Evolution | Potamopyrgus antipodarum | table 4, fig 1c | Linear\_Regression | NA | yes | obs | obs | Parasitism | adult | microsatellites | Kin | no | yes | NA | NA | 17 | -0.621 |
| 50 | Kotowska, AM; Cahill, JF; Keddie, BA | 2010 | Plant genetic diversity yields increased plant productivity and herbivore performance | Journal of Ecology | Arabidopsis thaliana | table 1 | GLM | NA | yes | obs | exp | Mortality | adult | genotypes | Kin | no | no | 160 | 360 | 520 | 0.118 |
| 51 | Crutsinger, Gregory M.; Collins, Michael D.; Fordyce, James A.; et al. | 2006 | Plant genotypic diversity predicts community structure and governs an ecosystem process | Science | Solidago altissima | fig 1b | Pearson\_Correlation | NA | yes | obs | exp | Parasitism | adult | genotypes | Kin | no | no | NA | NA | 63 | -0.539 |
| 52 | Teacher, AGF; Garner, TWJ; Nichols, RA | 2009 | Population genetic patterns suggest a behavioural change in wild common frogs (Rana temporaria) following disease outbreaks (Ranavirus) | Molecular Ecology | Rana temporaria | from table 2 | Permutation\_test | NA | yes | obs | obs | Parasitism | juvenile | microsatellites | Non-kin | no | yes | NA | NA | 34 | -0.082 |
| 53 | de Vere, N; Jongejans, E; Plowman, A; Williams, E | 2009 | Population size and habitat quality affect genetic diversity and fitness in the clonal herb Cirsium dissectum | OECOLOGIA | Cirsium dissectum | table 2 | Linear\_Regression | NA | yes | obs | obs | Mortality | adult | microsatellites | Kin | no | yes | NA | NA | 14 | 0.520 |
| 54 | Anton, Christian; Zeisset, Inga; Musche, Martin; et al. | 2007 | Population structure of a large blue butterfly and its specialist parasitoid in a fragmented landscape | Molecular Ecology | Maculinea nausithous | table 1 | Pearson\_Correlation | 9a | yes | obs | obs | Parasitism | juvenile | microsatellites | Kin | no | yes | NA | NA | 11 | -0.258 |
| 55 | Seeley, TD; Tarpy, DR | 2007 | Queen promiscuity lowers disease within honeybee colonies | Proc. R. Soc. Lond. B. | Apis mellifera | fig 1 meas 2 | Wilcoxon\_signed\_rank | NA | yes | exp | exp | Parasitism | adult | relatedness | Kin | no | no | 14 | 14 | 28 | 0.439 |
| 56 | Ugelvig, Line V.; Kronauer, Daniel J. C.; Schrempf, Alexandra; et al. | 2010 | Rapid anti-pathogen response in ant societies relies on high genetic diversity | PROCEEDINGS OF THE ROYAL SOCIETY B-BIOLOGICAL SCIENCES | Cardiocondyla obscurior | result text | Coxs\_ProportionalHazard | NA | yes | obs | exp | Mortality | adult\_juvenile | relatedness | Kin | yes | yes | 8 | 8 | 16 | 0.126 |
| 57 | Ben-Ami, F, Heller, J | 2005 | Spatial and temporal patterns of parthenogenesis and parasitism in the freshwater snail Melanoides tuberculata | Journal of Ecvolutionary Biology | Melanoides tuberculata | table 1 Heller & Farstey, 1990 | Pearson\_Correlation | NA | yes | obs | obs | Parasitism | adult | male frequency | Kin | no | no | NA | NA | 34 | 0.022 |
| 57 | Ben-Ami, F, Heller, J | 2005 | Spatial and temporal patterns of parthenogenesis and parasitism in the freshwater snail Melanoides tuberculata | Journal of Ecvolutionary Biology | Melanoides tuberculata | table 1 Second Survey 2001 | Pearson\_Correlation | NA | yes | obs | obs | Parasitism | adult | male frequency | Kin | no | no | NA | NA | 7 | 0.270 |
| 57 | Ben-Ami, F, Heller, J | 2005 | Spatial and temporal patterns of parthenogenesis and parasitism in the freshwater snail Melanoides tuberculata | Journal of Ecvolutionary Biology | Melanoides tuberculata | table 1 First Survey 1999 | Pearson\_Correlation | NA | yes | obs | obs | Parasitism | adult | male frequency | Kin | no | no | NA | NA | 23 | 0.353 |
| 58 | Pearman, PB; Garner, TWJ | 2005 | Susceptibility of Italian agile frog populations to an emerging strain of Ranavirus parallels population genetic diversity | Ecology Letters | Rana latastei | fig 3 table 1 low | GEE | NA | yes | exp | obs | Mortality | juvenile | microsatellites | Non-kin | yes | yes | 30 | 30 | 60 | 0.539 |
| 58 | Pearman, PB; Garner, TWJ | 2005 | Susceptibility of Italian agile frog populations to an emerging strain of Ranavirus parallels population genetic diversity | Ecology Letters | Rana latastei | fig 3 table 1 high | GEE | NA | yes | exp | obs | Mortality | juvenile | microsatellites | Non-kin | yes | yes | 30 | 30 | 60 | 0.469 |
| 58 | Pearman, PB; Garner, TWJ | 2005 | Susceptibility of Italian agile frog populations to an emerging strain of Ranavirus parallels population genetic diversity | Ecology Letters | Rana latastei | fig 3 table 1 low | GEE | NA | no | exp | obs | Mortality | juvenile | microsatellites | Non-kin | yes | yes | 30 | 30 | 60 | -0.048 |
| 59 | Crutsinger, Gregory M.; Reynolds, W. Nicholas; Classen, Aimee T.; et al. | 2008 | Temporal dynamics in non-additive responses of arthropods to host-plant genotypic diversity | Oikos | Solidago altissima | appendix 1 table 4, Sept | ANOVA | NA | yes | obs | exp | Parasitism | adult | relatedness | Kin | no | no | NA | NA | 63 | -0.412 |
| 59 | Crutsinger, Gregory M.; Reynolds, W. Nicholas; Classen, Aimee T.; et al. | 2008 | Temporal dynamics in non-additive responses of arthropods to host-plant genotypic diversity | Oikos | Solidago altissima | appendix 1 table 4, May | ANOVA | NA | yes | obs | exp | Parasitism | adult | relatedness | Kin | no | no | NA | NA | 63 | -0.208 |
| 59 | Crutsinger, Gregory M.; Reynolds, W. Nicholas; Classen, Aimee T.; et al. | 2008 | Temporal dynamics in non-additive responses of arthropods to host-plant genotypic diversity | Oikos | Solidago altissima | appendix 1 table 4, June | ANOVA | NA | yes | obs | exp | Parasitism | adult | relatedness | Kin | no | no | NA | NA | 63 | -0.607 |
| 59 | Crutsinger, Gregory M.; Reynolds, W. Nicholas; Classen, Aimee T.; et al. | 2008 | Temporal dynamics in non-additive responses of arthropods to host-plant genotypic diversity | Oikos | Solidago altissima | appendix 1 table 4, July | ANOVA | NA | yes | obs | exp | Parasitism | adult | relatedness | Kin | no | no | NA | NA | 63 | -0.542 |
| 59 | Crutsinger, Gregory M.; Reynolds, W. Nicholas; Classen, Aimee T.; et al. | 2008 | Temporal dynamics in non-additive responses of arthropods to host-plant genotypic diversity | Oikos | Solidago altissima | appendix 1 table 2 July | ANOVA | NA | yes | obs | exp | Parasitism | adult | relatedness | Kin | no | no | NA | NA | 63 | -0.611 |
| 59 | Crutsinger, Gregory M.; Reynolds, W. Nicholas; Classen, Aimee T.; et al. | 2008 | Temporal dynamics in non-additive responses of arthropods to host-plant genotypic diversity | Oikos | Solidago altissima | appendix 1 table 4, Oct | ANOVA | NA | yes | obs | exp | Parasitism | adult | relatedness | Kin | no | no | NA | NA | 63 | -0.319 |
| 59 | Crutsinger, Gregory M.; Reynolds, W. Nicholas; Classen, Aimee T.; et al. | 2008 | Temporal dynamics in non-additive responses of arthropods to host-plant genotypic diversity | Oikos | Solidago altissima | appendix 1 table 2 May | ANOVA | NA | yes | obs | exp | Parasitism | adult | relatedness | Kin | no | no | NA | NA | 63 | -0.351 |
| 59 | Crutsinger, Gregory M.; Reynolds, W. Nicholas; Classen, Aimee T.; et al. | 2008 | Temporal dynamics in non-additive responses of arthropods to host-plant genotypic diversity | Oikos | Solidago altissima | appendix 1 table 2 June | ANOVA | NA | yes | obs | exp | Parasitism | adult | relatedness | Kin | no | no | NA | NA | 63 | -0.622 |
| 59 | Crutsinger, Gregory M.; Reynolds, W. Nicholas; Classen, Aimee T.; et al. | 2008 | Temporal dynamics in non-additive responses of arthropods to host-plant genotypic diversity | Oikos | Solidago altissima | appendix 1 table 2 Sept | ANOVA | NA | yes | obs | exp | Parasitism | adult | relatedness | Kin | no | no | NA | NA | 63 | -0.500 |
| 59 | Crutsinger, Gregory M.; Reynolds, W. Nicholas; Classen, Aimee T.; et al. | 2008 | Temporal dynamics in non-additive responses of arthropods to host-plant genotypic diversity | Oikos | Solidago altissima | appendix 1 table 2 October | ANOVA | NA | yes | obs | exp | Parasitism | adult | relatedness | Kin | no | no | NA | NA | 63 | -0.272 |
| 60 | Fraser, BA; Ramnarine, IW; Neff, BD | 2010 | TEMPORAL VARIATION AT THE MHC CLASS IIB IN WILD POPULATIONS OF THE GUPPY (POECILIA RETICULATA) | Evolution | Poecilia reticulata | table 1(fit) and table 2 (gd) 2007 | Spearman\_Correlation | 3b | yes | obs | obs | Parasitism | adult | microsatellites | Non-kin | no | yes | NA | NA | 9 | -0.062 |
| 60 | Fraser, BA; Ramnarine, IW; Neff, BD | 2010 | TEMPORAL VARIATION AT THE MHC CLASS IIB IN WILD POPULATIONS OF THE GUPPY (POECILIA RETICULATA) | Evolution | Poecilia reticulata | table 1(fit) and table 2 (gd) 2006 | Spearman\_Correlation | 3l | yes | obs | obs | Parasitism | adult | microsatellites | Non-kin | no | yes | NA | NA | 9 | 0.165 |
| 60 | Fraser, BA; Ramnarine, IW; Neff, BD | 2010 | TEMPORAL VARIATION AT THE MHC CLASS IIB IN WILD POPULATIONS OF THE GUPPY (POECILIA RETICULATA) | Evolution | Poecilia reticulata | table 1(fit) and table 2 (gd) 2007 | Spearman\_Correlation | 3j | yes | obs | obs | Parasitism | adult | microsatellites | Non-kin | no | yes | NA | NA | 9 | 0.348 |
| 60 | Fraser, BA; Ramnarine, IW; Neff, BD | 2010 | TEMPORAL VARIATION AT THE MHC CLASS IIB IN WILD POPULATIONS OF THE GUPPY (POECILIA RETICULATA) | Evolution | Poecilia reticulata | table 1(fit) and table 2 (gd) 2007 | Spearman\_Correlation | 3f | yes | obs | obs | Parasitism | adult | microsatellites | Non-kin | no | yes | NA | NA | 9 | -0.247 |
| 60 | Fraser, BA; Ramnarine, IW; Neff, BD | 2010 | TEMPORAL VARIATION AT THE MHC CLASS IIB IN WILD POPULATIONS OF THE GUPPY (POECILIA RETICULATA) | Evolution | Poecilia reticulata | table 1(fit) and table 2 (gd) 2006 | Spearman\_Correlation | 3d | yes | obs | obs | Parasitism | adult | microsatellites | Non-kin | no | yes | NA | NA | 9 | -0.183 |
| 60 | Fraser, BA; Ramnarine, IW; Neff, BD | 2010 | TEMPORAL VARIATION AT THE MHC CLASS IIB IN WILD POPULATIONS OF THE GUPPY (POECILIA RETICULATA) | Evolution | Poecilia reticulata | table 1(fit) and table 2 (gd) 2006 | Spearman\_Correlation | 3h | yes | obs | obs | Parasitism | adult | microsatellites | Non-kin | no | yes | NA | NA | 9 | 0.017 |
| 61 | Neumann, P; Mortiz, RFA | 2000 | Testing genetic variance hypotheses for the evolution of Polyandrous in the honeybee (Apis mellifera L.) | Insectes Sociaux | Apis mellifera | fig 1b | Spearman\_Correlation | NA | yes | obs | obs | Parasitism | adult | microsatellites | Kin | no | no | NA | NA | 60 | -0.030 |
| 62 | van Houte, S; Ekroth, AKE; Broniewski, JM; Chabas, H; Ashby, B; Bondy-Denomy, J; Gandon, S; Boots, M; Paterson, S; Buckling, A; Westra, ER | 2016 | The diversity-generating benefits of a prokaryotic adaptive immune system | Nature | Pseudomonas aeruginosa | ext fig 1e | Means and SD | see var dataset | yes | exp | exp | Parasitism | adult\_juvenile | relatedness | Kin | yes | no | 6 | 48 | 76 | 0.437 |
| 62 | van Houte, S; Ekroth, AKE; Broniewski, JM; Chabas, H; Ashby, B; Bondy-Denomy, J; Gandon, S; Boots, M; Paterson, S; Buckling, A; Westra, ER | 2016 | The diversity-generating benefits of a prokaryotic adaptive immune system | Nature | Pseudomonas aeruginosa | ext fig 3g | Means and SD | see var dataset | no | exp | exp | Mortality | adult\_juvenile | relatedness | Kin | yes | no | 6 | 48 | 54 | -0.020 |
| 62 | van Houte, S; Ekroth, AKE; Broniewski, JM; Chabas, H; Ashby, B; Bondy-Denomy, J; Gandon, S; Boots, M; Paterson, S; Buckling, A; Westra, ER | 2016 | The diversity-generating benefits of a prokaryotic adaptive immune system | Nature | Pseudomonas aeruginosa | text | ANOVA | see var dataset | no | exp | exp | Mortality | adult\_juvenile | relatedness | Kin | yes | no | 6 | 48 | 54 | 0.241 |
| 62 | van Houte, S; Ekroth, AKE; Broniewski, JM; Chabas, H; Ashby, B; Bondy-Denomy, J; Gandon, S; Boots, M; Paterson, S; Buckling, A; Westra, ER | 2016 | The diversity-generating benefits of a prokaryotic adaptive immune system | Nature | Pseudomonas aeruginosa | fig 3 | Pearson\_Correlation | 5a | yes | exp | exp | Mortality | adult\_juvenile | relatedness | Kin | yes | no | NA | NA | 76 | 0.863 |
| 63 | SERA, WE; GAINES, MS | 1994 | The Effect of Relatedness on Spacing Behavior and Fitness of Female Prairie Voles | Ecology | Microtus ochrogaster | table 2 spring | Means and SD | NA | yes | obs | exp | Mortality | adult | relatedness | Kin | no | no | 6 | 6 | 12 | -0.060 |
| 63 | SERA, WE; GAINES, MS | 1994 | The Effect of Relatedness on Spacing Behavior and Fitness of Female Prairie Voles | Ecology | Microtus ochrogaster | table 2 fall | Means and SD | NA | yes | obs | exp | Mortality | adult | relatedness | Kin | no | no | 6 | 6 | 12 | 0.204 |
| 64 | Hoggard, SJ; Wilson, PD; Beattie, AJ; Stow, AJ | 2013 | The Effectiveness of Antimicrobial Defenses Declines With Increasing Group Size and Genetic Similarity | Annals of the Entomological Society of America | Polistes humilis | table 3 | Pearson\_Correlation | NA | yes | obs | obs | Parasitism | adult | microsatellites | Kin | no | no | NA | NA | 10 | 0.339 |
| 65 | WAUTERS, LA; HUTCHINSON, Y; PARKIN, DT; DHONDT, AA | 1994 | THE EFFECTS OF HABITAT FRAGMENTATION ON DEMOGRAPHY AND ON THE LOSS OF GENETIC-VARIATION IN THE RED SQUIRREL | Proc. R. Soc. Lond. B | Sciurus vulgaris | table 1 & 2 adults 91 | Pearson\_Correlation | 7a | yes | obs | obs | Mortality | adult | minisatellites | Non-kin | no | yes | NA | NA | 5 | 0.048 |
| 65 | WAUTERS, LA; HUTCHINSON, Y; PARKIN, DT; DHONDT, AA | 1994 | THE EFFECTS OF HABITAT FRAGMENTATION ON DEMOGRAPHY AND ON THE LOSS OF GENETIC-VARIATION IN THE RED SQUIRREL | Proc. R. Soc. Lond. B | Sciurus vulgaris | table 1 & 2 juveniles 91 | Pearson\_Correlation | 7b | yes | obs | obs | Mortality | juvenile | minisatellites | Non-kin | no | yes | NA | NA | 5 | -0.138 |
| 65 | WAUTERS, LA; HUTCHINSON, Y; PARKIN, DT; DHONDT, AA | 1994 | THE EFFECTS OF HABITAT FRAGMENTATION ON DEMOGRAPHY AND ON THE LOSS OF GENETIC-VARIATION IN THE RED SQUIRREL | Proc. R. Soc. Lond. B | Sciurus vulgaris | table 1 & 2 adults 92 | Pearson\_Correlation | 7c | yes | obs | obs | Mortality | adult | minisatellites | Non-kin | no | yes | NA | NA | 5 | -0.054 |
| 65 | WAUTERS, LA; HUTCHINSON, Y; PARKIN, DT; DHONDT, AA | 1994 | THE EFFECTS OF HABITAT FRAGMENTATION ON DEMOGRAPHY AND ON THE LOSS OF GENETIC-VARIATION IN THE RED SQUIRREL | Proc. R. Soc. Lond. B | Sciurus vulgaris | table 1 & 2 juveniles 92 | Pearson\_Correlation | 7d | yes | obs | obs | Mortality | juvenile | minisatellites | Non-kin | no | yes | NA | NA | 5 | -0.008 |
| 66 | Solazzo, G; Moritz, RFA; Settele, J | 2014 | The social parasite Phengaris (Maculinea) nausithous affects genetic diversity within Myrmica rubra host ant colonies | Journal of Insect Conservation | Myrmica rubra | result text | Means and SD | NA | yes | obs | obs | Parasitism | adult | microsatellites | Kin | no | no | 3 | 6 | 9 | 0.324 |
| 66 | Solazzo, G; Moritz, RFA; Settele, J | 2014 | The social parasite Phengaris (Maculinea) nausithous affects genetic diversity within Myrmica rubra host ant colonies | Journal of Insect Conservation | Myrmica rubra | fig 2, result text | Means and SD | NA | yes | obs | obs | Parasitism | adult | microsatellites | Kin | no | no | 12 | 24 | 36 | -0.003 |
| 67 | Agashe, Deepa | 2009 | The Stabilizing Effect of Intraspecific Genetic Variation on Population Dynamics in Novel and Ancestral Habitats | The American Naturalist | Tribolium castaneum | fig 1a + text corn treatment | Pearson\_Correlation | NA | no | obs | exp | Mortality | adult | relatedness | Non-kin | yes | no | NA | NA | 43 | 0.755 |
| 67 | Agashe, Deepa | 2009 | The Stabilizing Effect of Intraspecific Genetic Variation on Population Dynamics in Novel and Ancestral Habitats | The American Naturalist | Tribolium castaneum | fig 1a + text wheat treatment | Pearson\_Correlation | NA | no | obs | exp | Mortality | adult | relatedness | Non-kin | yes | no | NA | NA | 43 | 0.445 |
| 68 | Baer, B; Schmid-Hempel, P | 2001 | Unexpected consequences of Polyandrous for parasitism and fitness in the bumblebee, Bombus terrestris | Evolution | Bombus terrestris | fig 1 | Means and SD | NA | yes | obs | exp | Parasitism | adult | relatedness | Kin | no | no | 6 | 6 | 12 | 0.355 |
| 68 | Baer, B; Schmid-Hempel, P | 2001 | Unexpected consequences of Polyandrous for parasitism and fitness in the bumblebee, Bombus terrestris | Evolution | Bombus terrestris | fig 1 | Means and SD | NA | yes | obs | exp | Parasitism | adult | relatedness | Kin | no | no | 3 | 3 | 6 | 0.102 |
| 68 | Baer, B; Schmid-Hempel, P | 2001 | Unexpected consequences of Polyandrous for parasitism and fitness in the bumblebee, Bombus terrestris | Evolution | Bombus terrestris | fig 1 | Means and SD | NA | yes | obs | exp | Parasitism | adult | relatedness | Kin | no | no | 3 | 6 | 9 | 0.124 |
| 68 | Baer, B; Schmid-Hempel, P | 2001 | Unexpected consequences of Polyandrous for parasitism and fitness in the bumblebee, Bombus terrestris | Evolution | Bombus terrestris | fig 1 | Means and SD | NA | yes | obs | exp | Parasitism | adult | relatedness | Kin | no | no | 3 | 6 | 9 | 0.018 |
| 68 | Baer, B; Schmid-Hempel, P | 2001 | Unexpected consequences of Polyandrous for parasitism and fitness in the bumblebee, Bombus terrestris | Evolution | Bombus terrestris | fig 1 | Means and SD | NA | yes | obs | exp | Parasitism | adult | relatedness | Kin | no | no | 6 | 3 | 9 | 0.179 |
| 68 | Baer, B; Schmid-Hempel, P | 2001 | Unexpected consequences of Polyandrous for parasitism and fitness in the bumblebee, Bombus terrestris | Evolution | Bombus terrestris | fig 1 | Means and SD | NA | yes | obs | exp | Parasitism | adult | relatedness | Kin | no | no | 3 | 3 | 6 | 0.238 |
| 68 | Baer, B; Schmid-Hempel, P | 2001 | Unexpected consequences of Polyandrous for parasitism and fitness in the bumblebee, Bombus terrestris | Evolution | Bombus terrestris | fig 1 | Means and SD | NA | yes | obs | exp | Parasitism | adult | relatedness | Kin | no | no | 6 | 6 | 12 | 0.529 |
| 68 | Baer, B; Schmid-Hempel, P | 2001 | Unexpected consequences of Polyandrous for parasitism and fitness in the bumblebee, Bombus terrestris | Evolution | Bombus terrestris | fig 1 | Means and SD | NA | yes | obs | exp | Parasitism | adult | relatedness | Kin | no | no | 6 | 3 | 9 | 0.241 |
| 68 | Baer, B; Schmid-Hempel, P | 2001 | Unexpected consequences of Polyandrous for parasitism and fitness in the bumblebee, Bombus terrestris | Evolution | Bombus terrestris | fig 1 | Means and SD | NA | yes | obs | exp | Parasitism | adult | relatedness | Kin | no | no | 6 | 3 | 8 | 0.456 |
| 68 | Baer, B; Schmid-Hempel, P | 2001 | Unexpected consequences of Polyandrous for parasitism and fitness in the bumblebee, Bombus terrestris | Evolution | Bombus terrestris | fig 1 | Means and SD | NA | yes | obs | exp | Parasitism | adult | relatedness | Kin | no | no | 6 | 3 | 8 | 0.340 |
| 68 | Baer, B; Schmid-Hempel, P | 2001 | Unexpected consequences of Polyandrous for parasitism and fitness in the bumblebee, Bombus terrestris | Evolution | Bombus terrestris | fig 1 | Means and SD | NA | yes | obs | exp | Parasitism | adult | relatedness | Kin | no | no | 3 | 6 | 9 | 0.000 |
| 68 | Baer, B; Schmid-Hempel, P | 2001 | Unexpected consequences of Polyandrous for parasitism and fitness in the bumblebee, Bombus terrestris | Evolution | Bombus terrestris | fig 1 | Means and SD | NA | yes | obs | exp | Parasitism | adult | relatedness | Kin | no | no | 3 | 6 | 9 | 0.291 |
| 69 | Woyciechowski, M; Krol, E | 2001 | Worker genetic diversity and infection by Nosema apis in honey bee colonies | FOLIA BIOLOGICA-KRAKOW | Apis mellifera | text | Mann\_Whitney | NA | yes | obs | exp | Parasitism | adult | relatedness | Kin | no | no | 5 | 5 | 10 | -0.163 |
| 70 | Bensch, H; Cornwallis, C. K. | 2017 | NA | NA | Struthio camelus | our own study. See ostrich.csv in inputfiles folder | Pearson\_Correlation | 11a | yes | obs | exp | Mortality | juvenile | relatedness | Non-kin | no | no | NA | NA | 45 | 0.030 |
| 71 | Winternitz, JC; Wares, JP; Yabsley, MJ; Altizer, S | 2014 | Wild cyclic voles maintain high neutral and MHC diversity without strong evidence for parasite-mediated selection | Evolutionary Ecology | Microtus montanus | Table S7 | Linear\_Regression | 10a | yes | obs | obs | Parasitism | adult | microsatellites | Non-kin | no | no | NA | NA | 4 | -0.104 |
| 71 | Winternitz, JC; Wares, JP; Yabsley, MJ; Altizer, S | 2014 | Wild cyclic voles maintain high neutral and MHC diversity without strong evidence for parasite-mediated selection | Evolutionary Ecology | Microtus montanus | Table S7 | Linear\_Regression | 10b | yes | obs | obs | Parasitism | adult | microsatellites | Non-kin | no | no | NA | NA | 4 | -0.108 |
| 71 | Winternitz, JC; Wares, JP; Yabsley, MJ; Altizer, S | 2014 | Wild cyclic voles maintain high neutral and MHC diversity without strong evidence for parasite-mediated selection | Evolutionary Ecology | Microtus montanus | Table S7 | Linear\_Regression | 10e | yes | obs | obs | Parasitism | adult | microsatellites | Non-kin | no | no | NA | NA | 4 | -0.277 |
| 71 | Winternitz, JC; Wares, JP; Yabsley, MJ; Altizer, S | 2014 | Wild cyclic voles maintain high neutral and MHC diversity without strong evidence for parasite-mediated selection | Evolutionary Ecology | Microtus montanus | Table S7 | Linear\_Regression | 10c | yes | obs | obs | Parasitism | adult | microsatellites | Non-kin | no | no | NA | NA | 4 | -0.131 |
| 71 | Winternitz, JC; Wares, JP; Yabsley, MJ; Altizer, S | 2014 | Wild cyclic voles maintain high neutral and MHC diversity without strong evidence for parasite-mediated selection | Evolutionary Ecology | Microtus montanus | Table S7 | Linear\_Regression | 10d | yes | obs | obs | Parasitism | adult | microsatellites | Non-kin | no | no | NA | NA | 4 | 0.105 |
| 72 | Siemens, DH; Roy, BA | 2005 | Tests for parasite-mediated frequency-dependent selection in natural populations of an asexual plant species | Evolutionary Ecology | Arabis holboellii | table 1 & fig 1a | ANOVA | NA | yes | obs | obs | Parasitism | adult | allozyme | Kin | no | no | NA | NA | 4 | 0.796 |
| 73 | Ellison, Amy; Cable, Jo; Consuegra, Sofia | 2011 | Best of both worlds? Association between outcrossing and parasite loads in a selfing fish | Evolution | Kryptolebias marmoratus | table 1 | Pearson\_Correlation | 4g | yes | obs | obs | Parasitism | adult | microsatellites | Kin | no | no | NA | NA | 4 | 0.187 |
| 74 | Strauss, Alexander; Hite, Jessica; Shocket, Marta; Caceres, Carla; Duffy, Meghan; Hall, Spencer | 2017 | Rapid evolution rescues hosts from competition and disease butdespite a dilution effectincreases the density of infected hosts | Proc. R. Soc. Lond. B | Daphnia dentifera | fig 2. parasites with competition | Means and SD | NA | yes | exp | exp | Mortality | adult | genotypes | Kin | yes | no | 5 | 5 | 10 | 0.332 |
| 74 | Strauss, Alexander; Hite, Jessica; Shocket, Marta; Caceres, Carla; Duffy, Meghan; Hall, Spencer | 2017 | Rapid evolution rescues hosts from competition and disease butdespite a dilution effectincreases the density of infected hosts | Proc. R. Soc. Lond. B | Daphnia dentifera | stats from fig s1 | ANOVA | NA | yes | exp | exp | Parasitism | adult | genotypes | Kin | yes | no | 5 | 5 | 10 | 0.530 |
| 74 | Strauss, Alexander; Hite, Jessica; Shocket, Marta; Caceres, Carla; Duffy, Meghan; Hall, Spencer | 2017 | Rapid evolution rescues hosts from competition and disease butdespite a dilution effectincreases the density of infected hosts | Proc. R. Soc. Lond. B | Daphnia dentifera | fig 2. parasites without competition | Means and SD | NA | yes | exp | exp | Mortality | adult | genotypes | Kin | yes | no | 5 | 5 | 10 | 0.359 |
| 74 | Strauss, Alexander; Hite, Jessica; Shocket, Marta; Caceres, Carla; Duffy, Meghan; Hall, Spencer | 2017 | Rapid evolution rescues hosts from competition and disease butdespite a dilution effectincreases the density of infected hosts | Proc. R. Soc. Lond. B | Daphnia dentifera | fig 2. control with compeititon | Means and SD | NA | no | exp | exp | Mortality | adult | genotypes | Kin | yes | no | 5 | 5 | 10 | -0.123 |
| 74 | Strauss, Alexander; Hite, Jessica; Shocket, Marta; Caceres, Carla; Duffy, Meghan; Hall, Spencer | 2017 | Rapid evolution rescues hosts from competition and disease butdespite a dilution effectincreases the density of infected hosts | Proc. R. Soc. Lond. B | Daphnia dentifera | fig 2. control without competition | Means and SD | NA | no | exp | exp | Mortality | adult | genotypes | Kin | yes | no | 5 | 5 | 10 | -0.569 |
| 75 | de Morais, Claire Tito; Kettle, Chris J; Philipson, Christopher D; Maycock, Colin R; Burslem, David F. R. P; Khoo, Eyen; Ghazoul, Jaboury | 2020 | Exploring the Role of Genetic Diversity and Relatedness in Tree Seedling Growth and Mortality: A Multispecies Study in a Bornean Rainforest | Journal of Ecology | Parashorea tomentella | Table S4 | GLMM | NA | yes | obs | obs | Mortality | juvenile | microsatellites | Kin | no | no | NA | NA | 8 | 0.111 |
| 75 | de Morais, Claire Tito; Kettle, Chris J; Philipson, Christopher D; Maycock, Colin R; Burslem, David F. R. P; Khoo, Eyen; Ghazoul, Jaboury | 2020 | Exploring the Role of Genetic Diversity and Relatedness in Tree Seedling Growth and Mortality: A Multispecies Study in a Bornean Rainforest | Journal of Ecology | Shorea acuminatissima | Table S4 | GLMM | NA | yes | obs | obs | Mortality | juvenile | microsatellites | Kin | no | yes | NA | NA | 8 | -0.004 |
| 75 | de Morais, Claire Tito; Kettle, Chris J; Philipson, Christopher D; Maycock, Colin R; Burslem, David F. R. P; Khoo, Eyen; Ghazoul, Jaboury | 2020 | Exploring the Role of Genetic Diversity and Relatedness in Tree Seedling Growth and Mortality: A Multispecies Study in a Bornean Rainforest | Journal of Ecology | Shorea argentifolia | Table S4 | GLMM | NA | yes | obs | obs | Mortality | juvenile | microsatellites | Kin | no | no | NA | NA | 8 | -0.276 |
| 75 | de Morais, Claire Tito; Kettle, Chris J; Philipson, Christopher D; Maycock, Colin R; Burslem, David F. R. P; Khoo, Eyen; Ghazoul, Jaboury | 2020 | Exploring the Role of Genetic Diversity and Relatedness in Tree Seedling Growth and Mortality: A Multispecies Study in a Bornean Rainforest | Journal of Ecology | Shorea gibbosa | Table S4 | GLMM | NA | yes | obs | obs | Mortality | juvenile | microsatellites | Kin | no | yes | NA | NA | 8 | -0.859 |
| 76 | Abdi, Mohamed Khadar and Lupi, Daniela and Hardy, Ian C. W | 2020 | Co-Foundress Confinement Elicits Kinship Effects in a Naturally Sub-Social Parasitoid | Journal of Evolutionary Biology | Goniozus nephantidis | text | GLMM | NA | no | exp | exp | Mortality | juvenile | relatedness | Kin | yes | no | NA | NA | 184 | -0.100 |
| 76 | Abdi, Mohamed Khadar and Lupi, Daniela and Hardy, Ian C. W | 2020 | Co-Foundress Confinement Elicits Kinship Effects in a Naturally Sub-Social Parasitoid | Journal of Evolutionary Biology | Goniozus nephantidis | Fig 1 & text | GLM | NA | no | exp | exp | Mortality | adult | relatedness | Kin | yes | no | 110 | 110 | 220 | -0.394 |
| 77 | Mott, CL; Dzaferbegovic, H; Timm, SR; Whiteman, HH | 2019 | Influences of facultative paedomorphosis on kin selection in a larval salamander, Ambystoma talpoideum | Behavior | Ambystoma talpoideum | Fig 3b | ANOVA | NA | yes | obs | exp | Mortality | juvenile | relatedness | Non-kin | yes | no | 15 | 15 | 30 | -0.581 |
| 78 | Parsche, S; Lattorff, HMG | 2018 | The relative contributions of host density and genetic diversity on prevalence of a multi-host parasite in bumblebees | Biological Journal of the Linnean Society | Bombus lapidarius | table 3 | Multiple\_Regression\_Ttest | NA | yes | obs | obs | Parasitism | adult | microsatellites | Kin | no | no | NA | NA | 362 | 0.056 |
| 78 | Parsche, S; Lattorff, HMG | 2018 | The relative contributions of host density and genetic diversity on prevalence of a multi-host parasite in bumblebees | Biological Journal of the Linnean Society | Bombus lapidarius | Fig 2c, table 3 & text | Multiple\_Regression\_Ttest | NA | yes | obs | obs | Parasitism | adult | microsatellites | Kin | no | no | NA | NA | 362 | -0.051 |
| 78 | Parsche, S; Lattorff, HMG | 2018 | The relative contributions of host density and genetic diversity on prevalence of a multi-host parasite in bumblebees | Biological Journal of the Linnean Society | Bombus terrestris | Fig 2c, table 3 & text | Multiple\_Regression\_Ttest | NA | yes | obs | obs | Parasitism | adult | microsatellites | Kin | no | no | NA | NA | 396 | 0.221 |
| 78 | Parsche, S; Lattorff, HMG | 2018 | The relative contributions of host density and genetic diversity on prevalence of a multi-host parasite in bumblebees | Biological Journal of the Linnean Society | Bombus terrestris | table 3 | Multiple\_Regression\_Ttest | NA | yes | obs | obs | Parasitism | adult | microsatellites | Kin | no | no | NA | NA | 396 | 0.105 |
| 79 | Dagan, Y; Kosman, E; Ben-Ami, F | 2017 | Cost of resistance to trematodes in freshwater snail populations with low clonal diversity | BMC Ecology | Melanoides tuberculata | table 2 | Multiple\_Regression\_Ftest | NA | no | exp | obs | Mortality | adult | allozyme | Kin | yes | no | NA | NA | 30 | -0.938 |
| 79 | Dagan, Y; Kosman, E; Ben-Ami, F | 2017 | Cost of resistance to trematodes in freshwater snail populations with low clonal diversity | BMC Ecology | Melanoides tuberculata | table 2 | Multiple\_Regression\_Ftest | NA | no | exp | obs | Mortality | adult | allozyme | Kin | yes | no | NA | NA | 30 | -0.253 |

## **Table S3:** Data on whether species typically live with kin or non-kin.

| Kingdom | Phylum | Class | Order | Family | Genus | Specific | Species | Stage Zr | Relatedness notes | Relatedness reference | Mating system | Mating system notes | Mating system reference | Dispersal notes | Dispersal reference | Kin strcuture | Reference.Number | Species | Year | Author | Title | Source |
| --- | --- | --- | --- | --- | --- | --- | --- | --- | --- | --- | --- | --- | --- | --- | --- | --- | --- | --- | --- | --- | --- | --- |
| Animalia | Arthropoda | Insecta | Hymenoptera | Formicidae | Monomorium | pharaonis | Monomorium pharaonis | Ad | “Pharaoh ants form large polygynous colonies” & “New queens and males mate within their natal nest (all queens are singly inseminated)” & “gene flow between colonies may therefore be rare” & “Microsatellite markers have previously been employed to show that M. pharaonis colonies, both from laboratories and from the field, have very low levels of genetic diversity” (188). Probably monoandrous (Appenidx 1 Table, 189) | 191, 192 | Monoandry | Appendix 1 Table suggests monoandry | 192 | NA | NA | Kin | 1 | Kryptolebias marmoratus | 2015 | Avise | Population genetics and evolution of the mangrove rivulus Kryptolebias marmoratus, the world’s only self-fertilizing hermaphroditic vertebrate | Journal of Fish Biology |
| Animalia | Arthropoda | Insecta | Hymenoptera | Apidae | Bombus | lapidarius | Bombus lapidarius | Ad | “Mating frequencies in social Hymenoptera are particularly interesting because of their influence on colony relatedness patterns” & “the pattern found in B. terrestris, B. lucorum, B. pratorum, B. lapidarius, B. sicheli, B. hortorum, and B. pascuorum was compatible with single mating” (30); Single queen colonies Table 1 (187) | 30, 190 | Monoandry | “the pattern found in B. terrestris, B. lucorum, B. pratorum, B. lapidarius, B. sicheli, B. hortorum, and B. pascuorum was compatible with single mating” | 30 | NA (colonial) | NA (colonial) | Kin | 2 | Kryptolebias marmoratus | 2006 | Mackiewicz | A mixed-mating strategy in a hermaphroditic vertebrate | Proc Roy B |
| Animalia | Arthropoda | Insecta | Hymenoptera | Bethylidae | Goniozus | nephantidis | Goniozus nephantidis | Ad\_Juv | 187. Goniozus are sub-social, with single foundress females exhibiting post-ovipositional maternal care via short-term aggressive host and brood defence against conspecific females. | 186 | Monoandry | “Goniozus are sub-social, with single foundress females exhibiting post-ovipositional maternal care via short-term aggressive host and brood defence against conspecific females.” (187) | 187 | NA | NA | Kin | 5 | Mus musculus | 2006 | Dean | The frequency of multiple paternity suggests that sperm competition is common in house mice (Mus domesticus) | Molecular Ecology |
| Animalia | Arthropoda | Malacostraca | Ampioda | Gammaridae | Gammarus | duebeni | Gammarus duebeni | Ad | “Gammarus duebeni is a routinely outbreeding invertebrate” (114); Although they can occur in dense aggregations, they are unlikely to form stable social groups with kin: “Many species are highly mobile, being constantly on the move in search of food and mates” & “To date, there are no documented cases of familiar, true individual, or self-recognition in Amphipoda.” (113) | 113, 114 | Monogamy | Form pairs with prolonged male mate-guarding. 90% brood fathered my same male in closely related G. plurex. (112); “We are not aware of any reports of multiple paternity in G. duebeni” (114) | 112, 114 | Some limited dispersal: “A process involving restricted gene flow with some long distance dispersal was invoked for divergence between the basal amphi-gene flow with some long distance dispersal was invoked for divergence between the basal amphi- Atlantic grouping (hap 2; see Table 2, Figure 2) and invoked for divergence between the basal amphi- Atlantic grouping (hap 2; see Table 2, Figure 2) and populations in Rhode Island (hap 8), Plymouth (hap 6) Atlantic grouping (hap 2; see Table 2, Figure 2) and populations in Rhode Island (hap 8), Plymouth (hap 6) and Isle of Man (hap 10). Divergence within the overall populations in Rhode Island (hap 8), Plymouth (hap 6) and Isle of Man (hap 10). Divergence within the overall marine clade was characterized by restricted gene flow with isolation by distance from the ancestral haplotype” (109) | 109 | Non-kin | 6 | Apodemus flavicollis | 2008 | Bryja | Varying levels of female promiscuity in four Apodemus mice species | Behavioral Ecology and Sociobiology |
| Animalia | Arthropoda | Insecta | Hymenoptera | Apidae | Bombus | terrestris | Bombus terrestris | Ad | Monandry - “the pattern found in B. terrestris, B. lucorum, B. pratorum, B. lapidarius, B. sicheli, B. hortorum, and B. pascuorum was compatible with single mating” (30), large grp size (32). | 30, 32 | Monogamy | “the pattern found in B. terrestris, B. lucorum, B. pratorum, B. lapidarius, B. sicheli, B. hortorum, and B. pascuorum was compatible with single mating” (30) | 30 | NA (colonial) | NA (colonial) | Kin | 7 | Mus musculus | 1993 | Konig | Maternal investment of communally nursing mice | Behavioural Processes |
| Animalia | Arthropoda | Insecta | Hymenoptera | Formicidae | Formica | selysi | Formica selysi | Ad | Variable - Monandry, but can have multiple queens: “About two-thirds (63%) of the 112 nests analyzed were headed by a single queen, usually mated to a single male, and the rest of the nests (37%) had multiple queens. In these polygynous nests, the effective number of queens was 4.3, and the actual number of queens was likely to be much greater than this value if queens were related or if their shares of reproduction were unequal (e.g. Chapuisat and Crozier 2001).” (53) | 53 | Monogamy | “About two-thirds (63%) of the 112 nests analyzed were headed by a single queen, usually mated to a single male” | 53 | NA (colonial) | NA (colonial) | Kin | 9 | Kryptolebias marmoratus | 2012 | Edenbrow | Kin and familiarity influence association preferences and aggression in the mangrove killifish Kryptolebias marmoratus | J Fish Biology |
| Animalia | Arthropoda | Insecta | Hymenoptera | Vespodae | Polistes | humilis | Polistes humilis | Ad | “Our genetic data show that colonies of Polistes humilis are characterised by multiple queens and that queen number increases with colony size. Within a colony each queen is singly mated by a different male and this may explain why reproductive females are not full sisters” (138) | 138 | Monogamy | “Within a colony each queen is singly mated by a different male” (138) | 138 | NA (colonial) | NA (colonial) | Kin | 11 | Apodemus flavicollis | 2007 | Simeonovska-Nikolova | Interspecific social interactions and behavioral responses of Apodemus agrarius and Apodemus flavicollis to conspecific and heterospecific odors | J Ethology |
| Animalia | Chordata | Aves | Passeriformes | Passeridae | Passer | domesticus | Passer domesticus | Ad | “We found that most birds were unrelated to each other in the flocks (mean ± SE relatedness coefficient: 0.06 ± 0.002), although most individuals had at least a few close relatives in their home flock (14.3 ± 0.6% of flock-mates).”. Looked at stable flock members. same individuals resident in same area, so regularly interact (193) | 193 | Monogamy | Monogamous pair breeders. Low rate of EPP “the proportion of extrapair offspring varies between geographic populations, ranging from 1 up to 20%” (e.g., nesting habitats and climate; see Cramp, 1994)" (73) | 73 | “As dispersal is often limited in the species (Summers-Smith 1963; Altwegg et al. 2000; Anderson 2006; but see Fleischer et al. 1984), related individuals may regularly forage and roost as members of the same flock” (193) | 193 | Non-kin | 14 | Poecilia reticulata | 2004 | Russell | Kin structure and shoal composition dynamics in the guppy, Poecilia reticulata | Oikos |
| Animalia | Arthropoda | Insecta | Hymenoptera | Formicidae | Myrmica | rubra | Myrmica rubra | Ad | “Relatedness among worker nestmates varied from 0 to 0.82. The most important reason for this variation was the extensive variation in the queen number among populations. Most populations were moderately or highly polygynous resulting in low relatedness among worker nestmates, but effectively monogynous populations were also found.” (144) | 144 | Monogamy | “This gives the proportion of multiply mated mothers as 17%.” (144) Predominantly monogamy | 144 | NA (colonial) | NA (colonial) | Kin | 17 | Acromyrmex echinatior | 2014 | Sturup | Sperm mixing in the polyandrous leaf-cutting ant Acromyrmex echinatior | Ecology & Evolution |
| Animalia | Chordata | Mammalia | Rodentia | Cricetidae | Microtus | townsendii | Microtus townsendii | Ad | “Females were more philopatric than males and females thought to be members of the same family group lived adjacent to each other or had overlapping home ranges.” & “Thirty-five percent of the philopatric females became pregnant for the first time when the male spatially associated with their mother in the spring was still alive and thus could potentially have mated with their fathers”. &“Groups of related breeding females used adjacent and partly overlapping ranges.” (78) | 78 | Monogamy | “The 1 : 1 operational sex ratio and the spatial association of pairs of males and females suggest that a monogamous mating system was prevalent” (78) | 78 | “Females were more philopatric than males and females thought to be members of the same family group lived adjacent to each other or had overlapping home ranges.” (78) | 78 | Kin | 23 | Ambystoma opacum | 2009 | Croshaw | Comparing the performance of analytical techniques for genetic parentage of half-sib progeny arrays | Genetic Research |
| Animalia | Chordata | Mammalia | Rodentia | Sciurudae | Sciurus | vulgaris | Sciurus vulgaris | Ad | Singeltons defend territories & do not interact much with related individuals as adults: males & females defend individual territories (66);“Sciurus vulgaris, S. carolinensis and Tamiasciurus hudsonicus, which also remain solitary as adults except for a short pairing time during the mating season” (67); “Dispersal in red squirrels (Sciurus vulgaris) is not sex-biased and strict philopatry is rare.” (194) | 66, 67, 194 | Monogamy | “although multiple mating existed it was rare. The mean number of males mating with a female was 1.12” (182) | 182 | “Dispersal in red squirrels (Sciurus vulgaris) is not sex-biased and strict philopatry is rare.” (194) | 194 | Non-kin | 30 | Bombus terrestris/Bombus lapidarius | 2000 | Schmid-Hempel | Female mating frequencies in Bombus spp. from Central Europe | Insect Sociaux |
| Animalia | Arthropoda | Insecta | Hymenoptera | Formicidae | Cardiocondyla | obscurior | Cardiocondyla obscurior | Ad | “84% of Cardiocondyla obscurior (YY Du and A Schrempf, unpublished) mated with their brothers” (45); “Generally, relatedness varied greatly among colonies and in some cases was surprisingly low. Overall, worker relatedness and queen relatedness are not different from each other (worker relatedness: 0.580 ± SE 0.127 (102 viduals); t-test for paired samples: t =−0.22, P=0.83; see individuals), queen relatedness: 0.612 ± SE 0.366 (61 indi- table 2). Relatively low relatedness among queens in some of the colonies (colonies OypC48, OypE3, OypE5 and OypE4, r <0.35) might indicate that alien queens can be easily adopted in the colonies as has already been suggested by Kinomura and Yamauchi (1987)” & “disperse, wingless males stay inside their maternal nests throughout their lives and mate with young nestmate queens (Kinomura and Yamauchi 1987;Stuart et al. 1987). This results in considerable inbreeding: from fixation coefficients in single-queen colonies of noninvasive Cardiocondyla,from Mediterranean Europe and Madagascar it has been estimated that up to 90% of the mating are between brothers and sisters (Schrempf et al. 2005, 2014; Lenoir et al. 2007;Heinze et al. 2014)” & “Their colonies typically contain multiple queens” (178); “Several studies point to an important role of seminal fluids in determining fitness of the mostly monandrous C. obscurior queens” (48); | 45, 48, 178 | Monogamy | “Several studies point to an important role of seminal fluids in determining fitness of the mostly monandrous C. obscurior queens.” | 48 | NA (colonial) | NA (colonial) | Kin | 32 | Bombus terrestris | 2002 | Goulson | Colony growth of the bumblebee, Bombus terrestris, in improved and conventional agricultural and suburban habitats | Oecologia |
| Animalia | Chordata | Amphibia | Anura | Ranidae | Rana | latastei | Rana latastei | Juv | Multiple clutches per population/pond (table 1 of Ref 58) | 58 | Monogamy | “Multiple paternity in a R. latastei clutch has not been described; therefore, each clutch was assumed to be a full-sib family.” (58) | 58 | NA | NA | Non-kin | 36 | Capreolus capreolus | 2009 | Vanpe | Multiple paternity occurs with low frequency in the territorial roe deer, Capreolus capreolus | Biological Journal of the Linnean Society |
| Animalia | Chordata | Ascidiacea | Enterogona | Cionidae | Ciona | intestinalis | Ciona intestinalis | Ad | “Adult C. intestinalis are often found in dense (>5000 individuals mâˆ’2) monocultures” (99); “Local recruitment is accomplished by producing mucus strings which contain spawned eggs. Such eggs are virtually always fertilized and subsequently deposited among the adults or caught on other protruding objects.” (184) | 99, 184 | Outcrossing | “both sperm and eggs are broadcast into the surrounding environment” (99); “In the ascidian, Ciona intestinalis, the gametes of an individual are usually self-sterile” (100) | 99, 100 | likely that fertilzed eggs do not disperse very far and there are large aggregations of related individuals: “Thus, the dense aggregations of both adults and newly settled Cionn which are commonly observed in the field could be explained by abbreviated dispersal due to mucus string spawning and epibenthic retention of eggs.” (184) | 184 | Kin | 45 | Cardiocondyla obscurior | 2006 | Schrempf | Sex determination and inbreeding depression in an ant with regular sib-mating | Heredity |
| Animalia | Nematoda | Chromadorea | Rhabditida | Steinernematidae | Steinernema | longicaudum | Steinernema longicaudum | Ad | “A host can be infected either by a group of Infective Juveniles (IJs) originating from a single host, single IJs originating from different hosts and all the possible scenarios in between these two extremes, leading to a considerable variation of relatedness among competitor males.” (160); “A single insect cadaver can support several generations and produce up to 300,000 nematodes” (161) | 160, 161 | Outcrossing | Not hermaphrodite “Steinernema IJ develops into either a male or female” (163); Females may only mate once due to sexual plugs | 163 | NA | NA | Kin | 46 | Ant: Formica lemani | 2009 | Seppa | Population genetics of the black ant Formica lemani (Hymenoptera: Formicidae) | Biological Journal of the Linnean Society |
| Animalia | Arthropoda | Insecta | Lepidoptera | Lycaenidae | Maculinea | nausithous | Maculinea nausithous | Juv | Females lay eggs on flowers of Sanguisorba officinalis (171), so juveniles likely to interact with siblings =>0.25. | 171 | Polyandry | Not known but closey realted spp M.arion probably polyandrous (173). | 173 | Females lay eggs on flowers of Sanguisorba officinalis, which hatch into caterpillars and remain on flower eating the seeds (171) | 171 | Kin | 47 | Ant: Formica lemani | 2007 | Gardner | Increased genetic diversity as a defence against parasites is undermined by social parasites: Microdon mutabilis hoverflies infesting Formica lemani ant colonies | ProcB |
| Animalia | Arthropoda | Insecta | Hymenoptera | Formicidae | Formica | lemani | Formica lemani | Ad | “In most of our study populations, the average worker nestmate relatedness was consistent with the hypothesis of a single pair breeding in the nests. However, in some populations, the average worker nestmate relatedness was significantly lower than the expected for the offspring of a single pair (r = 0.75).” & “The genetically effective queen number varied in the range 1.0-1.6 (Table 4) and the average across all populations was 1.28 (weighed with sample size).” (46) | 46 | Polyandry | “at least facultatively polygynous” & Fig 3 (47) | 47 | NA (colonial) | NA (colonial) | Kin | 48 | Cardiocondyla obscurior | 2016 | Oettler | Fitness and aging in Cardiocondyla obscurior ant queens | Current Opinion in Insect Science |
| Animalia | Arthropoda | Insecta | Lepidoptera | Lasiocampisae | Malacosoma | californicum | Malacosoma californicum pluviale | Juv | Live in tight family grps “They are gregarious and females lay a single egg mass and larvae stay together during the larval stages to form a tent.” But multiple paternity (average 1.92 sires = highR). (166). live in large family grps of at least 80 larvae (“4th instar larvae were removed from all families for a total of 80 larvae from each family” (167) | 166, 167 | Polyandry | “In our study, the minimum number of sires per family ranged from one to three, with an average of two fathers per family.” | 166 | NA | NA | Kin | 53 | Formica selysi | 2004 | Chapuisat | Variable Queen Number in Ant Colonies : No Impact on Queen Turnover , Inbreeding , and Population Genetic Differentiation in the Ant Formica selysi | Evolution |
| Animalia | Arthropoda | Insecta | Hymenoptera | Vespodae | Vespula | vulgaris | Vespula vulgaris | Ad | “All workers in a nest had microsatellite genotypes consistent with a single mother, suggesting that no foreign queens or workers had been recruited. Two to three patrilines were observed in each colony.” (140) | 140 | Polyandry | “All workers in a nest had microsatellite genotypes consistent with a single mother, suggesting that no foreign queens or workers had been recruited. Two to three patrilines were observed in each colony.” | 140 | NA (colonial) | NA (colonial) | Kin | 55 | Crinia georgiana | 2000 | Byrne | Does multiple paternity improve fitness of the frog Crinia georgiana? | Evolution |
| Animalia | Chordata | Actinopterygii | Cyprinodontiformes | Poeciliidae | Poecilia | reticulata | Poecilia reticulata | Ad | “Relatedness levels within each shoal did not differ significantly from zero, indicating that kin selection does not contribute to gross structure” (14) | 14 | Polyandry | “We detected multiple paternity in all ten populations” (180) | 180 | NA | NA | Non-kin | 58 | Rana latastei | 2010 | Ficetola | Polygyny, census and effective population size in the threatened frog, Rana latastei | Animal Conservation |
| Animalia | Arthropoda | Insecta | Coleoptera | Tenebrionidae | Tribolium | castaneum | Tribolium castaneum | Ad | promiscuous mating “Males as well as females mate with multiple partners” (158); don’t seem to live in stable social groups but may aggregate. But unlikely to be with consistently highly related individuals. “This species is subject to cylical chamges in population density (Hagstrum & Gilbert, 1976). After the ‘conolization period’ when density is low, there is a period of rapid population growth” (159) | 158, 159 | Polyandry | “Males as well as females mate with multiple partners” (158) | 158 | ? | ? | Non-kin | 60 | Rana sylvatica | 1982 | Waldman | Adaptive significance of communal oviposition in wood frogs (Rana sylvatica) | Beh Ecol & Sociobiol |
| Animalia | Annelida | Polychaeta | Canalipalpata | Serpulidae | Galeolaria | caespitosa | Galeolaria caespitosa | Juv | external fert, promiscuous, broadcast spawners (115) | 115 | Polyandry | “Similar to other studies on broadcast spawners, eggs enjoyed higher fertilisation success when they were fertilised under polyandrous conditions” (115) | 115 | NA | NA | Non-kin | 61 | Rana sylvatica | 2007 | Bee | Selective phonotaxis by male wood frogs (Rana sylvatica) to the sound of a chorus | Beh Ecol & Sociobiol |
| Animalia | Chordata | Mammalia | Rodentia | Cricetidae | Microtus | Microtus | Microtus montanus | Ad | As adults unlikely to associate with kin for long: “In contrast, montane voles (M. montanus) or meadow voles (M. pennsylvanicus) are much less social: males and females have separate territories and nests, and they only meet for mating. They are highly promiscuous and paren-tal care is provided by the female only. When montane vole pups are socially isolated, they do not emit distress vocalizations and their plasma corticosterone levels remain low.” (203) | 203 | Polyandry | Table 1 (80) & females mate with dominant & subordinate males in test situation (179) | 80, 179 | NA | NA | Non-kin | 62 | Apis mellifera | 2003 | Tarpy | Genetic diversity within honeybee colonies prevents severe infections and promotes colony growth | ProcB |
| Animalia | Chordata | Mammalia | Rodentia | Muroidae | Apodemus | flavicollis | Apodemus flavicollis | Ad | Not well known.“during the breeding period wood mice and yellow-necked mice are semi-social, and there are no stable associations between individuals. However, other authors have documented the existence of larger or smaller family groups” & “a tendency for association of kin in winter and spring was found also by analysis of the mtDNA (Rosakis 1999).” (11); Polyandry (6). May not live in groups generally, as most common group size is 1 from trapping study (195). | 6, 11, 195 | Polyandry | Table 2: 60% multiple sired litters (6) | 6 | Not philopatric: “Both species are known to disperse in autumn.”. | 196 | Non-kin | 63 | Rana temporaria | 1998 | Laurila | Multiple paternity in the common frog (Rana temporaria): genetic evidence from tadpole kin groups | Bio J of the Linnean Soc |
| Animalia | Chordata | Aves | Struthioniformes | Struthionidae | Struthio | camelus | Struthio camelus | Juv | chicks hatch out into group of mixed parentage “multiple paternity and maternity within a nest were common; 68.9% of all incubated eggs on a nest were not parented by both the resident territorial male and the major female of that nest” | 70 | Polyandry | “multiple paternity and maternity within a nest were common” (70) | 70 | NA | NA | Non-kin | 66 | Sciurus vulgaris | 1992 | Wauters | Spacing behaviour of red squirrels, Sciurus vulgaris: variation between habitats and the sexes | Animal Behaviour |
| Animalia | Chordata | Amphibia | Anura | Ranidae | Rana | temporaria | Rana temporaria | Juv | Multiple females lay in same pools: “a total of 121 females bred in 21 different rock-pools in 1994.” & Multiple paternity: “The results show that egg clutches of R. temporaria commonly contain multiply sired offspring.” (63). | 63 | Polyandry | “The results show that egg clutches of R. temporaria commonly contain multiply sired offspring.” (63) | 63 | NA | NA | Non-kin | 67 | Sciurus vulgaris | 1972 | Farentinos | SOCIAL DOMINANCE AND MATING ACTIVITY IN THE TASSEL-EARED SQUIRREL (SCIURUS ABERTI FERREUS) | Animal Behaviour |
| Animalia | Chordata | Amphibia | Anura | Ranidae | Lithobates | sylvatica | Rana sylvatica | Juv | Communal spawners so tadpoles hatch into mixed groups: “Males amplex females throughout breeding ponds, and pairs move toward a communal oviposition site. Females usually deposit eggs on submerged twigs or vegetation directly contiguous to other egg masses. Late” (60); “In “explosive breeders” (Wells 1977a), reproduction occurs over a short period (e.g., < 24 h) and commonly takes place in bodies of water that are often ephemeral or otherwise spatially and temporally unpredictable. In such species, the mating system is typically scramble competition polygyny" (61) | 60, 61 | Polyandry | “In such species, the mating system is typically scramble competition polygyny.” (61) | 61 | NA | NA | Non-kin | 70 | Struthio camelus | 2003 | Kimwele | A molecular genetic analysis of the communal nesting of the ostrich (Struthio camelus) | Mol. Ecol |
| Animalia | Chordata | Mammalia | Artiodactyla | Cervidae | Capreolus | capreolus | Capreolus capreolus | Juv | Group composition is variable, but often small family groups.“a local reproductive unit composed of family groups where the level of genetic relatedness is high and hence incestuous mating and associated elevated levels of inbreeding are potentially common” (36) | 36 | Polyandry | “From paternity analyses, we found that 13.5% of polytocous litters were sired by more than one male. We also found that a half-sib relationship was more likely than a full-sib relationship for 20.5% of all litters” (36) | 36 | “a large proportion of young of both sexes is philopatric, remaining on, or adjacent to, their mother’s range for the rest of their reproductive lives.” (36) | 36 | Kin | 76 | Microtus ochrogaster | 1993 | Getz | Social Organization of the Prairie Vole (Microtus ochrogaster) | J. Mamm |
| Animalia | Chordata | Amphibia | Caudata | Ambystomatidae | Ambystoma | opacum | Ambystoma opacum | Juv | Female lay egg masses under logs - females can lay together: “breeding sites may support numerous groups of siblings from different parents” (200)“; Polyandry”31–54%" multiple paternity. (23) | 200, 23 | Polyandry | “31–54%” multiple paternity. ~ 3 sires per clutch | 23 | NA | NA | Non-kin | 77 | Microtus ochrogaster | 2004 | Solomon | Multiple paternity in socially monogamous prairie voles ( Microtus ochrogaster ) | Canadian Journal of Zoology |
| Animalia | Arthropoda | Insecta | Hymenoptera | Apidae | Apis | mellifera | Apis mellifera | Ad | “honeybees (Apis mellifera L.), whose queens mate with an average of 12 males (Tarpy & Nielsen 2002), have among the highest levels of polyandry in all of the social insects (Strassmann 2001).” (62); “Apis mellifera, live in highly eusocial colonies that are each typically headed by a single queen” (186) | 62, 195 | Polyandry | “honeybees (Apis mellifera L.), whose queens mate with an average of 12 males (Tarpy & Nielsen 2002), have among the highest levels of polyandry in all of the social insects (Strassmann 2001)” (62) | 62 | NA (colonial) | NA (colonial) | Kin | 78 | Micvotus townsendii | 1991 | Lambin | Spatial organization and mating system of Microtus townsendii | Behavioral Ecology and Sociobiology |
| Animalia | Arthropoda | Insecta | Hymenoptera | Formicidae | Acromyrmex | echinatior | Acromyrmex echinatior | Ad | “All 15 A. echinatior colonies were headed by a single, multiply-mated queen, with absolute queen mating frequency (i.e., the number of males fathering offspring; Mp) being 6.07 (+/-0.51 SE; range 3–9), and genetically effective mating frequency (i.e., the number of males fathering offspring when adjusted for paternity skew; Me,p) being 3.88 (+/-0.27) (Table 1)” (17) | 17 | Polyandry | “All 15 A. echinatior colonies were headed by a single, multiply-mated queen, with absolute queen mating frequency (i.e., the number of males fathering offspring) being 6.07” (17) | 17 | NA (colonial) | NA (colonial) | Kin | 80 | Microtus montanus | 1990 | Heske | Sexual Dimorphism in Size, Relative Size of Testes, and Mating Systems in North AmericanVoles | Journal of Mammalogy, |
| Animalia | Chordata | Mammalia | Rodentia | Cricetidae | Lasiopodomys | brandtii | Lasiopodomys brandtii | Ad | “Brandt’s voles live in groups, each of which occupies a burrow system that” & “Estimates of mean group relatedness revealed that the majority of sampled Brandt’s vole groups were comprised of closely related individuals.” & “We suggest that extra-group polyandry is a reasonable explanation for the low incidence of inbreeding, because it may increase the frequency of mating among distantly-related individuals.” (87) | 87 | Polyandry | “Brandts vole females were extra-group polyandrous, and that the majority of pregnant females had mated with one or more males outside the group, along with one within-group male.” & “Molecular detection of multiple paternity revealed that Brandt’s vole females are polyandrous in natural populations” (87) | 87 | NA | NA | Kin | 87 | Lasiopodomys brandtii | 2013 | Liu | Inbreeding Avoidance Drives Consistent Variation of Fine-Scale Genetic Structure Caused by Dispersal in the Seasonal Mating System of Brandt ’ s Voles | Plos One |
| Animalia | Chordata | Mammalia | Rodentia | Cricetidae | Microtus | ochrogaster | Microtus ochrogaster | Ad | “Microtus ochrogaster lives in communal groups formed by retention of philopatric offspring and subsequent addition of unrelated adults” (76) | 76 | Polyandry | “found evidence of multiple paternity in five of nine litters (56%) analyzed.” (77) | 77 | NA | NA | Kin | 90 | Arabidopsis thaliana | 2010 | Platt | The scale of population structure in Arabidopsis thaliana | Plos Genetics |
| Animalia | Chordata | Mammalia | Rodentia | Muroidae | Rhabdomys | pumilio | Rhabdomys pumilio | Ad | “The striped mouse is group living with one single breeding male and up to 4 communally breeding females per group [20]. Groups typically contain several philopatric adult sons (and daughters) that are believed not to breed in their natal group” (181) | 181 | Polyandry | “Multiple paternity of litters was estimated as 36.0% (95% confi- dence interval: 16.0% - 56.0%)” 181 | 181 | Philopatry: “Groups typically contain several philopatric adult sons (and daughters)” (181) | 181 | Kin | 91 | Arabidopsis thaliana | 2003 | Hoffmann | Flower Visitors in a Natural Population of Arabidopsis thaliana | Plant Biology |
| Animalia | Chordata | Mammalia | Rodentia | Muroidae | Mus | muscorum | Mus musculus | Juv | “typically they occur in small polygynous breeding groups comprising a dominant male, a few females with their litters and sometimes some subordinate males (Crowcroft and Rowe, 1963; Reimer and Petras, 1967; Selander, 1970; Lidicker, 1976; Mackintosh, 1981).” (7); Juveniles raised in litters of full and half sibs" (201); Polyandry: “We found evidence of multiple paternity in 33 litters (23%).” (5); | 5, 7, 201 | Polyandry | “We found evidence of multiple paternity in 33 litters (23%).” (5) | 5 | NA | NA | Kin | 92 | Arabis holboellii | 2005 | Siemens | Tests for parasite-mediated frequency-dependent selection in natural populations of an asexual plant species | Evolutionary Ecology |
| Animalia | Annelida | Clitellata | Haplotaxida | Lumbricidae | Lumbricus | terrestris | Lumbricus terrestris | Ad | Although dispersal is limited, they have their own burrows, do not live with kin and don’t socially interact with conspecifics apart from to mate: “The vast majority of nocturnal surface visits, during which the tail remains securely anchored within the burrow, serves two main purposes: to forage for food and to search for mating partners. An intimate relationship between earthworm and burrow exists, burrows being an integral part of the life cycle that offer protection from predators and contain essential microhabitat and microbial communities (bacterial, fungal and chemical; Tiunov & Scheu, 1999). As a result, individuals are particularly devoted to their burrows, migrate rarely and are restricted to mating partners having an overlapping radius of surface activity.” (117) | 117 | Polyandry | “Lumbricus terrestris (or dew worm) is an obligatory out-crossing hermaphroditic earthworm” Sequential mating. (117) | 117 | “Natural dispersal of L. terrestris is reportedly quite low” (117) | 117 | Kin | 94 | Banksia hookeriana | 2005 | Barrett | Temporal patterns of genetic variation across a 9-year-old aerial seed bank of the shrub Banksia hookeriana (Proteaceae) | Mol Ecol |
| Animalia | Chordata | Amphibia | Anura | Rhacophoridae | Chiromantis | xerampelina | Chiromantis xerampelina | Juv | Extreme polyandry: “90% of females mating with 10 or more males during the deposition of a single clutch” (199); Tadpoles drop into waterbody and probably mix with tadpoles from other nests: “After oviposition, embryos develop rapidly within the nest, and within ~6 days, tadpoles break free from the foam and drop into the water below, where development is then completed within ~6 to 8 weeks”. Study looked at tadpoles after they dropped from foam nest. (199) | 199 | Polyandry | Extreme polandry: “90% of females mating with 10 or more males during the deposition of a single clutch” (199) | 199 | NA | NA | Non-kin | 95 | Banksia hookeriana | 2004 | He | Long-distance seed dispersal in a metapopulation of Banksia hookeriana inferred from a population allocation analysis of amplified fragment length polymorphism data | Mol Ecol |
| Animalia | Chordata | Amphibia | Caudata | Ambystomatidae | Ambystoma | talpoideum | Ambystoma talpoideum | Juv | Likely that communal spawning takes place: “breeding seasons typically are explosive (a few days) to brief (a few weeks) in duration (Salthe & Mecham, 1974). Large numbers of otherwise-terrestrial individuals gather at breeding sites, resulting in high densities” & “multiple paternity has been detected genetically within clutches” (188); | 188 | Polyandry | “multiple paternity has been detected genetically within clutches obtained from both field- and laboratory-mated A. talpoideum” (188) | 188 | NA | NA | Non-kin | 96 | Bugula neritina | 2012 | Aguirre | Genetic diversity increases population productivity in a sessile marine invertebrate | Ecology |
| Animalia | Chordata | Amphibia | Anura | Myobatrachidae | Crinia | georgiana | Crinia georgiana | Juv | “Polyandry occur 50% of matings” Multiple females lay in small pools where males congregate to R must range from 0.5 to 0 = medR¨; “In nature, embryos and tadpoles of C. georgiana develop in shallow, temporary pools” & “Females are attracted to aggregations of calling males”. Likely multiple females spawn in same area so tadpoles hatch out into mixed groups. 55 | 55 | Polyandry | “simultaneous polyandry occurs in approximately 50% of matings and results in multiple paternity” (55) | 55 | NA | NA | Non-kin | 97 | Bugula neritina | 1989 | Keough | Variation in Growth Rate and Reproduction of the Bryozoan Bugula neritina | The Biological Bulletin |
| Plantae | Angiosperms | Eudicots | Proteales | Proeaceae | Banksia | hookeriana | Banksia hookeriana | Ad | The study populations show high genetic differentiation even though they were 270-500m apart indicating related plants within populations (95); “B. hookeriana exists in at least part of its range as a meta- population in which individual populations on dune crests are geographically separated by uninhabitable intervening swales metres to kilometres in width” (94) | 94, 95 | Outcrossing | "B . hookeriana is self-compatible, producing seeds from self pollen, although seed set was higher for non-self pollen (94) | 94 | Limited as only about 7% of individuals originate form a different populatiln from the one in which they occur. | 94 | Kin | 98 | Bugula neritina | 1987 | Keough | Dispersal and Population Variation in the Bryozoan Bugula Neritina | Ecology |
| Plantae | Angiosperms | Eudicots | Asterales | Asteraceae | Cirsium | dissectum | Cirsium dissectum | Ad | “reproduces vegetatively via long rhizomes and typically forms dense patches within all habitat types.” (104); Clones in patches “Each patch generally contains more than one multilocus genotype suggesting that patches often contain more than one clone.” (104); “considerable clonal as well as sexual reproduction.” (103) | 103, 104 | Outcrossing | “It is self-compatible but selfed plants produce fewer seeds compared with those that are crossed” & “considerable clonal as well as sexual reproduction.” (103) | 103 | NA | NA | Kin | 99 | Ciona intestinalis | 2012 | Aguirre | DOES GENETIC DIVERSITY REDUCE SIBLING COMPETITION? | Evolution |
| Animalia | Arthropoda | Branchiopoda | Cladocera | Daphniidae | Daphnia | dentifera | Daphnia dentifera | Ad | “clones can quickly form populations of more than 10 000 individuals.” (106); “When a single clone founds a new population, selfing is unavoidable for the production of resting eggs to survive the winter. Even with less severe bottlenecks, inbreeding is expected to occur as a consequence of founder effects” (105) | 105, 106 | Selfing | “Daphnia species are cyclic parthenogens and under favourable environmental conditions reproduce by amictic parthenogenesis, they form all-female clones, which often become the dominant component of lakes and ponds zooplankton communities. Daphnia switch to sexual reproduction when conditions deteriorate and only then males are produced and sexually receptive females appear in the population” (105% | 105 | Not relevant as selfing | NA | Kin | 100 | Ciona intestinalis | 1991 | Kawamura | Self-Nonself Recognition Activity Extracted from Self-sterile Eggs of the Ascidian, Ciona intestinalis | Development Growth & Differentiation |
| Animalia | Arthropoda | Branchiopoda | Cladocera | Daphniidae | Daphnia | magna | Daphnia magna | Ad | “clones can quickly form populations of more than 10 000 individuals.” (106); “When a single clone founds a new population, selfing is unavoidable for the production of resting eggs to survive the winter. Even with less severe bottlenecks, inbreeding is expected to occur as a consequence of founder effects” (105) | 105, 106 | Selfing | “Daphnia species are cyclic parthenogens and under favourable environmental conditions reproduce by amictic parthenogenesis, they form all-female clones, which often become the dominant component of lakes and ponds zooplankton communities. Daphnia switch to sexual reproduction when conditions deteriorate and only then males are produced and sexually receptive females appear in the population” (105) | 105 | Not relevant as selfing | NA | Kin | 103 | Cirsium dissectum | 2009 | De Vere | Population size and habitat quality affect genetic diversity and fitness in the clonal herb Cirsium dissectum | Oecologia |
| Animalia | Mollusca | Gastropoda | Hygrophila | Lymnaeidae | Lymnaea | truncatula | Lymnaea truncatula | Ad | “L. truncatula is a preferential selfer, with a low genetic diversity within and high differentiation between populations” (121) Found in small water bodies, so probably high contact rates with related individuals “twenty-five snails/m2” (122) | 121, 122 | Selfing | “a self-compatible hermaphroditic freshwater snail.” & “Selfing rates were generally higher than 80%” (121) | 121 | Presumably stay within the contained water body. Low genetic diversity within populations suggests dispersal may be minimal (121) | 121 | Kin | 104 | Cirsium dissectum | 2007 | De Vere | Biological Flora of the British Isles: Cirsium dissectum (L.) Hill (Cirsium tuberosum (L.) All. subsp. anglicum (Lam.) Bonnier; Cnicus pratensis (Huds.) Willd., non Lam.; Cirsium anglicum (Lam.) DC.) | Journal\_of\_Ecology |
| Animalia | Mollusca | Gastropoda | Sorbeoconcha | Thiaridae | Melanoides | tuberculata | Melanoides tuberculata | Ad | “Most populations reproduce through obligate apomixis” (155); “Clonal diversity varied considerably among host populations, with some populations consisting of only two host genotypes, whereas in others every single host individual had a different genotype” (156); live in close proximity, mean of 18.5 snails per 25cm2 (range 0.2 to 107.3) so likely to interact with closely related individuals (156) | 155, 156 | Selfing | “both sexual and parthenogenetic individuals exist” & " Most populations reproduce through obligate apomixis" (155) | 155 | Found in clonal groups so long-distance dispersal unlikely | 156 | Kin | 105 | Daphnia magna | 2010 | Pietrzak | Longevity of Daphnia magna males and females | Hydrobiologia |
| Plantae | Angiosperms | Rosids | Brassicales | Brassicaeae | Arabidopsis | thaliana | Arabidopsis thaliana | Ad | selfing and occurs at high densities. “As a whole our sample selfed 97% of the time overall” & “Approximately 1/5th of sites are dominated by a single haplogroup (.80%).” (90). Can occur at high densities “The studied rather dense stand covers roughly 6m2 and comprises approximately 1500 plants” (91) | 90, 91 | Selfing | “Arabidopsis thaliana frequently reproduces by self-fertilizing and only occasionally outcrosses.” & “As a whole our sample selfed 97% of the time overall” (90) | 90 | NA | NA | Kin | 106 | Daphnia magna | 2002 | Haag | Strong inbreeding depression in a Daphnia metapopulation | Evolution |
| Animalia | Chordata | Actinopterygii | Cyprinodontiformes | Aplocheilidae | Kryptolebias | marmoratus | Kryptolebias marmoratus | Ad | Highly inbred: “Populations comprise of individuals that are homozygous across most, if not all, of their genome”; (9) “most individuals are simultaneous hermaphrodites that characteristically self-fertilize, resulting in local populations that consist of (nearly) homozygous lines.” (2). Likley to have close contact with related individuals: “While K. marmoratus is generally considered as solitary (Mackiewicz et al., 2006c), individuals are widely documented to seek refuge in high-density aggregations within fossorial habitats such as land crab burrows and emergent logs (Taylor, 1990, 2000; Taylor et al., 2008). These aggregations may be stable for up to a period of 9 months (Taylor, 2000).” (9) | 2, 9 | Selfing | “mixed-mating strategy of selfing plus occasional outcrossing” (1); “In this androdioecious species, most individuals are simultaneous hermaphrodites that characteristically self-fertilize, resulting in local populations that consist of (nearly) homozygous lines.” (2). | 1, 2 | May be some dispersal but still mostly clonal genotypes wthin populations: “Although mutation is the ultimate source of geneticdiversity in K. marmoratus , our data indicate that interlocality dispersal and outcross-mediated genetic recombination (and probably genetic drift also) play key proximate roles in the local ‘clonal’ dynamics of this species.” (202) | 202 | Kin | 109 | Gammarus duebeni | 2007 | Rock | Phylogeography and environmental diversification of a highly adaptable marine amphipod, Gammarus duebeni | Heredity |
| Plantae | Angiosperms | Rosids | Brassicales | Brassicaceae | Arabis | holboellii | Arabis holboellii | Ad | selfing: “Because seed production is apomictic, all progeny produced by an individual are genetically identical and, thus, genetic clones of the parent plant”. Grow in dense patches eight 25 m X 2 m transect per site and hundreds of individual plants sampled in each & low numbers of clones (see Table 2). Live with clones (92) | NA | Selfing | “all progeny produced by an individual are genetically identical and, thus, genetic clones of the parent plant” (92) | 92 | NA | NA | Kin | 112 | Gammarus duebeni | 2010 | Sutcliffe | Reproduction in Gammarus (Crustacea, Amphipoda): basic processes. | Freshwater Forum |
| Plantae | Angiosperms | Rosids | Myrtales | Ongaraceae | Oenothera | biennis | Oenothera biennis | Ad | “primarily self-pollinating species that forms discrete populations containing one to 14 genotypes” (134); Can be in dense patches “Plants typically grow in open habitats where they form discrete patches of one individual to several hundred individuals” (136) | 134, 136 | Selfing | “primarily self-pollinating species” | 134 | NA | NA | Kin | 113 | Gammarus duebeni | 2015 | Beermann | Social Recognition in Amphipods: An Overview | Book: Social recognition in invertebrates |
| Plantae | Angiosperms | Eudicots | Asterales | Asteraceae | Solidago | altissima | Solidago altissima | Ad | “Genotypic diversity in natural goldenrod patches can vary from 1 to 12 genotypes m?2 creating a natural mosaic of single-genotype and mixed-genotype patches of plants” (131) | 131 | Selfing | “reproduce vegetatively by rhizomes and sexually by seeds” | 132 | Not relevant as relatedness measured | NA | Kin | 114 | Gammarus duebeni | 2007 | Gamfeldt | Increasing intraspecific diversity increases predictability in population survival in the face of perturbations | Oikos |
| Bacteria | Proteobacteria | Gammaproteobacteria | Pseudomonadales | Pseudomonadaceae | Pseudomonas | aeruginosa | Pseudomonas aeruginosa | Ad | “Bacteria predominantly live in high-density communities, called biofilms” & “Clonal with horizontal gene trasfer”Horizontal gene transfer is a cause of genomic diversity in P. aeruginosa" (145) | 145 | Selfing | Clonal with horizontal gene trasfer “Horizontal gene transfer is a cause of genomic diversity in P. aeruginosa” (147) | 147 | Not relevant as selfing | NA | Kin | 115 | Galeolaria caespitosa | 2009 | McLeod | Do genetic diversity effects drive the benefits associated with multiple mating? A test in a marine invertebrate | PlosOne |
| Animalia | Mollusca | Gastropoda | Littorinimorpha | Thiaridae | Potamopyrgus | antipodarum | Potamopyrgus antipodarum | Ad | “Stream snail populations consisted of both sexual and asexual snails, as well as a wide range of clonal genotypes” (148); Live in aggregations with high contact rates “densities in infested waters typically range from 10,000/m2 to 40,000/m2” (149); Clonal lines common within populations “British populations consist of a small number of highly distinct geno-type groups with very few outlying genotypes, suggesting clonal lineages containing minor variation generated by mutation.”(185) | 148 | Selfing | “Individual snails are either triploid parthenogenetic females or diploid dioecious sexuals (Wallace 1992). Populations can be mixed, with both clones and sexual individuals, or strictly clonal” (148) | 148 | NA | NA | Kin | 117 | Lumbricus terrestris | 2007 | Field | Genetic diversity and parasite defense in a fragmented urban metapopulation of earthworms | Animal Conservation |
| Animalia | Bryozoa | Gymnolaemata | Cheilostomata | Bugulidae | Bugula | neritina | Bugula neritina | Ad | “Hermaphroditic, self-fertilization is possible (see Maturo [1991] for laboratory results), however, the prevalence and consequences of selfing in wild B. neritina populations are unknown.” & “After brooding, mothers release swimming, non-feeding larvae that typically settle and metamorphose within hours (Keough 1989, Marshall and Keough 2003), limiting the potential for long-distance dispersal” (96); “a single colony represents the genet.” & “The relatedness between larvae within a single colony is unclear; barring somatic mutation, the maternal zooids are genetically identical, but the number of paternal genotypes is unknown.” (97); “The spatial pattern is maintained by restricted dispersal” (98) | 96, 97, 98 | Selfing | hermaphroditic but rate of selfing unknown. “As B. neritina is hermaphroditic, self-fertilization is possible (see Maturo [1991] for laboratory results), however, the prevalence and consequences of selfing in wild B. neritina populations are unknown.” | 96 | The spatial pattern is maintained by restricted dispersal (98); “After brooding, mothers release swimming, non-feeding larvae that typically settle and metamorphose within hours (Keough 1989, Marshall and Keough 2003), limiting the potential for long-distance dispersal” (96) | 98, 96 | Kin | 121 | Lymnaea truncatula | 2003 | Trouvae | Evolutionary Implications of a High Selfing Rate in the Freshwater Snail Lymnaea Truncatula | Evolution |
| Plantae | Angiosperms | Monocots | Alismatales | Zosteraceae | Zostera | marina | Zostera marina | Ad | “Treatments reflect natural levels of Zostera genetic diversity in Bodega Bay, which range from 1 to 12 genotypes per m2, with a mean of 3.04 per m2” (126). Most identical genotypes occurred in clusters directly adjacent to one another, suggesting that they belong to the same genet.(127); “ramets having the same multilocus genotype were mainly adjacent” & “reproduce both sexually through seeds and vegetatively through horizontal rhizomes” (128) | 126, 127, 128 | Selfing | “reproduce both sexually through seeds and vegetatively through horizontal rhizomes” (128) | 128 | Most identical genotypes occurred in clusters directly (127) | 127 | Kin | 122 | Lymnaea truncatula | 1972 | Heppleston | Life History and Population Fluctuations of Lymnaea truncatula ( Mull ), the Snail Vector of Fascioliasis | J App Ecol. |
| Animalia | Mollusca | Gastropoda | Caenogastropoda | Batillariidae | Zeacumantus | subcarinatus | Zeacumantus subcarinatus | Ad | probably interact regularly with kin as limited disperal, low genetic diversity within populations & occur at high denisty: “Zeacumantus subcarinatus does not possess a planktonic larval stage and its eggs hatch into crawl-away larvae, i.e. miniature copies of the adult that are believed to remain within their site of origin” (154) & “widespread and in some places abundant gastropod” (151) Little gene-flow and high degree of pop gen structure: “Populations of the snail Zeacumantus subcarinatus displayed strong genetic differentiation and low levels of gene flow” (154) | 151, 153, 154 | Selfing | Possibly outcrossing "clumps of spawn small though sometimes the product of more than one parent (153) | 153 | probably live in closely related groups as limited disperal: “Zeacumantus subcarinatus does not possess a planktonic larval stage and its eggs hatch into crawl-away larvae, i.e. miniature copies of the adult that are believed to remain within their site of origin” & “Zeacumantus subcarinatus had a low number of effective female migrants in all comparisons” (154) | 154 | Kin | 126 | Zostera marina | 2004 | Hughes | Genetic diversity enhances the resistance of a seagrass ecosystem to disturbance | PNAS |
| Plantae | Angiosperms | Dicotyledonous | Malvales | Dipterocarpaceae | Shorea | acuminatissima | Shorea acuminatissima | Juv | 186. mean seedling relatedness of species 0.160 [i.e. half-sibs] | 186 | Selfing | mean seedling relatedness of species 0.160 [i.e. half-sibs] (186) | 186 | NA | NA | Kin | 127 | Zostera marina | 1999 | Meling-lopez | Annual life cycles of two Zostera marina L . populations in the Gulf of California contrasts in | Aquatic botany |
| Plantae | Angiosperms | Dicotyledonous | Malvales | Dipterocarpaceae | Shorea | argentifolia | Shorea argentifolia | Juv | 186. mean seedling relatedness of species 0.421 [i.e. nearly identical] | 186 | Selfing | mean seedling relatedness of species 0.421 [i.e. nearly identical] (186) | 186 | NA | NA | Kin | 128 | Zostera marina | 1999 | Reusch | Size and estimated age of genets in eelgrass, Zostera marina, assessed with microsatellite markers | Marine Biology |
| Plantae | Angiosperms | Dicotyledonous | Malvales | Dipterocarpaceae | Shorea | gibbosa | Shorea gibbosa | Juv | 186. mean seedling relatedness of species 0.122 [i.e. half-sib] | 186 | Selfing | mean seedling relatedness of species 0.122 [i.e. half-sib] (186) | 186 | NA | NA | Kin | 131 | Solidago altissima | 2008a | Crutsinger | Temporal dynamics in non-additive responses of arthropods to host-plant genotypic diversity | Oikos |
| Plantae | Angiosperms | Dicotyledonous | Malvales | Dipterocarpaceae | Parashorea | tomentella | Parashorea tomentella | Juv | 186. mean seedling relatedness of species 0.098 [i.e. half-sib] | 186 | Selfing | mean seedling relatedness of species 0.098 [i.e. half-sib] (196) | 186 | NA | NA | Kin | 132 | Solidago altissima | 2001 | Walck | Why Is Solidago shortii Narrowly Endemic and S . altissima Geographically Widespread ? A Comprehensive Comparative Study of Biological Traits Stable URL : https://www.jstor.org/stable/827604 vWhy is Solidago shortii narrowly endemic and S . altissima geographically widespread ? A comprehensive comparative study of biological traits | Journal of Biogeography |
| NA | NA | NA | NA | NA | NA | NA | NA | NA | NA | NA | NA | NA | NA | NA | NA | NA | 134 | Oenothera biennis | 2006 | Johnson | Additive and interactive effects of plant genotypic diversity on arthropod communities and plant fitness | Ecology\_Letters |
| NA | NA | NA | NA | NA | NA | NA | NA | NA | NA | NA | NA | NA | NA | NA | NA | NA | 136 | Oenothera biennis | 2007 | Johnson | Genotype-by-environment interactions leads to variable selection on life-history strategy in Common Evening Primrose ( Oenothera biennis ) | Journal\_of\_Evolutionary\_Biology |
| NA | NA | NA | NA | NA | NA | NA | NA | NA | NA | NA | NA | NA | NA | NA | NA | NA | 138 | Polistes humilis | 2009 | Hoggard | Mating system and genetic structure in the paper wasp (Polistes humilis) | Australian Journal of Zoology |
| NA | NA | NA | NA | NA | NA | NA | NA | NA | NA | NA | NA | NA | NA | NA | NA | NA | 140 | Vespula vulgaris | 2017 | Dobelmann | Fitness in invasive social wasps : the role of variation in viral load , immune response and paternity in predicting nest size and reproductive output | Oikos |
| NA | NA | NA | NA | NA | NA | NA | NA | NA | NA | NA | NA | NA | NA | NA | NA | NA | 144 | Myrmica rubra | 1996 | Sepp | Sociogenetic organization of the red ant Myrmica rubra | Behavioural Ecology & Sociobiology |
| NA | NA | NA | NA | NA | NA | NA | NA | NA | NA | NA | NA | NA | NA | NA | NA | NA | 145 | Pseudomonas aeruginosa | 2009a | Rice | The biofilm life cycle and virulence of Pseudomonas aeruginosa are dependent on a filamentous prophage | The ISME Journal |
| NA | NA | NA | NA | NA | NA | NA | NA | NA | NA | NA | NA | NA | NA | NA | NA | NA | 147 | Pseudomonas aeruginosa | 2004 | Head | Cross-Sectional Analysis of Clinical and Environmental Isolates of Pseudomonas aeruginosa : Biofilm Formation , Virulence , and Genome Diversity | Infection & Immunity |
| NA | NA | NA | NA | NA | NA | NA | NA | NA | NA | NA | NA | NA | NA | NA | NA | NA | 148 | Potamopyrgus antipodarum | 2011 | King | PARASITES , SEX , AND CLONAL DIVERSITY IN NATURAL SNAIL POPULATIONS. | Evolution |
| NA | NA | NA | NA | NA | NA | NA | NA | NA | NA | NA | NA | NA | NA | NA | NA | NA | 149 | Potamopyrgus antipodarum | 2013 | Goldberg | Environmental DNA as a new method for early detection of New Zealand mudsnails (Potamopyrgus antipodarum ) | Freshwater Science |
| NA | NA | NA | NA | NA | NA | NA | NA | NA | NA | NA | NA | NA | NA | NA | NA | NA | 151 | Zeacumantus subcarinatus | 2005 | Fredensborg | Impact of trematodes on host survival and population density in the intertidal gastropod Zeacumantus subcarinatus | Marine Progress Series |
| NA | NA | NA | NA | NA | NA | NA | NA | NA | NA | NA | NA | NA | NA | NA | NA | NA | 153 | Zeacumantus subcarinatus | 1941 | Graham | Breeding habits of twenty-two species of Marine Mollusca | Proceedings of the Royal Society New Zealand |
| NA | NA | NA | NA | NA | NA | NA | NA | NA | NA | NA | NA | NA | NA | NA | NA | NA | 154 | Zeacumantus subcarinatus | 2009 | Keeney | Contrasting mtDNA diversity and population structure in a direct-developing marine gastropod and its trematode parasites | Molecular Ecology |
| NA | NA | NA | NA | NA | NA | NA | NA | NA | NA | NA | NA | NA | NA | NA | NA | NA | 155 | Melanoides tuberculata | 2005 | Ben-ami | Spatial and temporal patterns of parthenogenesis and parasitism in the freshwater snail Melanoides tuberculata | J Ev Bio |
| NA | NA | NA | NA | NA | NA | NA | NA | NA | NA | NA | NA | NA | NA | NA | NA | NA | 156 | Melanoides tuberculata | 2013 | Dagan | Clonal diversity driven by parasitism in a freshwater snail | J Ev Bio |
| NA | NA | NA | NA | NA | NA | NA | NA | NA | NA | NA | NA | NA | NA | NA | NA | NA | 158 | Tribolium castaneum | 2007 | Pai | Polyandry and female control: the red flour beetle Tribolium castaneum as a case study | Journal of Experimental Zoology Part B |
| NA | NA | NA | NA | NA | NA | NA | NA | NA | NA | NA | NA | NA | NA | NA | NA | NA | 159 | Tribolium castaneum | 1999 | Arnaud | Mating Behaviour and Male Mate Choice in Tribolium castaneum (Coleoptera , Tenebrionidae) | Behavioural types and life history strategies during ontogeny in the mangrove killifish, Kryptolebias marmoratus |
| NA | NA | NA | NA | NA | NA | NA | NA | NA | NA | NA | NA | NA | NA | NA | NA | NA | 160 | Steinernema longicaudum | 2016 | Kapranas | Higher relatedness mitigates mortality in a nematode with lethal male fighting | Journal of Evolutionary Biology |
| NA | NA | NA | NA | NA | NA | NA | NA | NA | NA | NA | NA | NA | NA | NA | NA | NA | 161 | Steinernema longicaudum | 2014 | Zenner | Lethal Fighting in Nematodes Is Dependent on Developmental Pathway : Male-Male Fighting in the Entomopathogenic Nematode Steinernema longicaudum | PLosOne |
| NA | NA | NA | NA | NA | NA | NA | NA | NA | NA | NA | NA | NA | NA | NA | NA | NA | 163 | Steinernema longicaudum | 2012 | Griffin | Perspectives on the Behavior of Entomopathogenic Nematodes from Dispersal to Reproduction : Traits Contributing to Nematode Fitness and Biocontrol Efficacy | Journal of Nematodology |
| NA | NA | NA | NA | NA | NA | NA | NA | NA | NA | NA | NA | NA | NA | NA | NA | NA | 166 | Malacosoma californicum pluviale | 2012 | Franklin | Multiple Mating and Family Structure of the Western Tent Caterpillar , Malacosoma californicum pluviale : Impact on Disease Resistance | PLosOne |
| NA | NA | NA | NA | NA | NA | NA | NA | NA | NA | NA | NA | NA | NA | NA | NA | NA | 167 | Malacosoma californicum pluviale | 2011 | Myers | The effect of food limitation on immunity factors and disease resistance in the western tent caterpillar | Oecologia |
| NA | NA | NA | NA | NA | NA | NA | NA | NA | NA | NA | NA | NA | NA | NA | NA | NA | 171 | Maculinea nausithous | 2007 | Anton | Population structure of a large blue butterfly and its specialist parasitoid in a fragmented landscape | Molecular Ecology |
| NA | NA | NA | NA | NA | NA | NA | NA | NA | NA | NA | NA | NA | NA | NA | NA | NA | 173 | Maculinea nausithous | 2017 | Osvath-Ferencz | Population demography of the endangered large blue butterfly Maculinea arion in Europe | Journal of Insect Conservation |
| NA | NA | NA | NA | NA | NA | NA | NA | NA | NA | NA | NA | NA | NA | NA | NA | NA | 178 | Cardiocondyla obscurior | 2016 | Schmidt | Microsatellite markers for the tramp ant, Cardiocondyla obscurior (Formicidae: Myrmicinae) | Journal of Genetics |
| NA | NA | NA | NA | NA | NA | NA | NA | NA | NA | NA | NA | NA | NA | NA | NA | NA | 179 | Microtus montanus | 1986 | Shapiro | Male dominance, female choice and male copulatory behavior in two species of voles (Microtus ochrogaster and Microtus montanus) | Beh Ecol & Sociobiol |
| NA | NA | NA | NA | NA | NA | NA | NA | NA | NA | NA | NA | NA | NA | NA | NA | NA | 180 | Poecilia reticulata | 1999 | Kelly | Geographical variation in multiple paternity within natural populations of the guppy (Poecilia reticulata) | Proc B |
| NA | NA | NA | NA | NA | NA | NA | NA | NA | NA | NA | NA | NA | NA | NA | NA | NA | 181 | Rhabdomys pumilio | 2010 | Schradin | The nasty neighbour in the striped mouse (Rhabdomys pumilio) steals paternity and elicits aggression | Frontiers in Zoology |
| NA | NA | NA | NA | NA | NA | NA | NA | NA | NA | NA | NA | NA | NA | NA | NA | NA | 182 | Sciurus vulgaris | 1990 | Wauters | Factors affecting male mating success in red squirrels (Sciurus vulgaris) | Ethology Ecolofgy & Evolution |
| NA | NA | NA | NA | NA | NA | NA | NA | NA | NA | NA | NA | NA | NA | NA | NA | NA | 184 | Ciona intestinalis | 1993 | Svane | Spawning and Dispersal in Ciona intestinalis (L.) | Marine Ecology |
| NA | NA | NA | NA | NA | NA | NA | NA | NA | NA | NA | NA | NA | NA | NA | NA | NA | 186 | Shorea sp | 2020 | de Morais | Exploring the role of genetic diversity and relatedness in tree seedling growth and mortality: A multispecies study in a Bornean rainforest | Journal of Ecology |
| NA | NA | NA | NA | NA | NA | NA | NA | NA | NA | NA | NA | NA | NA | NA | NA | NA | 187 | Goniozus nephantidis | 2020 | Abdi | Co-foundress confinement elicits kinship effects in a naturally sub-social parasitoid | Journal of Evolutionary Biology |
| NA | NA | NA | NA | NA | NA | NA | NA | NA | NA | NA | NA | NA | NA | NA | NA | NA | 188 | Ambystoma talpoideum | 1998 | Verrell | Competition for Mates in the Mole Salamander , Ambystoma talpoideum : Tactics That May Maximize Male Mating Success | Behaviour |
| NA | NA | NA | NA | NA | NA | NA | NA | NA | NA | NA | NA | NA | NA | NA | NA | NA | 190 | Bombus lapidarius | 2010 | Lepais | Estimation of bumblebee queen dispersal distances using sibship reconstruction method | Molecular Ecology |
| NA | NA | NA | NA | NA | NA | NA | NA | NA | NA | NA | NA | NA | NA | NA | NA | NA | 191 | Monomorium pharaonis | 2011 | Schmidt | No benefit in diversity? The effect of genetic variation on survival and disease resistance in a polygynous social insect | Ecological Entomology |
| NA | NA | NA | NA | NA | NA | NA | NA | NA | NA | NA | NA | NA | NA | NA | NA | NA | 192 | Monomorium pharaonis | 1999 | Schmid-Hempel | Polyandry versus polygyny versus parasites | Philosophical Transactions of the Royal Society B: Biological Sciences |
| NA | NA | NA | NA | NA | NA | NA | NA | NA | NA | NA | NA | NA | NA | NA | NA | NA | 193 | Passer domesticus | 2009 | Liker | Genetic relatedness in wintering groups of house sparrows (Passer domesticus) | Molecular Ecology |
| NA | NA | NA | NA | NA | NA | NA | NA | NA | NA | NA | NA | NA | NA | NA | NA | NA | 194 | Sciurus vulgaris | 1994a | Wauters | Survival and lifetime reproductive success in dispersing and resident red squirrels | Behavioral Ecology and Sociobiology |
| NA | NA | NA | NA | NA | NA | NA | NA | NA | NA | NA | NA | NA | NA | NA | NA | NA | 195 | Apis mellifera | 2017 | Amiri | Queen Quality and the Impact of Honey Bee Diseases on Queen Health: Potential for Interactions between Two Major Threats to Colony Healt | Insects |
| NA | NA | NA | NA | NA | NA | NA | NA | NA | NA | NA | NA | NA | NA | NA | NA | NA | 196 | Apodemus flavicollis | 2009 | Kozakiewicz | The spatial genetic structure of bank vole (Myodes glareolus) and yellow-necked mouse (Apodemus flavicollis) populations: The effect of distance and habitat barriers | Animal Biology |
| NA | NA | NA | NA | NA | NA | NA | NA | NA | NA | NA | NA | NA | NA | NA | NA | NA | 199 | Chiromantis xerampelina | 2011 | Byrne | Effects of simultaneous polyandry on offspring fitness in an African tree frog | Behavioural Ecology |
| NA | NA | NA | NA | NA | NA | NA | NA | NA | NA | NA | NA | NA | NA | NA | NA | NA | 200 | Ambystoma opacum | 1994 | Walls | Does Kinship Influence Density Dependence in a Larval Salamander? | Oikos |
| NA | NA | NA | NA | NA | NA | NA | NA | NA | NA | NA | NA | NA | NA | NA | NA | NA | 201 | Mus musculus | 2016 | Thonhauser | Does multiple paternity influence offspring disease resistance? | Journal of Evolutionary Biology |
| NA | NA | NA | NA | NA | NA | NA | NA | NA | NA | NA | NA | NA | NA | NA | NA | NA | 202 | Kryptolebias marmoratus | 2007 | Tatarenkov | Strong population structure despite evidence of recent migration in a selfing hermaphroditic vertebrate, the mangrove killifish (Kryptolebias marmoratus) | Molecular Ecology |
|  |  |  |  |  |  |  |  |  |  |  |  |  |  |  |  |  | 203 | Microtus montanus | 2009 | Goymann | Pair-Bonding, Mating Systems and Hormones | Book: Encyclopedia of Animal Behavior |

## **Table S4:** Data used for analysis of the effect of within-group relatedness on variance in mortality and pathogen abundance (LnCVR).

| Study | Author | Year | Title | Journal | Species | Pathogen present | Pathogen manipulated | Relatedness manipulated | Measurement | Kin structure | Lab | Possible Inbreeding | Low r mean | High r mean | Low r SD | High r SD | Low r N | High r N | Ln CVR | Variance Ln CVR |
| --- | --- | --- | --- | --- | --- | --- | --- | --- | --- | --- | --- | --- | --- | --- | --- | --- | --- | --- | --- | --- |
| 2 | Johnson, MTJ; Lajeunesse, MJ; Agrawal, AA | 2006 | Additive and interactive effects of plant genotypic diversity on arthropod communities and plant fitness | Ecology Letters | Oenothera biennis | yes | obs | exp | Parasitism | Kin | no | no | 39.500 | 43.50 | 12.800 | 1.19700e+01 | 16.000 | 14.000 | -0.158 | 0.041 |
| 9 | McLeod, Laura; Marshall, Dustin J. | 2009 | Do genetic diversity effects drive the benefits associated with multiple mating? A test in a marine invertebrate | Plos One | Galeolaria caespitosa | no | obs | exp | Mortality | Non-kin | yes | no | 98.500 | 85.00 | 2.000 | 1.00000e+01 | 4.000 | 4.000 | 1.757 | 0.288 |
| 9 | McLeod, Laura; Marshall, Dustin J. | 2009 | Do genetic diversity effects drive the benefits associated with multiple mating? A test in a marine invertebrate | Plos One | Galeolaria caespitosa | no | obs | exp | Mortality | Non-kin | yes | no | 0.920 | 0.86 | 1.730 | 1.73000e+00 | 3.000 | 3.000 | 0.067 | 1.365 |
| 10 | Hughes, WOH; Boomsma, JJ | 2006 | Does genetic diversity hinder parasite evolution in social insect colonies? | Journal of Evolutionary Biology | Acromyrmex echinatior | yes | exp | exp | Parasitism | Kin | yes | no | 5.400 | 5.90 | 2.420 | 3.46000e+00 | 12.000 | 12.000 | 0.269 | 0.040 |
| 13 | WALLS, SC; BLAUSTEIN, AR | 1994 | Does Kinship Influence Density Dependence in a Larval Salamander? | Oikos | Ambystoma opacum | yes | obs | exp | Mortality | Non-kin | no | no | 0.420 | 0.27 | 0.250 | 9.00000e-02 | 5.000 | 5.000 | -0.580 | 0.144 |
| 13 | WALLS, SC; BLAUSTEIN, AR | 1994 | Does Kinship Influence Density Dependence in a Larval Salamander? | Oikos | Ambystoma opacum | yes | obs | exp | Mortality | Non-kin | no | no | 0.420 | 0.27 | 0.270 | 1.10000e-01 | 5.000 | 5.000 | -0.456 | 0.137 |
| 14 | Byrne, PG; Roberts, JD | 2000 | Does multiple paternity improve fitness of the frog Crinia georgiana? | Evolution | Crinia georgiana | no | obs | exp | Mortality | Non-kin | yes | no | 80.910 | 84.96 | 20.250 | 2.03400e+01 | 8.000 | 8.000 | -0.044 | 0.090 |
| 14 | Byrne, PG; Roberts, JD | 2000 | Does multiple paternity improve fitness of the frog Crinia georgiana? | Evolution | Crinia georgiana | no | obs | exp | Mortality | Non-kin | yes | no | 52.700 | 53.34 | 20.230 | 2.05900e+01 | 6.000 | 8.000 | -0.023 | 0.093 |
| 14 | Byrne, PG; Roberts, JD | 2000 | Does multiple paternity improve fitness of the frog Crinia georgiana? | Evolution | Crinia georgiana | no | obs | exp | Mortality | Non-kin | yes | no | 28.700 | 42.17 | 22.800 | 2.25400e+01 | 6.000 | 7.000 | -0.413 | 0.110 |
| 14 | Byrne, PG; Roberts, JD | 2000 | Does multiple paternity improve fitness of the frog Crinia georgiana? | Evolution | Crinia georgiana | no | obs | exp | Mortality | Non-kin | yes | no | 40.800 | 28.08 | 22.410 | 2.24100e+01 | 5.000 | 6.000 | 0.349 | 0.107 |
| 15 | Thonhauser, KE; Raveh, S; Thoss, M; Penn, DJ | 2016 | Does multiple paternity influence offspring disease resistance? | Journal of Evolutionary Biology | Mus musculus | yes | exp | exp | Parasitism | Kin | yes | no | 81.451 | 70.11 | 1.160 | 1.12000e+00 | 15.000 | 15.000 | 0.115 | 0.069 |
| 16 | Byrne, PG; Whiting, MJ | 2011 | Effects of simultaneous Polyandrous on offspring fitness in an African tree frog | Behavioral Ecology | Chiromantis xerampelina | yes | obs | obs | Mortality | Non-kin | no | no | 56.000 | 34.00 | 9.490 | 2.21400e+01 | 10.000 | 10.000 | 1.346 | 0.053 |
| 20 | Baer, B; Schmid-Hempel, P | 1999 | Experimental variation in Polyandrous affects parasite loads and fitness in a bumble-bee | Nature | Bombus terrestris | yes | obs | exp | Parasitism | Kin | no | no | 0.650 | 0.90 | 0.350 | 2.10000e-01 | 12.000 | 7.000 | -0.798 | 0.078 |
| 20 | Baer, B; Schmid-Hempel, P | 1999 | Experimental variation in Polyandrous affects parasite loads and fitness in a bumble-bee | Nature | Bombus terrestris | yes | obs | exp | Parasitism | Kin | no | no | 1.600 | 2.50 | 1.040 | 6.60000e-01 | 12.000 | 7.000 | -0.863 | 0.077 |
| 20 | Baer, B; Schmid-Hempel, P | 1999 | Experimental variation in Polyandrous affects parasite loads and fitness in a bumble-bee | Nature | Bombus terrestris | yes | obs | exp | Parasitism | Kin | no | no | 0.020 | 0.13 | 0.050 | 1.01000e-01 | 12.000 | 7.000 | -1.135 | 0.412 |
| 20 | Baer, B; Schmid-Hempel, P | 1999 | Experimental variation in Polyandrous affects parasite loads and fitness in a bumble-bee | Nature | Bombus terrestris | yes | obs | exp | Parasitism | Kin | no | no | 0.550 | 0.68 | 0.350 | 2.10000e-01 | 12.000 | 7.000 | -0.685 | 0.071 |
| 20 | Baer, B; Schmid-Hempel, P | 1999 | Experimental variation in Polyandrous affects parasite loads and fitness in a bumble-bee | Nature | Bombus terrestris | yes | obs | exp | Parasitism | Kin | no | no | 1.100 | 2.40 | 0.350 | 1.32000e+00 | 12.000 | 7.000 | 0.585 | 0.048 |
| 20 | Baer, B; Schmid-Hempel, P | 1999 | Experimental variation in Polyandrous affects parasite loads and fitness in a bumble-bee | Nature | Bombus terrestris | yes | obs | exp | Parasitism | Kin | no | no | 0.010 | 1.40 | 0.030 | 9.30000e-01 | 12.000 | 7.000 | -1.470 | 0.607 |
| 21 | Reber, A; Castella, G; Christe, P; Chapuisat, M | 2008 | Experimentally increased group diversity improves disease resistance in an ant species | Ecology Letters | Formica selysi | no | exp | exp | Mortality | Kin | yes | no | 84.000 | 88.00 | 7.600 | 8.00000e+00 | 16.000 | 64.000 | -0.021 | 0.036 |
| 21 | Reber, A; Castella, G; Christe, P; Chapuisat, M | 2008 | Experimentally increased group diversity improves disease resistance in an ant species | Ecology Letters | Formica selysi | yes | exp | obs | Mortality | Kin | yes | no | 59.500 | 69.00 | 26.460 | 2.59800e+01 | 28.000 | 27.000 | -0.166 | 0.019 |
| 21 | Reber, A; Castella, G; Christe, P; Chapuisat, M | 2008 | Experimentally increased group diversity improves disease resistance in an ant species | Ecology Letters | Formica selysi | yes | exp | exp | Mortality | Kin | yes | no | 62.000 | 51.00 | 19.200 | 3.84000e+01 | 16.000 | 64.000 | 0.863 | 0.025 |
| 21 | Reber, A; Castella, G; Christe, P; Chapuisat, M | 2008 | Experimentally increased group diversity improves disease resistance in an ant species | Ecology Letters | Formica selysi | no | exp | obs | Mortality | Kin | yes | no | 85.500 | 88.00 | 4.230 | 5.20000e+00 | 28.000 | 27.000 | 0.178 | 0.034 |
| 25 | Hughes, WOH; Boomsma, JJ | 2004 | Genetic diversity and disease resistance in leaf-cutting ant societies | Evolution | Acromyrmex echinatior | no | exp | exp | Mortality | Kin | yes | no | 2.900 | 2.95 | 0.720 | 7.20000e-01 | 80.000 | 80.000 | -0.017 | 0.008 |
| 25 | Hughes, WOH; Boomsma, JJ | 2004 | Genetic diversity and disease resistance in leaf-cutting ant societies | Evolution | Acromyrmex echinatior | yes | exp | exp | Mortality | Kin | yes | no | 0.850 | 0.90 | 0.720 | 7.20000e-01 | 80.000 | 80.000 | -0.057 | 0.008 |
| 25 | Hughes, WOH; Boomsma, JJ | 2004 | Genetic diversity and disease resistance in leaf-cutting ant societies | Evolution | Acromyrmex echinatior | yes | exp | exp | Mortality | Kin | yes | no | 2.000 | 1.50 | 0.890 | 8.90000e-01 | 80.000 | 80.000 | 0.288 | 0.005 |
| 25 | Hughes, WOH; Boomsma, JJ | 2004 | Genetic diversity and disease resistance in leaf-cutting ant societies | Evolution | Acromyrmex echinatior | no | exp | exp | Mortality | Kin | yes | no | 2.800 | 2.80 | 0.890 | 8.90000e-01 | 80.000 | 80.000 | 0.000 | 0.007 |
| 27 | Hughes, AR; Stachowicz, JJ | 2004 | Genetic diversity enhances the resistance of a seagrass ecosystem to disturbance | Proceedings of the National Academy of Sciences | Zostera marina | yes | obs | exp | Mortality | Kin | no | no | 64.000 | 47.50 | 24.000 | 8.57000e+00 | 9.000 | 6.000 | -0.694 | 0.107 |
| 28 | Aguirre, JD; Marshall, DJ | 2012 | Genetic diversity increases population productivity in a sessile marine invertebrate | Ecology | Bugula neritina | yes | obs | exp | Mortality | Kin | no | no | 0.680 | 0.52 | 0.210 | 2.10000e-01 | 9.000 | 9.000 | 0.268 | 0.064 |
| 29 | Altermatt, F; Ebert, D | 2008 | Genetic diversity of Daphnia magna populations enhances resistance to parasites | Ecology Letters | Daphnia magna | yes | exp | exp | Parasitism | Kin | yes | no | 0.400 | 0.74 | 0.190 | 3.10000e-01 | 15.000 | 15.000 | -0.126 | 0.035 |
| 29 | Altermatt, F; Ebert, D | 2008 | Genetic diversity of Daphnia magna populations enhances resistance to parasites | Ecology Letters | Daphnia magna | yes | exp | exp | Parasitism | Kin | yes | no | 0.020 | 0.06 | 0.080 | 1.90000e-01 | 15.000 | 15.000 | -0.234 | 1.309 |
| 29 | Altermatt, F; Ebert, D | 2008 | Genetic diversity of Daphnia magna populations enhances resistance to parasites | Ecology Letters | Daphnia magna | yes | exp | exp | Parasitism | Kin | yes | no | 0.950 | 0.99 | 0.040 | 4.00000e-02 | 15.000 | 15.000 | -0.041 | 0.066 |
| 29 | Altermatt, F; Ebert, D | 2008 | Genetic diversity of Daphnia magna populations enhances resistance to parasites | Ecology Letters | Daphnia magna | yes | exp | exp | Parasitism | Kin | yes | no | 0.770 | 1.00 | 0.460 | 4.00000e-02 | 15.000 | 15.000 | -2.704 | 0.060 |
| 29 | Altermatt, F; Ebert, D | 2008 | Genetic diversity of Daphnia magna populations enhances resistance to parasites | Ecology Letters | Daphnia magna | yes | exp | exp | Parasitism | Kin | yes | no | 0.390 | 0.77 | 0.580 | 1.90000e-01 | 15.000 | 15.000 | -1.796 | 0.122 |
| 29 | Altermatt, F; Ebert, D | 2008 | Genetic diversity of Daphnia magna populations enhances resistance to parasites | Ecology Letters | Daphnia magna | yes | exp | exp | Parasitism | Kin | yes | no | 0.960 | 1.00 | 0.120 | 4.00000e-02 | 15.000 | 15.000 | -1.139 | 0.062 |
| 29 | Altermatt, F; Ebert, D | 2008 | Genetic diversity of Daphnia magna populations enhances resistance to parasites | Ecology Letters | Daphnia magna | yes | exp | exp | Parasitism | Kin | yes | no | 0.190 | 0.28 | 0.270 | 3.10000e-01 | 15.000 | 15.000 | -0.250 | 0.113 |
| 29 | Altermatt, F; Ebert, D | 2008 | Genetic diversity of Daphnia magna populations enhances resistance to parasites | Ecology Letters | Daphnia magna | yes | exp | exp | Parasitism | Kin | yes | no | 0.650 | 0.70 | 0.230 | 2.70000e-01 | 15.000 | 15.000 | 0.086 | 0.036 |
| 29 | Altermatt, F; Ebert, D | 2008 | Genetic diversity of Daphnia magna populations enhances resistance to parasites | Ecology Letters | Daphnia magna | yes | exp | exp | Parasitism | Kin | yes | no | 0.050 | 0.08 | 0.010 | 1.90000e-01 | 15.000 | 15.000 | 2.474 | 0.225 |
| 29 | Altermatt, F; Ebert, D | 2008 | Genetic diversity of Daphnia magna populations enhances resistance to parasites | Ecology Letters | Daphnia magna | yes | exp | exp | Parasitism | Kin | yes | no | 0.840 | 0.99 | 0.390 | 4.00000e-02 | 15.000 | 15.000 | -2.442 | 0.058 |
| 29 | Altermatt, F; Ebert, D | 2008 | Genetic diversity of Daphnia magna populations enhances resistance to parasites | Ecology Letters | Daphnia magna | yes | exp | exp | Parasitism | Kin | yes | no | 0.640 | 0.68 | 0.310 | 3.10000e-01 | 15.000 | 15.000 | -0.061 | 0.034 |
| 29 | Altermatt, F; Ebert, D | 2008 | Genetic diversity of Daphnia magna populations enhances resistance to parasites | Ecology Letters | Daphnia magna | yes | exp | exp | Parasitism | Kin | yes | no | 0.160 | 0.33 | 0.120 | 2.30000e-01 | 15.000 | 15.000 | -0.073 | 0.039 |
| 29 | Altermatt, F; Ebert, D | 2008 | Genetic diversity of Daphnia magna populations enhances resistance to parasites | Ecology Letters | Daphnia magna | yes | exp | exp | Parasitism | Kin | yes | no | 0.750 | 0.60 | 0.310 | 3.50000e-01 | 15.000 | 15.000 | 0.345 | 0.031 |
| 29 | Altermatt, F; Ebert, D | 2008 | Genetic diversity of Daphnia magna populations enhances resistance to parasites | Ecology Letters | Daphnia magna | no | exp | exp | Mortality | Kin | yes | no | 290.000 | 450.00 | 232.380 | 5.80950e+02 | 15.000 | 15.000 | 0.477 | 0.066 |
| 29 | Altermatt, F; Ebert, D | 2008 | Genetic diversity of Daphnia magna populations enhances resistance to parasites | Ecology Letters | Daphnia magna | yes | exp | exp | Parasitism | Kin | yes | no | 0.270 | 0.63 | 0.270 | 2.30000e-01 | 15.000 | 15.000 | -1.008 | 0.061 |
| 29 | Altermatt, F; Ebert, D | 2008 | Genetic diversity of Daphnia magna populations enhances resistance to parasites | Ecology Letters | Daphnia magna | yes | exp | exp | Mortality | Kin | yes | no | 325.000 | 225.00 | 96.820 | 3.87300e+02 | 15.000 | 15.000 | 1.754 | 0.104 |
| 29 | Altermatt, F; Ebert, D | 2008 | Genetic diversity of Daphnia magna populations enhances resistance to parasites | Ecology Letters | Daphnia magna | yes | exp | exp | Mortality | Kin | yes | no | 625.000 | 310.00 | 154.920 | 3.48570e+02 | 15.000 | 15.000 | 1.512 | 0.045 |
| 30 | Desai, SD; Currie, RW | 2015 | Genetic diversity within honey bee colonies affects pathogen load and relative virus levels in honey bees, Apis mellifera L | Behav Ecol and Sociobiol | Apis mellifera | yes | obs | exp | Parasitism | Kin | no | no | 0.434 | 1.00 | 0.810 | 8.10000e-01 | 24.000 | 11.000 | -0.806 | 0.114 |
| 30 | Desai, SD; Currie, RW | 2015 | Genetic diversity within honey bee colonies affects pathogen load and relative virus levels in honey bees, Apis mellifera L | Behav Ecol and Sociobiol | Apis mellifera | yes | obs | exp | Parasitism | Kin | no | no | 0.925 | 0.52 | 0.550 | 5.50000e-01 | 24.000 | 11.000 | 0.605 | 0.037 |
| 30 | Desai, SD; Currie, RW | 2015 | Genetic diversity within honey bee colonies affects pathogen load and relative virus levels in honey bees, Apis mellifera L | Behav Ecol and Sociobiol | Apis mellifera | yes | obs | exp | Parasitism | Kin | no | no | 100.000 | 60.00 | 195.960 | 1.65830e+02 | 24.000 | 11.000 | 0.372 | 0.518 |
| 30 | Desai, SD; Currie, RW | 2015 | Genetic diversity within honey bee colonies affects pathogen load and relative virus levels in honey bees, Apis mellifera L | Behav Ecol and Sociobiol | Apis mellifera | yes | obs | exp | Parasitism | Kin | no | no | 320.000 | 90.00 | 195.960 | 1.99000e+02 | 24.000 | 11.000 | 1.312 | 0.236 |
| 30 | Desai, SD; Currie, RW | 2015 | Genetic diversity within honey bee colonies affects pathogen load and relative virus levels in honey bees, Apis mellifera L | Behav Ecol and Sociobiol | Apis mellifera | yes | obs | exp | Parasitism | Kin | no | no | 0.035 | 0.19 | 0.330 | 3.30000e-01 | 24.000 | 11.000 | -1.675 | 3.610 |
| 30 | Desai, SD; Currie, RW | 2015 | Genetic diversity within honey bee colonies affects pathogen load and relative virus levels in honey bees, Apis mellifera L | Behav Ecol and Sociobiol | Apis mellifera | yes | obs | exp | Parasitism | Kin | no | no | 0.366 | 0.55 | 0.550 | 5.50000e-01 | 24.000 | 11.000 | -0.378 | 0.083 |
| 30 | Desai, SD; Currie, RW | 2015 | Genetic diversity within honey bee colonies affects pathogen load and relative virus levels in honey bees, Apis mellifera L | Behav Ecol and Sociobiol | Apis mellifera | yes | obs | exp | Parasitism | Kin | no | no | 0.113 | 0.16 | 0.330 | 3.30000e-01 | 24.000 | 11.000 | -0.320 | 0.461 |
| 30 | Desai, SD; Currie, RW | 2015 | Genetic diversity within honey bee colonies affects pathogen load and relative virus levels in honey bees, Apis mellifera L | Behav Ecol and Sociobiol | Apis mellifera | yes | obs | exp | Parasitism | Kin | no | no | 0.837 | 0.96 | 0.810 | 8.10000e-01 | 24.000 | 11.000 | -0.109 | 0.039 |
| 31 | Tarpy, DR | 2003 | Genetic diversity within honeybee colonies prevents severe infections and promotes colony growth | Proc. R. Soc. Lond. B. | Apis mellifera | yes | obs | exp | Parasitism | Kin | no | no | 5.474 | 3.49 | 1.280 | 3.00000e+00 | 24.000 | 24.000 | 1.302 | 0.021 |
| 32 | SHYKOFF, JA; SCHMIDHEMPEL, P | 1991 | Genetic relatedness and eusociality parasite mediated selection on the genetic composition of groups | Behavioral Ecology and Sociobiology | Bombus terrestris | yes | exp | exp | Parasitism | Kin | yes | no | 0.671 | 0.91 | 0.490 | 3.70000e-01 | 15.000 | 15.000 | -0.586 | 0.043 |
| 34 | Liersch, and Schmid-Hempel | 1998 | Genetic variation within social insect colonies reduces parasite load | Proc. R. Soc. Lond. B. | Bombus terrestris | yes | obs | exp | Parasitism | Kin | no | no | 0.270 | 0.52 | 0.130 | 2.20000e-01 | 10.000 | 7.000 | -0.102 | 0.063 |
| 34 | Liersch, and Schmid-Hempel | 1998 | Genetic variation within social insect colonies reduces parasite load | Proc. R. Soc. Lond. B. | Bombus terrestris | yes | obs | exp | Parasitism | Kin | no | no | 0.312 | 0.58 | 0.220 | 2.90000e-01 | 10.000 | 7.000 | -0.316 | 0.066 |
| 34 | Liersch, and Schmid-Hempel | 1998 | Genetic variation within social insect colonies reduces parasite load | Proc. R. Soc. Lond. B. | Bombus terrestris | yes | obs | exp | Parasitism | Kin | no | no | 1.500 | 2.43 | 0.540 | 1.14000e+00 | 10.000 | 7.000 | 0.293 | 0.060 |
| 38 | Kapranas, A; Maher, AMD; Griffin, CT | 2016 | Higher relatedness mitigates mortality in a nematode with lethal male fighting | JOURNAL OF EVOLUTIONARY BIOLOGY | Steinernema longicaudum | no | obs | exp | Mortality | Kin | yes | no | 0.840 | 0.68 | 0.530 | 6.20000e-01 | 18.000 | 18.000 | 0.368 | 0.031 |
| 38 | Kapranas, A; Maher, AMD; Griffin, CT | 2016 | Higher relatedness mitigates mortality in a nematode with lethal male fighting | JOURNAL OF EVOLUTIONARY BIOLOGY | Steinernema longicaudum | no | obs | exp | Mortality | Kin | yes | no | 0.700 | 0.53 | 0.550 | 5.90000e-01 | 18.000 | 18.000 | 0.348 | 0.044 |
| 39 | Gardner, MG; Schonrogge, K; Elmes, GW; Thomas, JA | 2007 | Increased genetic diversity as a defence against parasites is undermined by social parasites: Microdon mutabilis hoverflies infesting Formica lemani ant colonies | Proc. R. Soc. Lond. B. | Formica lemani | yes | obs | obs | Parasitism | Kin | no | no | 0.170 | 0.33 | 0.070 | 9.00000e-02 | 22.000 | 19.000 | -0.408 | 0.029 |
| 39 | Gardner, MG; Schonrogge, K; Elmes, GW; Thomas, JA | 2007 | Increased genetic diversity as a defence against parasites is undermined by social parasites: Microdon mutabilis hoverflies infesting Formica lemani ant colonies | Proc. R. Soc. Lond. B. | Formica lemani | yes | obs | obs | Parasitism | Kin | no | no | 0.458 | 0.57 | 0.080 | 8.00000e-02 | 20.000 | 10.000 | -0.190 | 0.059 |
| 40 | Gamfeldt, L; Kallstrom, B | 2007 | Increasing intraspecific diversity increases predictability in population survival in the face of perturbations | Oikos | Gammarus duebeni | no | obs | exp | Mortality | Non-kin | yes | no | 5.000 | 5.20 | 2.680 | 4.47000e+00 | 5.000 | 5.000 | 0.472 | 0.112 |
| 40 | Gamfeldt, L; Kallstrom, B | 2007 | Increasing intraspecific diversity increases predictability in population survival in the face of perturbations | Oikos | Gammarus duebeni | no | obs | exp | Mortality | Non-kin | yes | no | 3.200 | 5.20 | 2.240 | 3.58000e+00 | 5.000 | 5.000 | -0.017 | 0.122 |
| 41 | LAMBIN, X; KREBS, CJ | 1993 | Influence of Female Relatedness on the Demography of Townsend’s Vole Populations in Spring | Brittish Ecological Society | Microtus townsendii | yes | obs | exp | Mortality | Kin | no | no | 93.000 | 95.00 | 13.780 | 7.48000e+00 | 11.000 | 51.000 | -0.672 | 0.049 |
| 43 | Tarpy, DR; Seeley, TD | 2006 | Lower disease infections in honeybee (Apis mellifera) colonies headed by polyandrous vs monandrous queens | Naturwissenschaften | Apis mellifera | yes | obs | exp | Parasitism | Kin | no | no | 6.500 | 12.00 | 4.077 | 5.09600e+00 | 10.000 | 10.000 | -0.390 | 0.059 |
| 46 | Schmidt, AM; Linksvayer, TA; Boomsma, JJ; Pedersen, JS | 2011 | No benefit in diversity? The effect of genetic variation on survival and disease resistance in a polygynous social insect | Ecological Entomology | Monomorium pharaonis | yes | exp | exp | Mortality | Kin | yes | no | 1.000 | 6.60 | 0.790 | 3.15000e+00 | 4.000 | 12.000 | -0.625 | 0.158 |
| 46 | Schmidt, AM; Linksvayer, TA; Boomsma, JJ; Pedersen, JS | 2011 | No benefit in diversity? The effect of genetic variation on survival and disease resistance in a polygynous social insect | Ecological Entomology | Monomorium pharaonis | yes | exp | exp | Mortality | Kin | yes | no | 0.820 | 0.90 | 0.140 | 1.10000e+00 | 4.000 | 12.000 | 1.847 | 0.167 |
| 46 | Schmidt, AM; Linksvayer, TA; Boomsma, JJ; Pedersen, JS | 2011 | No benefit in diversity? The effect of genetic variation on survival and disease resistance in a polygynous social insect | Ecological Entomology | Monomorium pharaonis | yes | exp | exp | Mortality | Kin | yes | no | 0.910 | 0.82 | 0.080 | 1.00000e-01 | 5.667 | 18.000 | 0.250 | 0.117 |
| 46 | Schmidt, AM; Linksvayer, TA; Boomsma, JJ; Pedersen, JS | 2011 | No benefit in diversity? The effect of genetic variation on survival and disease resistance in a polygynous social insect | Ecological Entomology | Monomorium pharaonis | no | exp | exp | Mortality | Kin | yes | no | 0.910 | 0.98 | 0.080 | 2.00000e-02 | 5.667 | 9.000 | -1.505 | 0.155 |
| 46 | Schmidt, AM; Linksvayer, TA; Boomsma, JJ; Pedersen, JS | 2011 | No benefit in diversity? The effect of genetic variation on survival and disease resistance in a polygynous social insect | Ecological Entomology | Monomorium pharaonis | yes | exp | exp | Mortality | Kin | yes | no | 0.820 | 0.89 | 0.140 | 8.00000e-02 | 4.000 | 16.000 | -0.775 | 0.162 |
| 46 | Schmidt, AM; Linksvayer, TA; Boomsma, JJ; Pedersen, JS | 2011 | No benefit in diversity? The effect of genetic variation on survival and disease resistance in a polygynous social insect | Ecological Entomology | Monomorium pharaonis | no | exp | exp | Mortality | Kin | yes | no | 0.910 | 0.92 | 0.080 | 8.00000e-02 | 5.667 | 18.000 | -0.089 | 0.119 |
| 46 | Schmidt, AM; Linksvayer, TA; Boomsma, JJ; Pedersen, JS | 2011 | No benefit in diversity? The effect of genetic variation on survival and disease resistance in a polygynous social insect | Ecological Entomology | Monomorium pharaonis | yes | exp | exp | Mortality | Kin | yes | no | 1.000 | 6.00 | 0.790 | 2.83000e+00 | 4.000 | 11.000 | -0.632 | 0.159 |
| 46 | Schmidt, AM; Linksvayer, TA; Boomsma, JJ; Pedersen, JS | 2011 | No benefit in diversity? The effect of genetic variation on survival and disease resistance in a polygynous social insect | Ecological Entomology | Monomorium pharaonis | no | exp | exp | Mortality | Kin | yes | no | 1.000 | 6.80 | 0.970 | 2.34000e+00 | 5.667 | 9.000 | -1.081 | 0.151 |
| 46 | Schmidt, AM; Linksvayer, TA; Boomsma, JJ; Pedersen, JS | 2011 | No benefit in diversity? The effect of genetic variation on survival and disease resistance in a polygynous social insect | Ecological Entomology | Monomorium pharaonis | no | exp | exp | Mortality | Kin | yes | no | 0.820 | 0.78 | 0.140 | 1.70000e-01 | 4.000 | 11.000 | 0.127 | 0.163 |
| 46 | Schmidt, AM; Linksvayer, TA; Boomsma, JJ; Pedersen, JS | 2011 | No benefit in diversity? The effect of genetic variation on survival and disease resistance in a polygynous social insect | Ecological Entomology | Monomorium pharaonis | no | exp | exp | Mortality | Kin | yes | no | 1.000 | 6.40 | 0.790 | 2.06000e+00 | 4.000 | 16.000 | -1.031 | 0.160 |
| 46 | Schmidt, AM; Linksvayer, TA; Boomsma, JJ; Pedersen, JS | 2011 | No benefit in diversity? The effect of genetic variation on survival and disease resistance in a polygynous social insect | Ecological Entomology | Monomorium pharaonis | no | exp | exp | Mortality | Kin | yes | no | 1.000 | 5.50 | 0.970 | 3.02000e+00 | 5.667 | 18.000 | -0.647 | 0.134 |
| 46 | Schmidt, AM; Linksvayer, TA; Boomsma, JJ; Pedersen, JS | 2011 | No benefit in diversity? The effect of genetic variation on survival and disease resistance in a polygynous social insect | Ecological Entomology | Monomorium pharaonis | yes | exp | exp | Mortality | Kin | yes | no | 1.000 | 9.50 | 0.970 | 2.01000e+00 | 5.667 | 18.000 | -1.600 | 0.145 |
| 55 | Seeley, TD; Tarpy, DR | 2007 | Queen promiscuity lowers disease within honeybee colonies | Proc. R. Soc. Lond. B. | Apis mellifera | yes | exp | exp | Parasitism | Kin | no | no | 5.000 | 40.00 | 4.630 | 3.24300e+01 | 14.000 | 14.000 | -0.133 | 0.054 |
| 62 | van Houte, S; Ekroth, AKE; Broniewski, JM; Chabas, H; Ashby, B; Bondy-Denomy, J; Gandon, S; Boots, M; Paterson, S; Buckling, A; Westra, ER | 2016 | The diversity-generating benefits of a prokaryotic adaptive immune system | Nature | Pseudomonas aeruginosa | no | exp | exp | Mortality | Kin | yes | no | 2.200 | 1.60 | 0.670 | 6.90000e-01 | 6.000 | 48.000 | 0.259 | 0.075 |
| 62 | van Houte, S; Ekroth, AKE; Broniewski, JM; Chabas, H; Ashby, B; Bondy-Denomy, J; Gandon, S; Boots, M; Paterson, S; Buckling, A; Westra, ER | 2016 | The diversity-generating benefits of a prokaryotic adaptive immune system | Nature | Pseudomonas aeruginosa | yes | exp | exp | Mortality | Kin | yes | no | 2.500 | 0.20 | 0.520 | 1.70000e-01 | 6.000 | 48.000 | 1.318 | 0.080 |
| 62 | van Houte, S; Ekroth, AKE; Broniewski, JM; Chabas, H; Ashby, B; Bondy-Denomy, J; Gandon, S; Boots, M; Paterson, S; Buckling, A; Westra, ER | 2016 | The diversity-generating benefits of a prokaryotic adaptive immune system | Nature | Pseudomonas aeruginosa | yes | exp | exp | Parasitism | Kin | yes | no | 1000.000 | 31622777.00 | 476.450 | 3.41945e+07 | 6.000 | 48.000 | 0.730 | 0.076 |
| 62 | van Houte, S; Ekroth, AKE; Broniewski, JM; Chabas, H; Ashby, B; Bondy-Denomy, J; Gandon, S; Boots, M; Paterson, S; Buckling, A; Westra, ER | 2016 | The diversity-generating benefits of a prokaryotic adaptive immune system | Nature | Pseudomonas aeruginosa | no | exp | exp | Mortality | Kin | yes | no | 1.220 | 1.20 | 0.180 | 5.20000e-01 | 6.000 | 48.000 | 0.988 | 0.086 |
| 63 | SERA, WE; GAINES, MS | 1994 | The Effect of Relatedness on Spacing Behavior and Fitness of Female Prairie Voles | Ecology | Microtus ochrogaster | yes | obs | exp | Mortality | Kin | no | no | 0.660 | 0.54 | 0.220 | 3.40000e-01 | 6.000 | 6.000 | 0.636 | 0.088 |
| 63 | SERA, WE; GAINES, MS | 1994 | The Effect of Relatedness on Spacing Behavior and Fitness of Female Prairie Voles | Ecology | Microtus ochrogaster | yes | obs | exp | Mortality | Kin | no | no | 0.680 | 0.72 | 0.320 | 3.40000e-01 | 6.000 | 6.000 | 0.003 | 0.095 |
| 66 | Solazzo, G; Moritz, RFA; Settele, J | 2014 | The social parasite Phengaris (Maculinea) nausithous affects genetic diversity within Myrmica rubra host ant colonies | Journal of Insect Conservation | Myrmica rubra | yes | obs | obs | Parasitism | Kin | no | no | 0.481 | 0.34 | 0.080 | 5.00000e-02 | 3.000 | 6.000 | -0.273 | 0.276 |
| 66 | Solazzo, G; Moritz, RFA; Settele, J | 2014 | The social parasite Phengaris (Maculinea) nausithous affects genetic diversity within Myrmica rubra host ant colonies | Journal of Insect Conservation | Myrmica rubra | yes | obs | obs | Parasitism | Kin | no | no | 2.401 | 2.41 | 1.010 | 1.20000e+00 | 12.000 | 24.000 | 0.145 | 0.036 |
| 68 | Baer, B; Schmid-Hempel, P | 2001 | Unexpected consequences of Polyandrous for parasitism and fitness in the bumblebee, Bombus terrestris | Evolution | Bombus terrestris | yes | obs | exp | Parasitism | Kin | no | no | 61.000 | 75.00 | 31.620 | 3.39400e+01 | 3.333 | 2.667 | -0.050 | 0.249 |
| 68 | Baer, B; Schmid-Hempel, P | 2001 | Unexpected consequences of Polyandrous for parasitism and fitness in the bumblebee, Bombus terrestris | Evolution | Bombus terrestris | yes | obs | exp | Parasitism | Kin | no | no | 0.545 | 0.68 | 0.190 | 1.60000e-01 | 5.667 | 2.667 | -0.200 | 0.251 |
| 68 | Baer, B; Schmid-Hempel, P | 2001 | Unexpected consequences of Polyandrous for parasitism and fitness in the bumblebee, Bombus terrestris | Evolution | Bombus terrestris | yes | obs | exp | Parasitism | Kin | no | no | 75.000 | 76.00 | 33.940 | 2.12100e+01 | 2.667 | 6.000 | -0.683 | 0.258 |
| 68 | Baer, B; Schmid-Hempel, P | 2001 | Unexpected consequences of Polyandrous for parasitism and fitness in the bumblebee, Bombus terrestris | Evolution | Bombus terrestris | yes | obs | exp | Parasitism | Kin | no | no | 0.545 | 0.68 | 0.190 | 1.60000e-01 | 5.667 | 6.000 | -0.400 | 0.130 |
| 68 | Baer, B; Schmid-Hempel, P | 2001 | Unexpected consequences of Polyandrous for parasitism and fitness in the bumblebee, Bombus terrestris | Evolution | Bombus terrestris | yes | obs | exp | Parasitism | Kin | no | no | 0.545 | 0.63 | 0.190 | 2.80000e-01 | 5.667 | 3.333 | 0.350 | 0.142 |
| 68 | Baer, B; Schmid-Hempel, P | 2001 | Unexpected consequences of Polyandrous for parasitism and fitness in the bumblebee, Bombus terrestris | Evolution | Bombus terrestris | yes | obs | exp | Parasitism | Kin | no | no | 61.000 | 76.00 | 31.620 | 2.12100e+01 | 3.333 | 6.000 | -0.733 | 0.199 |
| 68 | Baer, B; Schmid-Hempel, P | 2001 | Unexpected consequences of Polyandrous for parasitism and fitness in the bumblebee, Bombus terrestris | Evolution | Bombus terrestris | yes | obs | exp | Parasitism | Kin | no | no | 0.625 | 0.68 | 0.280 | 1.60000e-01 | 3.333 | 6.000 | -0.758 | 0.204 |
| 68 | Baer, B; Schmid-Hempel, P | 2001 | Unexpected consequences of Polyandrous for parasitism and fitness in the bumblebee, Bombus terrestris | Evolution | Bombus terrestris | yes | obs | exp | Parasitism | Kin | no | no | 0.625 | 0.68 | 0.280 | 1.60000e-01 | 3.333 | 2.667 | -0.558 | 0.326 |
| 68 | Baer, B; Schmid-Hempel, P | 2001 | Unexpected consequences of Polyandrous for parasitism and fitness in the bumblebee, Bombus terrestris | Evolution | Bombus terrestris | yes | obs | exp | Parasitism | Kin | no | no | 0.675 | 0.68 | 0.160 | 1.60000e-01 | 2.667 | 6.000 | -0.207 | 0.288 |
| 68 | Baer, B; Schmid-Hempel, P | 2001 | Unexpected consequences of Polyandrous for parasitism and fitness in the bumblebee, Bombus terrestris | Evolution | Bombus terrestris | yes | obs | exp | Parasitism | Kin | no | no | 47.500 | 76.00 | 24.740 | 2.12100e+01 | 5.667 | 6.000 | -0.631 | 0.124 |
| 68 | Baer, B; Schmid-Hempel, P | 2001 | Unexpected consequences of Polyandrous for parasitism and fitness in the bumblebee, Bombus terrestris | Evolution | Bombus terrestris | yes | obs | exp | Parasitism | Kin | no | no | 47.500 | 61.00 | 24.740 | 3.16200e+01 | 5.667 | 3.333 | 0.102 | 0.129 |
| 68 | Baer, B; Schmid-Hempel, P | 2001 | Unexpected consequences of Polyandrous for parasitism and fitness in the bumblebee, Bombus terrestris | Evolution | Bombus terrestris | yes | obs | exp | Parasitism | Kin | no | no | 47.500 | 75.00 | 24.740 | 3.39400e+01 | 5.667 | 2.667 | 0.052 | 0.174 |
| 70 | Bensch, H; Cornwallis, C. K. | 2017 | NA | NA | Struthio camelus | yes | obs | exp | Mortality | Non-kin | no | no | 0.071 | 0.13 | 0.140 | 2.10000e-01 | 7.000 | 6.000 | -0.177 | 0.550 |
| 74 | Strauss, Alexander; Hite, Jessica; Shocket, Marta; Caceres, Carla; Duffy, Meghan; Hall, Spencer | 2017 | Rapid evolution rescues hosts from competition and disease butdespite a dilution effectincreases the density of infected hosts | Proc. R. Soc. Lond. B | Daphnia dentifera | no | exp | exp | Mortality | Kin | yes | no | 4.000 | 4.70 | 0.560 | 4.50000e-01 | 5.000 | 5.000 | -0.380 | 0.204 |
| 74 | Strauss, Alexander; Hite, Jessica; Shocket, Marta; Caceres, Carla; Duffy, Meghan; Hall, Spencer | 2017 | Rapid evolution rescues hosts from competition and disease butdespite a dilution effectincreases the density of infected hosts | Proc. R. Soc. Lond. B | Daphnia dentifera | no | exp | exp | Mortality | Kin | yes | no | 3.300 | 3.50 | 0.220 | 1.12000e+00 | 5.000 | 5.000 | 1.569 | 0.166 |
| 74 | Strauss, Alexander; Hite, Jessica; Shocket, Marta; Caceres, Carla; Duffy, Meghan; Hall, Spencer | 2017 | Rapid evolution rescues hosts from competition and disease butdespite a dilution effectincreases the density of infected hosts | Proc. R. Soc. Lond. B | Daphnia dentifera | yes | exp | exp | Mortality | Kin | yes | no | 3.280 | 2.30 | 0.220 | 1.79000e+00 | 5.000 | 5.000 | 2.451 | 0.133 |
| 74 | Strauss, Alexander; Hite, Jessica; Shocket, Marta; Caceres, Carla; Duffy, Meghan; Hall, Spencer | 2017 | Rapid evolution rescues hosts from competition and disease butdespite a dilution effectincreases the density of infected hosts | Proc. R. Soc. Lond. B | Daphnia dentifera | yes | exp | exp | Mortality | Kin | yes | no | 2.400 | 1.10 | 1.340 | 2.24000e+00 | 5.000 | 5.000 | 1.294 | 0.451 |
| 76 | Abdi, Mohamed Khadar and Lupi, Daniela and Hardy, Ian C. W | 2020 | Co-Foundress Confinement Elicits Kinship Effects in a Naturally Sub-Social Parasitoid | Journal of Evolutionary Biology | Goniozus nephantidis | no | exp | exp | Mortality | Kin | yes | no | 0.800 | 0.95 | 0.262 | 8.39000e-01 | 110.000 | 110.000 | 0.991 | 0.004 |
| 77 | Mott, CL; Dzaferbegovic, H; Timm, SR; Whiteman, HH | 2019 | Influences of facultative paedomorphosis on kin selection in a larval salamander, Ambystoma talpoideum | Behavior | Ambystoma talpoideum | yes | obs | exp | Mortality | Non-kin | yes | no | 0.620 | 0.92 | 0.542 | 5.81000e-01 | 15.000 | 15.000 | -0.326 | 0.046 |

## **Table S5**: The overall effect of within-group relatedness on mortality and pathogen abundance on Zr.

| Fixed Effects | Posterior Mode (CI) | pMCMC |
| --- | --- | --- |
| Intercept | 0.062 (-0.119, 0.26) | 0.402 |
| Random Effects | Posterior Mode (CI) | I2 % (CI) |
| Species | 0.059 (0.001, 0.13) | 34.455 (2.2, 58.032) |
| Study | 0.003 (0, 0.066) | 10.552 (0.131, 33.555) |
| Phylogeny | 0.002 (0, 0.078) | 8.195 (0.107, 31.591) |
| Residual | 0.063 (0.044, 0.092) | 34.746 (21.133, 51.26) |
| Sampling variance | 0.023 | 11.58 |

## **Table S6**: The effect of pathogen presence and whether mortaility or pathogen abundance was measured on Zr.

| Fixed Effects | Posterior Mode (CI) | pMCMC |
| --- | --- | --- |
| no\_pathogens: mortality | -0.161 (-0.348, 0.084) | 0.246 |
| yes\_pathogens: mortality | 0.125 (-0.092, 0.348) | 0.124 |
| yes\_pathogens: pathogen abundance | 0.096 (-0.1, 0.329) | 0.306 |
| no\_pathogens: mortality vs yes\_pathogens: mortality | -0.286 (-0.439, -0.117) | 0.002 |
| yes\_pathogens: mortality vs yes\_pathogens: pathogen abundance | 0.014 (-0.1, 0.194) | 0.512 |
| Random Effects | Posterior Mode (CI) | I2 % (CI) |
| Species | 0.07 (0.007, 0.161) | 39.806 (7.325, 66.238) |
| Study | 0.002 (0, 0.053) | 8.115 (0.07, 28.552) |
| Phylogeny | 0.002 (0, 0.09) | 9.239 (0.09, 37.216) |
| Residual | 0.055 (0.04, 0.085) | 30.841 (16.117, 48.788) |
| Sampling variance | 0.023 | 11.442 |

## **Table S7**: The effect of pathogen manipulation on Zr.

| Fixed Effects | Posterior Mode (CI) | pMCMC |
| --- | --- | --- |
| Exp no\_pathogens | -0.233 (-0.494, 0.033) | 0.108 |
| Exp yes\_pathogens | 0.166 (-0.093, 0.376) | 0.148 |
| Obs no\_pathogens | 0.1 (-0.114, 0.5) | 0.264 |
| Obs yes\_pathogens | 0.059 (-0.095, 0.273) | 0.356 |
| Exp no\_pathogens vs Exp yes\_pathogens | -0.396 (-0.568, -0.212) | 0.001 |
| Exp no\_pathogens vs Obs no\_pathogens | -0.255 (-0.705, -0.053) | 0.03 |
| Exp yes\_pathogens vs Obs yes\_pathogens | 0.094 (-0.152, 0.29) | 0.444 |
| Obs no\_pathogens vs Obs yes\_pathogens | 0.082 (-0.21, 0.383) | 0.574 |
| Random Effects | Posterior Mode (CI) | I2 % (CI) |
| Species | 0.081 (0.01, 0.144) | 37.546 (10.081, 64.581) |
| Study | 0.001 (0, 0.053) | 8.832 (0.106, 30.316) |
| Phylogeny | 0.001 (0, 0.078) | 7.961 (0.137, 33.2) |
| Residual | 0.057 (0.039, 0.084) | 32.875 (16.189, 47.369) |
| Sampling variance | 0.023 | 12.282 |

## **Table S8**: The effect of relatedness manipulations and pathogen presence on Zr.

| Fixed Effects | Posterior Mode (CI) | pMCMC |
| --- | --- | --- |
| Exp relatedness: no\_pathogens | -0.05 (-0.301, 0.175) | 0.552 |
| Exp relatedness: yes\_pathogens | 0.134 (-0.067, 0.362) | 0.09 |
| Obs relatedness: no\_pathogens | -0.32 (-0.679, 0.04) | 0.072 |
| Obs relatedness: yes\_pathogens | 0.038 (-0.187, 0.256) | 0.51 |
| Exp relatedness: no\_pathogens vs Exp relatedness: yes\_pathogens | -0.191 (-0.418, -0.075) | 0.01 |
| Exp relatedness: no\_pathogens vs Obs relatedness: no\_pathogens | 0.285 (-0.079, 0.624) | 0.162 |
| Exp relatedness: yes\_pathogens vs Obs relatedness: yes\_pathogens | 0.075 (-0.062, 0.275) | 0.194 |
| Obs relatedness: no\_pathogens vs Obs relatedness: yes\_pathogens | -0.303 (-0.767, -0.101) | 0.022 |
| Random Effects | Posterior Mode (CI) | I2 % (CI) |
| Species | 0.069 (0.02, 0.17) | 42.789 (15.612, 67.698) |
| Study | 0.001 (0, 0.043) | 6.581 (0.137, 22.734) |
| Phylogeny | 0.002 (0, 0.088) | 9.188 (0.125, 35.694) |
| Residual | 0.057 (0.037, 0.081) | 29.617 (15.969, 44.109) |
| Sampling variance | 0.023 | 11.327 |

## **Table S9**: The effect of kin structure and pathogen presence measures on Zr.

| Fixed Effects | Posterior Mode (CI) | pMCMC |
| --- | --- | --- |
| Kin: exp no\_pathogens | -0.303 (-0.551, -0.034) | 0.036 |
| Non-kin: exp no\_pathogens | 0.016 (-0.814, 0.77) | 0.98 |
| Kin: exp yes\_pathogens | 0.077 (-0.15, 0.361) | 0.53 |
| Non-kin: exp yes\_pathogens | 0.608 (-0.146, 1.272) | 0.122 |
| Kin: obs no\_pathogens | -0.121 (-0.54, 0.293) | 0.43 |
| Non-kin: obs no\_pathogens | 0.392 (-0.011, 0.826) | 0.042 |
| Kin: obs yes\_pathogens | 0.113 (-0.118, 0.285) | 0.354 |
| Non-kin: obs yes\_pathogens | 0.016 (-0.285, 0.355) | 0.846 |
| Kin: exp no\_pathogens vs Kin: exp yes\_pathogens | -0.333 (-0.534, -0.157) | 0.001 |
| Kin: obs no\_pathogens vs Non-kin: obs no\_pathogens | -0.568 (-1.108, 0.024) | 0.034 |
| Kin: obs yes\_pathogens vs Non-kin: obs yes\_pathogens | 0.09 (-0.313, 0.373) | 0.79 |
| Random Effects | Posterior Mode (CI) | I2 % (CI) |
| Species | 0.055 (0.002, 0.123) | 37.022 (9.391, 61.97) |
| Study | 0.001 (0, 0.049) | 8.136 (0.174, 27.856) |
| Phylogeny | 0.002 (0, 0.063) | 7.599 (0.163, 30.536) |
| Residual | 0.059 (0.038, 0.083) | 33.957 (17.962, 48.679) |
| Sampling variance | 0.023 | 12.824 |

## **Table S10**: The overall effect of within-group relatedness on variance (LnCVR) mortality and pathogen abundances.

| Fixed Effects | Posterior Mode (CI) | pMCMC |
| --- | --- | --- |
| Intercept | 0.077 (-0.259, 0.43) | 0.61 |
| Random Effects | Posterior Mode (CI) | I2 % (CI) |
| Species | 0.003 (0, 0.178) | 6.462 (0.022, 22.94) |
| Study | 0.002 (0, 0.139) | 4.532 (0.03, 17.733) |
| Phylogeny | 0.003 (0, 0.22) | 5.653 (0.032, 23.504) |
| Residual | 0.527 (0.401, 0.795) | 76.597 (56.433, 93.06) |
| Sampling variance | 0.051 | 6.548 |

## **Table S11**: The effect of pathogen manipulation on LnCVR.

| Fixed Effects | Posterior Mode (CI) | pMCMC |
| --- | --- | --- |
| Exp no\_pathogens | 0.075 (-0.487, 0.698) | 0.666 |
| Exp yes\_pathogens | 0.156 (-0.295, 0.74) | 0.424 |
| Obs no\_pathogens | 0.246 (-0.408, 0.846) | 0.358 |
| Obs yes\_pathogens | -0.09 (-0.501, 0.42) | 0.788 |
| Exp no\_pathogens vs Exp yes\_pathogens | -0.172 (-0.612, 0.487) | 0.784 |
| Exp no\_pathogens vs Obs no\_pathogens | -0.435 (-0.93, 0.604) | 0.666 |
| Exp yes\_pathogens vs Obs yes\_pathogens | 0.232 (-0.261, 0.773) | 0.348 |
| Obs no\_pathogens vs Obs yes\_pathogens | 0.221 (-0.345, 1.03) | 0.332 |
| Random Effects | Posterior Mode (CI) | I2 % (CI) |
| Species | 0.002 (0, 0.231) | 7.696 (0.016, 25.768) |
| Study | 0.002 (0, 0.154) | 4.776 (0.019, 18.904) |
| Phylogeny | 0.003 (0, 0.217) | 5.427 (0.029, 22.524) |
| Residual | 0.559 (0.398, 0.808) | 75.572 (52.767, 92.226) |
| Sampling variance | 0.051 | 6.269 |

## **Table S12**: The effect of pathogen manipulation and whether mortaility or pathogen abundance was measured on LnCVR.

| Fixed Effects | Posterior Mode (CI) | pMCMC |
| --- | --- | --- |
| Exp no\_pathogens: mortality | 0.206 (-0.33, 0.876) | 0.39 |
| Exp yes\_pathogens: mortality | 0.884 (0.213, 1.413) | 0.018 |
| Obs no\_pathogens: mortality | 0.435 (-0.318, 0.939) | 0.322 |
| Obs yes\_pathogens: mortality | -0.029 (-0.735, 0.648) | 0.928 |
| Exp yes\_pathogens: pathogen abundance | -0.342 (-0.952, 0.218) | 0.202 |
| Obs yes\_pathogens: pathogen abundance | -0.151 (-0.708, 0.495) | 0.832 |
| Exp no\_pathogens: mortality vs Exp yes\_pathogens: mortality | -0.729 (-1.107, 0.071) | 0.074 |
| Exp no\_pathogens: mortality vs Obs no\_pathogens: mortality | 0.029 (-0.776, 0.675) | 0.884 |
| Exp yes\_pathogens: mortality vs Obs yes\_pathogens: mortality | 1.05 (0.033, 1.629) | 0.056 |
| Exp yes\_pathogens: mortality vs Exp yes\_pathogens: pathogen abundance | 1.18 (0.557, 1.88) | 0.001 |
| Obs no\_pathogens: mortality vs Obs yes\_pathogens: mortality | 0.35 (-0.528, 1.155) | 0.438 |
| Obs yes\_pathogens: mortality vs Obs yes\_pathogens: pathogen abundance | 0.228 (-0.792, 0.85) | 0.944 |
| Random Effects | Posterior Mode (CI) | I2 % (CI) |
| Species | 0.003 (0, 0.243) | 9.325 (0.035, 29.815) |
| Study | 0.002 (0, 0.22) | 7.547 (0.02, 27.443) |
| Phylogeny | 0.004 (0, 0.393) | 9.158 (0.054, 39.271) |
| Residual | 0.484 (0.326, 0.702) | 67.109 (41.962, 86.907) |
| Sampling variance | 0.051 | 6.539 |

## **Table S13**: The effect of relatedness manipulations and pathogen presence on LnCVR.

| Fixed Effects | Posterior Mode (CI) | pMCMC |
| --- | --- | --- |
| Exp relatedness: no\_pathogens | 0.127 (-0.319, 0.591) | 0.518 |
| Exp relatedness: yes\_pathogens | -0.037 (-0.329, 0.476) | 0.87 |
| Obs relatedness: no\_pathogens | 0.536 (-1.344, 1.923) | 0.808 |
| Obs relatedness: yes\_pathogens | 0.246 (-0.685, 0.91) | 0.65 |
| Exp relatedness: no\_pathogens vs Exp relatedness: yes\_pathogens | 0.089 (-0.325, 0.538) | 0.65 |
| Exp relatedness: no\_pathogens vs Obs relatedness: no\_pathogens | -0.106 (-1.651, 1.579) | 0.938 |
| Exp relatedness: yes\_pathogens vs Obs relatedness: yes\_pathogens | -0.304 (-0.919, 0.639) | 0.742 |
| Obs relatedness: no\_pathogens vs Obs relatedness: yes\_pathogens | 0.254 (-1.553, 1.958) | 0.952 |
| Random Effects | Posterior Mode (CI) | I2 % (CI) |
| Species | 0.003 (0, 0.191) | 6.622 (0.029, 22.545) |
| Study | 0.002 (0, 0.124) | 3.676 (0.022, 14.588) |
| Phylogeny | 0.003 (0, 0.231) | 5.641 (0.023, 24.262) |
| Residual | 0.623 (0.42, 0.837) | 77.513 (55.206, 93.229) |
| Sampling variance | 0.051 | 6.3 |

# Verification analyses

## **Table S14**: The effect of kin structure and pathogen presence measures on Zr excluding potential effects of inbreeding.

| Fixed Effects | Posterior Mode (CI) | pMCMC |
| --- | --- | --- |
| Kin: exp no\_pathogens | -0.223 (-0.546, 0.007) | 0.06 |
| Kin: exp yes\_pathogens | 0.131 (-0.16, 0.38) | 0.458 |
| Kin: obs no\_pathogens | -0.24 (-0.586, 0.284) | 0.538 |
| Non-kin: obs no\_pathogens | 0.335 (-0.08, 0.831) | 0.13 |
| Kin: Obs yes\_pathogens | 0.08 (-0.091, 0.348) | 0.228 |
| Non-kin: obs yes\_pathogens | 0.071 (-0.358, 0.539) | 0.748 |
| Kin: exp no\_pathogens vs Kin: exp yes\_pathogens | -0.377 (-0.577, -0.17) | 0.002 |
| Kin: obs no\_pathogens vs Non-kin: obs no\_pathogens | -0.468 (-1.127, 0.091) | 0.11 |
| Random Effects | Posterior Mode (CI) | I2 % (CI) |
| Species | 0.062 (0.001, 0.156) | 39.669 (9.491, 67.337) |
| Study | 0.001 (0, 0.044) | 6.805 (0.1, 23.566) |
| Phylogeny | 0.002 (0, 0.083) | 9.206 (0.121, 35.301) |
| Residual | 0.065 (0.038, 0.084) | 32.141 (17.612, 48.55) |
| Sampling variance | 0.023 | 11.604 |

## **Table S15**: Effect of parasite manipulations and fitness measures on LnCVR excluding potential effects of inbreeding.

| Fixed Effects | Posterior Mode (CI) | pMCMC |
| --- | --- | --- |
| Exp no\_pathogens: mortality | 0.105 (-0.342, 0.919) | 0.388 |
| Exp yes\_pathogens: mortality | 0.597 (0.181, 1.463) | 0.008 |
| Obs no\_pathogens: mortality | 0.289 (-0.289, 1.061) | 0.27 |
| Obs yes\_pathogens: mortality | -0.093 (-0.706, 0.623) | 0.966 |
| Exp yes\_pathogens: pathogen abundance | -0.472 (-0.976, 0.359) | 0.222 |
| Obs yes\_pathogens: pathogen abundance | -0.146 (-0.687, 0.627) | 0.814 |
| Exp no\_pathogens: mortality vs Exp yes\_pathogens: mortality | -0.605 (-1.129, 0.031) | 0.054 |
| Exp no\_pathogens: mortality vs Obs no\_pathogens: mortality | -0.076 (-0.886, 0.675) | 0.838 |
| Exp yes\_pathogens: mortality vs Obs yes\_pathogens: mortality | 0.719 (0.029, 1.681) | 0.044 |
| Exp yes\_pathogens: mortality vs Exp yes\_pathogens: pathogen abundance | 1.142 (0.512, 1.769) | 0.002 |
| Obs no\_pathogens: mortality vs Obs yes\_pathogens: mortality | 0.413 (-0.483, 1.231) | 0.424 |
| Obs yes\_pathogens: mortality vs Obs yes\_pathogens: pathogen abundance | 0.112 (-0.763, 0.843) | 0.868 |
| Random Effects | Posterior Mode (CI) | I2 % (CI) |
| Species | 0.003 (0, 0.263) | 10.043 (0.036, 32.755) |
| Study | 0.001 (0, 0.209) | 7.637 (0.044, 25.214) |
| Phylogeny | 0.003 (0, 0.418) | 9.422 (0.023, 38.973) |
| Residual | 0.45 (0.309, 0.675) | 66.017 (41.762, 88.968) |
| Sampling variance | 0.051 | 6.543 |

## **Table S16**: Laboratory efffects on Zr.

| Fixed Effects | Posterior Mode (CI) | pMCMC |
| --- | --- | --- |
| Laboratory | 0.061 (-0.085, 0.269) | 0.342 |
| Field | 0.148 (-0.196, 0.383) | 0.488 |
| Laboratory vs Field | -0.004 (-0.295, 0.288) | 0.844 |
| Random Effects | Posterior Mode (CI) | I2 % (CI) |
| Species | 0.096 (0.001, 0.164) | 50.576 (12.194, 82.549) |
| Study | 0.011 (0, 0.08) | 17.998 (0.289, 52.223) |
| Phylogeny | 0.002 (0, 0.055) | 7.081 (0.131, 27.843) |
| Residual | 0.01 (0.002, 0.029) | 9.226 (1.165, 20.317) |
| Sampling variance | 0.023 | 14.194 |

## **Table S17**: Laboratory efffects on LnCVR

| Fixed Effects | Posterior Mode (CI) | pMCMC |
| --- | --- | --- |
| Laboratory | -0.072 (-0.479, 0.369) | 0.738 |
| Field | 0.156 (-0.357, 0.673) | 0.448 |
| Laboratory vs Field | -0.37 (-0.743, 0.318) | 0.382 |
| Random Effects | Posterior Mode (CI) | I2 % (CI) |
| Species | 0.002 (0, 0.209) | 11.336 (0.063, 43.359) |
| Study | 0.004 (0, 0.288) | 17.956 (0.136, 57.011) |
| Phylogeny | 0.001 (0, 0.223) | 9.13 (0.058, 37.451) |
| Residual | 0.192 (0.045, 0.315) | 47.807 (13.206, 80.994) |
| Sampling variance | 0.051 | 12.266 |

## **Table S18**: The sensitivity of analyses on Zr to the statistical techiques used in primary studies.

| Fixed Effects | Posterior Mode (CI) | pMCMC |
| --- | --- | --- |
| Exp no\_pathogens | -0.1 (-0.459, 0.091) | 0.206 |
| Exp yes\_pathogens | 0.168 (-0.057, 0.447) | 0.132 |
| Obs no\_pathogens | 0.227 (-0.135, 0.503) | 0.278 |
| Obs yes\_pathogens | 0.085 (-0.118, 0.287) | 0.318 |
| Exp no\_pathogens vs Exp yes\_pathogens | -0.423 (-0.565, -0.197) | 0.001 |
| Exp no\_pathogens vs Obs no\_pathogens | -0.37 (-0.669, 0.029) | 0.052 |
| Exp yes\_pathogens vs Obs yes\_pathogens | 0.075 (-0.119, 0.311) | 0.44 |
| Obs no\_pathogens vs Obs yes\_pathogens | 0.088 (-0.23, 0.387) | 0.604 |
| Random Effects | Posterior Mode (CI) | I2 % (CI) |
| Species | 0.06 (0.009, 0.148) | 38.949 (11.921, 63.201) |
| Study | 0.002 (0, 0.044) | 6.865 (0.1, 24.084) |
| Phylogeny | 0.002 (0, 0.062) | 7.082 (0.104, 28.364) |
| Analysis technique | 0.003 (0, 0.036) | 6.137 (0.133, 16.204) |
| Residual | 0.054 (0.034, 0.078) | 28.757 (14.833, 43.253) |
| Sampling variance | 0.023 | 11.699 |

# Unpublished data included in analyses

## **Table S19**: Data on within-group relatedness on mortality in ostrich chicks.

| year | relatedness | group | number\_dead | precentage\_dead |
| --- | --- | --- | --- | --- |
| 2016 | 0.08 | 1 | 1 | 0.125 |
| 2016 | 0.10 | 10 | 0 | 0.000 |
| 2016 | 0.04 | 11 | 1 | 0.125 |
| 2016 | 0.27 | 12 | 3 | 0.375 |
| 2016 | 0.09 | 13 | 0 | 0.000 |
| 2016 | 0.00 | 14 | 0 | 0.000 |
| 2016 | 0.14 | 15 | 1 | 0.125 |
| 2016 | 0.02 | 2 | 1 | 0.125 |
| 2016 | 0.23 | 3 | 1 | 0.125 |
| 2016 | 0.07 | 4 | 4 | 0.500 |
| 2016 | 0.23 | 5 | 0 | 0.000 |
| 2016 | 0.14 | 6 | 1 | 0.125 |
| 2016 | 0.00 | 7 | 0 | 0.000 |
| 2016 | 0.04 | 8 | 0 | 0.000 |
| 2016 | 0.02 | 9 | 1 | 0.125 |
| 2017 | 0.11 | 1 | 1 | 0.125 |
| 2017 | 0.04 | 10 | 1 | 0.125 |
| 2017 | 0.09 | 11 | 1 | 0.125 |
| 2017 | 0.08 | 12 | 2 | 0.250 |
| 2017 | 0.08 | 13 | 1 | 0.125 |
| 2017 | 0.06 | 14 | 2 | 0.250 |
| 2017 | 0.03 | 15 | 0 | 0.000 |
| 2017 | 0.05 | 2 | 0 | 0.000 |
| 2017 | 0.08 | 3 | 3 | 0.375 |
| 2017 | 0.25 | 4 | 0 | 0.000 |
| 2017 | 0.15 | 5 | 2 | 0.250 |
| 2017 | 0.25 | 6 | 0 | 0.000 |
| 2017 | 0.15 | 7 | 1 | 0.125 |
| 2017 | 0.02 | 8 | 4 | 0.500 |
| 2017 | 0.17 | 9 | 0 | 0.000 |
| 2018 | 0.03 | 1 | 2 | 0.250 |
| 2018 | 0.11 | 10 | 0 | 0.000 |
| 2018 | 0.02 | 11 | 0 | 0.000 |
| 2018 | 0.15 | 12 | 0 | 0.000 |
| 2018 | 0.08 | 13 | 2 | 0.250 |
| 2018 | 0.15 | 14 | 3 | 0.375 |
| 2018 | 0.09 | 15 | 2 | 0.250 |
| 2018 | 0.17 | 2 | 2 | 0.250 |
| 2018 | 0.08 | 3 | 1 | 0.125 |
| 2018 | 0.05 | 4 | 0 | 0.000 |
| 2018 | 0.06 | 5 | 0 | 0.000 |
| 2018 | 0.21 | 6 | 0 | 0.000 |
| 2018 | 0.23 | 7 | 0 | 0.000 |
| 2018 | 0.04 | 8 | 1 | 0.125 |
| 2018 | 0.08 | 9 | 1 | 0.125 |

# Session information

```
devtools::session_info()
```

```
## ─ Session info ───────────────────────────────────────────────────────────────
##  setting  value                       
##  version  R version 4.0.2 (2020-06-22)
##  os       macOS  10.16                
##  system   x86_64, darwin17.0          
##  ui       X11                         
##  language (EN)                        
##  collate  en_GB.UTF-8                 
##  ctype    en_GB.UTF-8                 
##  tz       Europe/Stockholm            
##  date     2021-06-14                  
## 
## ─ Packages ───────────────────────────────────────────────────────────────────
##  package              * version date       lib source        
##  abind                  1.4-5   2016-07-21 [1] CRAN (R 4.0.2)
##  ape                  * 5.4-1   2020-08-13 [1] CRAN (R 4.0.2)
##  arm                  * 1.11-2  2020-07-27 [1] CRAN (R 4.0.2)
##  arsenal              * 3.5.0   2020-07-13 [1] CRAN (R 4.0.2)
##  assertthat             0.2.1   2019-03-21 [1] CRAN (R 4.0.2)
##  backports              1.2.0   2020-11-02 [1] CRAN (R 4.0.2)
##  base64enc              0.1-3   2015-07-28 [1] CRAN (R 4.0.2)
##  BiocManager            1.30.10 2019-11-16 [1] CRAN (R 4.0.2)
##  bitops                 1.0-6   2013-08-17 [1] CRAN (R 4.0.2)
##  boot                 * 1.3-25  2020-04-26 [1] CRAN (R 4.0.2)
##  broom                  0.7.2   2020-10-20 [1] CRAN (R 4.0.2)
##  callr                  3.5.1   2020-10-13 [1] CRAN (R 4.0.2)
##  car                  * 3.0-10  2020-09-29 [1] CRAN (R 4.0.2)
##  carData              * 3.0-4   2020-05-22 [1] CRAN (R 4.0.2)
##  cellranger             1.1.0   2016-07-27 [1] CRAN (R 4.0.2)
##  checkmate              2.0.0   2020-02-06 [1] CRAN (R 4.0.2)
##  cli                    2.2.0   2020-11-20 [1] CRAN (R 4.0.2)
##  cluster                2.1.0   2019-06-19 [1] CRAN (R 4.0.2)
##  coda                 * 0.19-4  2020-09-30 [1] CRAN (R 4.0.2)
##  colorspace             2.0-0   2020-11-11 [1] CRAN (R 4.0.2)
##  corpcor                1.6.9   2017-04-01 [1] CRAN (R 4.0.2)
##  cowplot              * 1.1.0   2020-09-08 [1] CRAN (R 4.0.2)
##  crayon                 1.3.4   2017-09-16 [1] CRAN (R 4.0.2)
##  cubature               2.0.4.1 2020-07-06 [1] CRAN (R 4.0.2)
##  curl                   4.3     2019-12-02 [1] CRAN (R 4.0.1)
##  data.table           * 1.13.2  2020-10-19 [1] CRAN (R 4.0.2)
##  DBI                    1.1.0   2019-12-15 [1] CRAN (R 4.0.2)
##  dbplyr                 2.0.0   2020-11-03 [1] CRAN (R 4.0.2)
##  Deriv                  4.1.0   2020-10-02 [1] CRAN (R 4.0.2)
##  desc                   1.2.0   2018-05-01 [1] CRAN (R 4.0.2)
##  devtools               2.3.2   2020-09-18 [1] CRAN (R 4.0.2)
##  DiagrammeR           * 1.0.6.1 2020-05-08 [1] CRAN (R 4.0.2)
##  DiagrammeRsvg        * 0.1     2016-02-04 [1] CRAN (R 4.0.2)
##  digest                 0.6.27  2020-10-24 [1] CRAN (R 4.0.2)
##  doBy                 * 4.6.7   2020-07-08 [1] CRAN (R 4.0.2)
##  dplyr                * 1.0.2   2020-08-18 [1] CRAN (R 4.0.2)
##  ellipsis               0.3.1   2020-05-15 [1] CRAN (R 4.0.2)
##  evaluate               0.14    2019-05-28 [1] CRAN (R 4.0.1)
##  fansi                  0.4.1   2020-01-08 [1] CRAN (R 4.0.2)
##  farver                 2.0.3   2020-01-16 [1] CRAN (R 4.0.2)
##  forcats              * 0.5.0   2020-03-01 [1] CRAN (R 4.0.2)
##  foreign                0.8-80  2020-05-24 [1] CRAN (R 4.0.2)
##  Formula                1.2-4   2020-10-16 [1] CRAN (R 4.0.2)
##  fs                     1.5.0   2020-07-31 [1] CRAN (R 4.0.2)
##  generics               0.1.0   2020-10-31 [1] CRAN (R 4.0.2)
##  ggforce                0.3.2   2020-06-23 [1] CRAN (R 4.0.2)
##  ggmap                * 3.0.0   2019-02-05 [1] CRAN (R 4.0.2)
##  ggplot2              * 3.3.2   2020-06-19 [1] CRAN (R 4.0.2)
##  ggsci                * 2.9     2018-05-14 [1] CRAN (R 4.0.2)
##  glue                   1.4.2   2020-08-27 [1] CRAN (R 4.0.2)
##  gridExtra              2.3     2017-09-09 [1] CRAN (R 4.0.2)
##  gtable                 0.3.0   2019-03-25 [1] CRAN (R 4.0.2)
##  haven                  2.3.1   2020-06-01 [1] CRAN (R 4.0.2)
##  highr                  0.8     2019-03-20 [1] CRAN (R 4.0.2)
##  Hmisc                  4.4-1   2020-08-10 [1] CRAN (R 4.0.2)
##  hms                    0.5.3   2020-01-08 [1] CRAN (R 4.0.2)
##  htmlTable              2.1.0   2020-09-16 [1] CRAN (R 4.0.2)
##  htmltools              0.5.0   2020-06-16 [1] CRAN (R 4.0.2)
##  htmlwidgets            1.5.3   2020-12-10 [1] CRAN (R 4.0.2)
##  httr                   1.4.2   2020-07-20 [1] CRAN (R 4.0.2)
##  jpeg                   0.1-8.1 2019-10-24 [1] CRAN (R 4.0.2)
##  jsonlite               1.7.1   2020-09-07 [1] CRAN (R 4.0.2)
##  kableExtra           * 1.2.1   2020-08-27 [1] CRAN (R 4.0.2)
##  knitr                * 1.30    2020-09-22 [1] CRAN (R 4.0.2)
##  labeling               0.4.2   2020-10-20 [1] CRAN (R 4.0.2)
##  languageR            * 1.5.0   2019-01-30 [1] CRAN (R 4.0.2)
##  lattice              * 0.20-41 2020-04-02 [1] CRAN (R 4.0.2)
##  latticeExtra           0.6-29  2019-12-19 [1] CRAN (R 4.0.2)
##  lifecycle              0.2.0   2020-03-06 [1] CRAN (R 4.0.2)
##  lme4                 * 1.1-25  2020-10-23 [1] CRAN (R 4.0.2)
##  lubridate              1.7.9.2 2020-11-13 [1] CRAN (R 4.0.2)
##  magick               * 2.5.0   2020-10-16 [1] CRAN (R 4.0.2)
##  magrittr             * 2.0.1   2020-11-17 [1] CRAN (R 4.0.2)
##  MASS                 * 7.3-53  2020-09-09 [1] CRAN (R 4.0.2)
##  Matrix               * 1.2-18  2019-11-27 [1] CRAN (R 4.0.2)
##  MCMCglmm             * 2.32    2021-03-12 [1] CRAN (R 4.0.2)
##  memoise                1.1.0   2017-04-21 [1] CRAN (R 4.0.2)
##  metafor              * 2.4-0   2020-03-19 [1] CRAN (R 4.0.2)
##  mgcv                   1.8-33  2020-08-27 [1] CRAN (R 4.0.2)
##  minqa                  1.2.4   2014-10-09 [1] CRAN (R 4.0.2)
##  modelr                 0.1.8   2020-05-19 [1] CRAN (R 4.0.2)
##  munsell                0.5.0   2018-06-12 [1] CRAN (R 4.0.2)
##  nlme                   3.1-149 2020-08-23 [1] CRAN (R 4.0.2)
##  nloptr                 1.2.2.2 2020-07-02 [1] CRAN (R 4.0.2)
##  nnet                   7.3-14  2020-04-26 [1] CRAN (R 4.0.2)
##  openxlsx             * 4.2.2   2020-09-17 [1] CRAN (R 4.0.2)
##  pacman                 0.5.1   2019-03-11 [1] CRAN (R 4.0.2)
##  PerformanceAnalytics * 2.0.4   2020-02-06 [1] CRAN (R 4.0.2)
##  pillar                 1.4.7   2020-11-20 [1] CRAN (R 4.0.2)
##  pkgbuild               1.1.0   2020-07-13 [1] CRAN (R 4.0.2)
##  pkgconfig              2.0.3   2019-09-22 [1] CRAN (R 4.0.2)
##  pkgload                1.1.0   2020-05-29 [1] CRAN (R 4.0.2)
##  plyr                 * 1.8.6   2020-03-03 [1] CRAN (R 4.0.2)
##  png                    0.1-7   2013-12-03 [1] CRAN (R 4.0.2)
##  polyclip               1.10-0  2019-03-14 [1] CRAN (R 4.0.2)
##  prettyunits            1.1.1   2020-01-24 [1] CRAN (R 4.0.2)
##  processx               3.4.5   2020-11-30 [1] CRAN (R 4.0.2)
##  ps                     1.4.0   2020-10-07 [1] CRAN (R 4.0.2)
##  purrr                * 0.3.4   2020-04-17 [1] CRAN (R 4.0.2)
##  quadprog               1.5-8   2019-11-20 [1] CRAN (R 4.0.2)
##  QuantPsyc            * 1.5     2012-03-20 [1] CRAN (R 4.0.2)
##  R6                     2.5.0   2020-10-28 [1] CRAN (R 4.0.2)
##  RColorBrewer           1.1-2   2014-12-07 [1] CRAN (R 4.0.2)
##  Rcpp                   1.0.5   2020-07-06 [1] CRAN (R 4.0.2)
##  readr                * 1.4.0   2020-10-05 [1] CRAN (R 4.0.2)
##  readxl                 1.3.1   2019-03-13 [1] CRAN (R 4.0.2)
##  remotes                2.2.0   2020-07-21 [1] CRAN (R 4.0.2)
##  reprex                 0.3.0   2019-05-16 [1] CRAN (R 4.0.2)
##  reshape              * 0.8.8   2018-10-23 [1] CRAN (R 4.0.2)
##  RgoogleMaps            1.4.5.3 2020-02-12 [1] CRAN (R 4.0.2)
##  rio                    0.5.16  2018-11-26 [1] CRAN (R 4.0.2)
##  rjson                  0.2.20  2018-06-08 [1] CRAN (R 4.0.2)
##  rlang                  0.4.9   2020-11-26 [1] CRAN (R 4.0.2)
##  rmarkdown              2.5     2020-10-21 [1] CRAN (R 4.0.2)
##  Rmisc                * 1.5     2013-10-22 [1] CRAN (R 4.0.2)
##  rpart                  4.1-15  2019-04-12 [1] CRAN (R 4.0.2)
##  rprojroot              2.0.2   2020-11-15 [1] CRAN (R 4.0.2)
##  rstudioapi             0.13    2020-11-12 [1] CRAN (R 4.0.2)
##  rsvg                 * 2.1     2020-05-19 [1] CRAN (R 4.0.2)
##  rvcheck                0.1.8   2020-03-01 [1] CRAN (R 4.0.2)
##  rvest                  0.3.6   2020-07-25 [1] CRAN (R 4.0.2)
##  scales                 1.1.1   2020-05-11 [1] CRAN (R 4.0.2)
##  scatterpie           * 0.1.5   2020-09-09 [1] CRAN (R 4.0.2)
##  sessioninfo            1.1.1   2018-11-05 [1] CRAN (R 4.0.2)
##  sp                     1.4-4   2020-10-07 [1] CRAN (R 4.0.2)
##  statmod                1.4.35  2020-10-19 [1] CRAN (R 4.0.2)
##  stringdist           * 0.9.6.3 2020-10-09 [1] CRAN (R 4.0.2)
##  stringi                1.5.3   2020-09-09 [1] CRAN (R 4.0.2)
##  stringr              * 1.4.0   2019-02-10 [1] CRAN (R 4.0.2)
##  survival               3.2-7   2020-09-28 [1] CRAN (R 4.0.2)
##  tensorA                0.36.1  2018-07-29 [1] CRAN (R 4.0.2)
##  testthat               3.0.0   2020-10-31 [1] CRAN (R 4.0.2)
##  tibble               * 3.0.4   2020-10-12 [1] CRAN (R 4.0.2)
##  tidyr                * 1.1.2   2020-08-27 [1] CRAN (R 4.0.2)
##  tidyselect             1.1.0   2020-05-11 [1] CRAN (R 4.0.2)
##  tidyverse            * 1.3.0   2019-11-21 [1] CRAN (R 4.0.2)
##  tweenr                 1.0.1   2018-12-14 [1] CRAN (R 4.0.2)
##  usethis                1.6.3   2020-09-17 [1] CRAN (R 4.0.2)
##  V8                     3.4.2   2021-05-01 [1] CRAN (R 4.0.2)
##  vctrs                  0.3.5   2020-11-17 [1] CRAN (R 4.0.2)
##  viridisLite            0.3.0   2018-02-01 [1] CRAN (R 4.0.1)
##  visNetwork             2.0.9   2019-12-06 [1] CRAN (R 4.0.2)
##  webshot                0.5.2   2019-11-22 [1] CRAN (R 4.0.2)
##  wesanderson          * 0.3.6   2018-04-20 [1] CRAN (R 4.0.2)
##  withr                  2.3.0   2020-09-22 [1] CRAN (R 4.0.2)
##  xfun                   0.19    2020-10-30 [1] CRAN (R 4.0.2)
##  xml2                   1.3.2   2020-04-23 [1] CRAN (R 4.0.2)
##  xts                  * 0.12.1  2020-09-09 [1] CRAN (R 4.0.2)
##  yaml                   2.2.1   2020-02-01 [1] CRAN (R 4.0.2)
##  zip                    2.1.1   2020-08-27 [1] CRAN (R 4.0.2)
##  zoo                  * 1.8-8   2020-05-02 [1] CRAN (R 4.0.2)
## 
## [1] /Library/Frameworks/R.framework/Versions/4.0/Resources/library
```
